# Comparative Analysis of Oligosaccharides in Breast Milk and Feces of Breast-Fed Infants by Using LC-QE-HF-MS: A Communication

**DOI:** 10.3390/nu15040888

**Published:** 2023-02-09

**Authors:** Rui Li, Yalin Zhou, Yajun Xu

**Affiliations:** 1 Department of Nutrition and Food Hygiene, School of Public Health, Peking University, No. 38 Xueyuan Road, Beijing 100083, China; 2 Beijing Key Laboratory of Toxicological Research and Risk Assessment for Food Safety, Peking University, No. 38 Xueyuan Road, Beijing 100083, China; 3 PKUHSC-China Feihe Joint Research Institute of Nutrition and Healthy Lifespan Development, No. 38 Xueyuan Road, Beijing 100083, China

**Keywords:** breast milk, baby feces, oligosaccharides

## Abstract

Currently, it must be acknowledged that little is known about the quantity and make-up of oligosaccharides (OS) found in breast-fed babies’ feces as well as their metabolic fate. In the present work, UPLC-QE-HF-MS was successfully adopted to identify the profiles of human milk oligosaccharides (HMOs) in the breast milk of four mothers and fecal OS in the feces of their breast-fed infant. There were significant variations and differences in both number and composition between HMOs and fecal OS. The early-life gastrointestinal microbiota metabolism may be triggered into the advanced breakdown, synthesis, bioconversion, or redesign of HMOs. The fate of HMOs during passage through the gastrointestinal tract may be profoundly informed by the comparison of OS between breast milk and fecal OS profiles. The characterization of fecal OS could be applied as a valuable tool for monitoring the gastrointestinal fate of HMOs and reflecting infant development at different stages of lactation. Further research on the gastrointestinal bioconversion of HMOs profiles is required, including secretor type and the lactation time of milk, as well as baby feeding.

## 1. Introduction

Breast milk is a vital highly nutritional food, which provides almost all the necessary nutrients and important bioactive substances needed for infant growth and development. Oligosaccharides (OS) are one of them. OSs are bioactive molecules which rank the third most abundant component after lactose and lipids in human milk [1,2], and play important roles in improving the growth and development of newborns [3]. OSs are generally defined as carbohydrate polymers that contain 3 to 10 monosaccharide units covalently linked through glycosidic bonds [4], which are divided into neutral oligosaccharides (whose structures are mainly lactose-linked with neutral monosaccharides such as glucose or galactose (Hex), N-acetylglucosamine or N-acetylgalactosamine (HexNAc) and fucose) and acidic oligosaccharides (containing acidic components such as N-acetylneuraminic (Neu5Ac) also known as sialic acid) [5,6,7]. OSs have been demonstrated to have prebiotic activity, antiadhesion effects, anti-inflammatory properties, glycome-modifying activity, benefits in brain development, growth-related characteristics of intestinal cells, and immune system modulatory functions; to provide protection from necrotizing enterocolitis; and to strengthen colonic barrier function [8,9,10,11,12,13,14].

Until now, more than 200 OSs have been identified from human milk, named human milk oligosaccharides (HMOs) [15,16]. HMOs are classified into 20 series based on the core structures [17]. The content of HMOs is approximately 20–23 g/L in colostrum and 5–15 g/L in mature human milk [18,19,20]. The amount and composition of HMOs are different based on the secretor status and Lewis blood group, namely secretor, nonsecretor, or Lewis-negative donors [20,21], and also based on different lactation stages [19]. HMOs have many isomeric forms, such as lacto-N-fucopentaose, I, II, III or sialyllacto-N-tetraose a, b, c [22], and are divided into three categories, including: neutral (fucosylated) HMOs, which contain fucose at the terminal position (e.g., 2’-fucosyllactose (2’-FL), lacto-N-fucopentaose I) or the GlcNAc residue of the Galβ1-3/4GlcNAc unit (e.g., lacto-N-fucopentaose II, III); neutral N-containing (nonfucosylated) HMOs which contain N-acetylglucosamine (e.g., lacto-N-tetraose or lacto-N-neotetraose); and acid (sialylated) HMOs which contain sialic acid at the terminal position (e.g., 3’- and 6’-sialyllactose, sialyllacto-N-tetraose a, c) of the GlcNAc residue of the Galβ1-3GlcNAc unit (e.g., sialyllacto-N-tetraose b) [21,23,24]. The characteristic structures of HMOs are closely related to their biological functions, showing a structure–function relationship.

A typical breast-fed infant consumes several grams of HMOs from breast milk every day. It is generally accepted that HMO cannot be digested by infants, yet can perform as prebiotics and is readily fermented by intestinal microbiota, provided that certain intestinal bacteria can express the requisite glycolytic enzymes to selectively stimulate the growth of beneficial bacteria such as Bifidobacteria and Lactobacilli, maintaining or improving the balance of intestinal flora [25,26]. The gastrointestinal tract is regarded as the location of the possible bioconversion of HMOs [27]. In recent years, some highly abundant HMOs have been demonstrated to exist in the feces of breast-fed babies [28], which suggests that infant gut bacteria are selective for the utilization or metabolism of oligosaccharides in breast milk. However, few studies have analyzed the composition of HMOs extracted from the feces of breast-fed babies and the relationship with breast milk HMOs so far [26,27,28,29,30,31,32,33].

Although several methods have been reported to successfully identify and analyze HMOs in human milk, the complex OSs as gastrointestinal bioconversion products of HMOs in feces are more difficult to detect and more complicated to analyze due to their enormous structural complexity. In the present study, a new method was developed for analyzing HMOs in breast milk and feces by using ultra-performance liquid chromatography–Q Exactive–HF hybrid quadrupole–Orbitrap–mass spectrometry (UPLC-QE-HF-MS) with high resolution and high sensitivity. Some newly OSs were speculated to be found using this technique compared with other existing methods in some previous reports, such as nanoLC-MS [30] and CE–LIF–MS^n^ [27], etc.

The knowledge of amount and composition of HMOs in the feces of breast-fed babies and the metabolic fate of HMOs is still limited. The aim of this study was to investigate the composition of the breast milk and feces of the breast-fed babies in the dyad of mothers and their breast-fed infants, simultaneously comparing their differences using UPLC-QE-HF-MS. The analysis of HMO content and composition in breast milk and the corresponding breast-fed infant’s feces can provide profound basic information for the fate which HMOs undergo throughout the gastrointestinal tract. Meanwhile, comprehensively comparing the distinctions of HMOs between breast milk and the infant feces will be helpful for better understanding the structure–function relationship of HMOs. The characterization of fecal HMOs is able to be applied as a valuable tool for monitoring the gastrointestinal fate of HMOs and reflecting the development of infants during different lactation times.

## 2. Materials and Methods

### 2.1. Reagents

Ethanol, methanol, methanoic acid, acetonitrile, and ammonium formate were purchased from Beijing Chemical Reagent Co. (China). All reagents were of analytical grade or chromatographic grade.

### 2.2. Subject Enrollment and Sample Collection

Four puerperal women who underwent routine postpartum examination at postnatal day 42 were recruited at the Maternal and Child Health Care Hospital of Changping District, Beijing. All the women underwent a physical examination to confirm not having diabetes, hypertension, abnormal thyroid function, abnormal liver function, abnormal kidney function, mastitis, or other breast-related diseases. The women had no serious genetic defects or mental diseases, without a history of long-term use of antibiotics, and agreed to participate in this study. In the morning of the sampling day, after the baby passed feces, one breast of the mother was carefully disinfected and then emptied with a sterile pump. The pumped breast milk was put in a sterile bottle, mixed thoroughly, and 10 mL of each sample was removed for subsequent analysis. Then, the infant was fed with the other breast of his/her mother. The next feces after feeding of each baby were collected using a special sterile fecal collection tube with a suited spoon. The fresh feces were scraped off from the diaper of the infant using the sterile spoon. The collected feces were immediately put into the sterile fecal collection tube and stored at −80 ℃ for further analysis.

This study was performed according to the guidelines laid down in the Declaration of Helsinki and all procedures involving human subjects were approved by the Committee on Medical Ethics of the Peking University, with the ethics approval number IRB00001052-17107. Written informed consent was obtained from all subjects.

### 2.3. Sample Preparation for OS Analysis

The methods of milk sample preparation were performed according to a previously published method [1] and some modifications were made. An amount of 2 mL of each milk sample was centrifuged at 10,000× *g* for 30 min at 10 °C to remove the upper fat. Two volumes of methanol were subsequently added and incubated at 4 °C for 2 h for removing the whey protein. The mixture solution was centrifuged at 4000× *g* for 30 min at 4 °C for complete phase separation. The supernatant containing the OS fraction was carefully transferred to a new tube for later analysis.

An amount of 0.1 g of the fecal sample of each baby was placed in a sterile tube and mixed with 1 mL of ethanol, respectively, and then crushed using an automatic tissue grinding machine (JXFSTPRP-48). The fecal slurries were incubated at 4 °C for 2 h and then centrifuged at 8000× *g* for 10 min at 4 °C for complete phase separation. The supernatant containing the OS fraction was carefully transferred into a new tube for LC-MS/MS analysis.

### 2.4. OS Detection Using UPLC-QE-HF-MS

In the current research, an ACQUITY UPLC column (2.1 mm × 100 mm, 1.7 μm) was used to separate OS on a Waters system. The basic parameters were listed as follows: column temperature: 35 ℃; flow rate: 0.3 mL/min; mobile phase A: acetonitrile; mobile phase B: 10 mM ammonium formate; gradient elution condition: 0–20 min 95–78% A; 20–35 min, 78–73% A; 35–38 min, 73–62% A; 38–45 min, 62–50% A; and injection volume: 10 μL.

OS was determined using UPLC-QE-HF-MS (Thermo) with an electrospray ionization (ESI) source. The mass spectrum conditions included a heated capillary of 320 ℃, a spray voltage of 3.8 KV in positive mode, and 3.4 KV in negative mode; the curtain gas was at 35 psi and collision-activated dissociation was at medium. Each ion was scanned based on the optimized declustering voltage and collision energy during detection. The analysis was operated with full scan (*m*/*z* 300–2000) in positive and negative mode for all OS. The matching and analysis of all possible oligosaccharides were performed according to the JCGG database (https://jcggdb.jp/idb/indexList.do?id=inchikey, accessed date: 20 October 2022). The matching degree between the actual molecular weight and the theoretical molecular weight was controlled within 5 ppm. The mass range for MS was set to *m*/*z* 400–2000. The difference in retention time (RT) of less than 0.5 min was used as a repeating substance.

### 2.5. Statistical Analysis

MetaboAnalyst (http://www.metaboanalyst.ca/ (accessed on 2 October 2022)) was used for multivariable analysis. We conducted supervised orthogonal projections to latent structures discriminant analysis (OPLS-DA). A 7-fold cross-validation test was performed to evaluate the goodness of fit of the OPLS-DA model with the values of R2Y and Q2. R2Y indicates how well the variation in a variable is explained, and Q2 indicates how well a variable can be predicted. A 200-time permutation test was then conducted to assess the robustness of the model. The differences between groups were compared with a t-test. The Spearman rank correlation was used to analyze the correlation between OSs in maternal milk and infant feces. All statistical tests were two-tailed, and the differences reached statistical significance at *p* < 0.05.

## 3. Results

**Figure 1:**
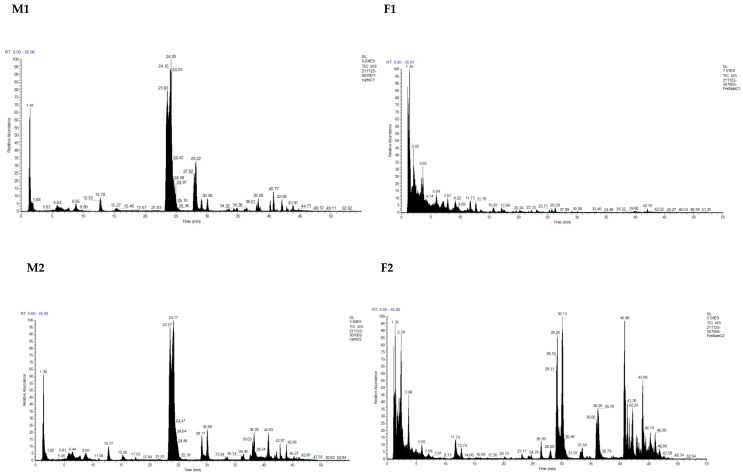

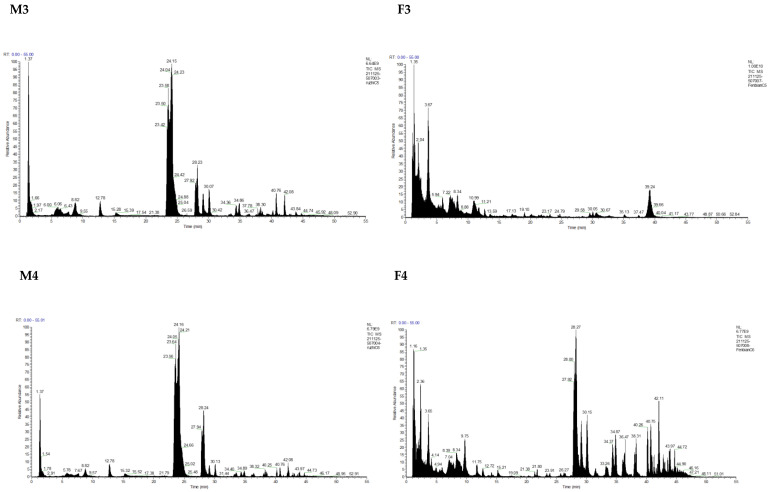
Total ion chromatogram of OSs from each pair of breast milk–infant feces sample.

The total ion chromatogram (TIC) of all the milk samples and fecal samples is shown in Figure 1, and the MS/MS spectra of LC fractions collected from each milk and fecal samples can be found in Supplementary Figure S1. JCGG databases were used to analyze the identified HMOs and their peak area in each breast milk and fecal sample, as seen in Supplementary Table S1. M1 and F1 represented breast milk and feces from the first woman and her baby, respectively, as did M2 and F2, M3 and F3, and M4 and F4. The composition and their peak area of HMOs only identified in the feces of the babies are shown in Supplementary Table S2. The abundance (peak area), relative content, and quantity of total, acidic, and neutral OSs in the breast milk and the feces of each mother and her baby are summarized in Table 1. The dHex in Supplementary Tables S1 and S2 is assumed to reflect Fuc (fucose) in this investigation based on the composition of the published data [34].

### 3.1. Identification and Analysis of OS in Breast Milk and Feces

In the current research, a total of 305 OS structures including isomers were identified in the breast milk and feces samples using UPLC-QE-HF-MS, ultimately indicating that the proposed approach was reliable and efficient for detecting OS. The OS count detected in each of the breast milk samples was similar, which was 64, 67, 61, and 69, respectively (Supplementary Table S1). In all the breast milk samples, neutral HMOs were more numerous and abundant than acidic HMOs, as depicted in Table 1. OS was also detected in all the feces samples and the results are shown in Supplementary Tables S1 and S2. A total of 4, 80, 18, and 58 newly formed OSs, which was not detected in the corresponding breast milk sample, were identified exclusively in each feces sample, respectively.

The OSs in the breast milk samples (M1-M4) with the highest abundance were Hex2Fuc1 and its isomers at *m*/*z* 488.174. The second most abundant HMOs in M1 and M2 were Hex3HexNAc1 (*m*/*z* 707.2483) and its isomers, which were followed by Hex4 (*m*/*z* 666.2217) and Hex2Neu5Ac1 (*m*/*z* 633.2115), respectively. Hex2dHex2 (*m*/*z* 634.2319) and its isomers ranked the second most abundant in M3 and M4. Hex3HexNAc1dHex2 (*m*/*z* 999.3641) and Hex2Neu5Ac1 (*m*/*z* 633.2115) and its isomers were the third most abundant HMOs in M3 and M4, respectively.

Hex2Fuc1 (*m*/*z* 488.174) and its isomers had the highest abundance in F1, F2, and F4 feces samples, which was consistent with the trend in abundance in breast milk. The OS with the highest abundance in the F3 sample was Hex2HexNAc1 at *m*/*z* 545.1955, as well as its isomers. Hex3 (*m*/*z* 504.169) and its isomers were the second most abundant OS in the F1 and F3 samples, followed by Hex1HexNAc1Fuc1 (*m*/*z* 529.2006) and Hex2Fuc1 (*m*/*z* 488.174), respectively. In the F2 sample, the abundance of Hex2Neu5Ac1 (*m*/*z* 633.2115) and Hex3HexNAc1Neu5Ac1 (*m*/*z* 998.3437) ranked the second and third highest, respectively, while, simultaneously, Hex3HexNAc1dHex2 (*m*/*z* 999.3641) and Hex2Neu5ac1 were the third highest in the F4 sample.

**Figure 2:**
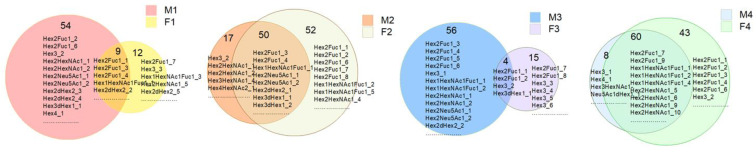
The Venn diagram of common OSs in the four pairs of breast milk–infant feces samples.

**Figure 3:**
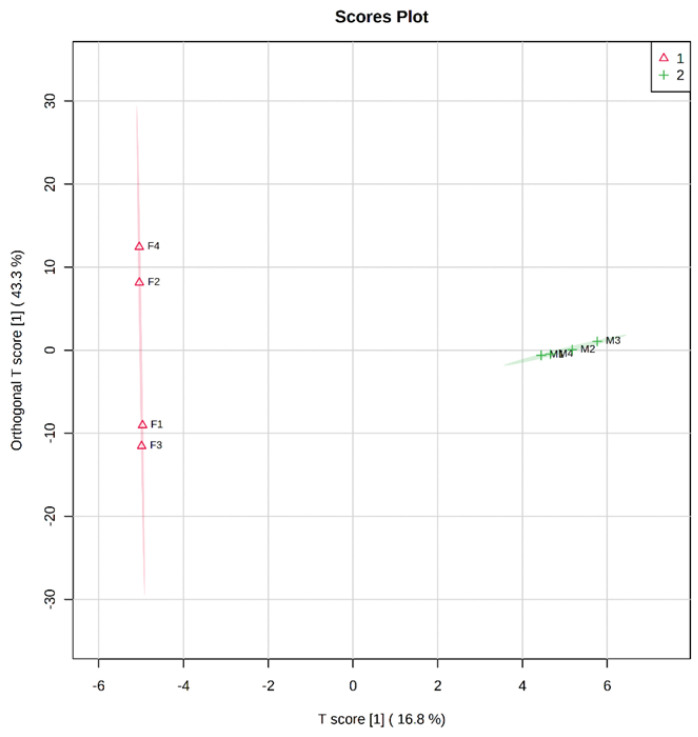
The scores plot of each breast milk and infant feces sample.

**Figure 4:**
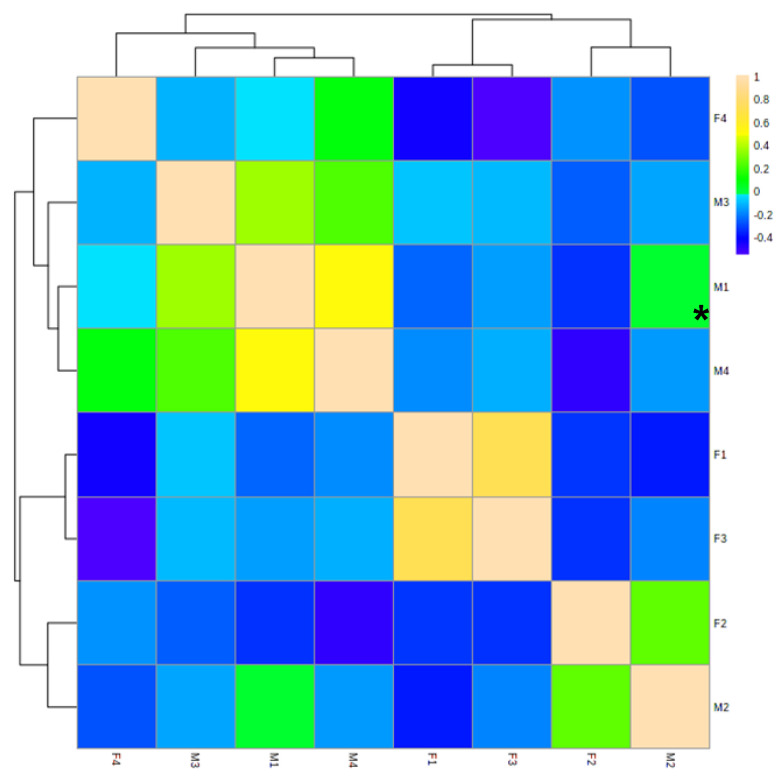
The correlation heat map of breast milk and infant feces samples. The yellow color represents positive association; the blue color represents negative association. The darker the color, the stronger the correlation between the two kinds of samples. The asterisk indicates statistical significance (*p* < 0.05).

As shown in Figure 2, breast milk and feces samples from the four mother–baby pairs shared 9, 50, 4, and 60 common OSs, respectively. The distribution of OSs was different between breast milk and feces samples (Figure 3); the cross-test results were R2Y = 0.996 and Q2Y = 0.778, and the replacement test result was *p* = 0.05, which indicated the model fit well. As is shown in Figure 2 and Figure 3, the composition of OS in breast milk was significantly different from that in the corresponding infant feces. However, the correlation heat map of feces and milk samples illustrates that there was a certain association between OSs in breast milk and the babies’ feces samples (Figure 4). Among them, M1 was negatively correlated with F1 (*p* < 0.05). These findings might indicate that the HMO composition in breast milk changed greatly throughout the gastrointestinal tract.

### 3.2. Analysis of Functional OS in Breast Milk and Feces

N-acetylhexosamine (HexNAc) was regarded as a component of so-called the bifidus factor [35]. The neutral HMOs which contained N-acetylglucosamine and fucose monomers were regarded as substances for the development of the intestinal microbiota typical for breast-fed infants [36]. Furthermore, the fucosylated HMOs were related to the lower risk of diarrhea and respiratory diseases in breast-fed infants [37].

**Figure 5:**
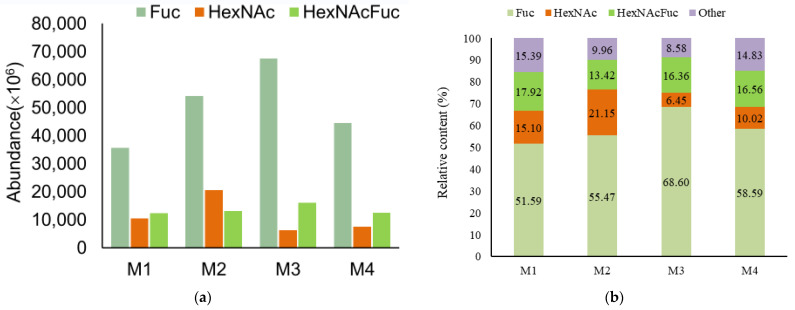
The abundance (peak area) (**a**) and relative content (**b**) of Fuc, HexNAc, and HexNAcFuc OS in various breast milk samples.

**Figure 6:**
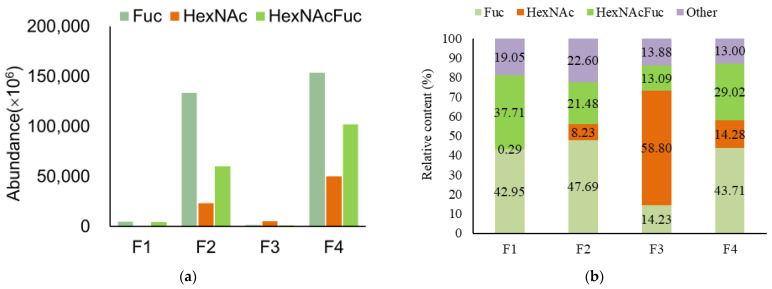
The abundance (peak area) (**a**) and relative content (**b**) of Fuc, HexNAc, and HexNAcFuc OS in various infant feces samples.

As shown in Figure 5, the fucosylated HMOs indicate OSs containing a fucose monomer, and HexNAcFuc-OS indicates OSs containing both N-acetylhexosamine and fucose monomers. There was a high concentration of fucosylated HMOs (69.511, 68.885, 84.967, and 75.152%) with varying degrees of complexity in the breast milk samples. HexNAcFuc was found in 17.92%, 13.42%, 16.36%, and 16.56% of the samples examined in the current study, respectively. It is worth noting that the abundance and content of fucosylated OS in each breast milk and feces sample were higher than those of HexNAc, with the exception of the F3 sample, which contributed to the highest abundance and content of HexNAc (Figure 6). HexNAc was more abundant and had a higher content than HexNAcFuc in M2, while HexNAcFuc was more prevalent and had a higher concentration than HexNAc in F2 (Figure 5B). Nonetheless, there were diametrically opposed changes in F3, with the abundance and content of HexNAcFuc being lower than those of HexNAc (Figure 6).

## 4. Discussion

As demonstrated in Supplementary Tables S1 and S2, it was obviously conspicuous that the kind and quantity of OS varied across the samples of breast milk and feces. The majority of OSs in breast milk were detected in the corresponding feces samples. The quantity and abundance of neutral OSs were greater than those of acidic OSs in each sample, which was consistent with the findings of a previous study [20]. In the breast-fed infant feces, a rather high concentration of oligosaccharides, which contained blood group A antigen (GalNAcα1-3(Fucα1-2)Gal) or B antigen (Galα1-3(Fucα1-2)Gal), were detected, while these existed at a trace level or did not exist in the mother’s milk. [31,32]. In accordance with earlier studies concluding that Hex2Fuc1 was one of the most common HMOs in human milk, Hex2Fuc1 (*m*/*z* 488.1741) was found to rank highest and second highest in abundance in the milk and feces samples, respectively [38].

For the four pairs of breast milk–feces samples, the number of some OSs decreased from mothers’ breast milk to their own baby’s feces while others increased (Table 1). There were no significant rules in the changes in number of OS types from breast milk to feces. The number of total OS types in F2 and F4 was apparently raised from the breast milk to feces by the baby’s digestive system (Table 1). Nevertheless, there were polar opposite changes in F1 and F3, with the number of total OSs in the feces being lower than that in the breast milk (Table 1). Differences were observed in the number of acidic and neutral OSs in the breast milk of all four mothers and their babies’ feces (M1 > F1, M2 < F2, M3 > F3, and M4 < F4). The difference in the number of OSs between the breast milk and fecal extracts could be attributed to the activity of gastrointestinal enzymes [32], which include normal lactase, fucosidase [39], sialidase, etc., as well as microbiota metabolization. The bacteria in the intestine can produce various glycosidases that decompose HMOs and other glycoconjugates. When HMOs pass through the digestive tract, some would be degraded if the right bacteria happen to be present, and even form new OSs [30]. Some glycoconjugates, such as glycoproteins and glycolipids, are able to be enzymatically degraded by microbiota such as *bifidobacteria* to release new free OSs [30]. It is of good value to elucidate the corresponding relationship between the type of enzyme and the specific structure of HMOs cleaved.

The abundance and content of fucosylated OSs in the F3 sample were lower than that of HexNAc (Figure 6); this finding might demonstrate that the effects of microbiota on fucosylated HMO degradation were more significant than those on the redesign or regeneration of other fucosylated HMOs in the third baby. The utilization of fucosyllactose has been demonstrated as a key genetic factor affecting infant gut microbiota development [40]. HexNAc was more abundant and had a higher content than HexNAcFuc in M2; this result denotes that HexNAc might be largely consumed in the digestive system or that more HexNAcFuc was formed in the M2 sample.

The variation in content of HexNAc, Fuc, and HexNAcFuc-OS from the breast milk to the infant feces was concerning, with different degrees of degradation and formation by the intestinal microbiota. The degree of degradation and formation depended on the species of gastrointestinal flora [30]. Theoretically, since the gastrointestinal flora of infants under 1 year old is still in the process of maturation, the composition of the flora will continue to change with the increase in age, and the decomposition and utilization of HMOs of infants will also change correspondingly, which needs to be confirmed by prospective cohort studies. The infants in this study were 42 days postpartum, and their gastrointestinal flora was still quite immature, so the decomposition and utilization of HMOs might still be at a relatively low level.

The OSs shown in Supplementary Table S2 were newly redesigned in feces via microbiota metabolization in the intestinal tract. A large amount of the newly discovered OSs in Supplementary Table S2 could be the result of the gastrointestinal degradation of higher-molecular-weight HMOs. According to the results shown in Figure 3 and Figure 4, there were significant variations in HMO composition when they went through the gastrointestinal tract. A similar factor likely contributed to the difference in OSs between breast milk and infant feces, with microbes in the gastrointestinal flora degrading and metabolizing OSs. Remarkably, only a few of the newly created OSs mentioned were abundant, with Hex4HexNAc3dHex2 (1048.744×10^6^) having the largest peak area in the F2 sample (Supplementary Table S2). The results were speculated to be caused by the digestive system’s breakdown of HMOs by the gastrointestinal enzymes of the intestinal microbiota, the digestion products, and free OSs passing through the gut and staying in feces. There was not an exclusively specified microbiota for the degradation and metabolization of HMOs [32]. The gastrointestinal bioconversion of the HMOs might include redesign with mucin oligosaccharides which are present in the glycoconjugates of the human gastrointestinal mucosa [32]. On the one hand, factors other than the composition of the individual breast milk may also have an impact on the fecal OS profile. For instance, a decline in gastrointestinal sialidase activity might be able to inhibit a further enzymatic degradation of the acidic OS [41]. However, the genotype of the host was also likely to contribute to the gastrointestinal OS metabolization [32]. The characteristics of fecal OS remained baby-specific and no overarching trends were found in the present study.

In the present study, no unifying trends in OS were found in the breast milk–infant feces pairs, with the characteristics of infant fecal OS showing individual specificity. Although the study has limitations such as the relatively small sample size and the unclear secretor status of the mothers, with which our further study will be concerned. The results make us understand that the oligosaccharides in breast milk are not completely unconsumed after entering the infant digestive tract, but are likely to undergo complex digestion and various stages of intestinal flora metabolism. The genetic background of the mother and infant and the differences in the colonization and composition of infant intestinal flora will have important effects on the utilization of oligosaccharides from breast milk. Understanding these specific effects and figuring out how they interact with each other will help us to develop more scientific infant-feeding strategies, and also be helpful for the research and development of functional infant formula.

## 5. Conclusions

In conclusion, this present study provides us with a detailed transformation on HMO gastrointestinal fate by comparing HMOs in breast milk and the fecal profiles of breast-milk-fed infants. The irregular changes in the number of OSs from breast milk to feces conspicuously demonstrated that gastrointestinal microbiota metabolization, such as degradation and reformation, played critical roles in the fate of HMOs in breast milk in early life. There should be more research on the gastrointestinal bioconversion of HMOs so as to use the fecal OS profiles as mirrors for monitoring the gastrointestinal ripening during the development of infants. It will be necessary to concern the secretor type and lactation time of milk, as well as the feeding pattern, in further studies. Meanwhile, comprehensively comparing the distinctions of OSs among breast milk and the feces of breast-fed babies would be significantly advantageous for better grasping the structure–functional relationship of feeding-related OSs.

## Figures and Tables

**Table 1:** The abundance (peak area), relative content, and abundance of total, acidic, and neutral OS in the four pairs of breast milk–infant feces samples.

Milk	Total OSs		Neutral OSs			Acidic OSs		
	Abundance (×10^6^)	Number	Abundance (×10^6^)	Content (%)	Number	Abundance (×10^6^)	Content (%)	Number
M1	69,218.8355	64	62,978.1215	90.98	46	6240.714	9.02	18
F1	11,510.129	25	11,488.215	99.81	24	21.914	0.19	1
M2	97,732.49	67	85,318.297	87.30	45	12,414.193	12.70	22
F2	279,884.703	183	190,641.31	68.11	121	89,243.393	31.89	62
M3	98,446.8111	61	93,316.3521	94.79	45	5130.459	5.21	16
F3	9040.734	36	8975.228	99.28	32	65.506	0.72	4
M4	76,029.096	69	67,880.439	89.28	50	8148.657	10.72	19
F4	351,025.401	163	289,776.038	82.55	108	61,249.363	17.45	55

## Data Availability

The datasets used during the current study are available from the corresponding author on reasonable request.

## Supplementary Materials

The following supporting information can be downloaded at: https://www.mdpi.com/article/10.3390/nu15040888/s1, Figure S1: The MS/MS spectra of LC fractions collected from various milk and fecal samples. Table S1: OS identified and their peak area in breast milk of four women and feces of the women’s babies. Table S2: The composition and their peak area of OS only identified in feces of four women’s babies.
