# Supplementary material for: Comparative Analysis of Oligosaccharides in Breast Milk and Feces of Breast-Fed Infants by Using LC-QE-HF-MS: A Communication
Source: Nutrients. 2023 Feb 9;15(4):888. doi: 10.3390/nu15040888 (PMC9963387; doi:10.3390/nu15040888)
Supplement: Supplementary file 1 [file nutrients-15-00888-s001.zip › Supplement FigureS1.pdf]

Compounds

Structure	Name	RT [min]	Formula	Calc. MW	Areas
n/a		11.33	n/a	488.17164	4.08e8 2.19e8 3.61e7 2.02e8

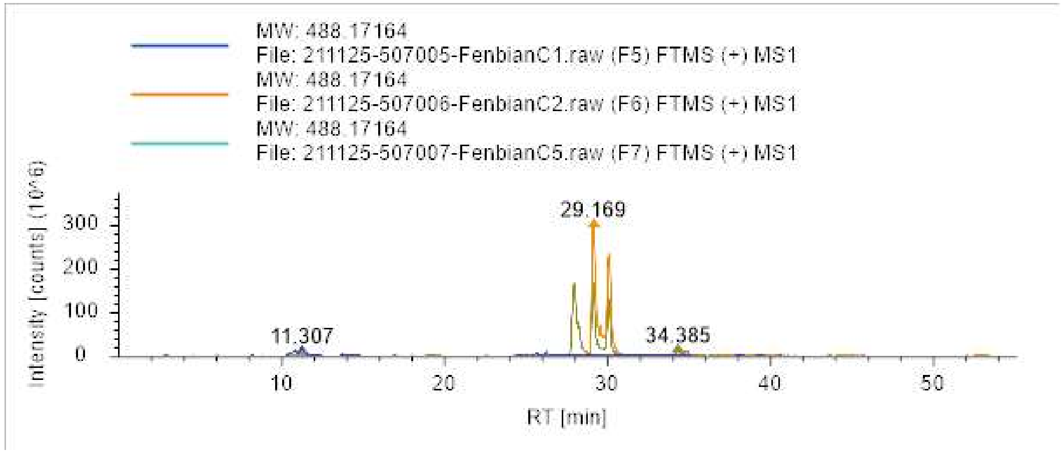

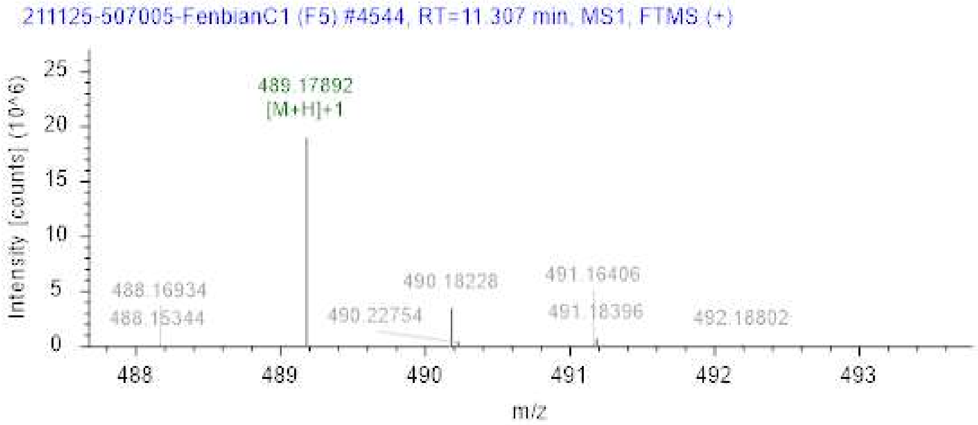

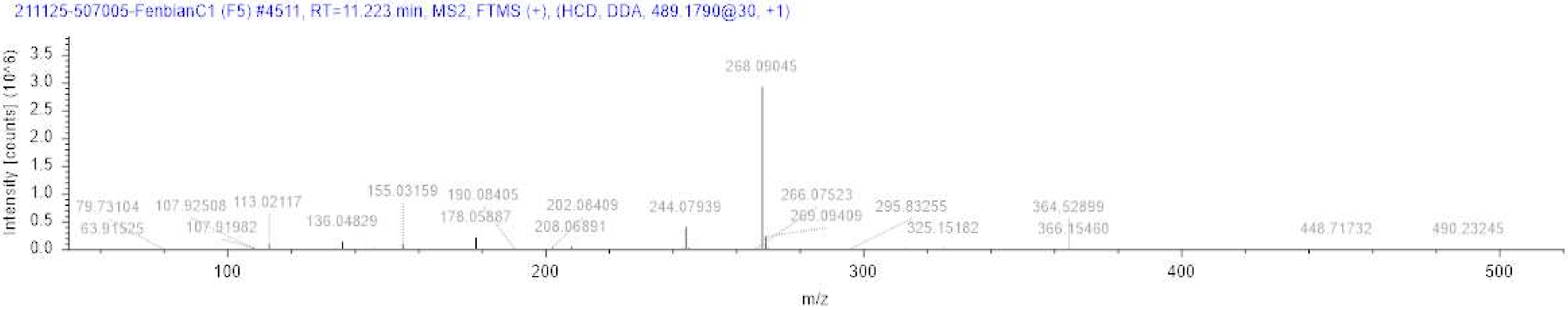

© Reported with Compound Discoverer 3.3

Compounds

Structure	Name	RT [min]	Formula	Calc. MW	Areas
n/a		34.38	n/a	488.17252	3.37e7 / 1.32e8 4.38e7 / 1.96e7 / 4.08e8

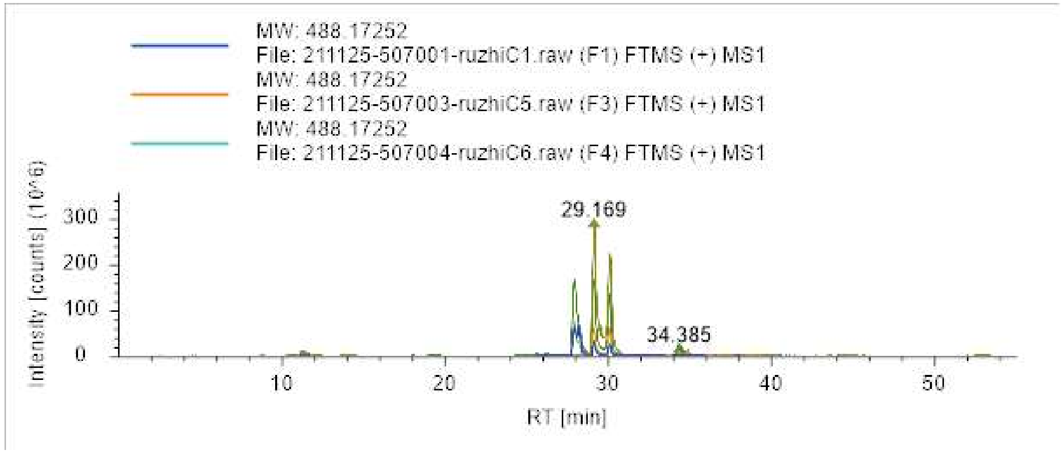

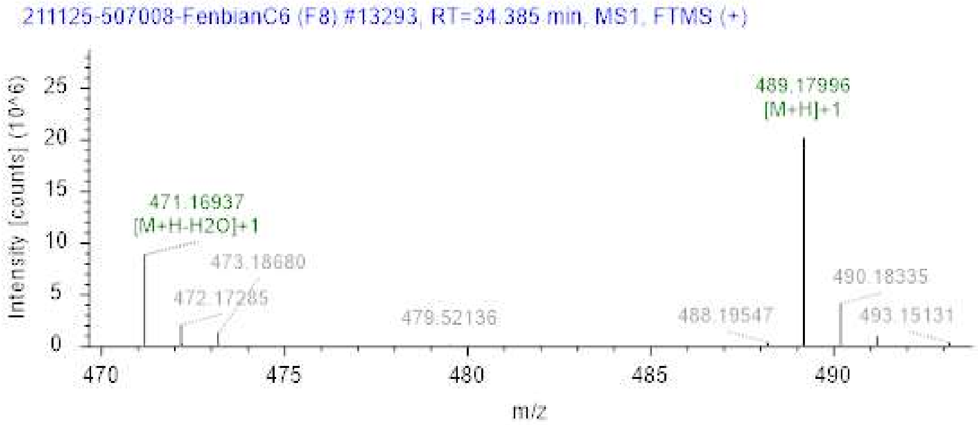

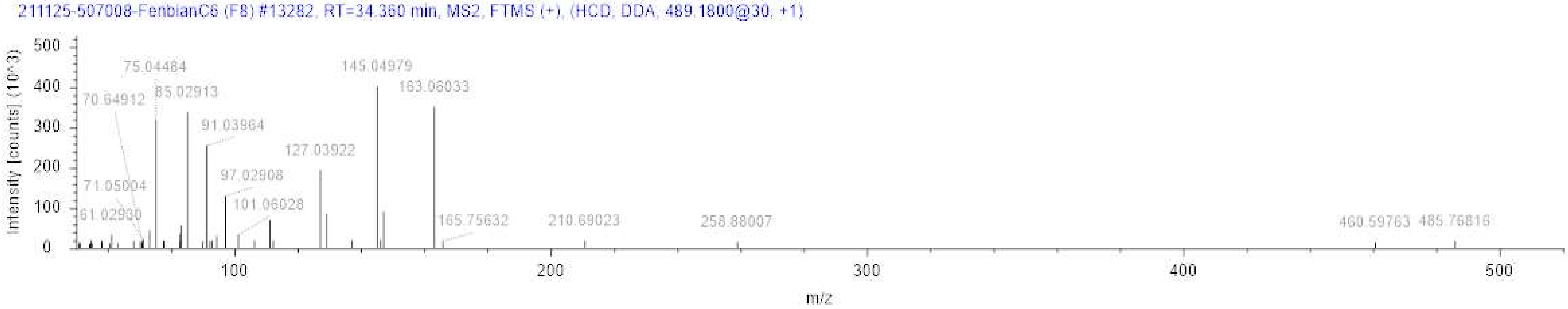

© Reported with Compound Discoverer 3.3

Compounds

Structure	Name	RT [min]	Formula	Calc. MW	Areas
n/a		29.18	n/a	488.17286	6.45e9 2.68e1[illegible] 2.20e1[illegible] 5.77e9 7.52e8 4.96e10 2.84e10

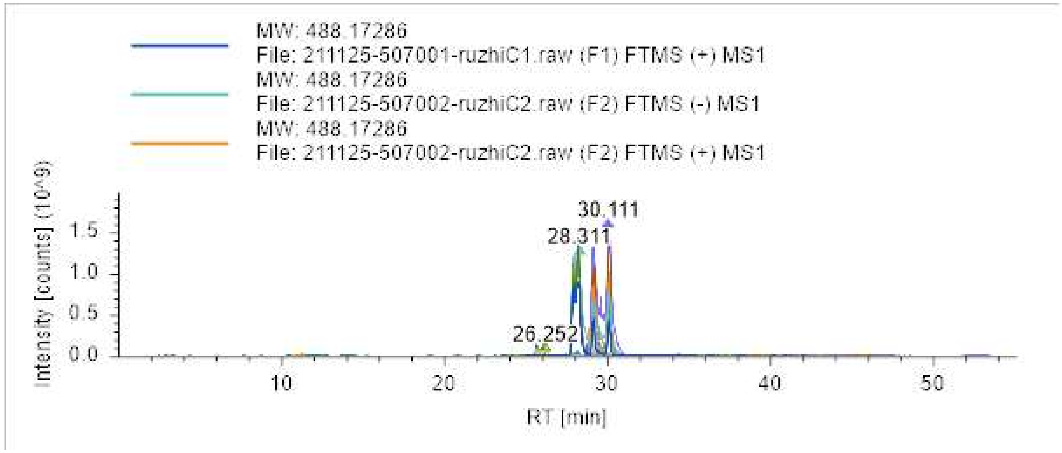

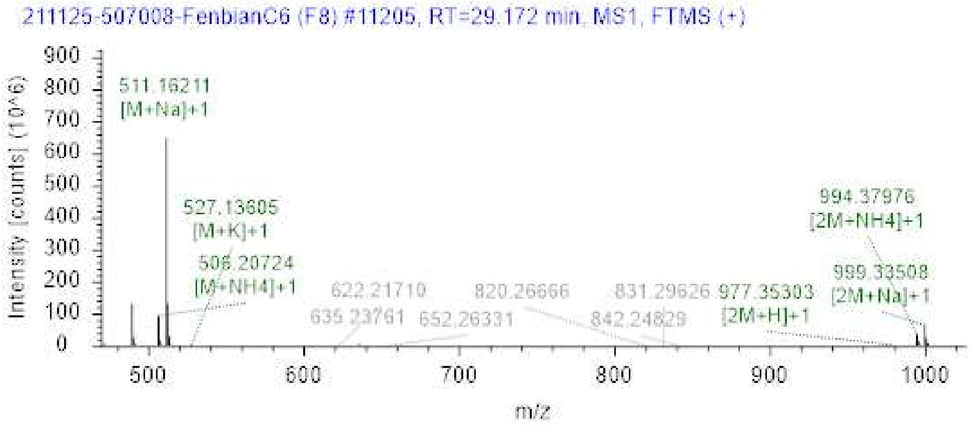

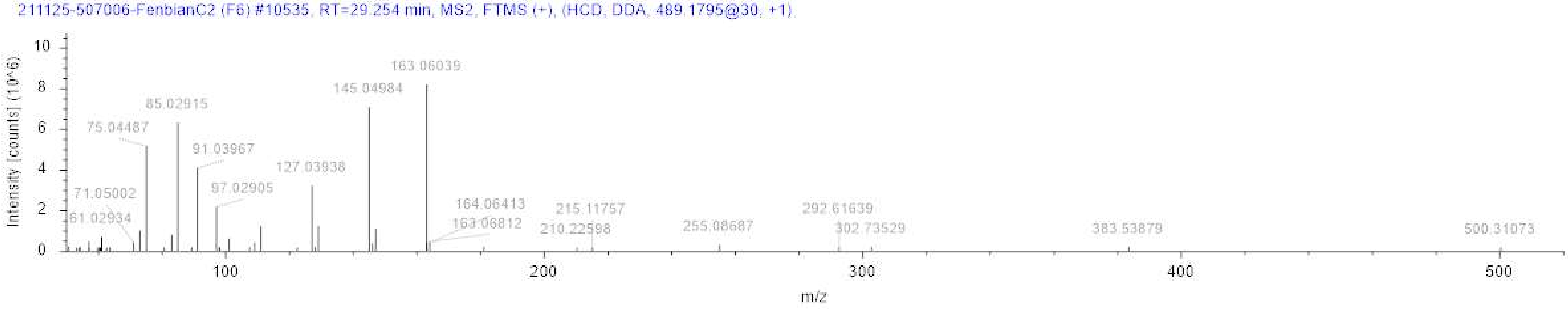

© Reported with Compound Discoverer 3.3

Structure	Name	RT [min]	Formula	Calc. MW	Areas
n/a		25.72	n/a	488.17301	2.19e8 · · 1.10e8 · 7.65e7 · 2.89e9 · 1.55e7 · 6.58e8 · 2.57e9 ·

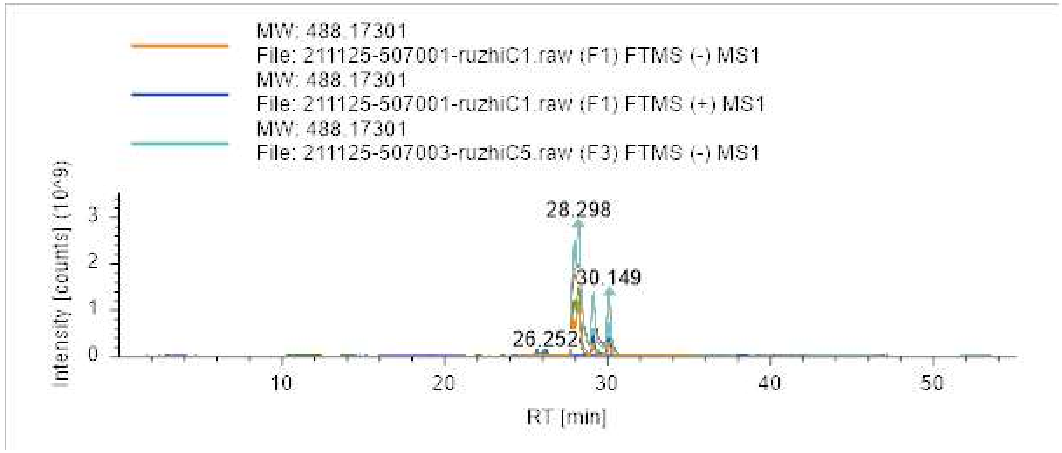

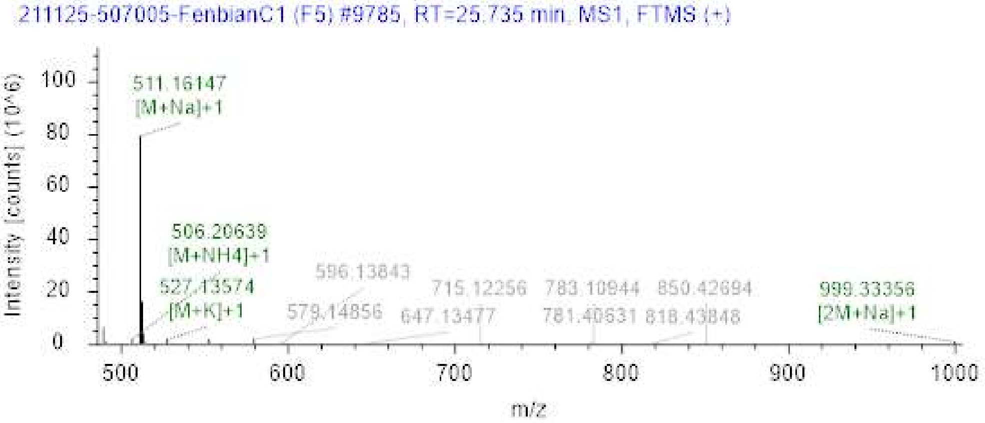

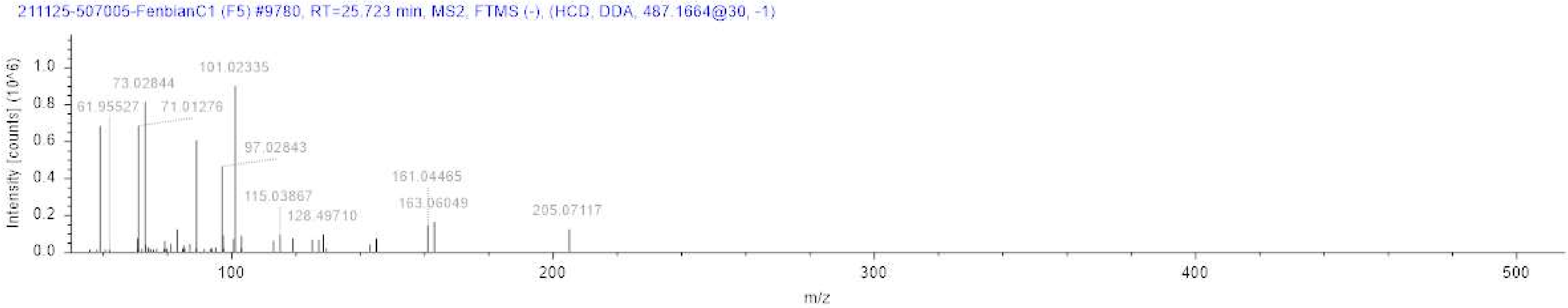

© Reported with Compound Discoverer 3.3

Compounds

Structure	Name	RT [min]	Formula	Calc. MW	Areas
n/a		28.00	n/a	488.17323	1.25e10 / 7.40e9 / 1.77e10 / 3.08e7 / 3.62e8 / 7.46e10

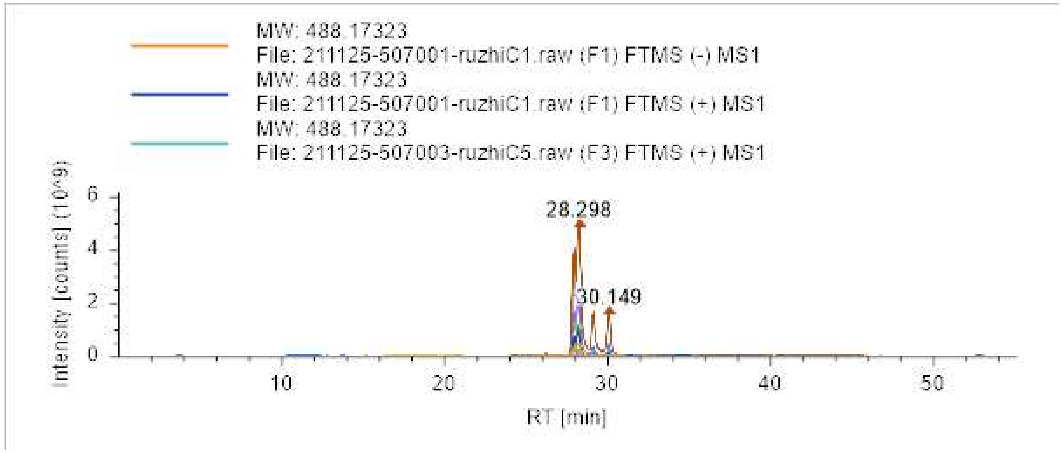

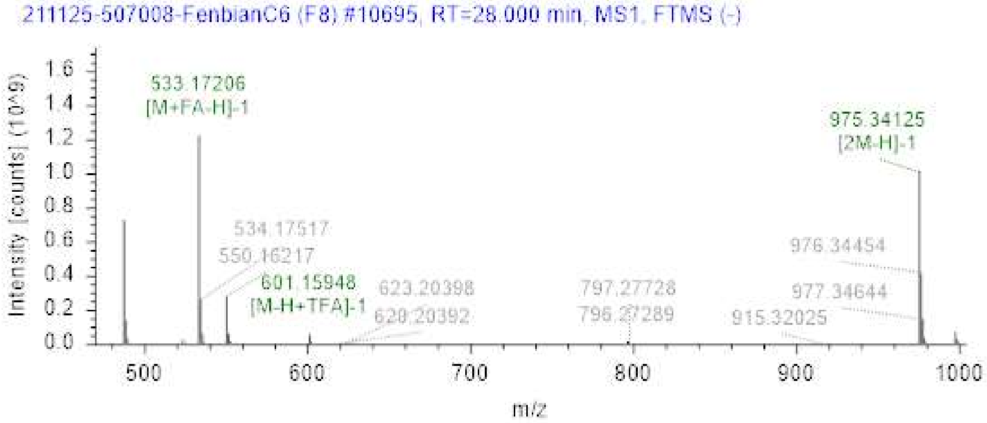

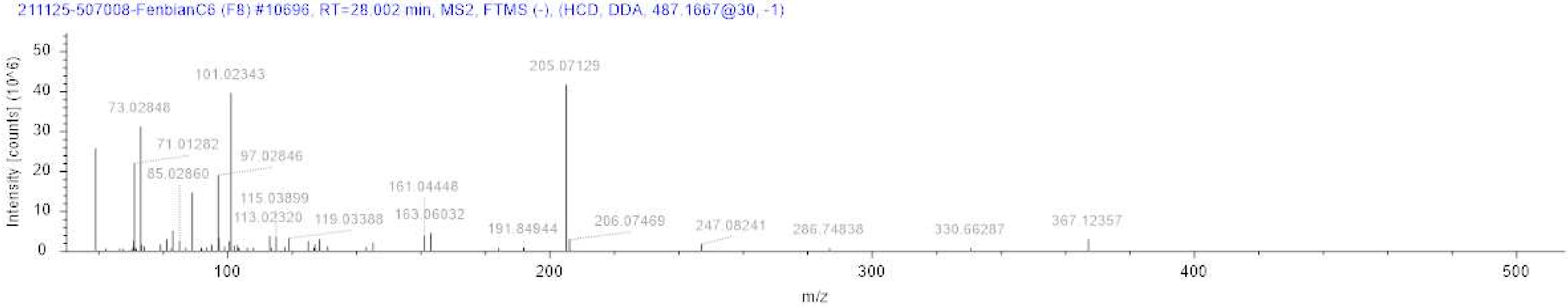

© Reported with Compound Discoverer 3.3

Compounds

Structure	Name	RT [min]	Formula	Calc. MW	Areas
n/a		27.45	n/a	488.17346	1.95e7 2.17e8

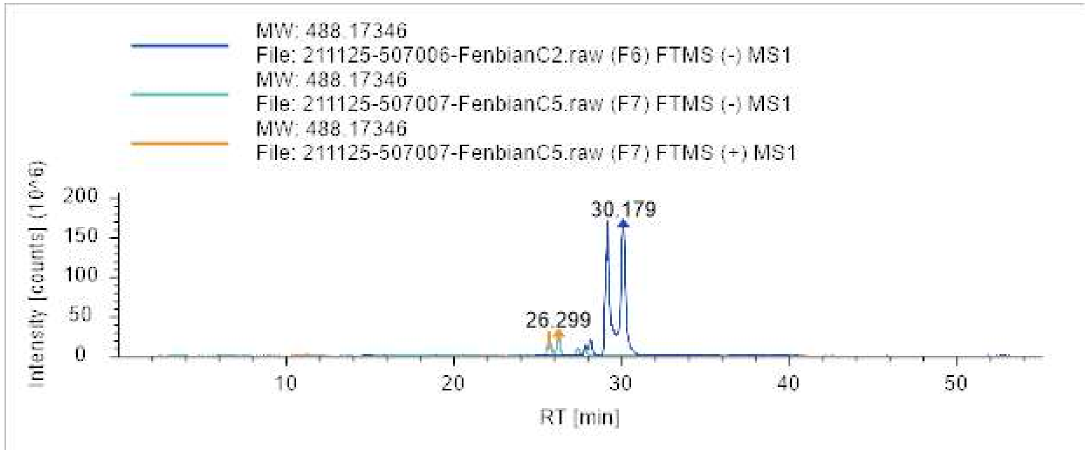

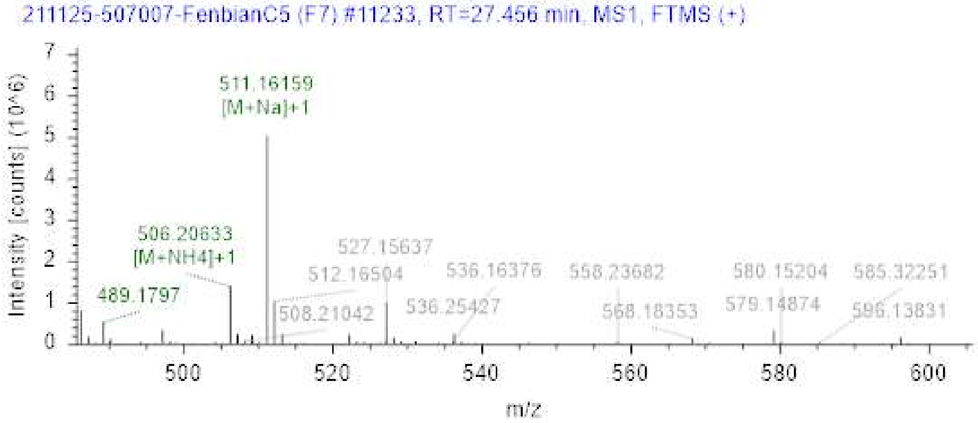

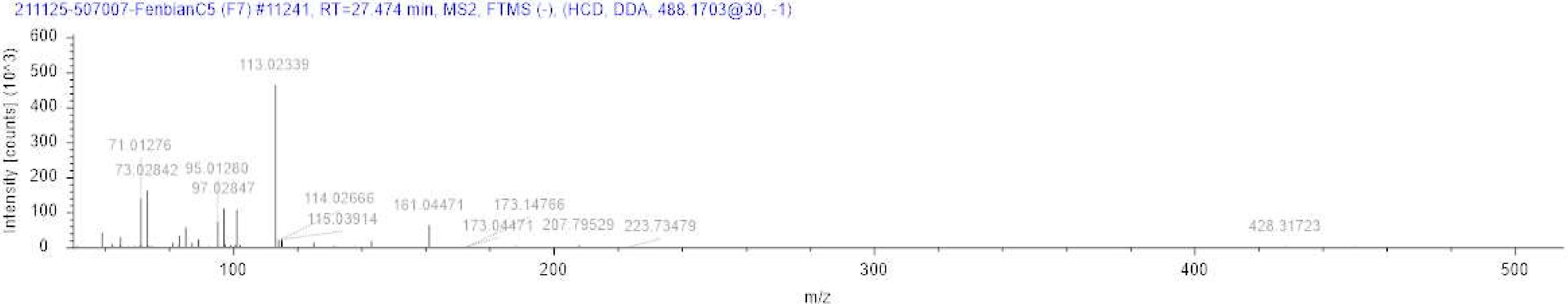

© Reported with Compound Discoverer 3.3

Compounds

Structure	Name	RT [min]	Formula	Calc. MW	Areas
n/a		32.33	n/a	488.17357	1.24e7

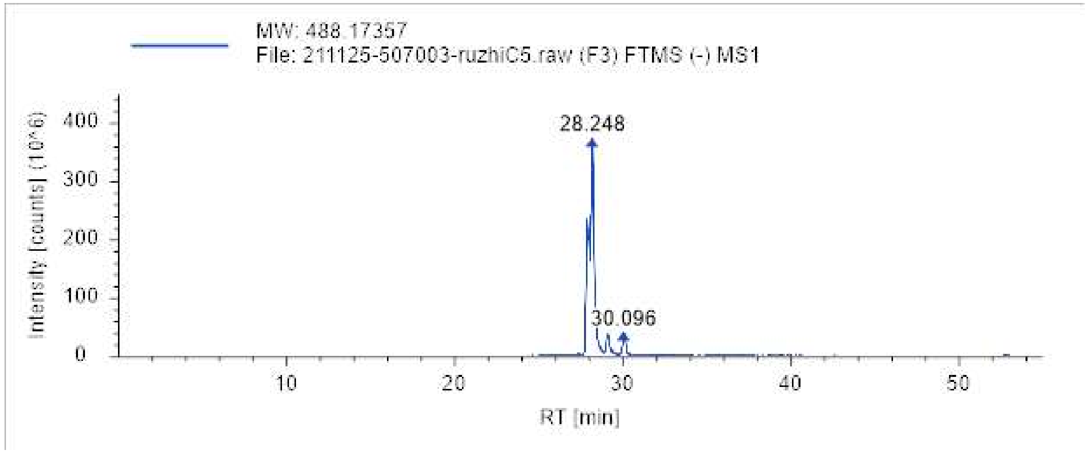

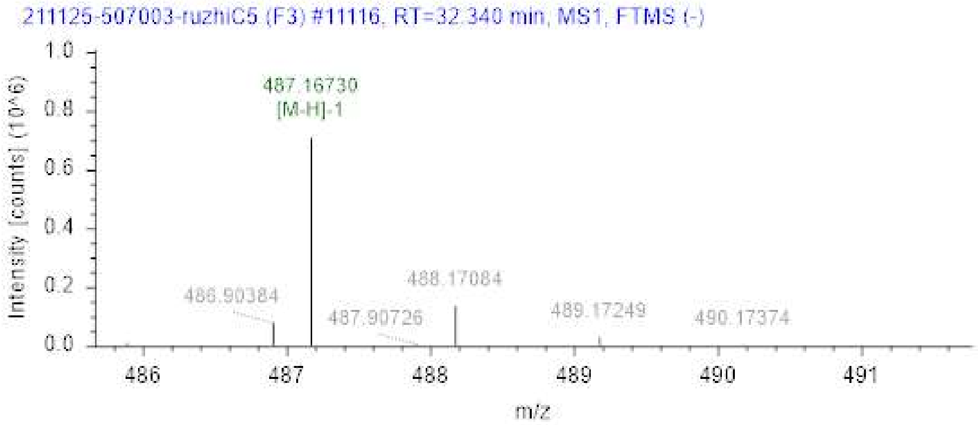

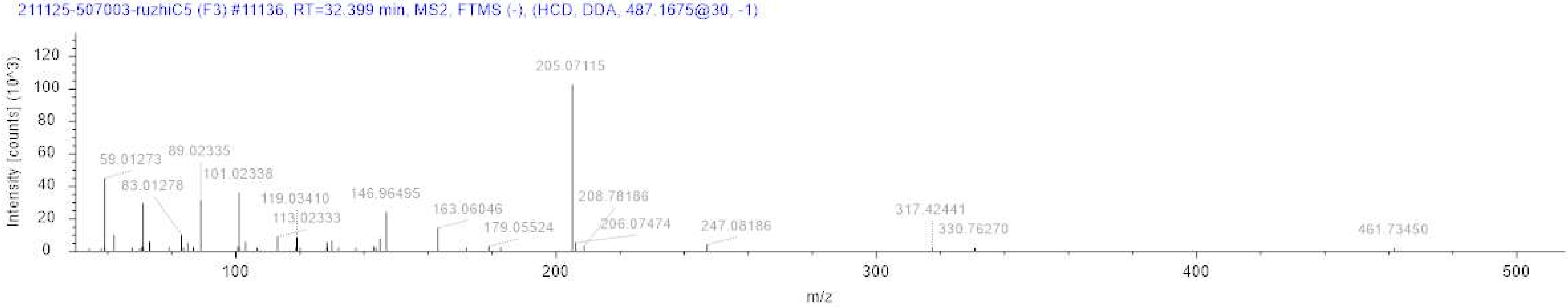

© Reported with Compound Discoverer 3.3

Structure	Name	RT [min]	Formula	Calc. MW	Areas
n/a		30.15	n/a	488.17372	1.14e10 2.72e10 2.38e10 1.09e10 6.91e8 7.88e10 6.29e9

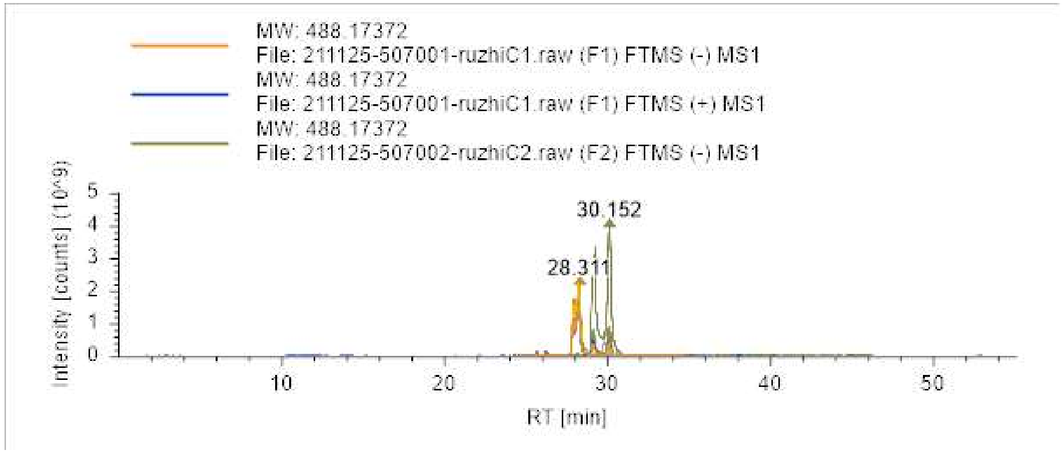

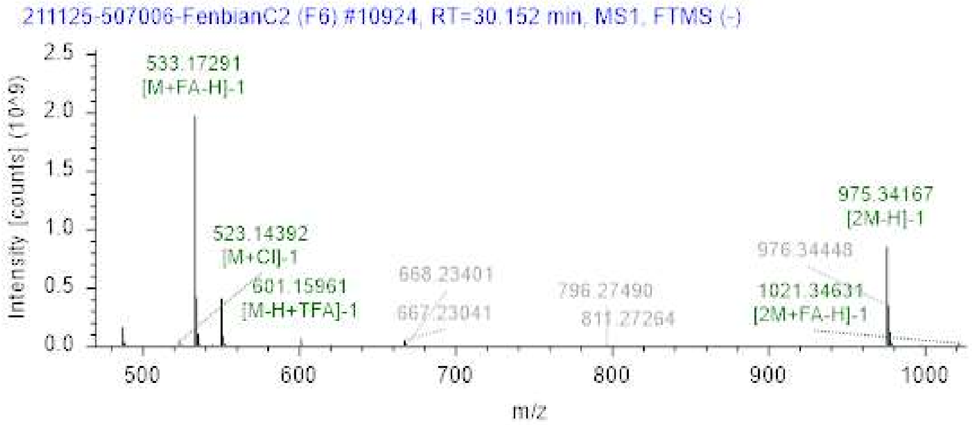

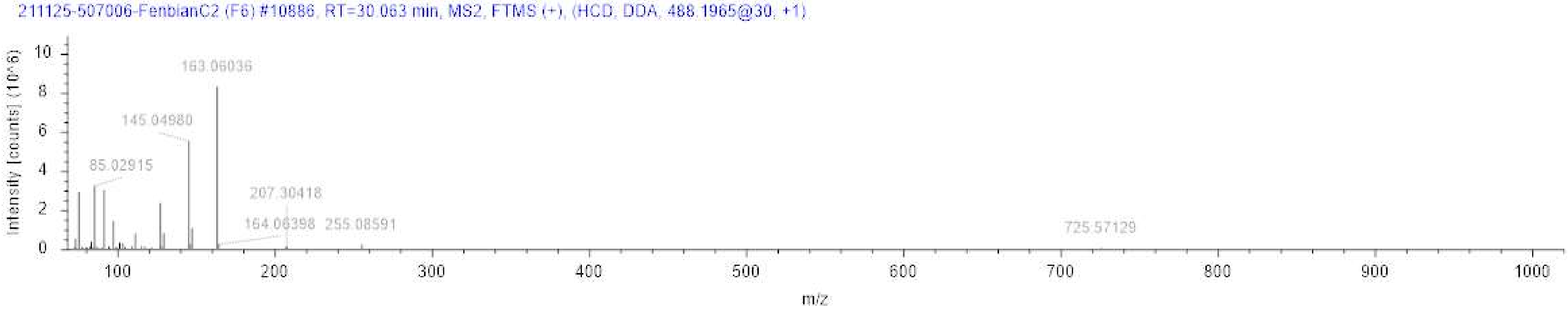

© Reported with Compound Discoverer 3.3

Compounds

Structure	Name	RT [min]	Formula	Calc. MW	Areas
n/a		40.67	n/a	488.17386	2.26e7

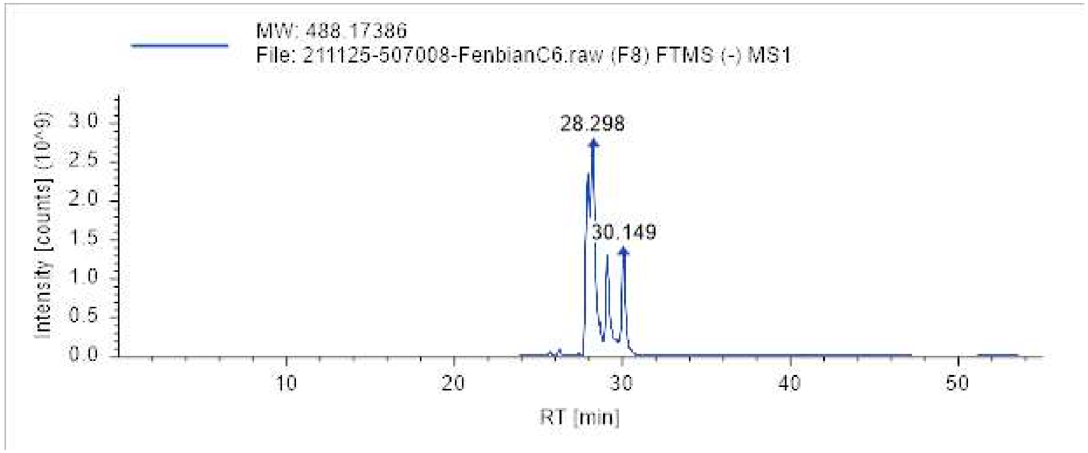

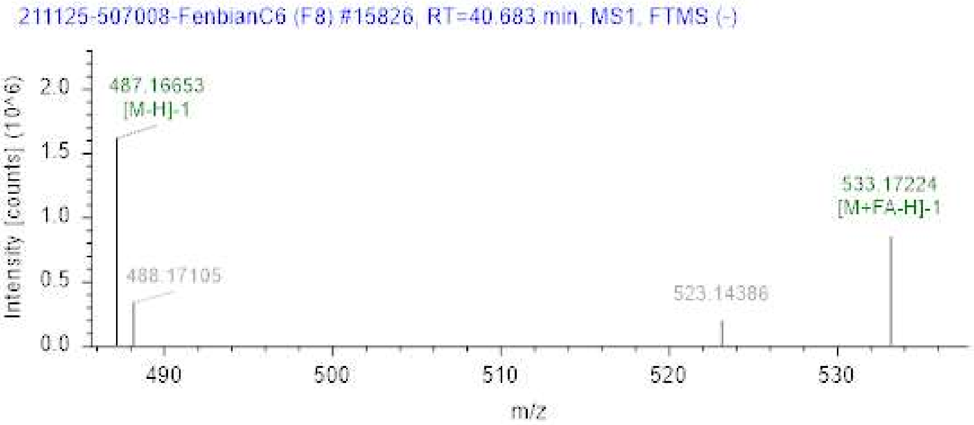

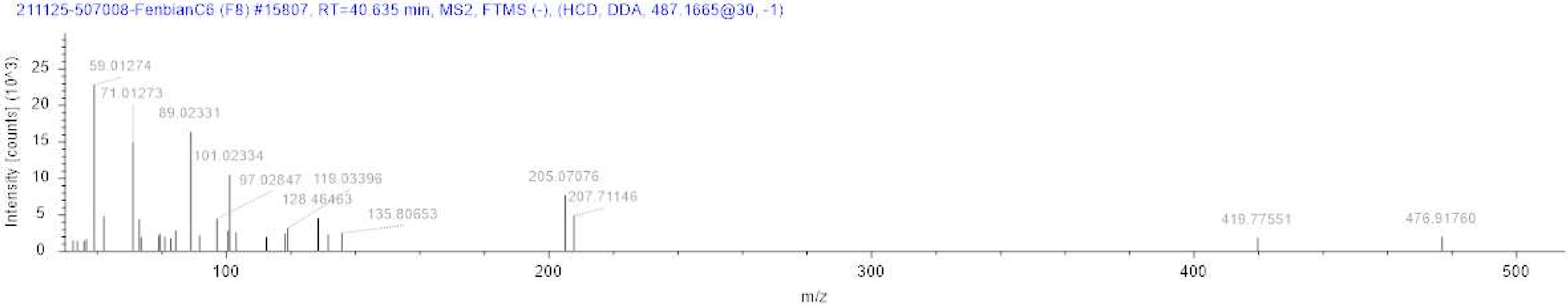

© Reported with Compound Discoverer 3.3

Compounds

Structure	Name	RT [min]	Formula	Calc. MW	Areas
n/a		24.12	n/a	504.16696	9.43e8 9.12e8

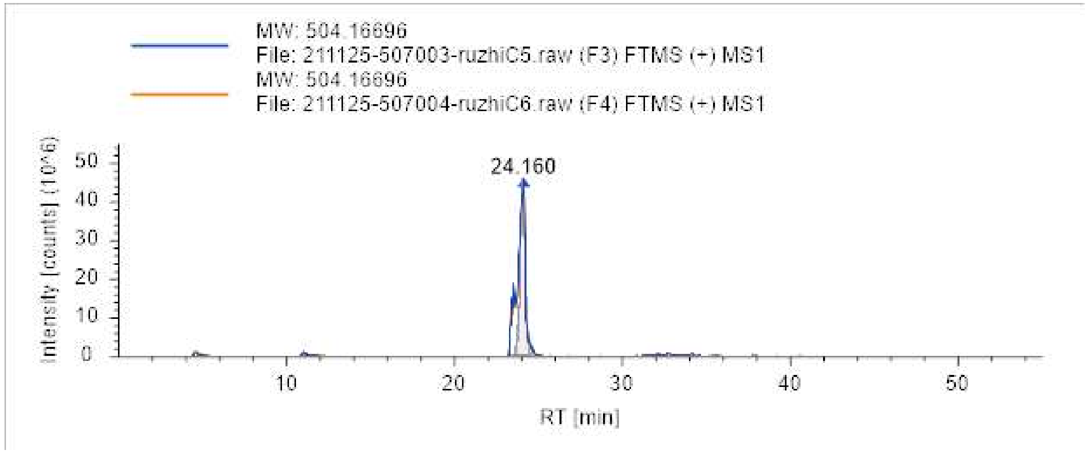

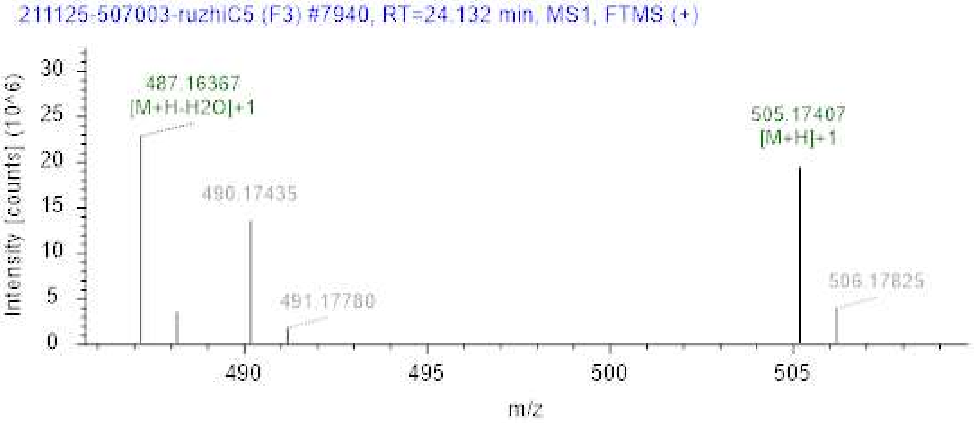

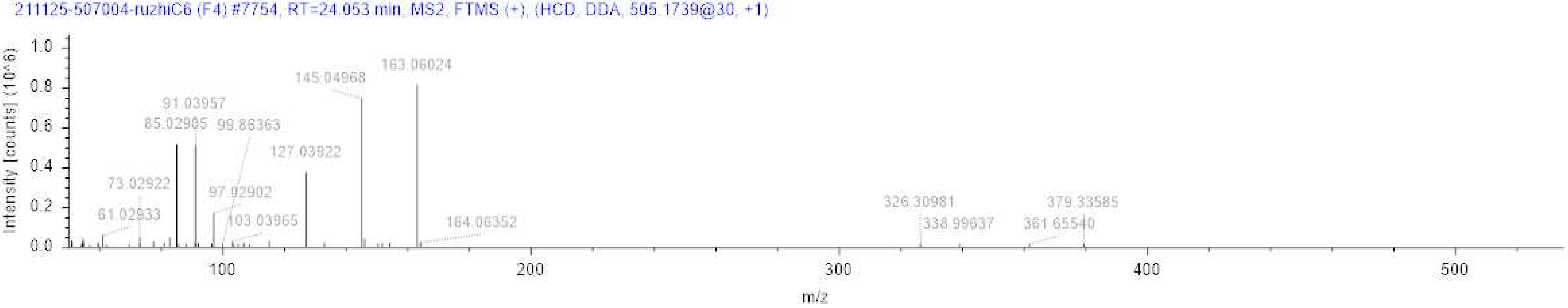

© Reported with Compound Discoverer 3.3

Structure	Name	RT [min]	Formula	Calc. MW	Areas
n/a		32.74	n/a	504.16871	1.62e8 3.87e8 1.11e8 2.31e8 9.94e8 1.32e8

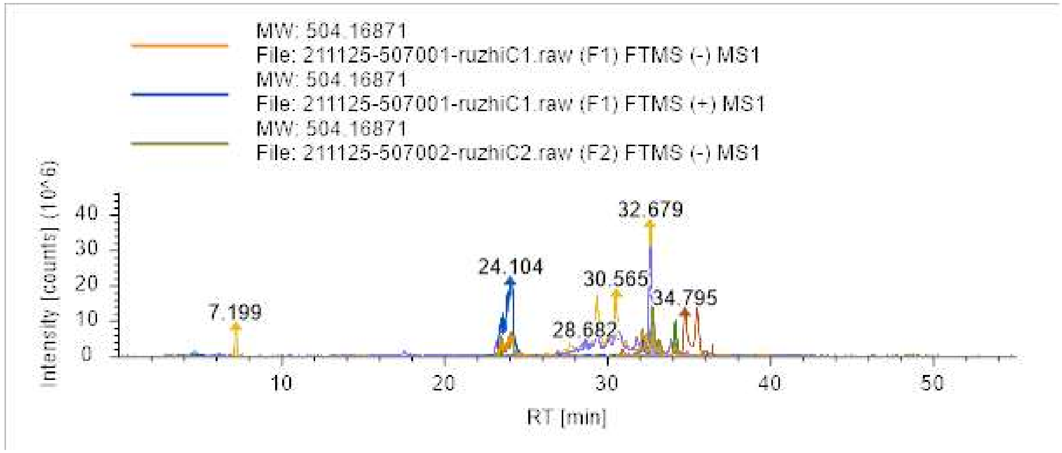

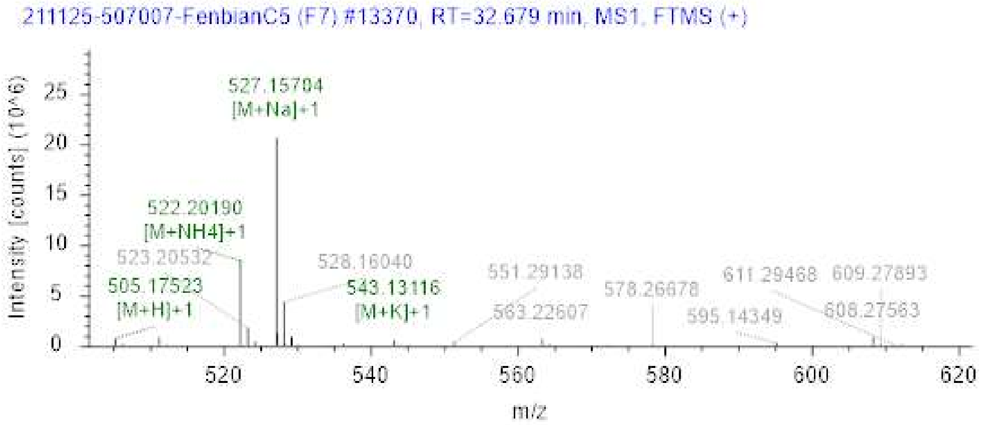

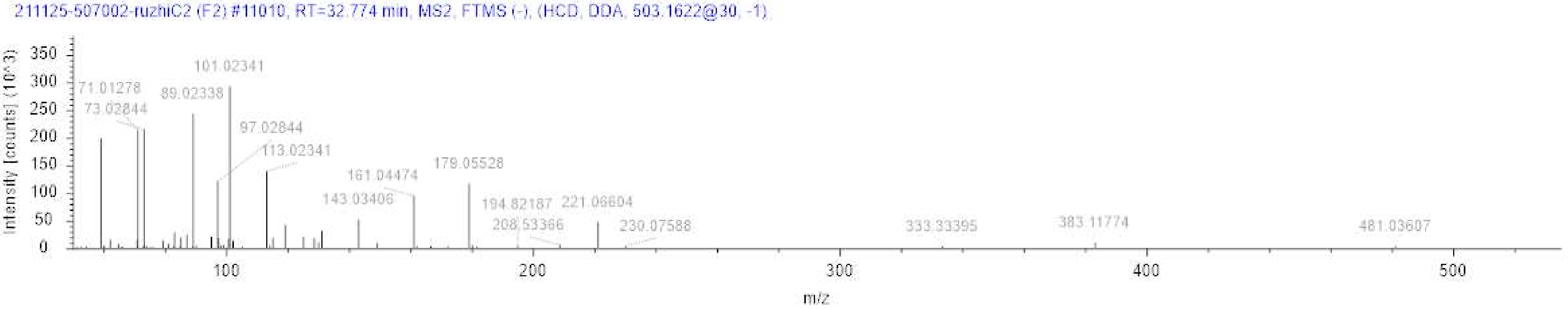

© Reported with Compound Discoverer 3.3

Compounds

Structure	Name	RT [min]	Formula	Calc. MW	Areas
n/a		17.57	n/a	504.16926	2.61e7

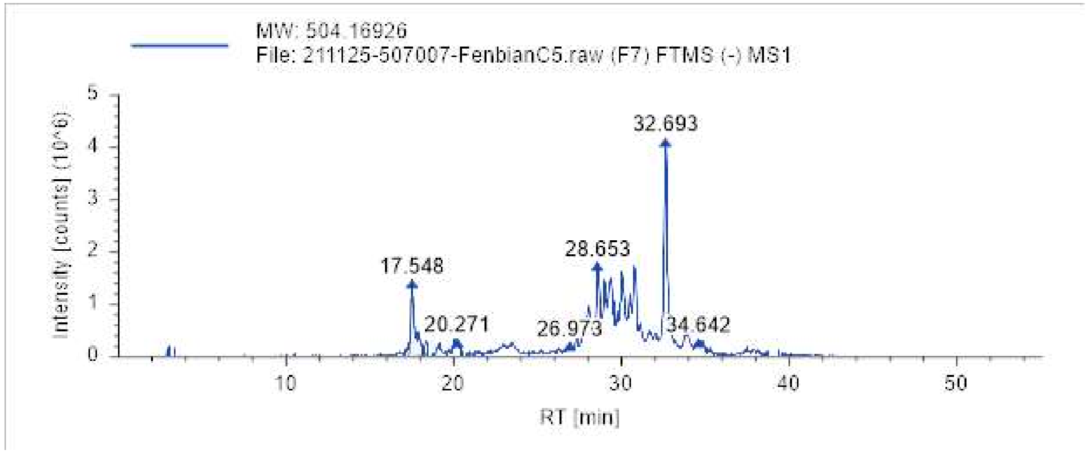

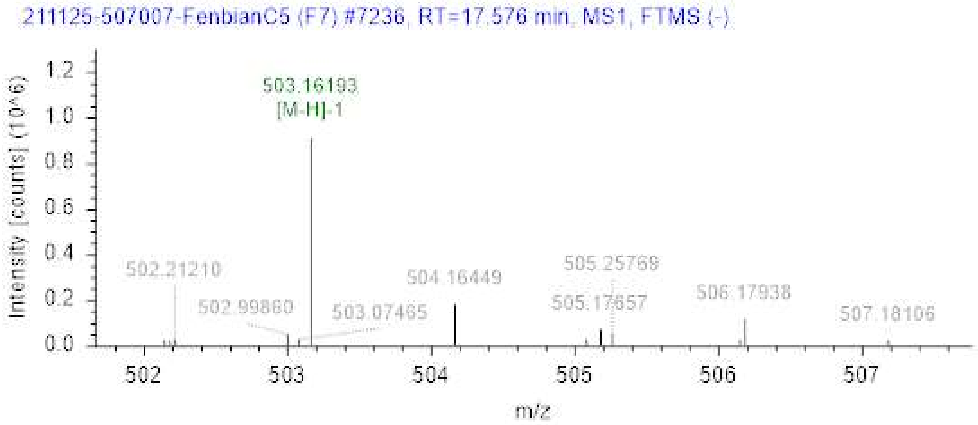

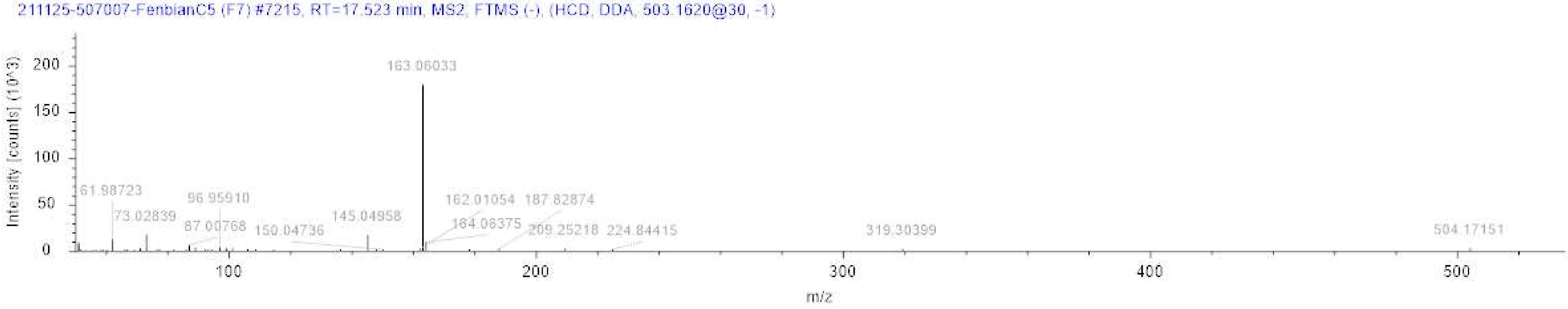

© Reported with Compound Discoverer 3.3

Compounds

Structure	Name	RT [min]	Formula	Calc. MW	Areas
n/a		30.85	n/a	504.16929	1.64e7

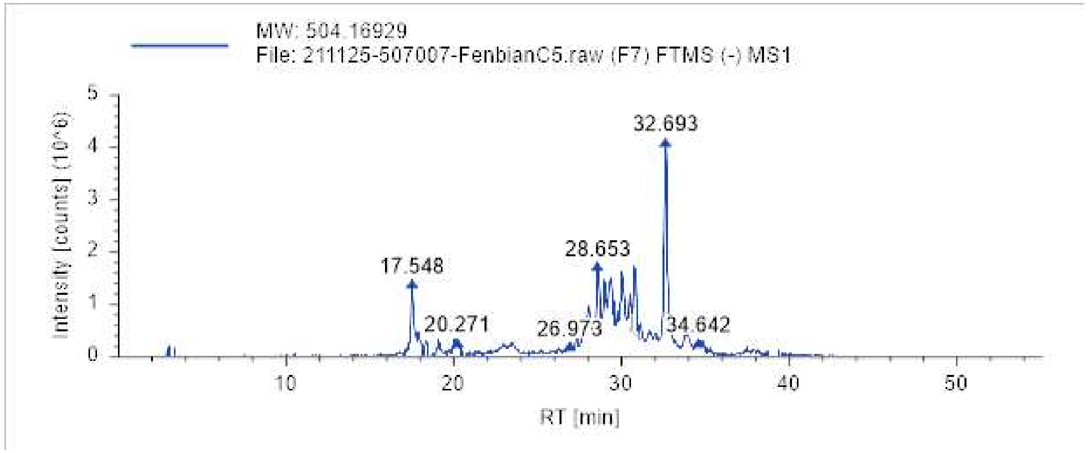

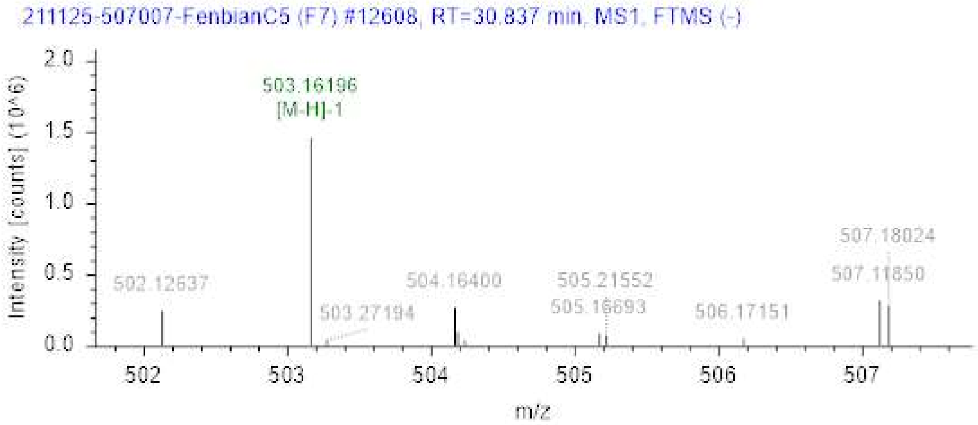

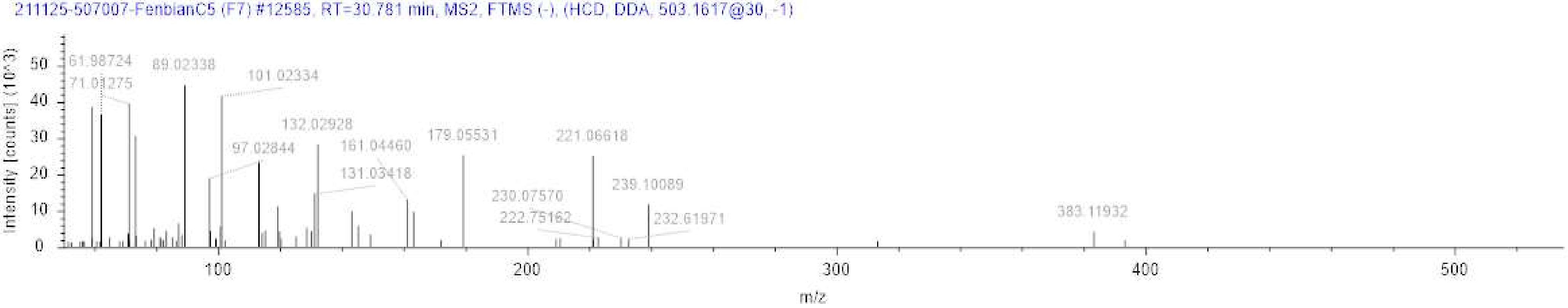

© Reported with Compound Discoverer 3.3

Structure	Name	RT [min]	Formula	Calc. MW	Areas
n/a		28.68	n/a	504.16929	1.30e7

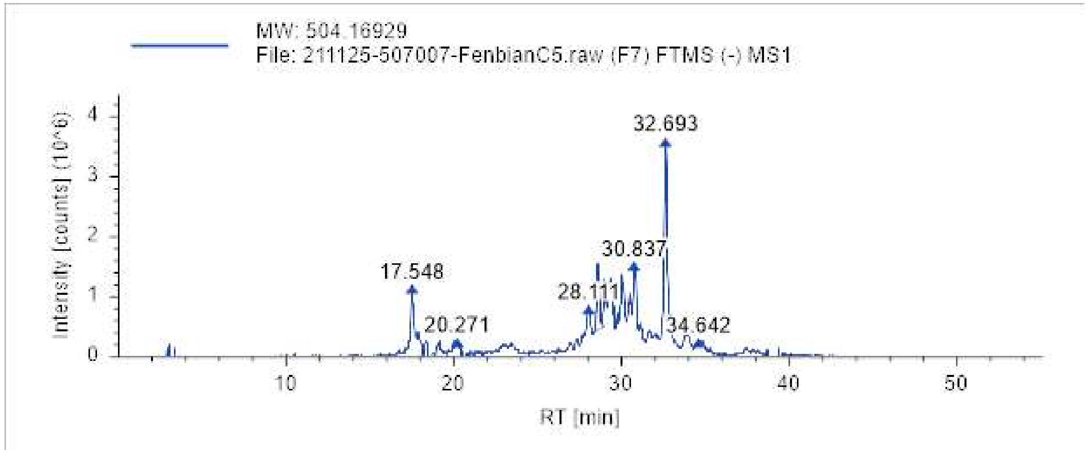

211125-507007-FenbianC5 (F7) #11722, RT=28.682 min, MS1, FTMS (-)

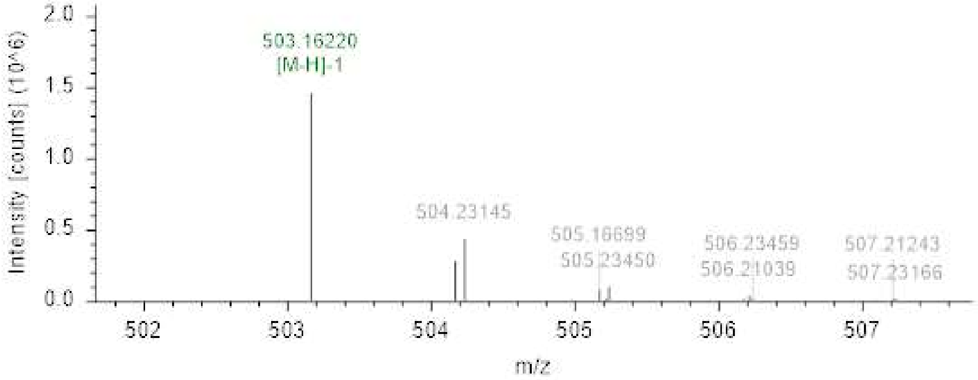

211125-507007-FenbianC5 (F7) #11751, RT=28.748 min, MS2, FTMS (-), (HCD, DDA, 504.2315@30, -1)

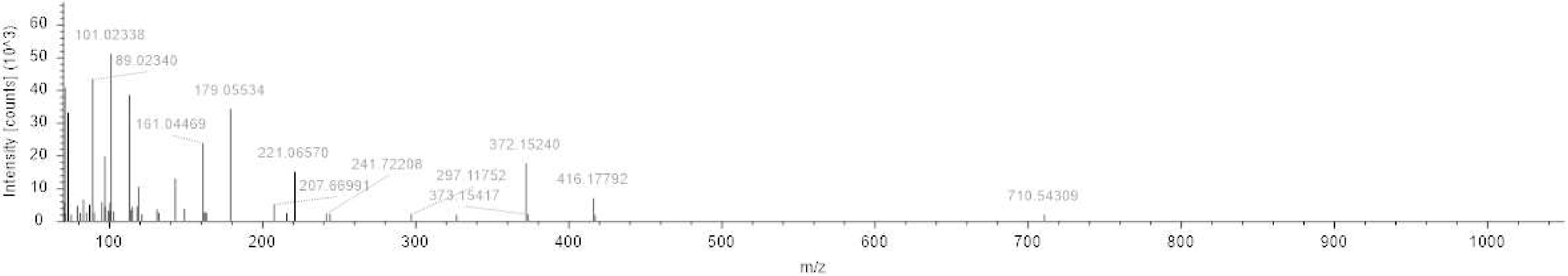

© Reported with Compound Discoverer 3.3

Compounds

Structure	Name	RT [min]	Formula	Calc. MW	Areas
n/a		7.24	n/a	504.17010	2.19e9; 1.52e8

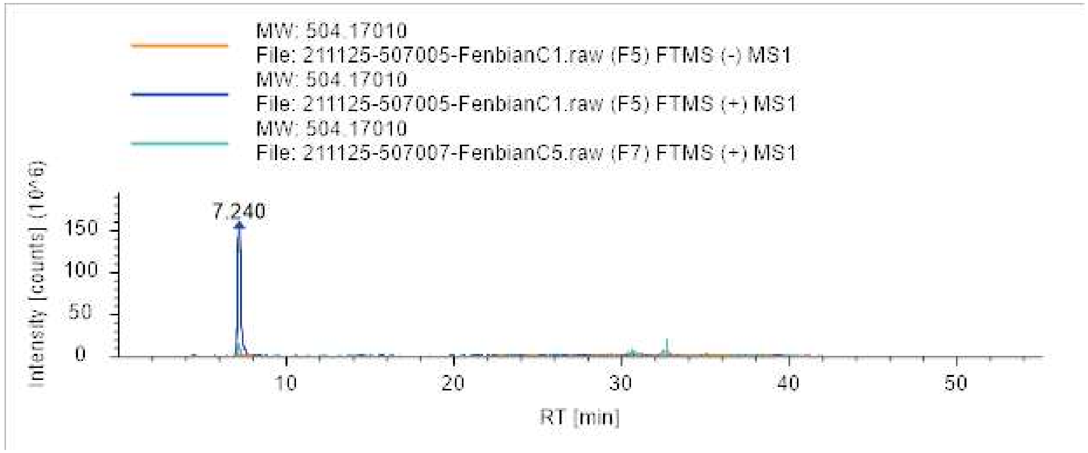

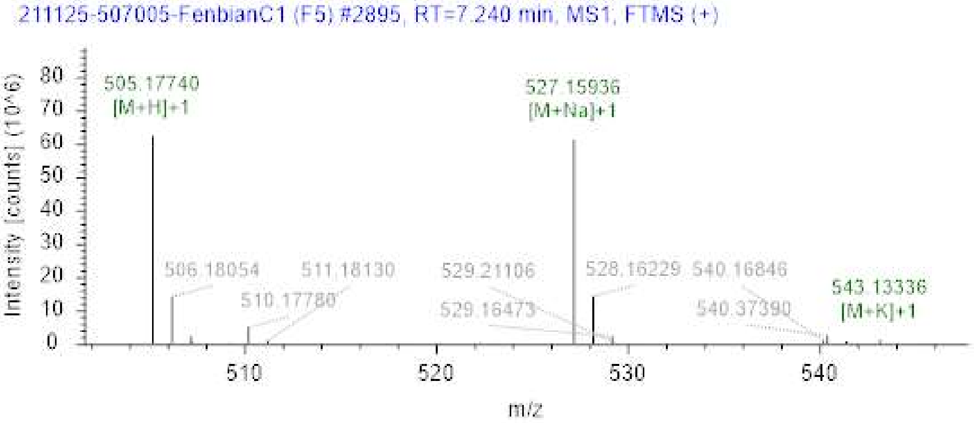

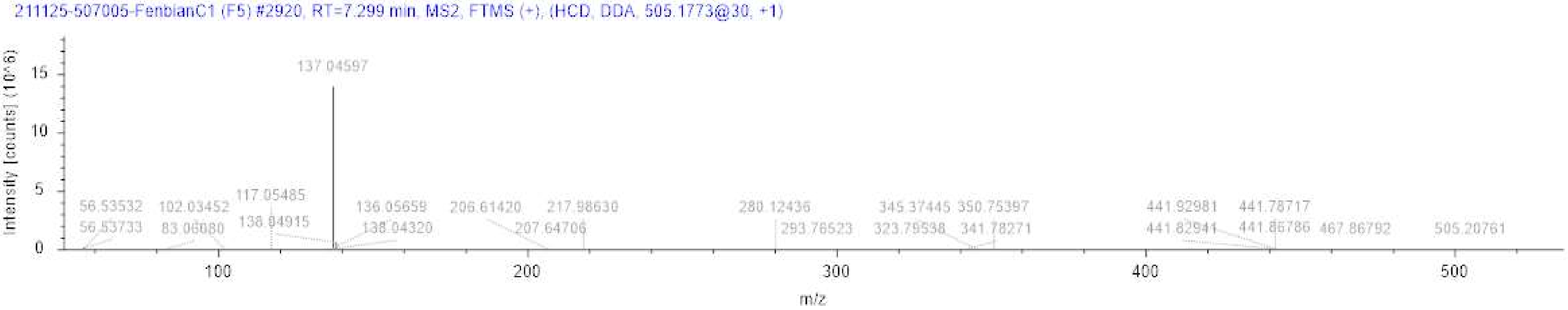

© Reported with Compound Discoverer 3.3

Structure	Name	RT [min]	Formula	Calc. MW	Areas
n/a		28.09	n/a	529.19879	1.16e8; 5.79e9; 1.13e8

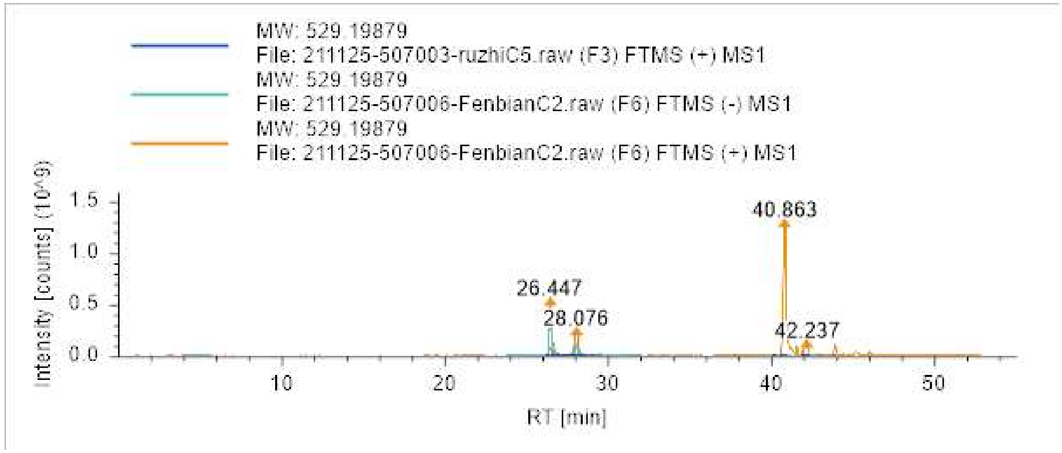

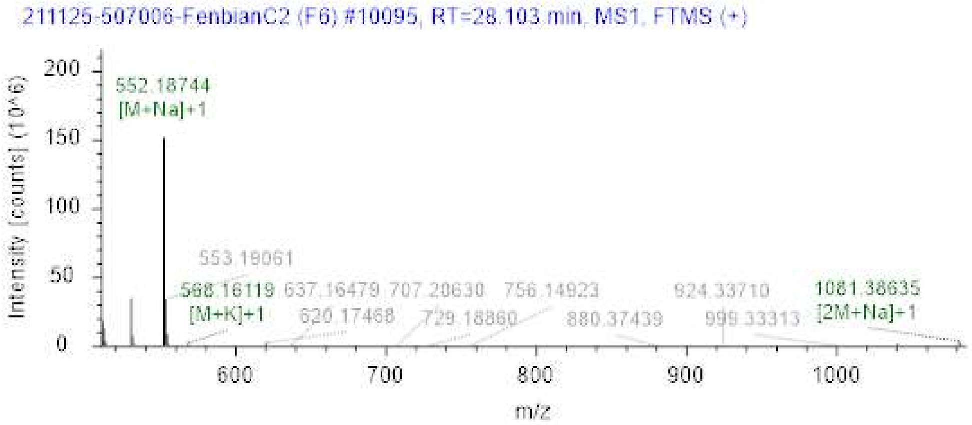

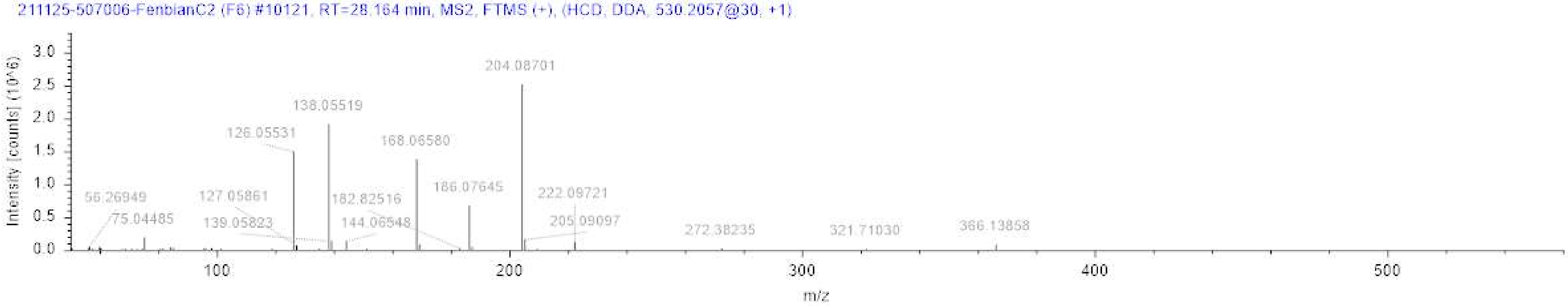

© Reported with Compound Discoverer 3.3

Compounds

Structure	Name	RT [min]	Formula	Calc. MW	Areas
n/a		26.47	n/a	529.19886	4.22e7 4.87e7 9.00e7 5.58e8 9.46e9 1.51e9

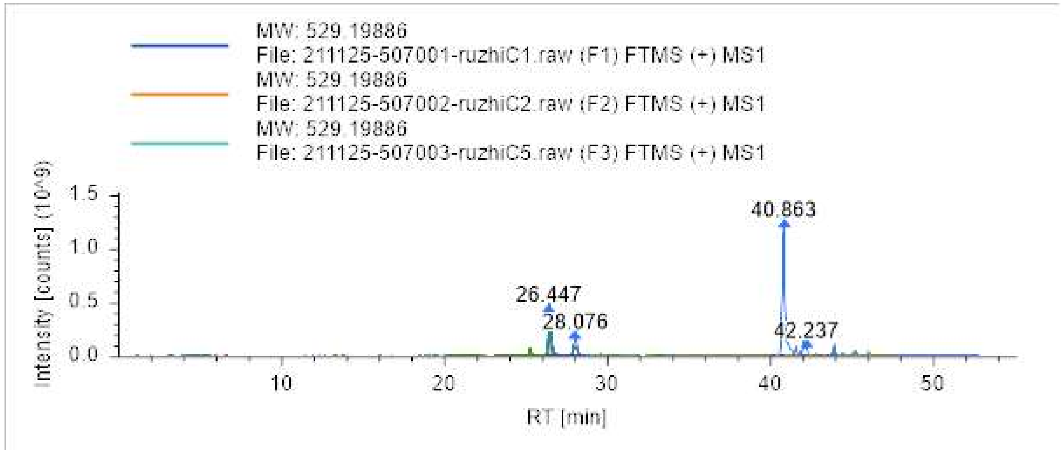

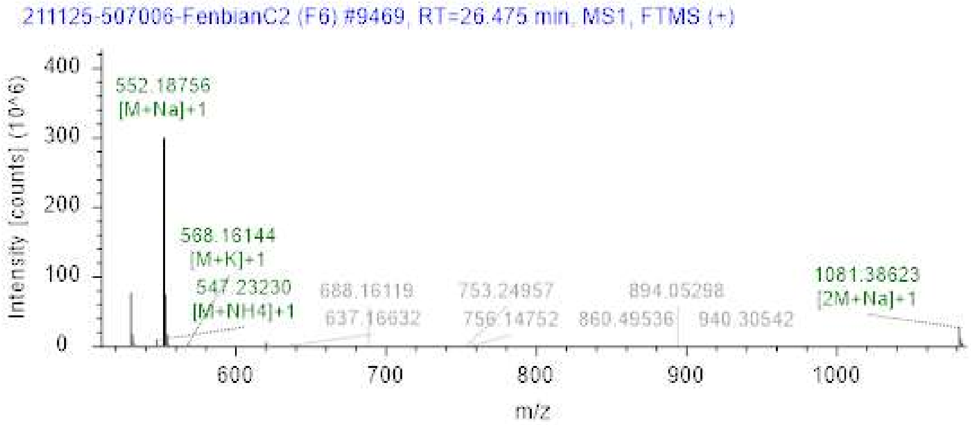

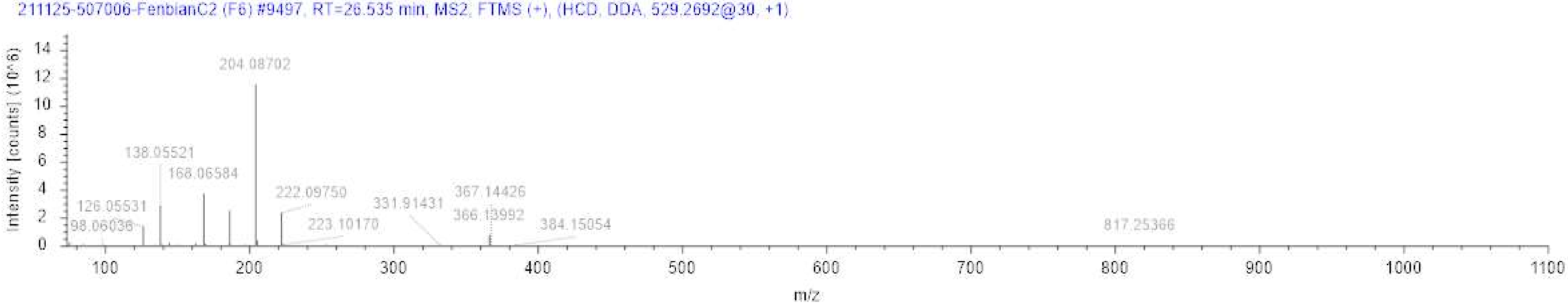

© Reported with Compound Discoverer 3.3

Compounds

Structure	Name	RT [min]	Formula	Calc. MW	Areas
n/a		25.31	n/a	529.19956	1.67e9

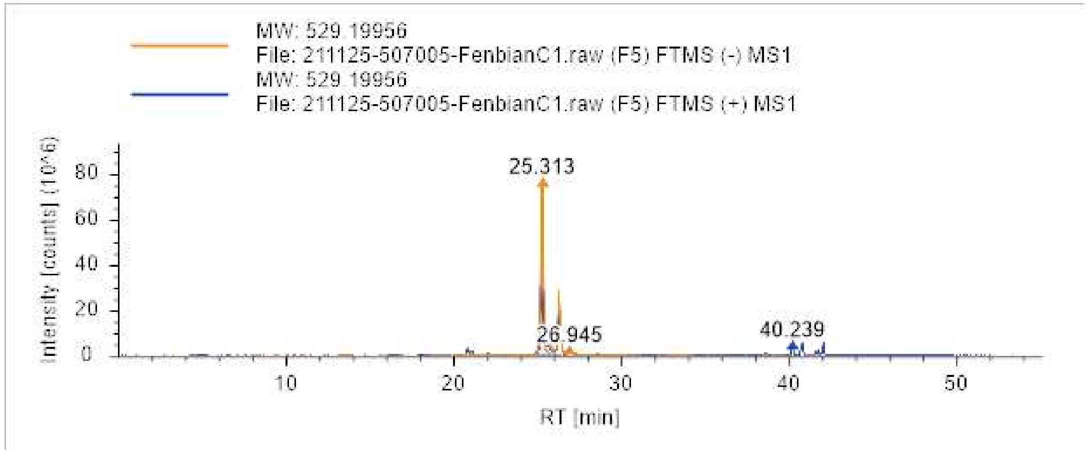

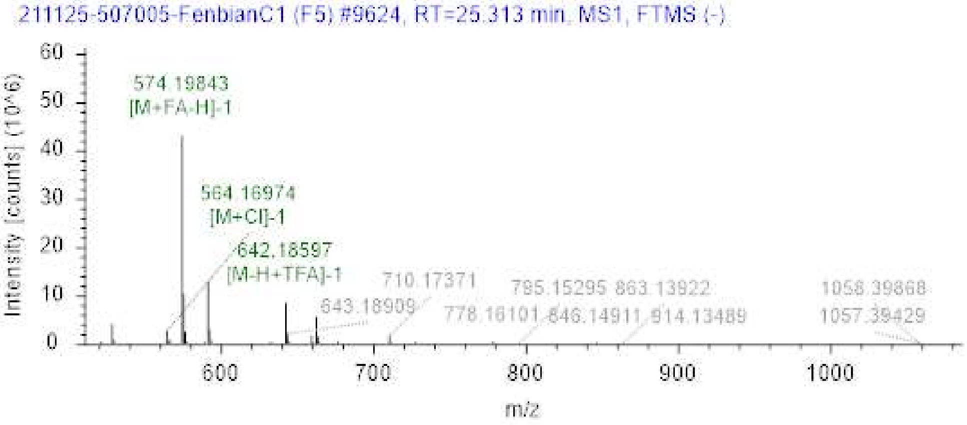

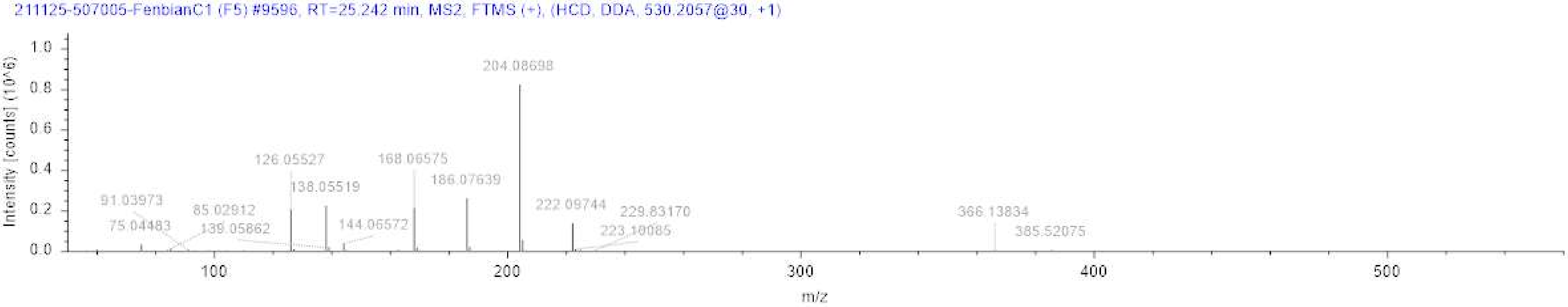

© Reported with Compound Discoverer 3.3

Compounds

Structure	Name	RT [min]	Formula	Calc. MW	Areas
n/a		31.14	n/a	529.20018	2.08e7

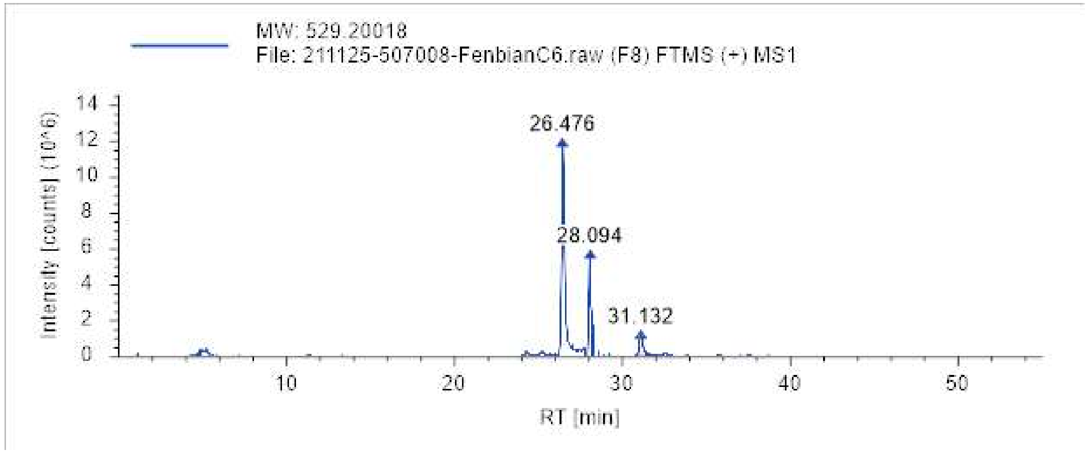

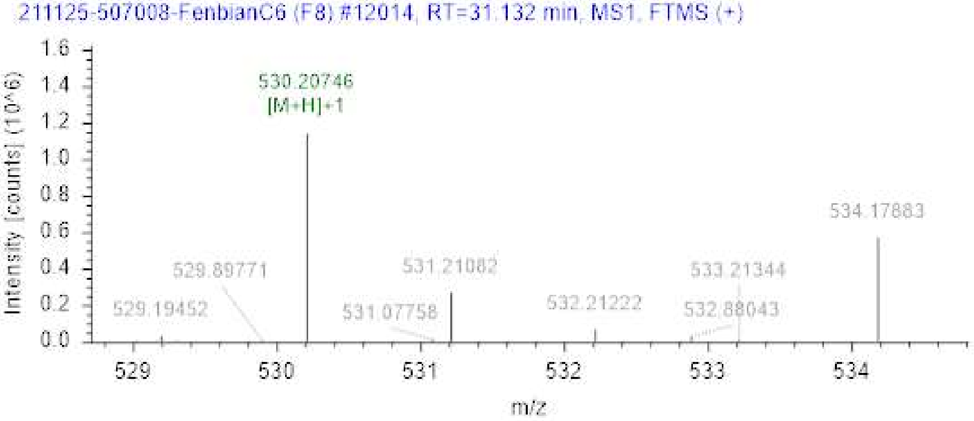

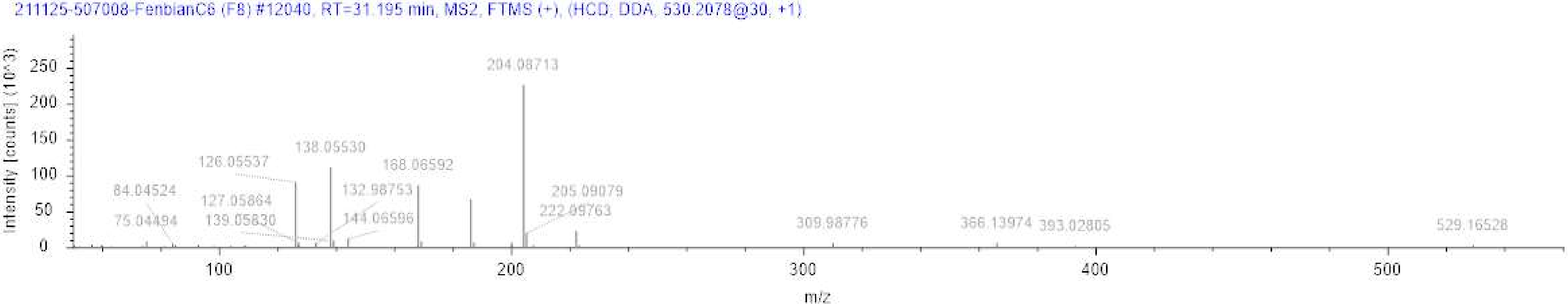

© Reported with Compound Discoverer 3.3

Structure	Name	RT [min]	Formula	Calc. MW	Areas
n/a		42.25	n/a	529.20050	4.88e7

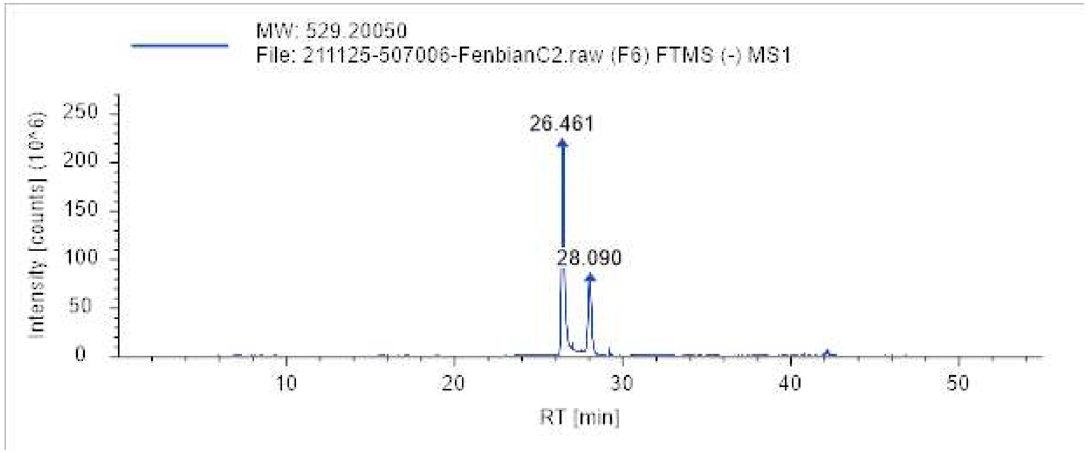

211125-507006-FenbianC2 (F6) #15548, RT=42.250 min, MS1, FTMS (-)

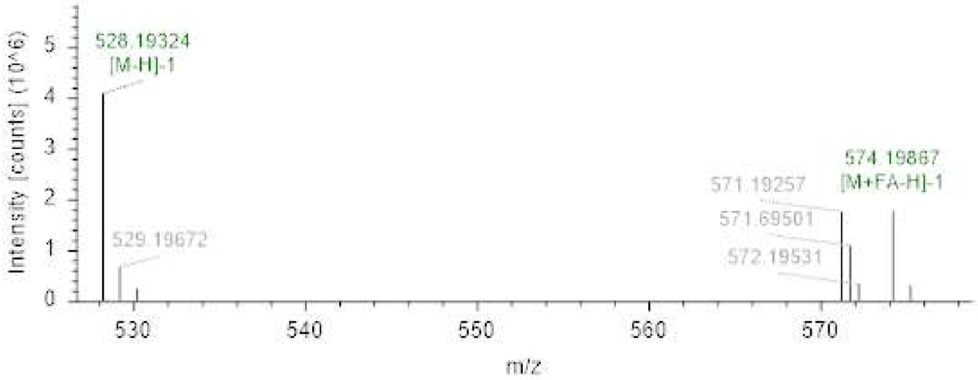

211125-507006-FenbianC2 (F6) #15552, RT=42.256 min, MS2, FTMS (-), (HCD, DDA, 528.1932@30, -1)

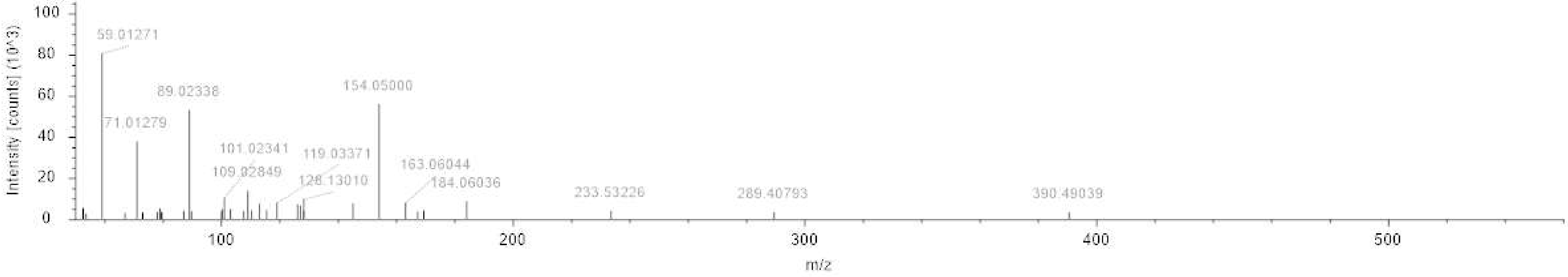

© Reported with Compound Discoverer 3.3

Compounds

Structure	Name	RT [min]	Formula	Calc. MW	Areas
n/a		36.84	n/a	545.19396	4.82e7

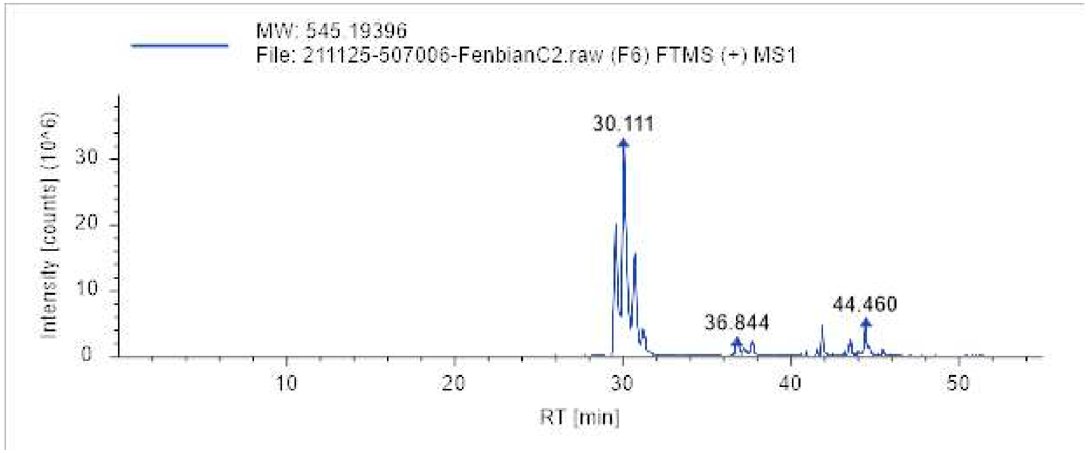

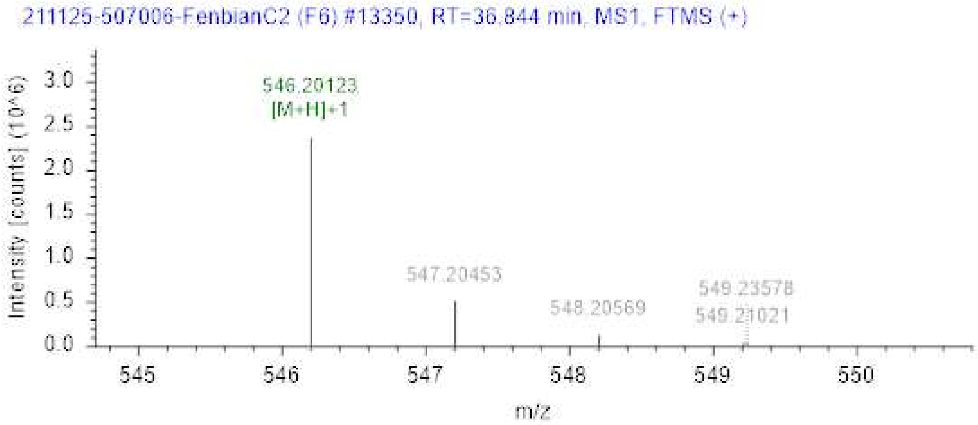

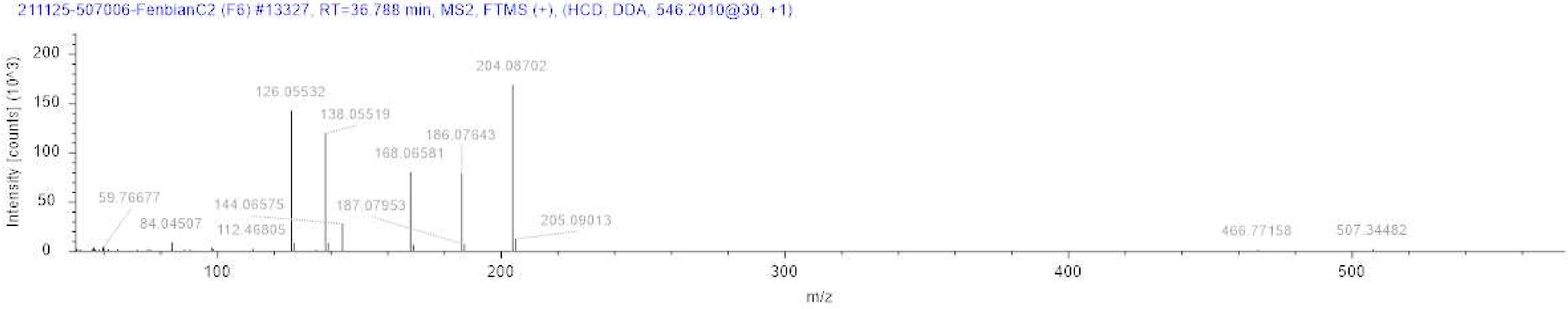

© Reported with Compound Discoverer 3.3

Compounds

Structure	Name	RT [min]	Formula	Calc. MW	Areas
n/a		37.74	n/a	545.19396	3.84e7

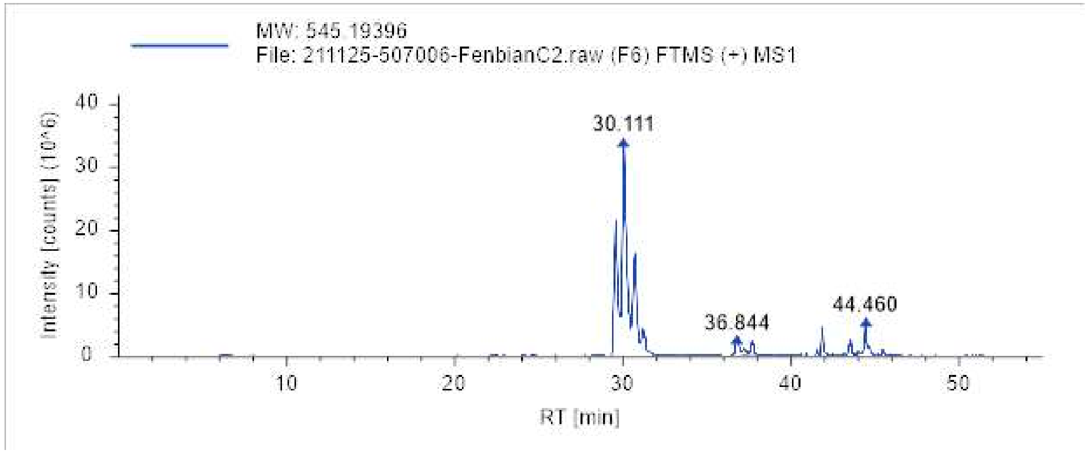

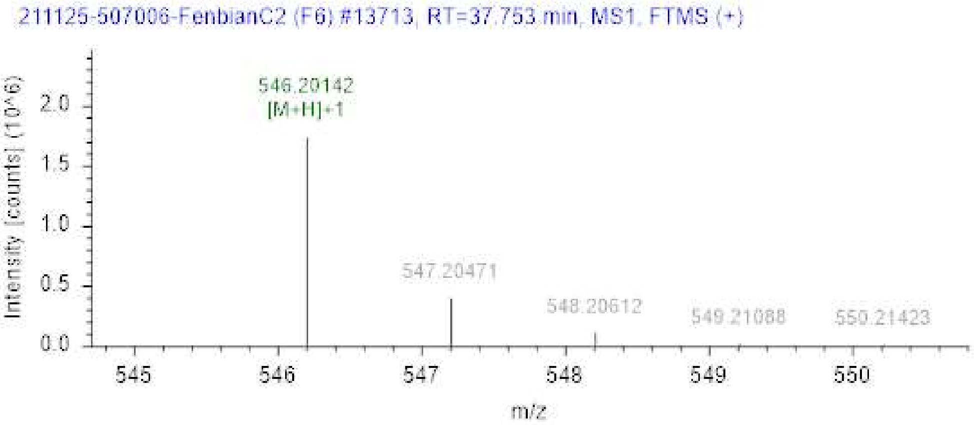

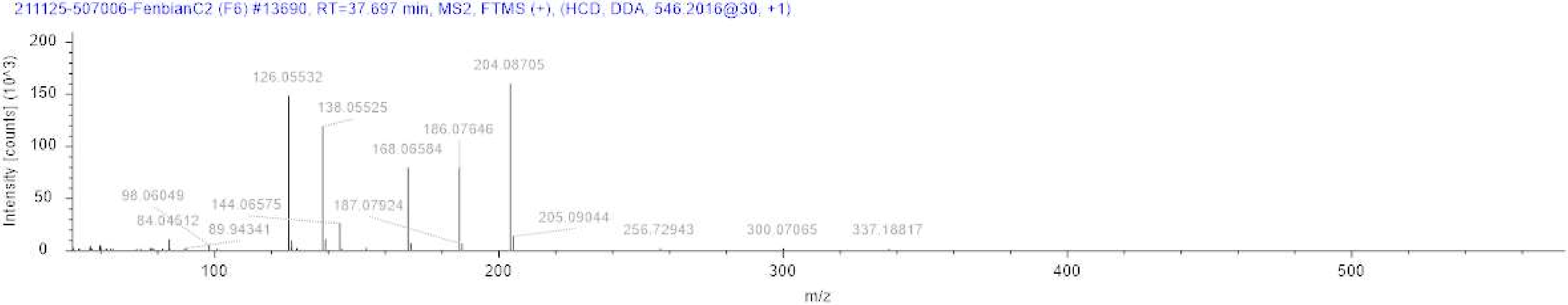

© Reported with Compound Discoverer 3.3

Compounds

Structure	Name	RT [min]	Formula	Calc. MW	Areas
n/a		28.45	n/a	545.19420	8.89e8

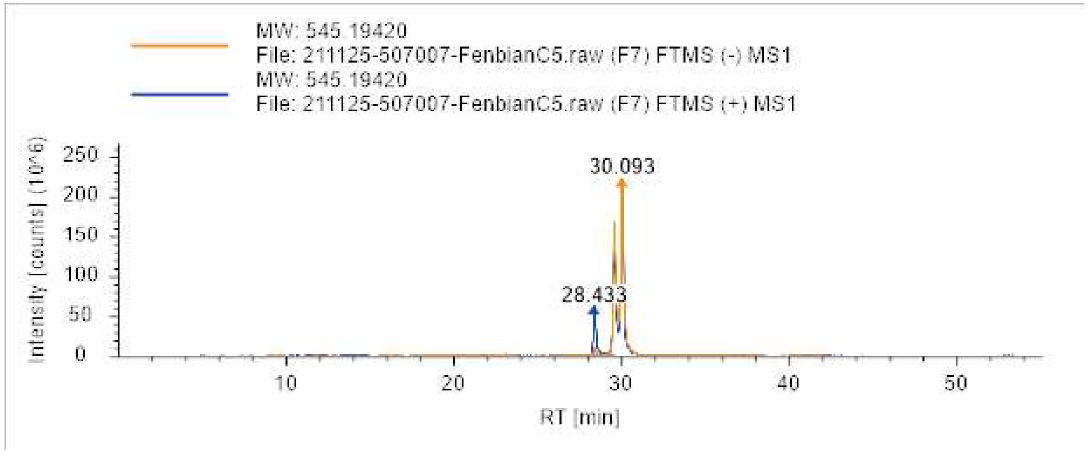

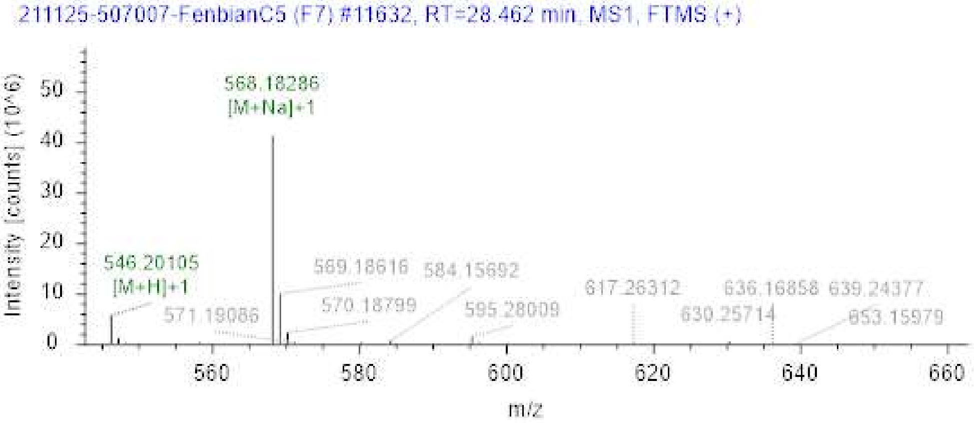

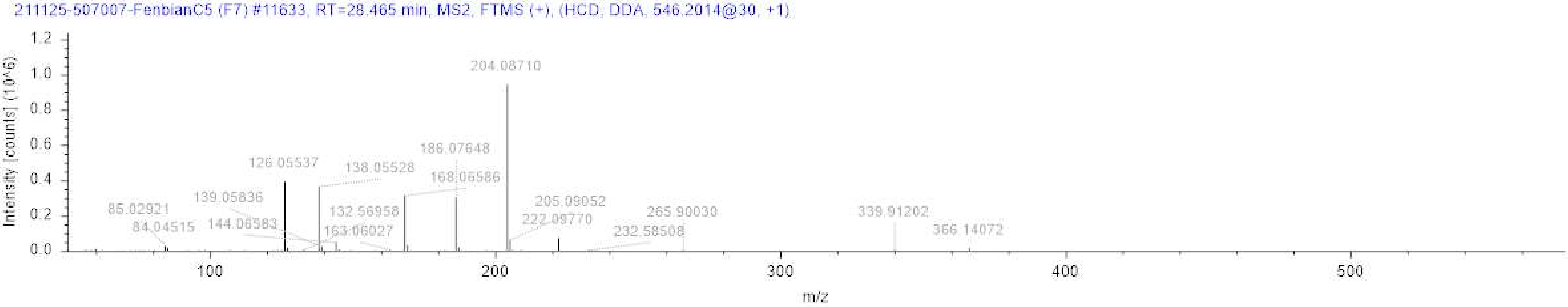

© Reported with Compound Discoverer 3.3

Structure	Name	RT [min]	Formula	Calc. MW	Areas
n/a		43.60	n/a	545.19442	2.95e7

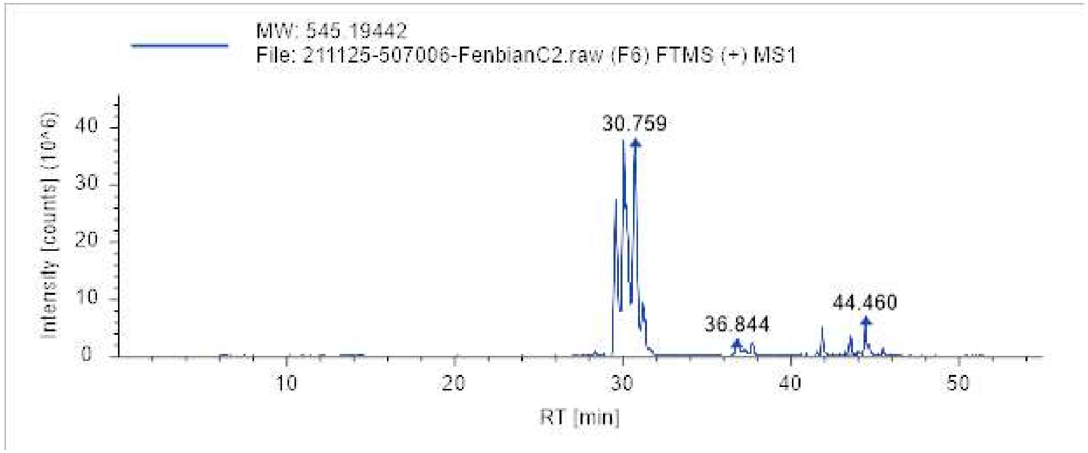

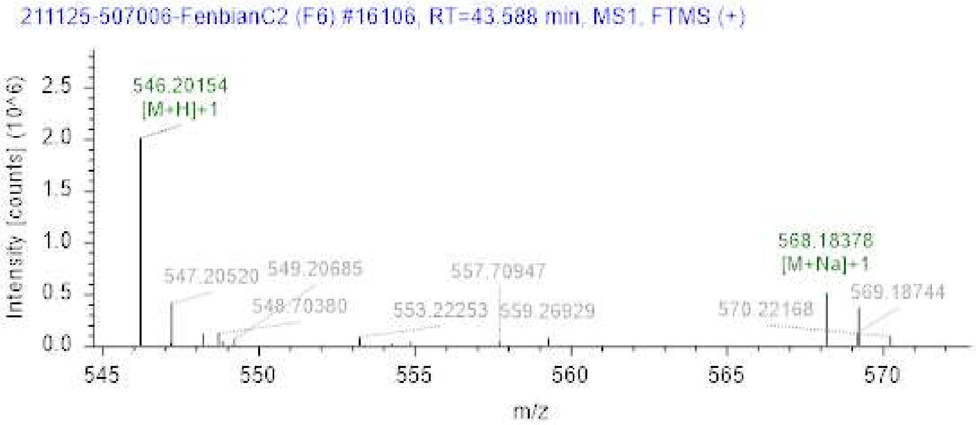

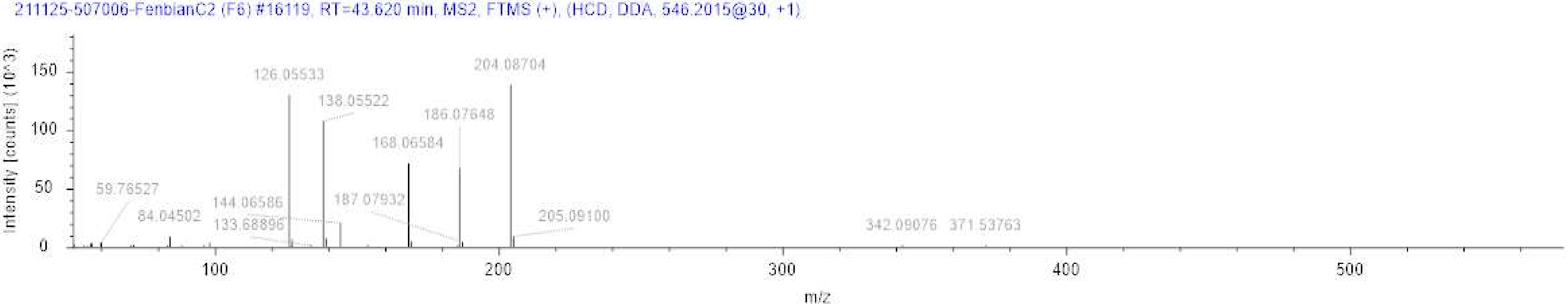

© Reported with Compound Discoverer 3.3

Structure	Name	RT [min]	Formula	Calc. MW	Areas
n/a		41.91	n/a	545.19451	4.19e7; 1.36e7

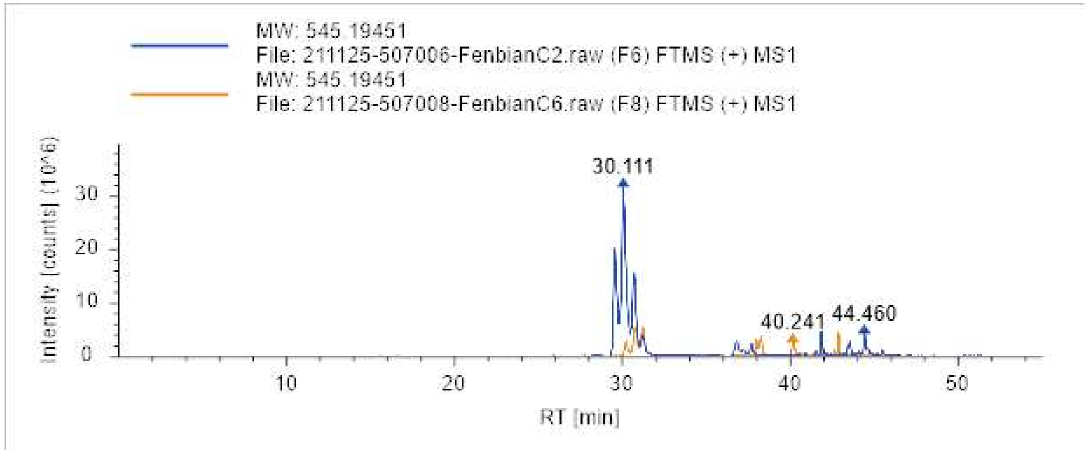

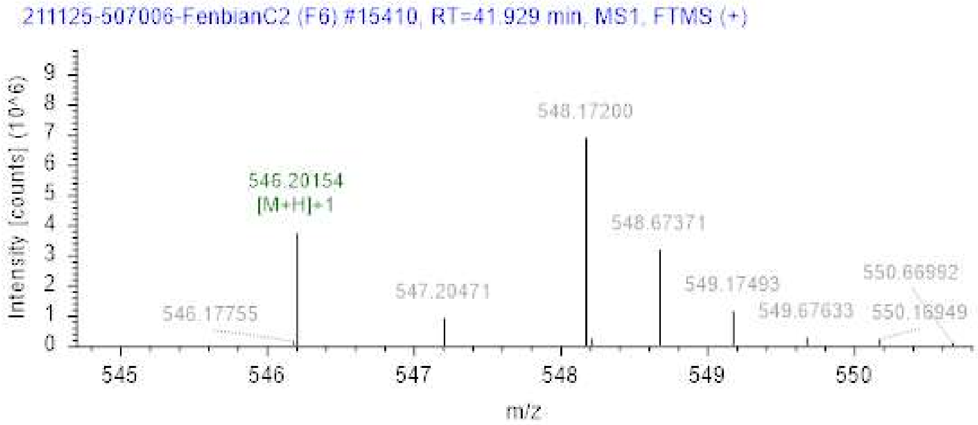

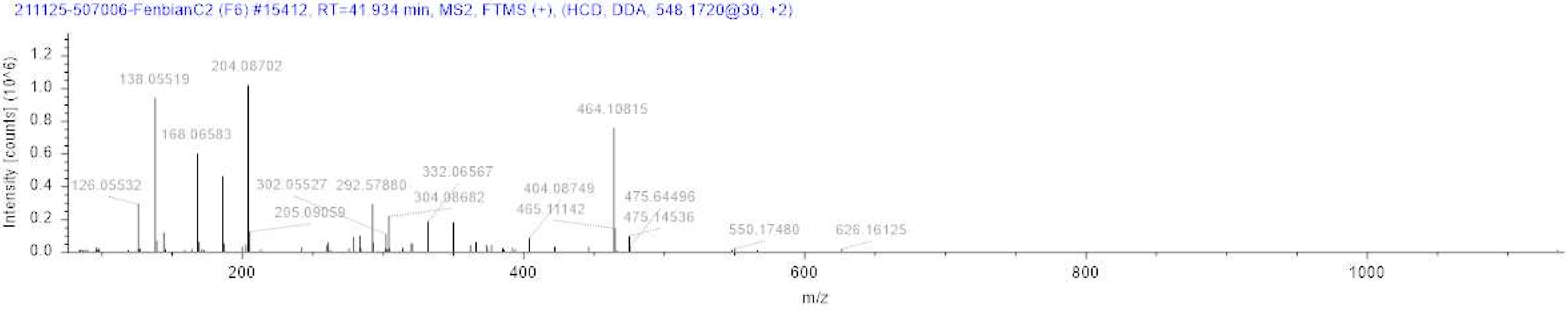

© Reported with Compound Discoverer 3.3

Compounds

Structure	Name	RT [min]	Formula	Calc. MW	Areas
n/a		42.92	n/a	545.19451	2.94e7

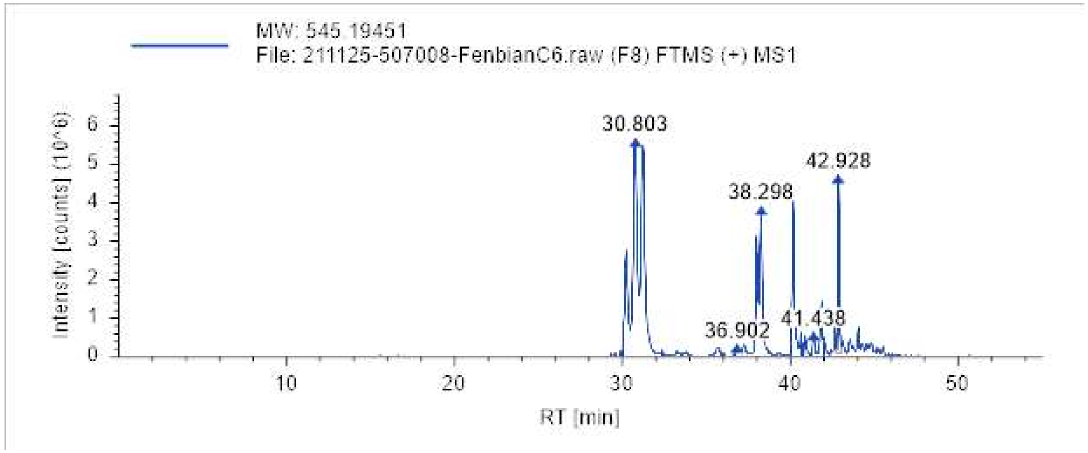

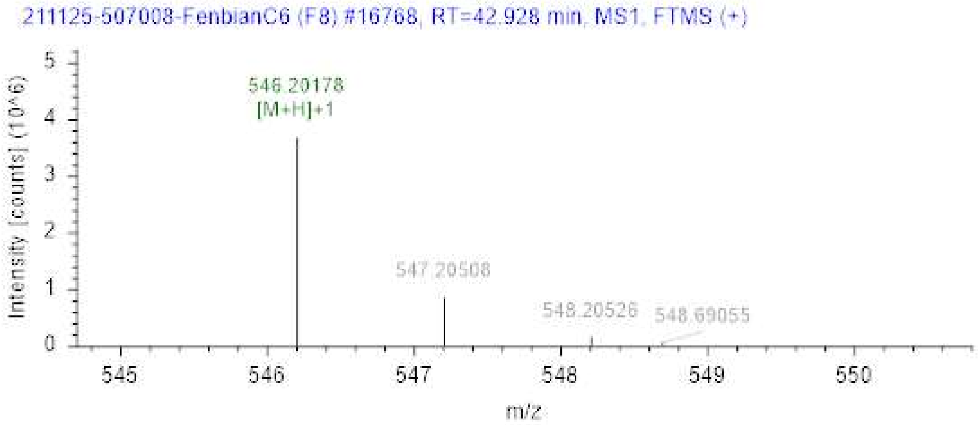

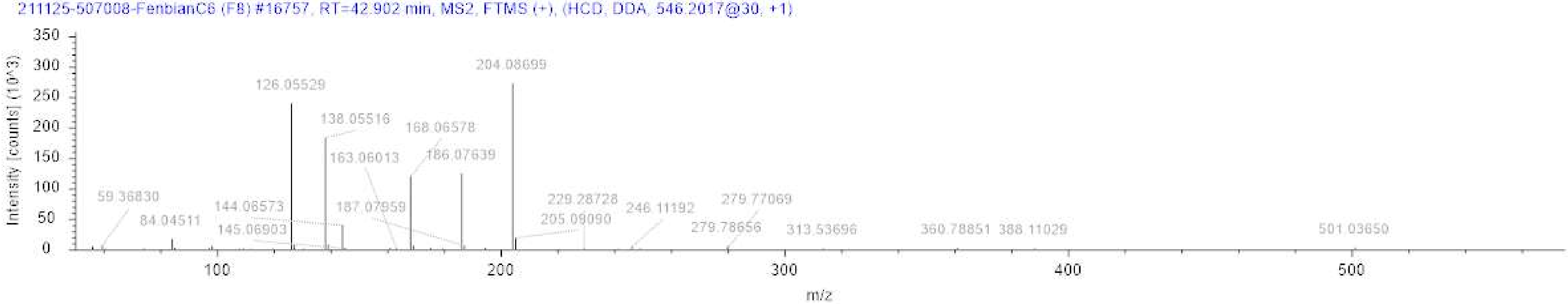

© Reported with Compound Discoverer 3.3

Compounds

Structure	Name	RT [min]	Formula	Calc. MW	Areas
n/a		45.52	n/a	545.19451	1.11e7

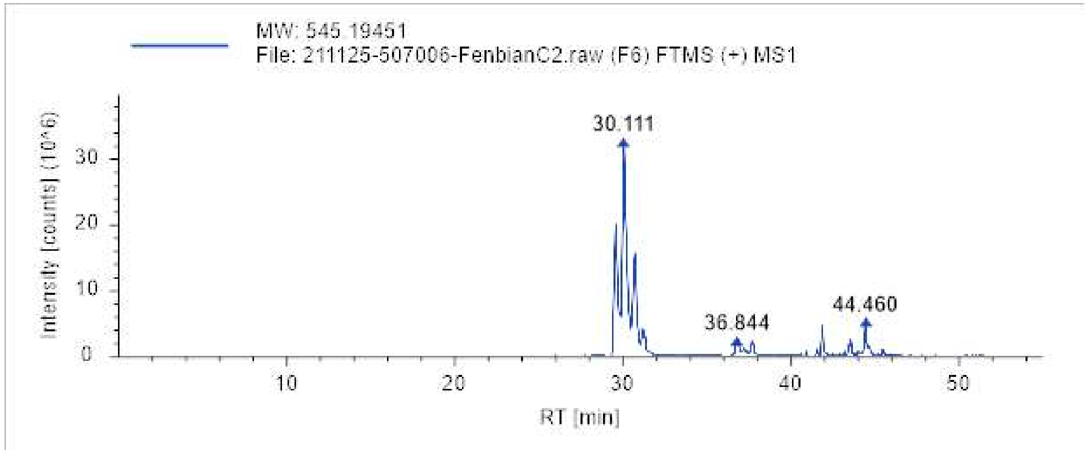

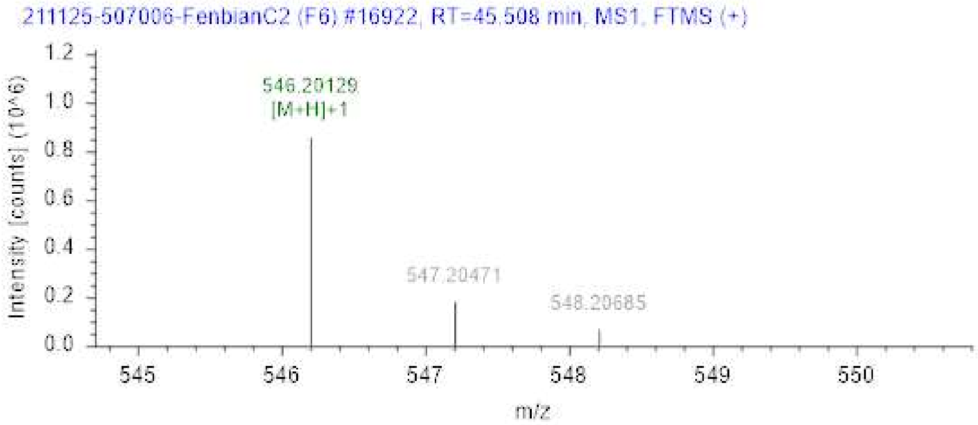

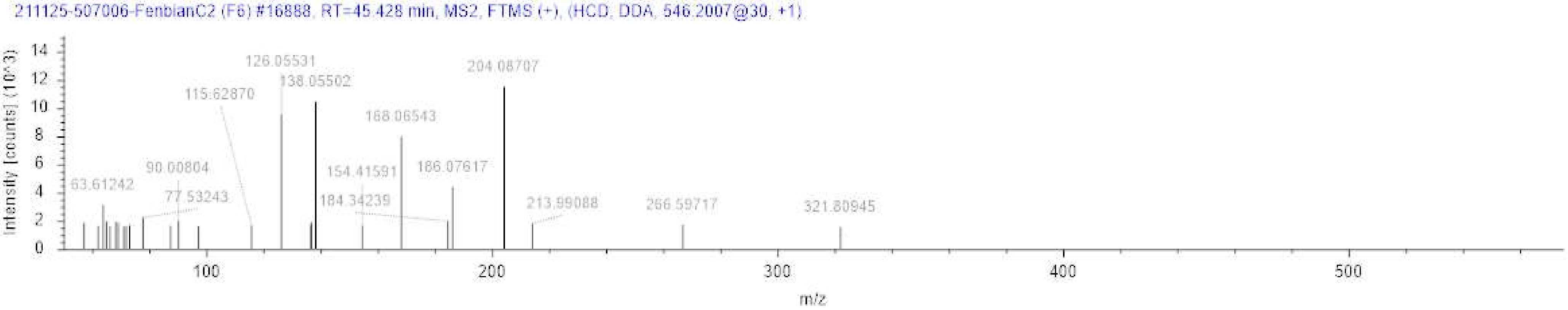

© Reported with Compound Discoverer 3.3

Compounds

Structure	Name	RT [min]	Formula	Calc. MW	Areas
n/a		30.78	n/a	545.19451	1.19e7 1.86e8 3.71e8

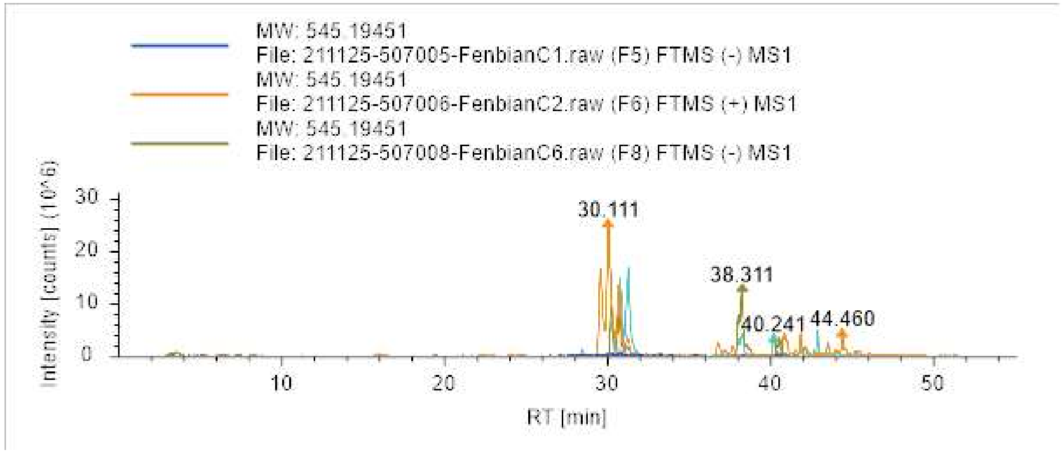

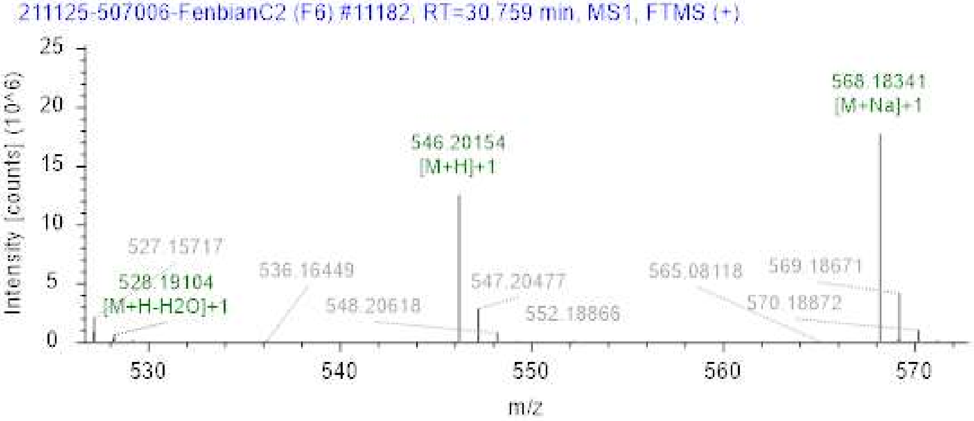

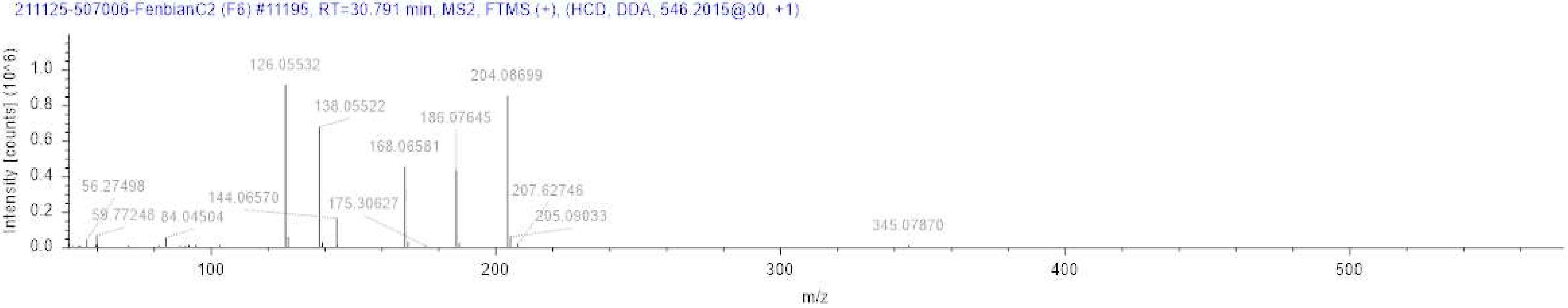

© Reported with Compound Discoverer 3.3

Structure	Name	RT [min]	Formula	Calc. MW	Areas
n/a		44.46	n/a	545.19455	6.70e7

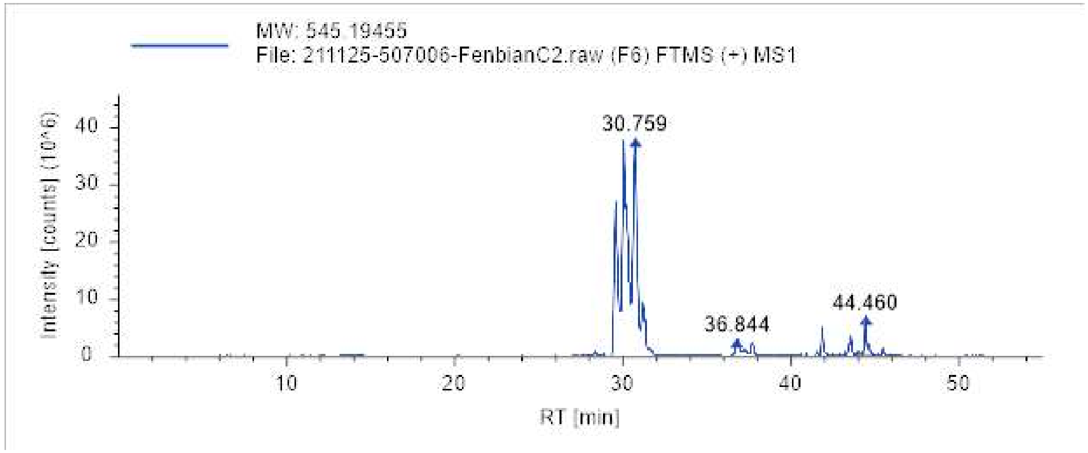

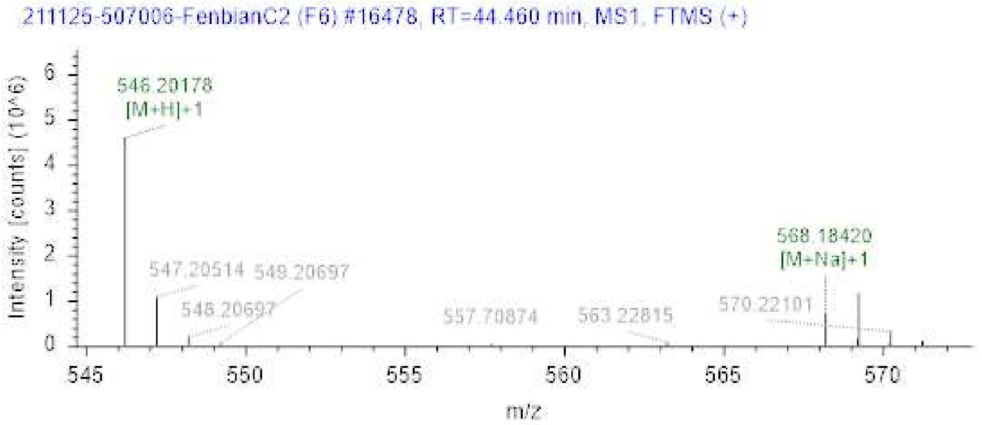

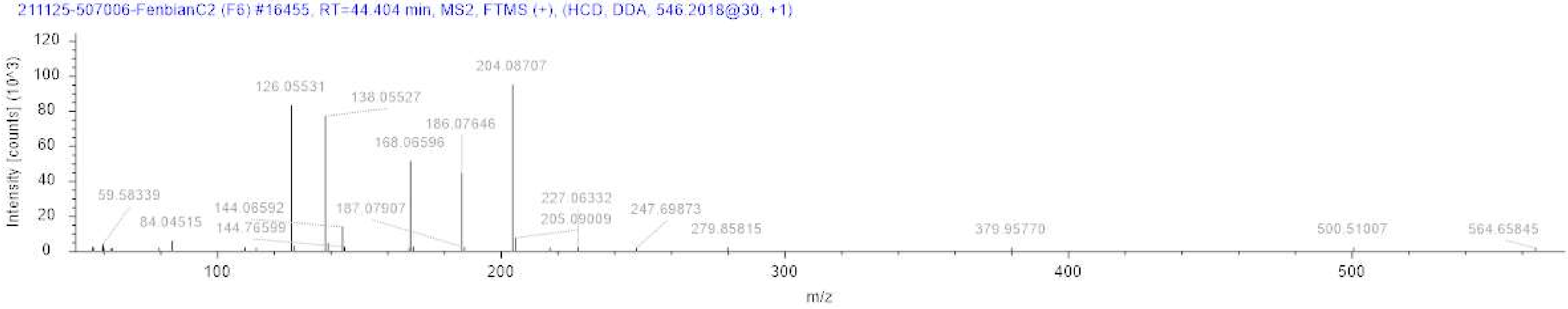

© Reported with Compound Discoverer 3.3

Compounds

Structure	Name	RT [min]	Formula	Calc. MW	Areas
n/a		29.64	n/a	545.19485	8.19e8 3.83e9

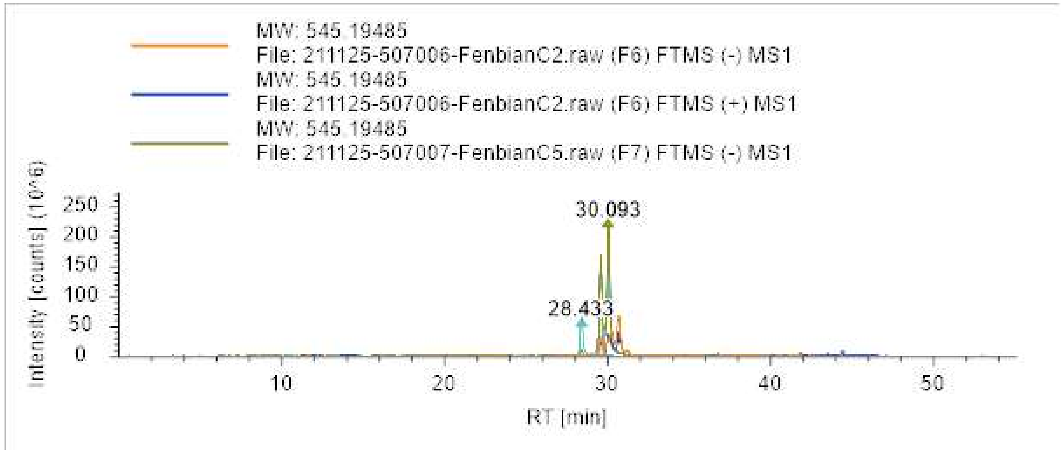

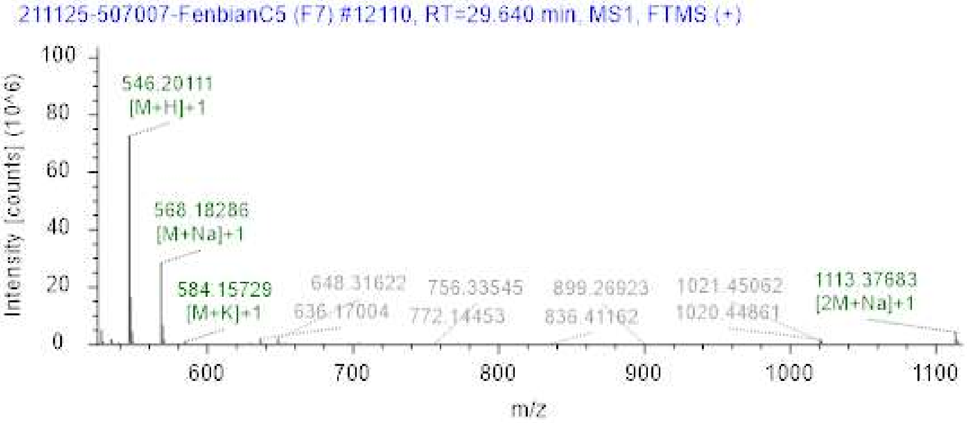

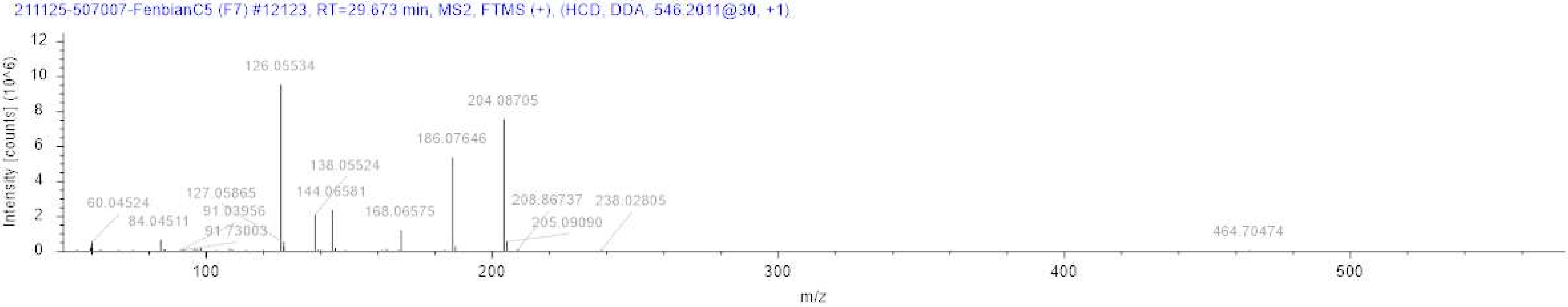

© Reported with Compound Discoverer 3.3

Structure	Name	RT [min]	Formula	Calc. MW	Areas
n/a		38.30	n/a	545.19487	1.02e8 2.22e8 3.33e7 3.34e7 3.61e8

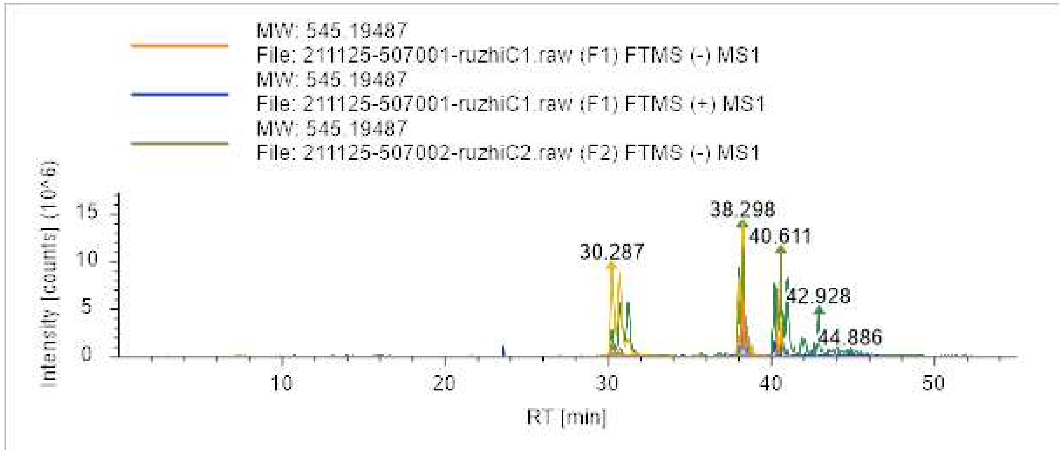

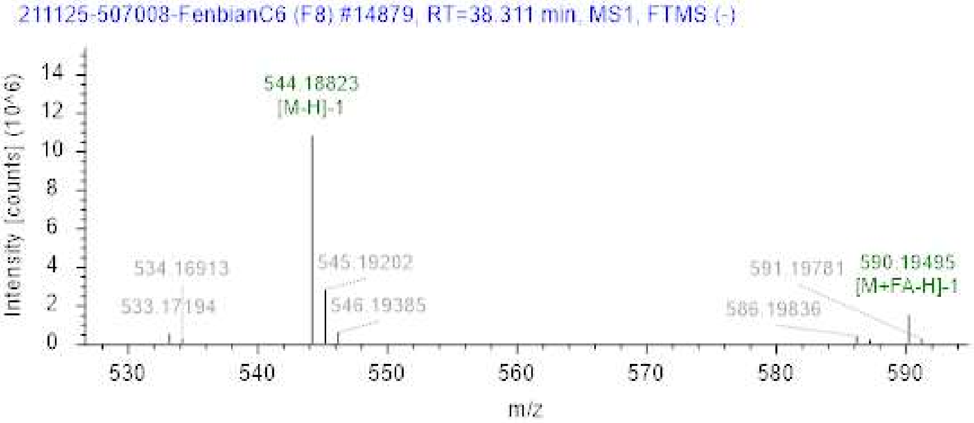

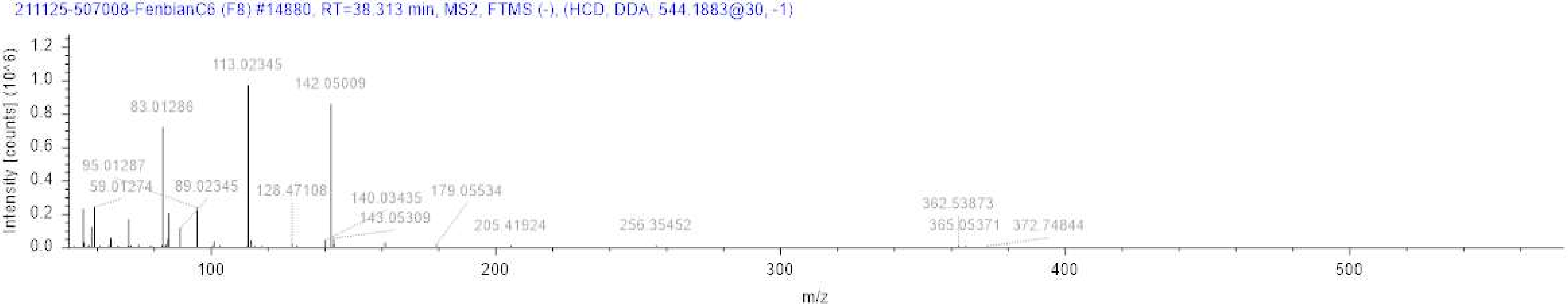

© Reported with Compound Discoverer 3.3

Compounds

Structure	Name	RT [min]	Formula	Calc. MW	Areas
n/a		40.62	n/a	545.19541	2.03e7 | 1.02e8 | 1.71e7 | 1.23e7 | | | | 5.62e7 |

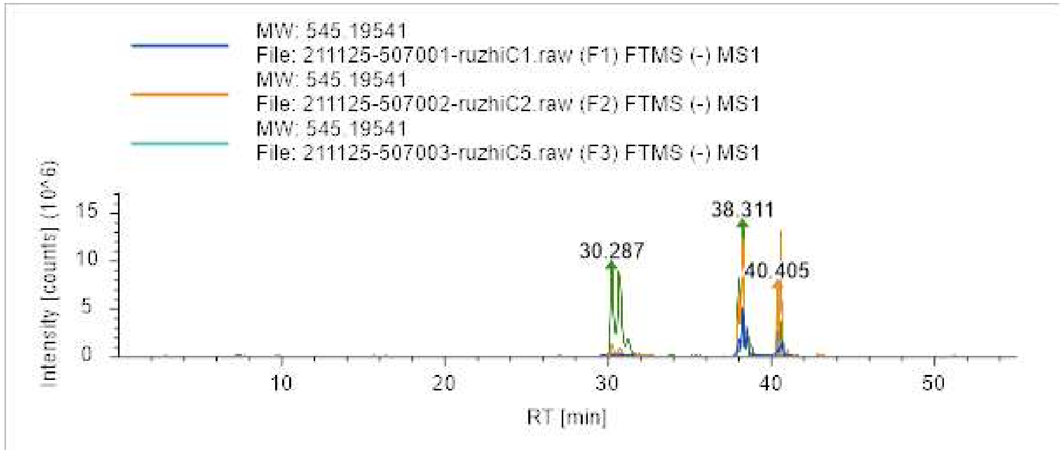

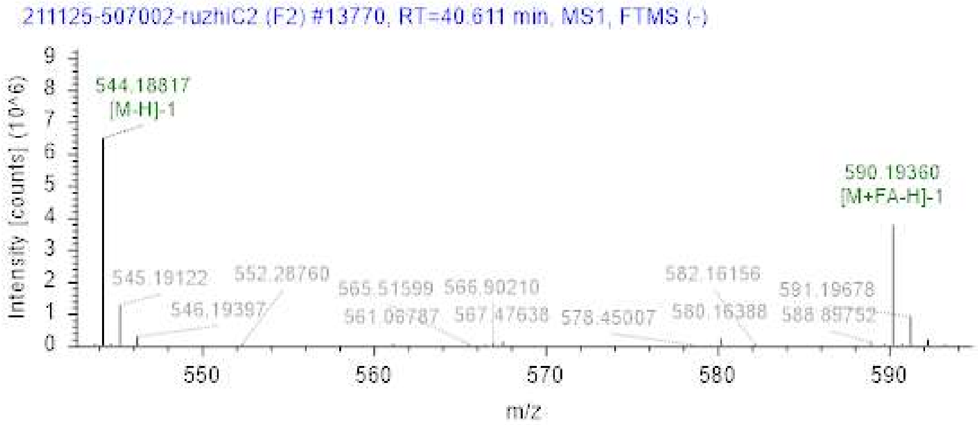

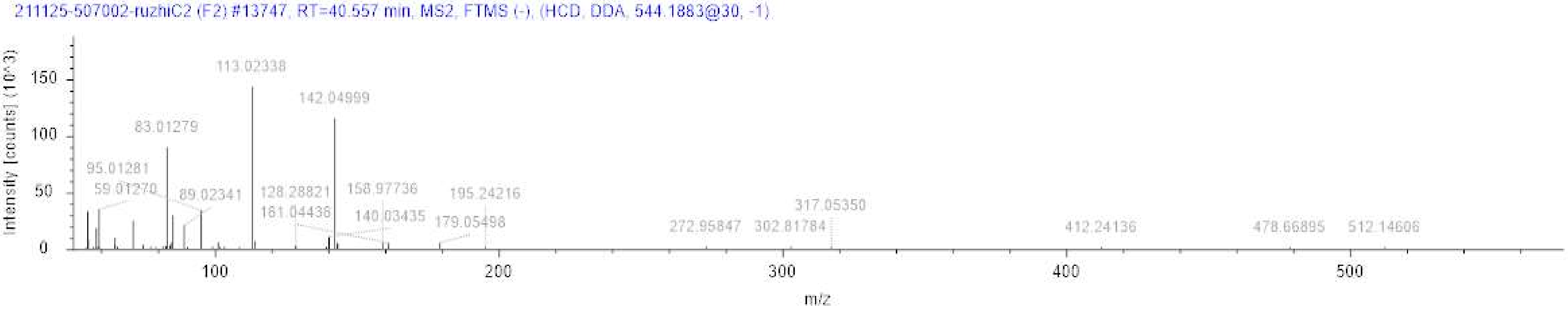

© Reported with Compound Discoverer 3.3

Compounds

Structure	Name	RT [min]	Formula	Calc. MW	Areas
n/a		31.28	n/a	545.19542	9.03e7

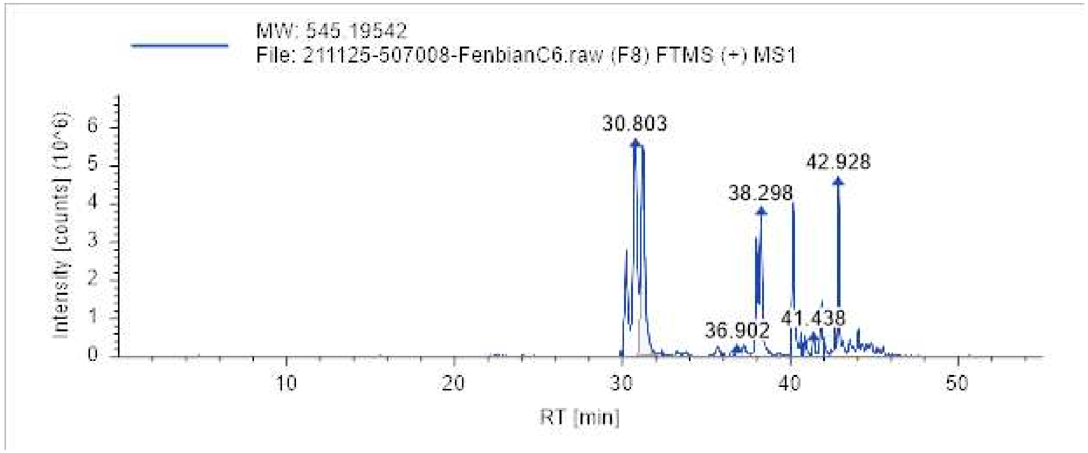

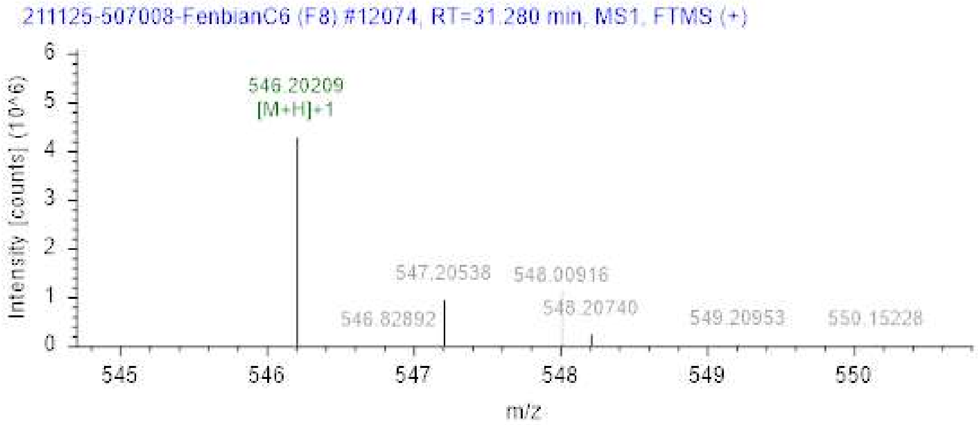

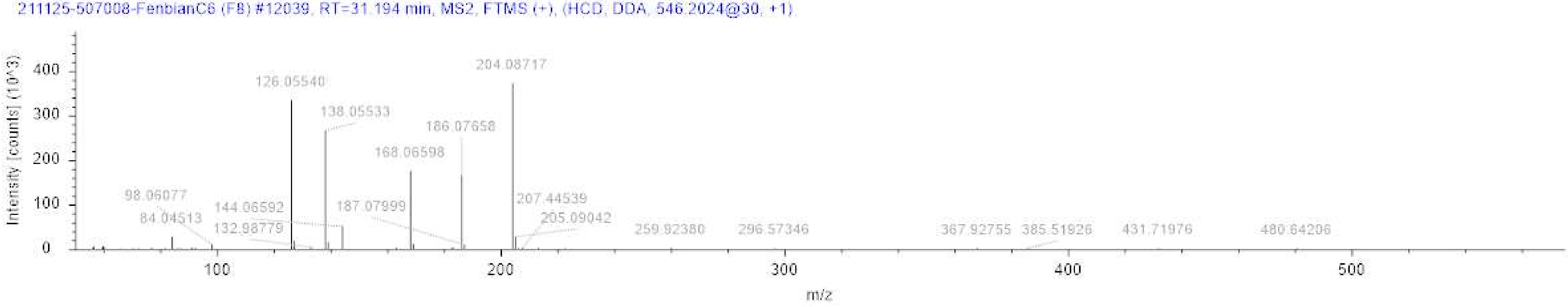

© Reported with Compound Discoverer 3.3

Compounds

Structure	Name	RT [min]	Formula	Calc. MW	Areas
n/a		21.39	n/a	547.21120	8.47e7

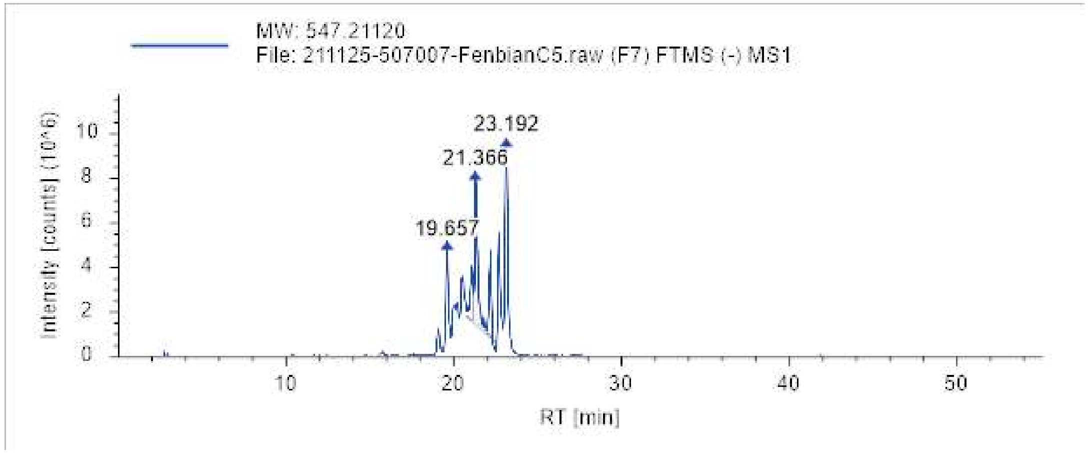

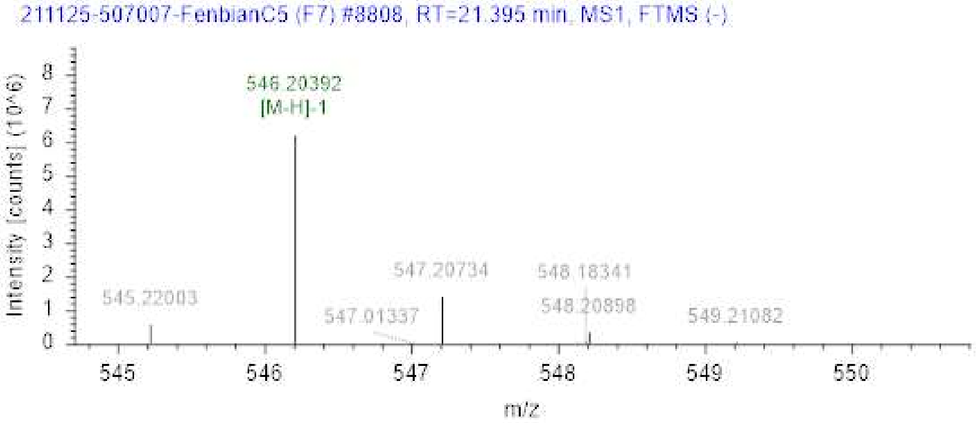

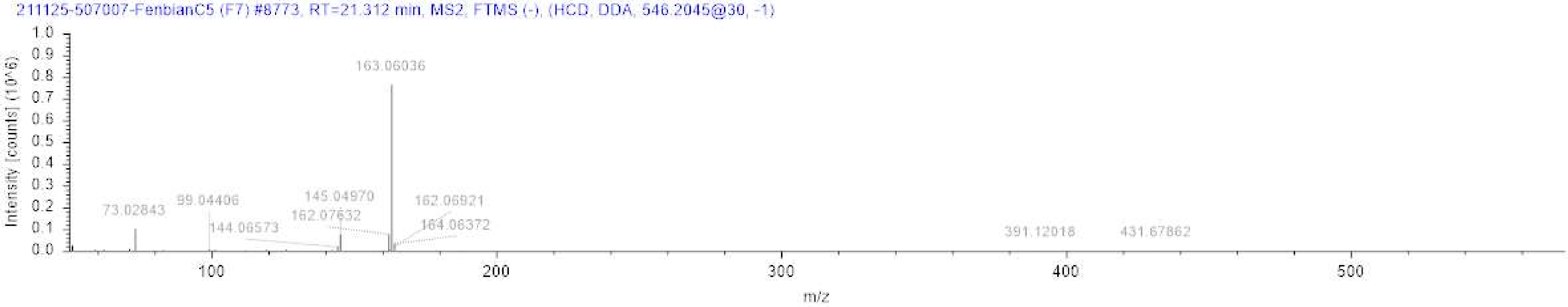

© Reported with Compound Discoverer 3.3

Structure	Name	RT [min]	Formula	Calc. MW	Areas
n/a		22.76	n/a	547.21120	5.86e7

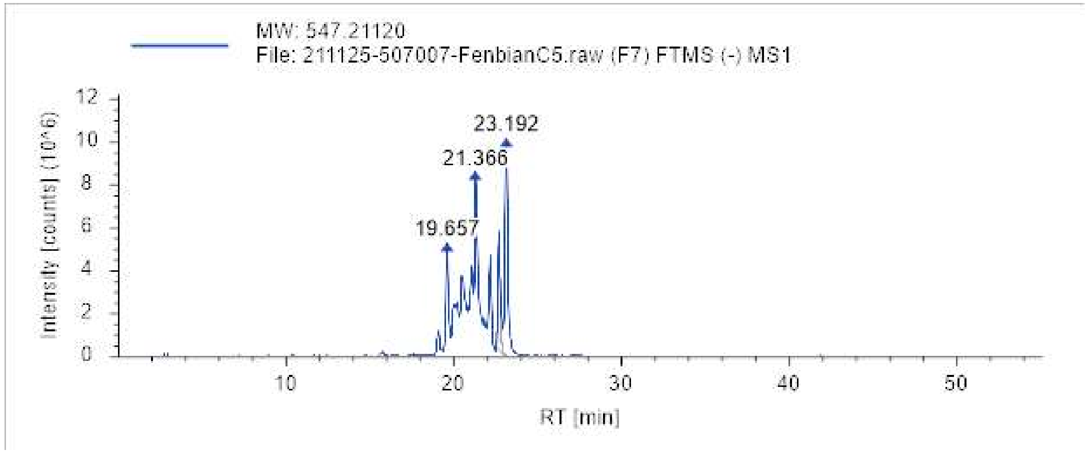

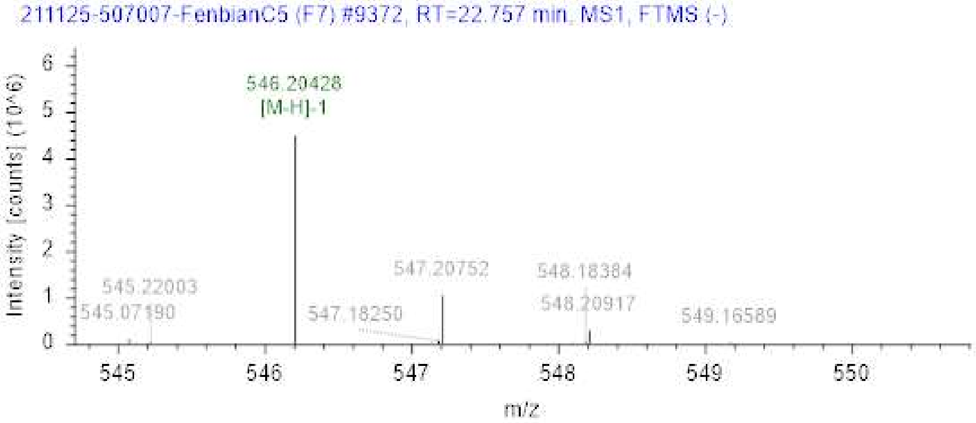

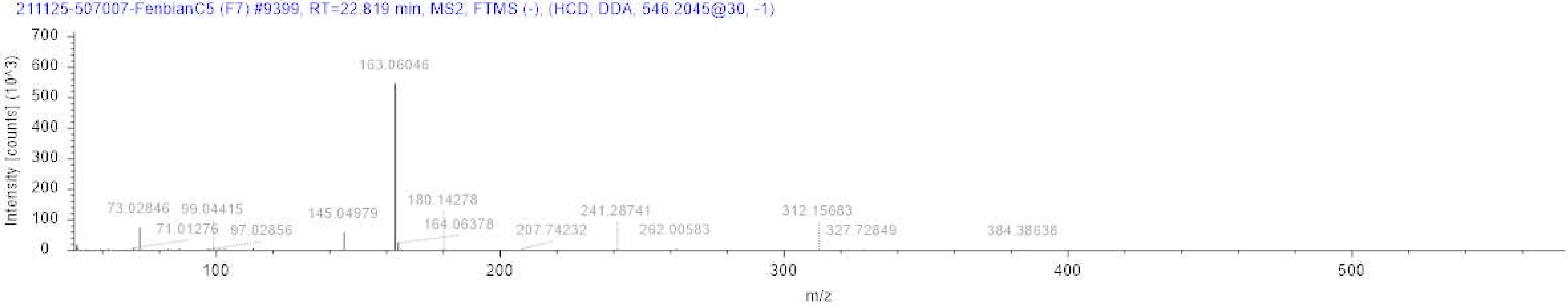

© Reported with Compound Discoverer 3.3

Compounds

Structure	Name	RT [min]	Formula	Calc. MW	Areas
n/a		19.65	n/a	547.21120	4.24e7

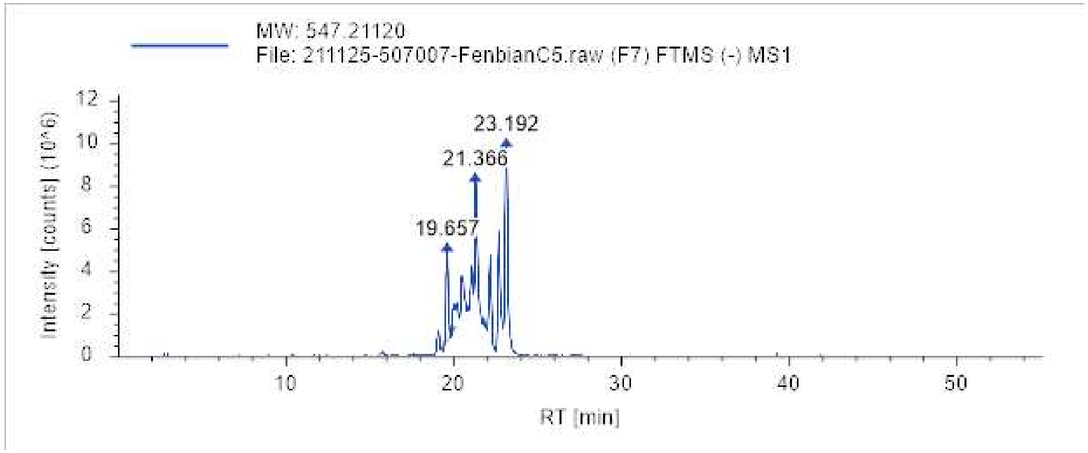

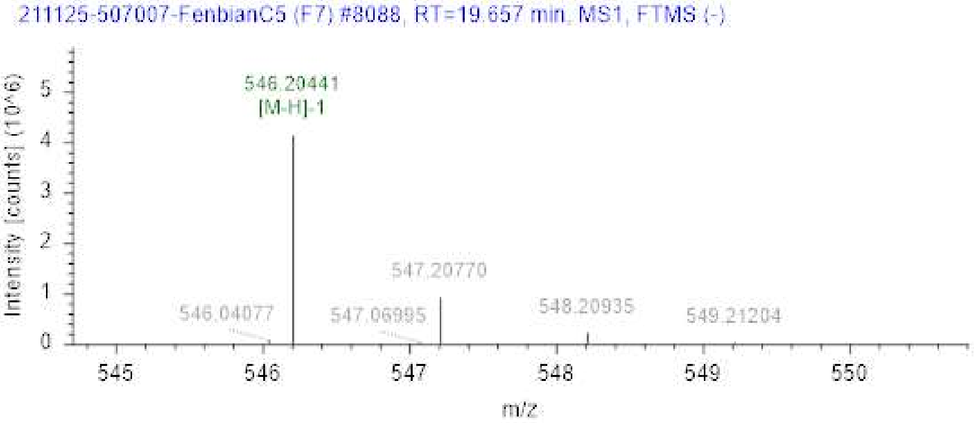

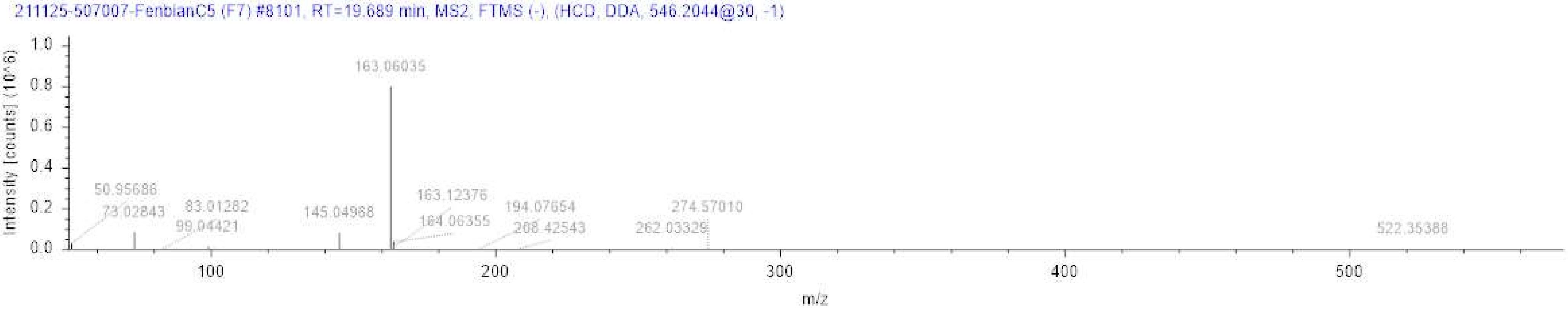

© Reported with Compound Discoverer 3.3

Compounds

Structure	Name	RT [min]	Formula	Calc. MW	Areas
n/a		23.20	n/a	563.20619	1.48e7

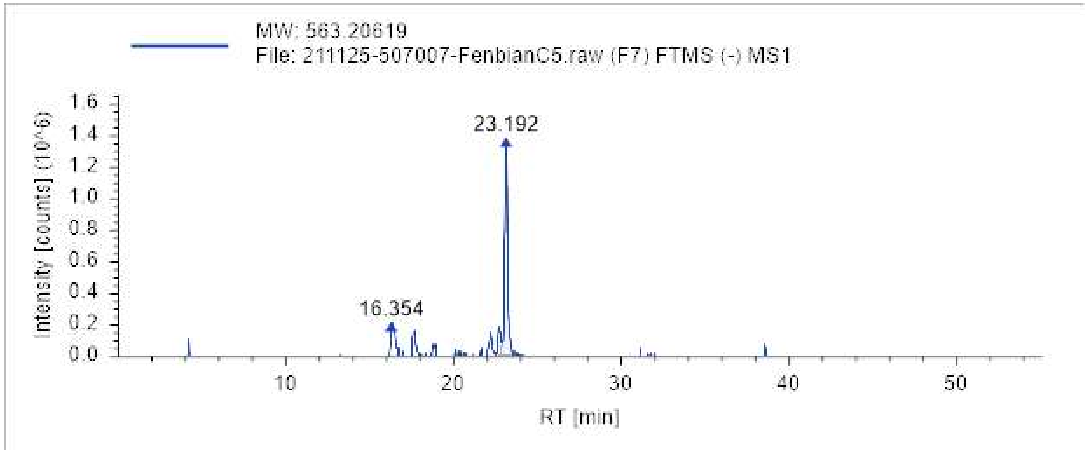

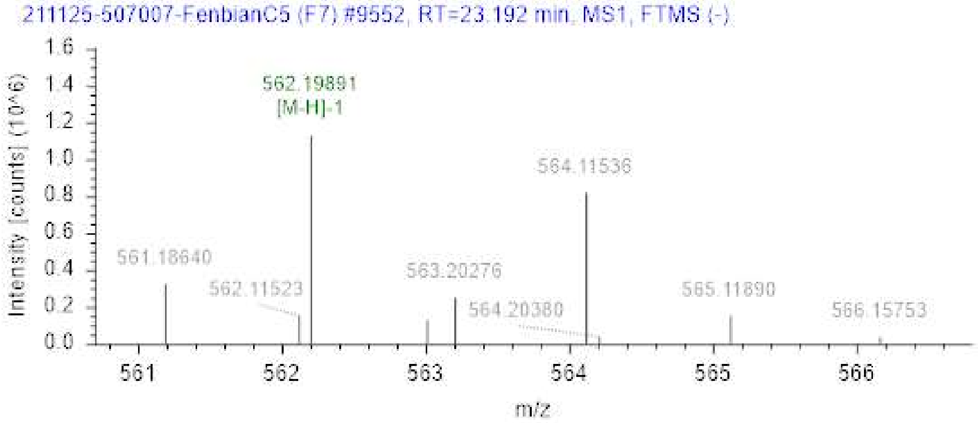

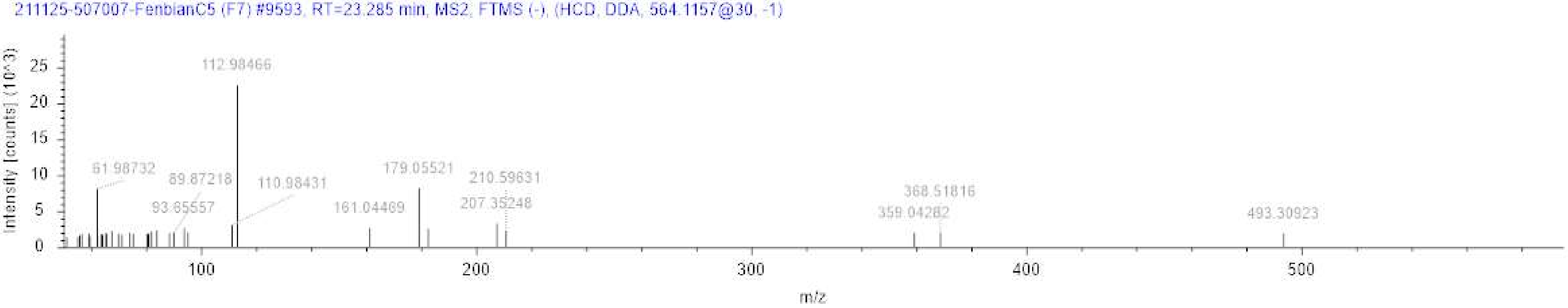

© Reported with Compound Discoverer 3.3

Structure	Name	RT [min]	Formula	Calc. MW	Areas
n/a		36.28	n/a	633.21150	4.40e9 7.63e9 2.63e9 4.35e9 5.78e10 4.14e10

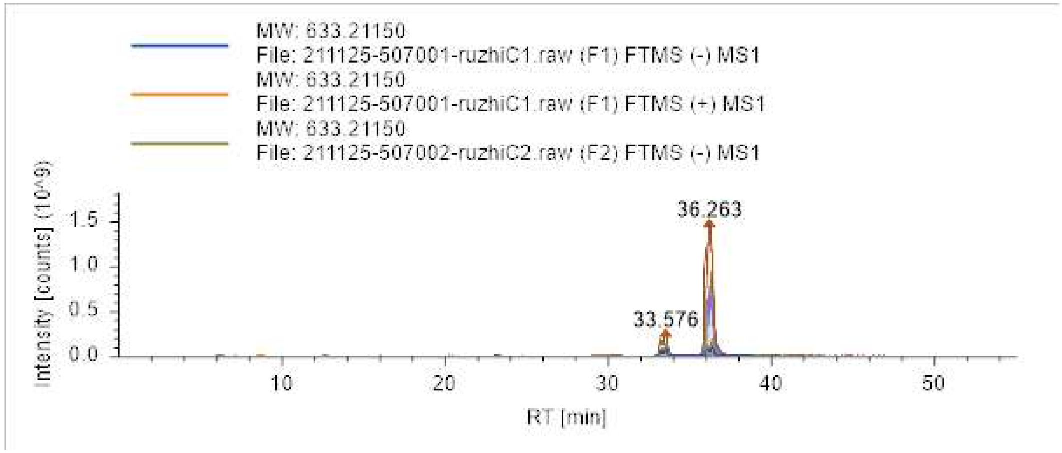

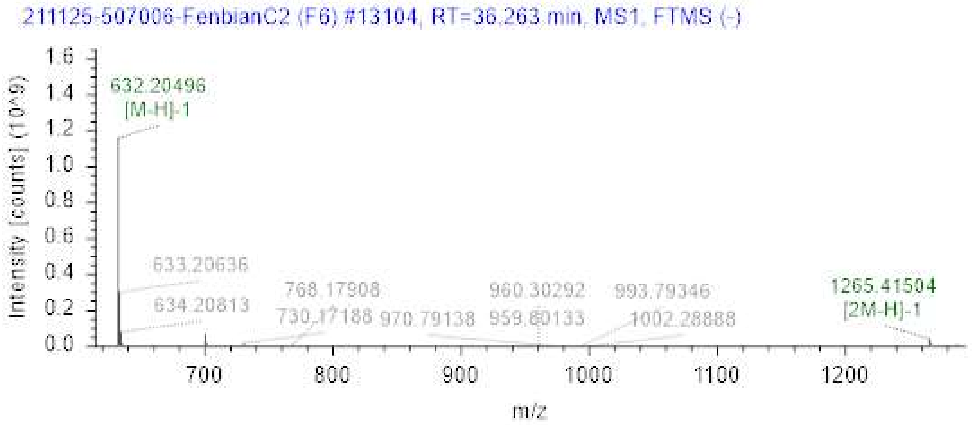

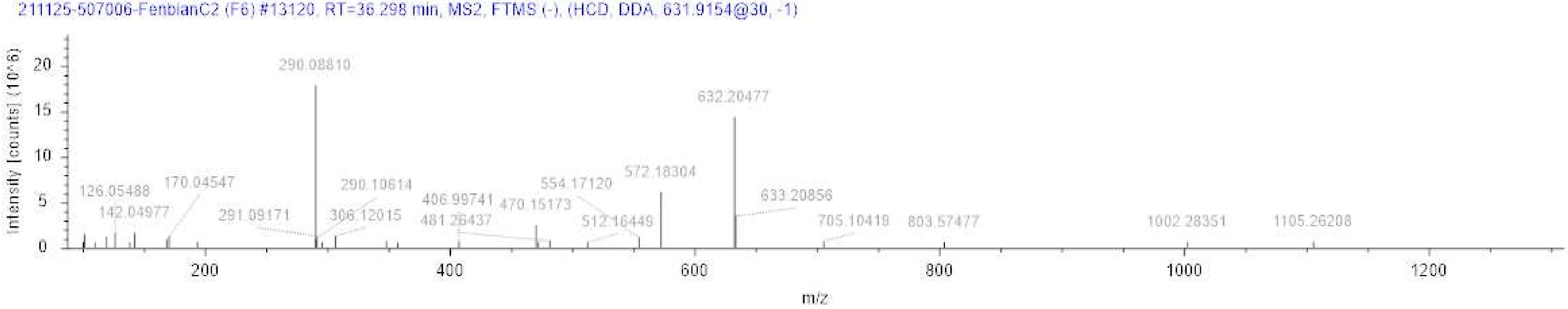

© Reported with Compound Discoverer 3.3

Compounds

Structure	Name	RT [min]	Formula	Calc. MW	Areas
n/a		39.27	n/a	633.21168	2.41e7

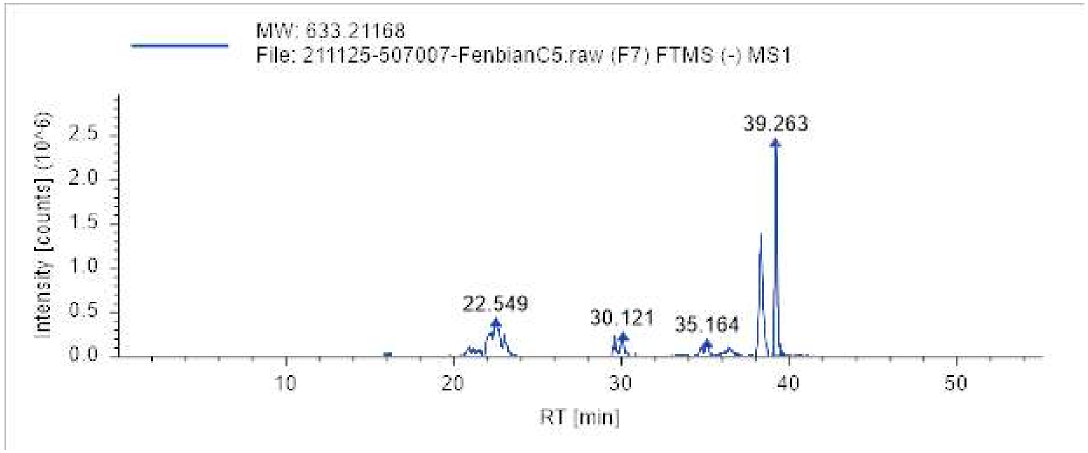

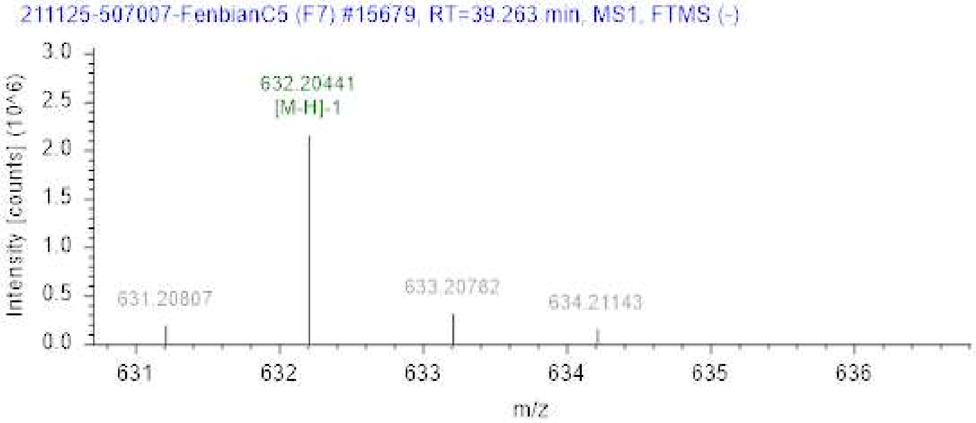

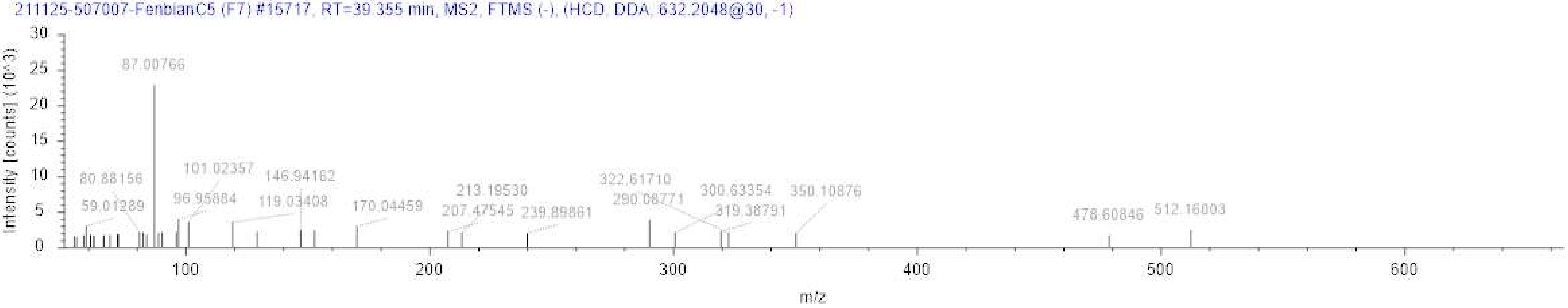

© Reported with Compound Discoverer 3.3

Compounds

Structure	Name	RT [min]	Formula	Calc. MW	Areas
n/a		38.37	n/a	633.21181	2.36e7

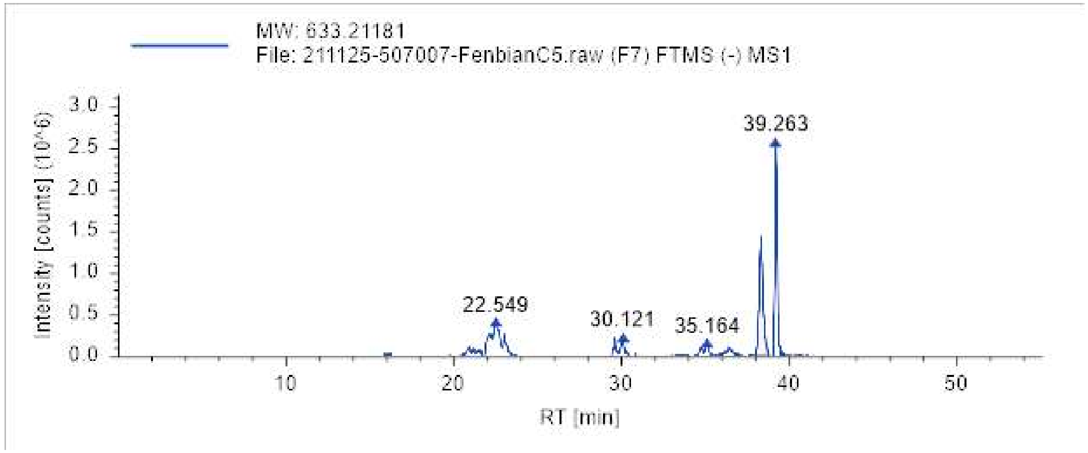

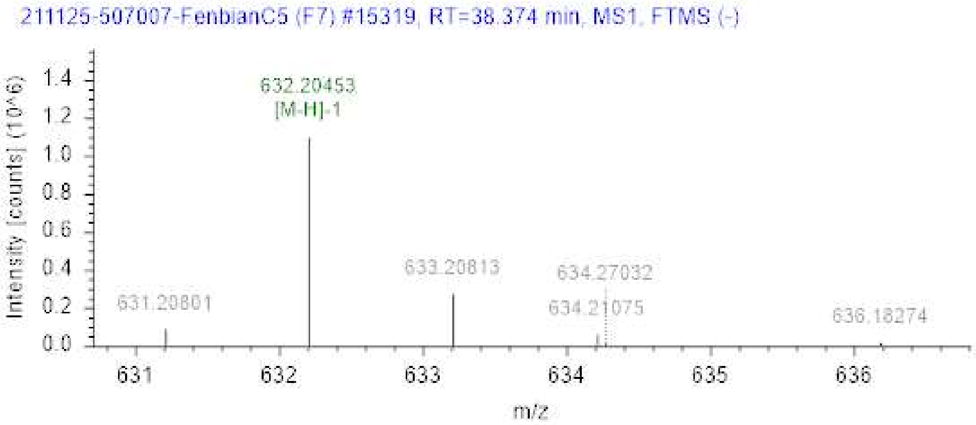

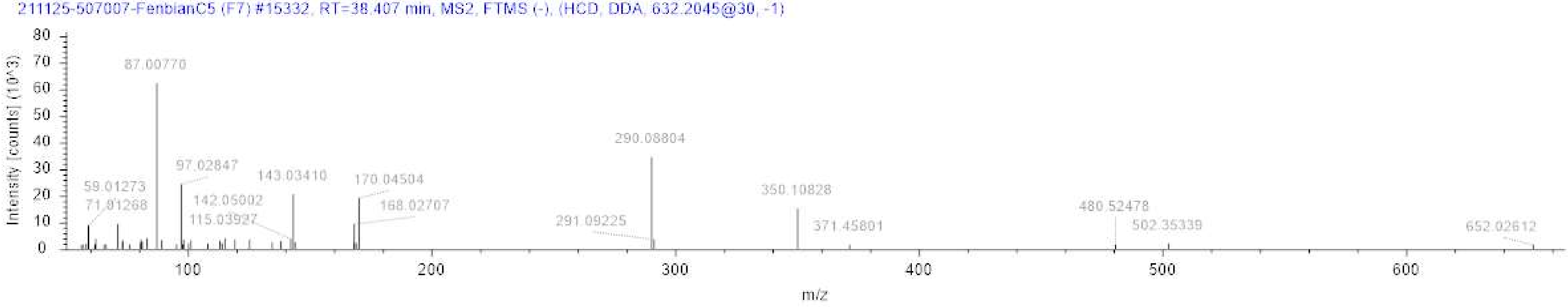

© Reported with Compound Discoverer 3.3

Compounds

Structure	Name	RT [min]	Formula	Calc. MW	Areas
n/a		33.56	n/a	633.21210	1.33e9 1.72e9 1.35e9 2.17e9 5.36e9 3.95e9

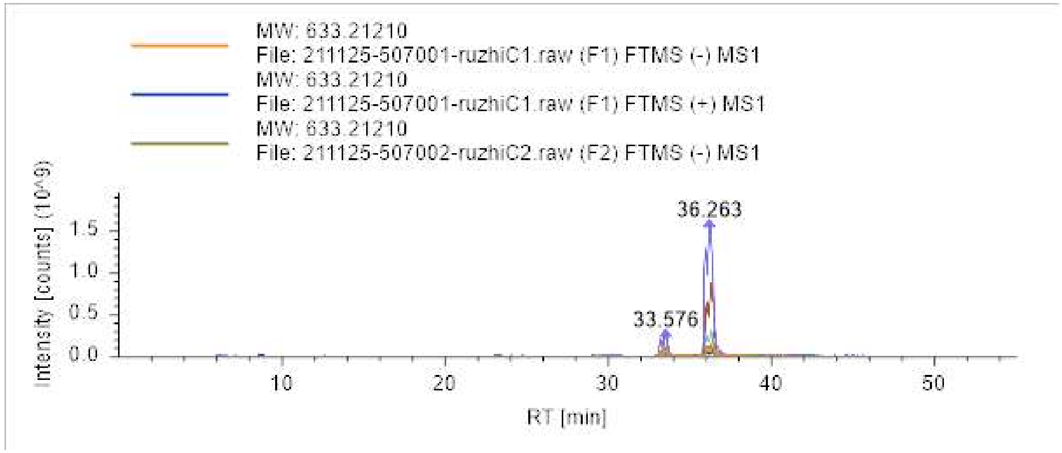

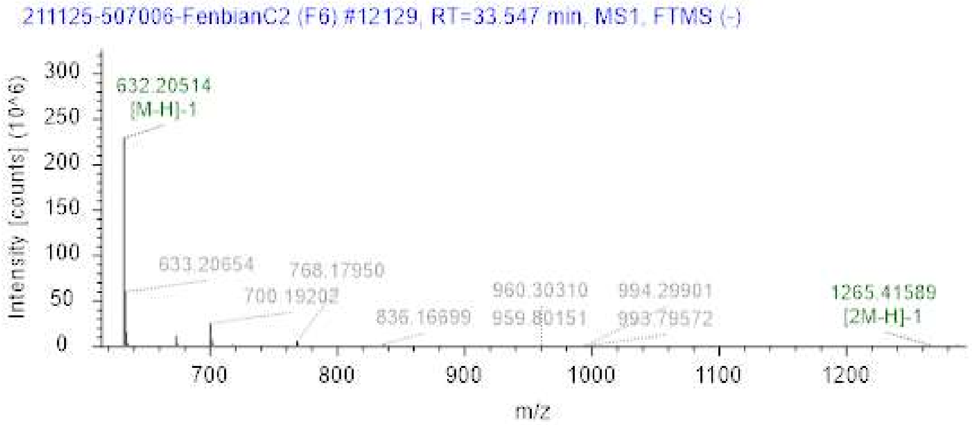

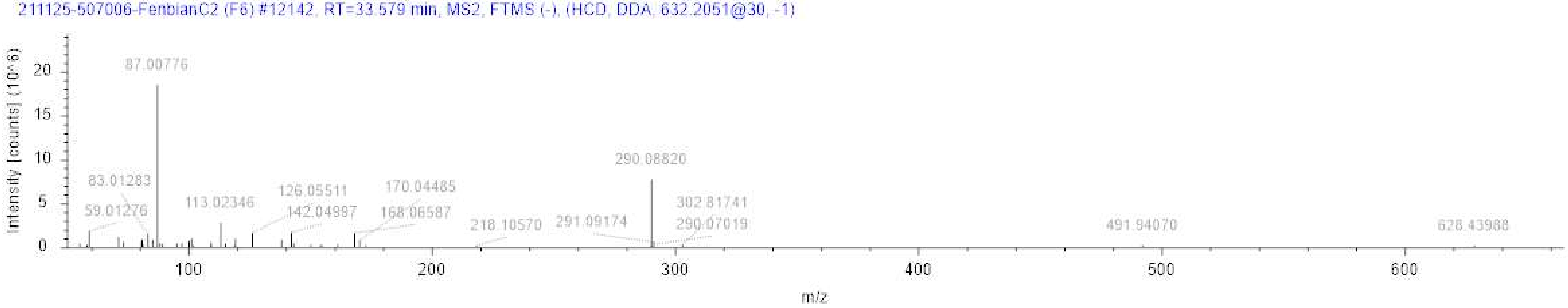

© Reported with Compound Discoverer 3.3

Structure	Name	RT [min]	Formula	Calc. MW	Areas
n/a		29.17	n/a	634.23004	2.05e7 4.27e8 1.36e8

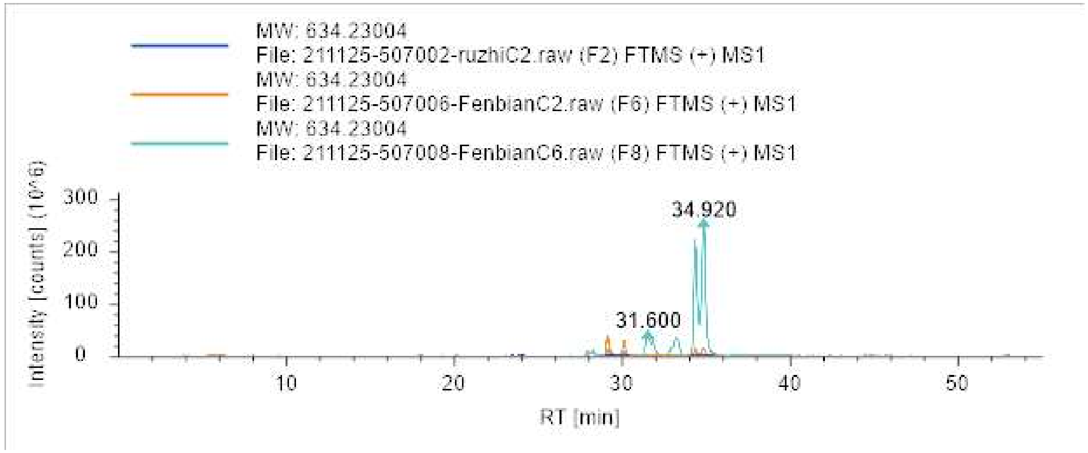

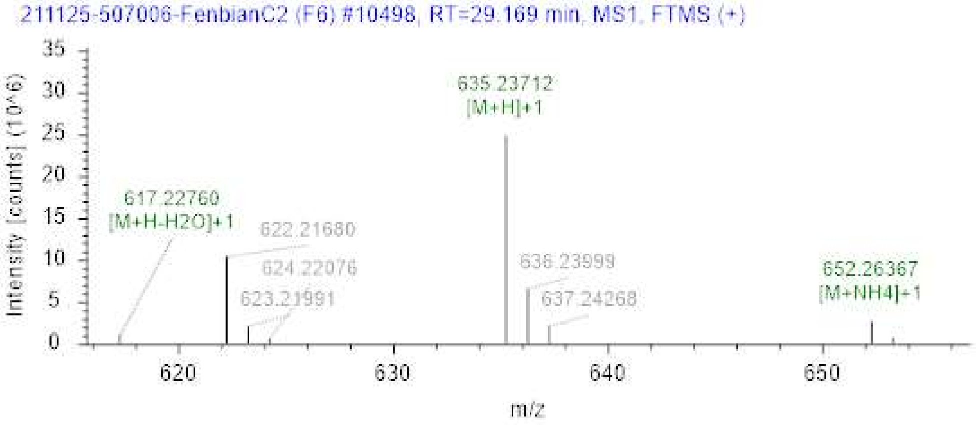

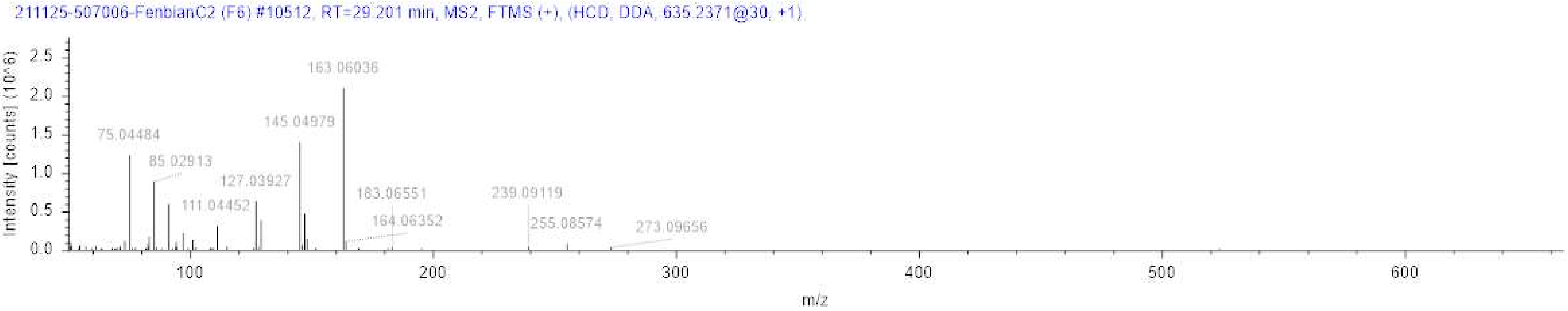

© Reported with Compound Discoverer 3.3

Structure	Name	RT [min]	Formula	Calc. MW	Areas
n/a		34.39	n/a	634.23043	2.16e9 5.78e9 4.36e9 4.36e8 4.11e9

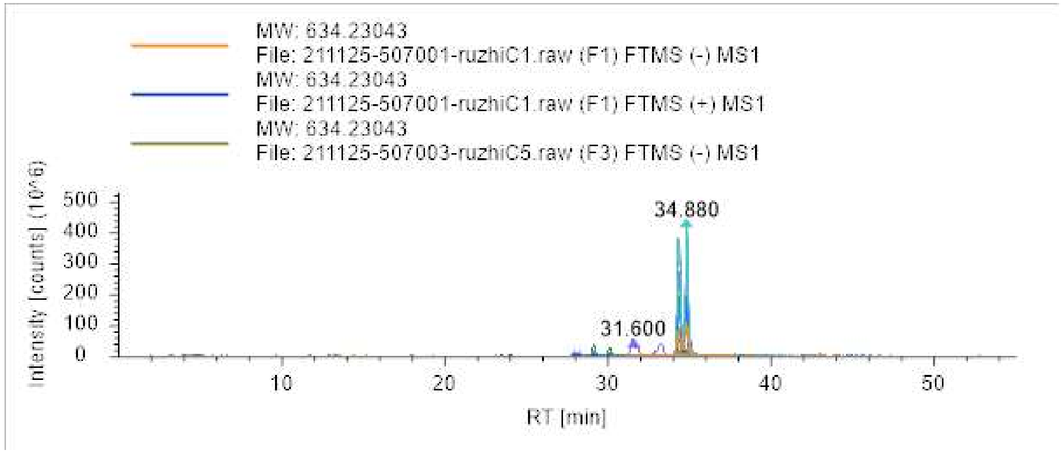

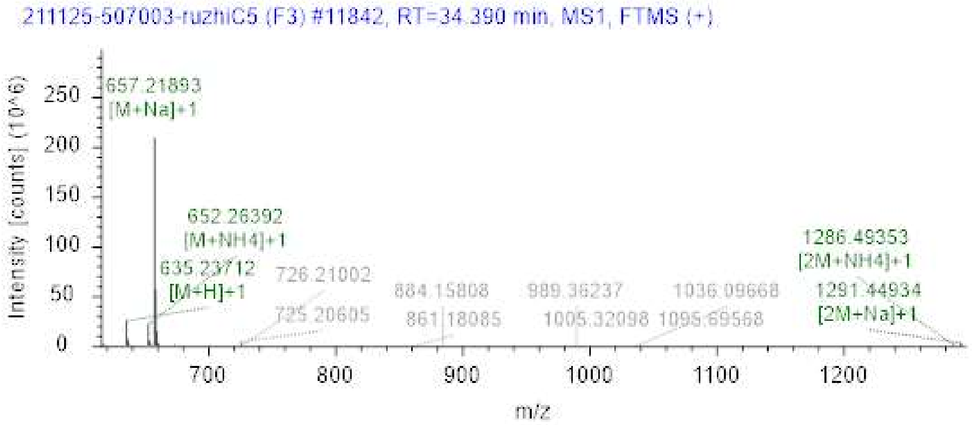

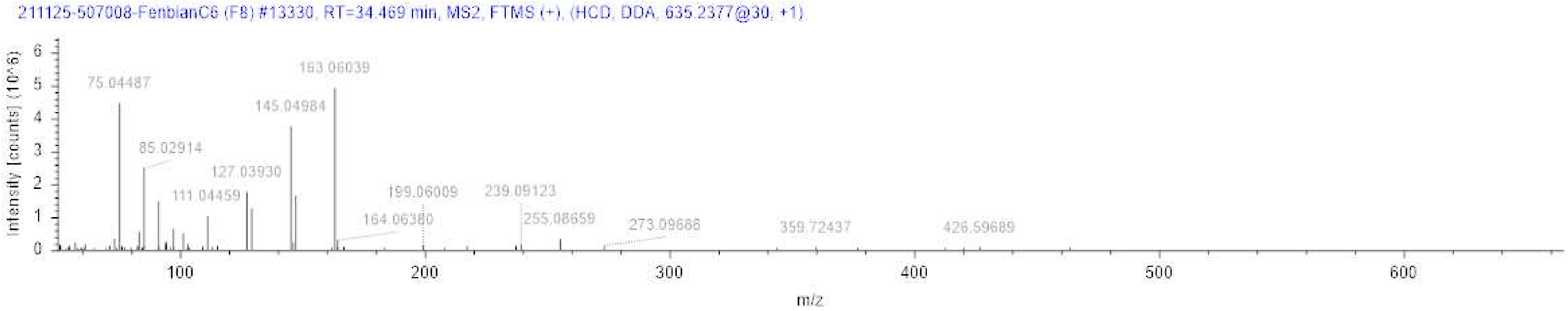

© Reported with Compound Discoverer 3.3

Structure	Name	RT [min]	Formula	Calc. MW	Areas
n/a		31.53	n/a	634.23119	1.45e7, 1.37e7, 1.68e7, 3.64e7, 4.93e9

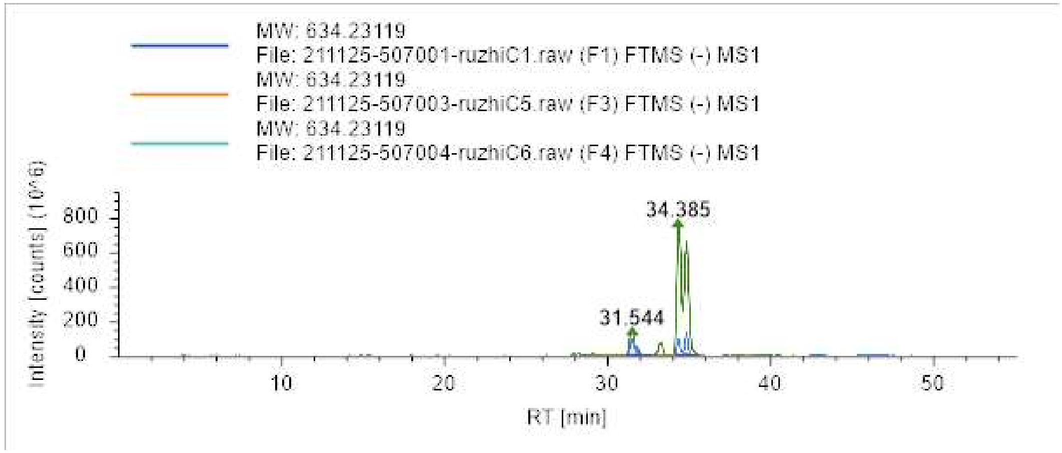

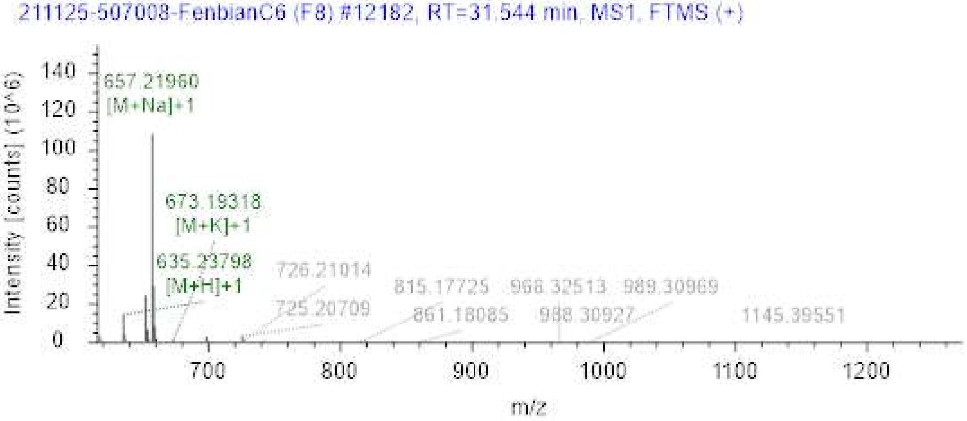

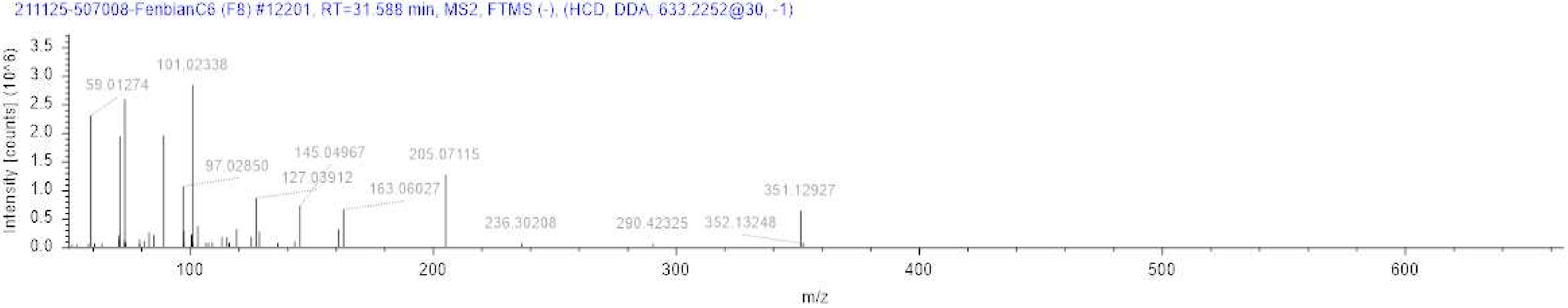

© Reported with Compound Discoverer 3.3

Compounds

Structure	Name	RT [min]	Formula	Calc. MW	Areas
n/a		34.89	n/a	634.23237	2.81e9 8.12e9 4.85e9 8.06e8 2.81e10

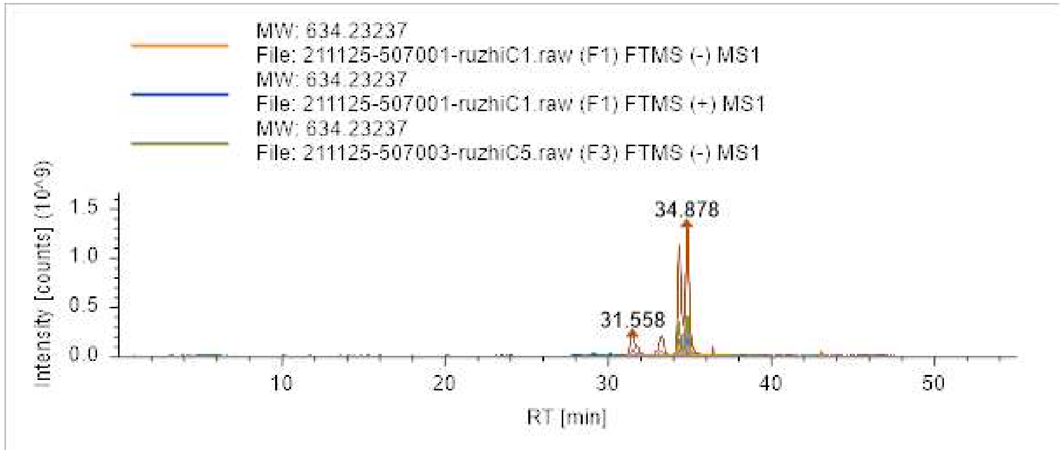

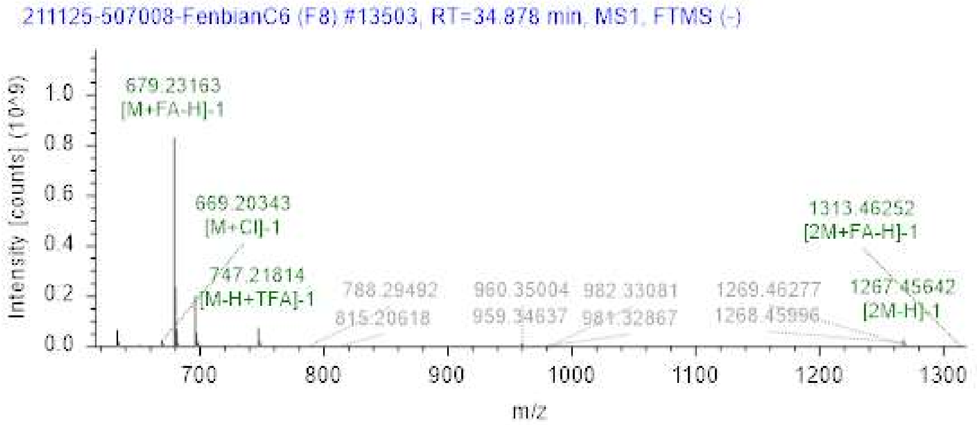

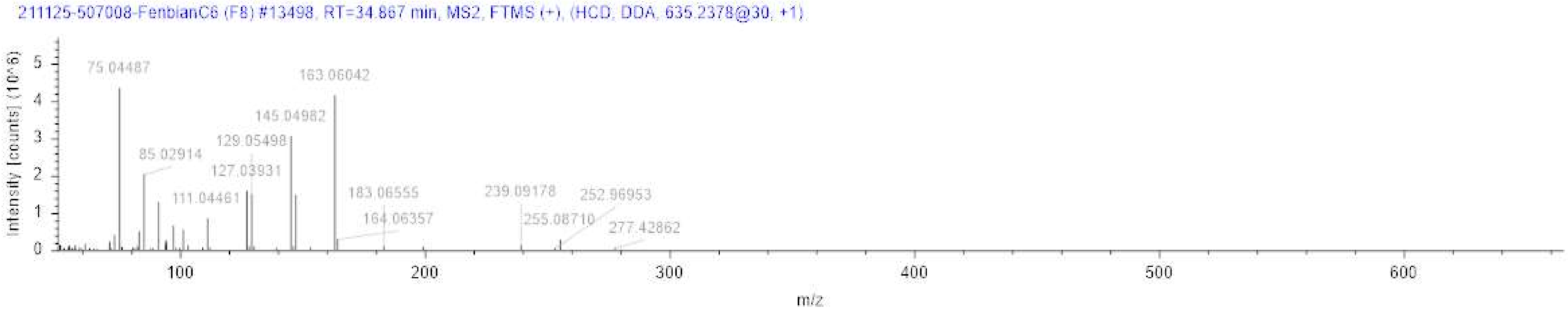

© Reported with Compound Discoverer 3.3

Structure	Name	RT [min]	Formula	Calc. MW	Areas
n/a		32.98	n/a	634.23248	1.56e7 4.78e7

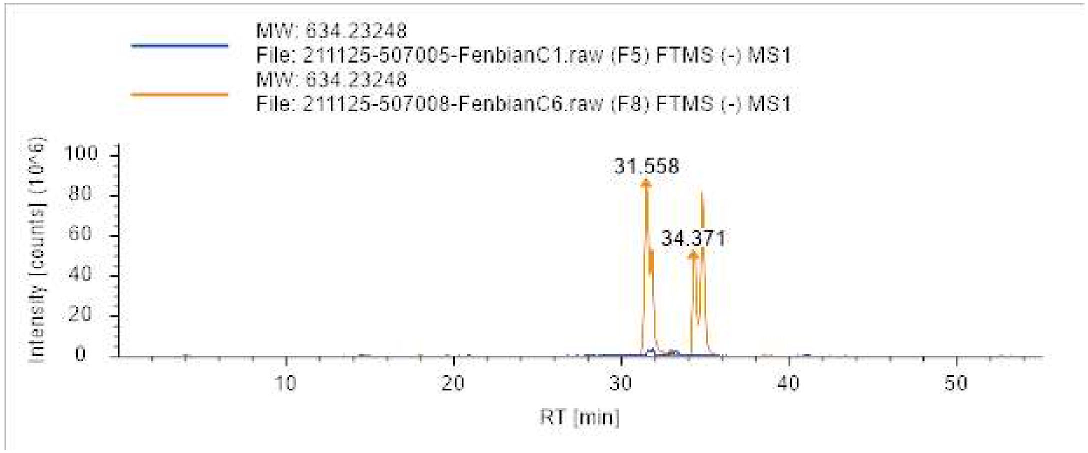

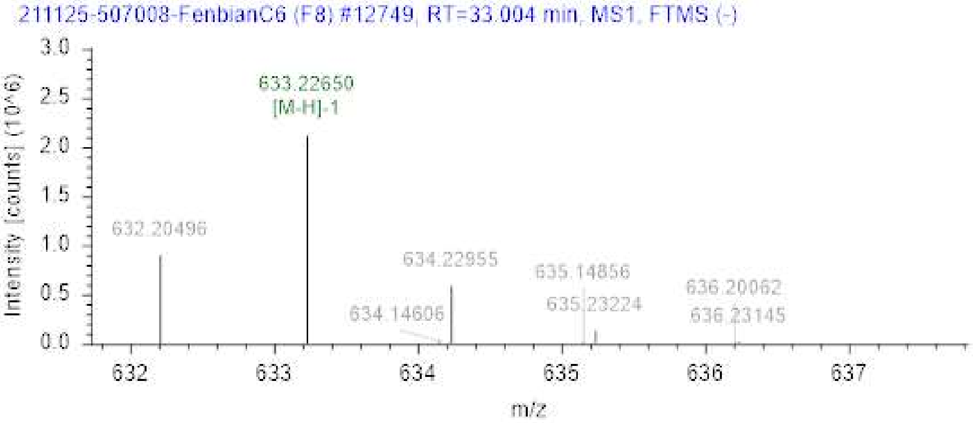

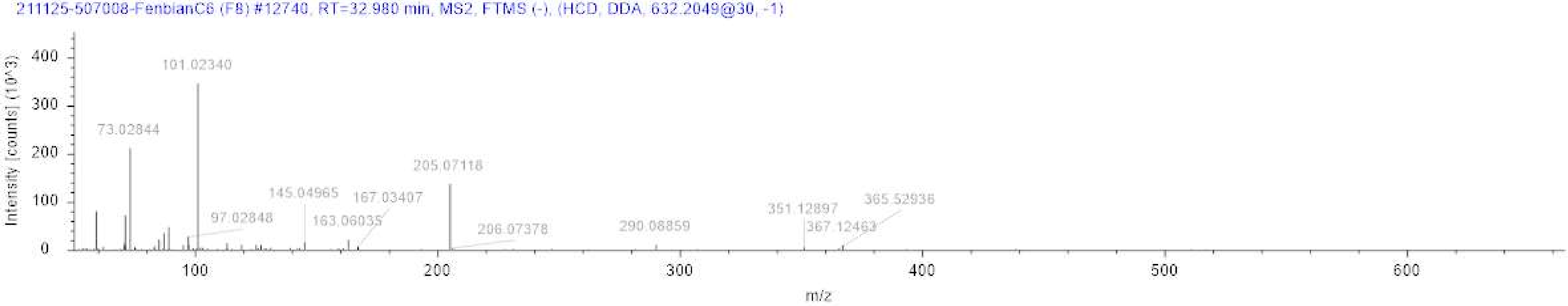

© Reported with Compound Discoverer 3.3

Structure	Name	RT [min]	Formula	Calc. MW	Areas
n/a		40.66	n/a	650.22516	1.82e7

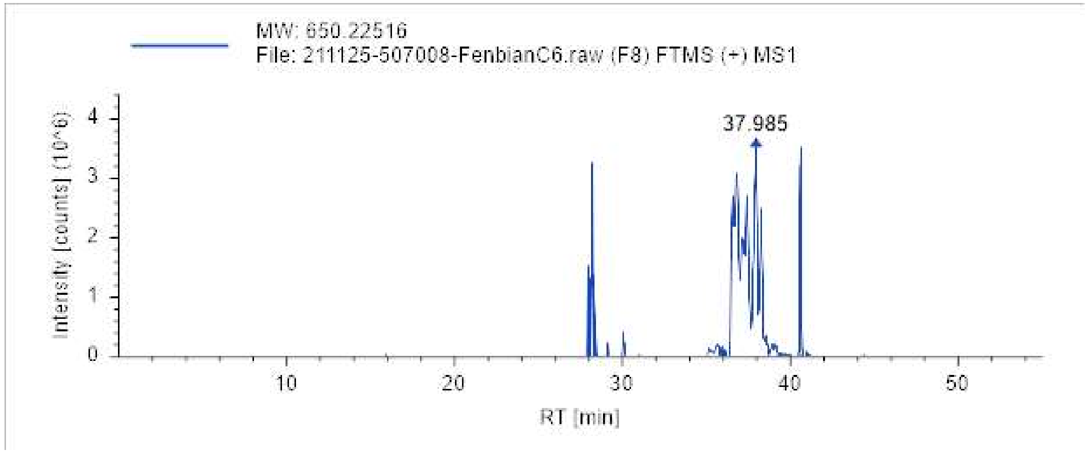

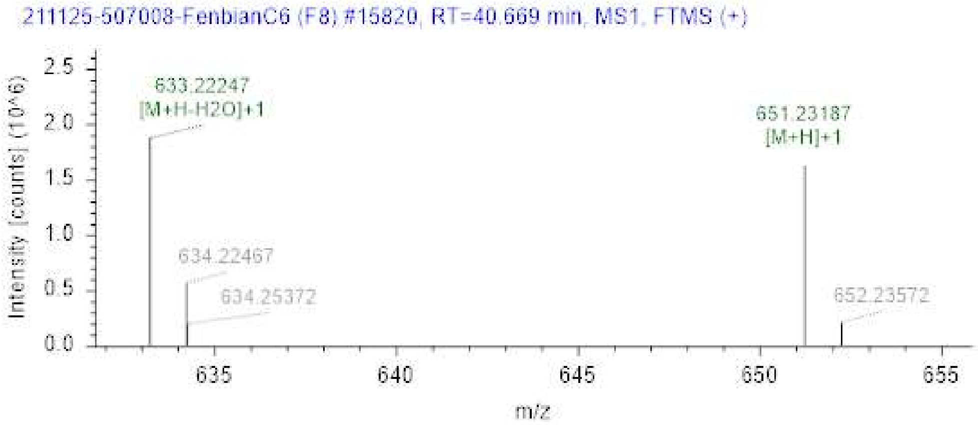

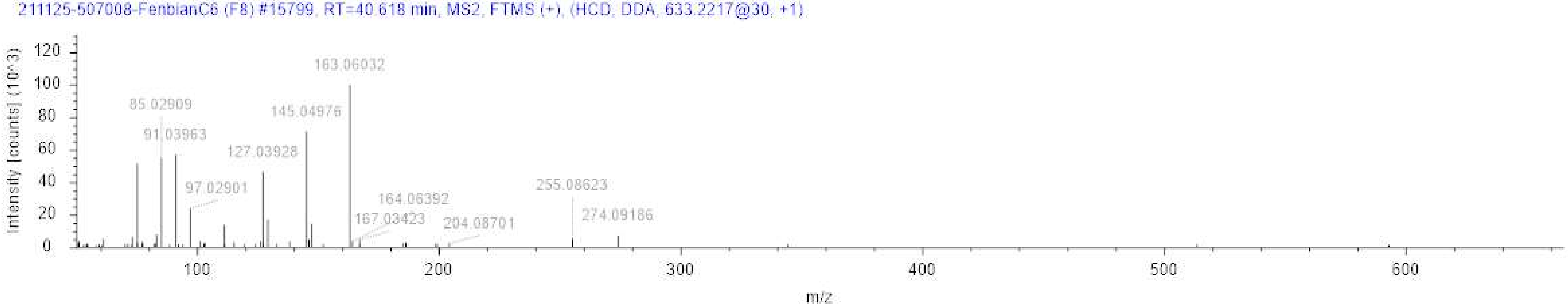

© Reported with Compound Discoverer 3.3

Compounds

Structure	Name	RT [min]	Formula	Calc. MW	Areas
n/a		37.99	n/a	650.22661	3.20e7 6.52e8 1.33e7

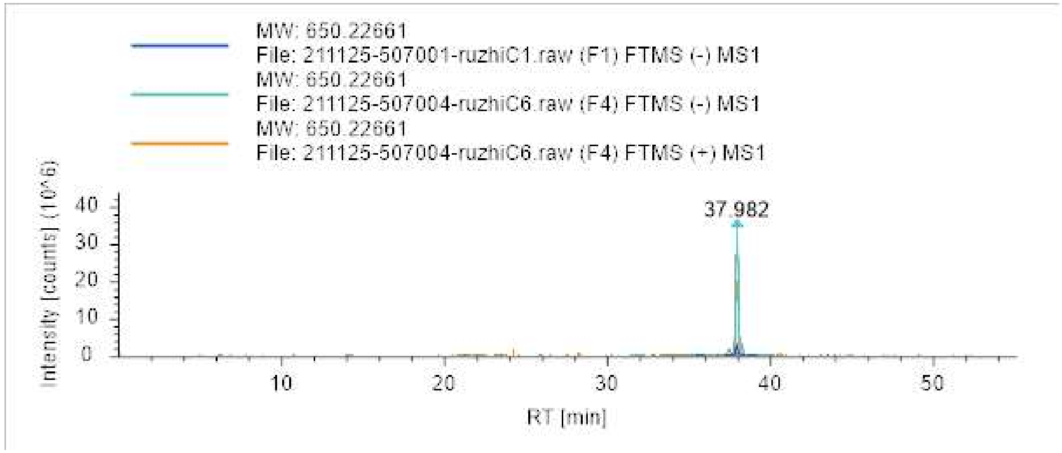

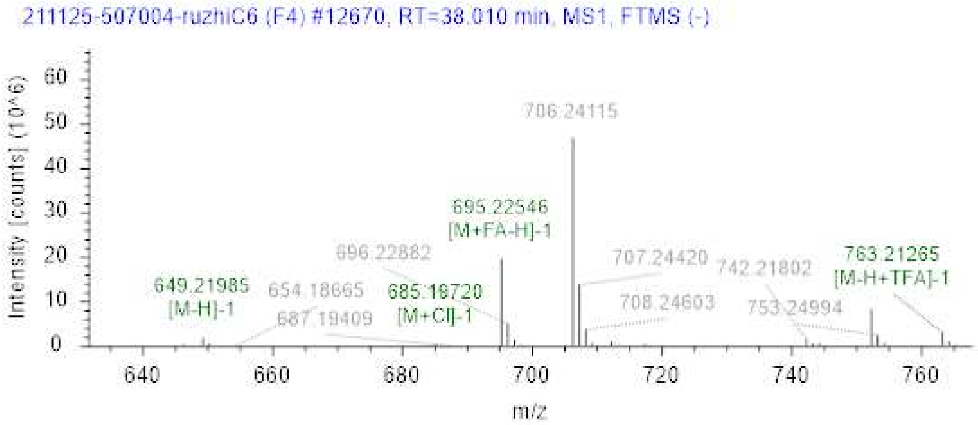

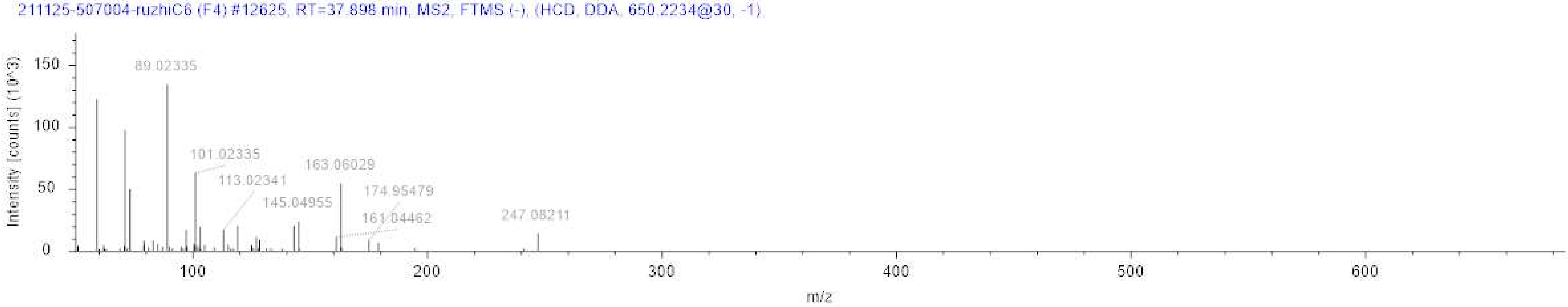

© Reported with Compound Discoverer 3.3

Structure	Name	RT [min]	Formula	Calc. MW	Areas
n/a		39.14	n/a	650.22798	1.83e7; 5.15e7

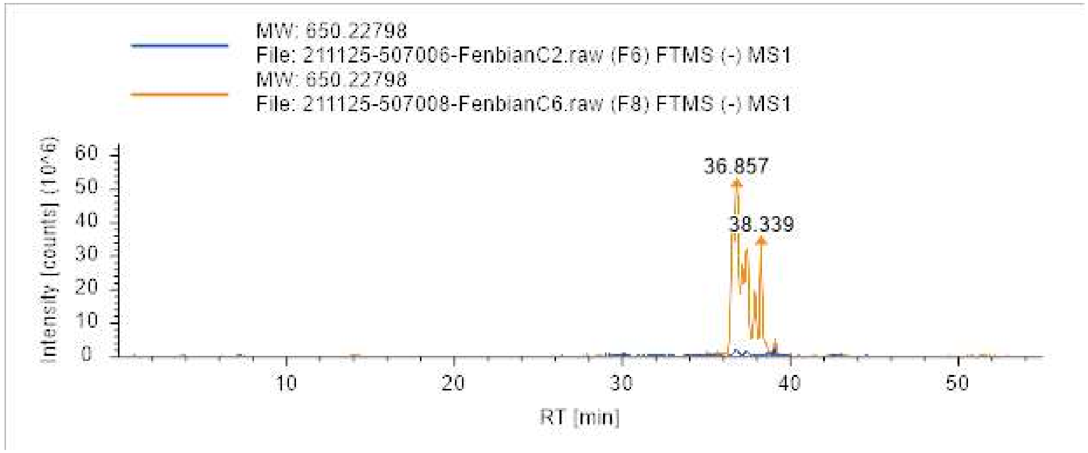

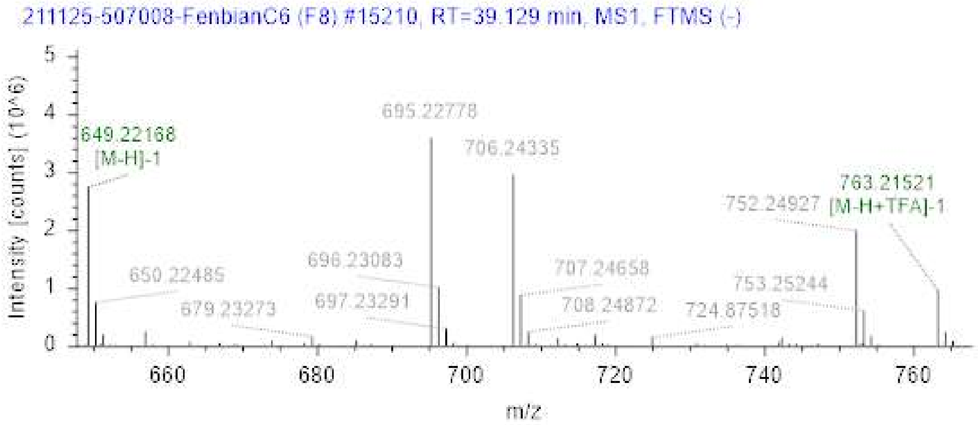

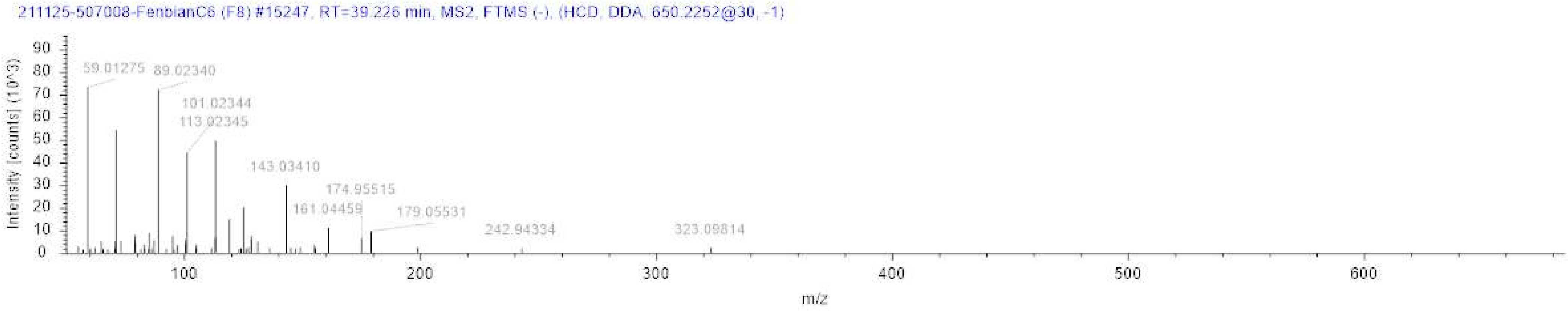

© Reported with Compound Discoverer 3.3

Structure	Name	RT [min]	Formula	Calc. MW	Areas
n/a		36.83	n/a	650.22819	3.76e7 8.95e8

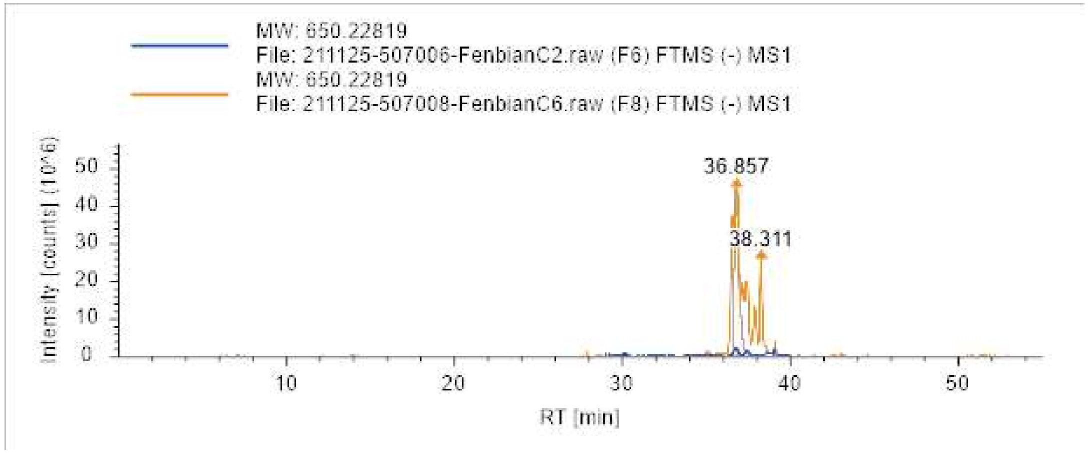

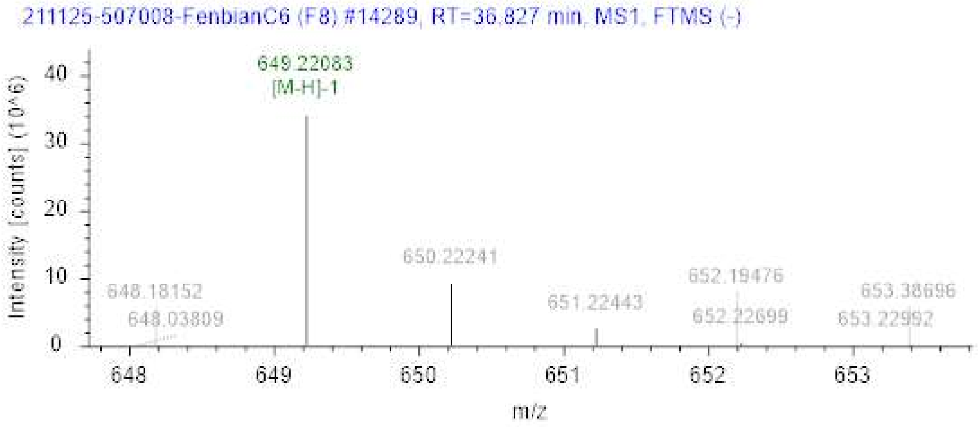

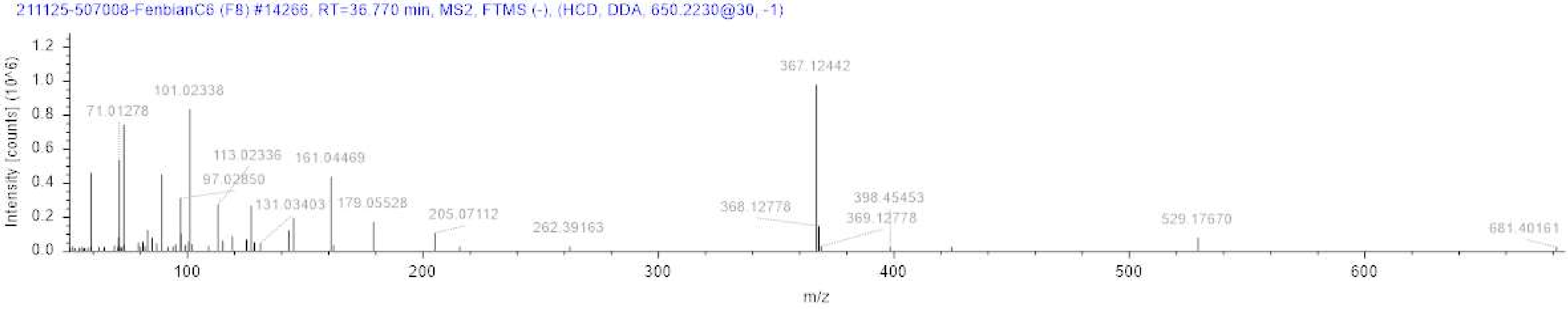

© Reported with Compound Discoverer 3.3

Compounds

Structure	Name	RT [min]	Formula	Calc. MW	Areas
n/a		13.61	n/a	651.22118	6.58e6

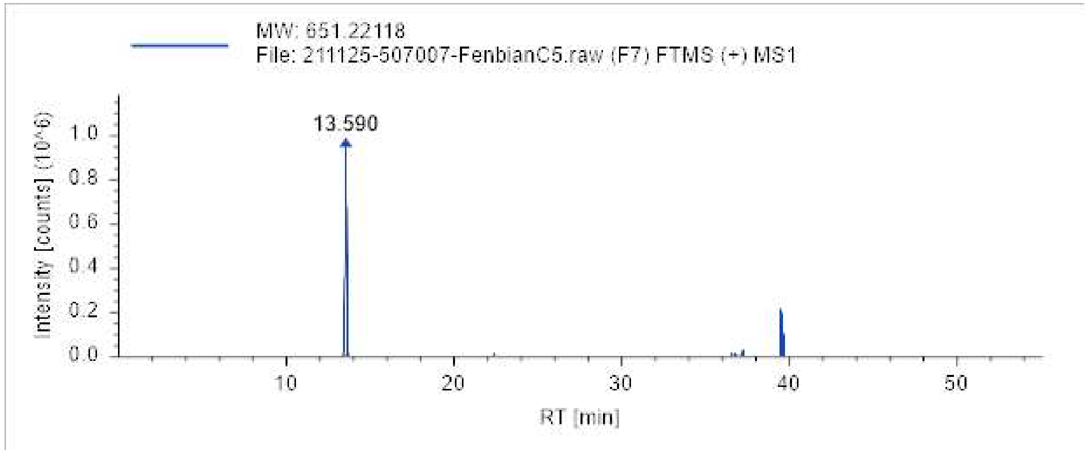

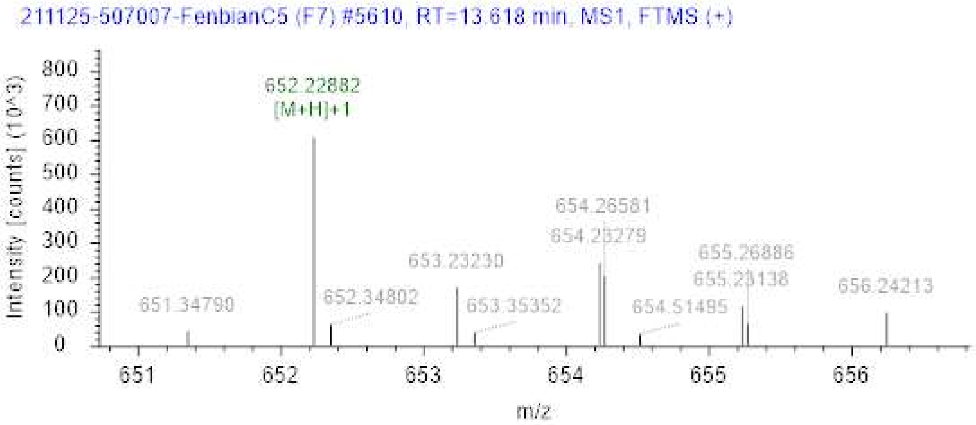

© Reported with Compound Discoverer 3.3

Compounds

Structure	Name	RT [min]	Formula	Calc. MW	Areas
n/a		24.14	n/a	666.21919	5.91e9 3.36e9 3.61e9

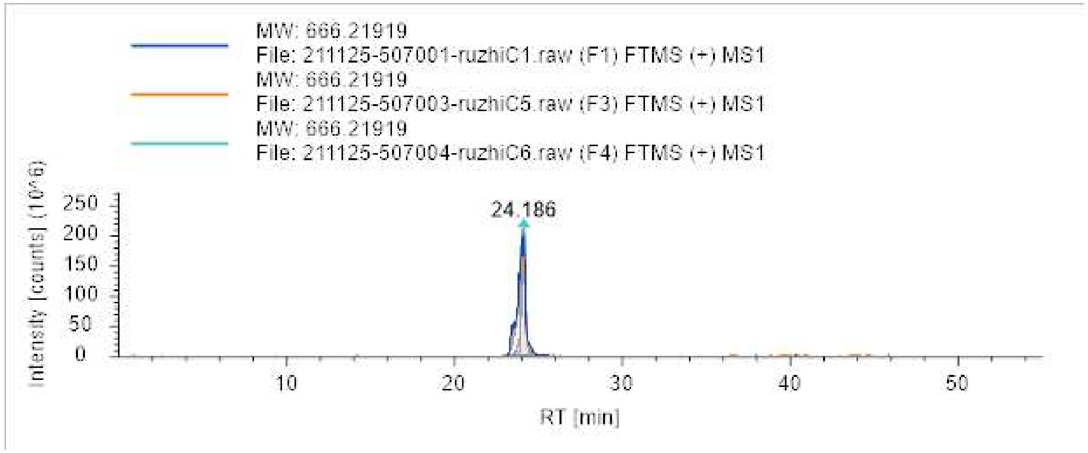

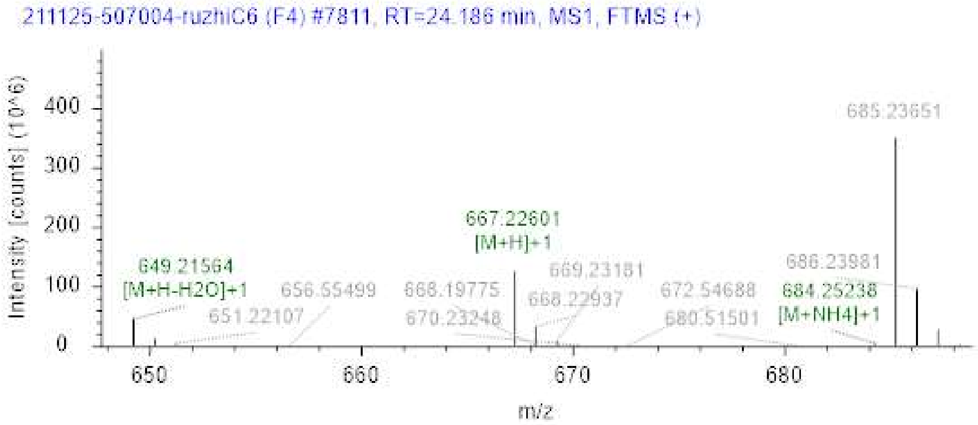

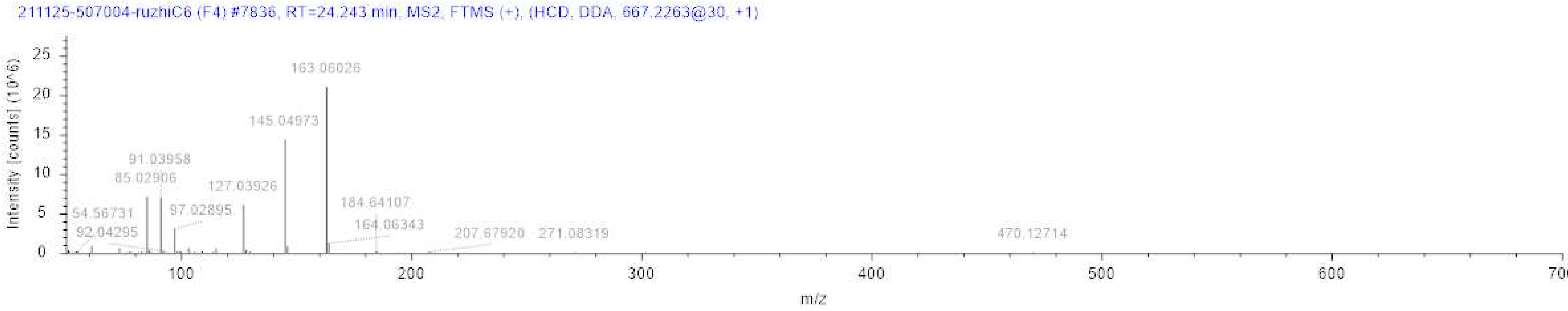

© Reported with Compound Discoverer 3.3

Compounds

Structure	Name	RT [min]	Formula	Calc. MW	Areas
n/a		40.36	n/a	666.22216	1.64e7

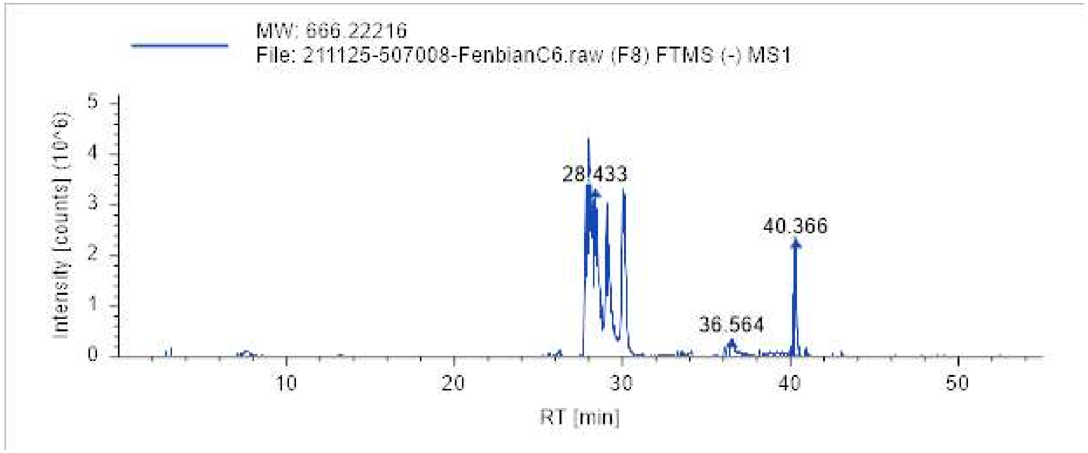

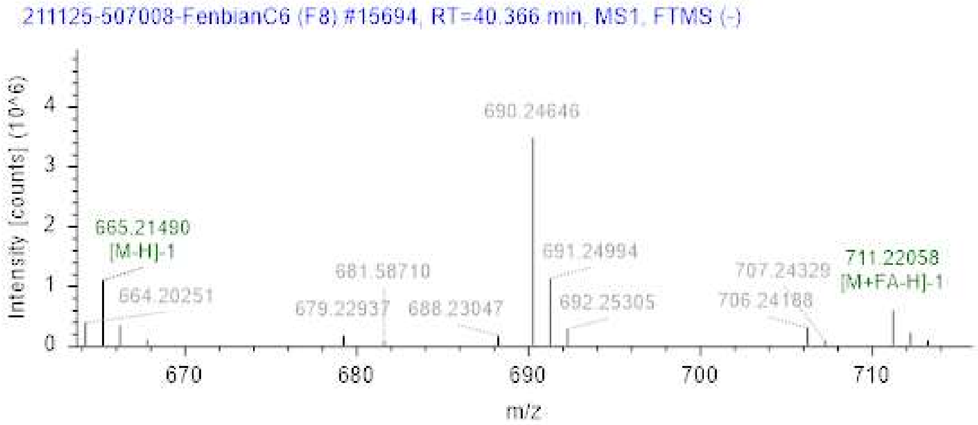

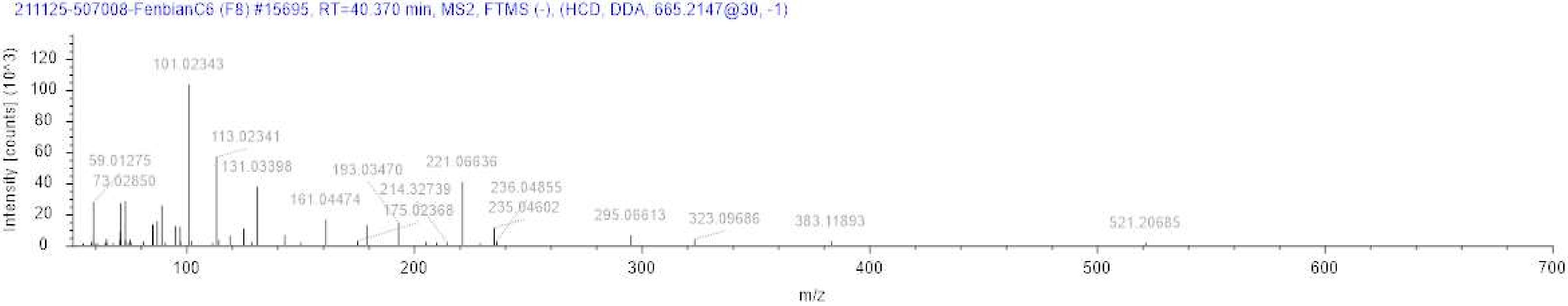

© Reported with Compound Discoverer 3.3

Structure	Name	RT [min]	Formula	Calc. MW	Areas
n/a		39.38	n/a	666.22274	5.39e7

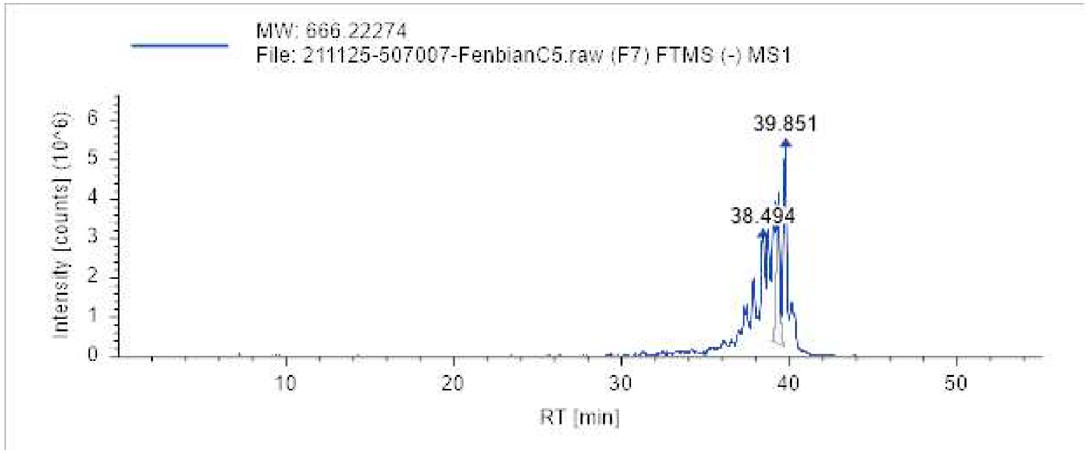

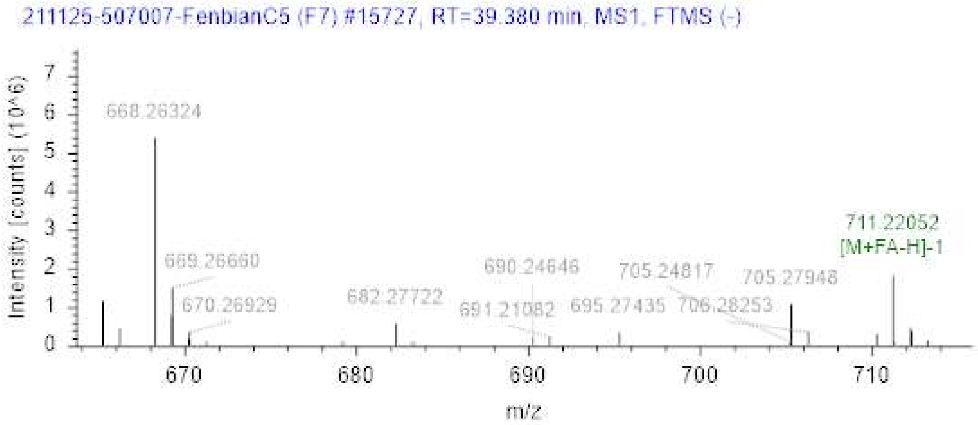

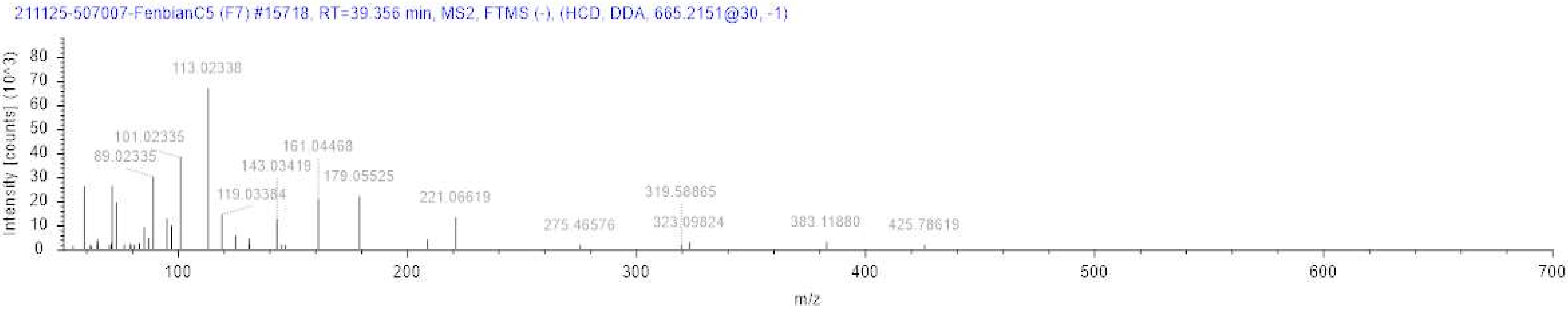

© Reported with Compound Discoverer 3.3

Structure	Name	RT [min]	Formula	Calc. MW	Areas
n/a		23.06	n/a	668.23387	5.11e6

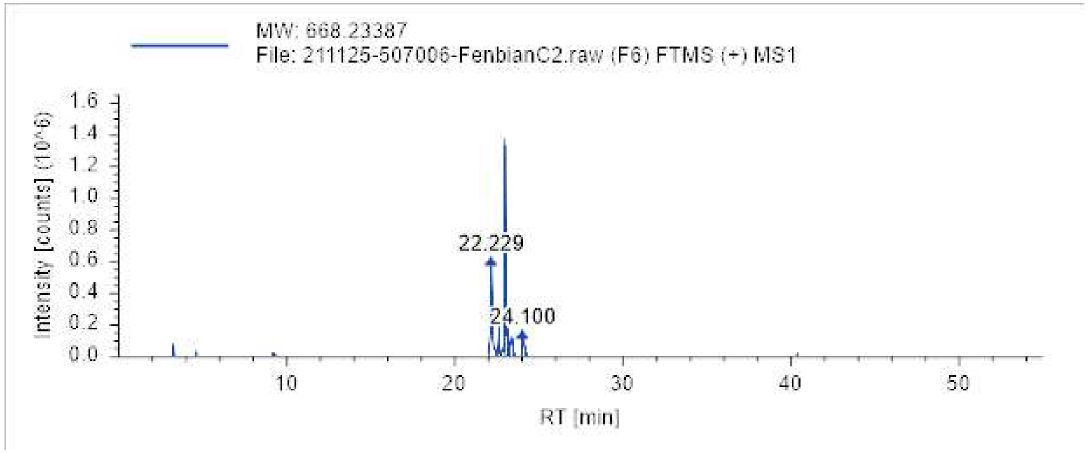

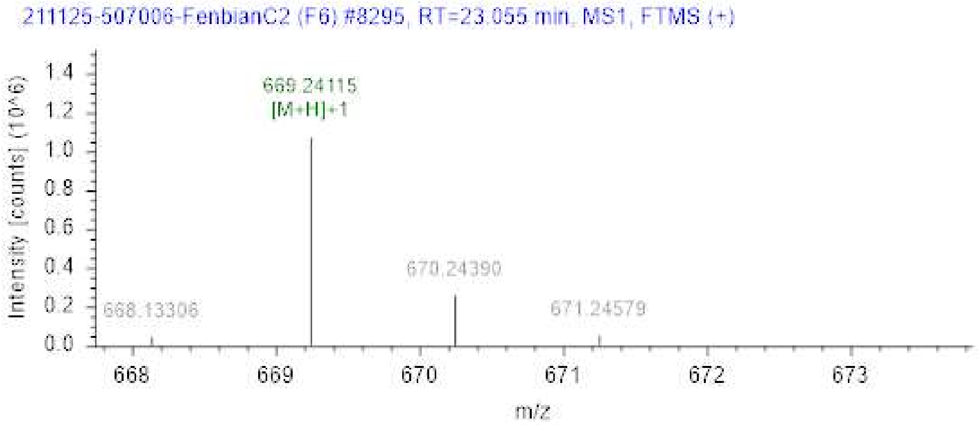

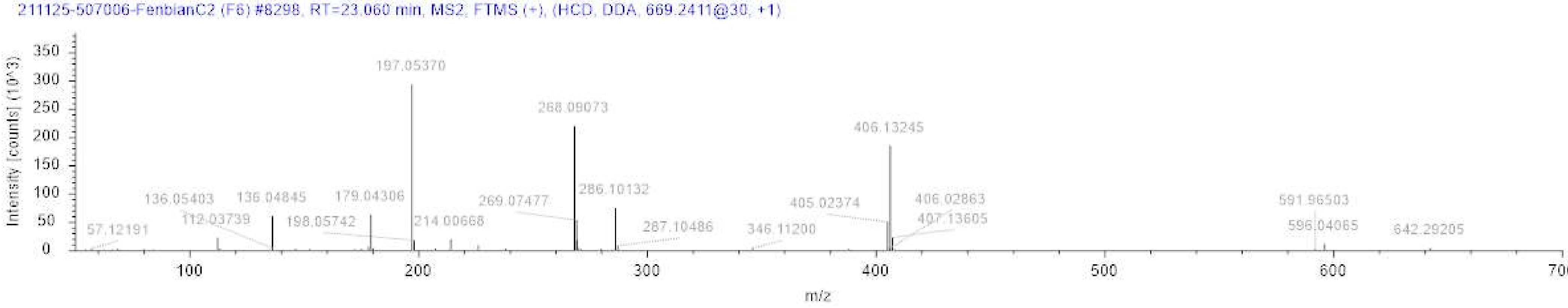

© Reported with Compound Discoverer 3.3

Structure	Name	RT [min]	Formula	Calc. MW	Areas
n/a		24.11	n/a	668.23418	1.33e7

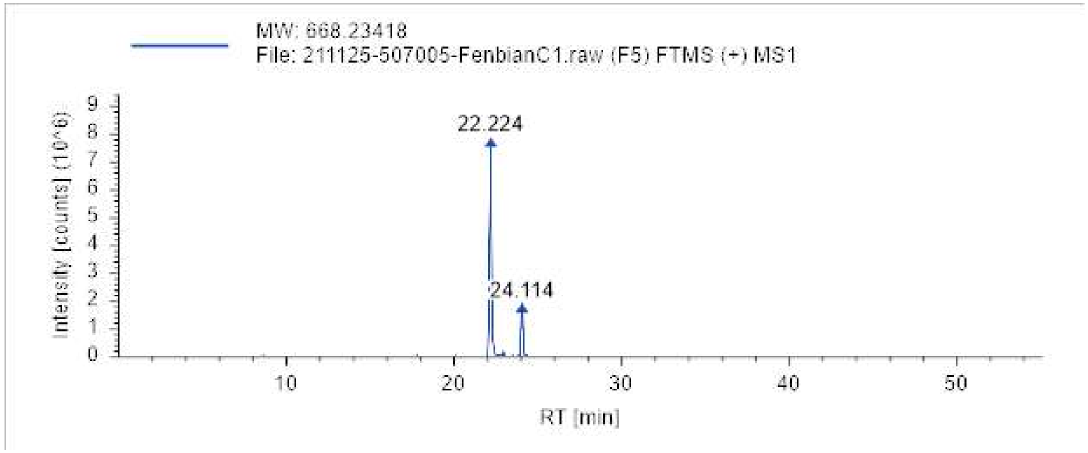

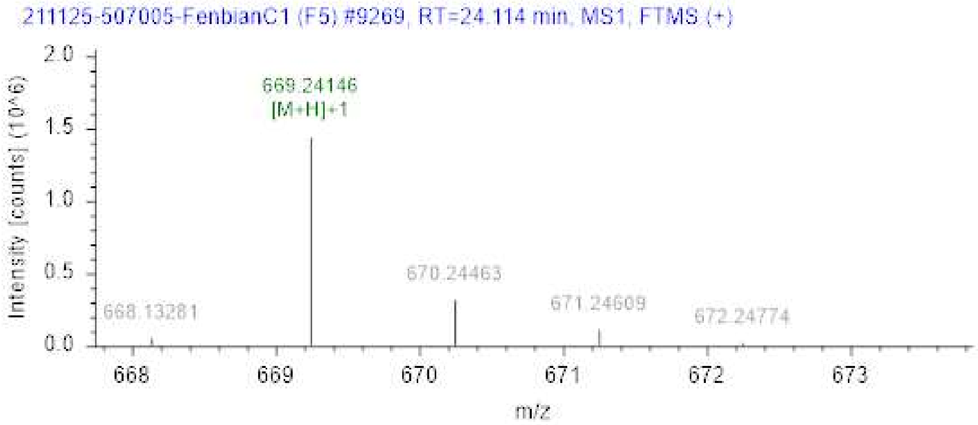

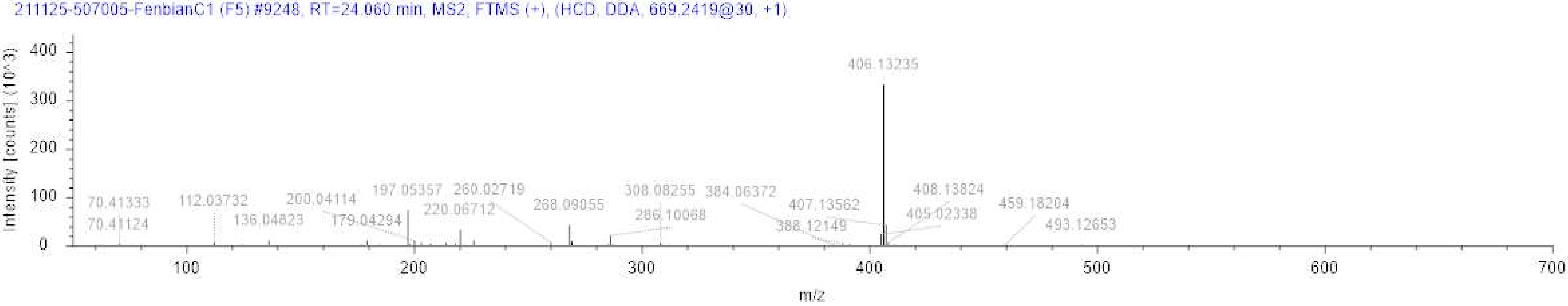

© Reported with Compound Discoverer 3.3

Compounds

Structure	Name	RT [min]	Formula	Calc. MW	Areas
n/a		22.23	n/a	668.23424	8.12e7

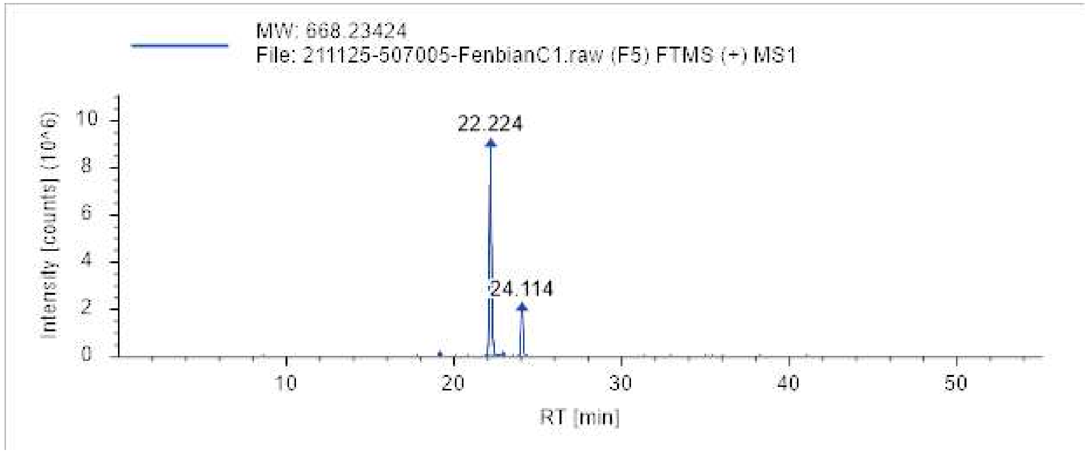

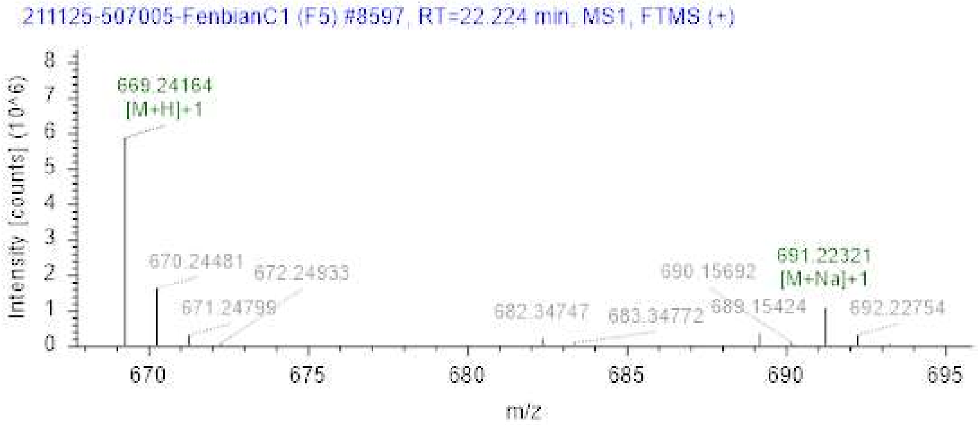

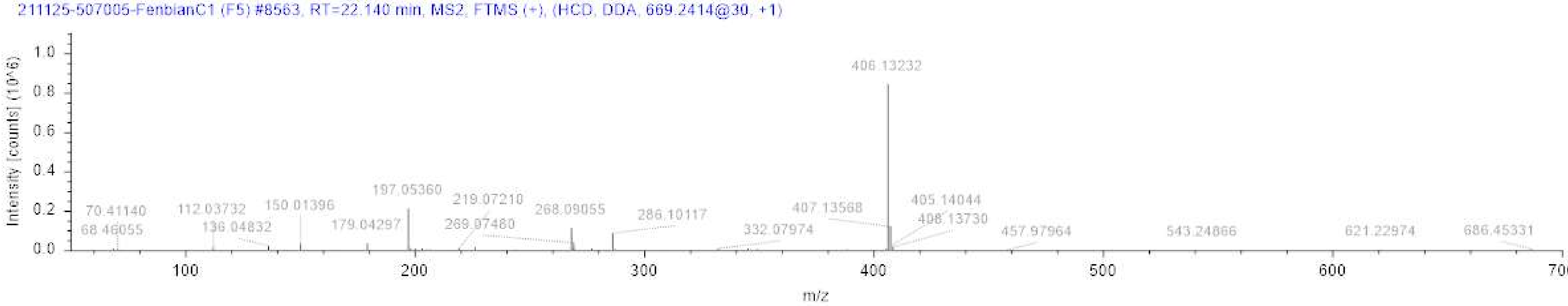

© Reported with Compound Discoverer 3.3

Compounds

Structure	Name	RT [min]	Formula	Calc. MW	Areas
n/a		29.19	n/a	668.23769	8.46e6 4.20e7 3.39e7 9.46e6 7.57e8 2.37e8

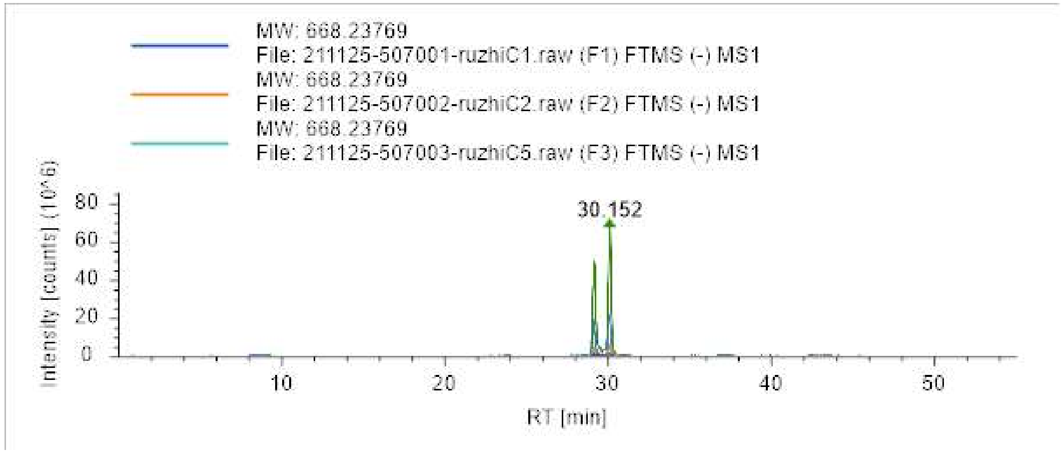

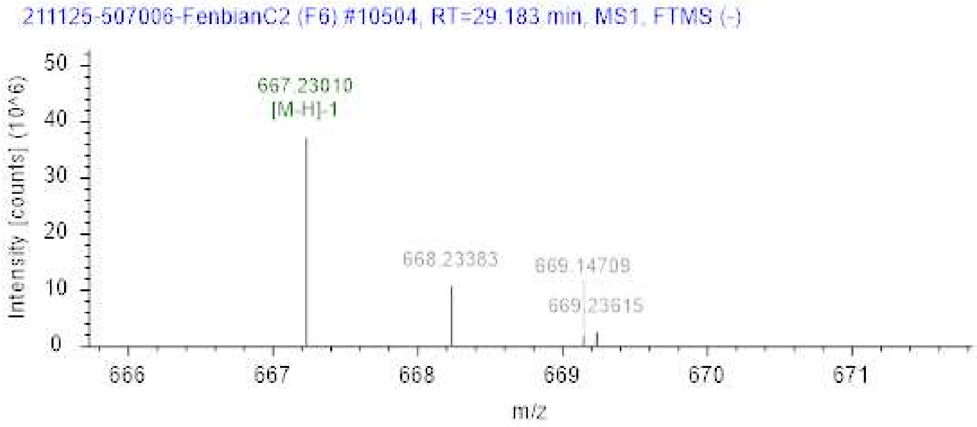

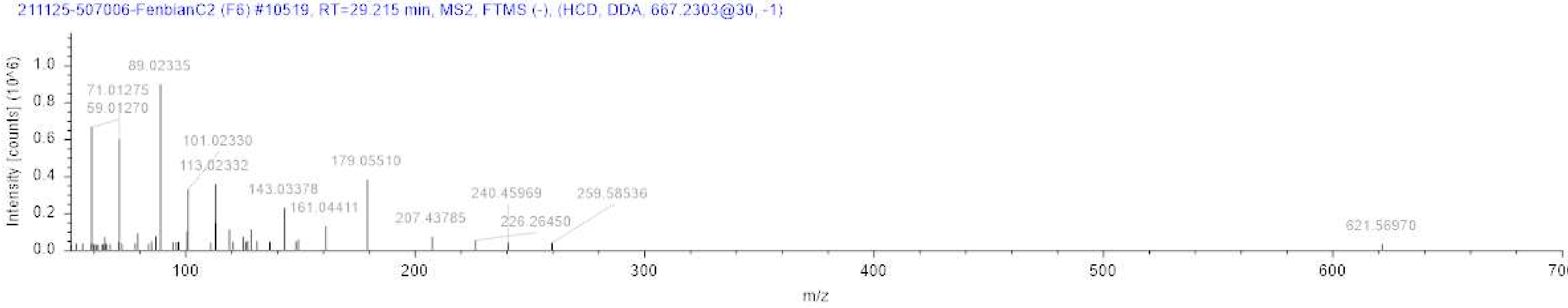

© Reported with Compound Discoverer 3.3

Structure	Name	RT [min]	Formula	Calc. MW	Areas
n/a		30.16	n/a	668.23769	1.14e7 6.17e7 4.46e7 1.08e7 9.61e8 2.09e8

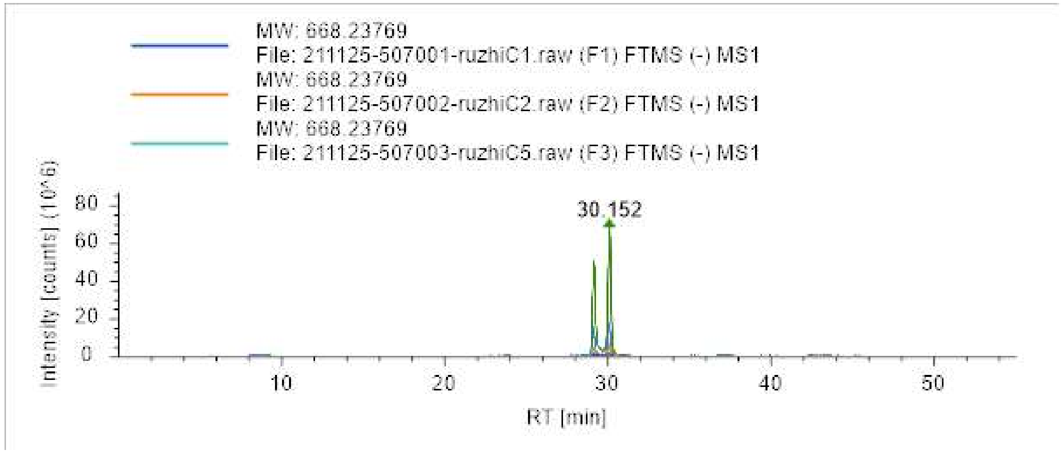

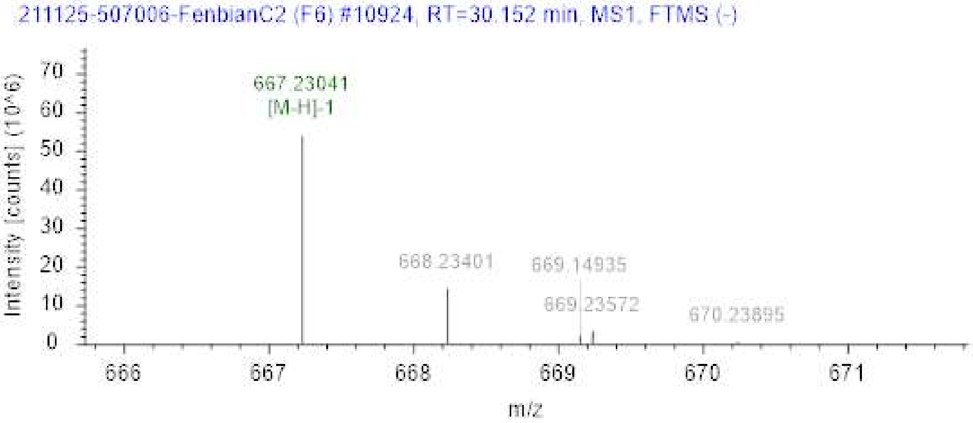

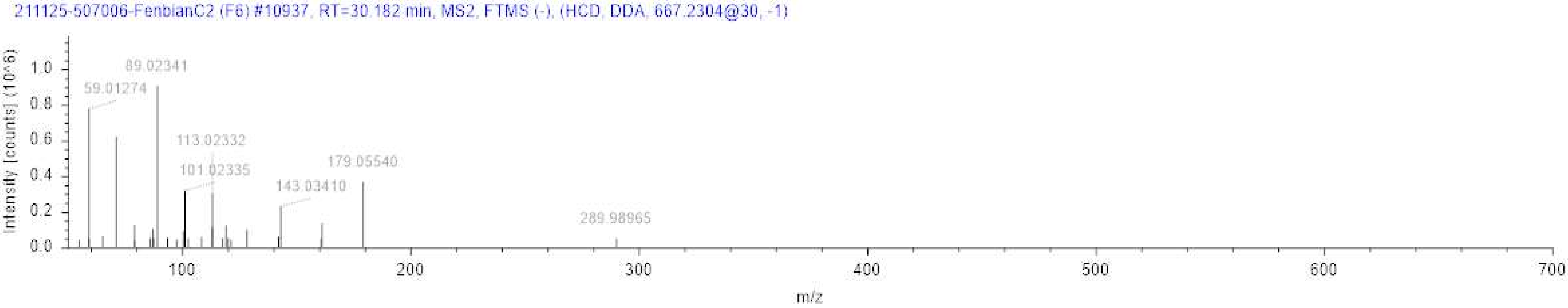

© Reported with Compound Discoverer 3.3

Compounds

Structure	Name	RT [min]	Formula	Calc. MW	Areas
n/a		33.59	n/a	674.23625	1.47e8

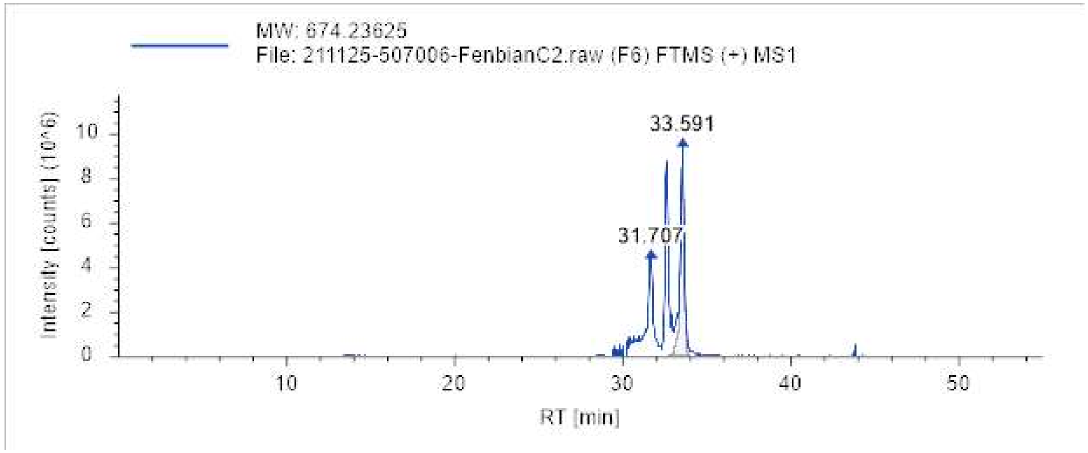

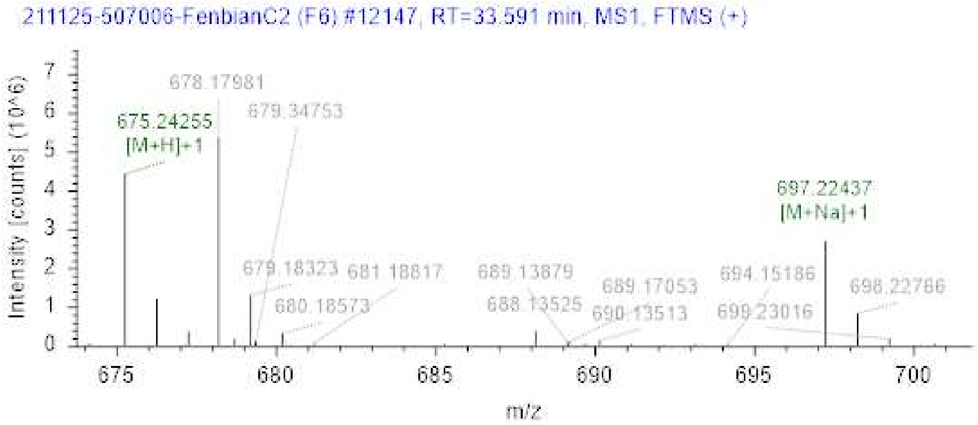

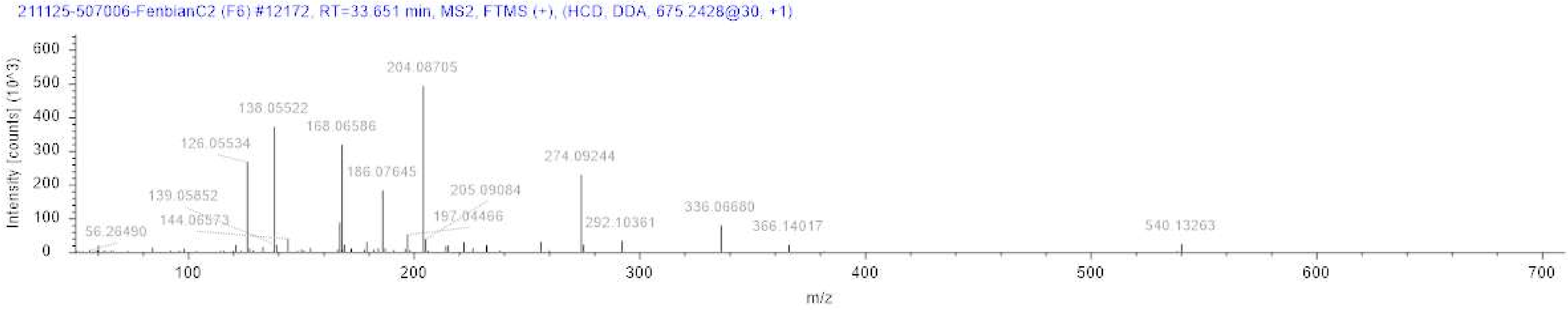

© Reported with Compound Discoverer 3.3

Structure	Name	RT [min]	Formula	Calc. MW	Areas
n/a		32.65	n/a	674.23906	2.40e8; 2.44e8

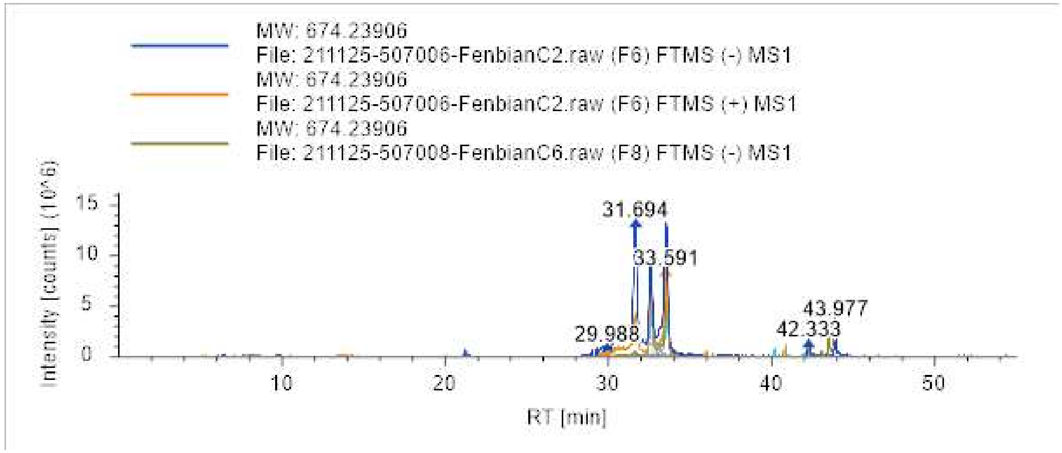

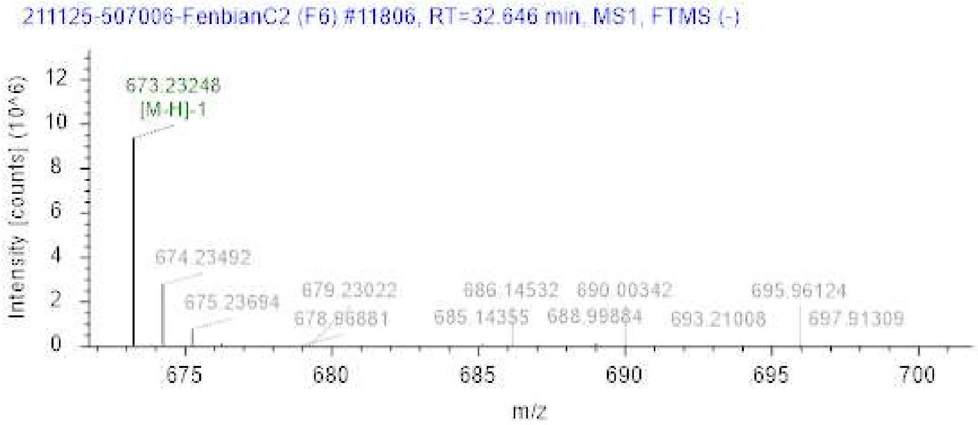

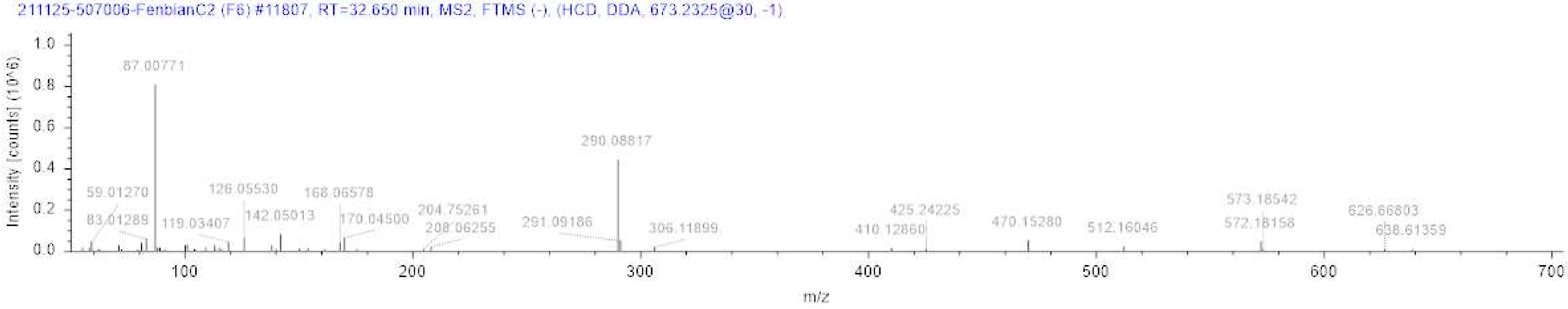

© Reported with Compound Discoverer 3.3

Structure	Name	RT [min]	Formula	Calc. MW	Areas
n/a		31.70	n/a	674.24043	2.32e8

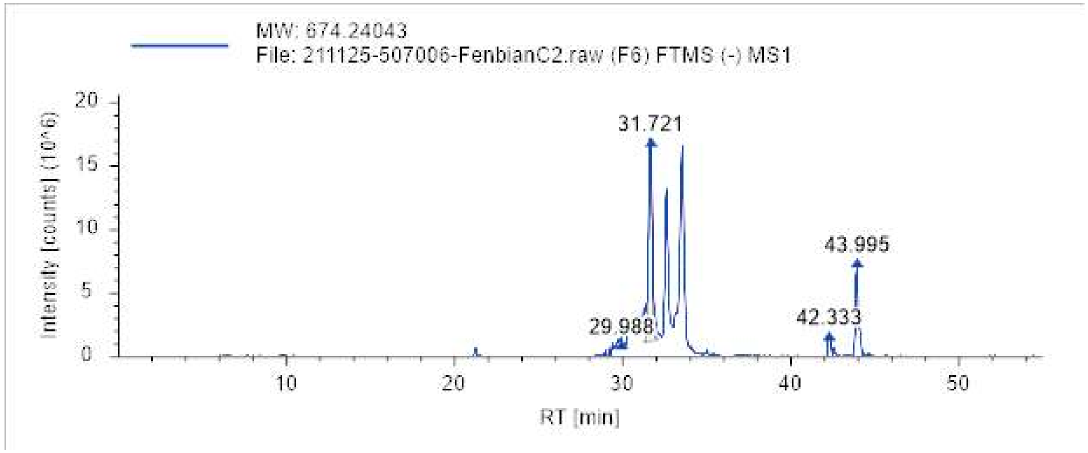

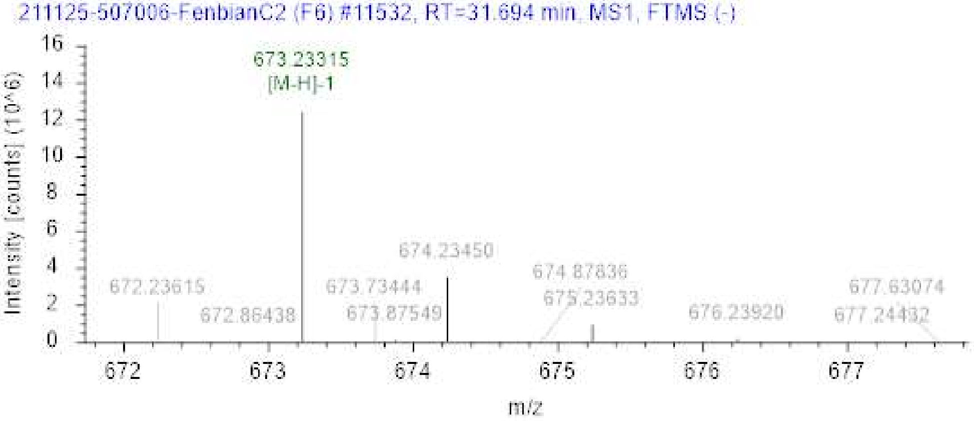

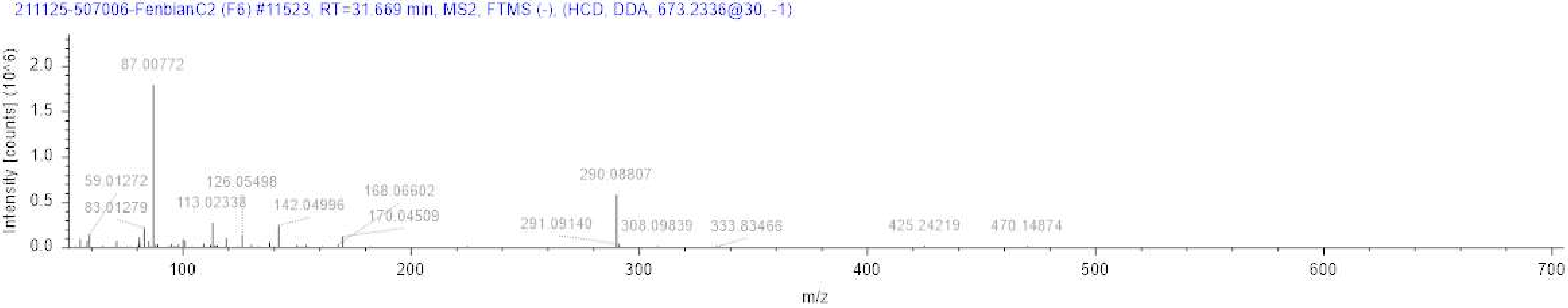

© Reported with Compound Discoverer 3.3

Compounds

Structure	Name	RT [min]	Formula	Calc. MW	Areas
n/a		37.45	n/a	675.25565	8.33e8

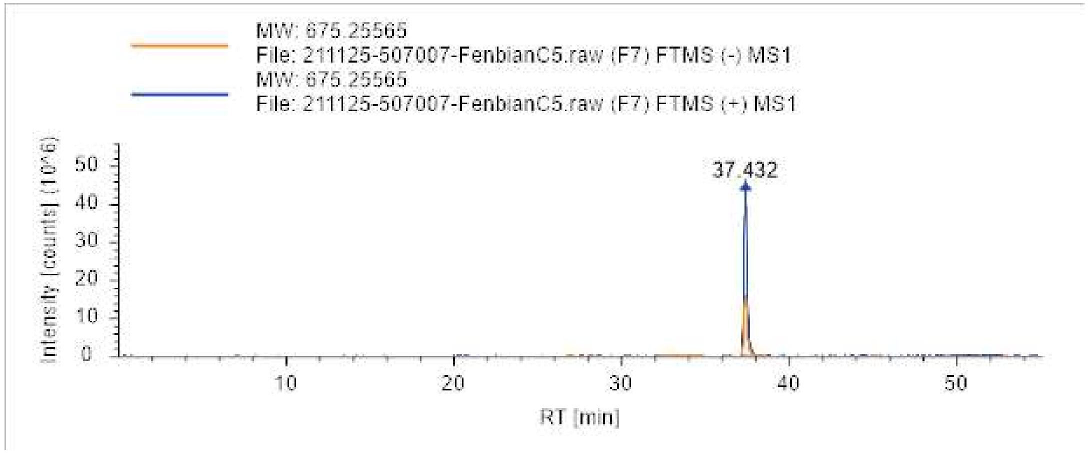

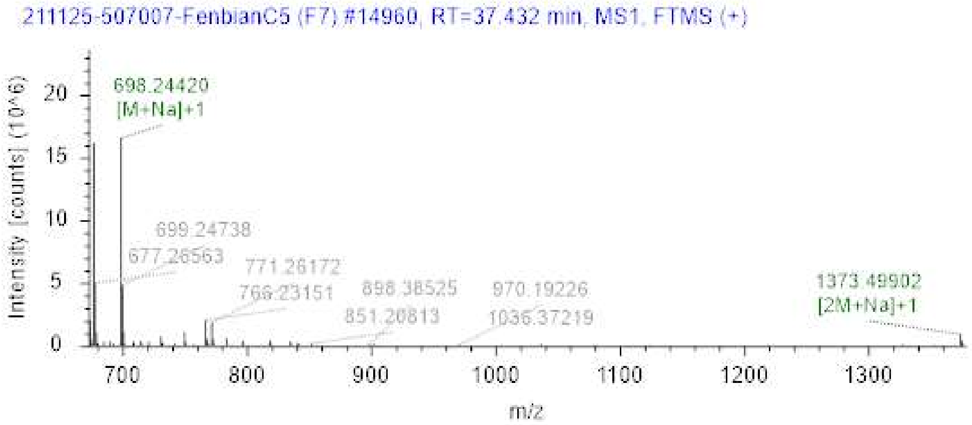

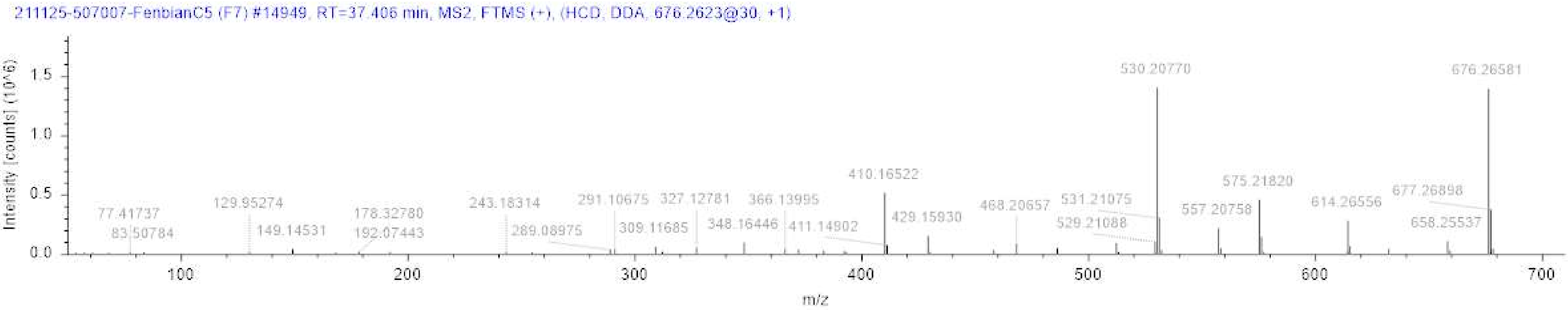

© Reported with Compound Discoverer 3.3

Compounds

Structure	Name	RT [min]	Formula	Calc. MW	Areas
n/a		34.54	n/a	675.25568	3.89e7

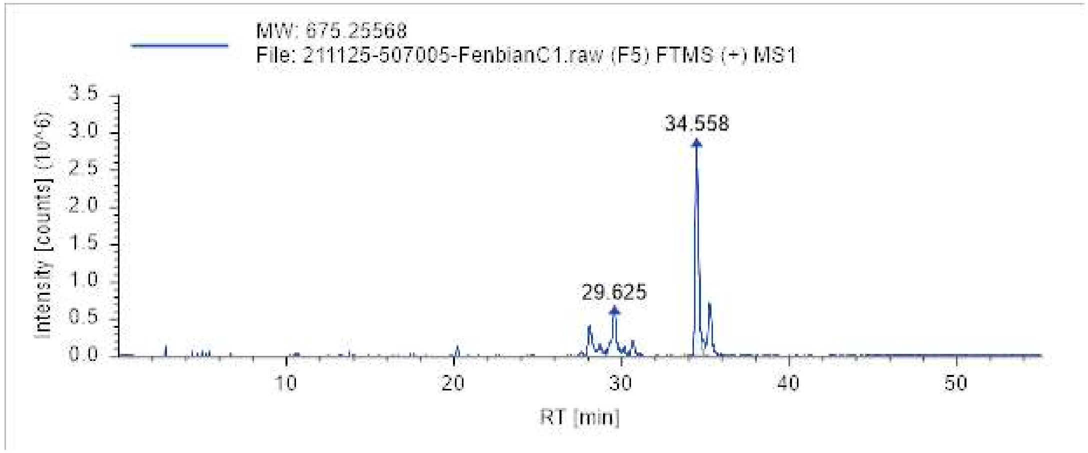

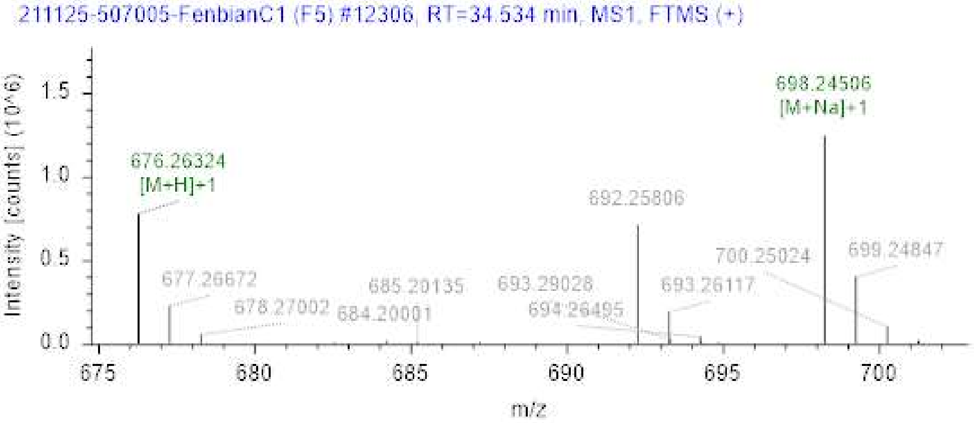

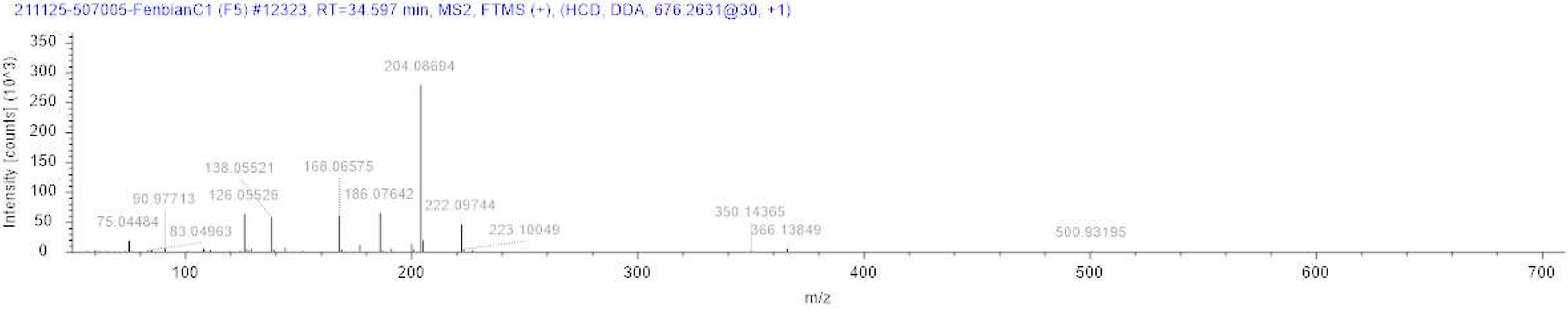

© Reported with Compound Discoverer 3.3

Compounds

Structure	Name	RT [min]	Formula	Calc. MW	Areas
n/a		31.17	n/a	675.25807	2.73e8

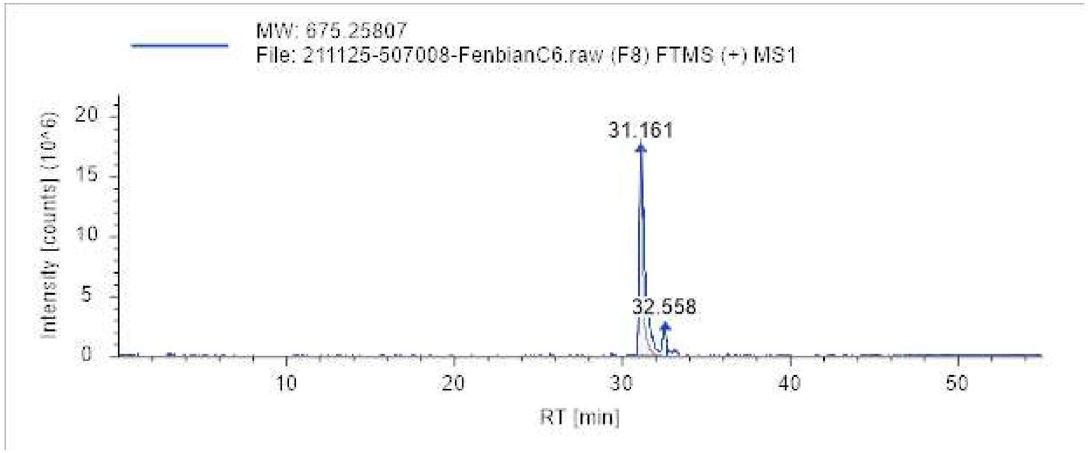

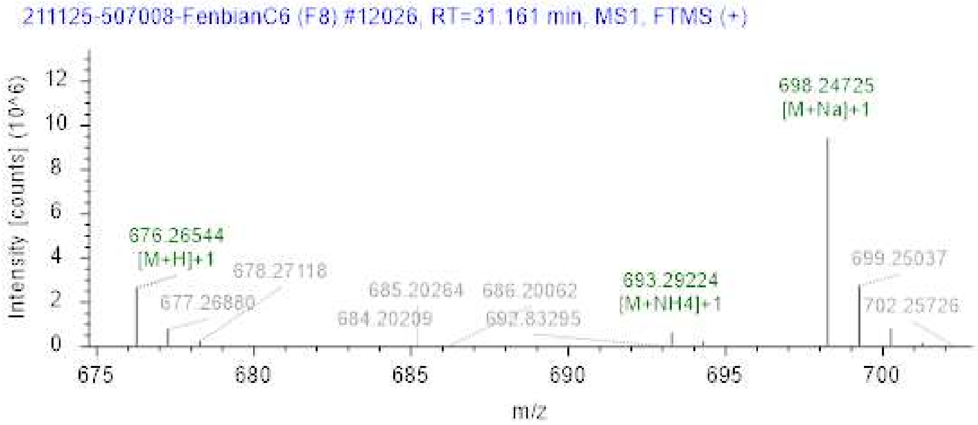

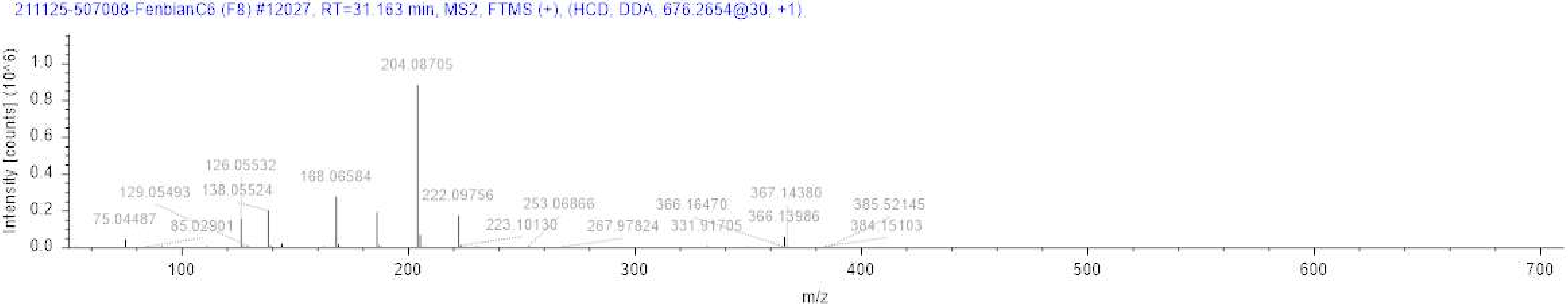

© Reported with Compound Discoverer 3.3

Structure	Name	RT [min]	Formula	Calc. MW	Areas
n/a		32.52	n/a	675.25817	7.04e6

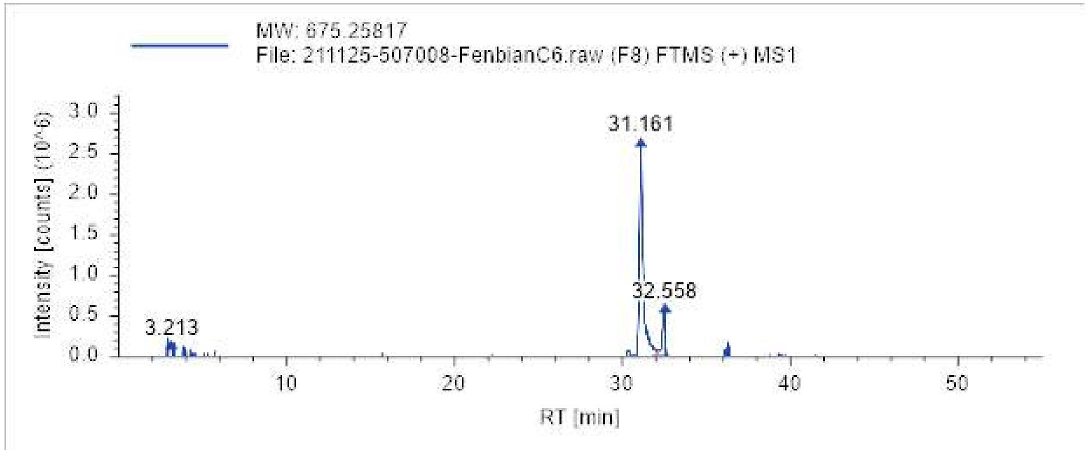

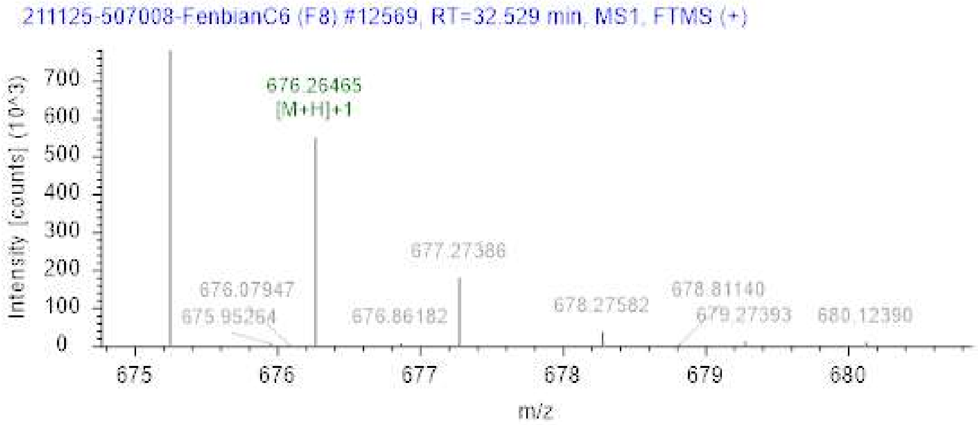

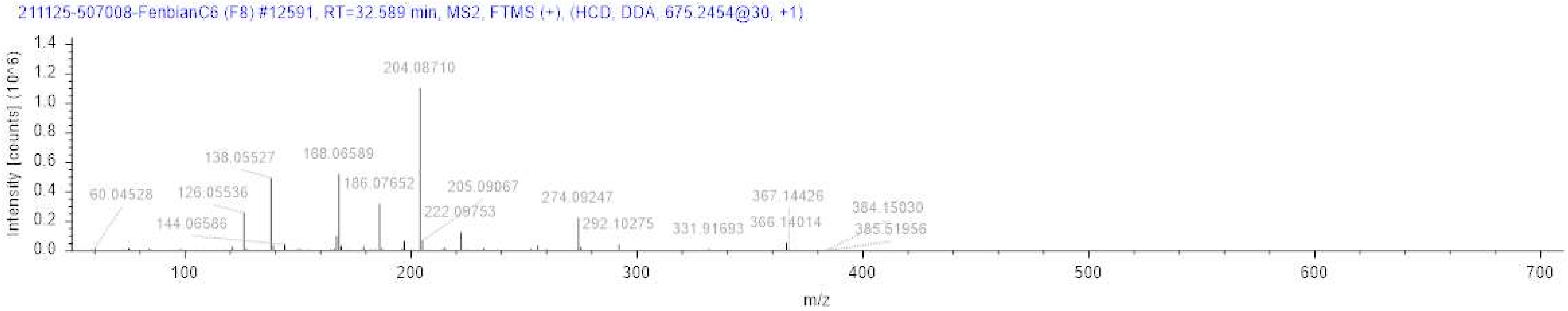

© Reported with Compound Discoverer 3.3

Structure	Name	RT [min]	Formula	Calc. MW	Areas
n/a		36.85	n/a	691.25121	1.80e8

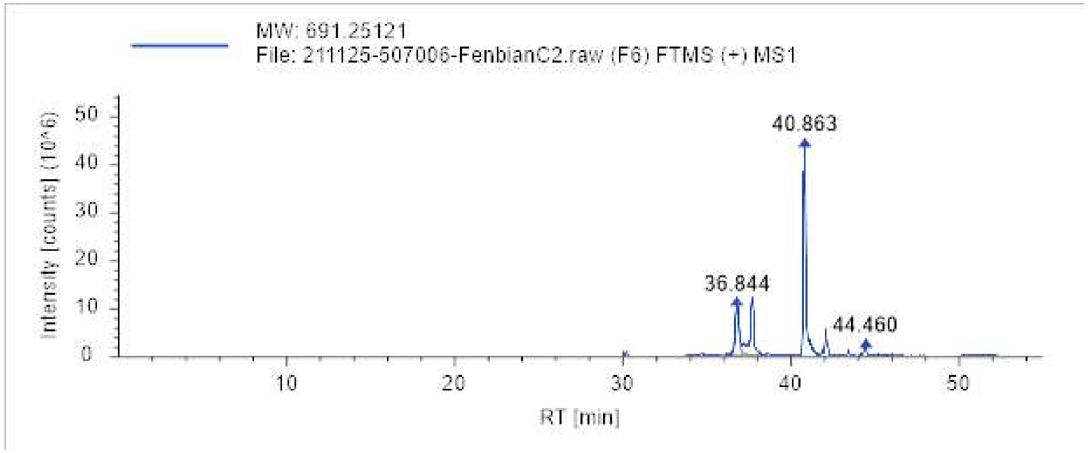

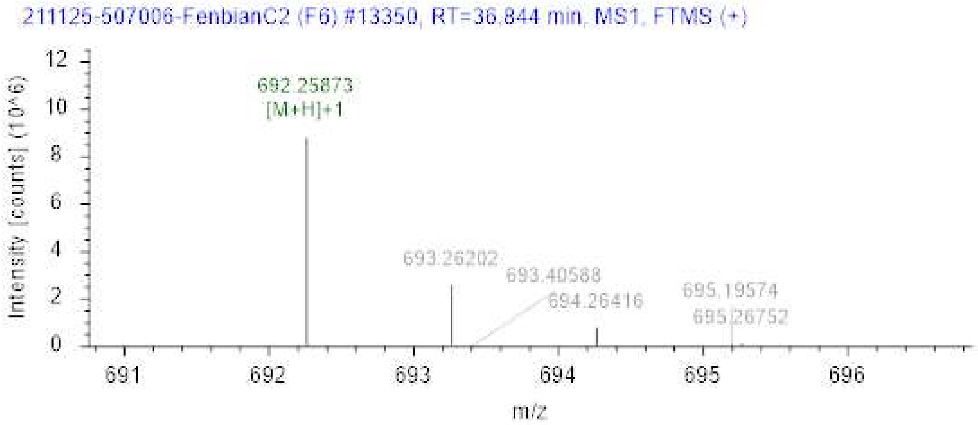

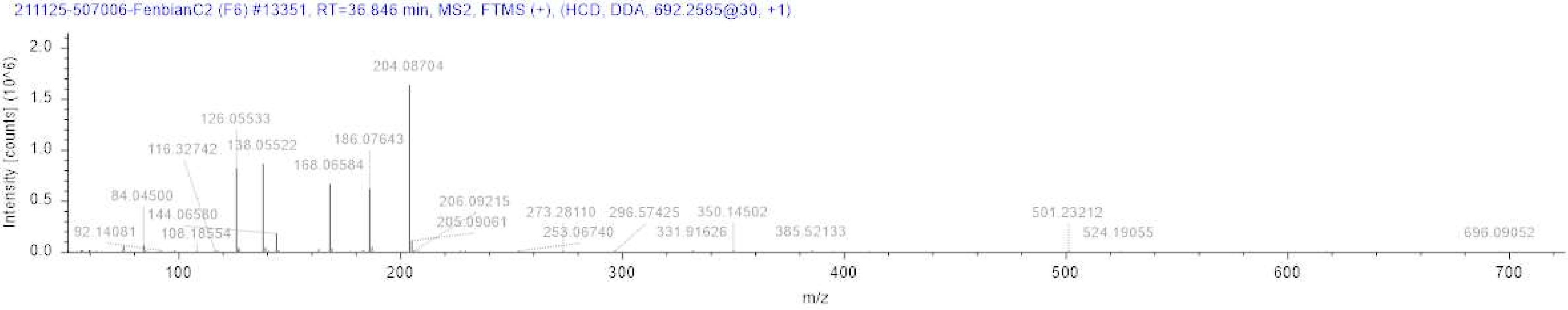

© Reported with Compound Discoverer 3.3

Compounds

Structure	Name	RT [min]	Formula	Calc. MW	Areas
n/a		43.45	n/a	691.25121	6.65e6

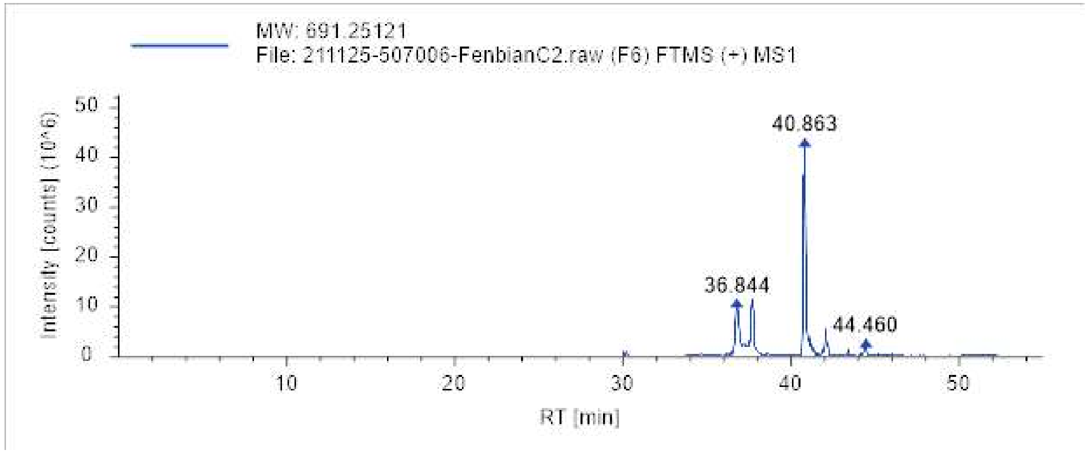

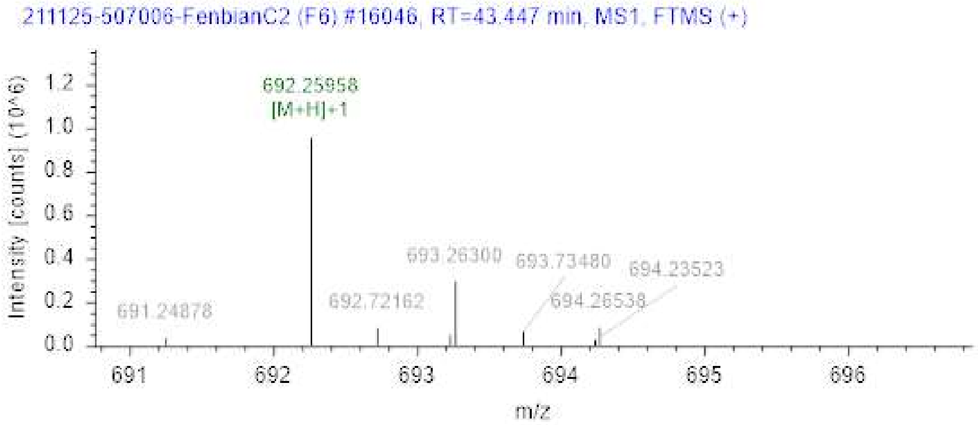

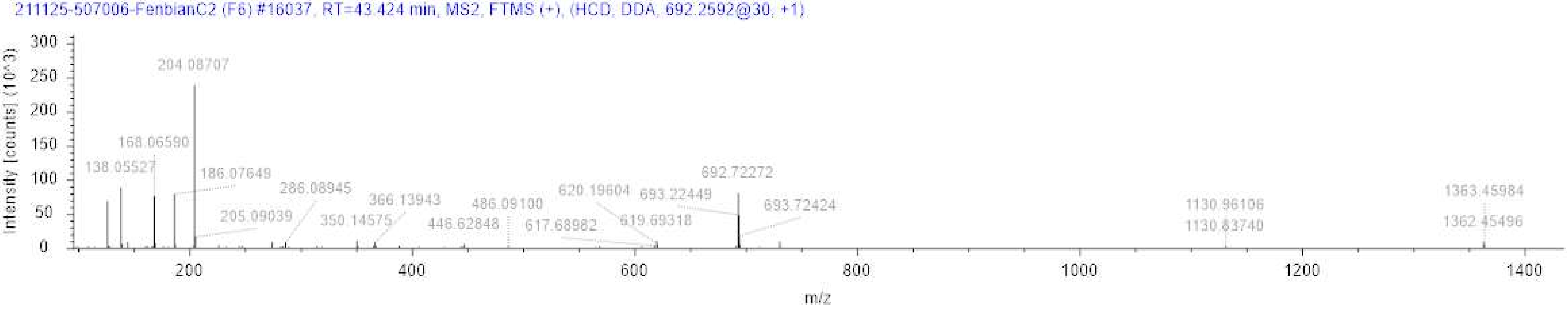

© Reported with Compound Discoverer 3.3

Structure	Name	RT [min]	Formula	Calc. MW	Areas
n/a		34.70	n/a	691.25133	5.17e7 … 2.48e7

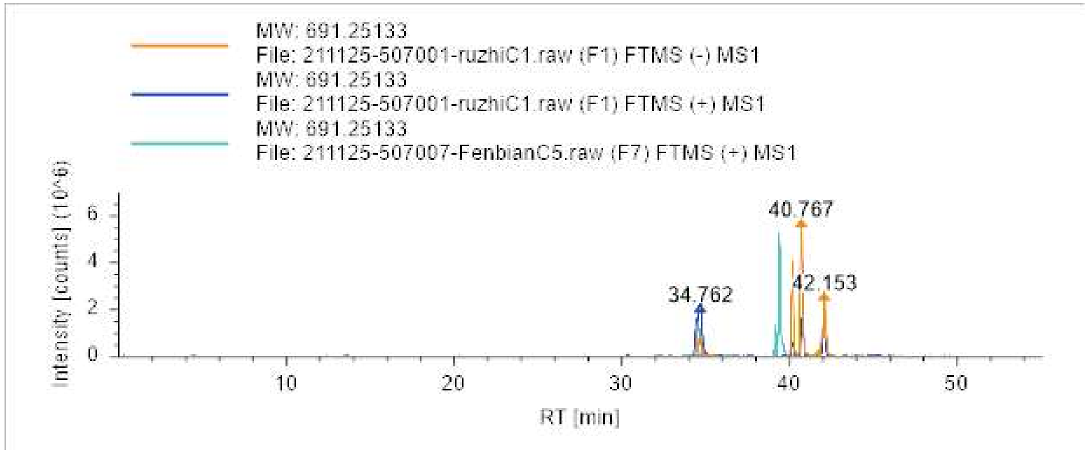

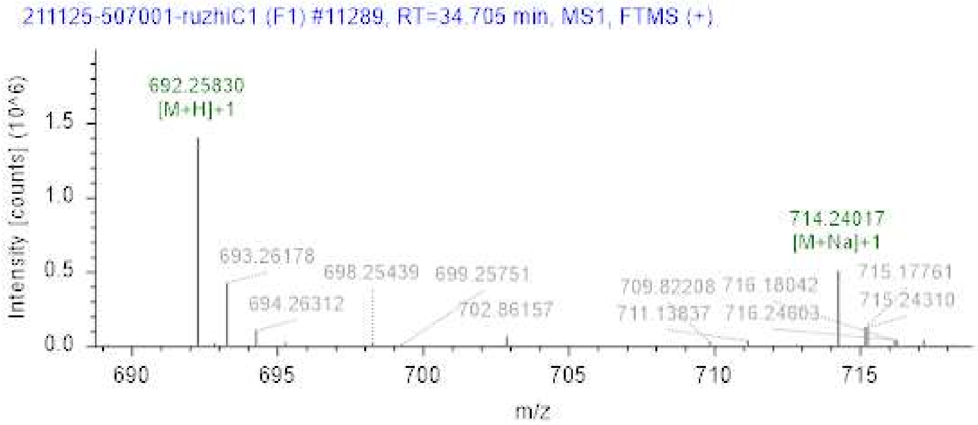

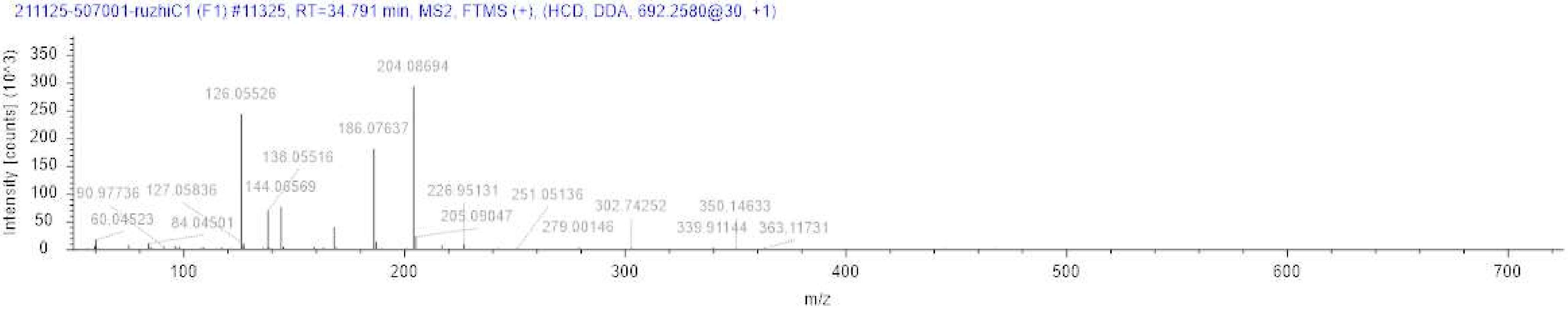

© Reported with Compound Discoverer 3.3

Compounds

Structure	Name	RT [min]	Formula	Calc. MW	Areas
n/a		44.47	n/a	691.25231	2.94e7

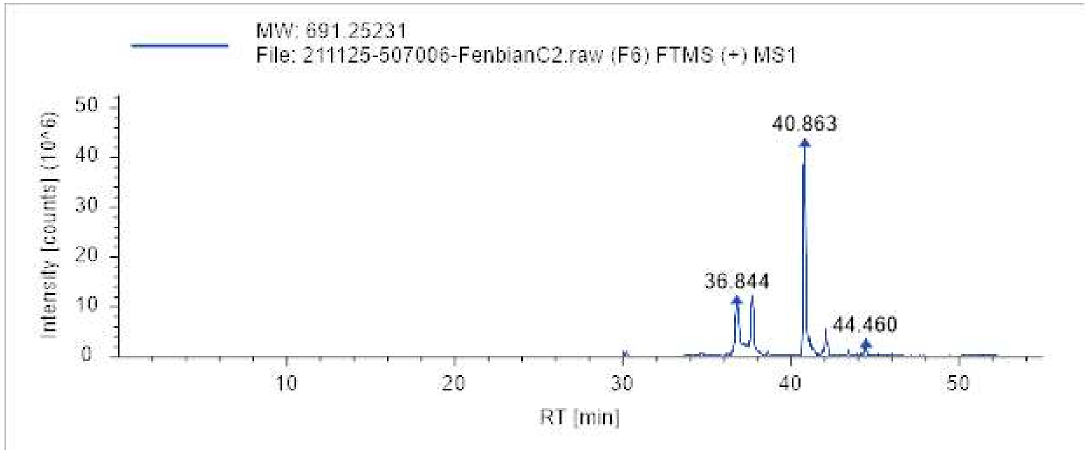

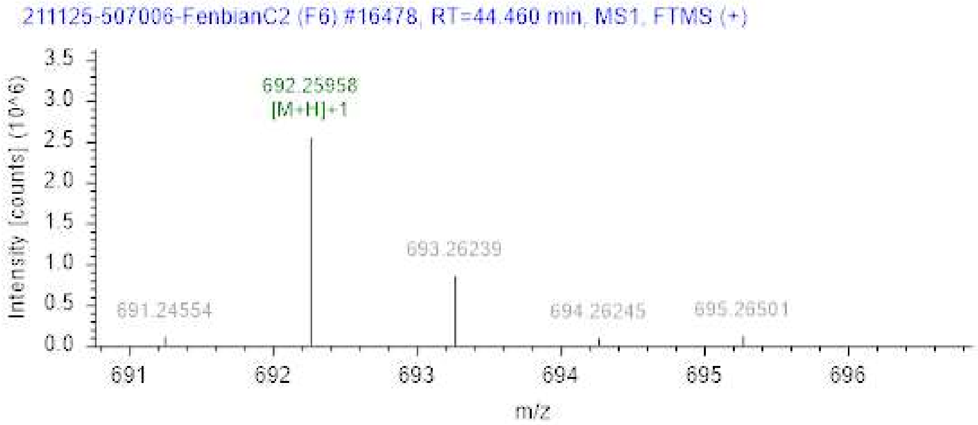

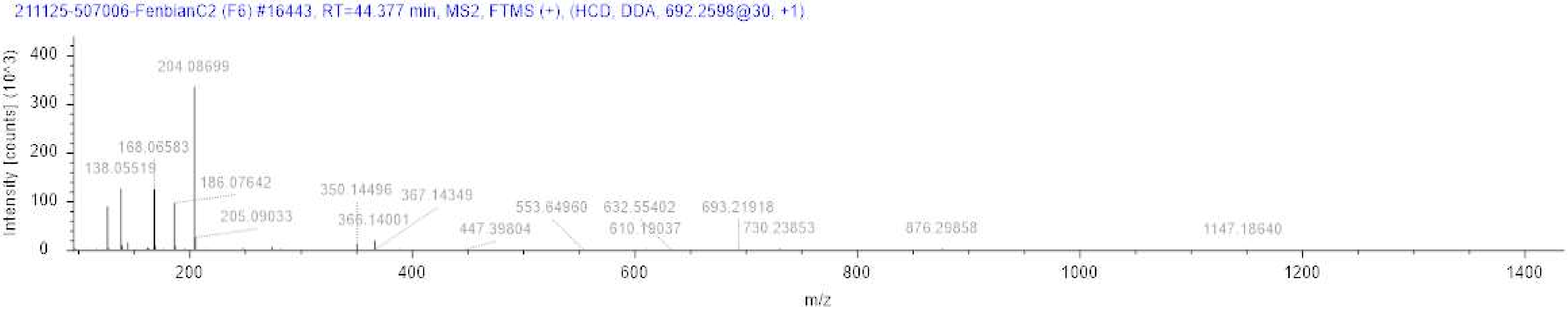

© Reported with Compound Discoverer 3.3

Structure	Name	RT [min]	Formula	Calc. MW	Areas
n/a		40.82	n/a	691.25281	6.00e7 2.77e8 9.85e7 4.41e7 1.81e9 4.41e8

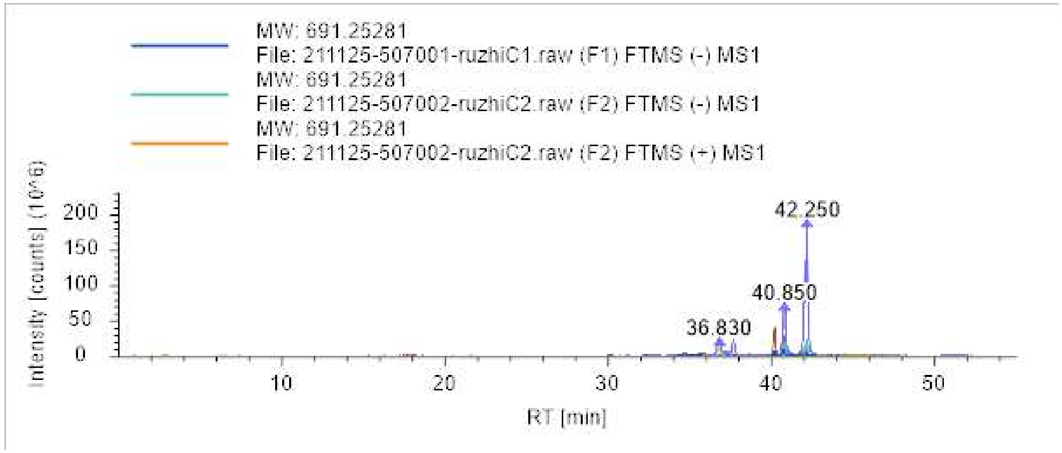

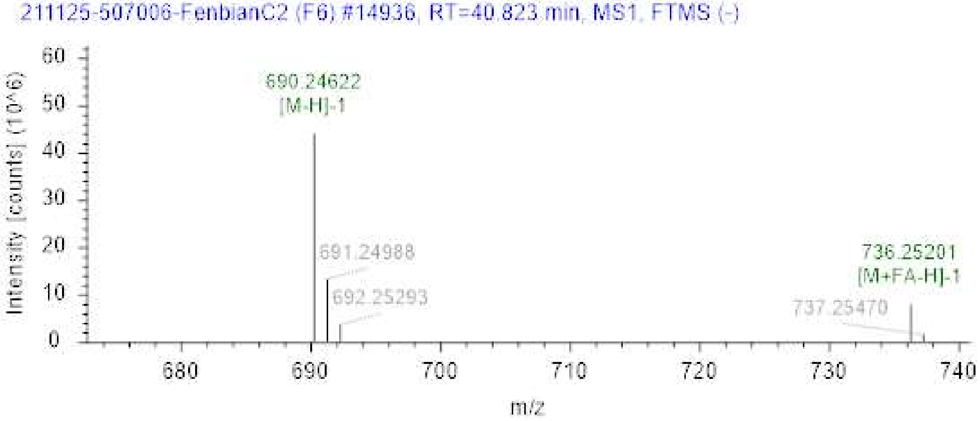

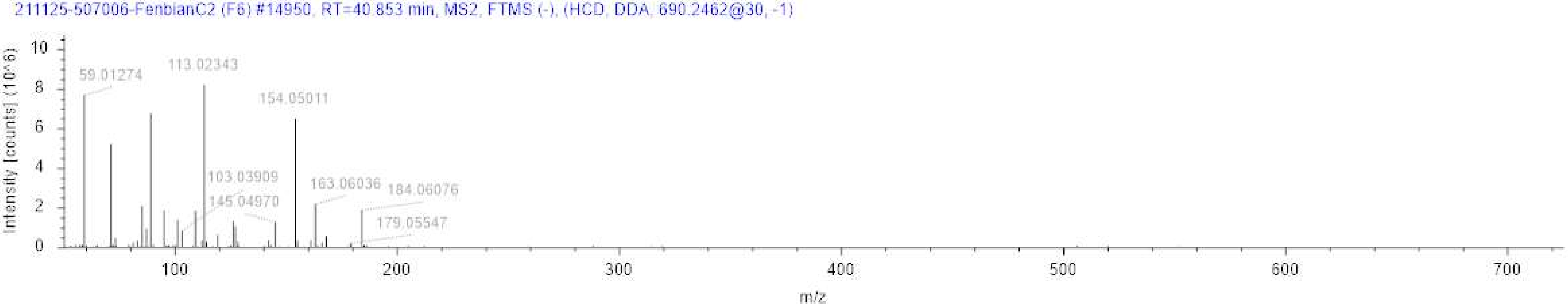

© Reported with Compound Discoverer 3.3

Compounds

Structure	Name	RT [min]	Formula	Calc. MW	Areas
n/a		39.46	n/a	691.25288	7.88e6

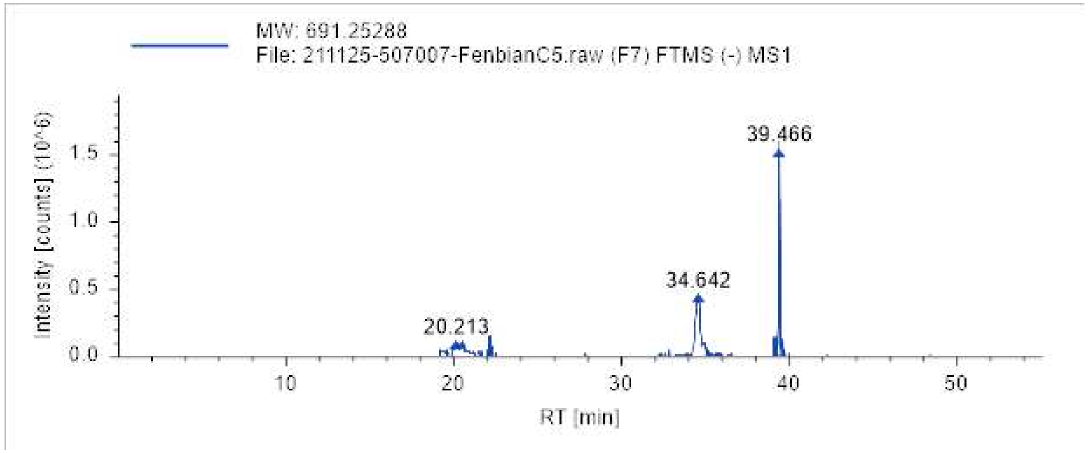

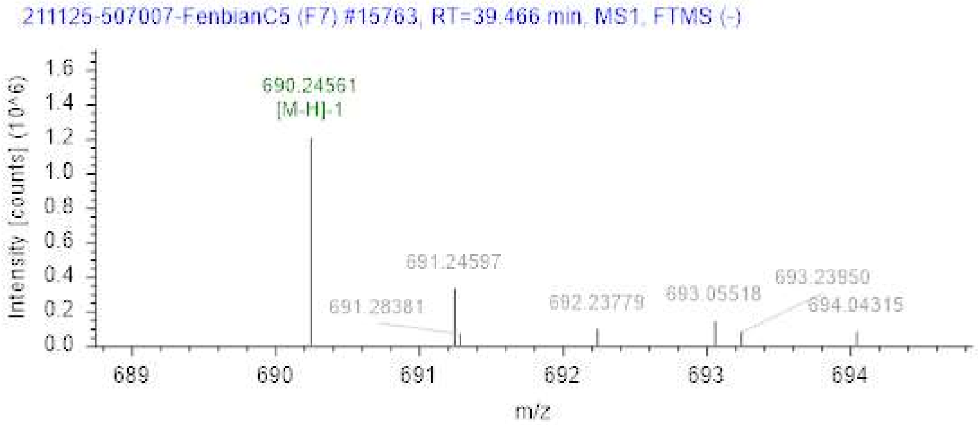

© Reported with Compound Discoverer 3.3

Compounds

Structure	Name	RT [min]	Formula	Calc. MW	Areas
n/a		42.23	n/a	691.25355	3.32e7 1.74e8 1.40e8 1.06e7 1.57e9 1.02e8

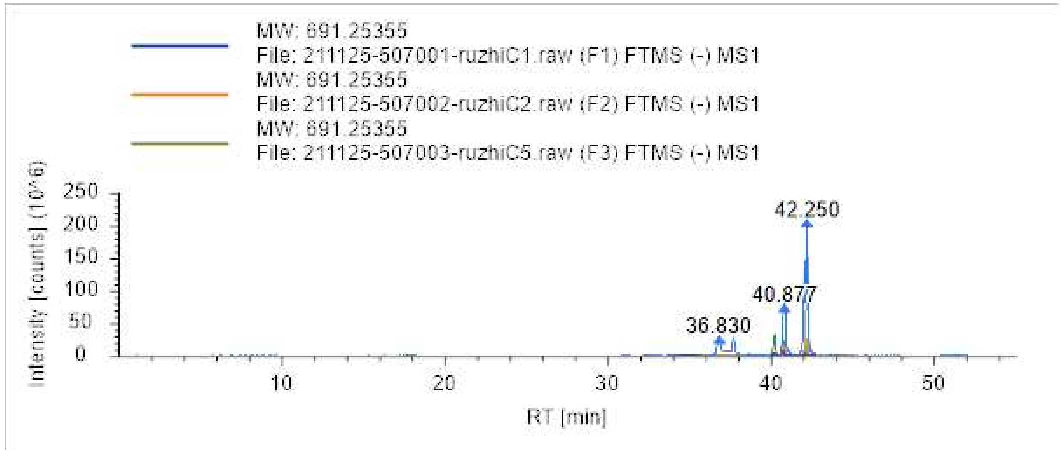

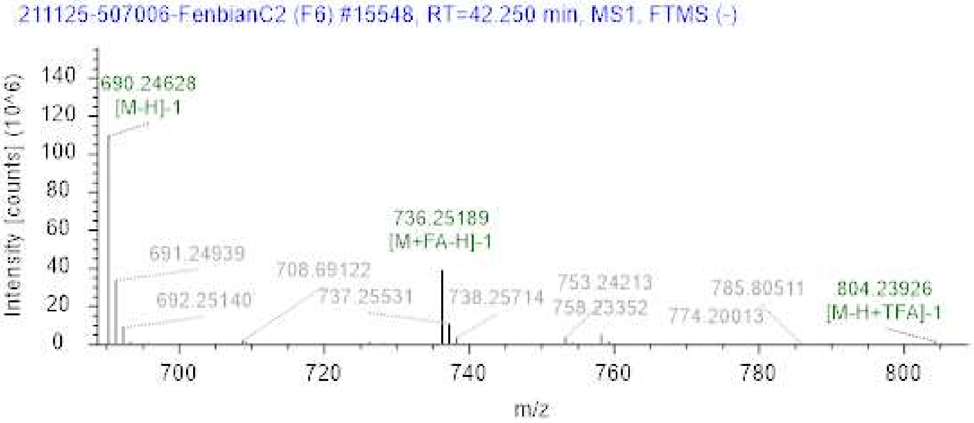

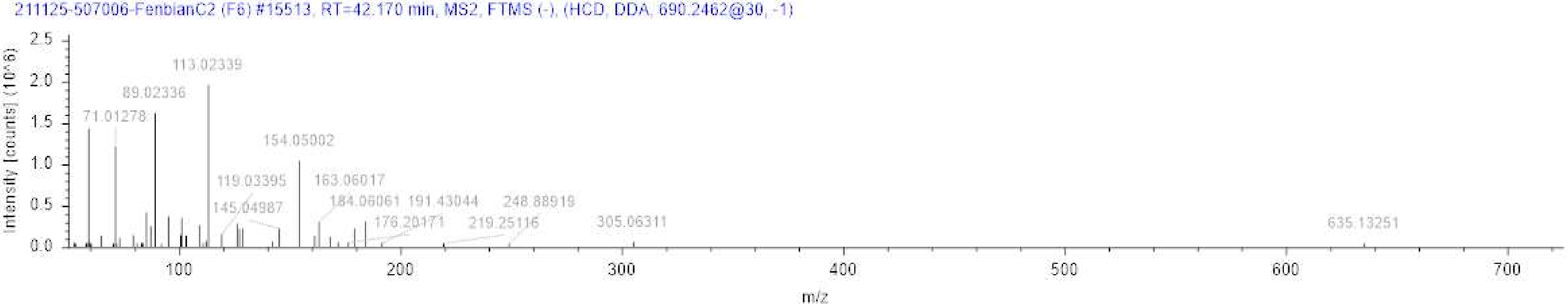

© Reported with Compound Discoverer 3.3

Compounds

Structure	Name	RT [min]	Formula	Calc. MW	Areas
n/a		40.24	n/a	691.25369	2.45e7 1.68e7 4.38e7 3.99e8

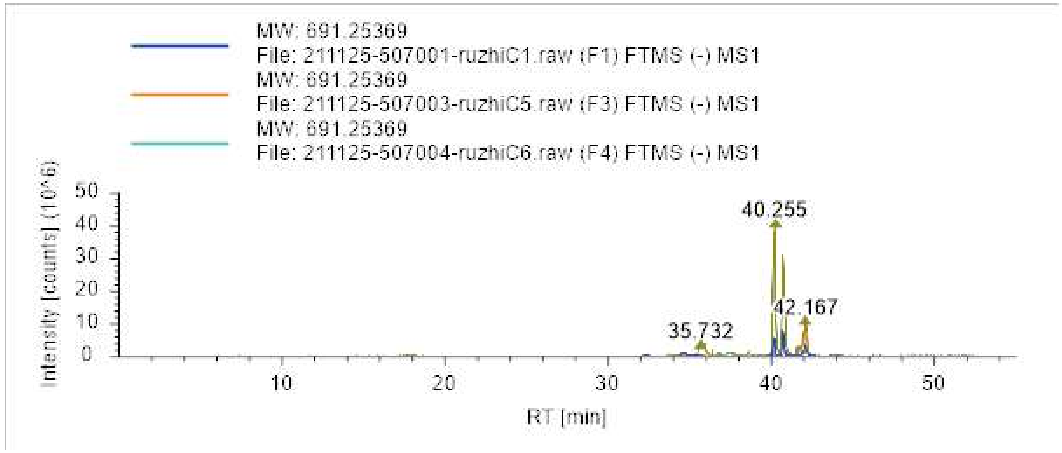

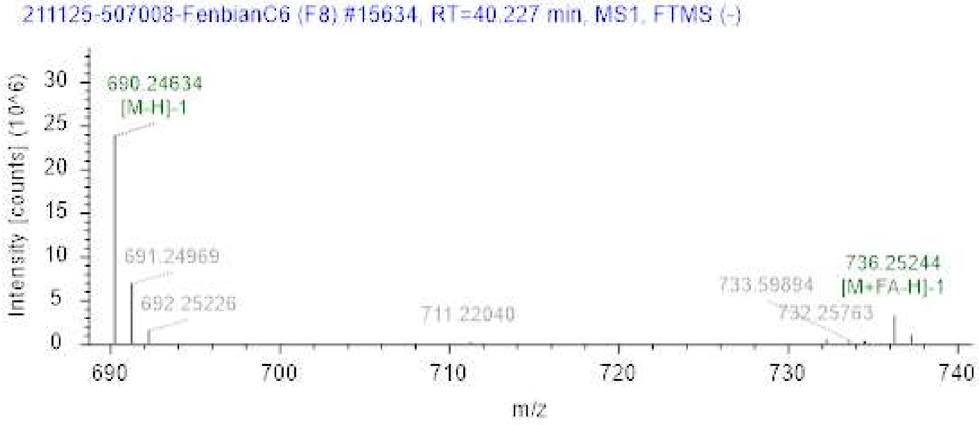

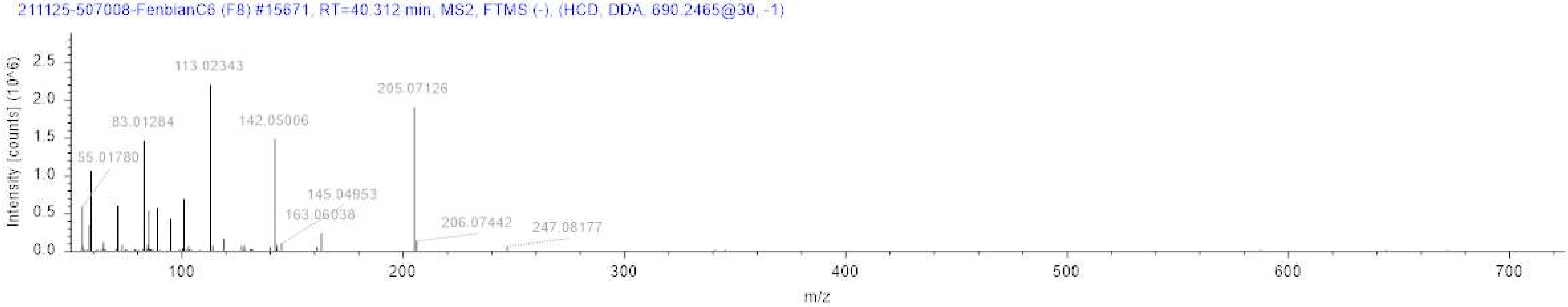

© Reported with Compound Discoverer 3.3

Compounds

Structure	Name	RT [min]	Formula	Calc. MW	Areas
n/a		37.74	n/a	691.25482	4.22e8

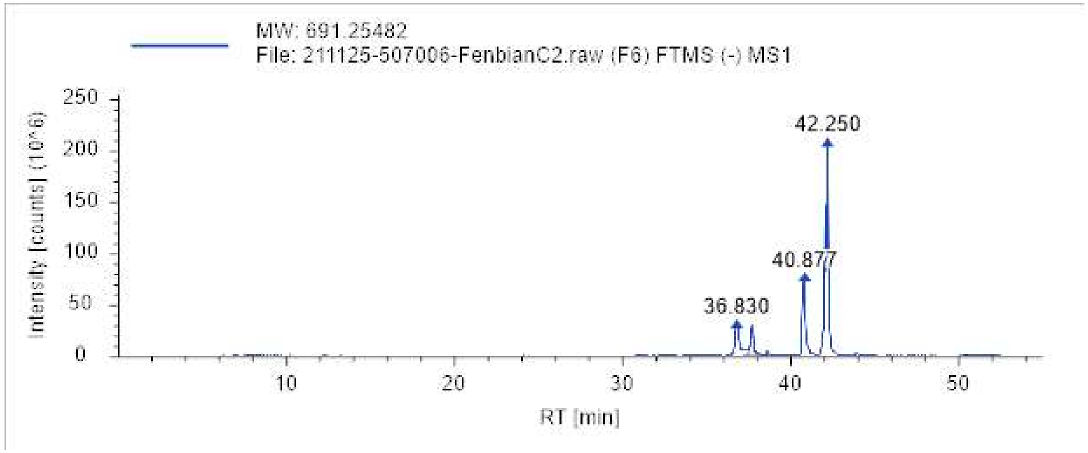

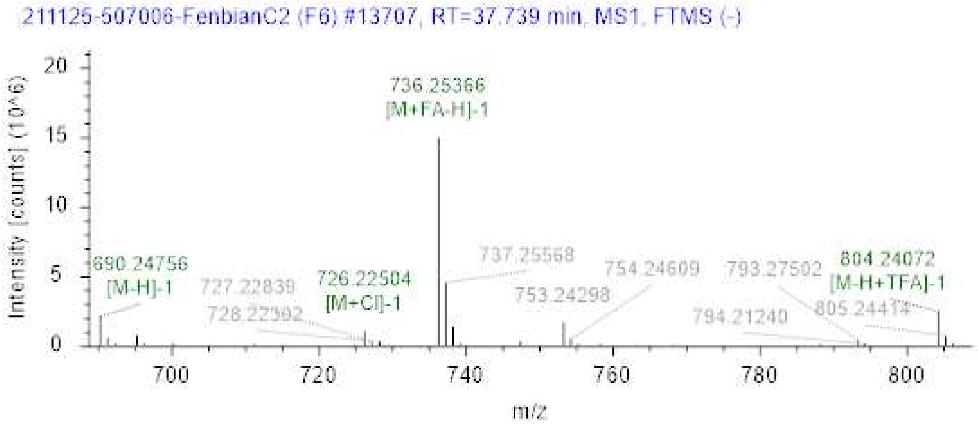

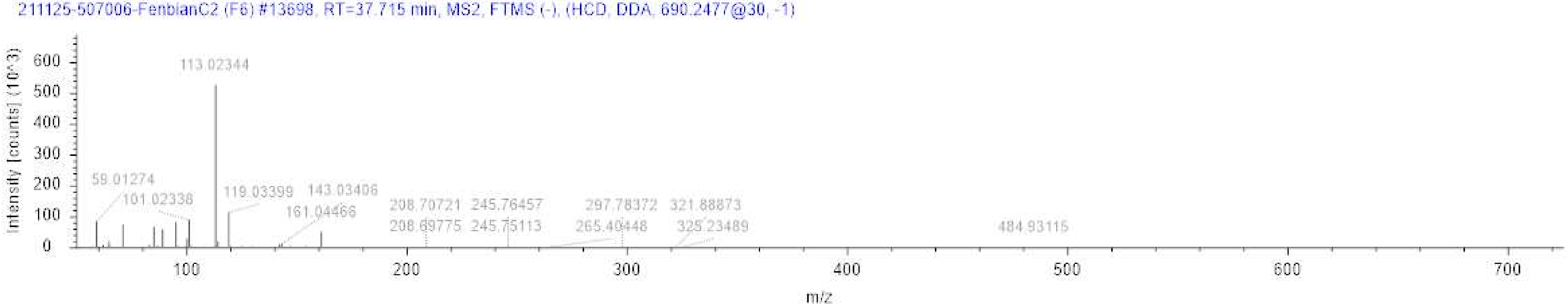

© Reported with Compound Discoverer 3.3

Structure	Name	RT [min]	Formula	Calc. MW	Areas
n/a		35.77	n/a	691.25561	1.53e8

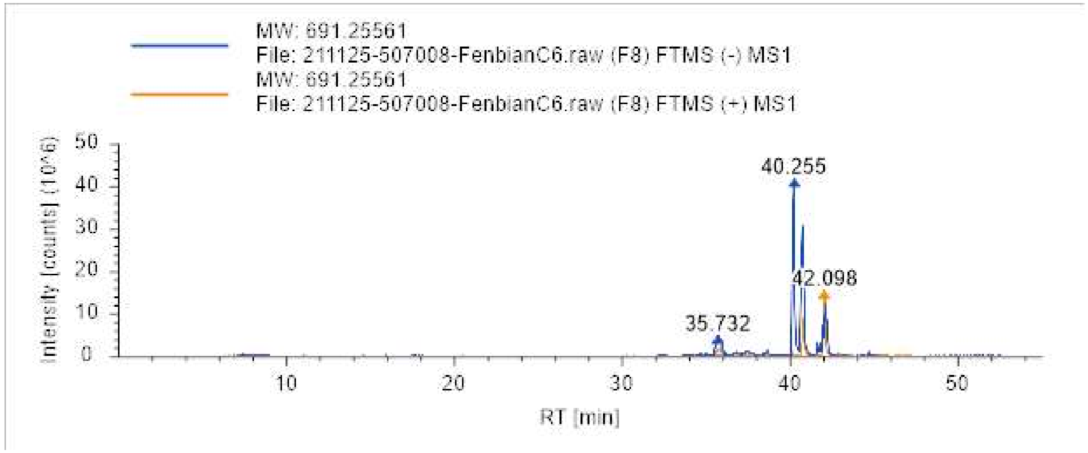

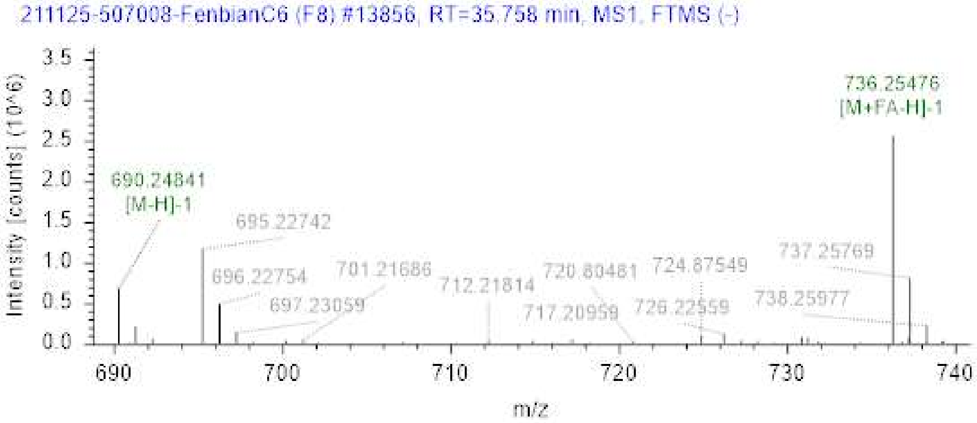

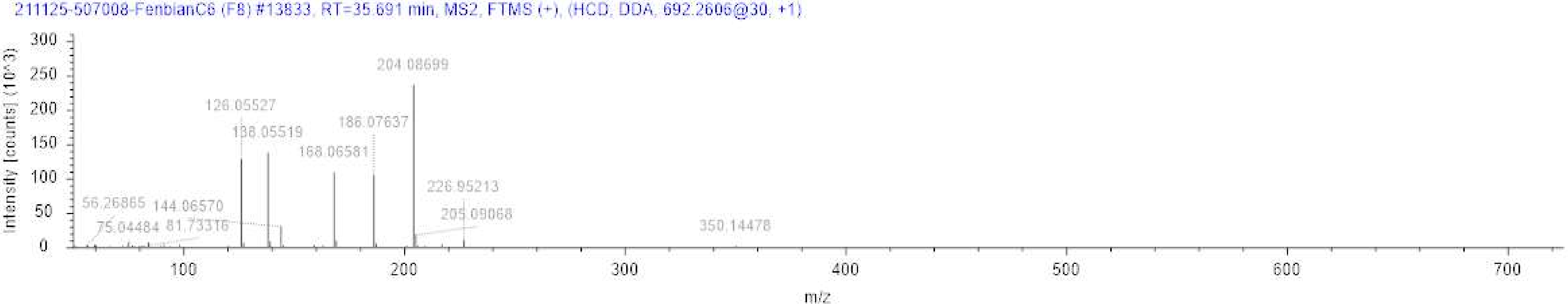

© Reported with Compound Discoverer 3.3

Compounds

Structure	Name	RT [min]	Formula	Calc. MW	Areas
n/a		2.27	n/a	692.25136	2.19e7

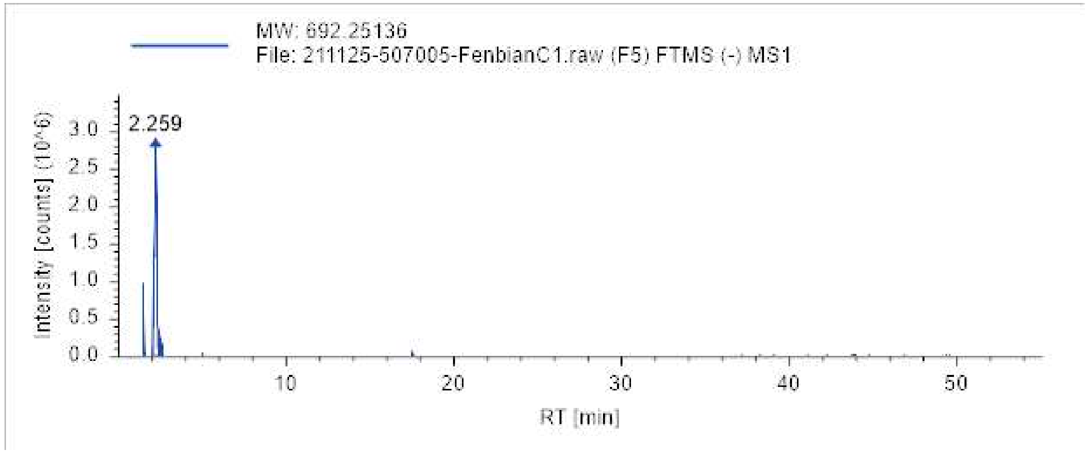

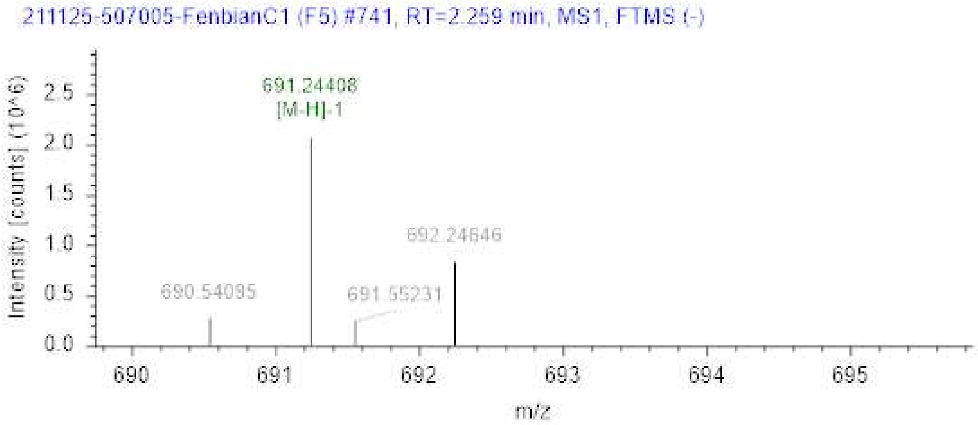

© Reported with Compound Discoverer 3.3

Compounds

Structure	Name	RT [min]	Formula	Calc. MW	Areas
n/a		40.75	n/a	707.24640	1.02e8 8.96e7 9.29e7 3.98e7 5.14e8 4.31e8

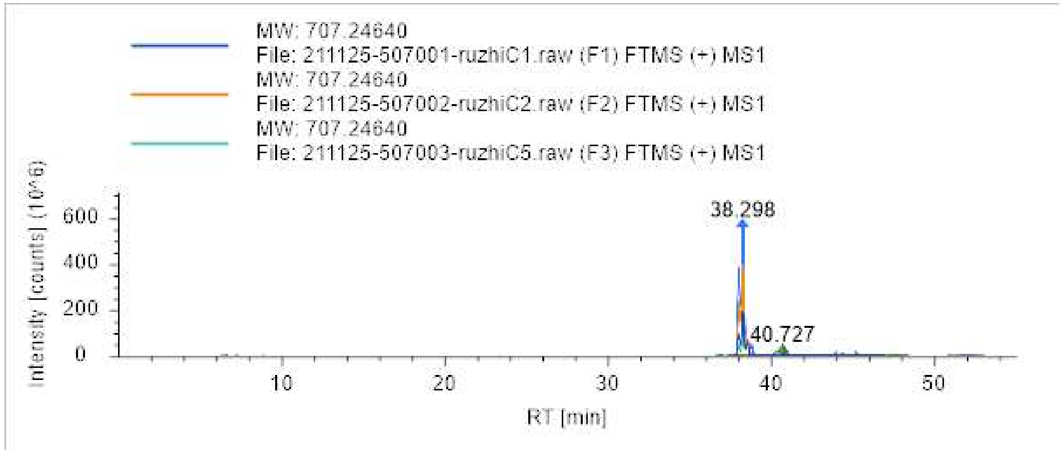

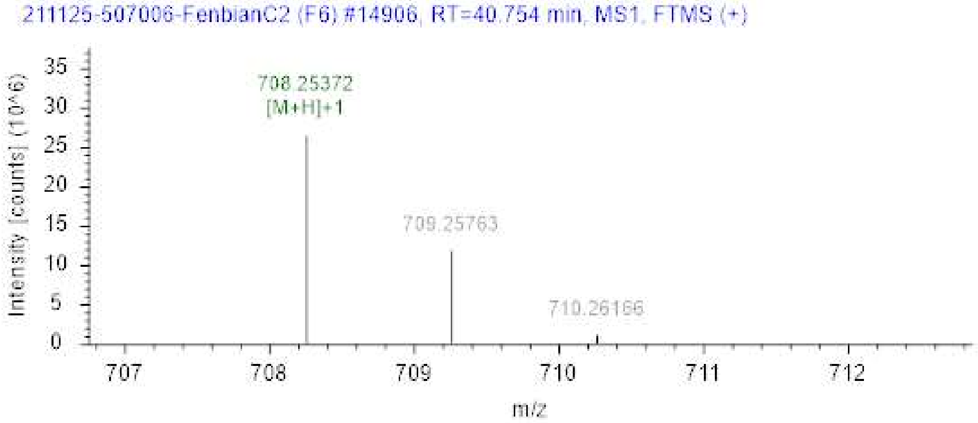

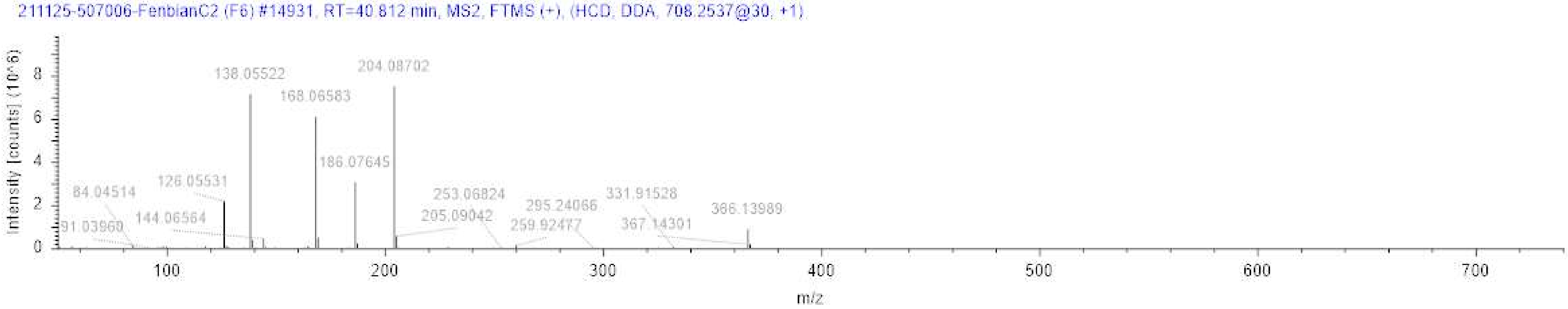

© Reported with Compound Discoverer 3.3

Structure	Name	RT [min]	Formula	Calc. MW	Areas
n/a		44.32	n/a	707.24651	2.49e7; 4.20e7

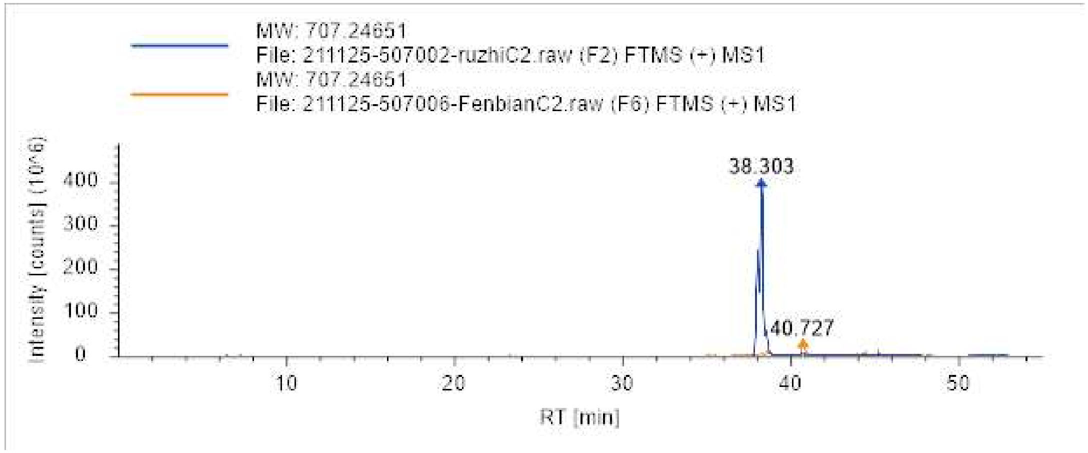

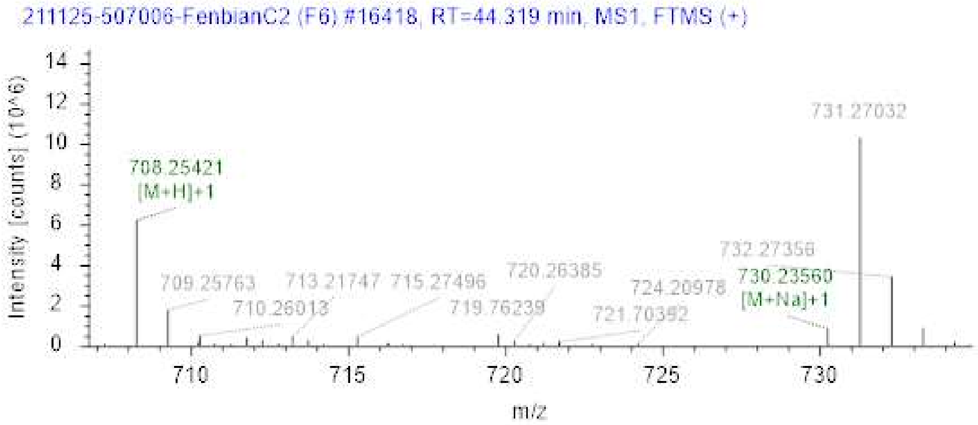

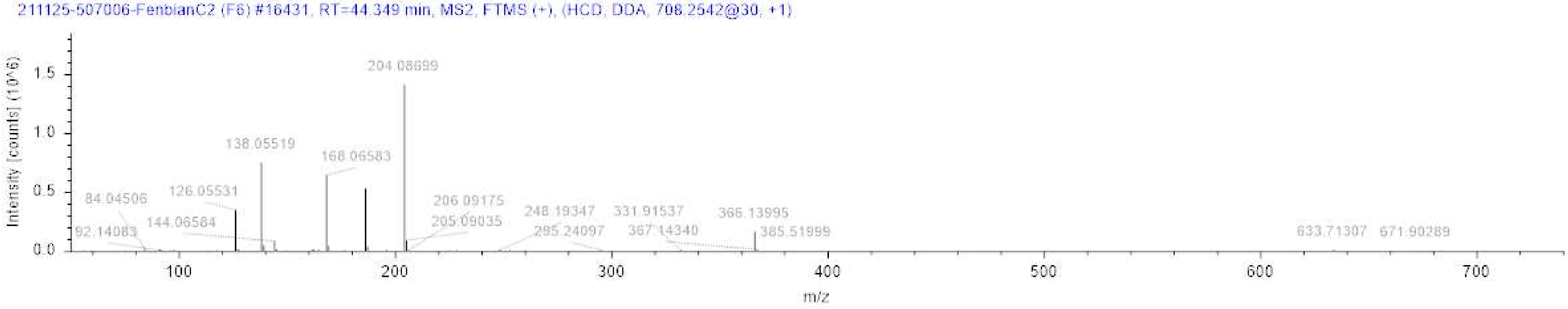

© Reported with Compound Discoverer 3.3

Compounds

Structure	Name	RT [min]	Formula	Calc. MW	Areas
n/a		41.92	n/a	707.24669	8.27e6 1.05e7 4.55e7

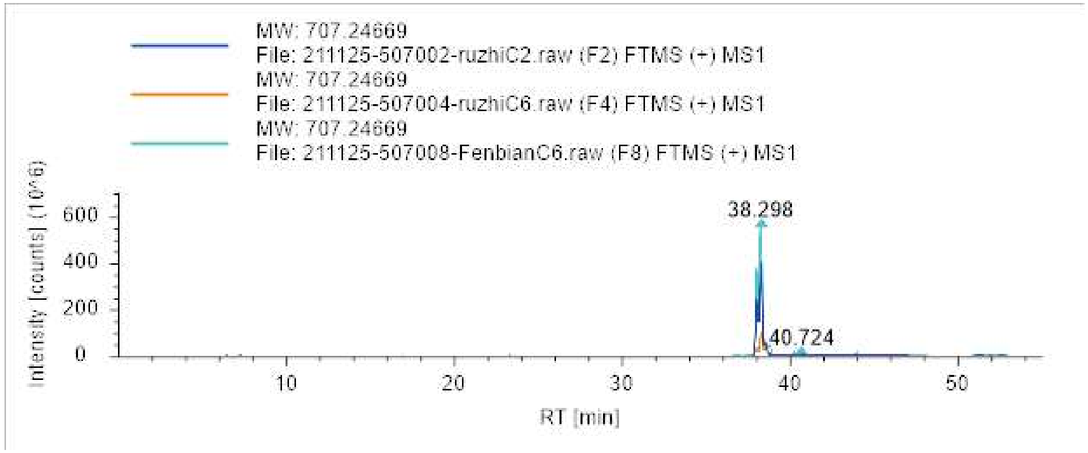

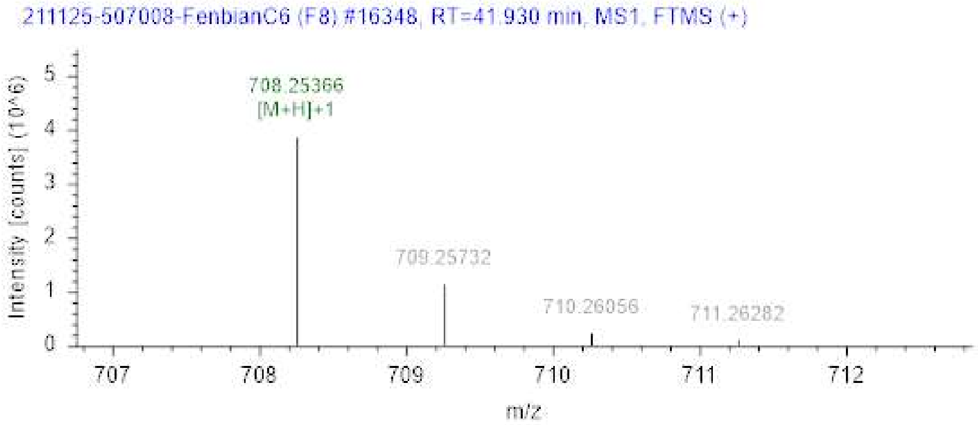

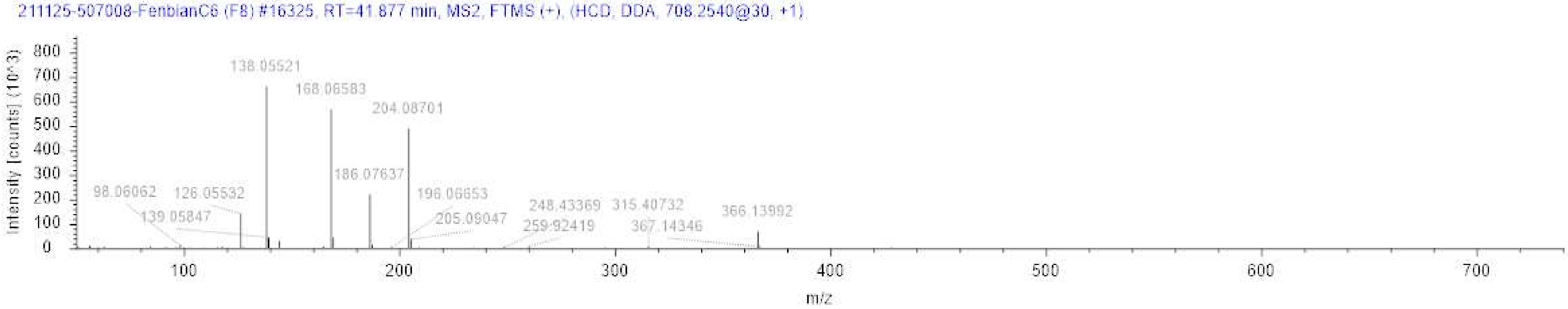

© Reported with Compound Discoverer 3.3

Compounds

Structure	Name	RT [min]	Formula	Calc. MW	Areas
n/a		45.25	n/a	707.24675	3.97e7; 1.68e8

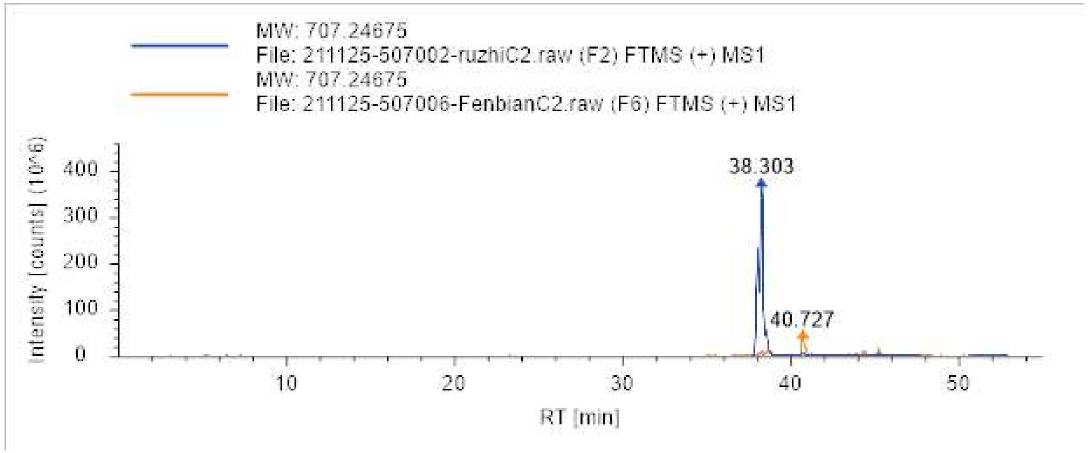

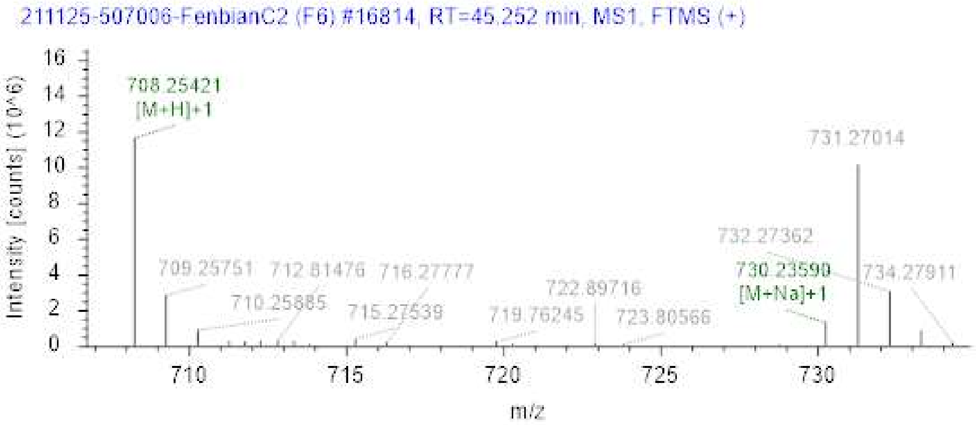

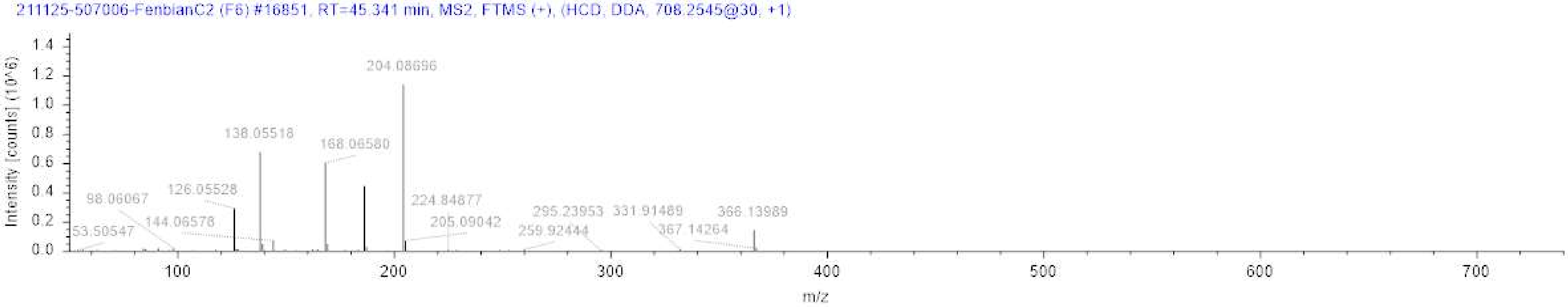

© Reported with Compound Discoverer 3.3

Compounds

Structure	Name	RT [min]	Formula	Calc. MW	Areas
n/a		40.24	n/a	707.24680	2.27e7 8.73e6 1.57e7 2.05e8

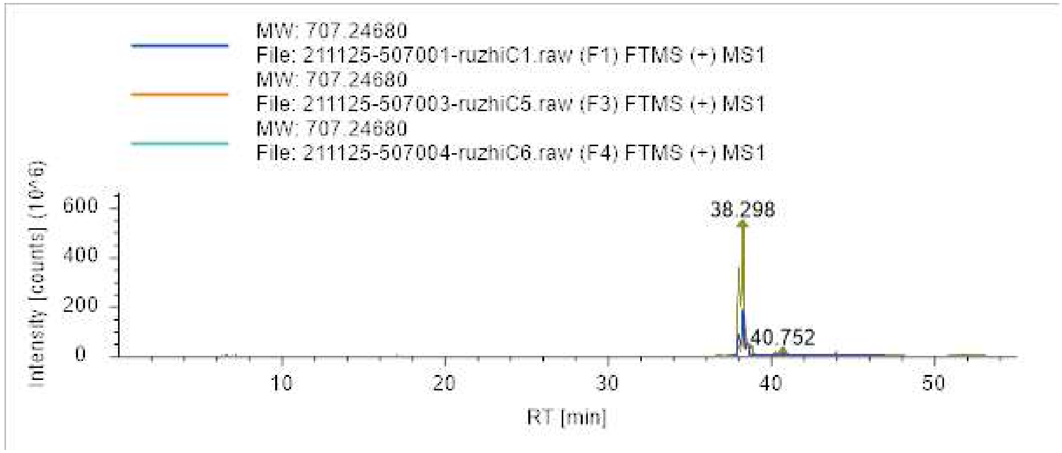

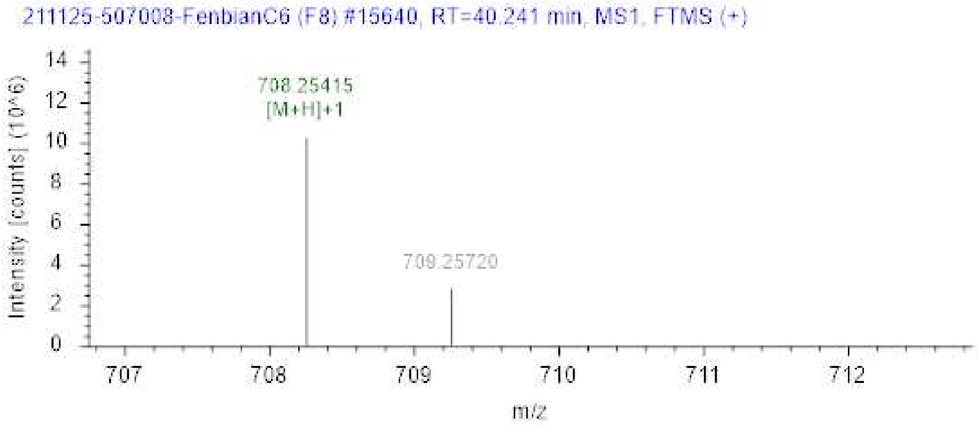

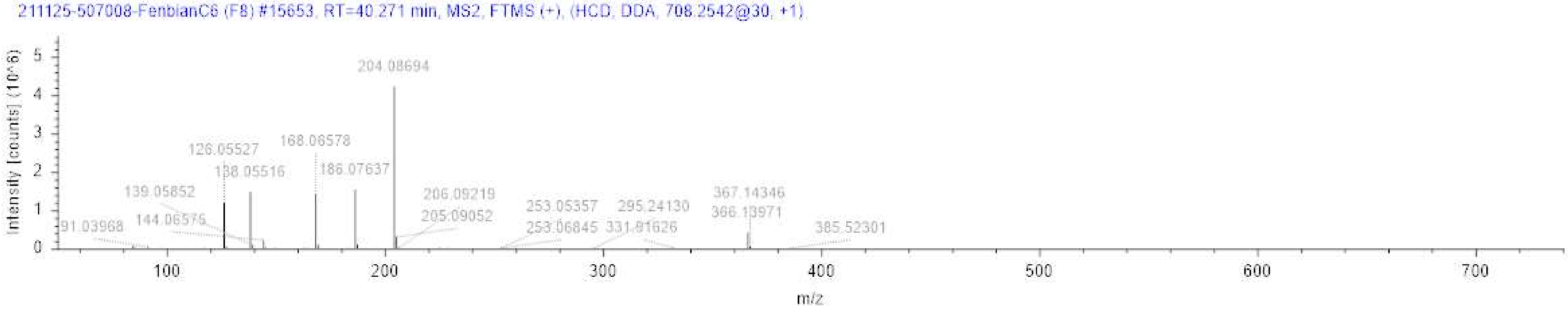

© Reported with Compound Discoverer 3.3

Structure	Name	RT [min]	Formula	Calc. MW	Areas
n/a		43.48	n/a	707.24694	3.48e7

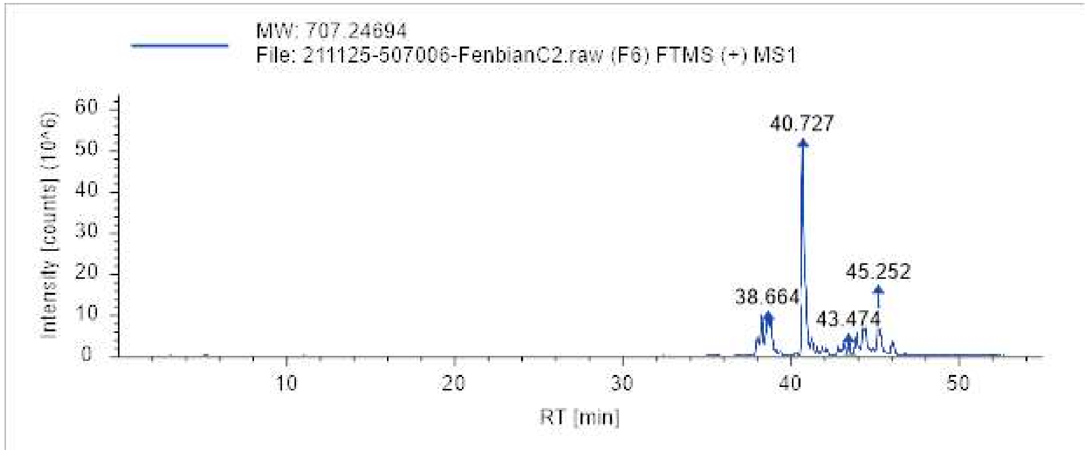

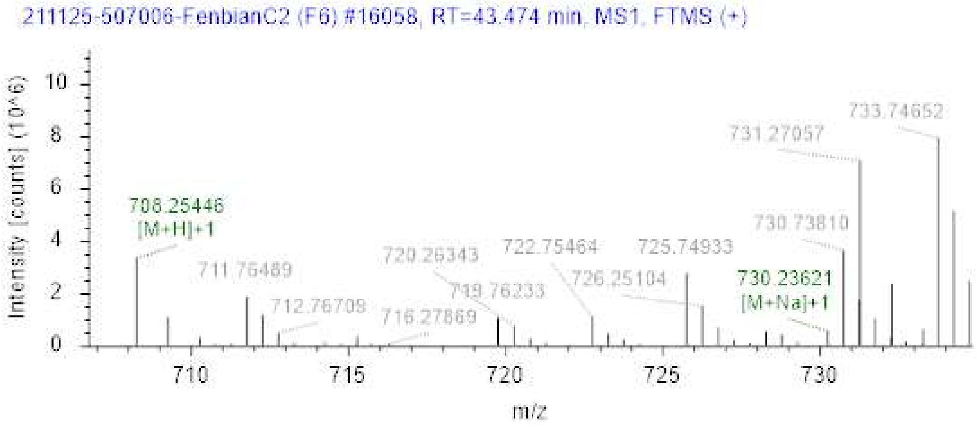

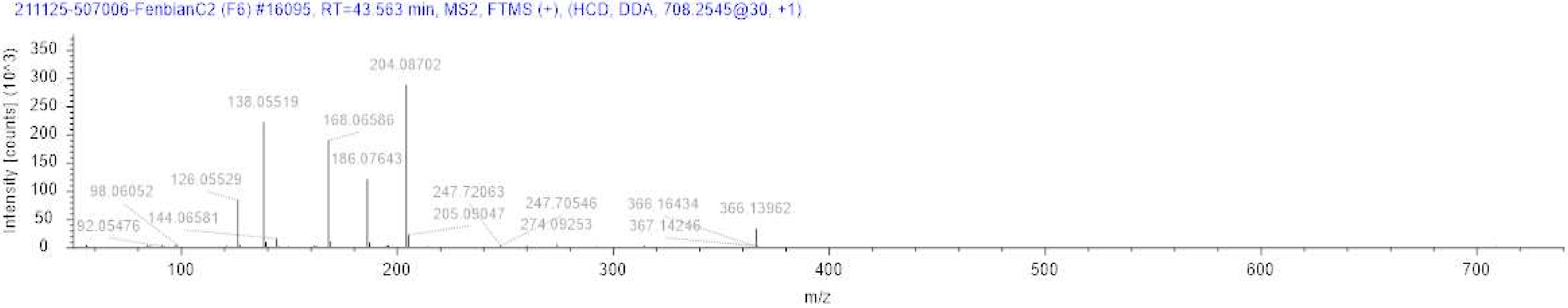

© Reported with Compound Discoverer 3.3

Compounds

Structure	Name	RT [min]	Formula	Calc. MW	Areas
n/a		38.30	n/a	707.24770	9.01e9 1.77e10 5.49e9 5.09e9 1.78e8 2.96e10

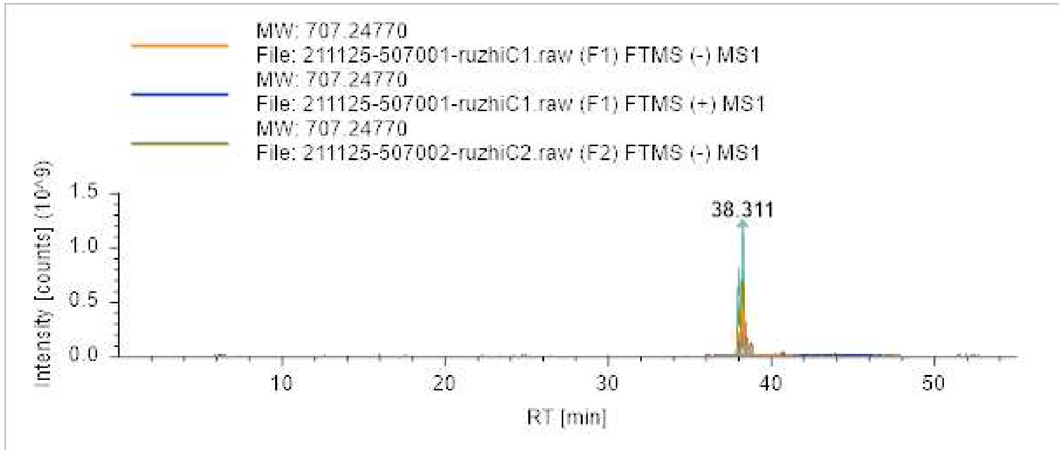

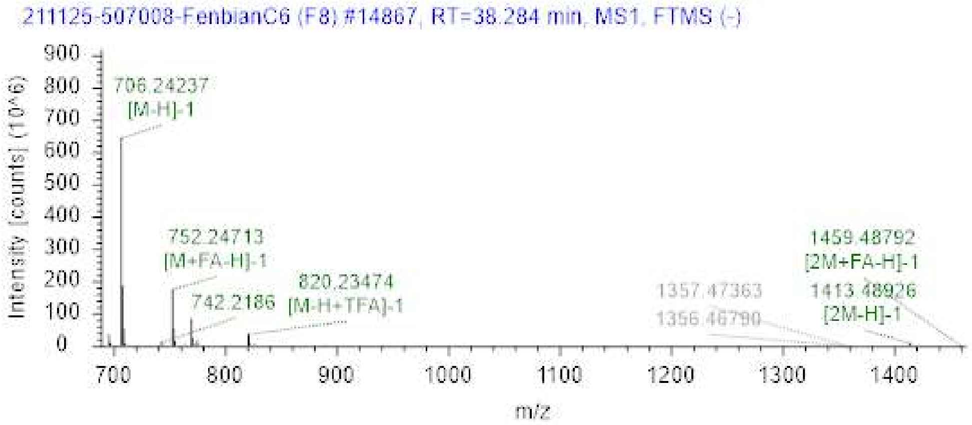

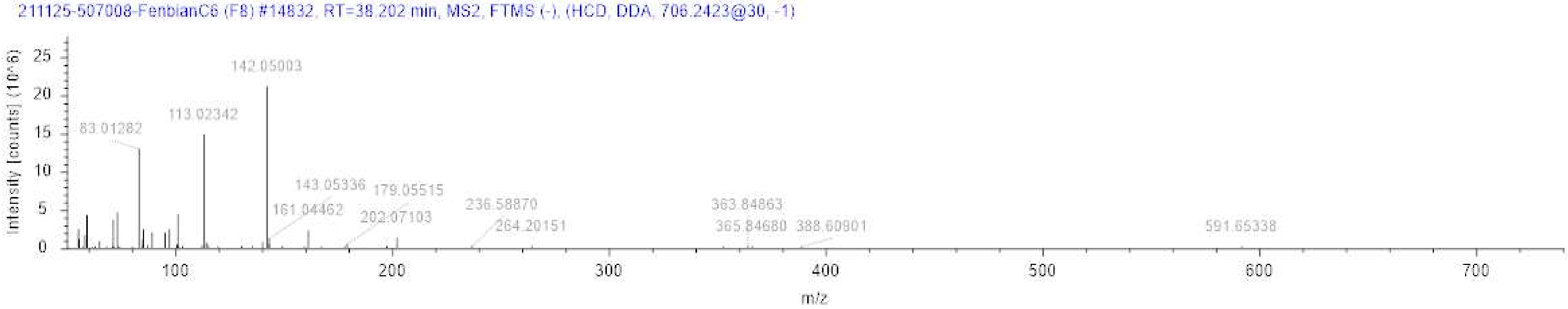

© Reported with Compound Discoverer 3.3

Compounds

Structure	Name	RT [min]	Formula	Calc. MW	Areas
n/a		29.64	n/a	709.26448	1.27e7

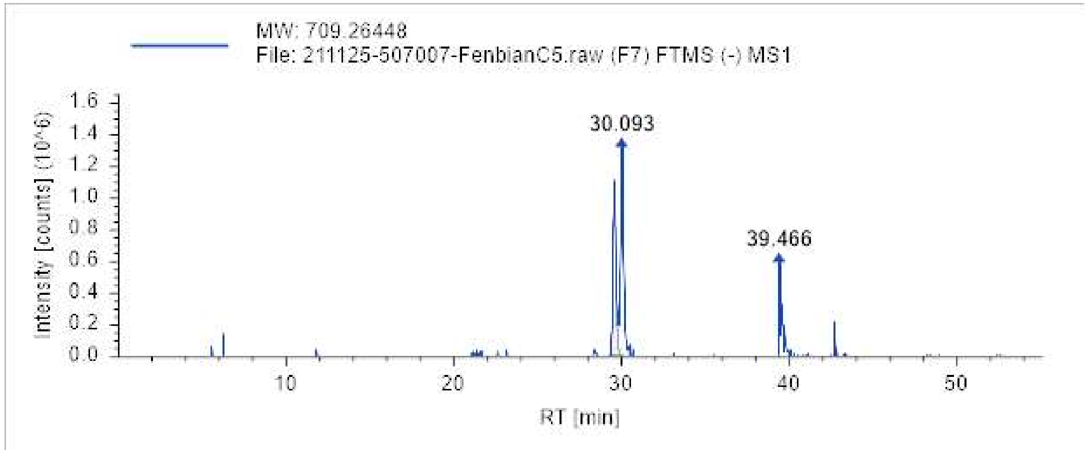

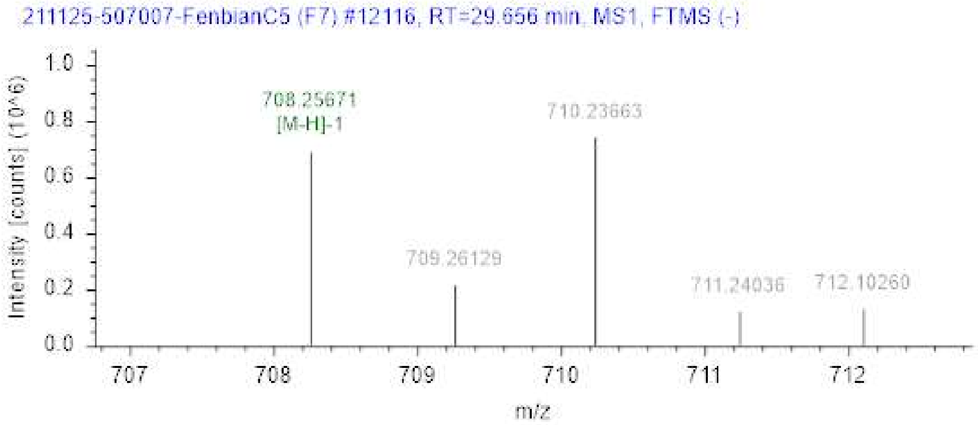

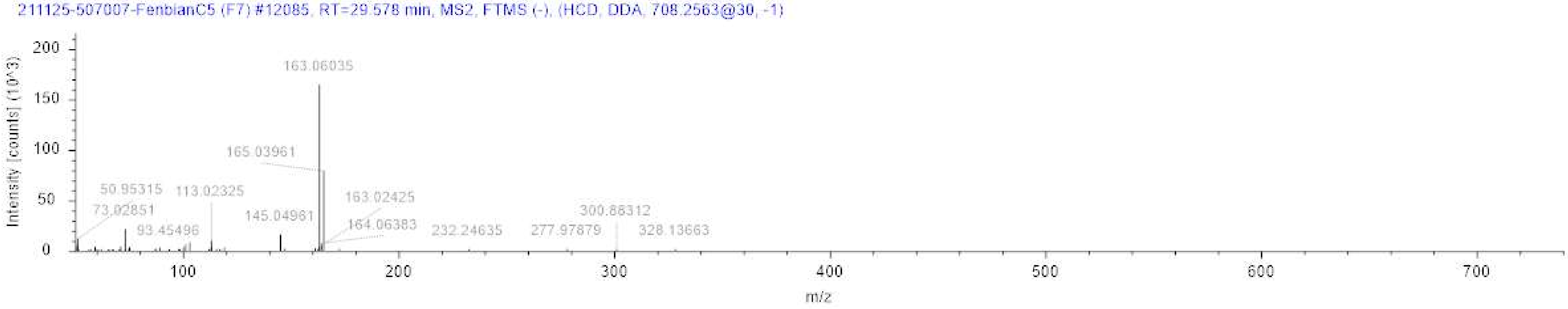

© Reported with Compound Discoverer 3.3

Compounds

Structure	Name	RT [min]	Formula	Calc. MW	Areas
n/a		30.12	n/a	709.26448	1.41e7

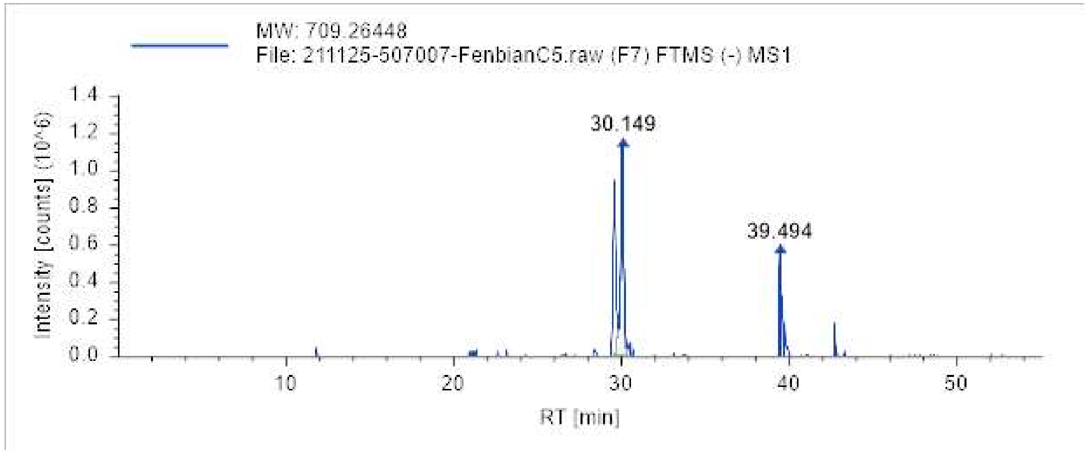

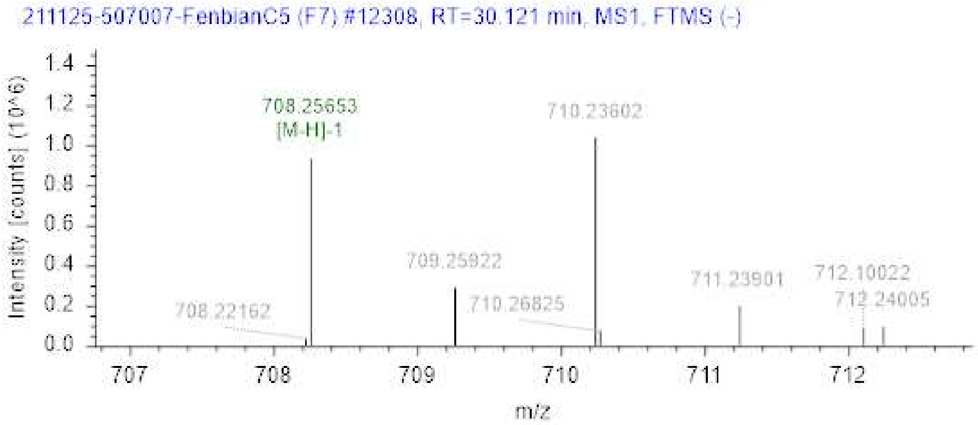

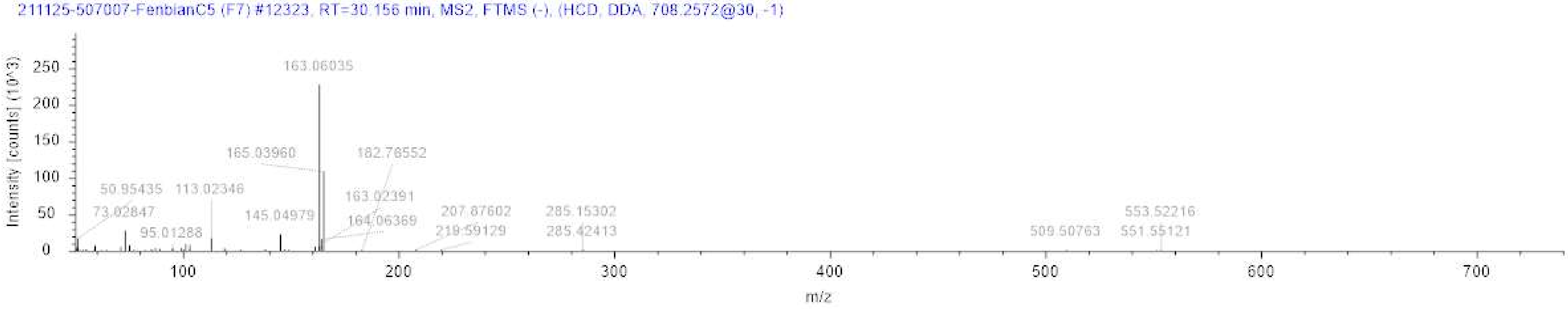

© Reported with Compound Discoverer 3.3

Compounds

Structure	Name	RT [min]	Formula	Calc. MW	Areas
n/a		40.65	n/a	732.27949	9.88e6

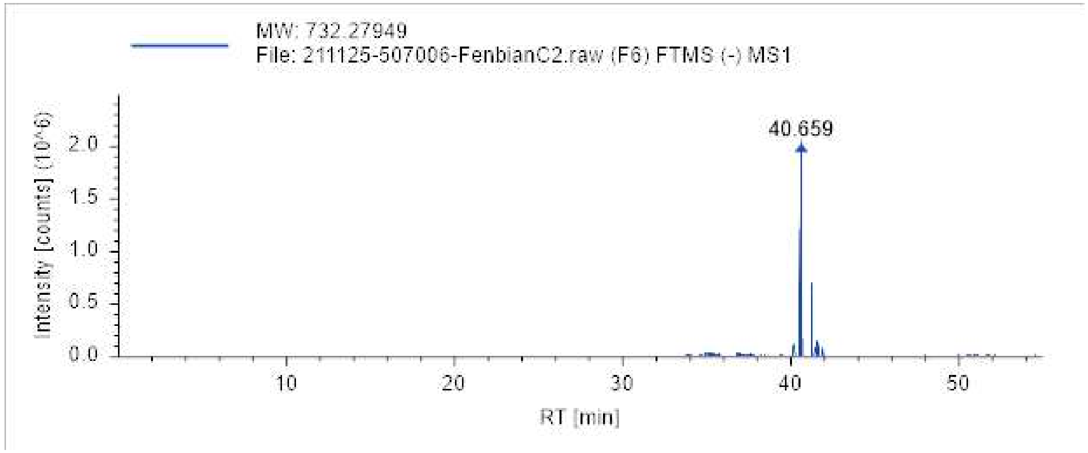

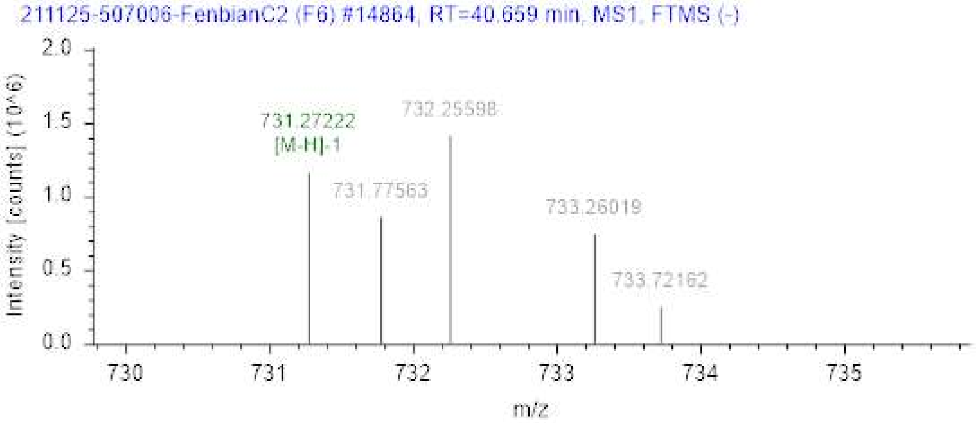

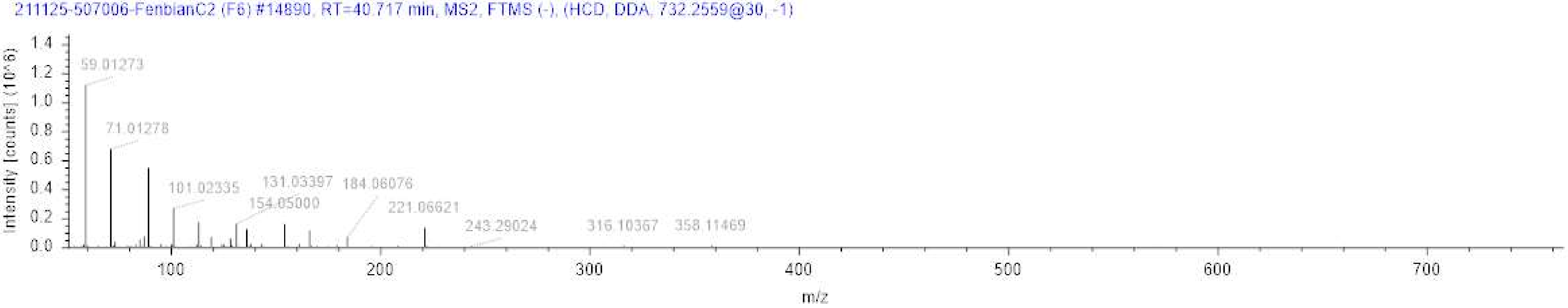

© Reported with Compound Discoverer 3.3

Compounds

Structure	Name	RT [min]	Formula	Calc. MW	Areas
n/a		40.17	n/a	748.27416	9.94e6

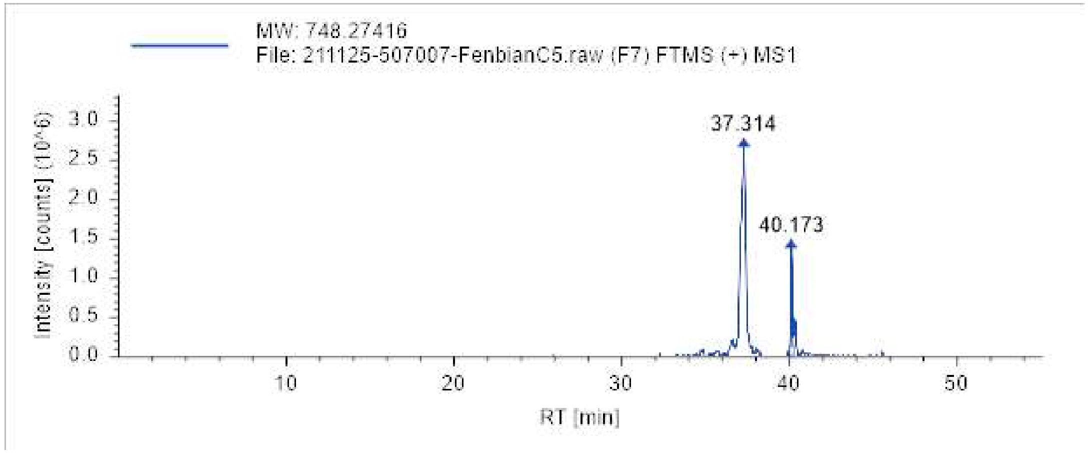

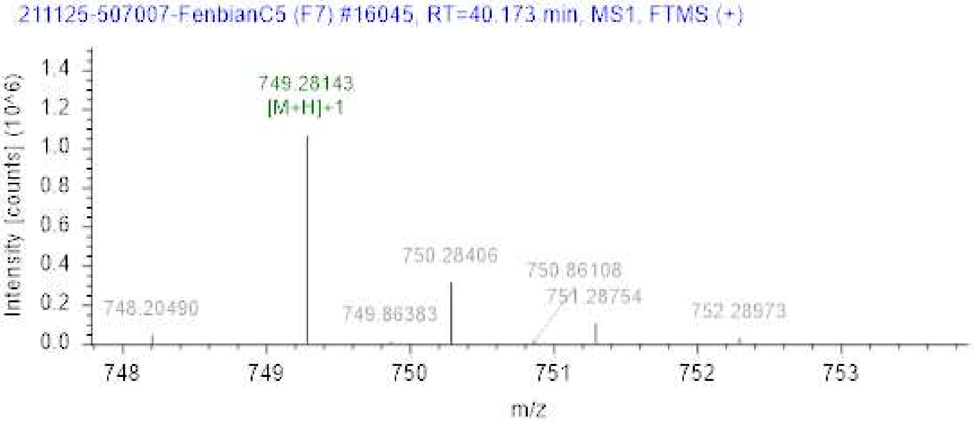

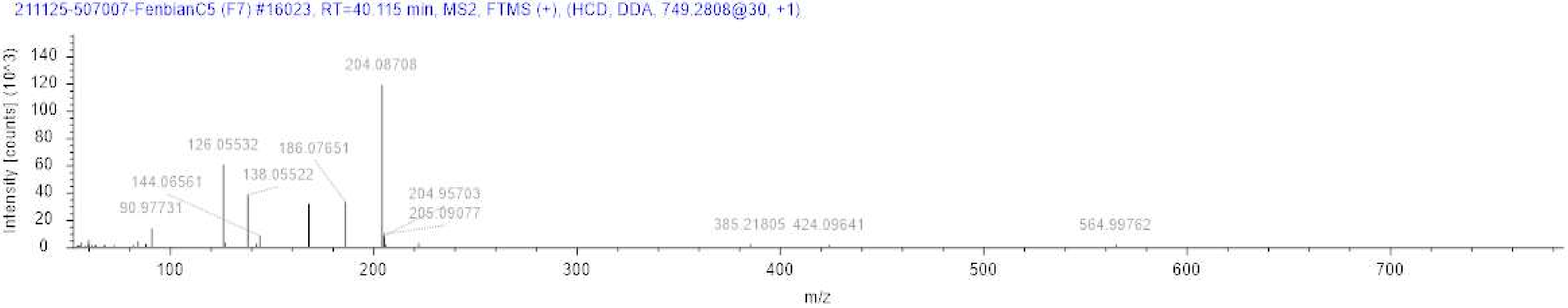

© Reported with Compound Discoverer 3.3

Compounds

Structure	Name	RT [min]	Formula	Calc. MW	Areas
n/a		37.26	n/a	748.27496	4.49e8 8.06e7 1.49e8

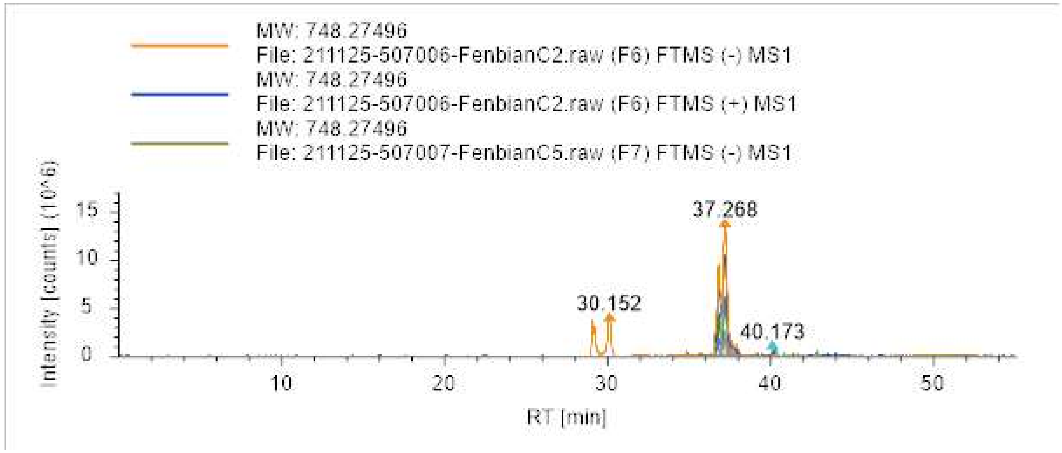

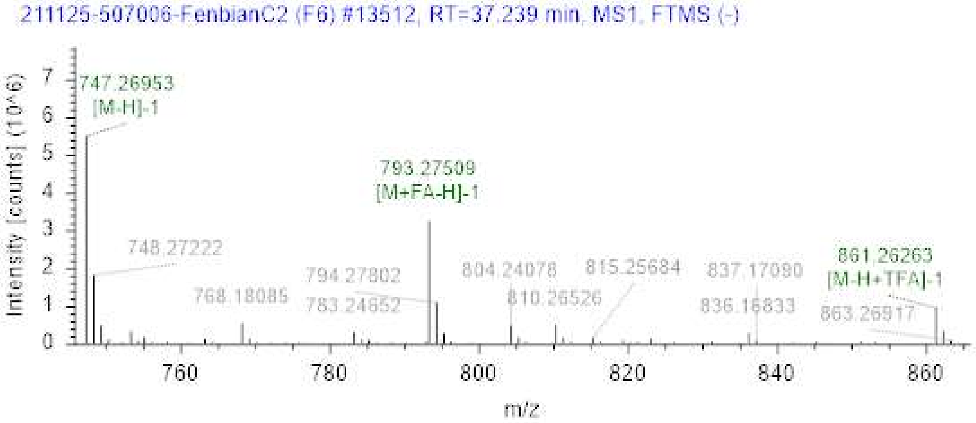

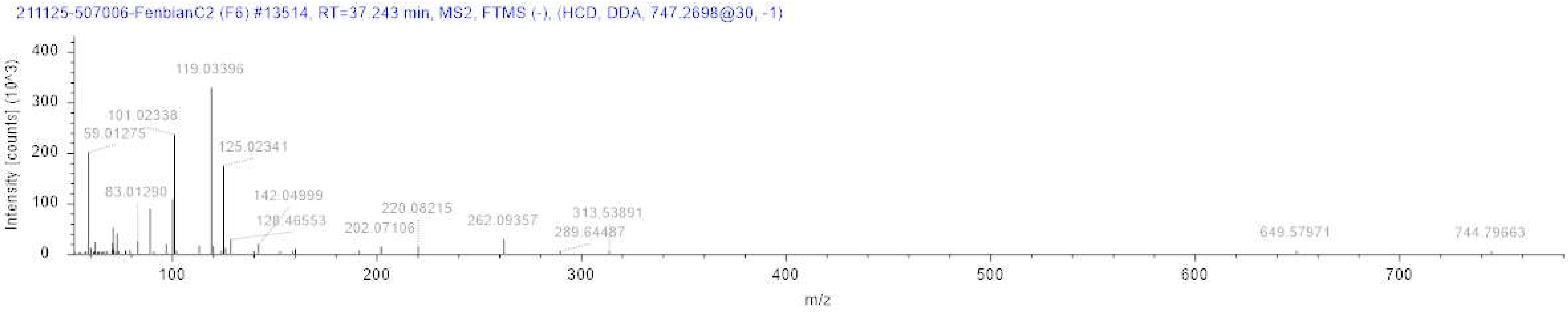

© Reported with Compound Discoverer 3.3

Structure	Name	RT [min]	Formula	Calc. MW	Areas
n/a		38.94	n/a	779.27001	1.33e7 6.90e7 4.38e7 2.33e7 1.07e9 1.42e8

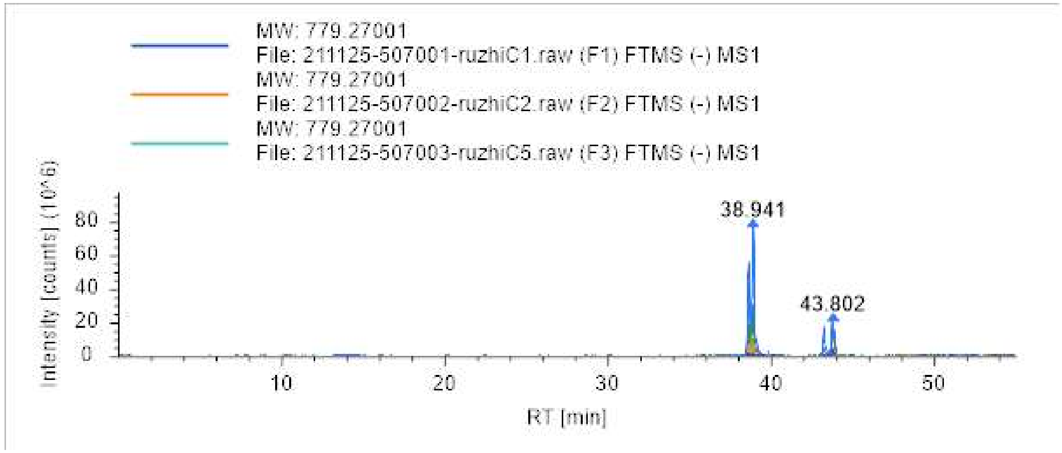

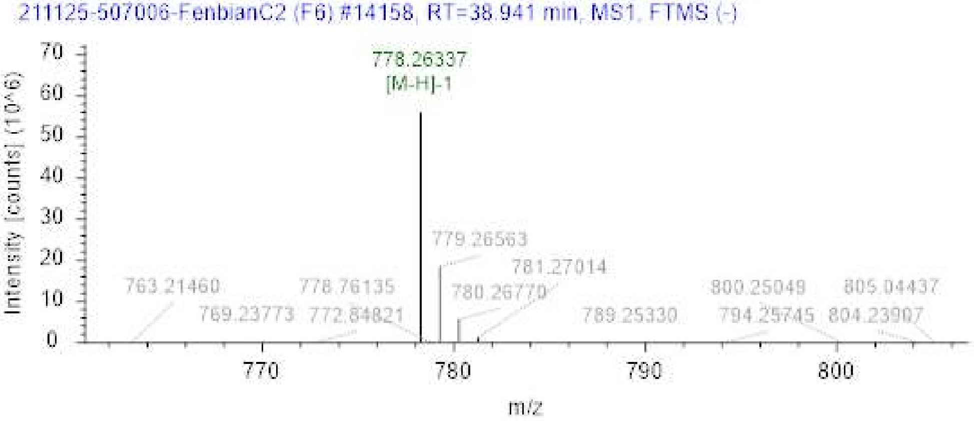

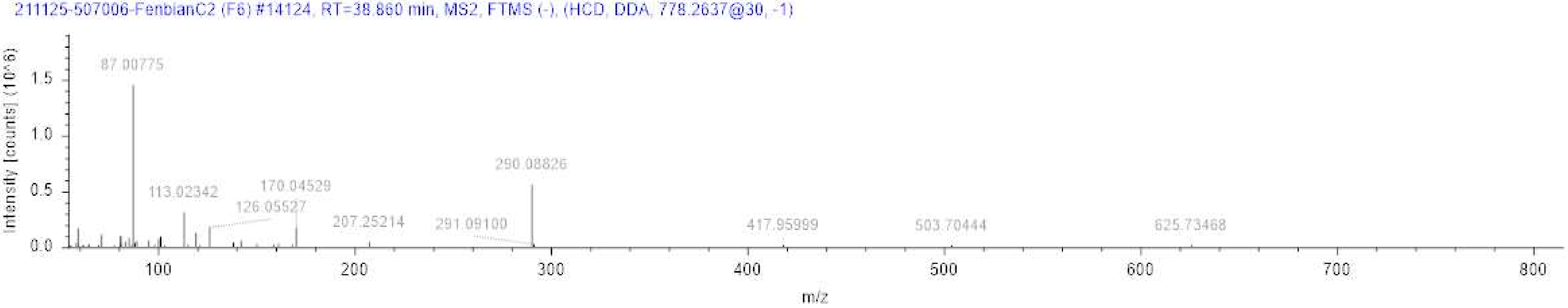

© Reported with Compound Discoverer 3.3

Structure	Name	RT [min]	Formula	Calc. MW	Areas
n/a		34.90	n/a	780.28661	9.01e6

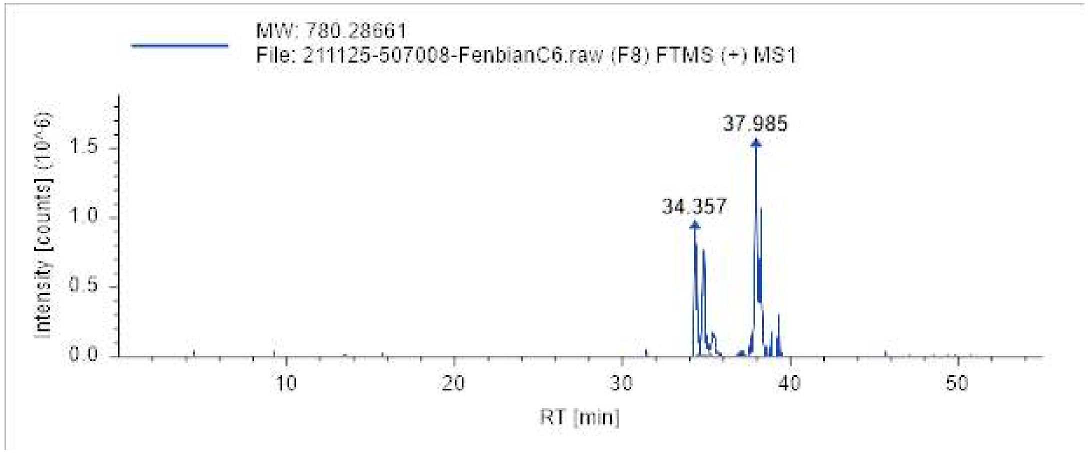

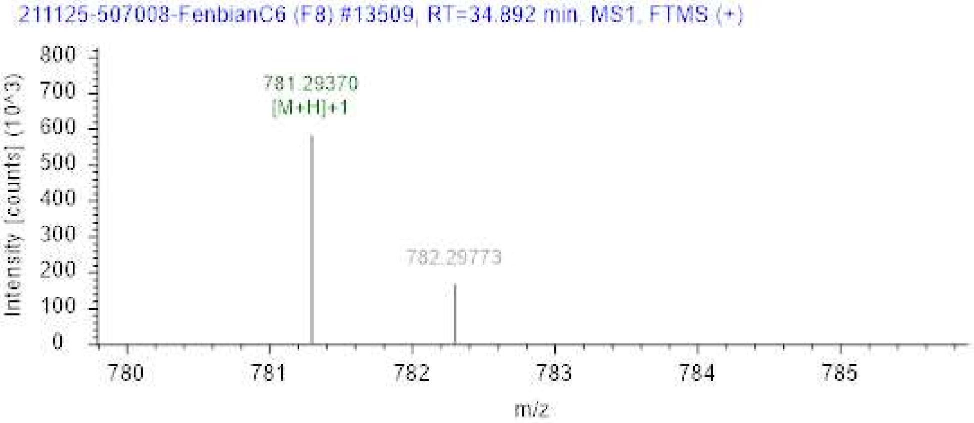

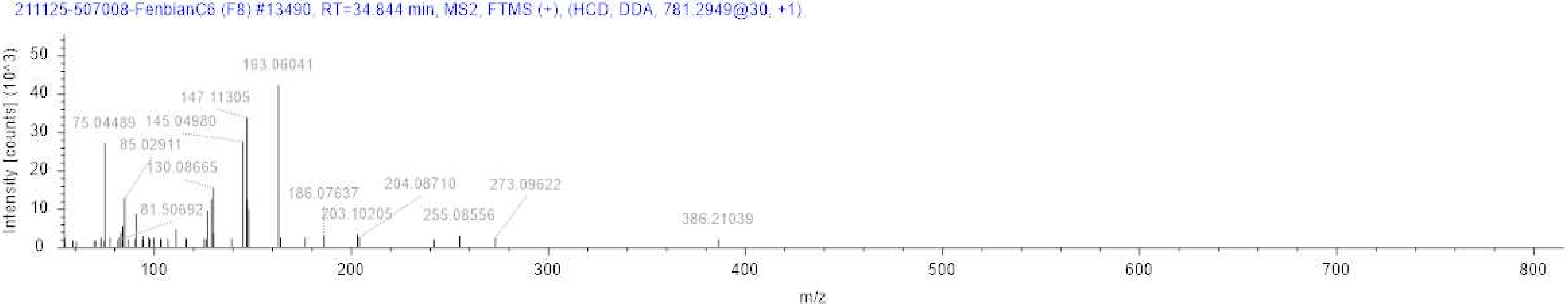

© Reported with Compound Discoverer 3.3

Compounds

Structure	Name	RT [min]	Formula	Calc. MW	Areas
n/a		39.30	n/a	780.28679	7.30e6

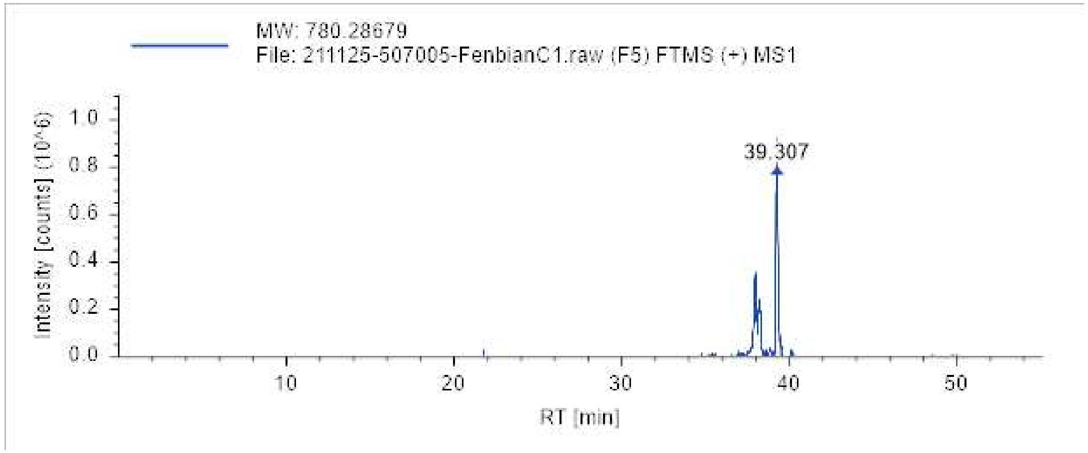

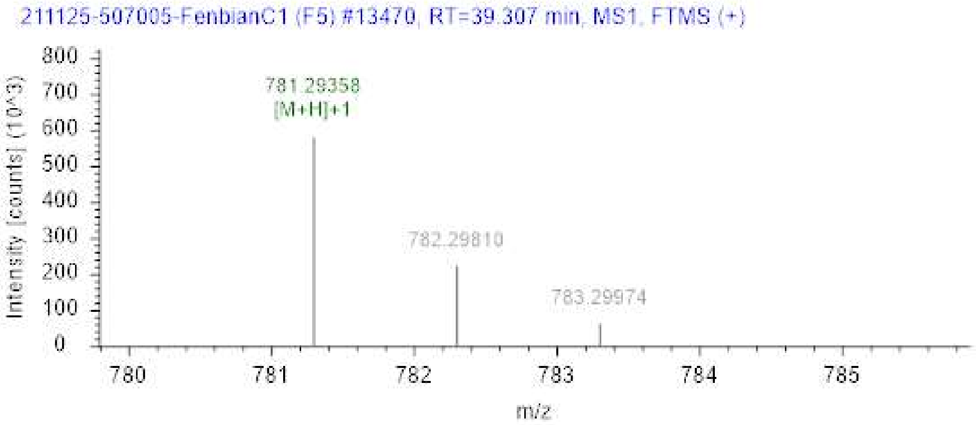

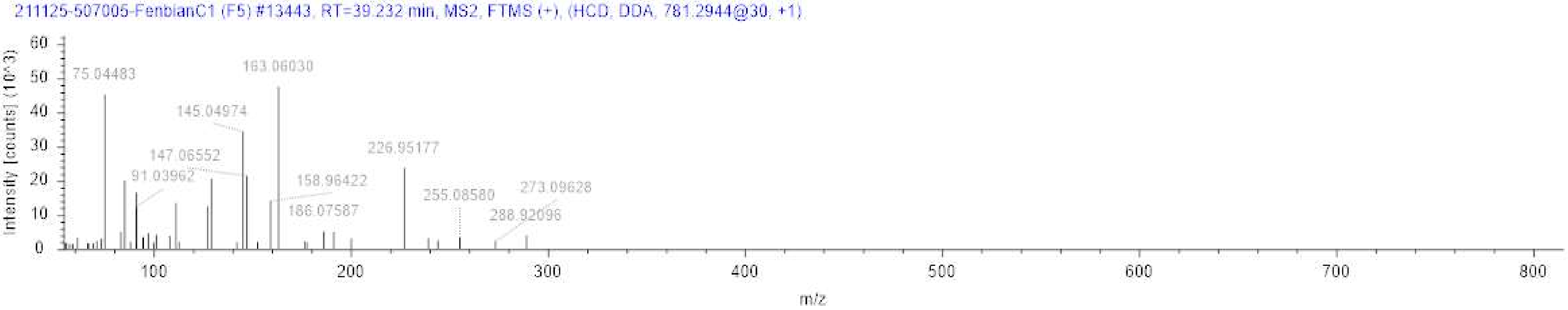

© Reported with Compound Discoverer 3.3

Structure	Name	RT [min]	Formula	Calc. MW	Areas
n/a		37.99	n/a	780.28833	6.39e6; 2.38e8

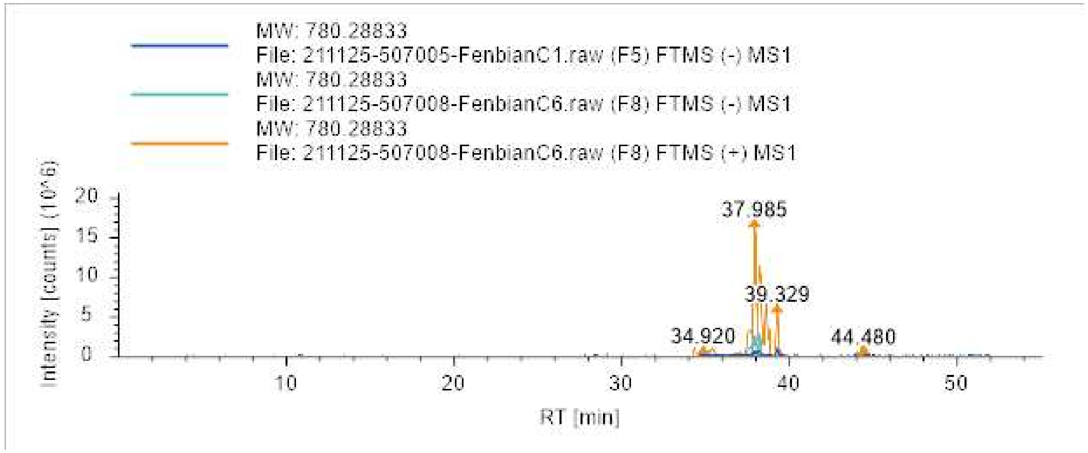

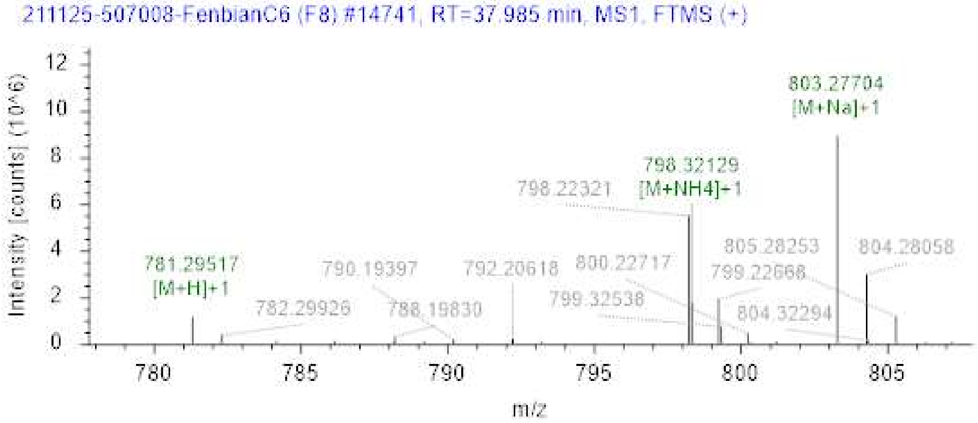

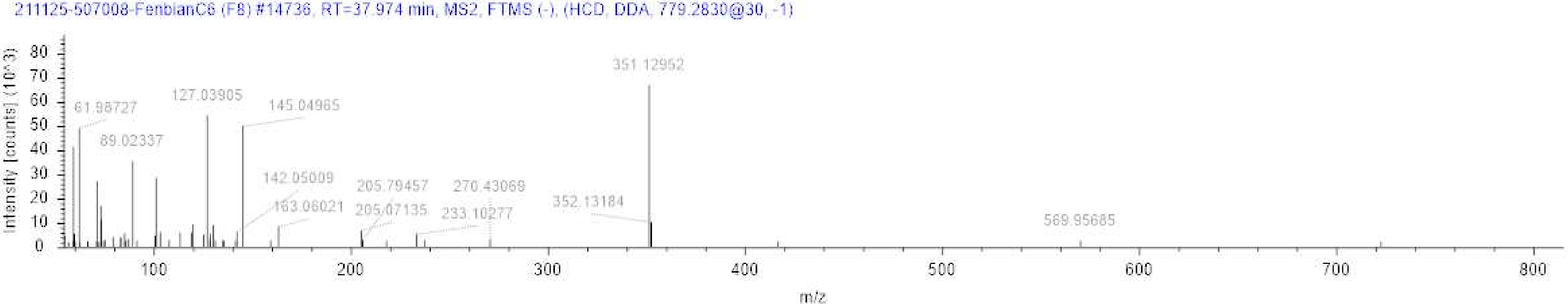

© Reported with Compound Discoverer 3.3

Compounds

Structure	Name	RT [min]	Formula	Calc. MW	Areas
n/a		34.91	n/a	789.30177	4.94e7

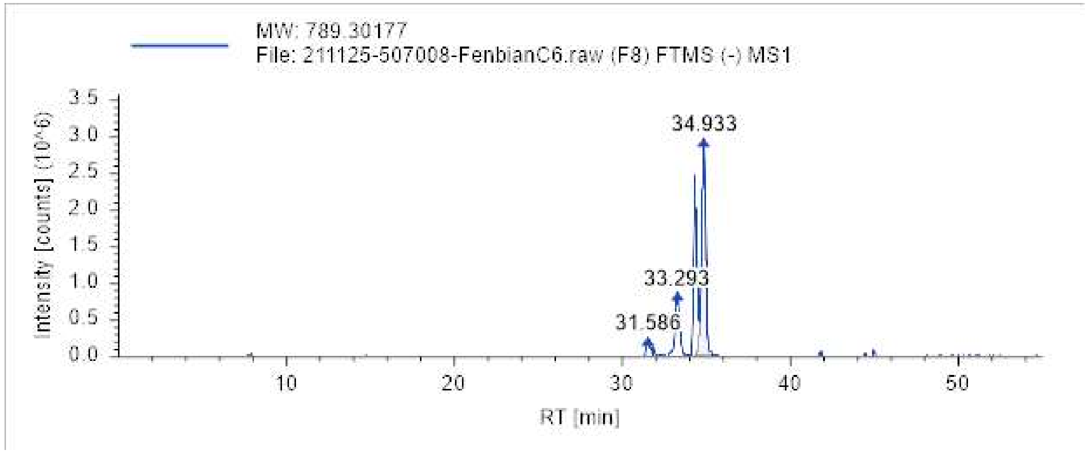

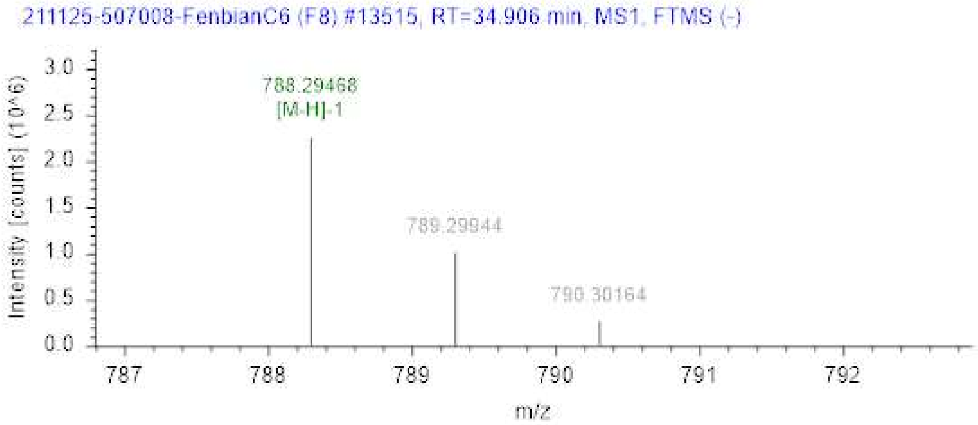

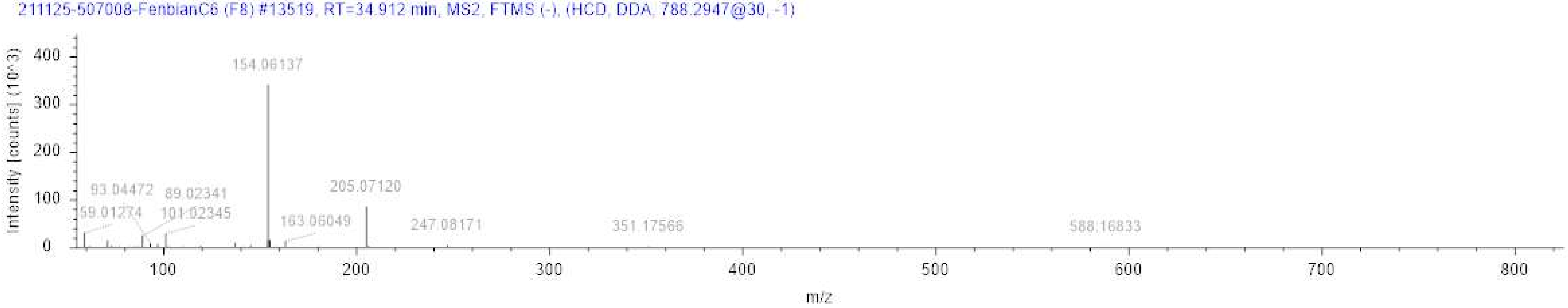

© Reported with Compound Discoverer 3.3

Structure	Name	RT [min]	Formula	Calc. MW	Areas
n/a		33.30	n/a	789.30232	1.93e7

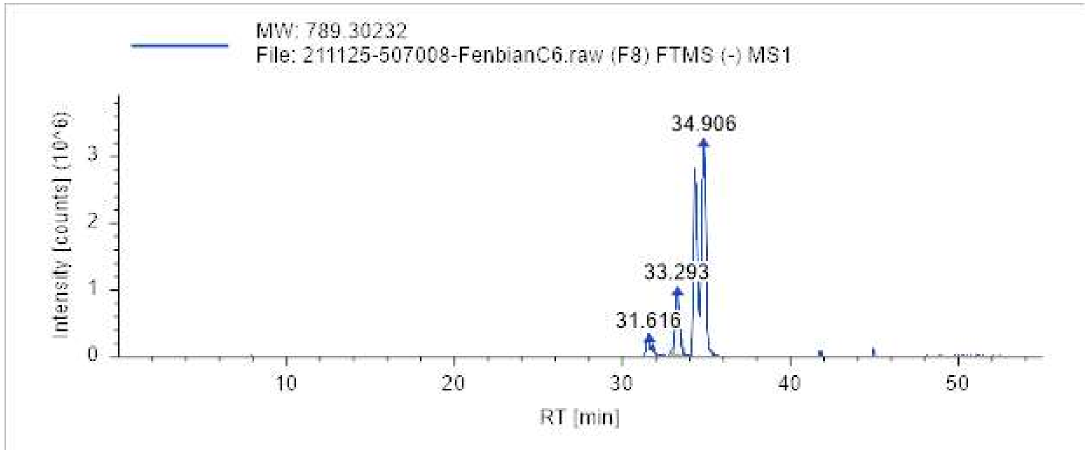

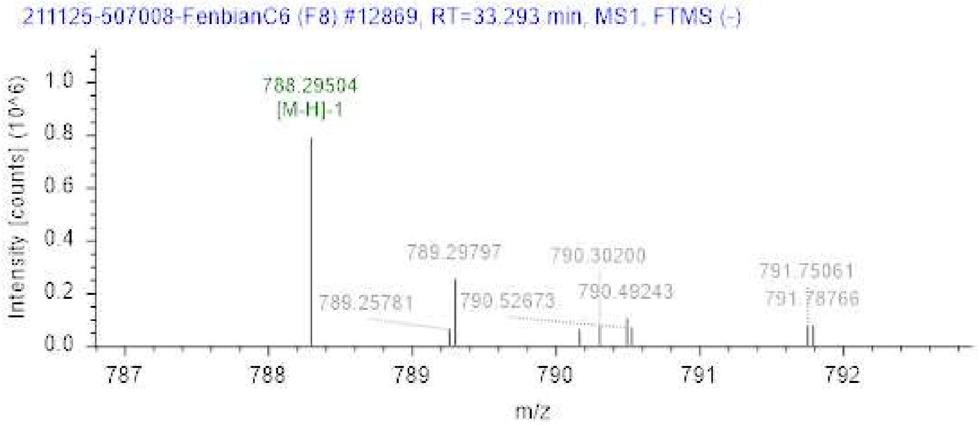

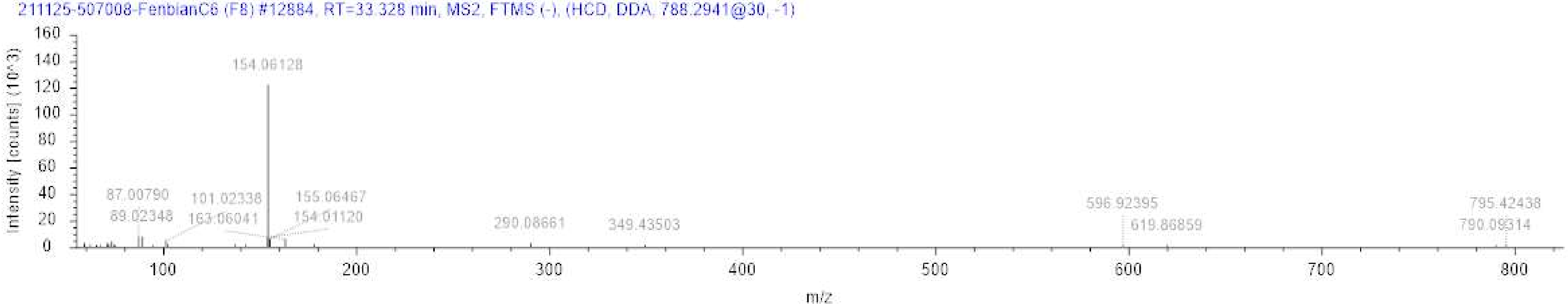

© Reported with Compound Discoverer 3.3

Compounds

Structure	Name	RT [min]	Formula	Calc. MW	Areas
n/a		40.15	n/a	795.26426	7.24e7 9.09e7

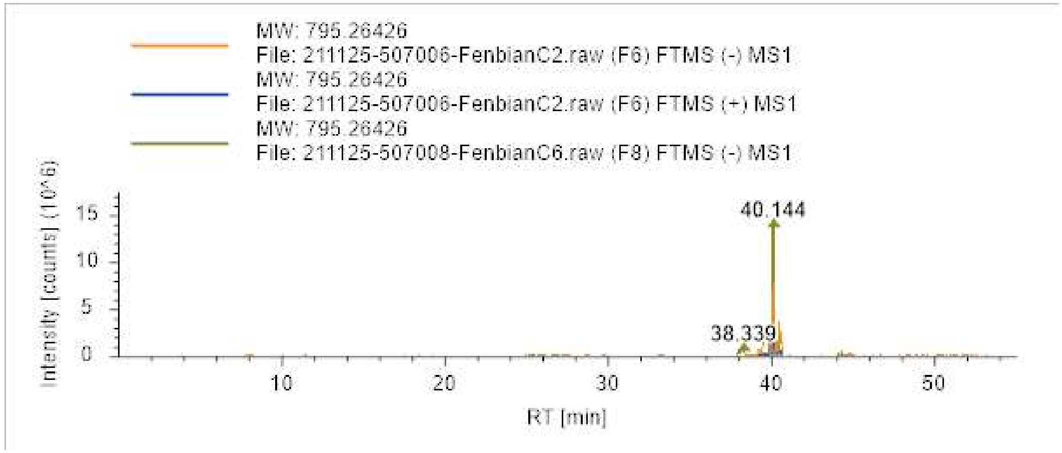

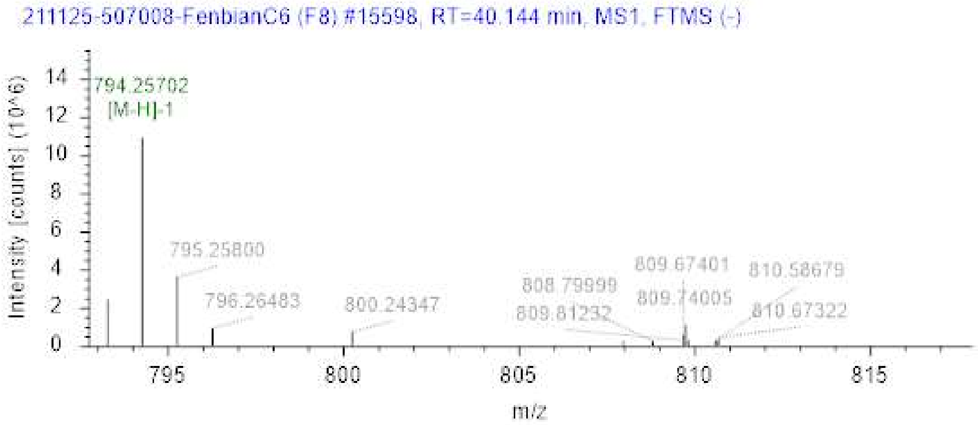

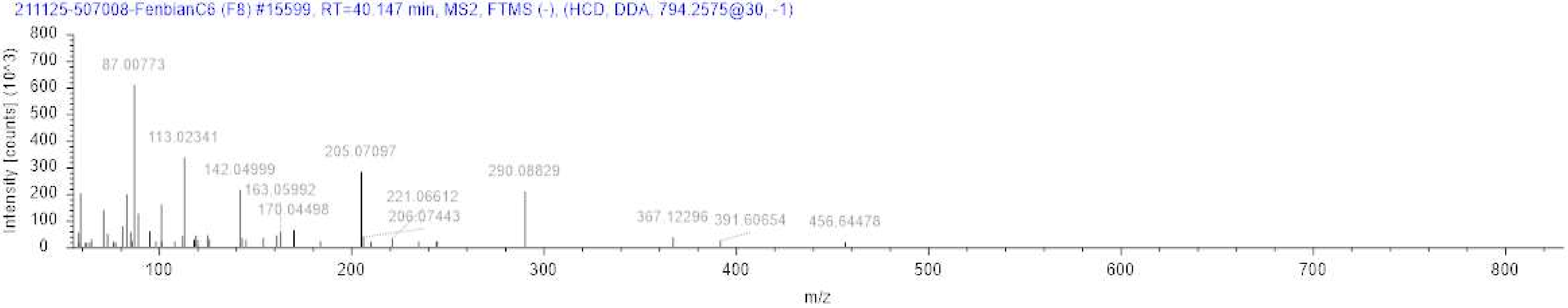

© Reported with Compound Discoverer 3.3

Compounds

Structure	Name	RT [min]	Formula	Calc. MW	Areas
n/a		40.66	n/a	796.28482	2.98e7 · 2.78e7 · 1.13e8 · 4.63e7 · 1.46e9

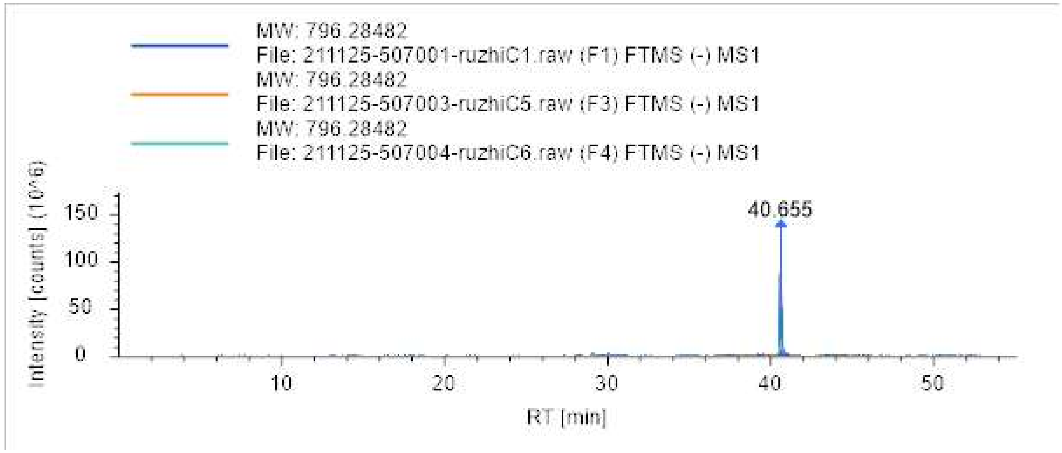

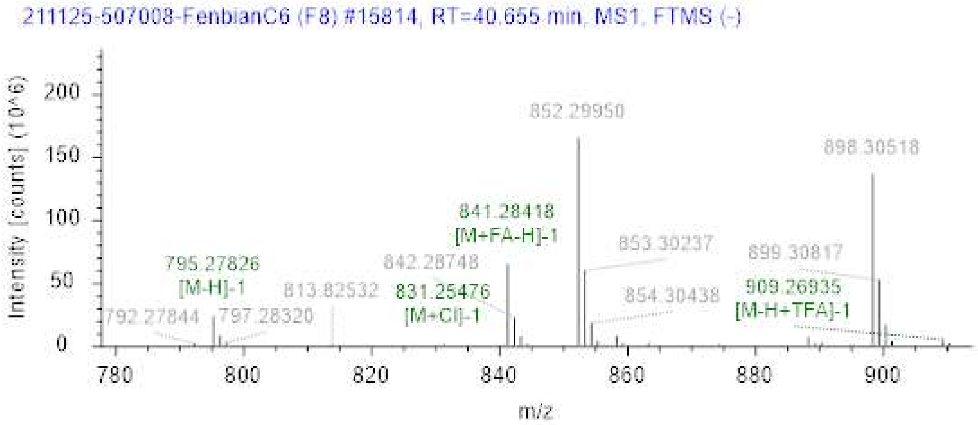

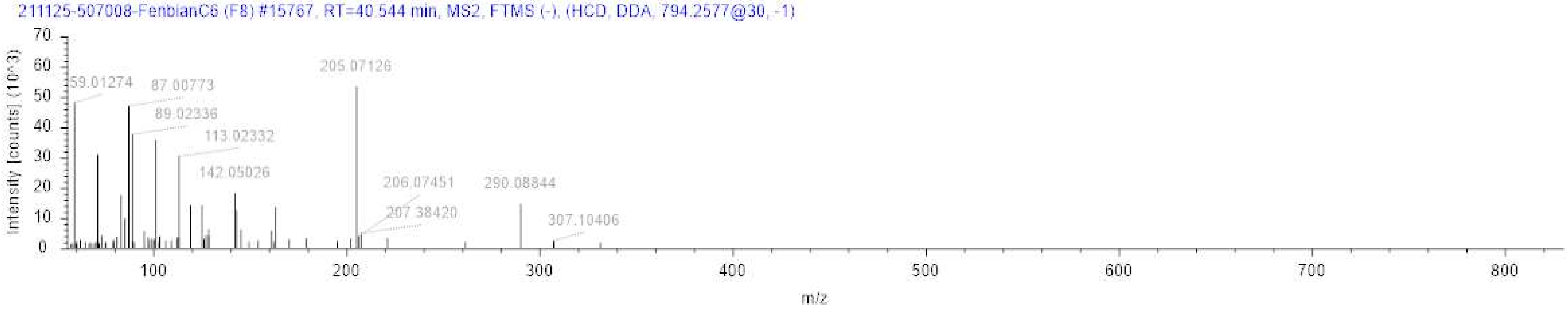

© Reported with Compound Discoverer 3.3

Compounds

Structure	Name	RT [min]	Formula	Calc. MW	Areas
n/a		39.26	n/a	796.28810	1.50e7

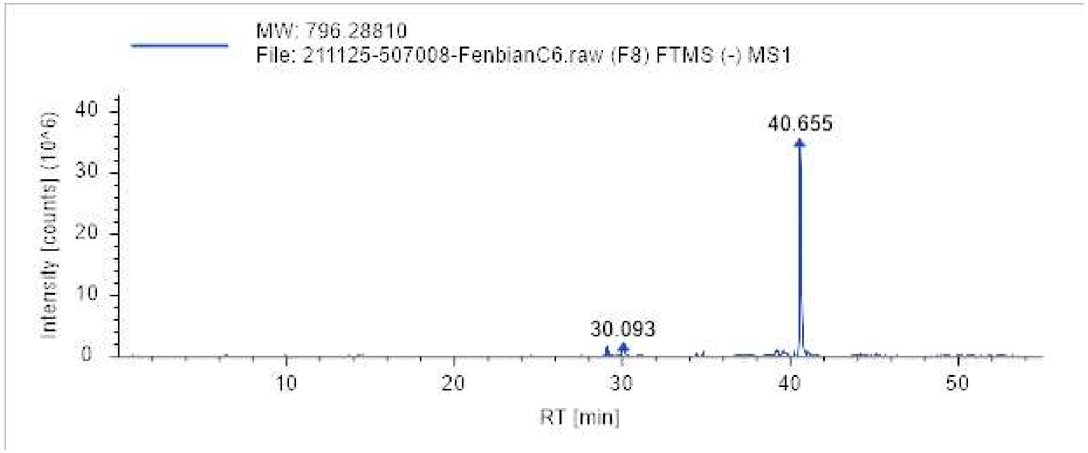

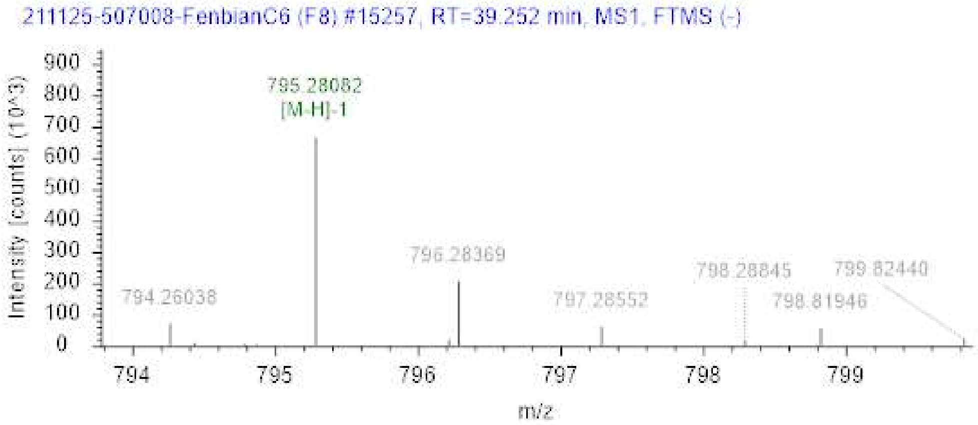

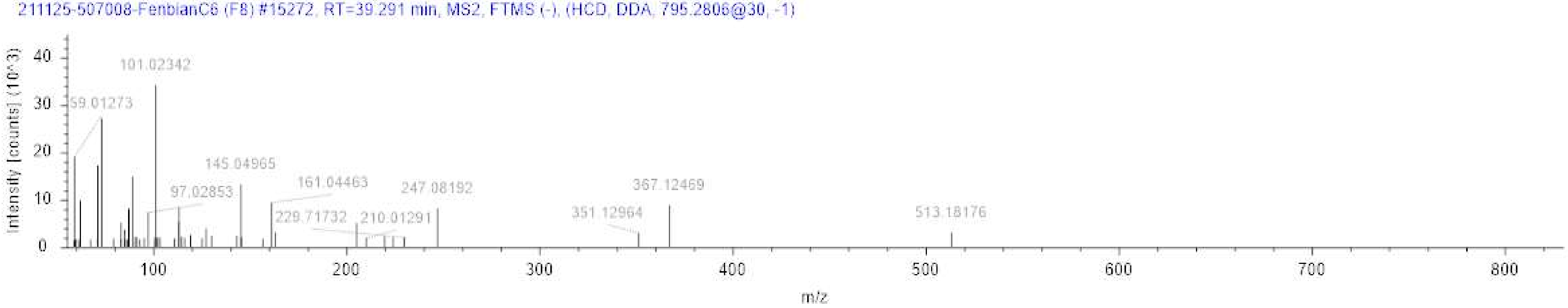

© Reported with Compound Discoverer 3.3

Compounds

Structure	Name	RT [min]	Formula	Calc. MW	Areas
n/a		38.30	n/a	797.28065	2.12e7

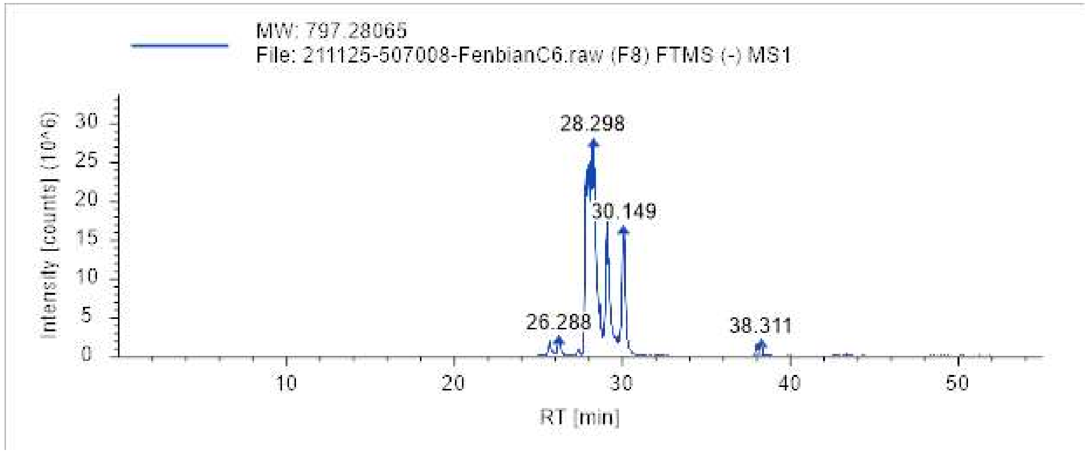

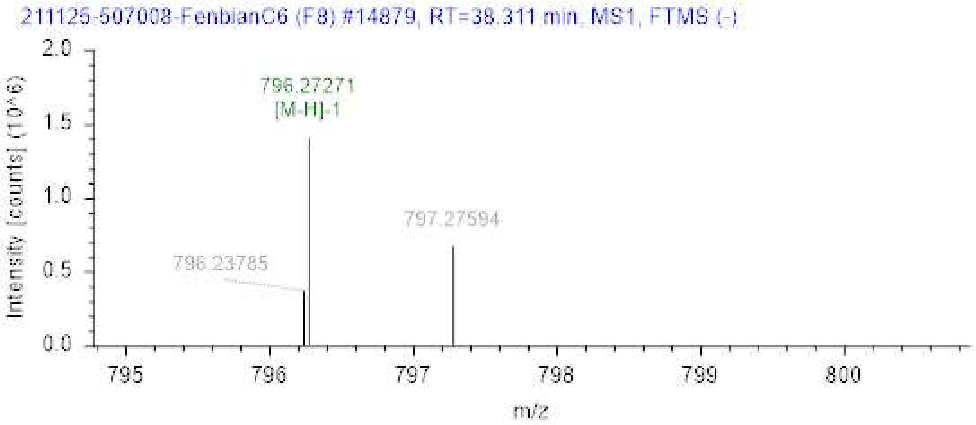

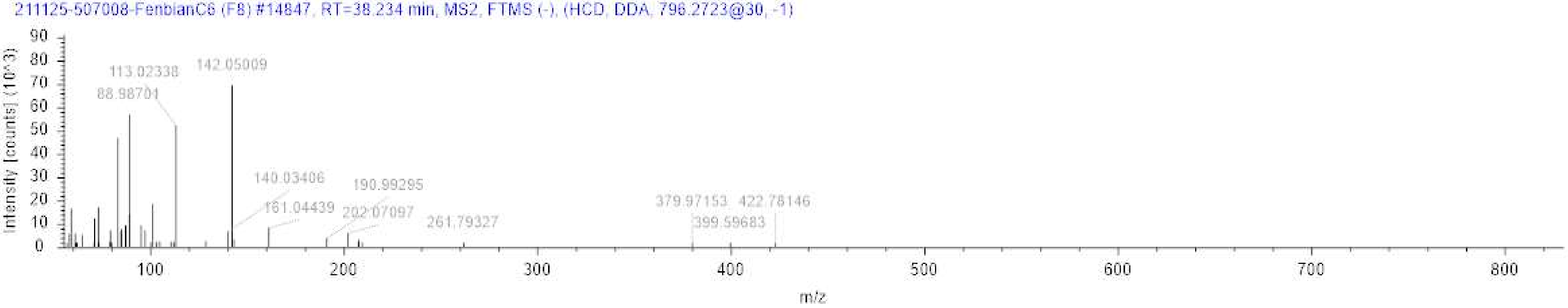

© Reported with Compound Discoverer 3.3

Compounds

Structure	Name	RT [min]	Formula	Calc. MW	Areas
n/a		25.73	n/a	797.28108	2.90e7

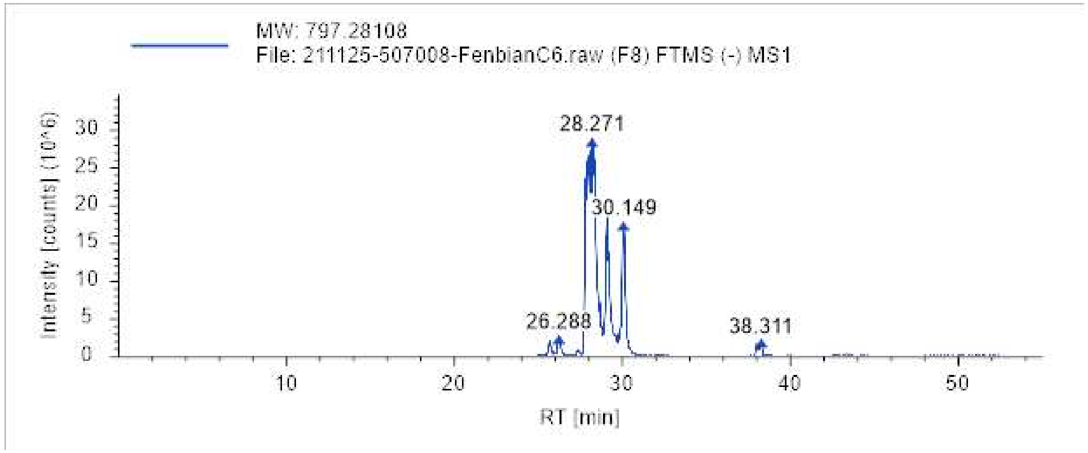

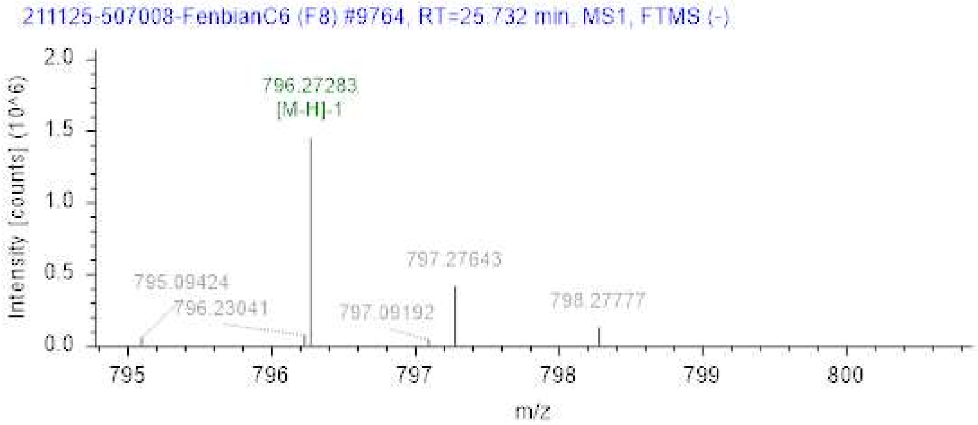

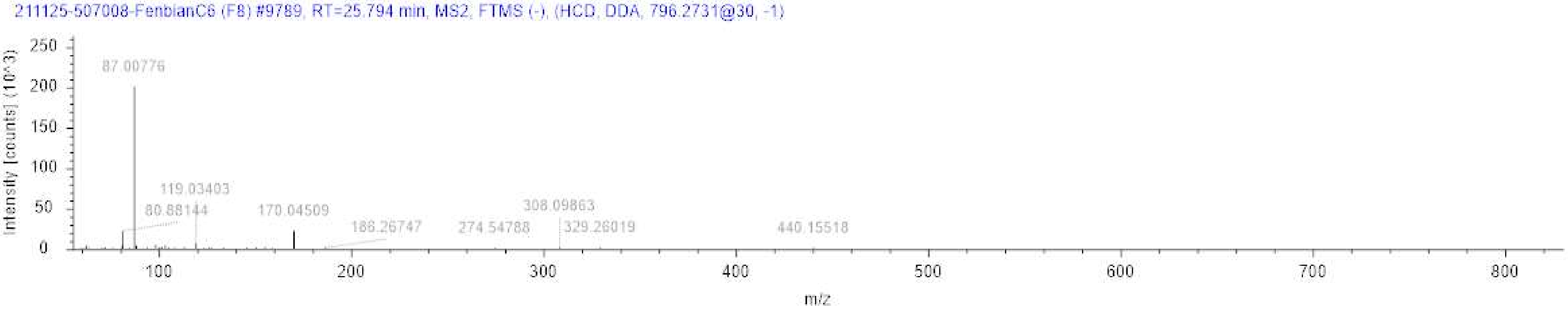

© Reported with Compound Discoverer 3.3

Compounds

Structure	Name	RT [min]	Formula	Calc. MW	Areas
n/a		30.14	n/a	797.28177	1.31e8; 2.65e8

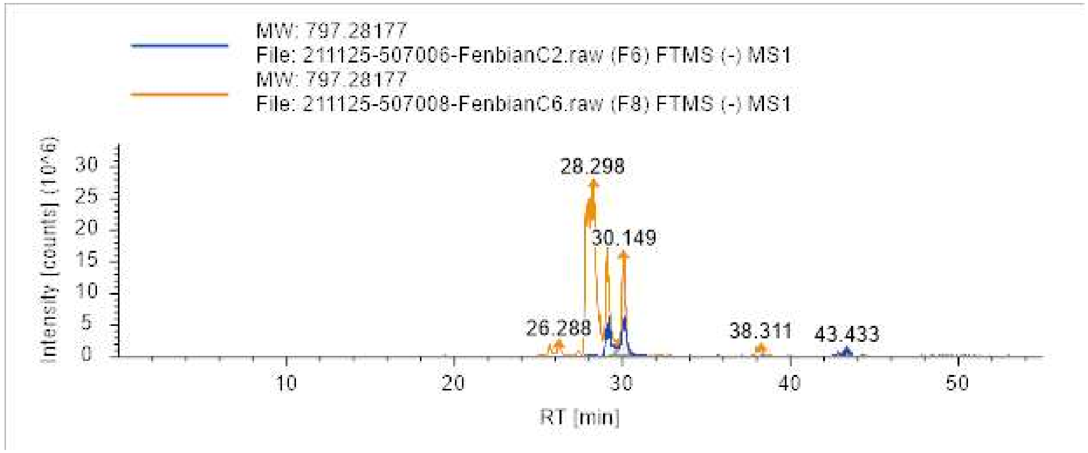

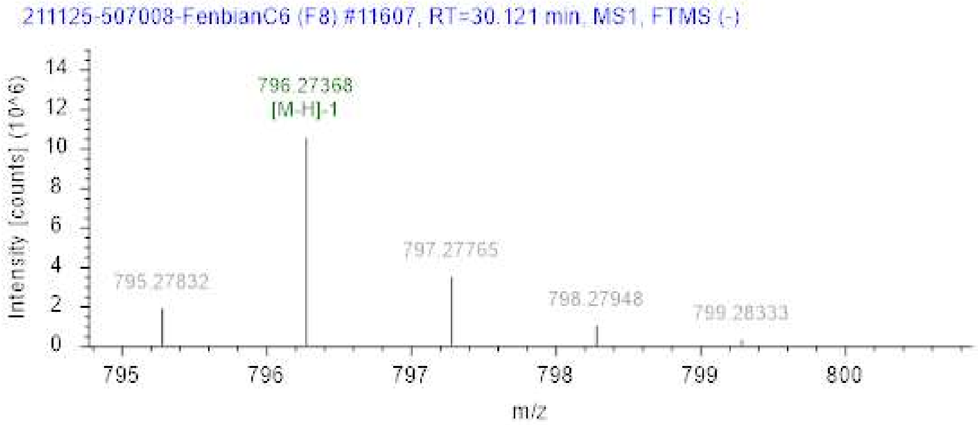

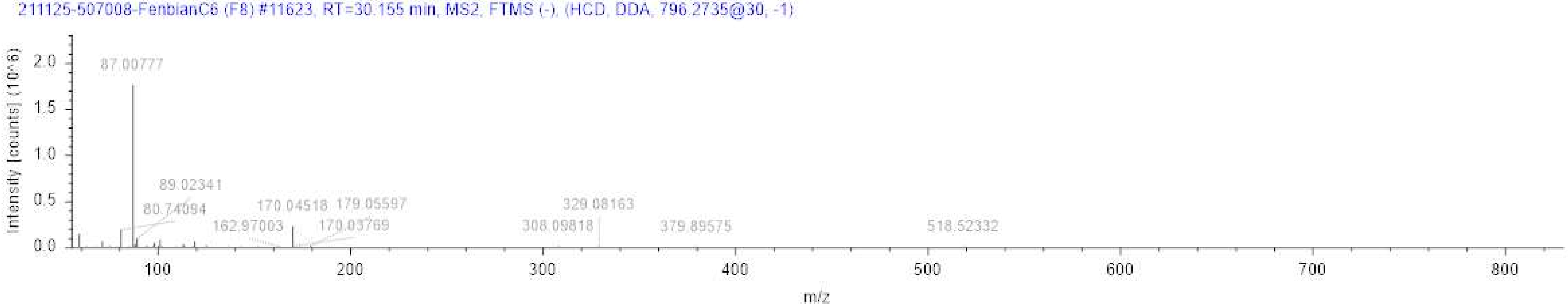

© Reported with Compound Discoverer 3.3

Compounds

Structure	Name	RT [min]	Formula	Calc. MW	Areas
n/a		42.20	n/a	836.28863	2.18e7

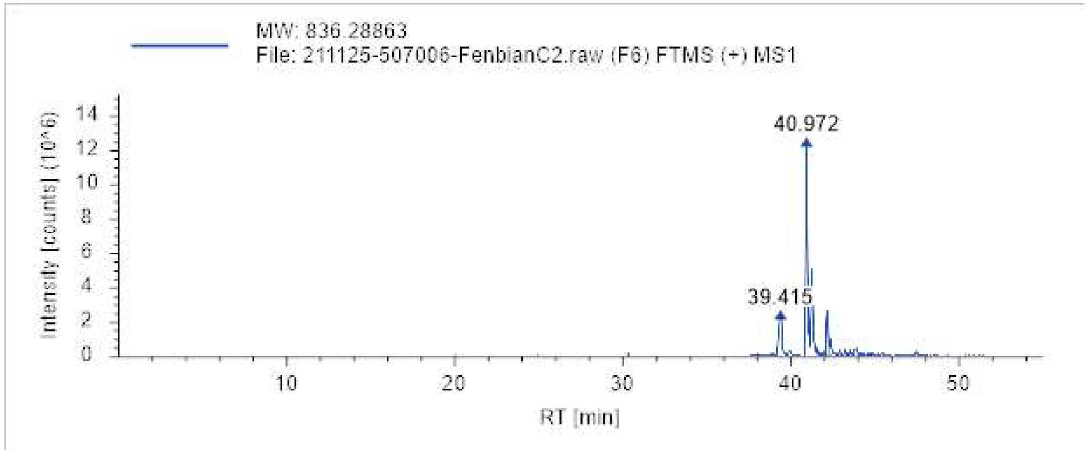

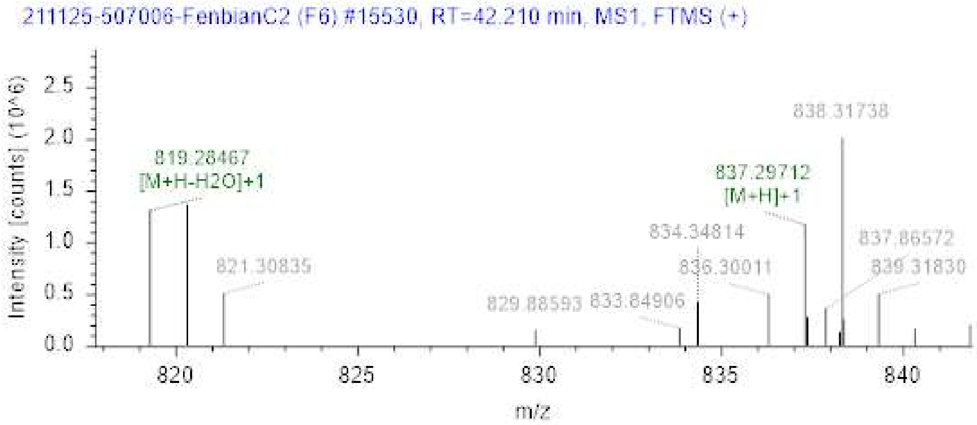

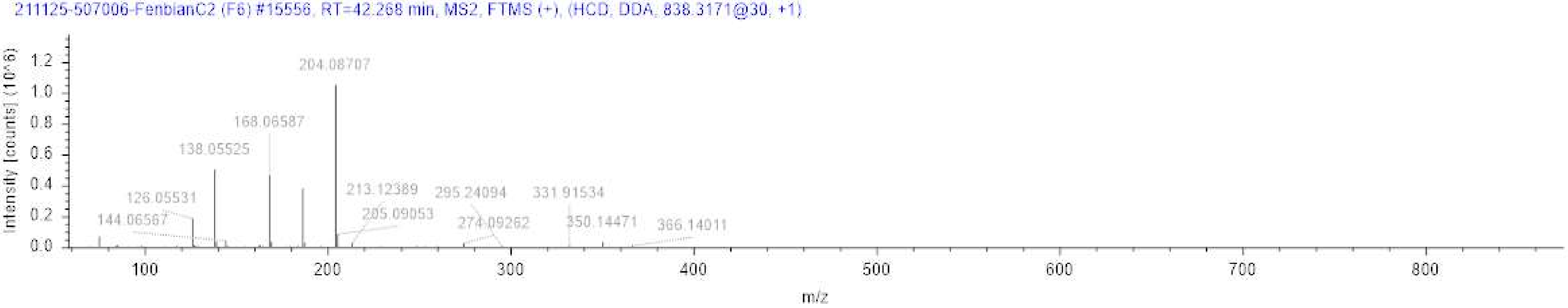

© Reported with Compound Discoverer 3.3

Structure	Name	RT [min]	Formula	Calc. MW	Areas
n/a		39.40	n/a	836.29242	2.80e8 2.04e8

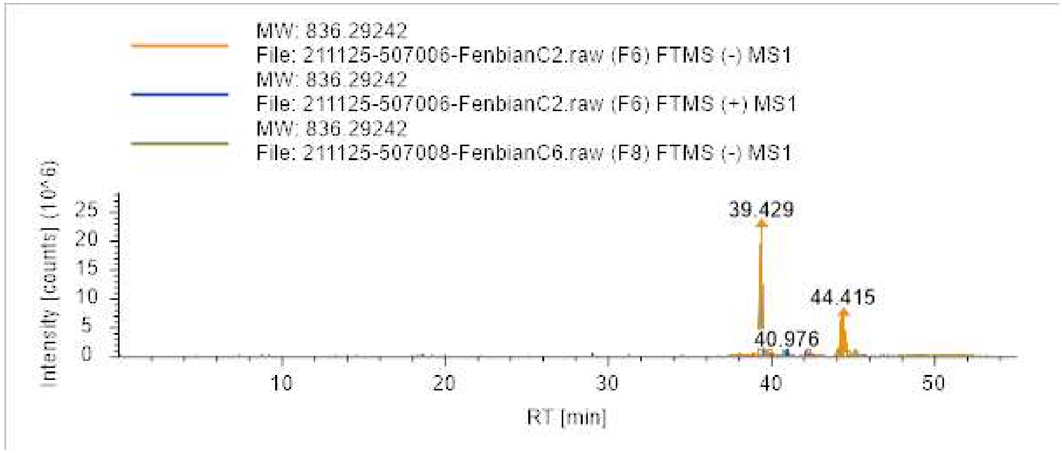

211125-507006-FenbianC2 (F6) #14361, RT=39.429 min, MS1, FTMS (-)

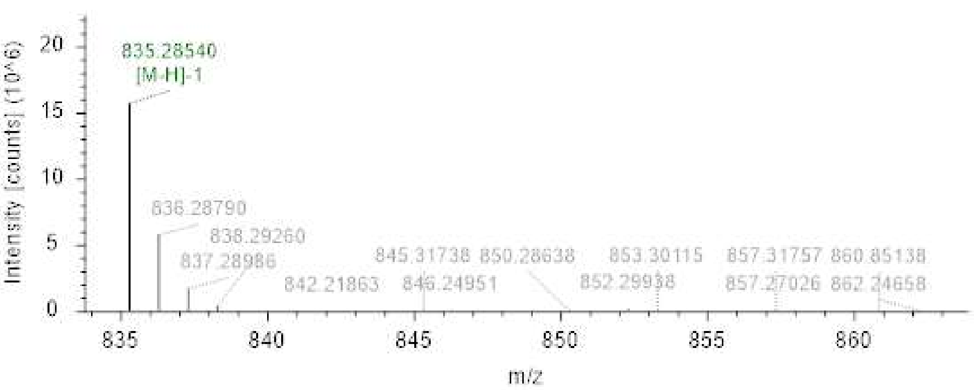

211125-507006-FenbianC2 (F6) #14362, RT=39.431 min, MS2, FTMS (-), (HCD, DDA, 835.2854@30, -1)

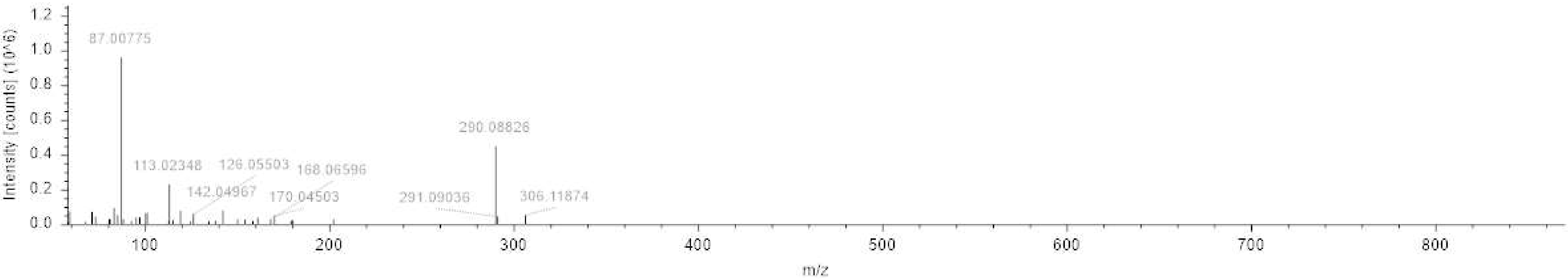

© Reported with Compound Discoverer 3.3

Compounds

Structure	Name	RT [min]	Formula	Calc. MW	Areas
n/a		42.09	n/a	837.31117	4.87e7 1.28e8 5.68e7 1.36e7 1.04e8 6.22e8

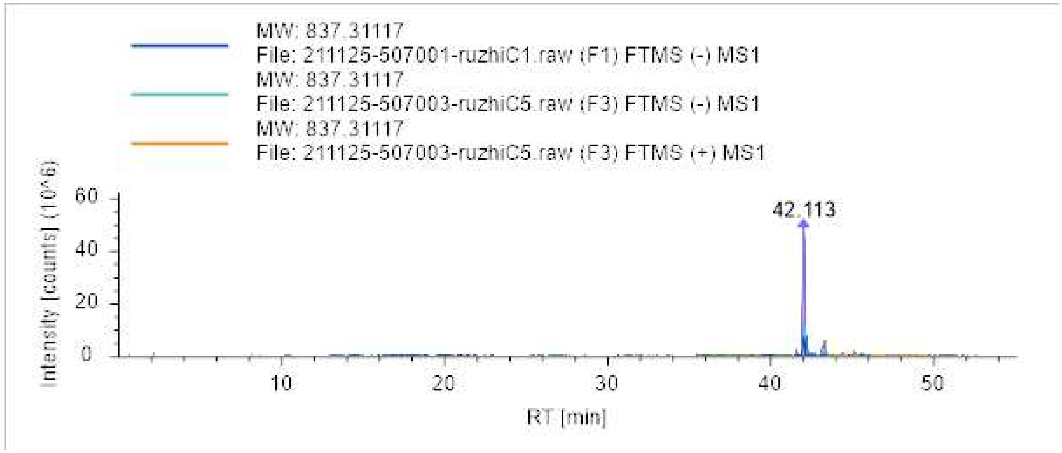

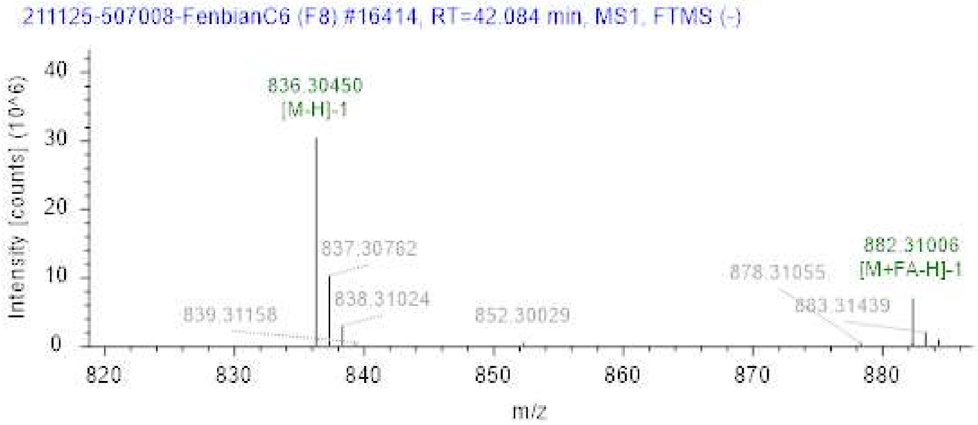

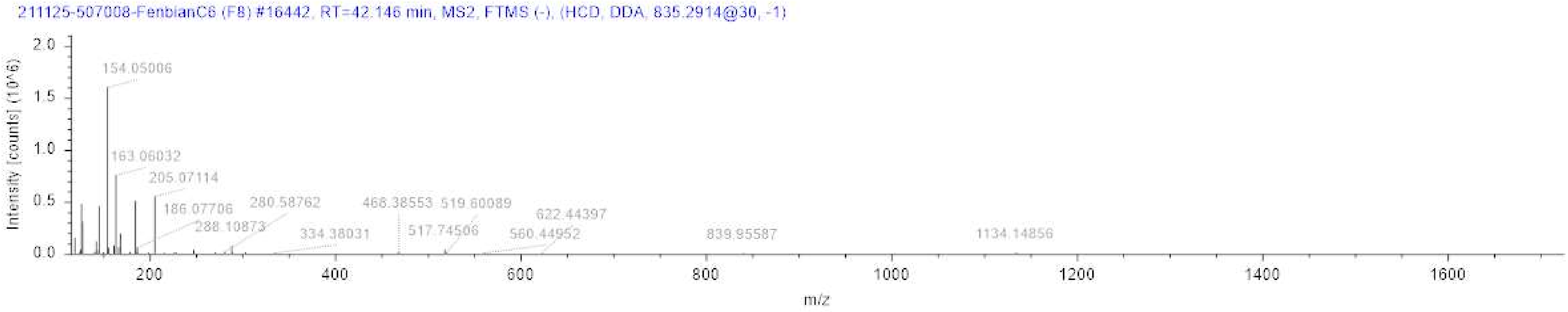

© Reported with Compound Discoverer 3.3

Compounds

Structure	Name	RT [min]	Formula	Calc. MW	Areas
n/a		41.65	n/a	837.31196	1.58e7

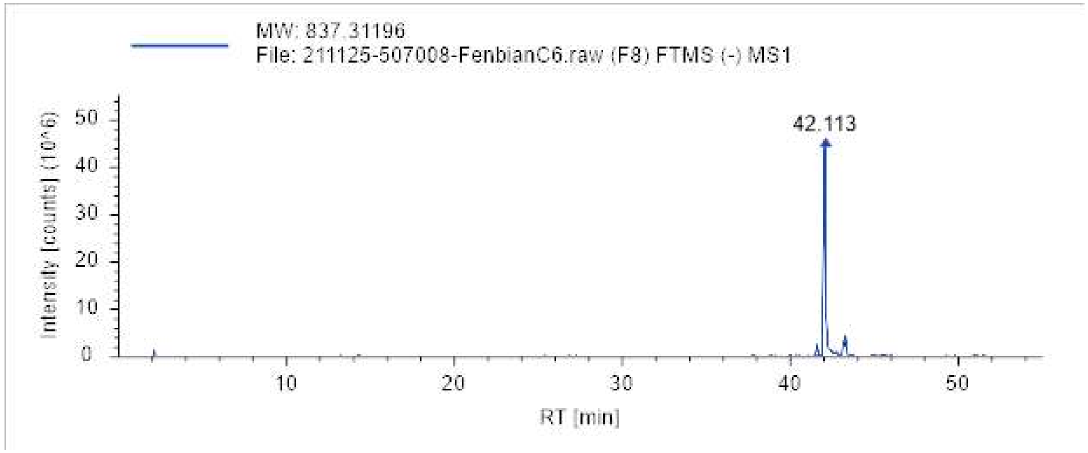

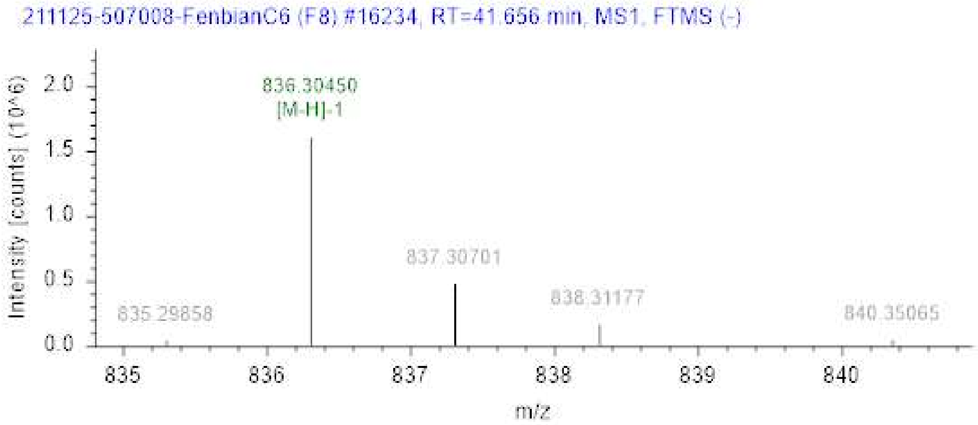

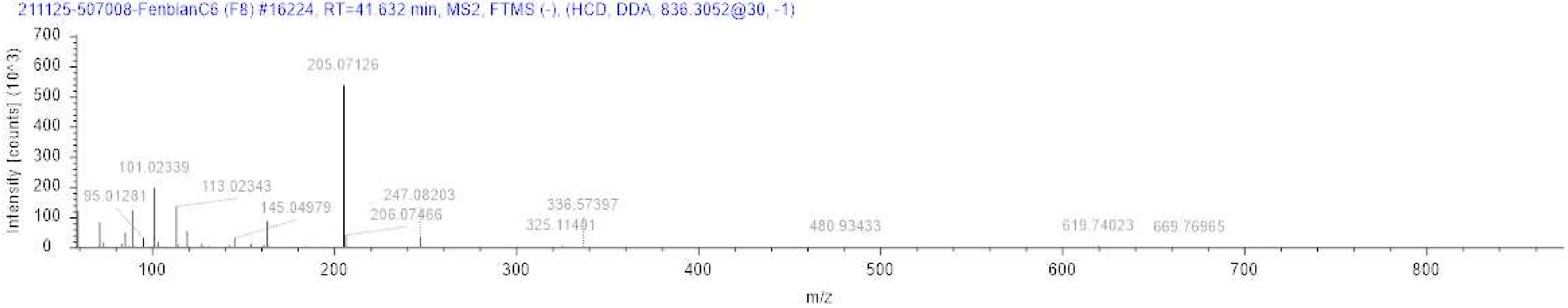

© Reported with Compound Discoverer 3.3

Compounds

Structure	Name	RT [min]	Formula	Calc. MW	Areas
n/a		43.34	n/a	837.31212	1.13e7 9.30e6 1.11e7 4.30e7

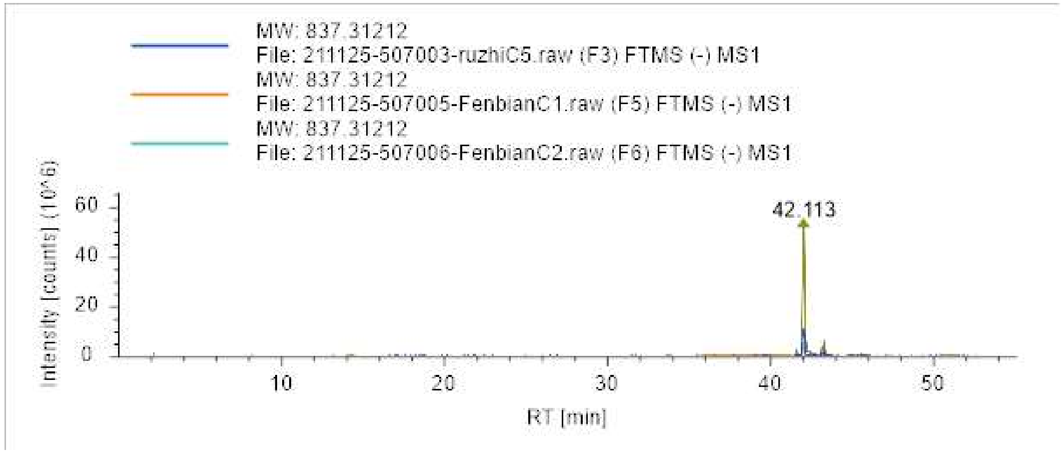

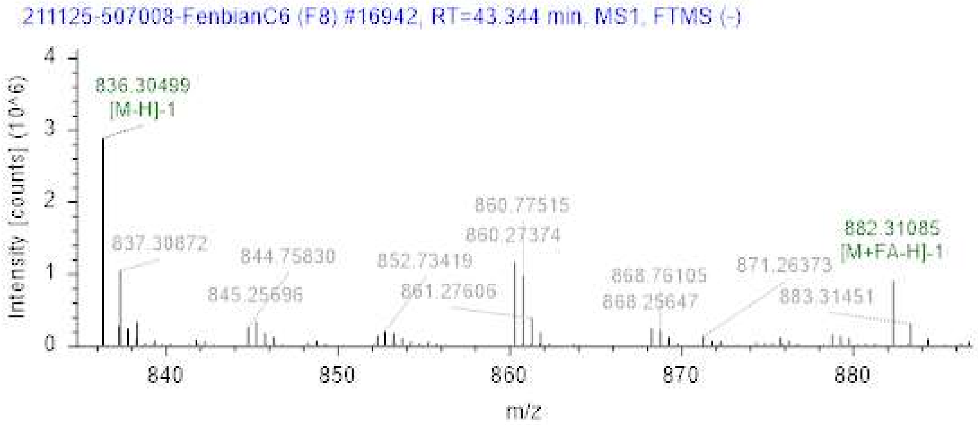

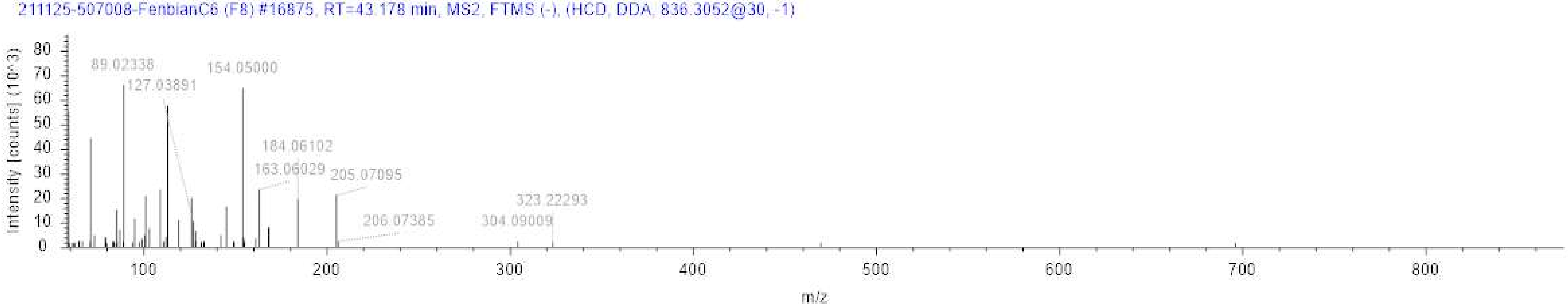

© Reported with Compound Discoverer 3.3

Compounds

Structure	Name	RT [min]	Formula	Calc. MW	Areas
n/a		26.48	n/a	838.30702	1.08e7

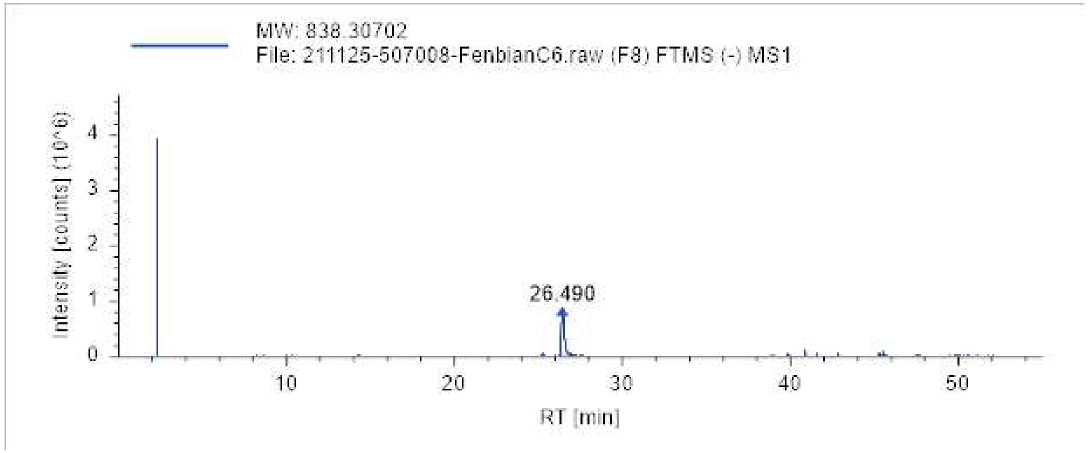

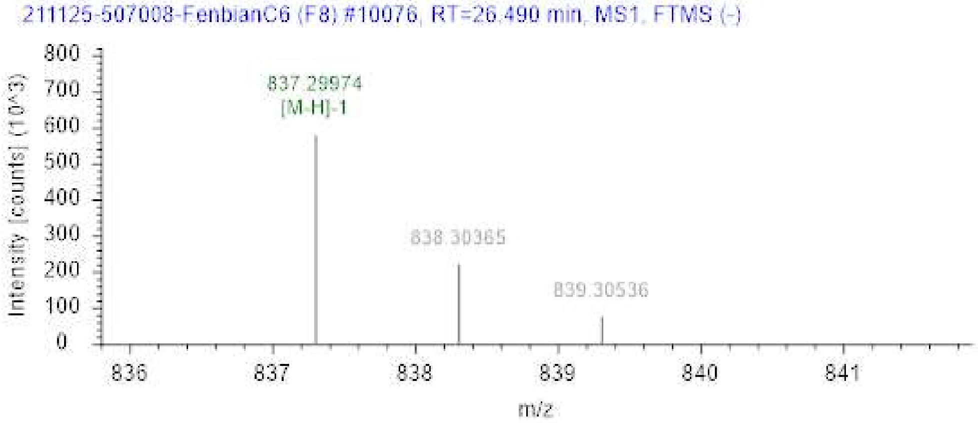

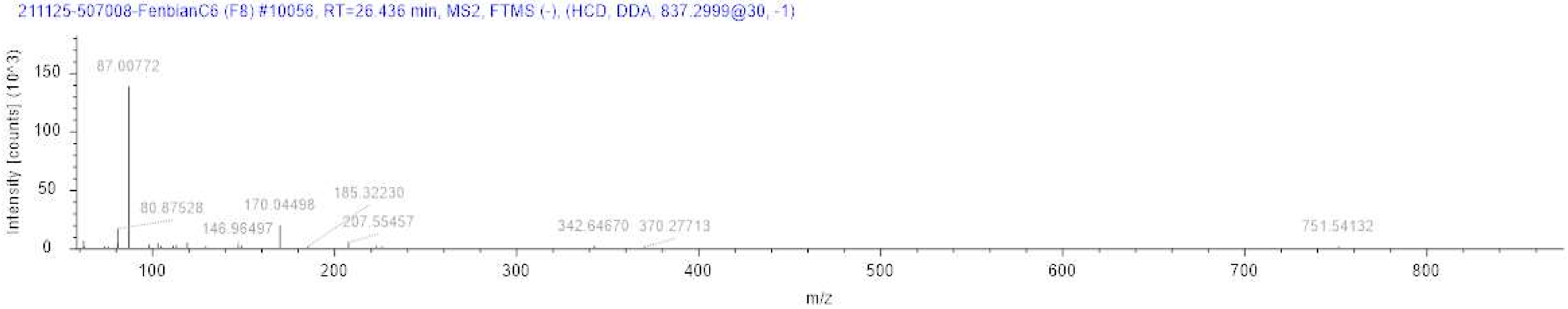

© Reported with Compound Discoverer 3.3

Compounds

Structure	Name	RT [min]	Formula	Calc. MW	Areas
n/a		44.74	n/a	853.30301	1.62e7, 1.33e7, 2.85e7, 3.15e8

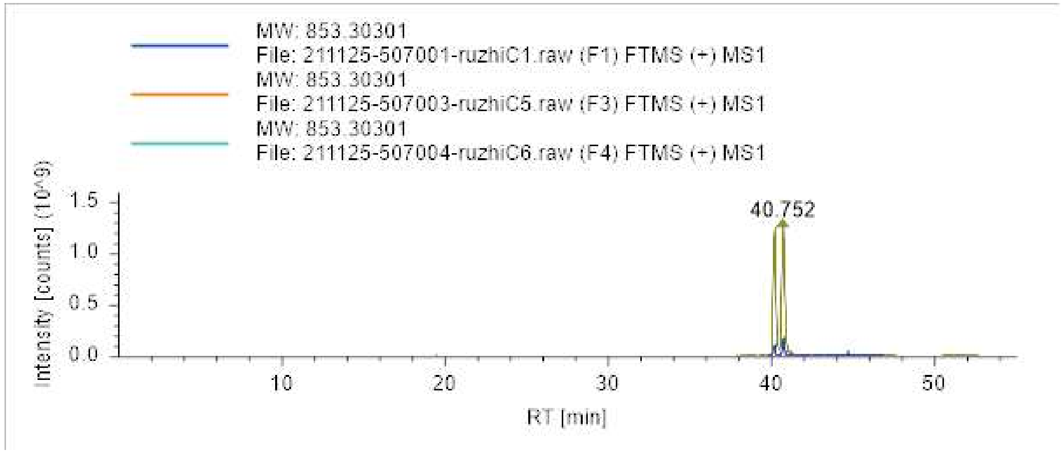

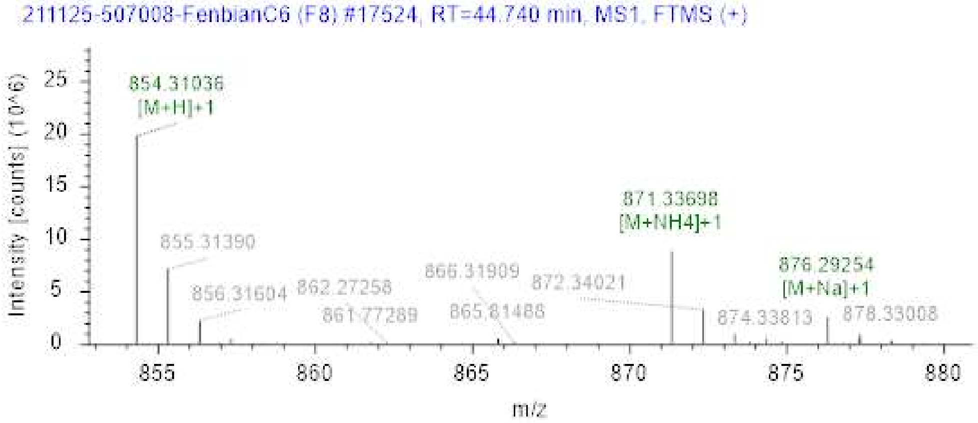

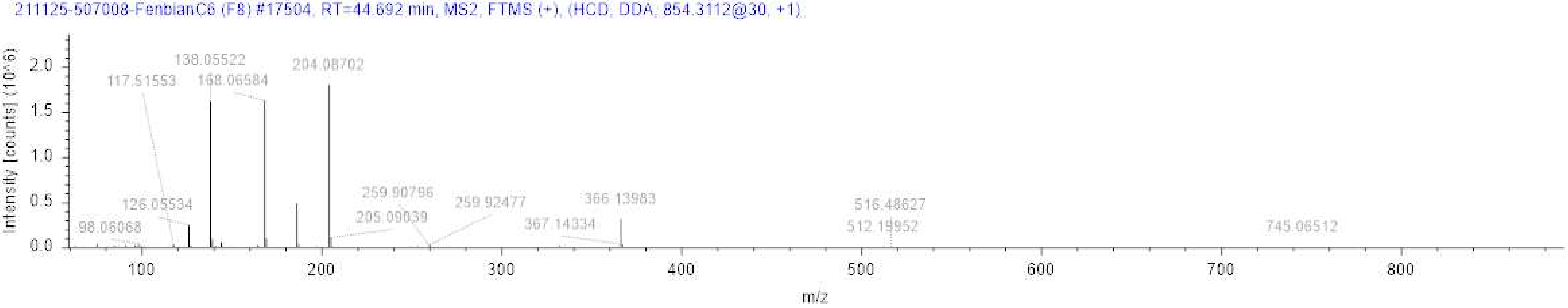

© Reported with Compound Discoverer 3.3

Compounds

Structure	Name	RT [min]	Formula	Calc. MW	Areas
n/a		42.09	n/a	853.30306	1.03e7 2.31e7 1.03e7 1.18e8

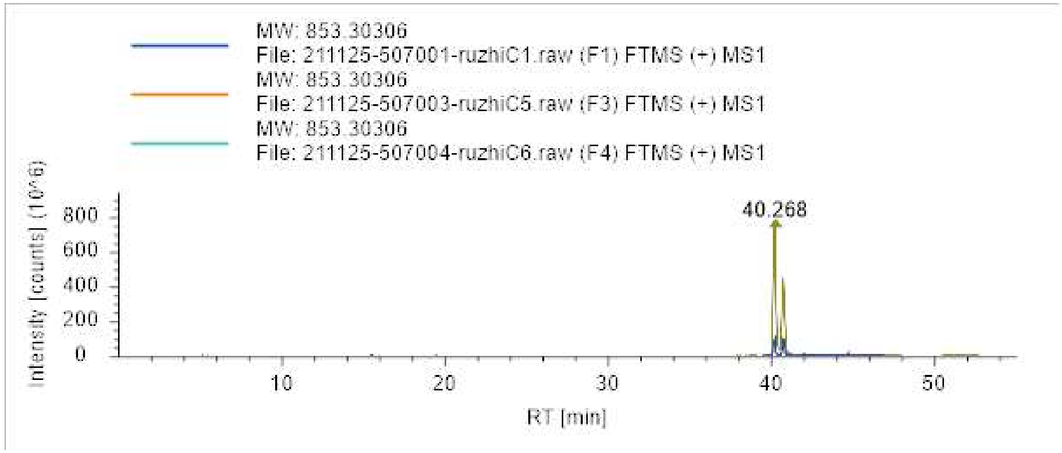

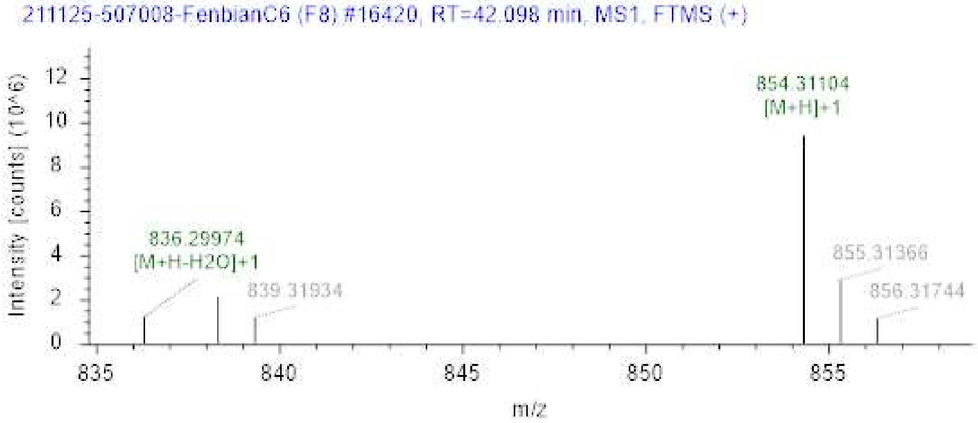

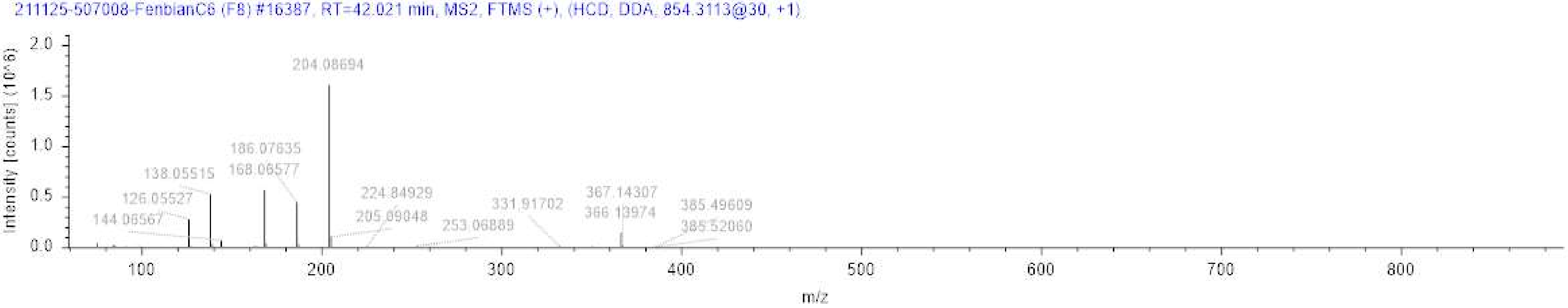

© Reported with Compound Discoverer 3.3

Compounds

Structure	Name	RT [min]	Formula	Calc. MW	Areas
n/a		42.86	n/a	853.30324	5.76e6 1.37e7 2.60e7 3.41e7

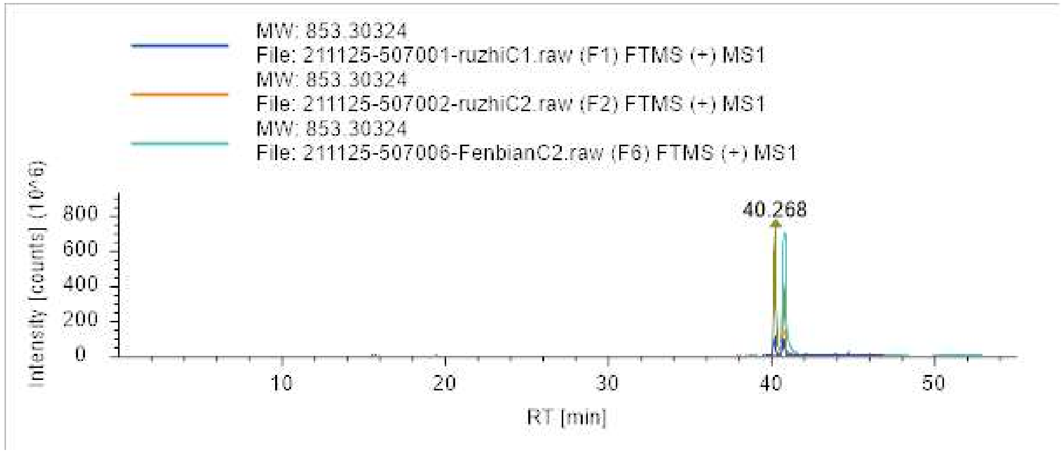

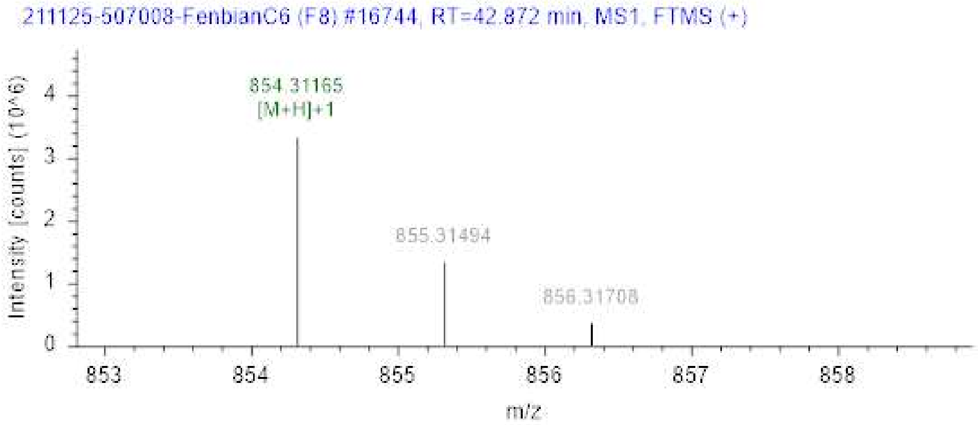

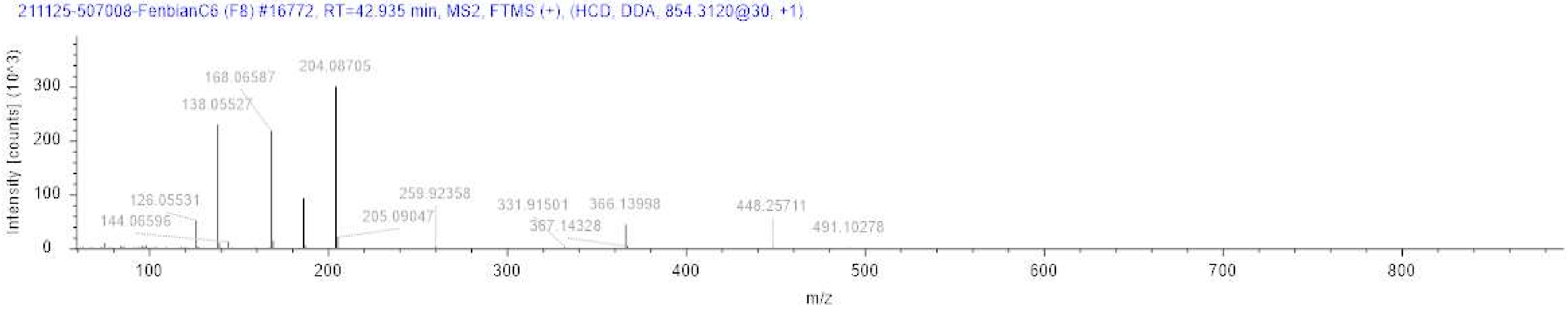

© Reported with Compound Discoverer 3.3

Compounds

Structure	Name	RT [min]	Formula	Calc. MW	Areas
n/a		43.58	n/a	853.30344	4.75e6 4.70e6 6.37e7

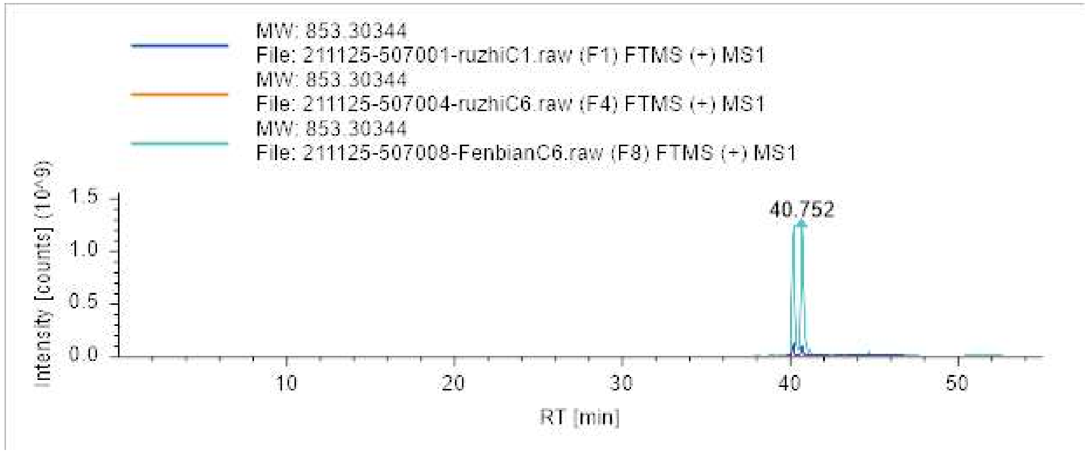

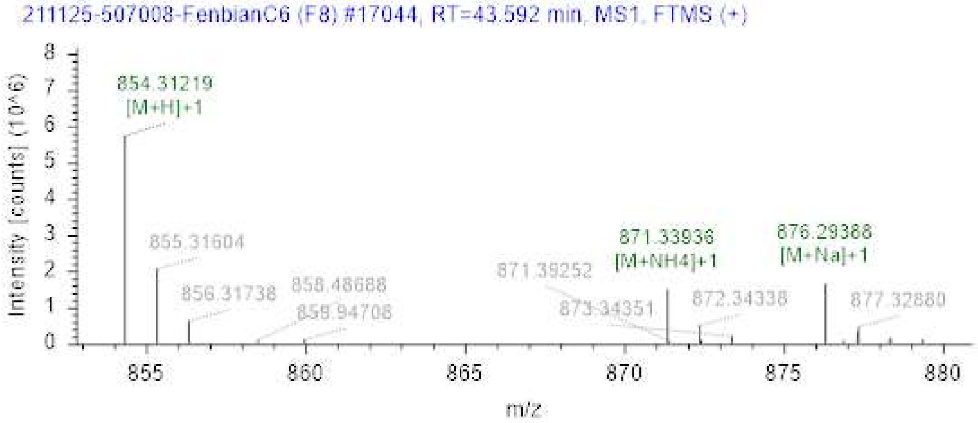

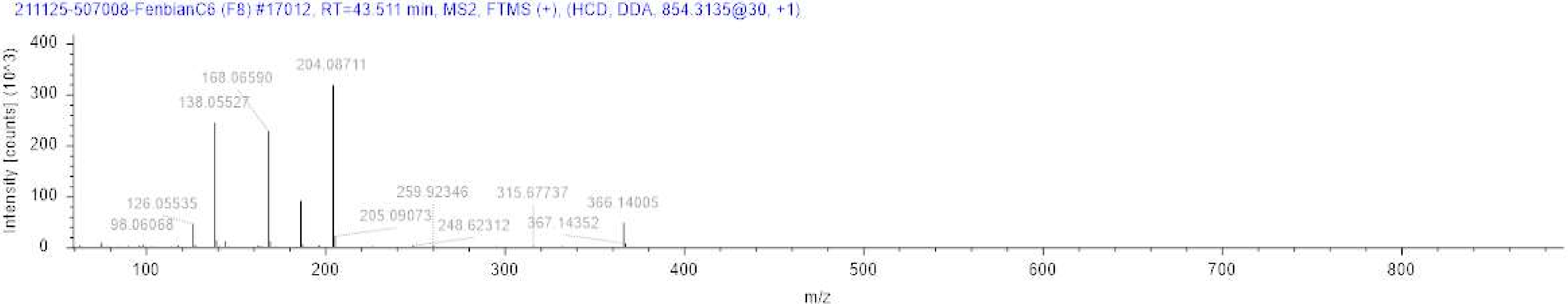

© Reported with Compound Discoverer 3.3

Structure	Name	RT [min]	Formula	Calc. MW	Areas
n/a		40.24	n/a	853.30667	2.56e9 9.02e7 2.36e9 3.90e9 4.11e8 3.90e8 1.87e10

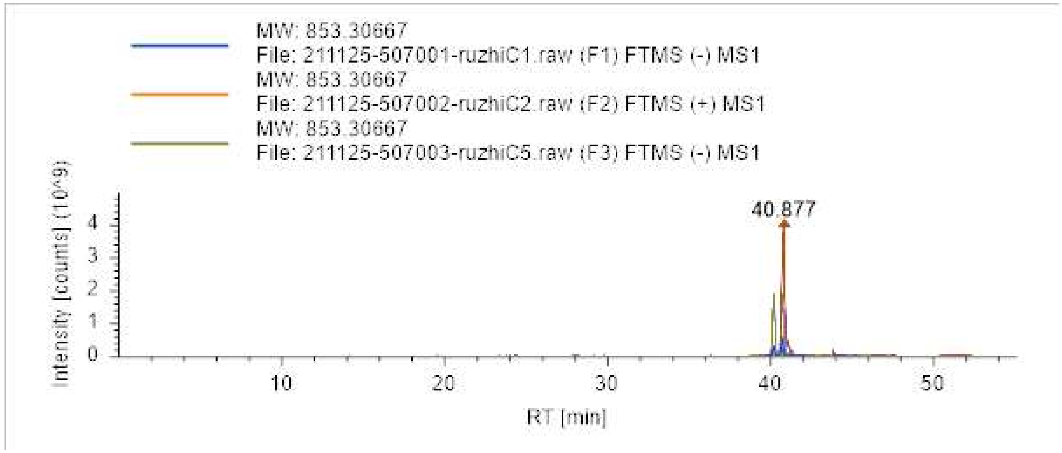

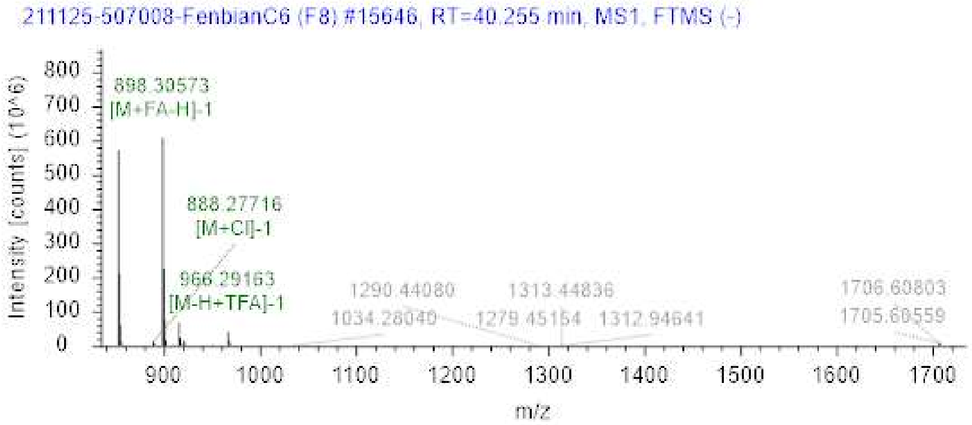

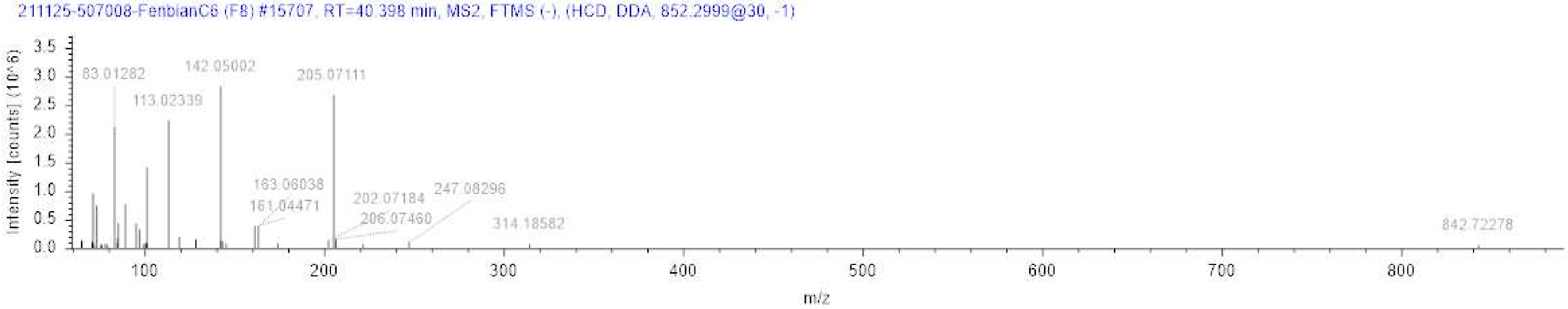

© Reported with Compound Discoverer 3.3

Structure	Name	RT [min]	Formula	Calc. MW	Areas
n/a		30.10	n/a	854.30214	1.13e7

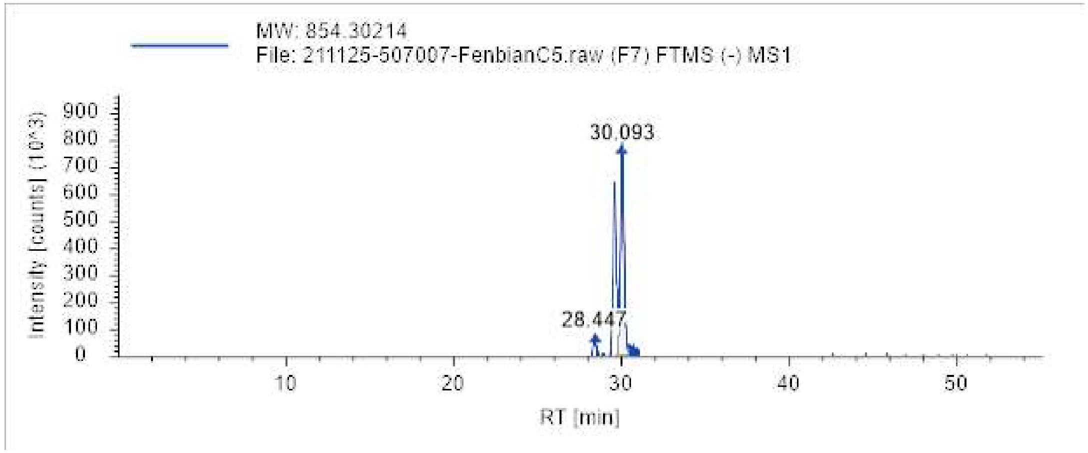

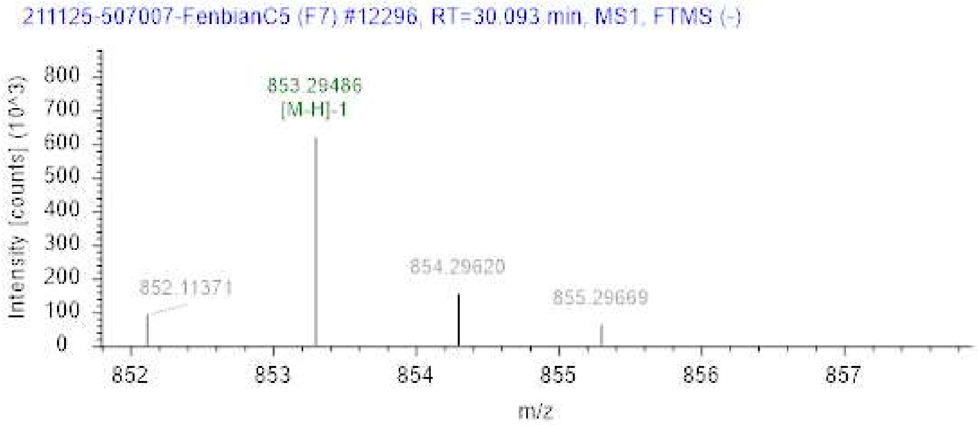

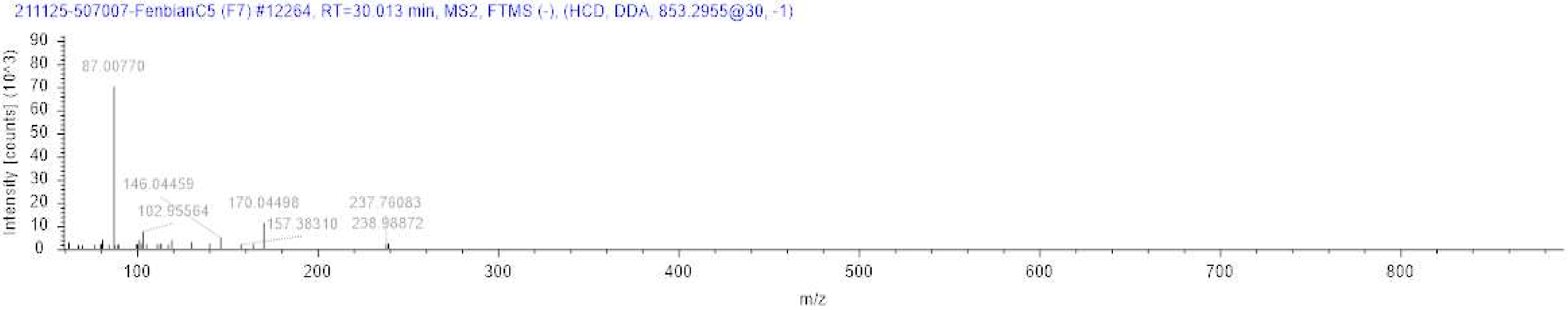

© Reported with Compound Discoverer 3.3

Compounds

Structure	Name	RT [min]	Formula	Calc. MW	Areas
n/a		41.02	n/a	869.30053	1.30e7 2.55e7 2.39e7 1.11e8 2.27e7 9.54e8

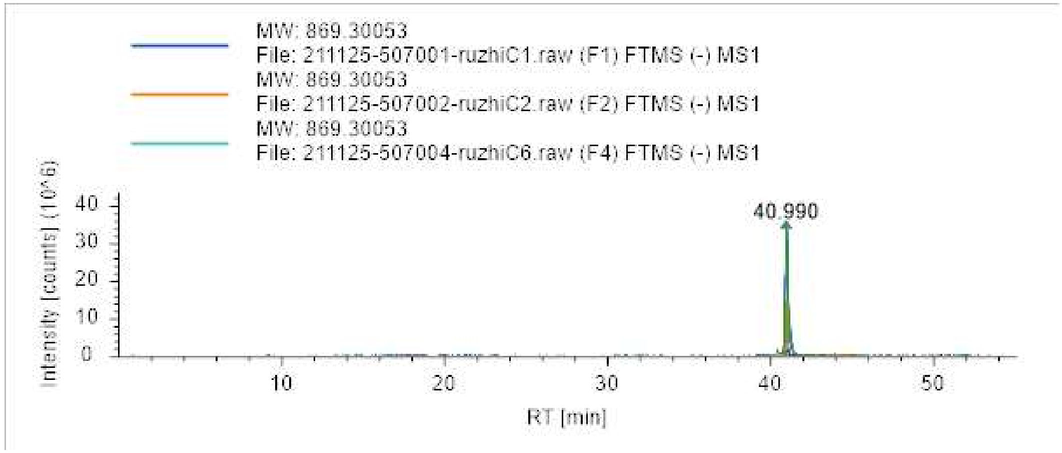

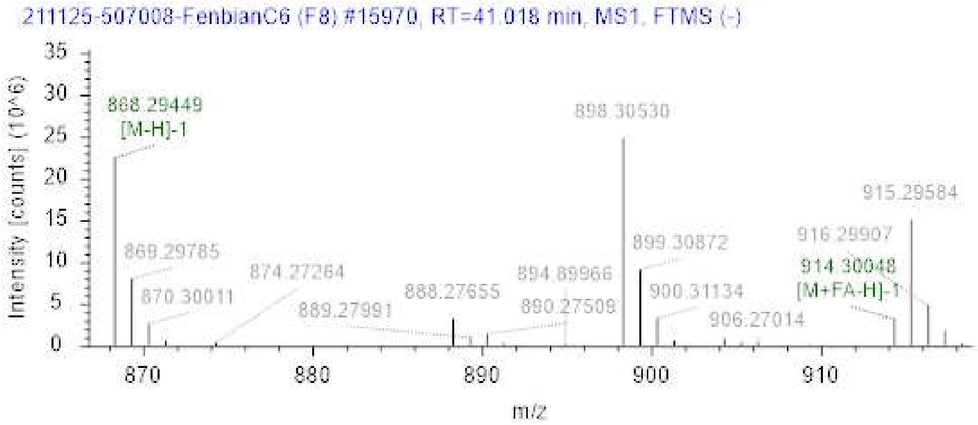

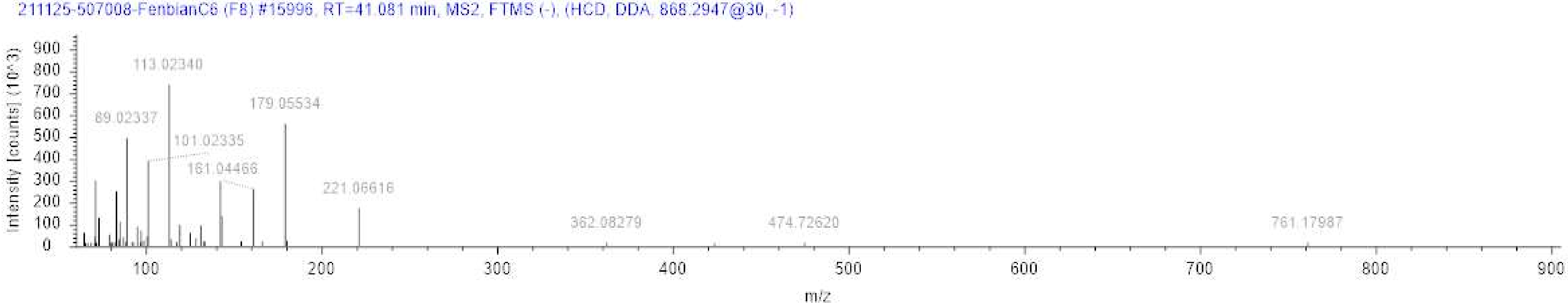

© Reported with Compound Discoverer 3.3

Compounds

Structure	Name	RT [min]	Formula	Calc. MW	Areas
n/a		41.04	n/a	870.29595	2.01e7

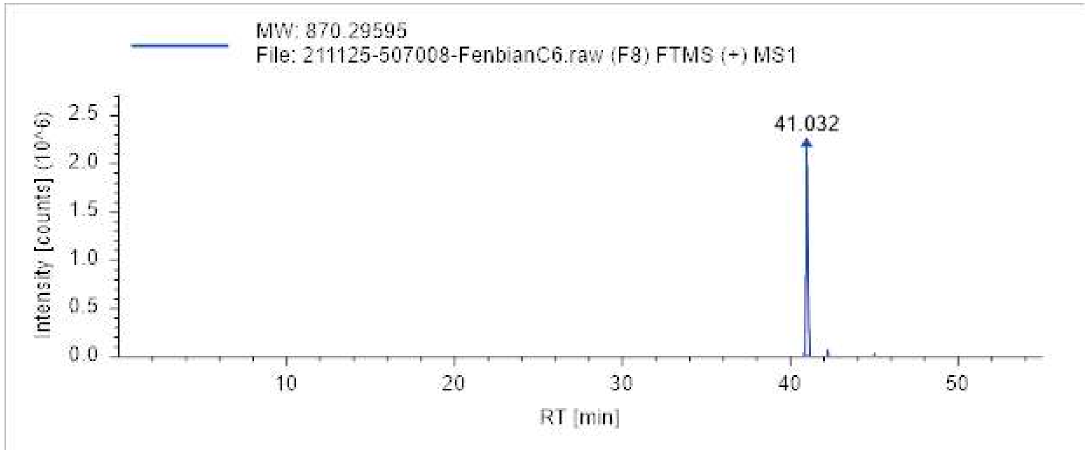

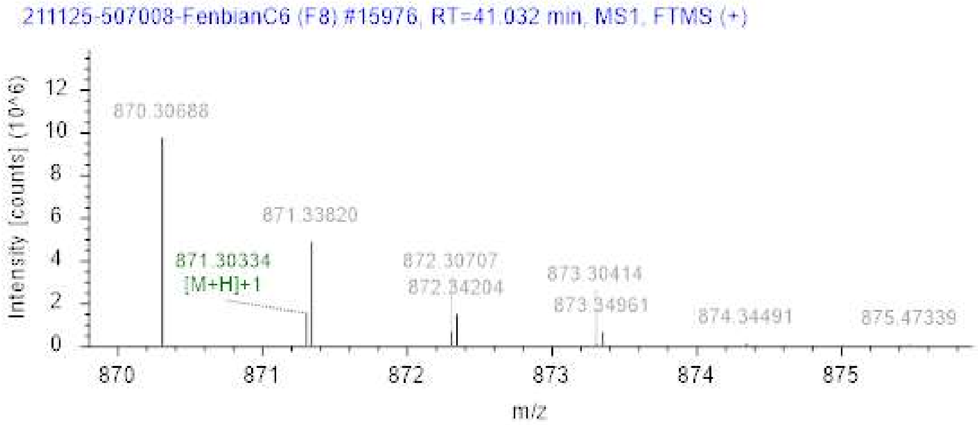

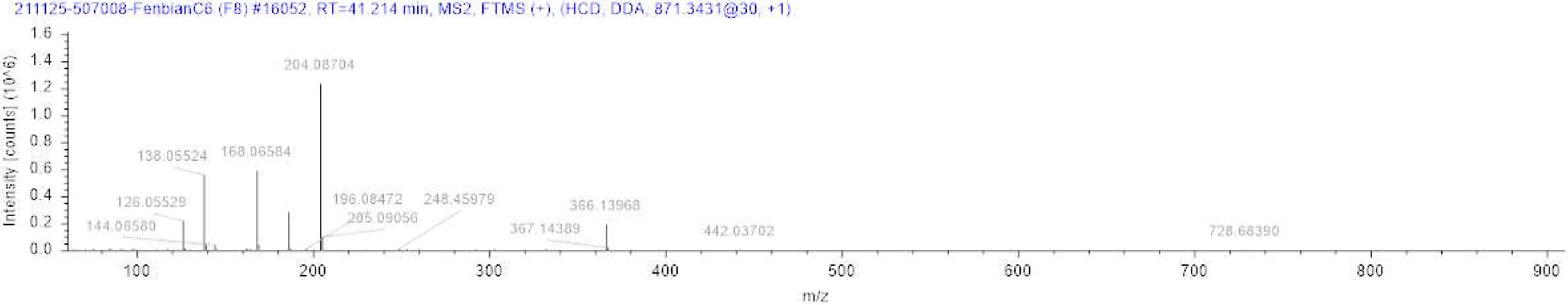

© Reported with Compound Discoverer 3.3

Compounds

Structure	Name	RT [min]	Formula	Calc. MW	Areas
n/a		40.20	n/a	894.33231	1.07e8

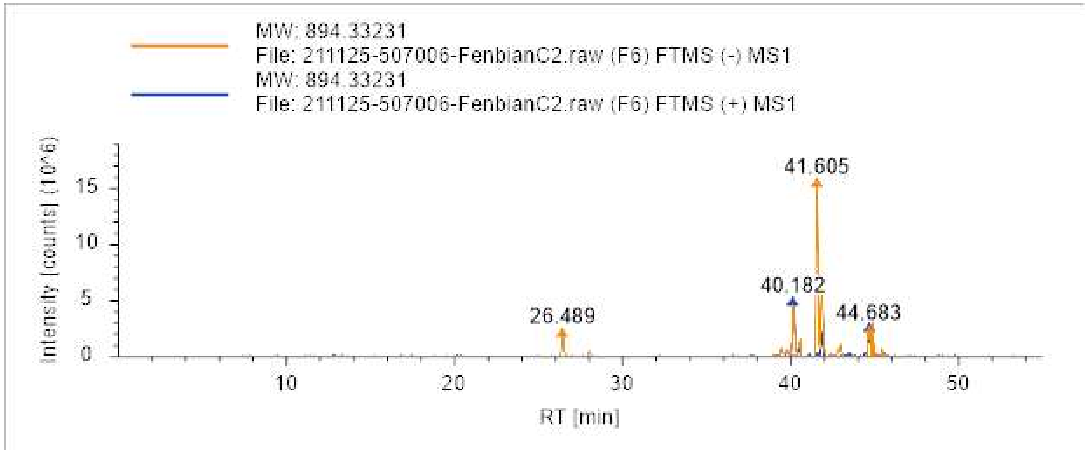

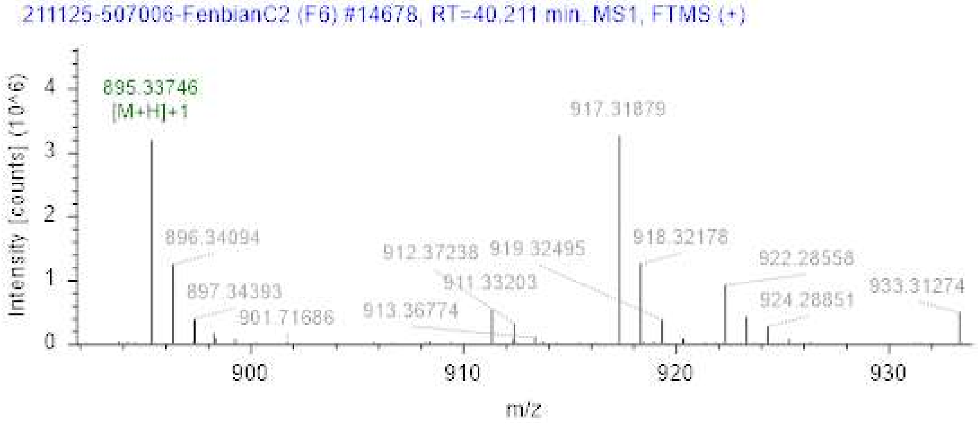

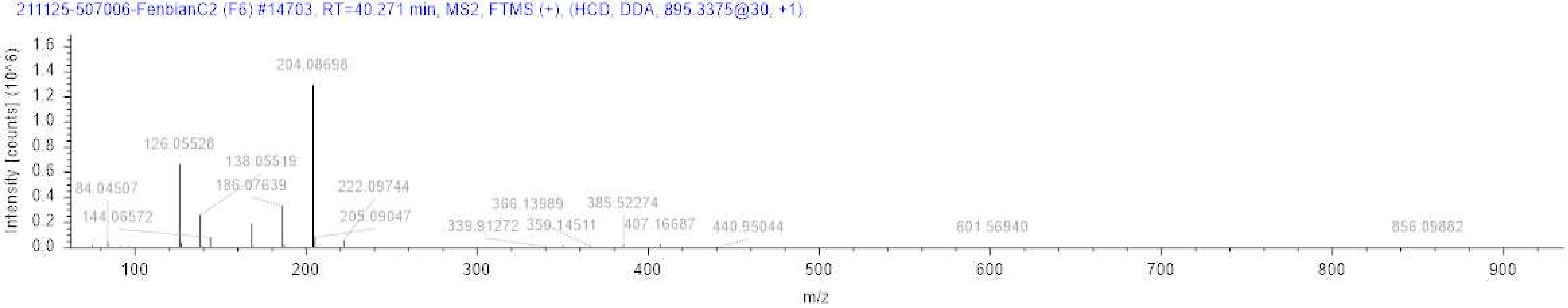

© Reported with Compound Discoverer 3.3

Structure	Name	RT [min]	Formula	Calc. MW	Areas
n/a		26.49	n/a	894.33284	1.82e7

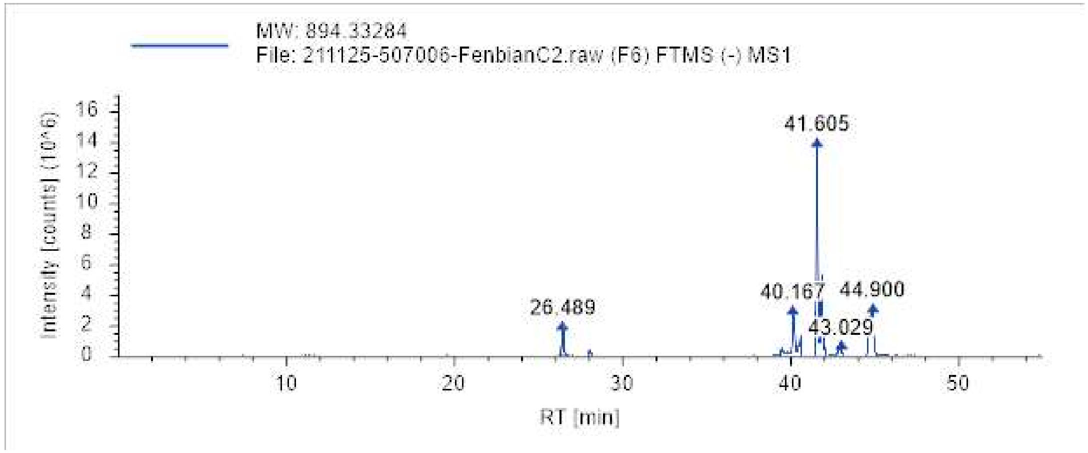

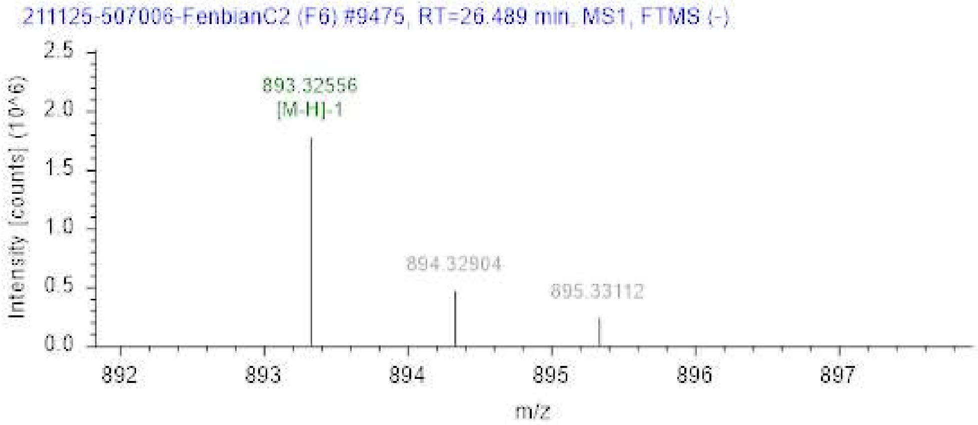

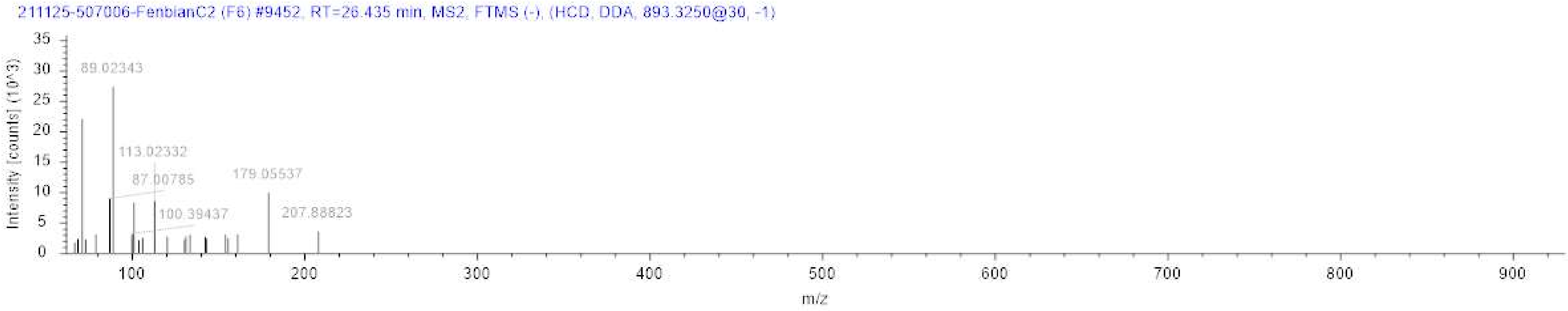

© Reported with Compound Discoverer 3.3

Compounds

Structure	Name	RT [min]	Formula	Calc. MW	Areas
n/a		41.60	n/a	894.33336	9.62e7 1.01e7

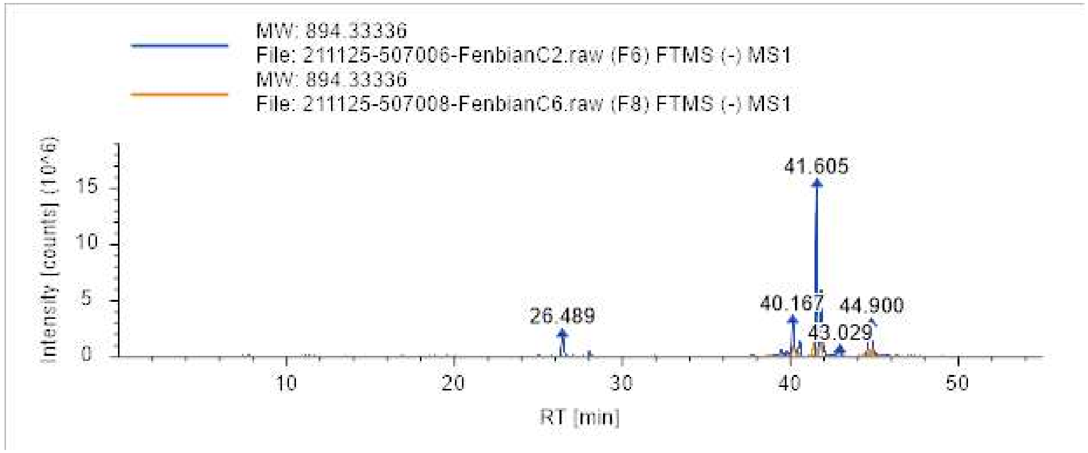

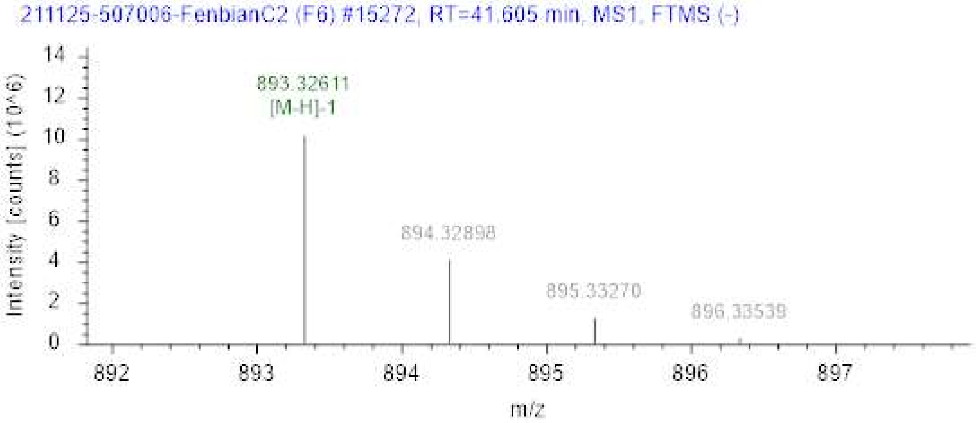

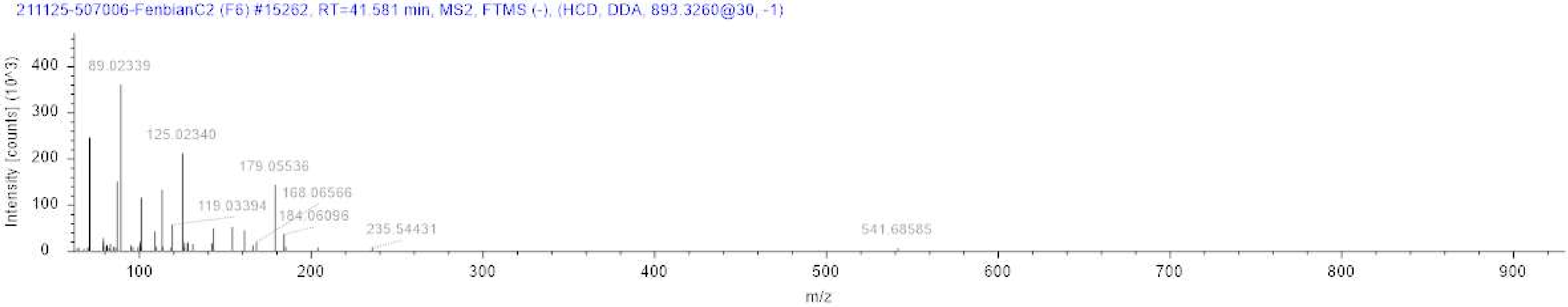

© Reported with Compound Discoverer 3.3

Compounds

Structure	Name	RT [min]	Formula	Calc. MW	Areas
n/a		41.61	n/a	910.32494	6.52e7

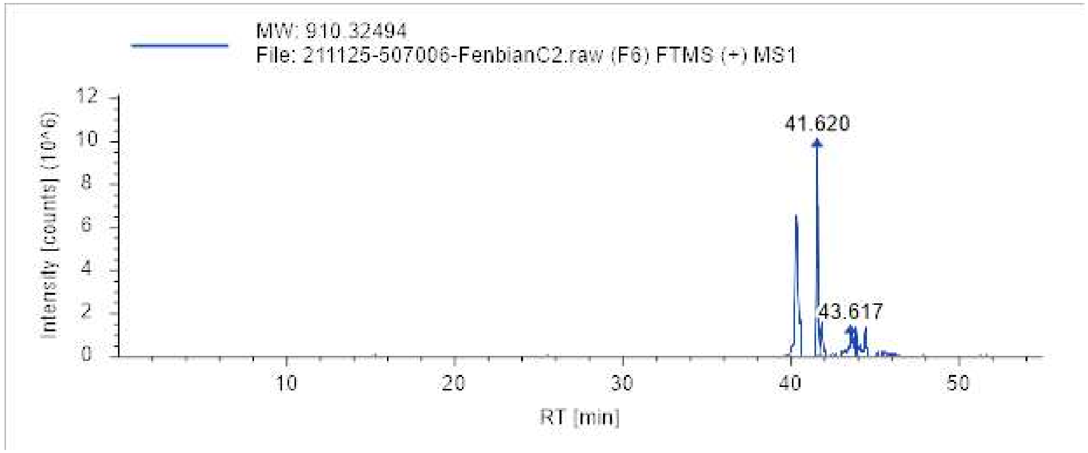

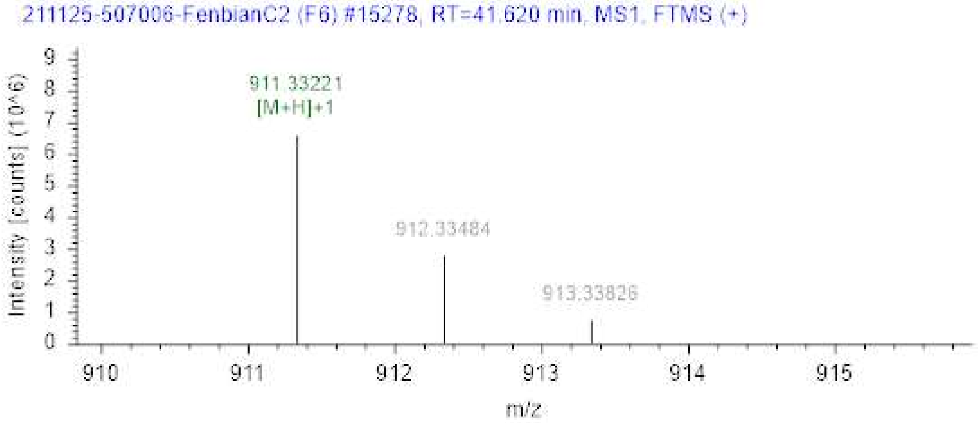

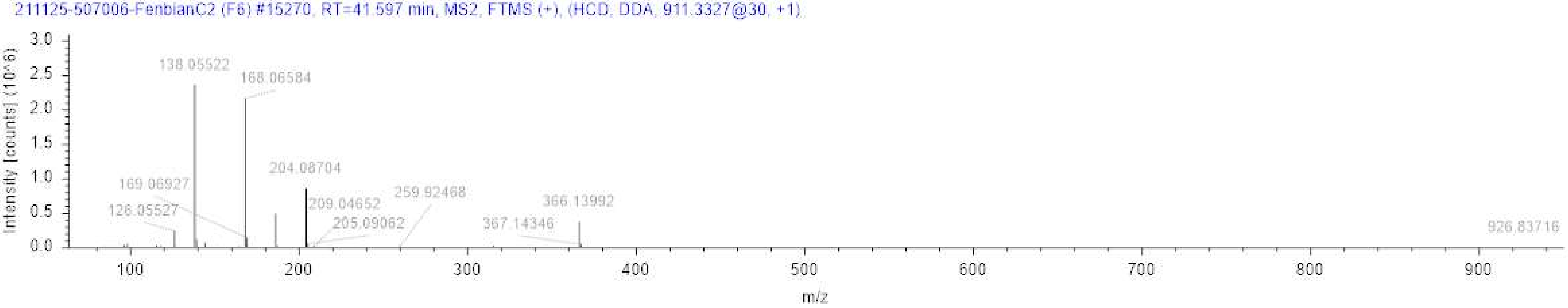

© Reported with Compound Discoverer 3.3

Compounds

Structure	Name	RT [min]	Formula	Calc. MW	Areas
n/a		42.71	n/a	910.32561	1.01e7 8.30e7

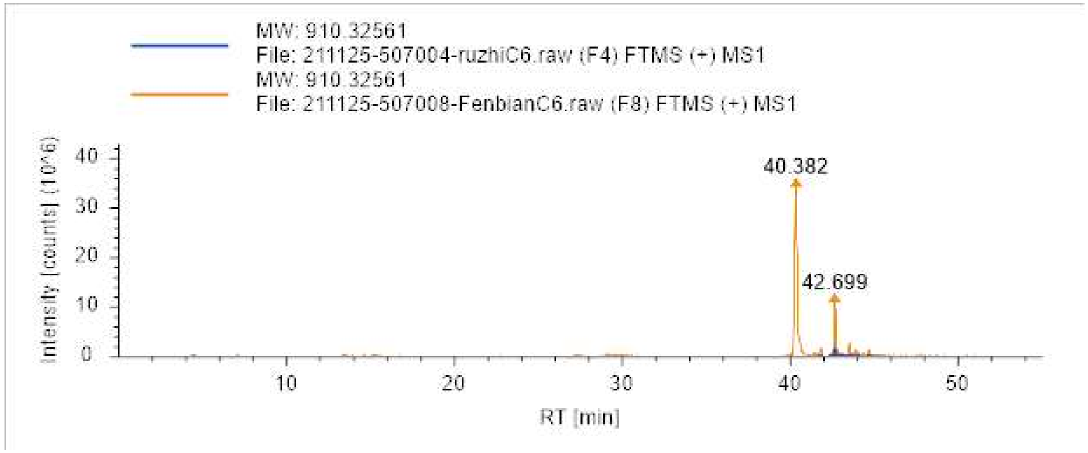

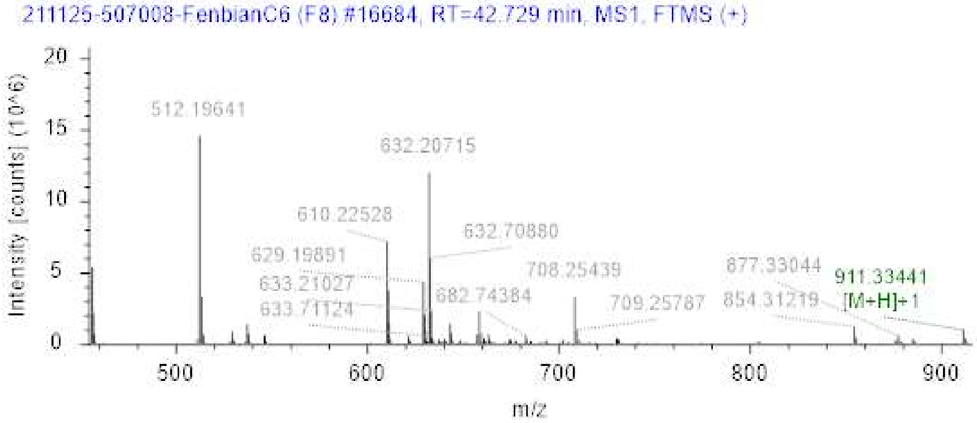

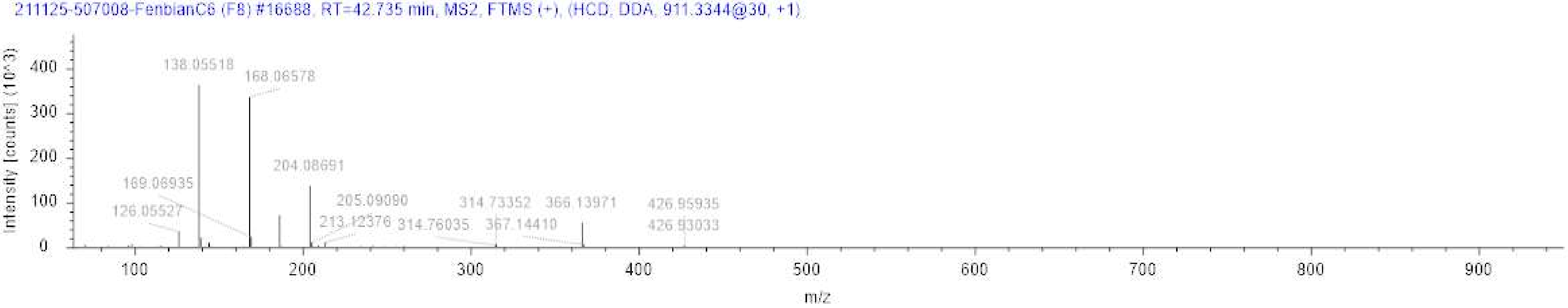

© Reported with Compound Discoverer 3.3

Compounds

Structure	Name	RT [min]	Formula	Calc. MW	Areas
n/a		40.37	n/a	910.32608	3.66e8 1.67e7 1.89e9

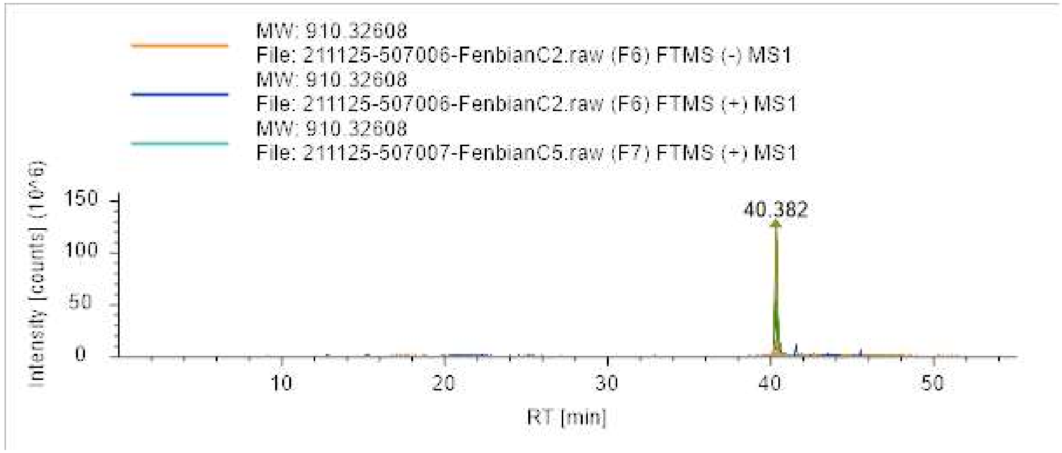

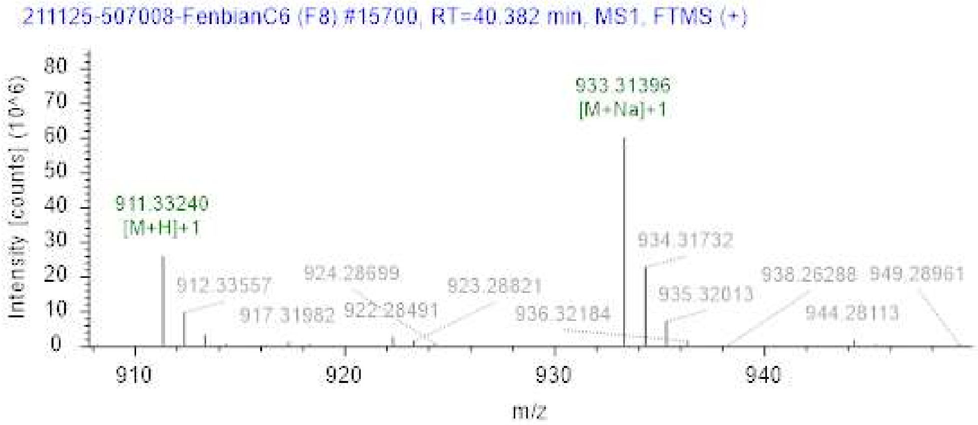

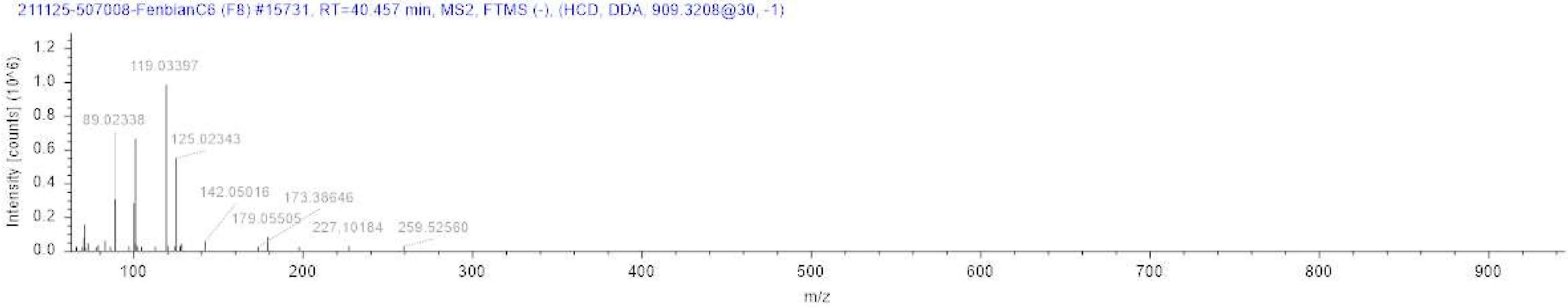

© Reported with Compound Discoverer 3.3

Structure	Name	RT [min]	Formula	Calc. MW	Areas
n/a		36.37	n/a	942.31996	1.68e7

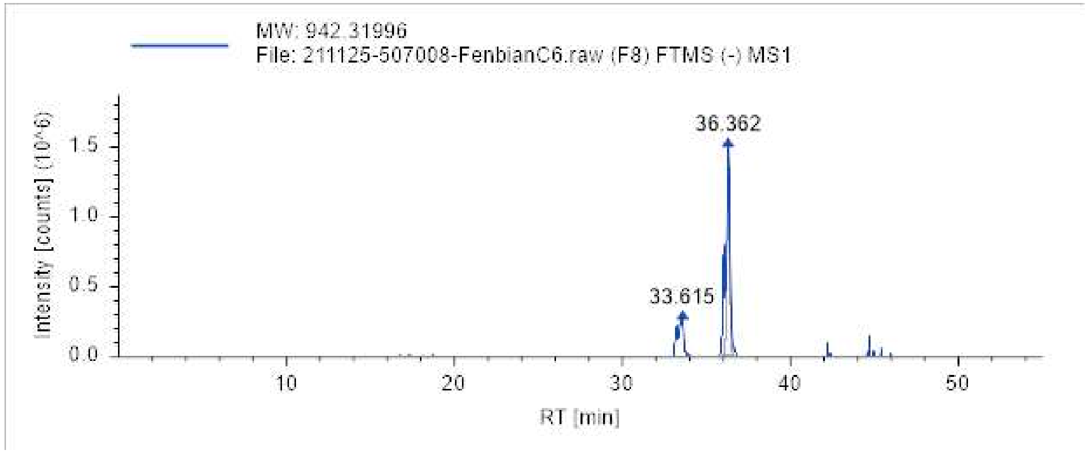

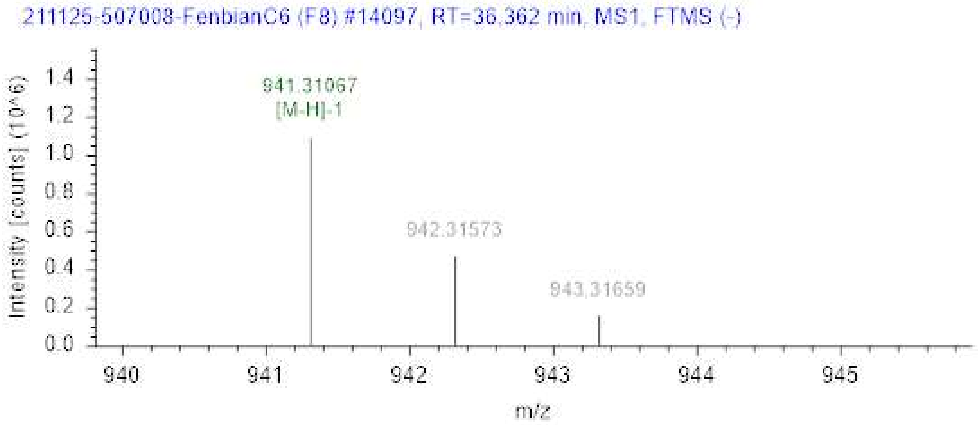

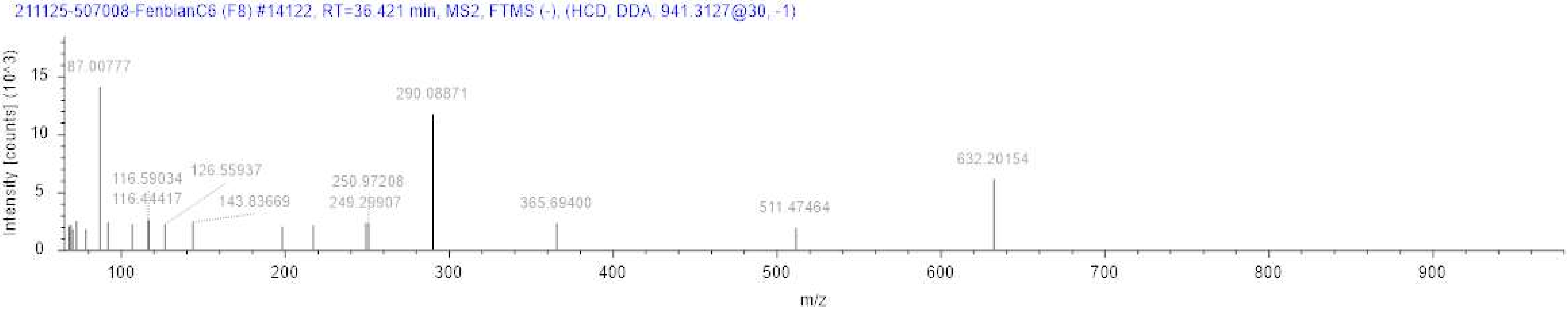

© Reported with Compound Discoverer 3.3

Compounds

Structure	Name	RT [min]	Formula	Calc. MW	Areas
n/a		39.24	n/a	951.35124	1.51e8

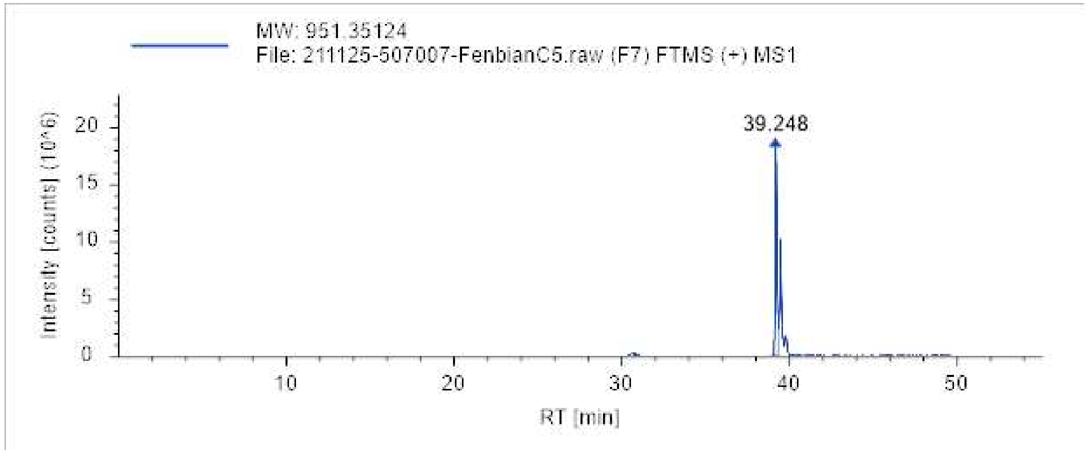

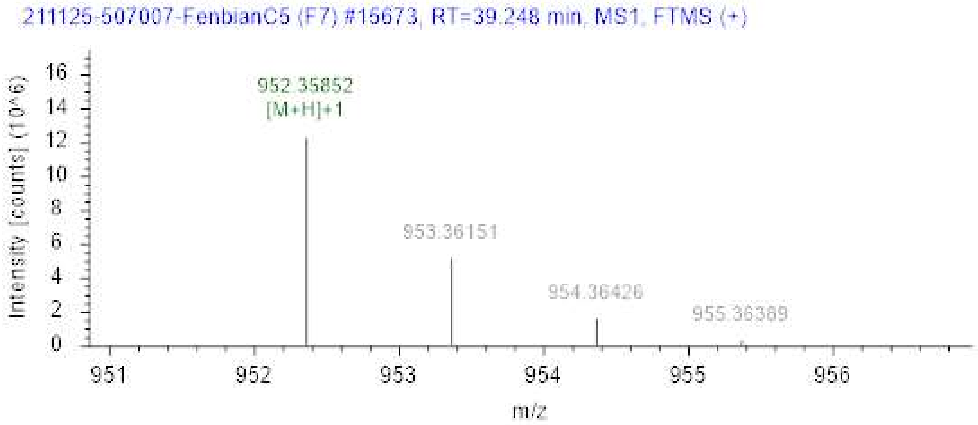

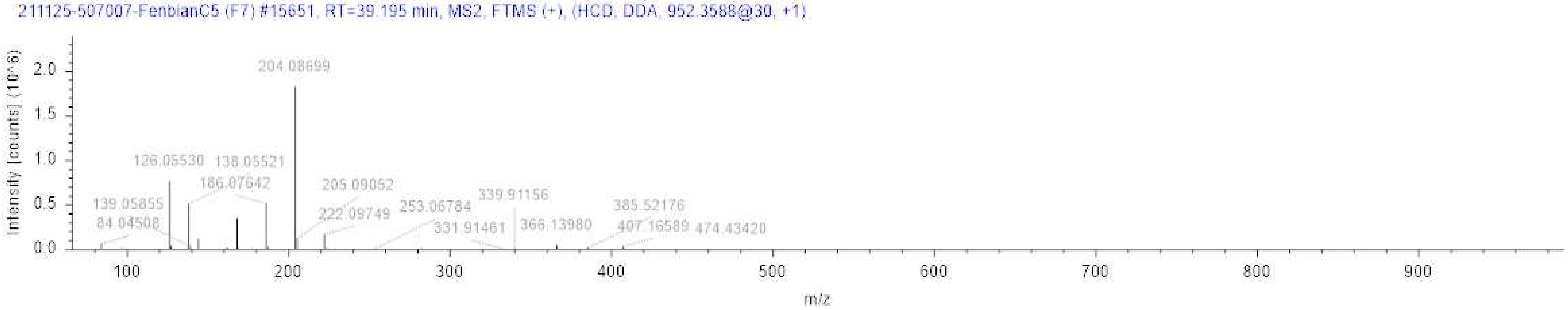

© Reported with Compound Discoverer 3.3

Compounds

Structure	Name	RT [min]	Formula	Calc. MW	Areas
n/a		30.13	n/a	958.33569	3.14e7

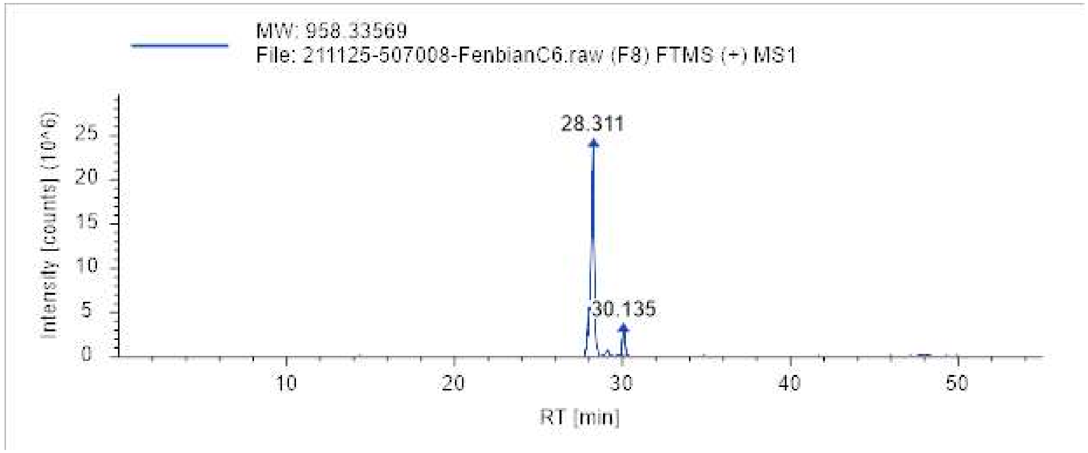

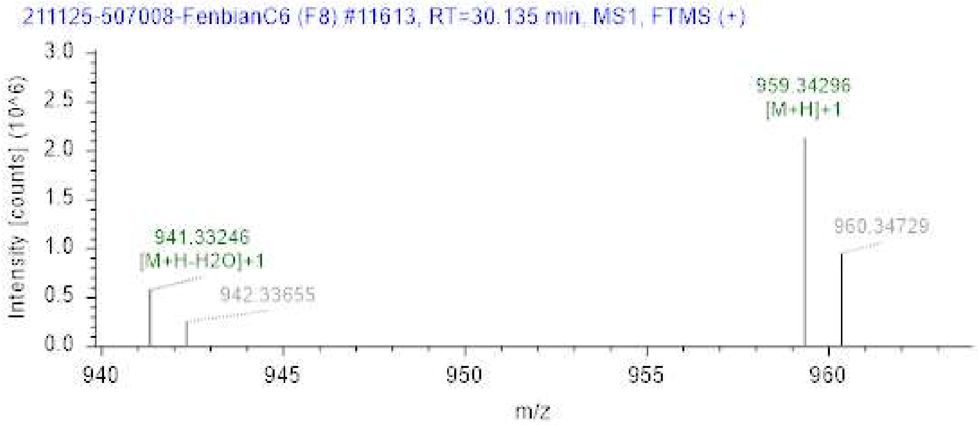

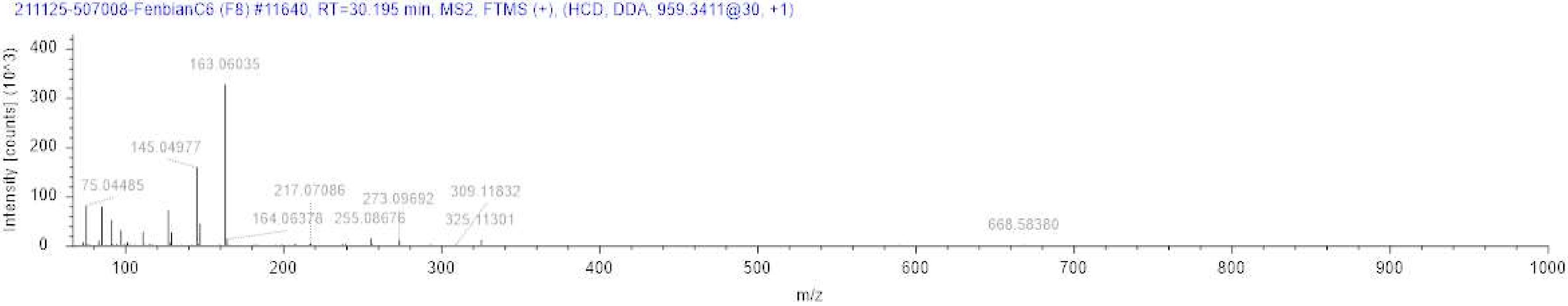

© Reported with Compound Discoverer 3.3

Compounds

Structure	Name	RT [min]	Formula	Calc. MW	Areas
n/a		40.22	n/a	958.33601	8.79e6

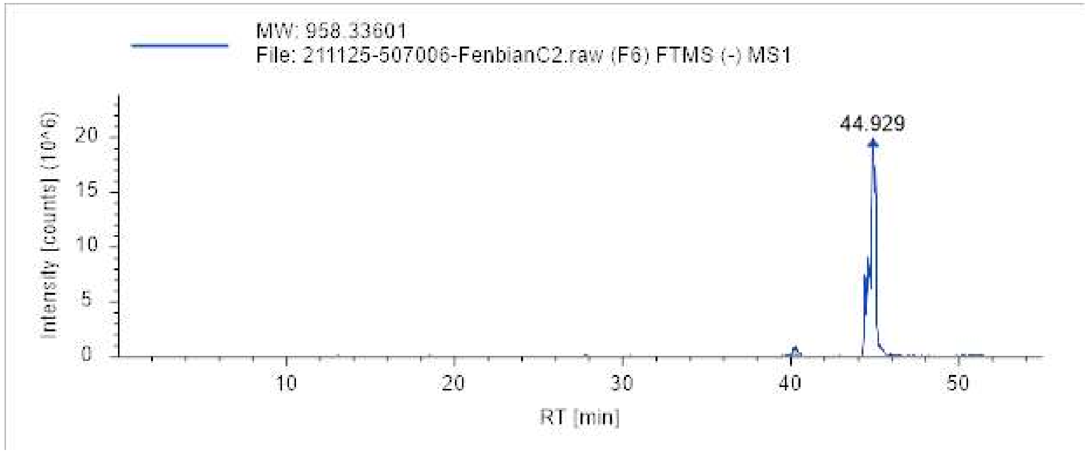

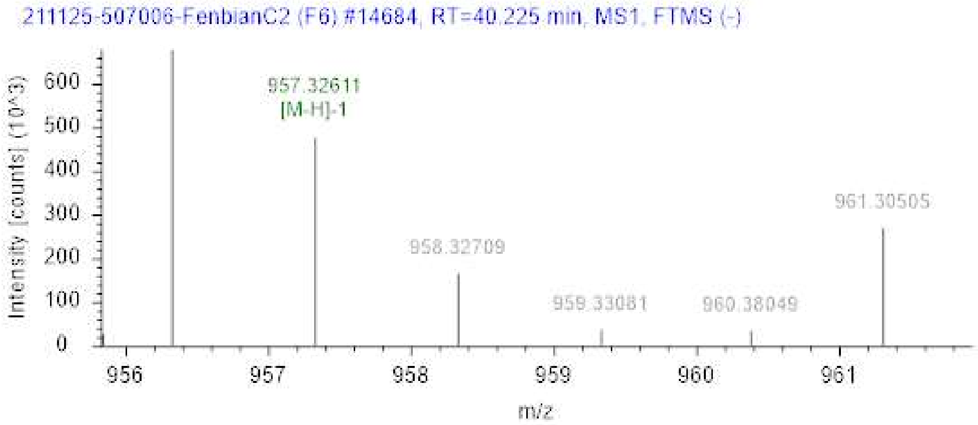

© Reported with Compound Discoverer 3.3

Compounds

Structure	Name	RT [min]	Formula	Calc. MW	Areas
n/a		41.32	n/a	981.33165	1.30e7

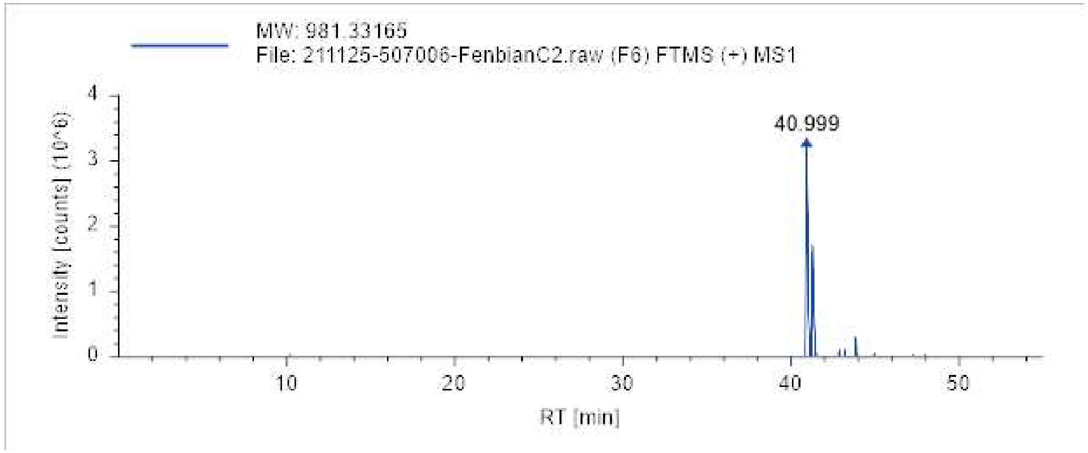

211125-507006-FenbianC2 (F6) #15146, RT=41.308 min, MS1, FTMS (+)

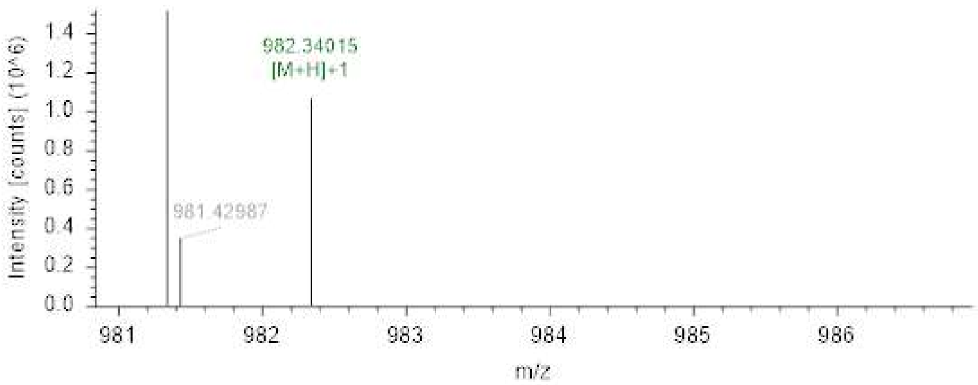

211125-507006-FenbianC2 (F6) #15150, RT=41.314 min, MS2, FTMS (+), (HCD, DDA, 981.3372@30, +1)

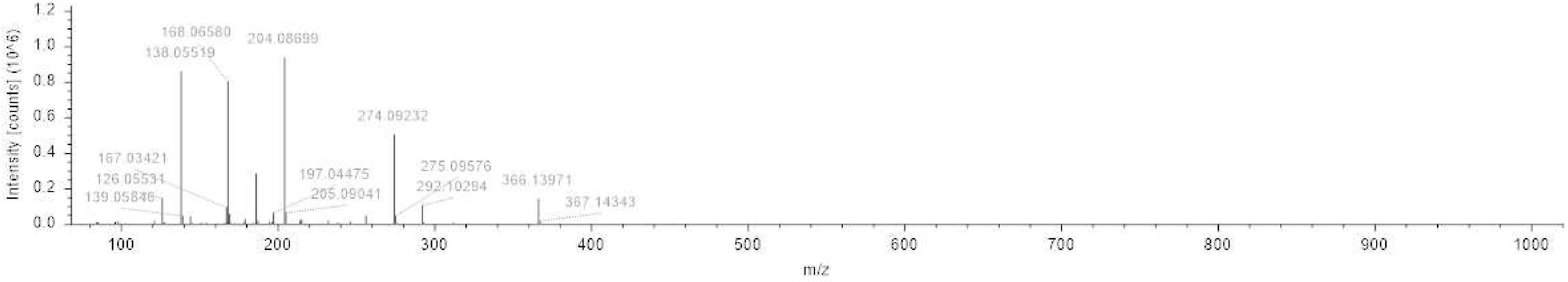

© Reported with Compound Discoverer 3.3

Compounds

Structure	Name	RT [min]	Formula	Calc. MW	Areas
n/a		45.72	n/a	998.34020	3.14e7

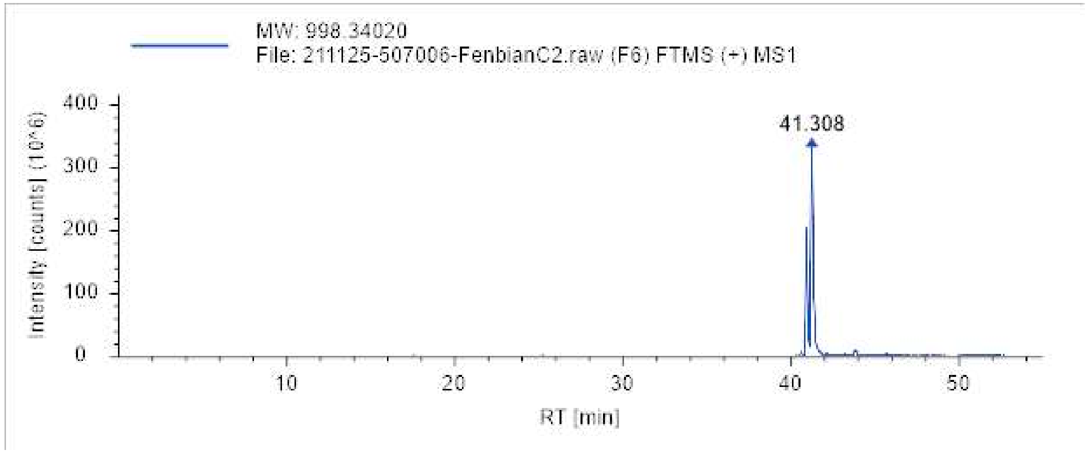

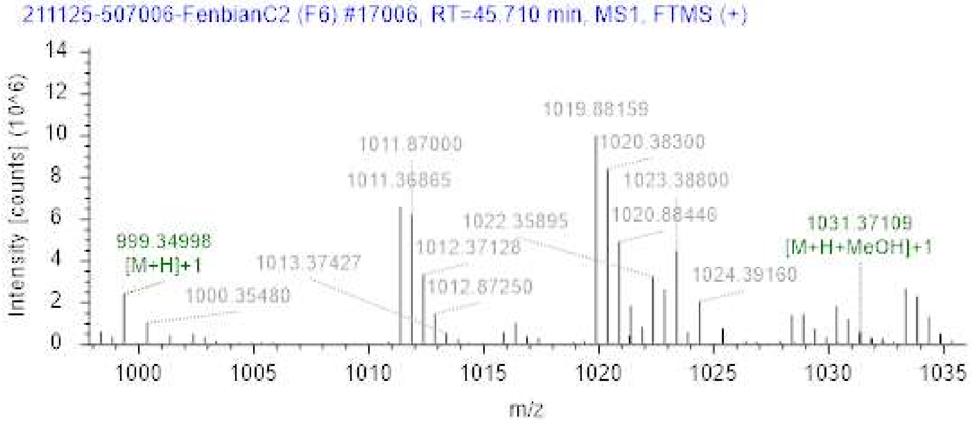

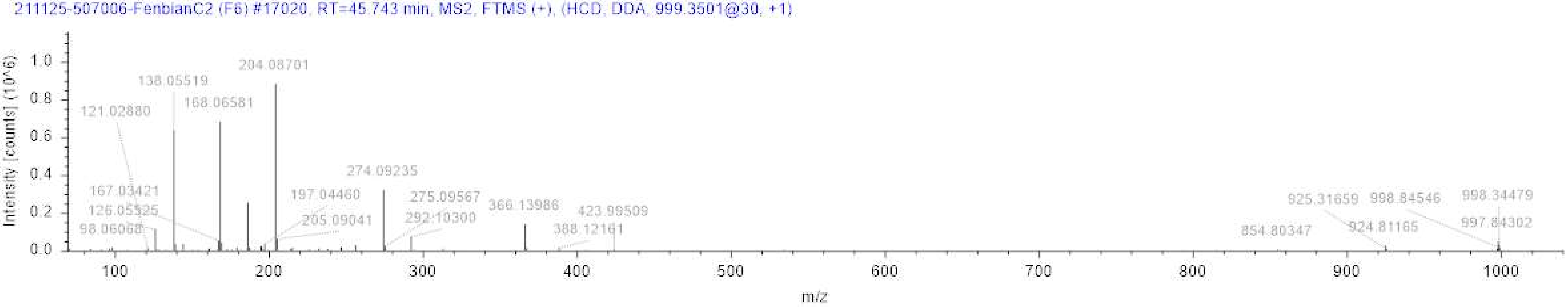

© Reported with Compound Discoverer 3.3

Compounds

Structure	Name	RT [min]	Formula	Calc. MW	Areas
n/a		43.23	n/a	998.34092	4.08e7; 1.95e7

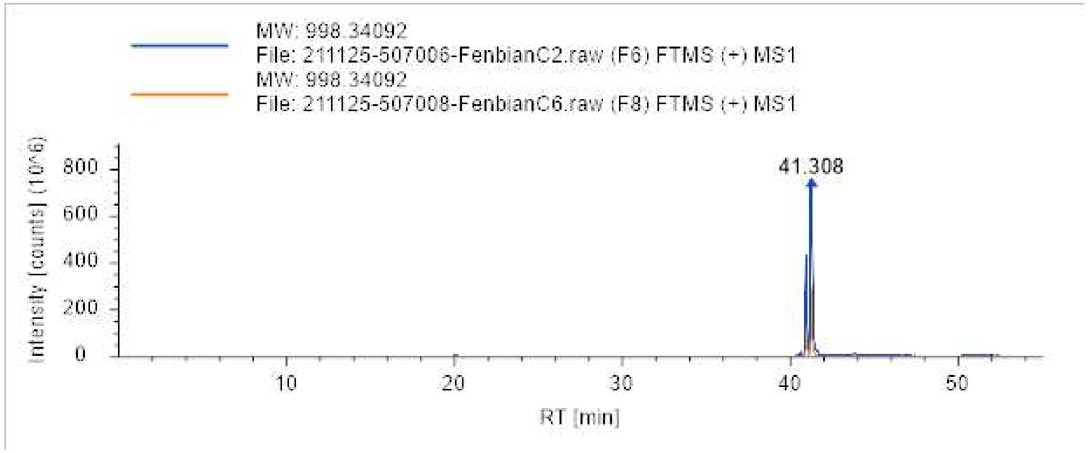

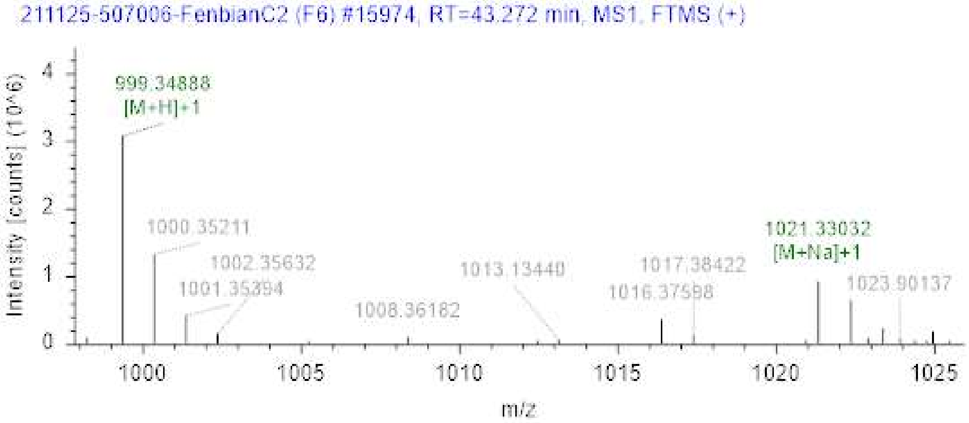

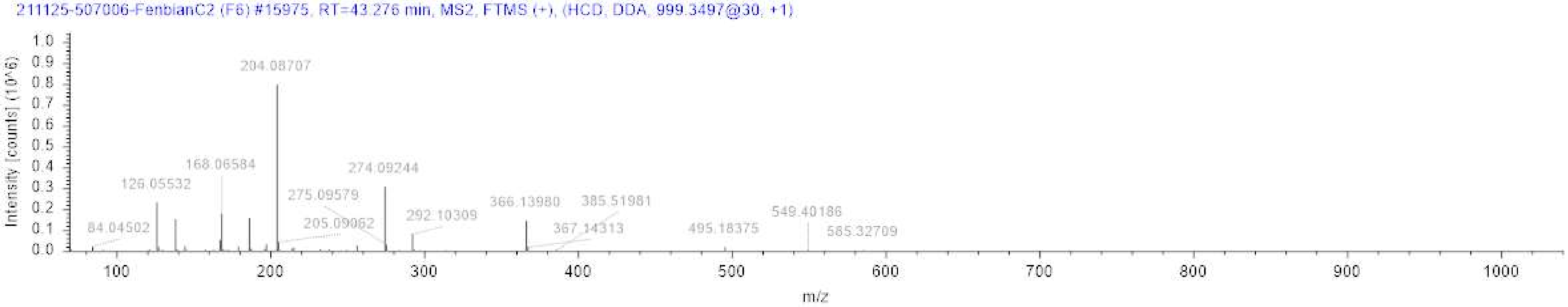

© Reported with Compound Discoverer 3.3

Structure	Name	RT [min]	Formula	Calc. MW	Areas
n/a		43.88	n/a	998.34099	8.61e6 1.83e7 4.93e6 6.28e6 1.43e8 7.57e7

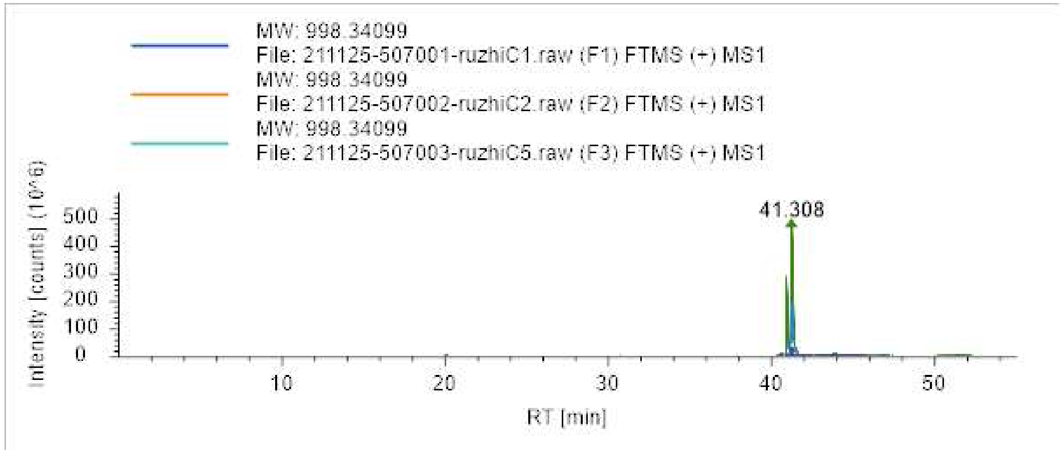

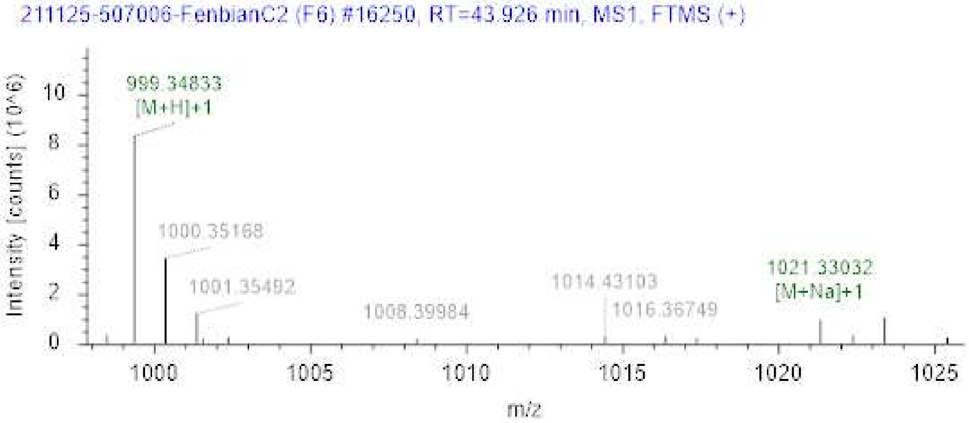

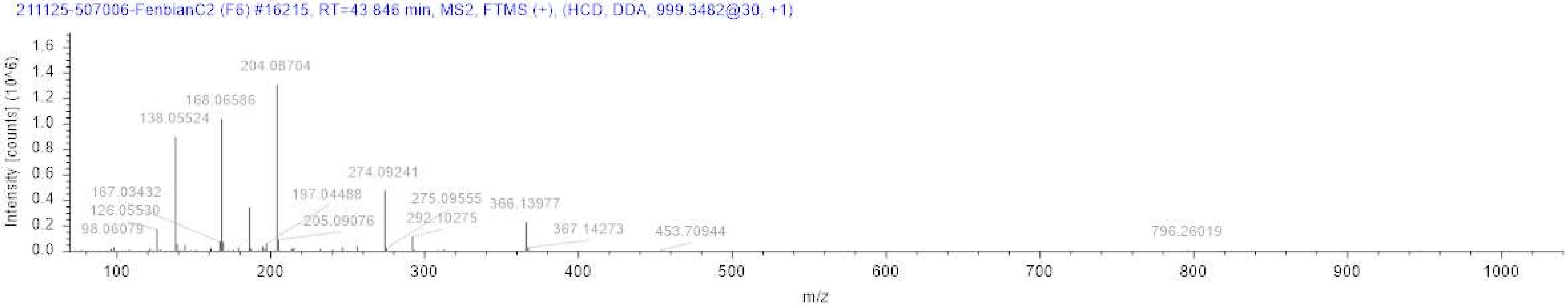

© Reported with Compound Discoverer 3.3

Compounds

Structure	Name	RT [min]	Formula	Calc. MW	Areas
n/a		40.98	n/a	998.34340	2.12e8 | 4.44e8 | 3.42e8 | 1.31e8 | | 1.03e10 | | 5.45e9 |

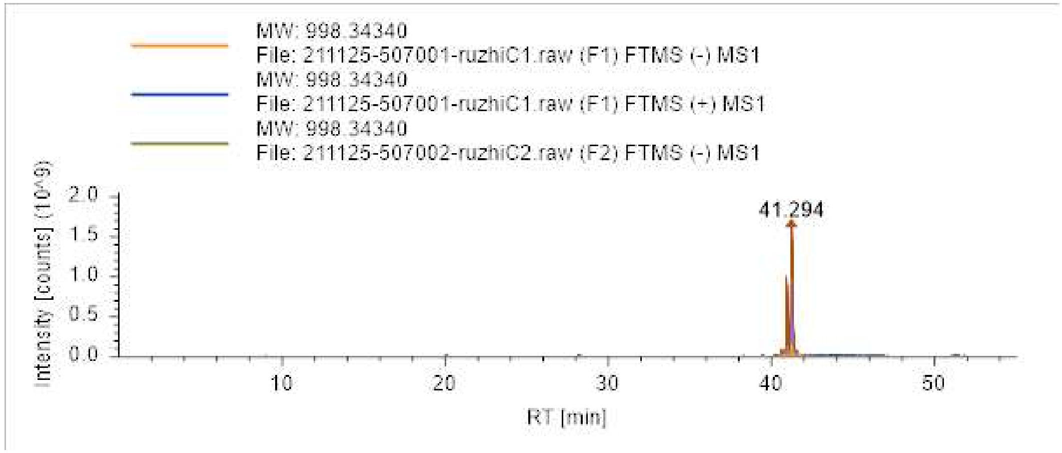

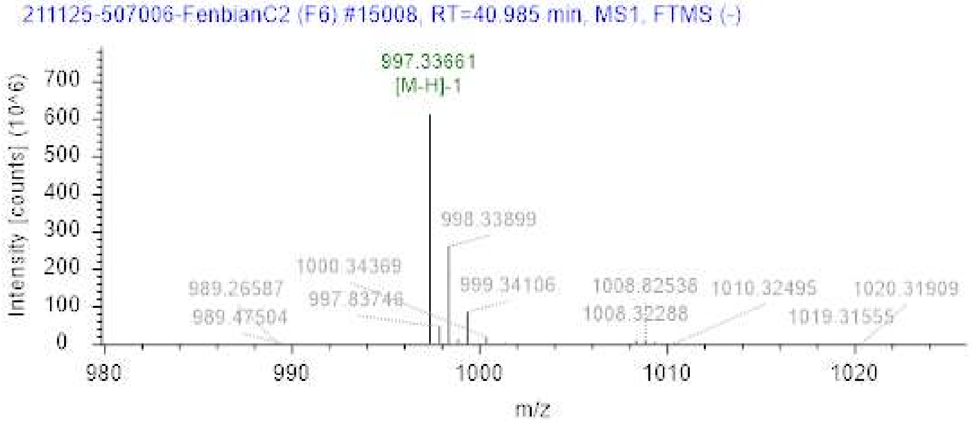

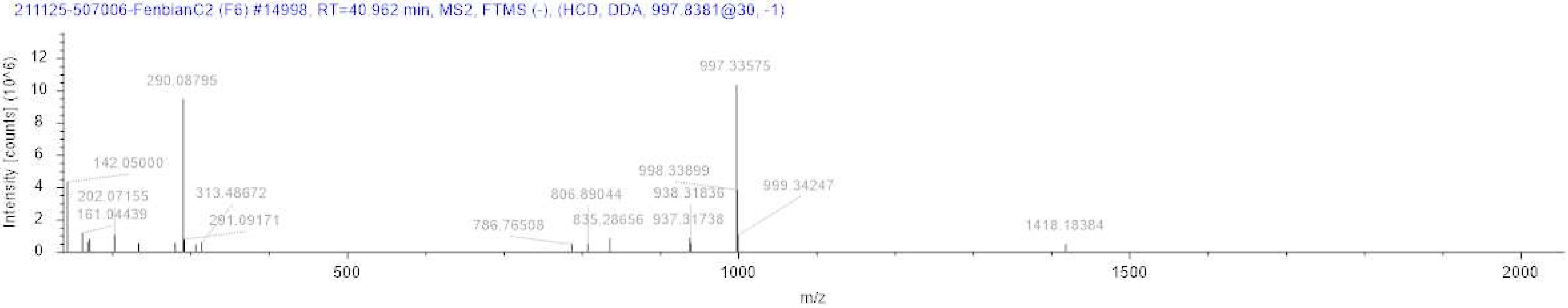

© Reported with Compound Discoverer 3.3

Compounds

Structure	Name	RT [min]	Formula	Calc. MW	Areas
n/a		44.94	n/a	999.34490	1.18e7

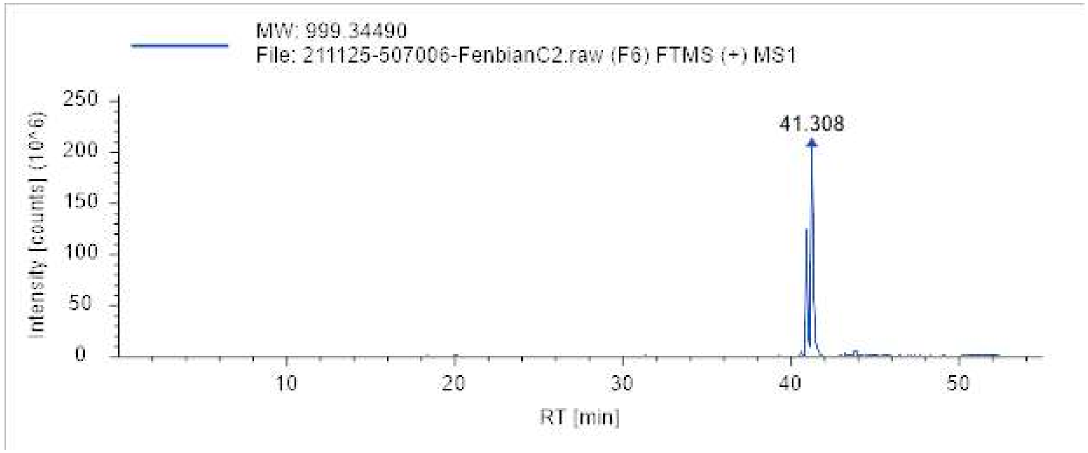

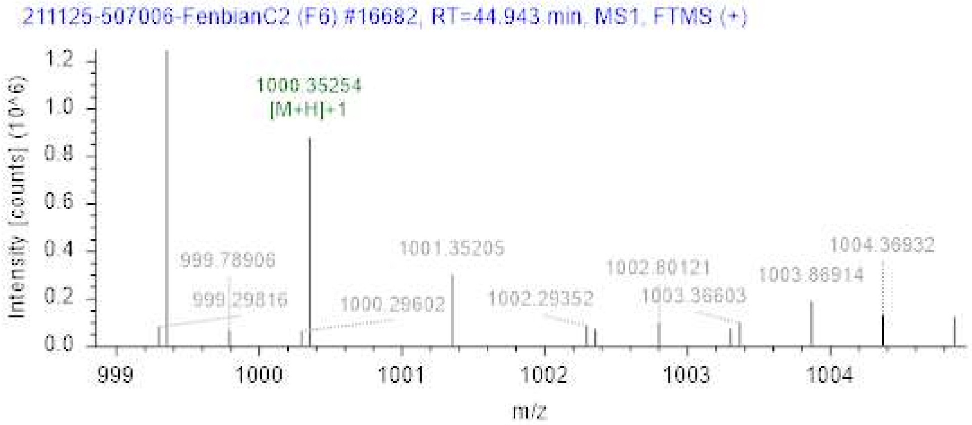

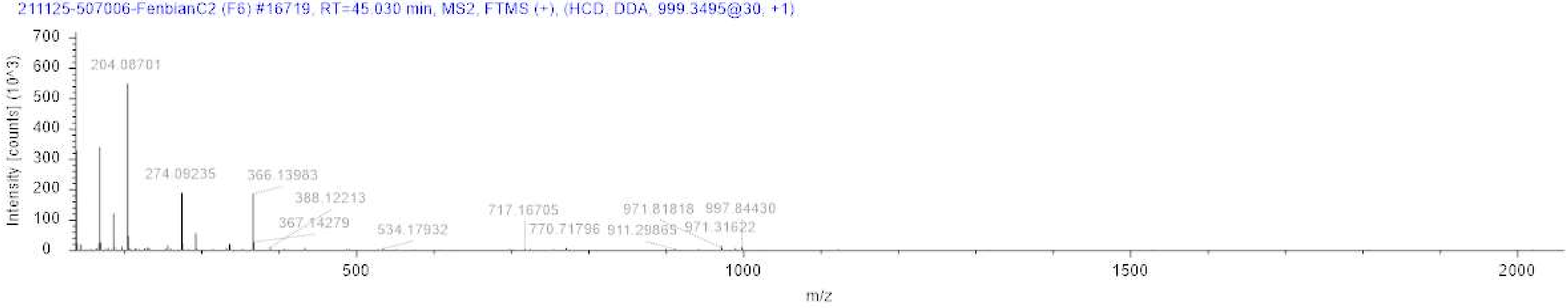

© Reported with Compound Discoverer 3.3

Structure	Name	RT [min]	Formula	Calc. MW	Areas
n/a		41.10	n/a	999.36003	8.81e6

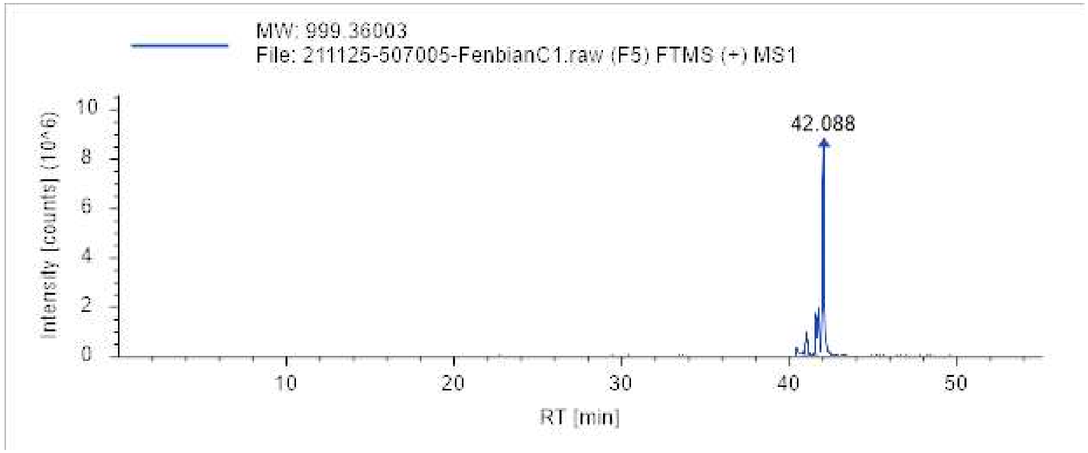

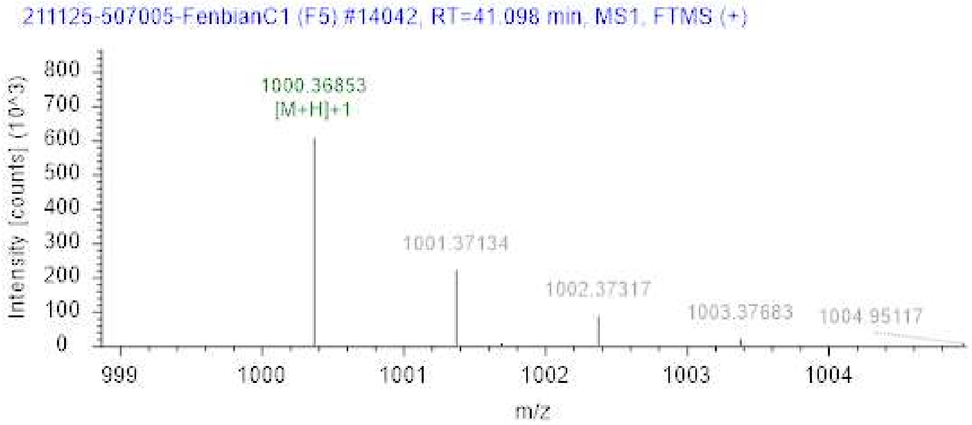

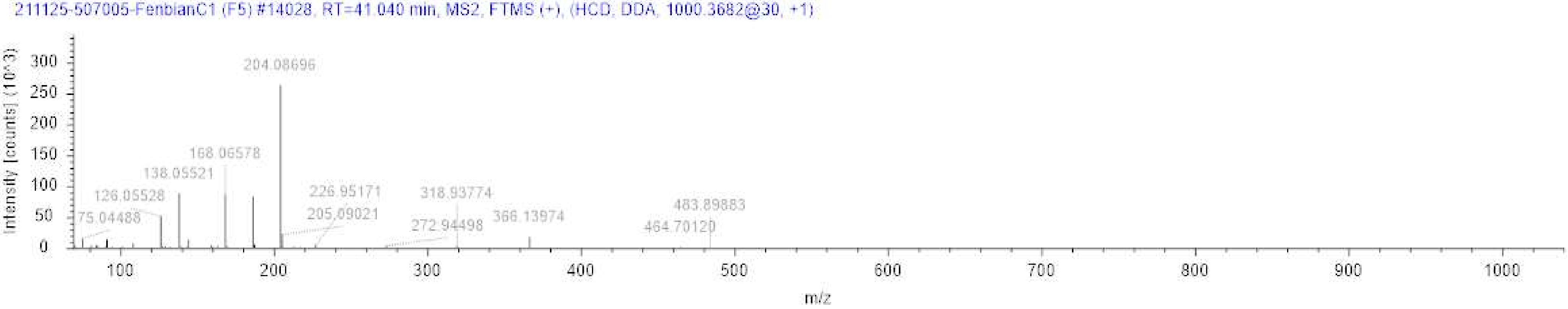

© Reported with Compound Discoverer 3.3

Structure	Name	RT [min]	Formula	Calc. MW	Areas
n/a		44.78	n/a	999.36022	2.51e7

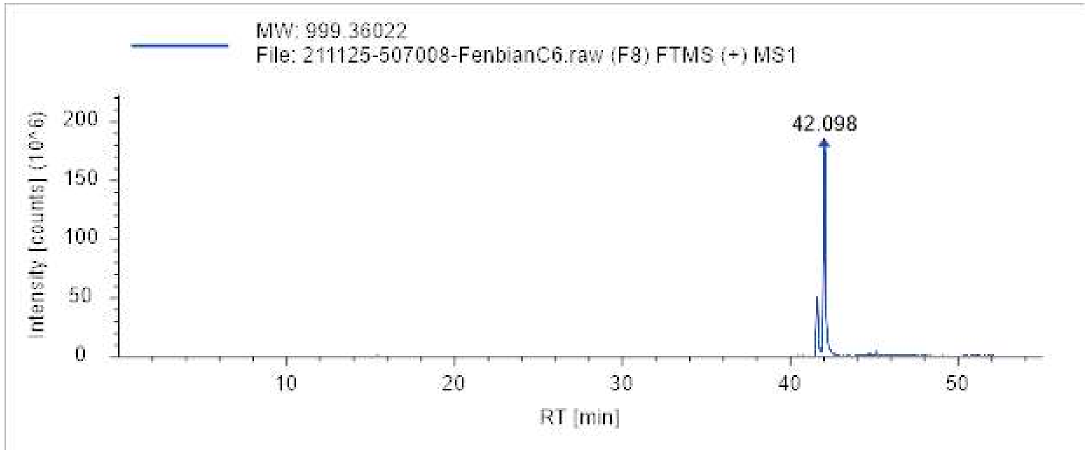

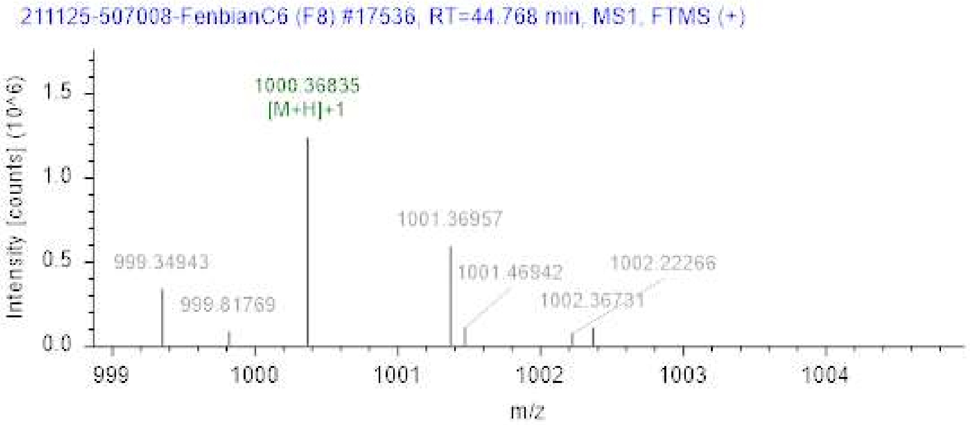

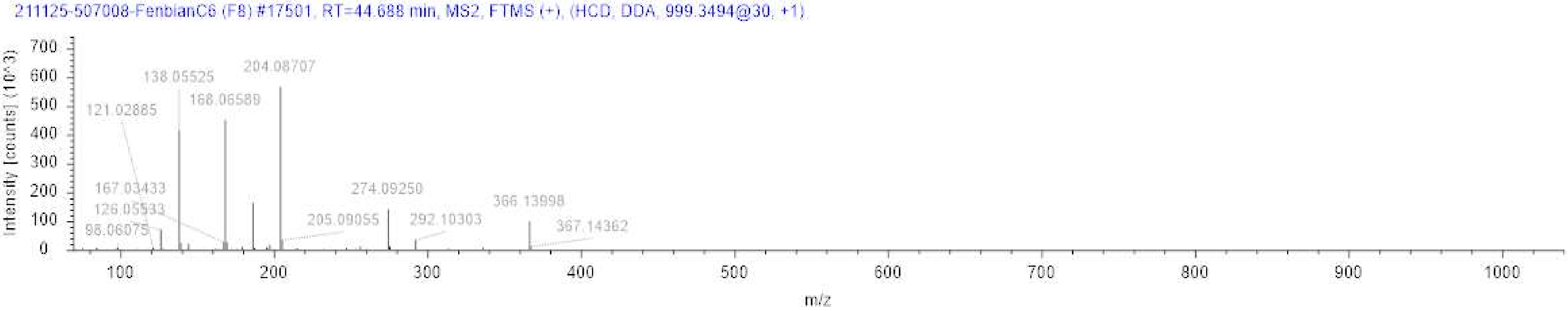

© Reported with Compound Discoverer 3.3

Compounds

Structure	Name	RT [min]	Formula	Calc. MW	Areas
n/a		42.09	n/a	999.36352	4.69e9 6.85e8 1.02e10 5.09e9 1.55e9 9.81e9 4.39e10

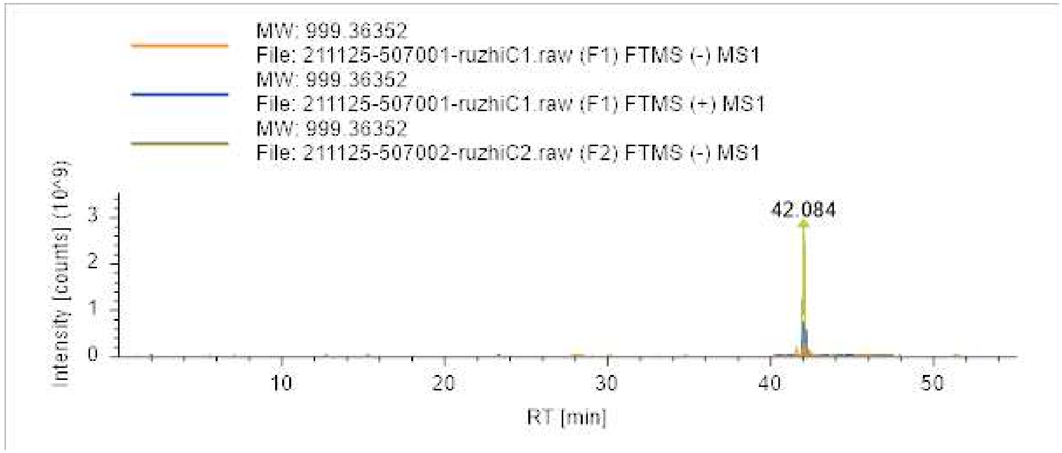

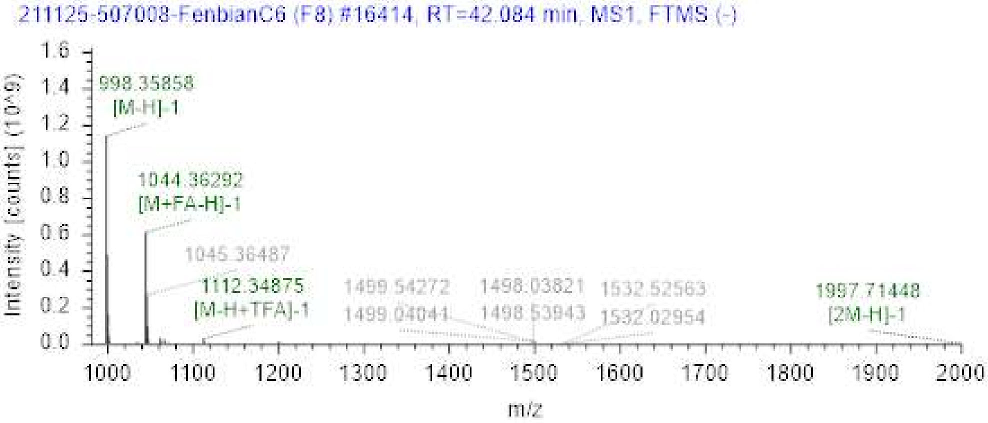

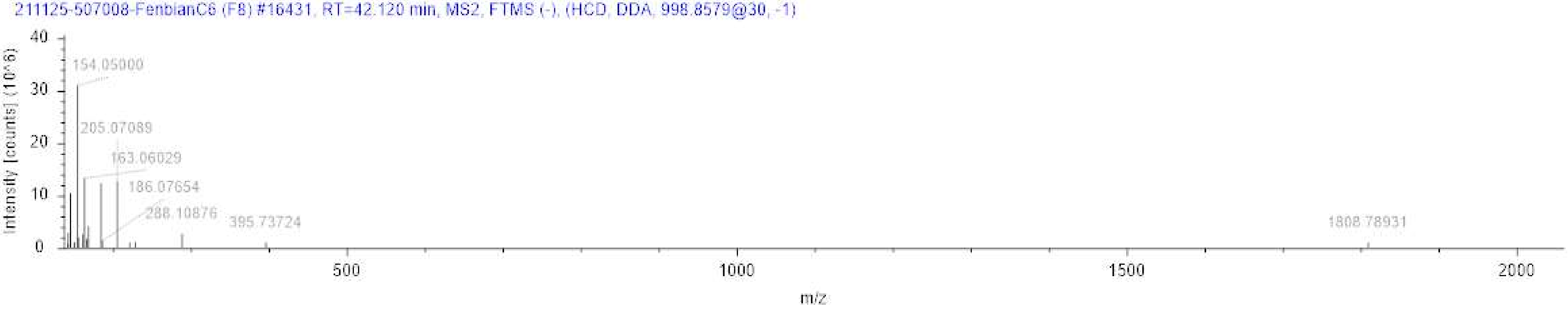

© Reported with Compound Discoverer 3.3

Compounds

Structure	Name	RT [min]	Formula	Calc. MW	Areas
n/a		42.19	n/a	1000.36518	6.24e7 6.31e8

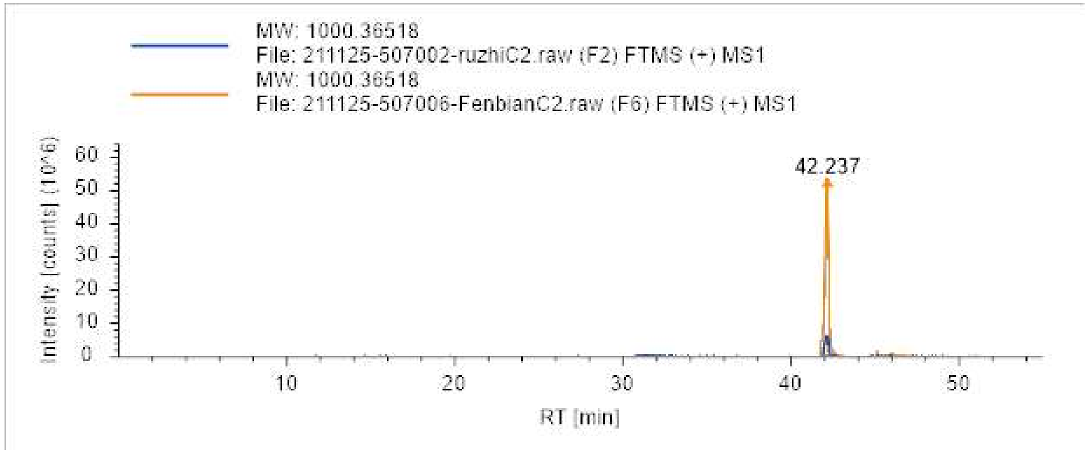

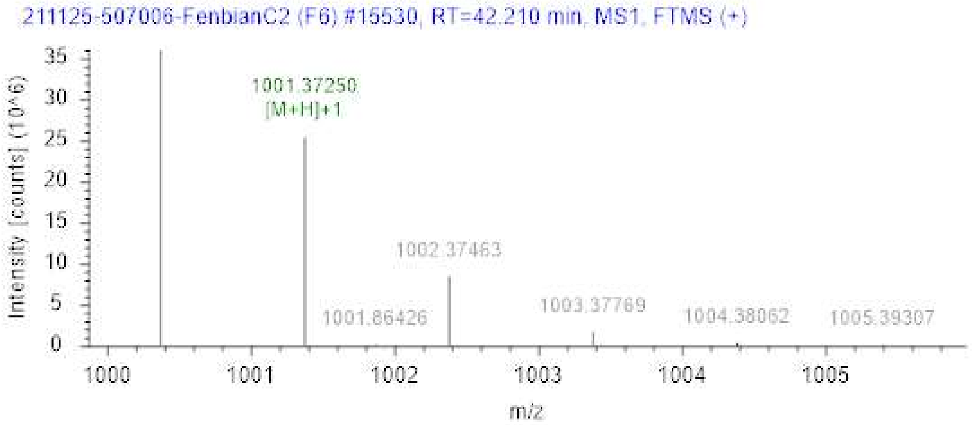

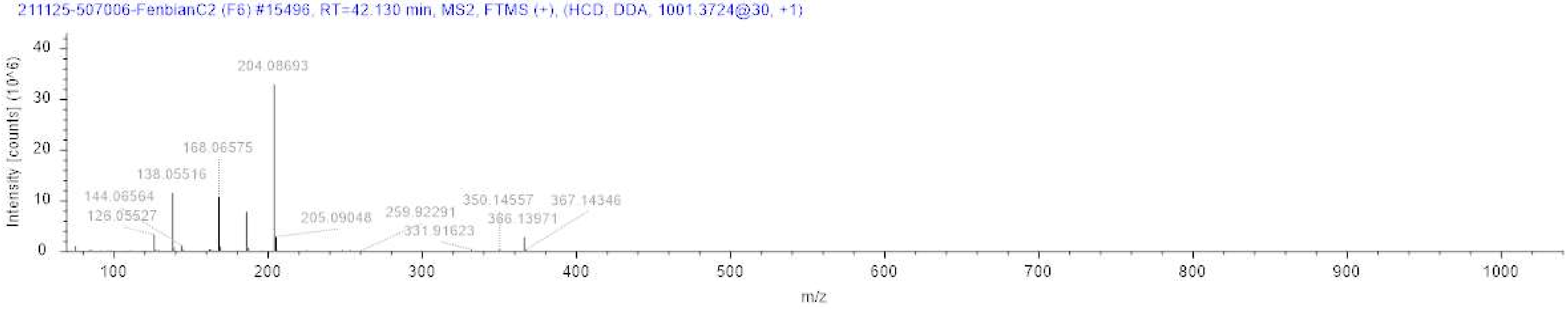

© Reported with Compound Discoverer 3.3

Structure	Name	RT [min]	Formula	Calc. MW	Areas
n/a		43.97	n/a	1015.35749	1.21e7

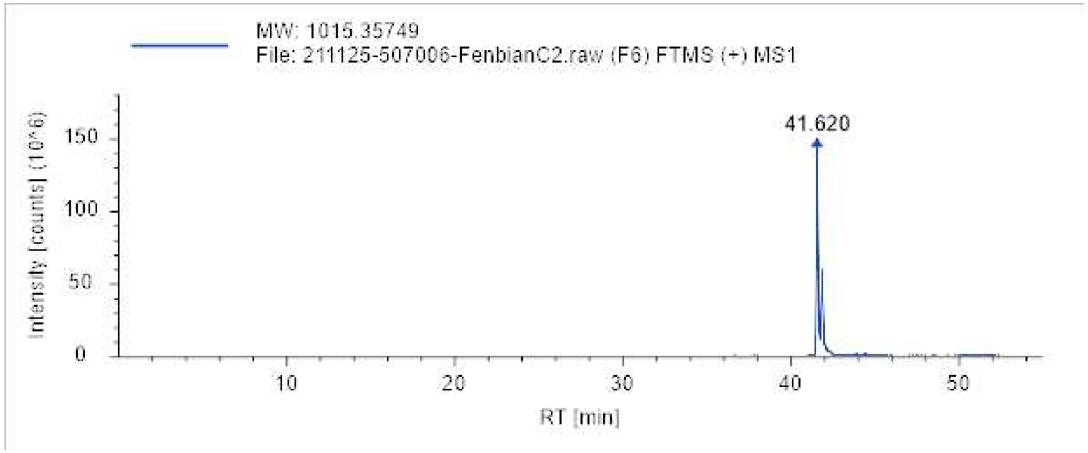

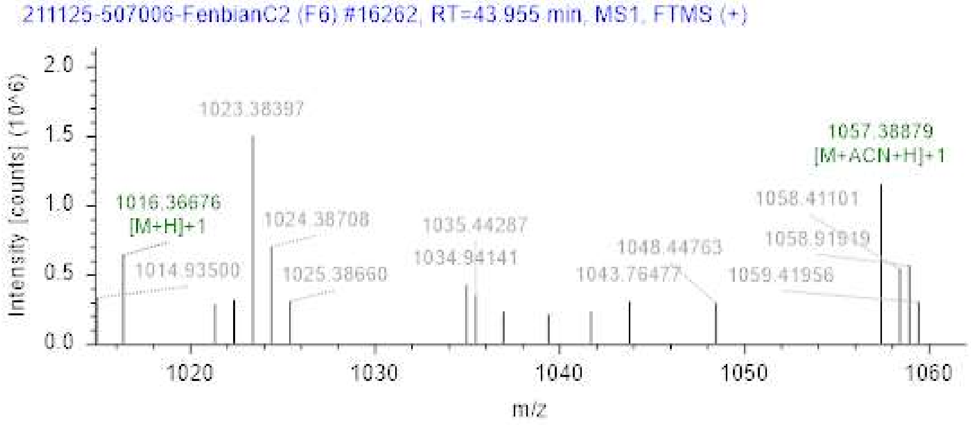

© Reported with Compound Discoverer 3.3

Compounds

Structure	Name	RT [min]	Formula	Calc. MW	Areas
n/a		42.33	n/a	1015.35874	8.92e7 / 1.20e8 / 3.33e7 / 2.53e7 / / 1.47e8 / / 3.88e8 /

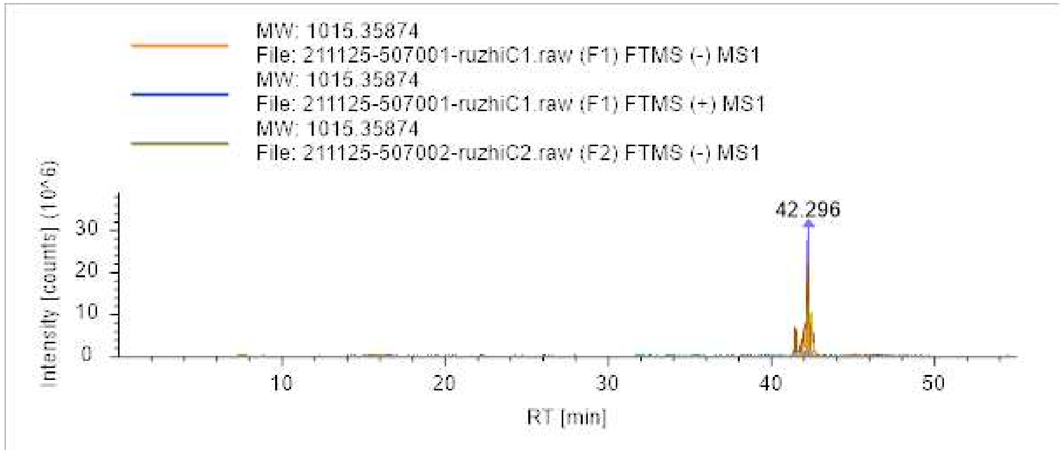

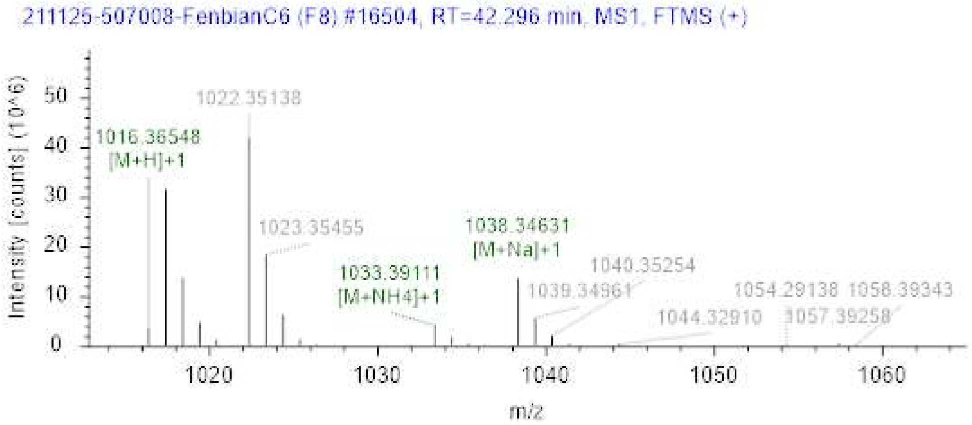

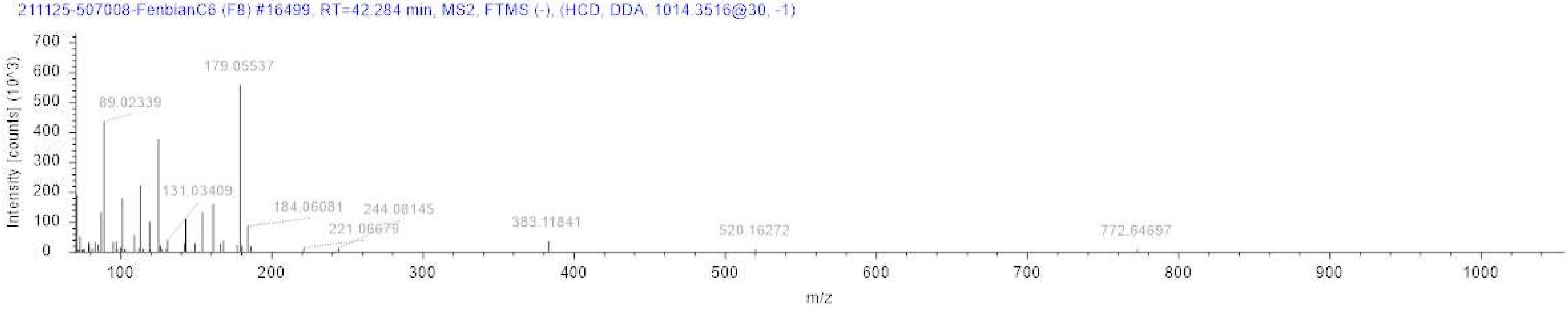

© Reported with Compound Discoverer 3.3

Structure	Name	RT [min]	Formula	Calc. MW	Areas
n/a		41.51	n/a	1015.35942	1.73e7; 1.04e8

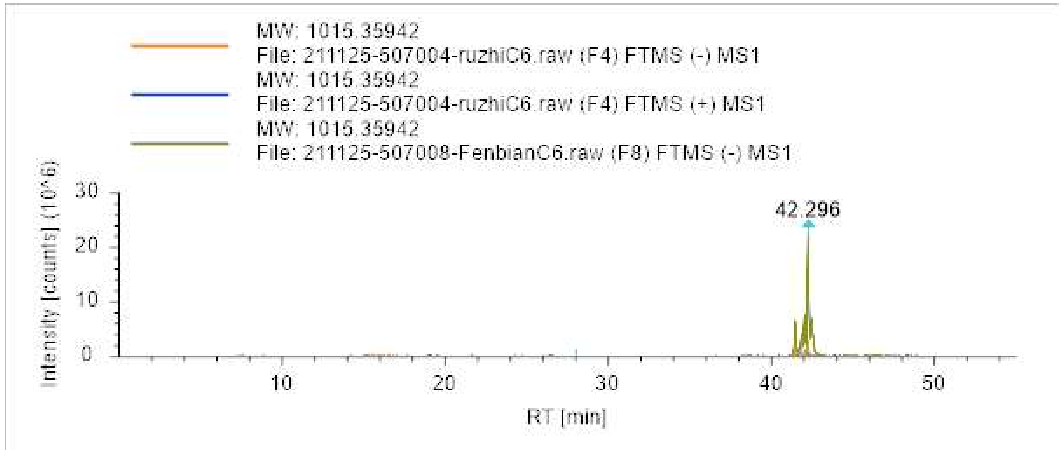

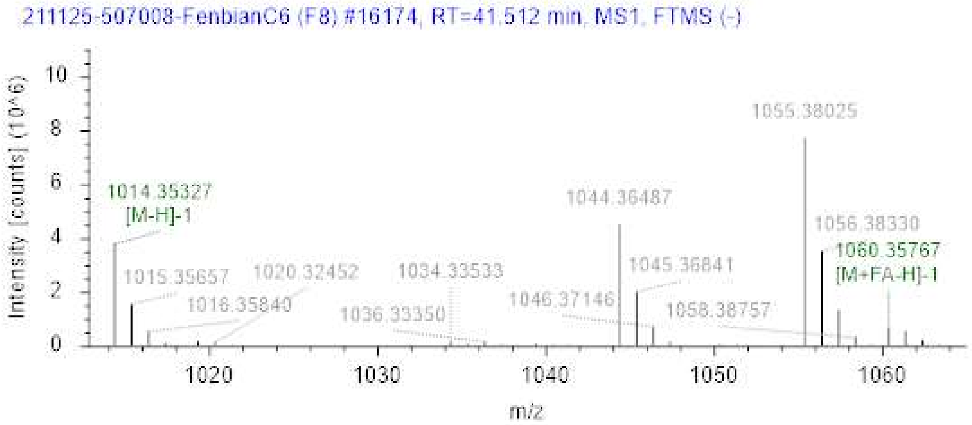

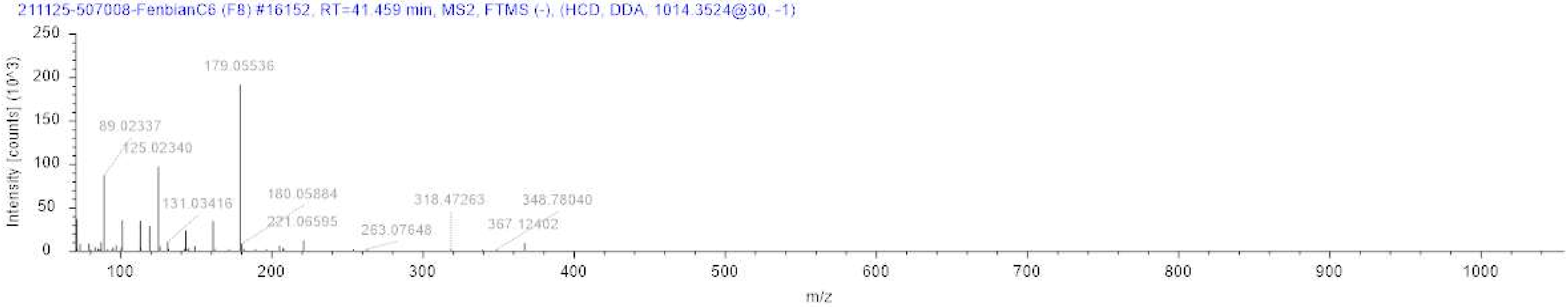

© Reported with Compound Discoverer 3.3

Structure	Name	RT [min]	Formula	Calc. MW	Areas
n/a		28.11	n/a	1017.37471	1.36e7

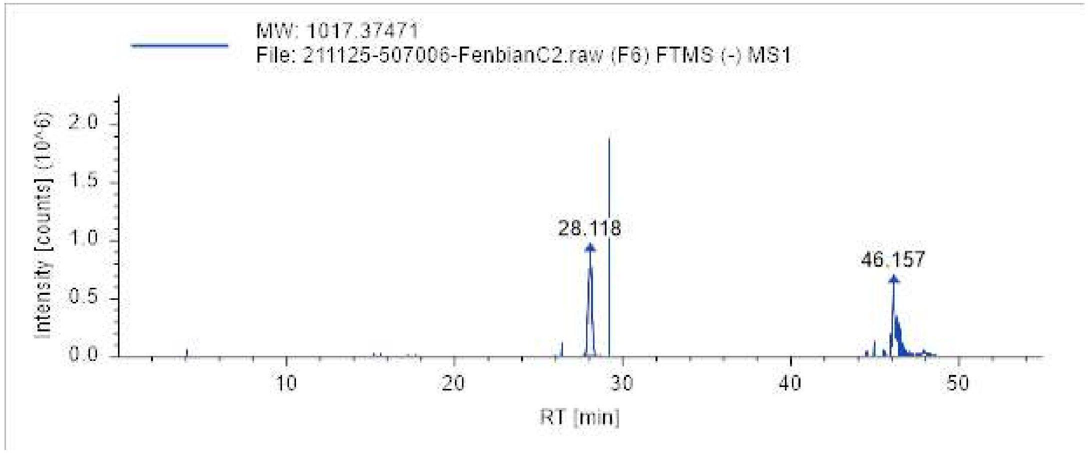

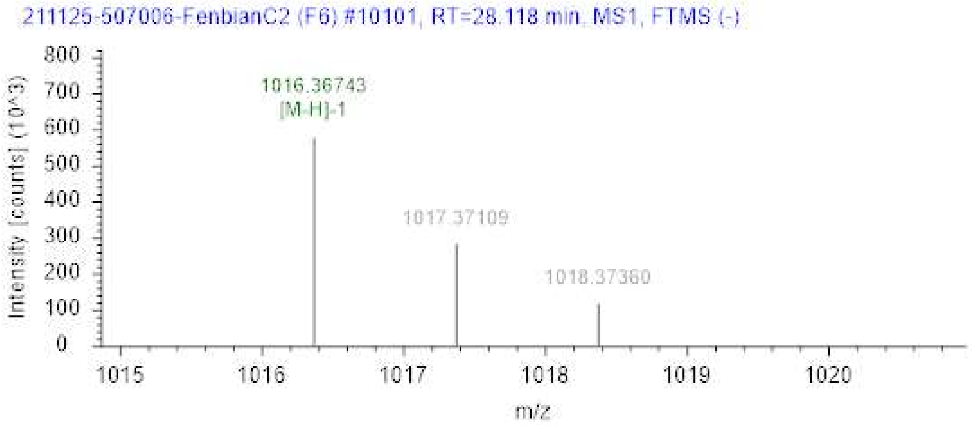

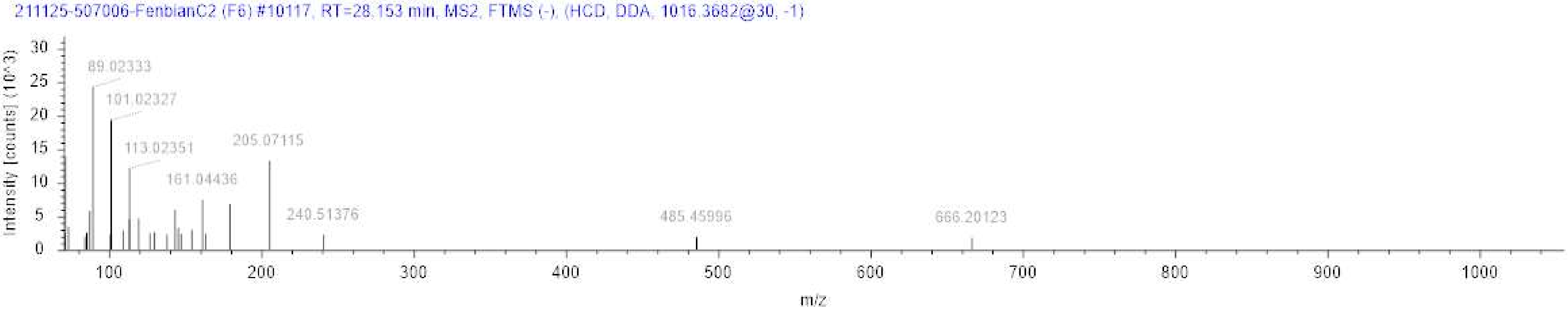

© Reported with Compound Discoverer 3.3

Compounds

Structure	Name	RT [min]	Formula	Calc. MW	Areas
n/a		43.80	n/a	1023.38042	5.11e6

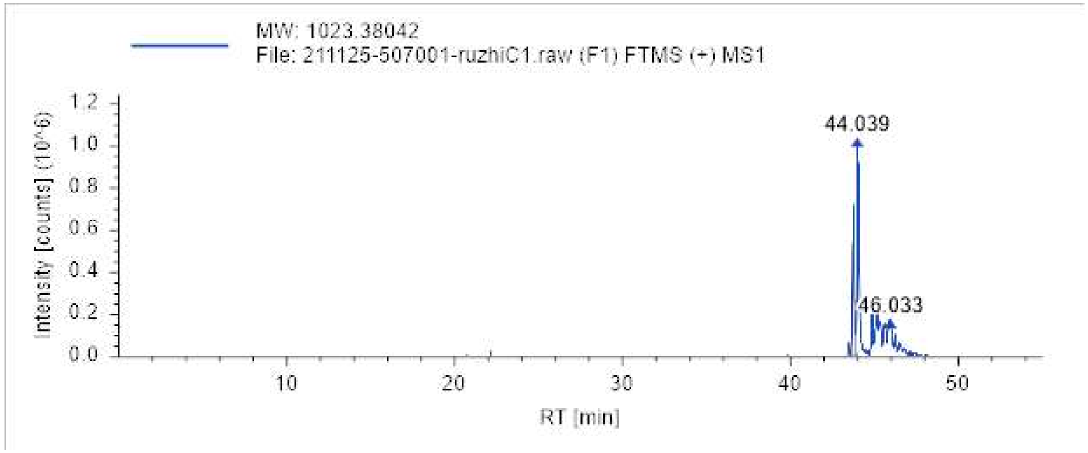

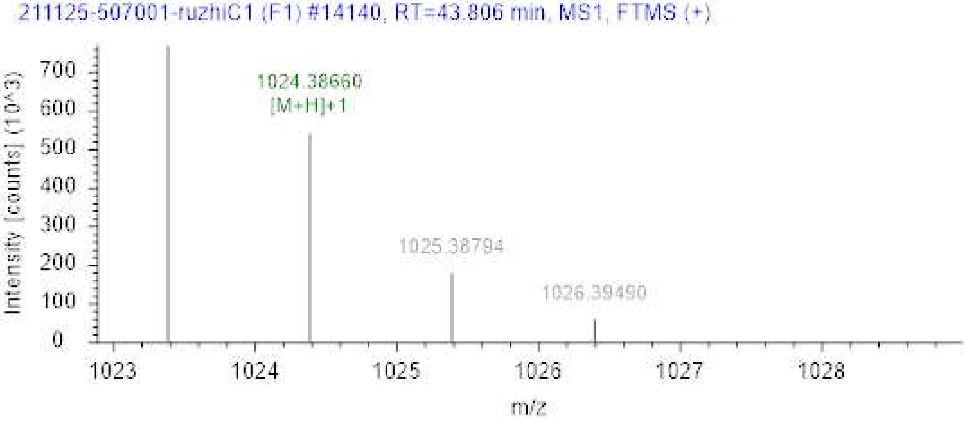

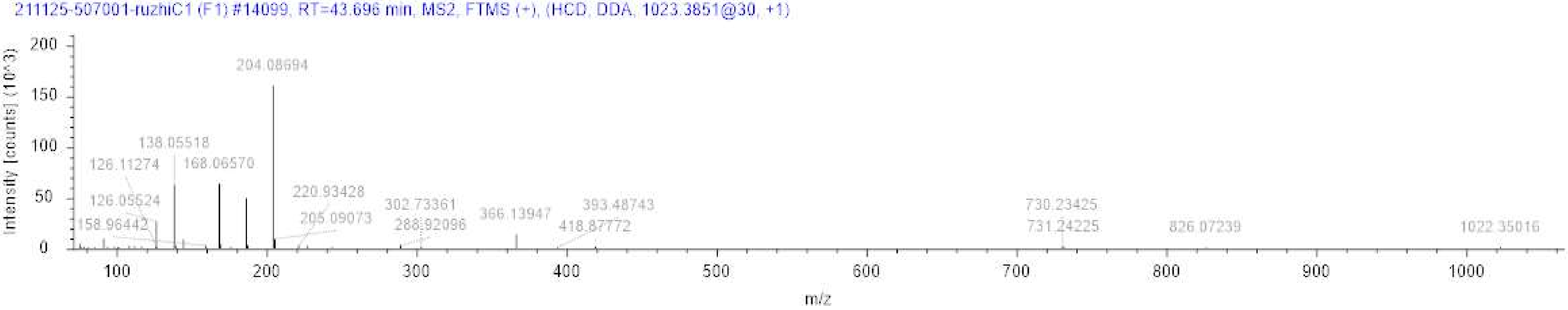

© Reported with Compound Discoverer 3.3

Compounds

Structure	Name	RT [min]	Formula	Calc. MW	Areas
n/a		45.44	n/a	1031.34920	4.17e7

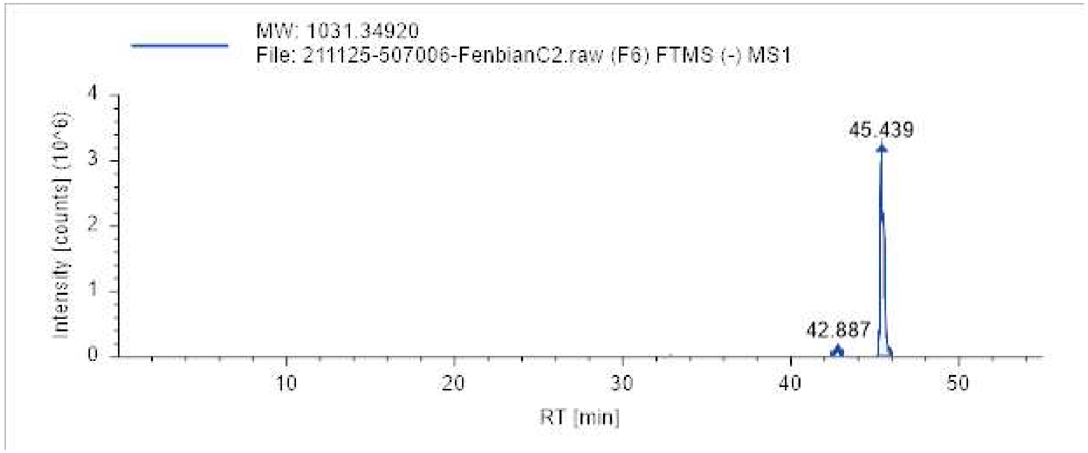

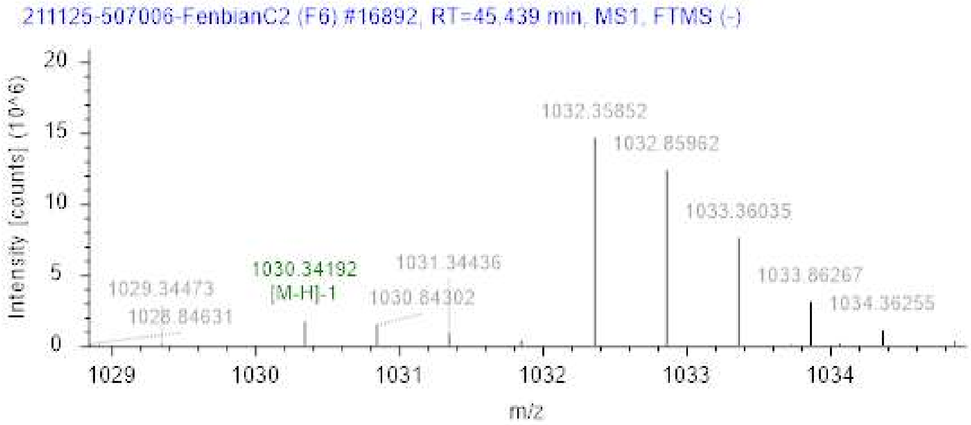

© Reported with Compound Discoverer 3.3

Compounds

Structure	Name	RT [min]	Formula	Calc. MW	Areas
n/a		29.62	n/a	1033.37019	4.13e7

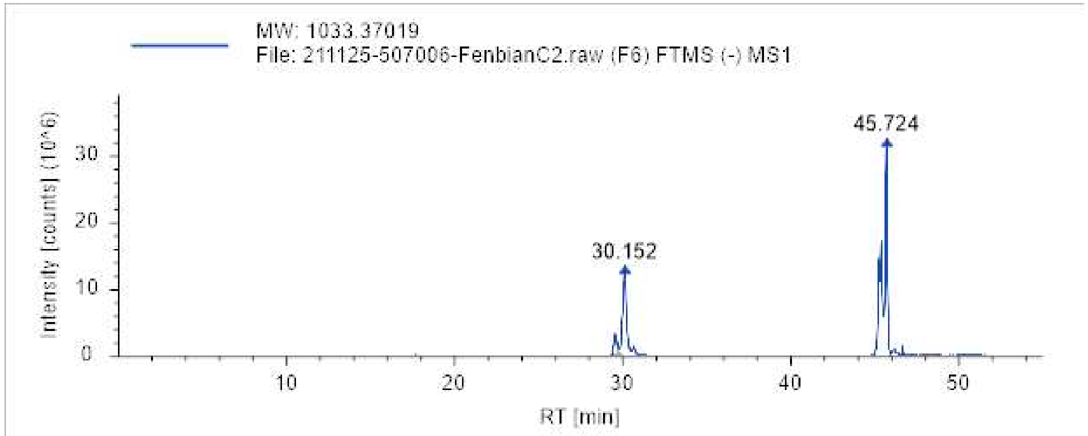

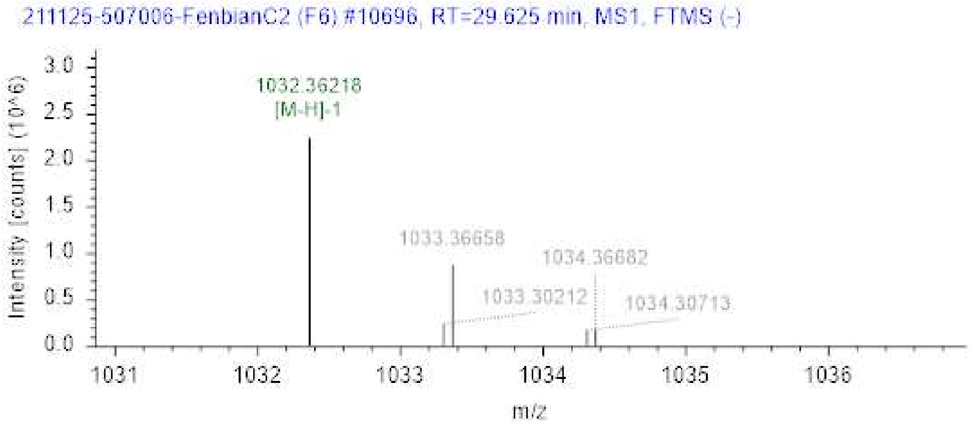

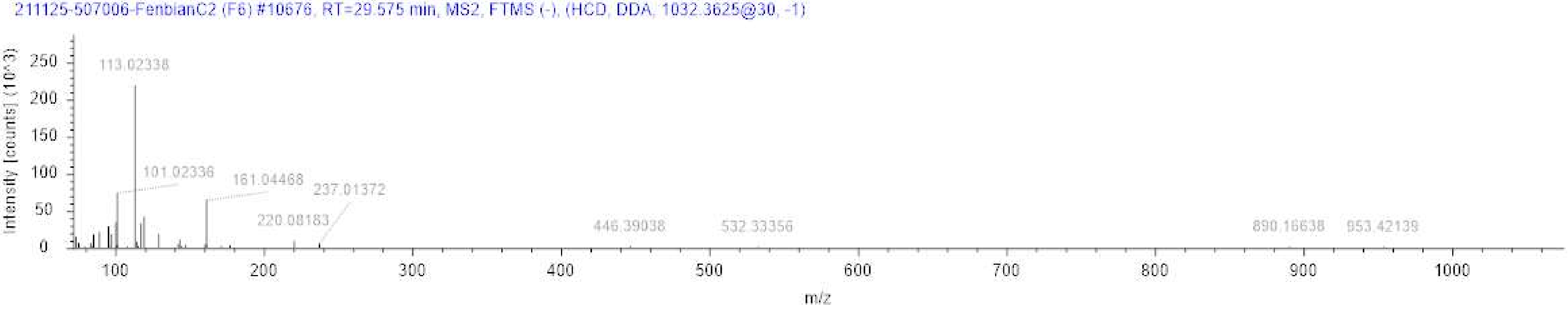

© Reported with Compound Discoverer 3.3

Compounds

Structure	Name	RT [min]	Formula	Calc. MW	Areas
n/a		40.35	n/a	1039.37153	9.91e6

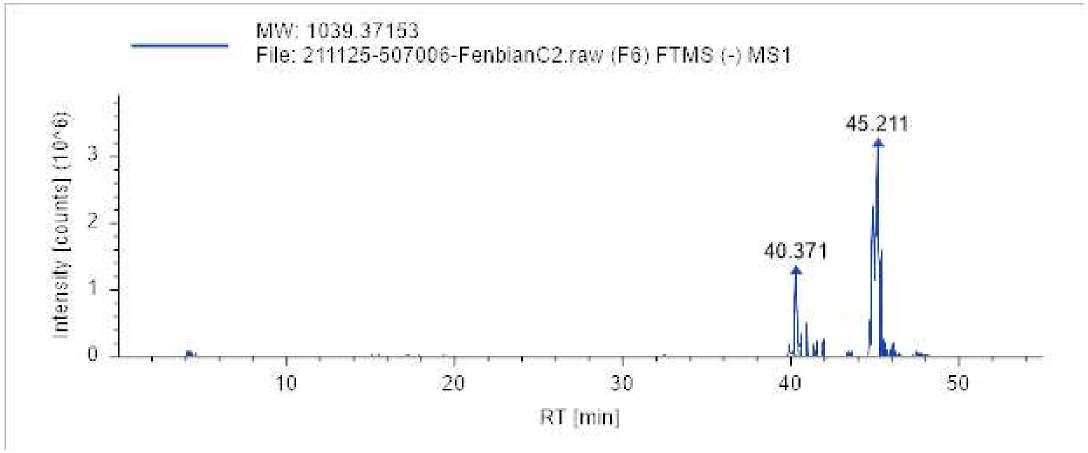

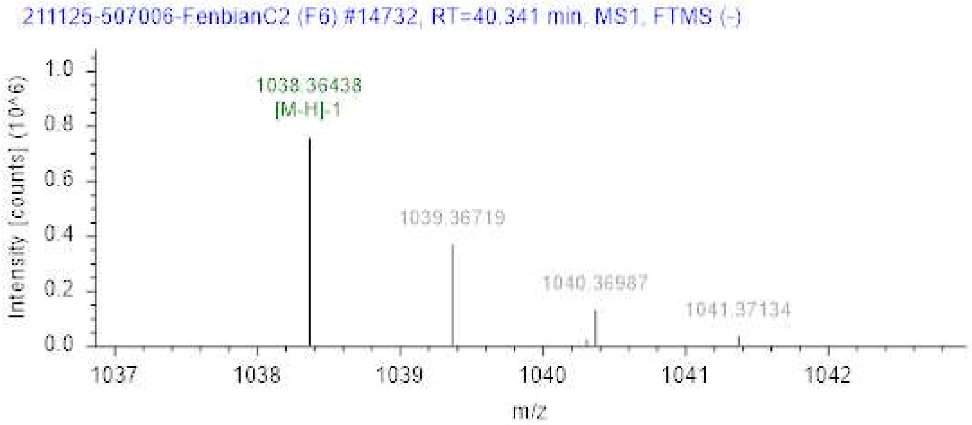

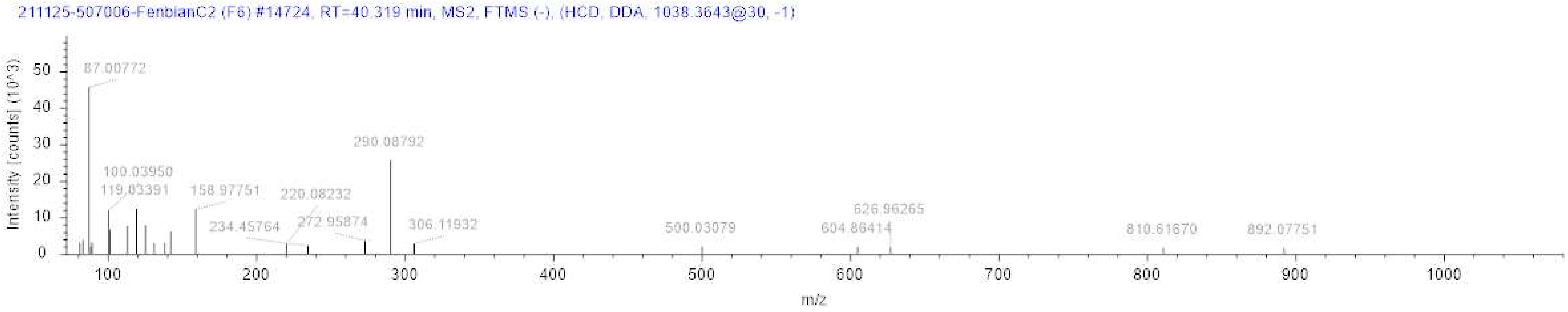

© Reported with Compound Discoverer 3.3

Structure	Name	RT [min]	Formula	Calc. MW	Areas
n/a		40.97	n/a	1040.38997	7.84e6

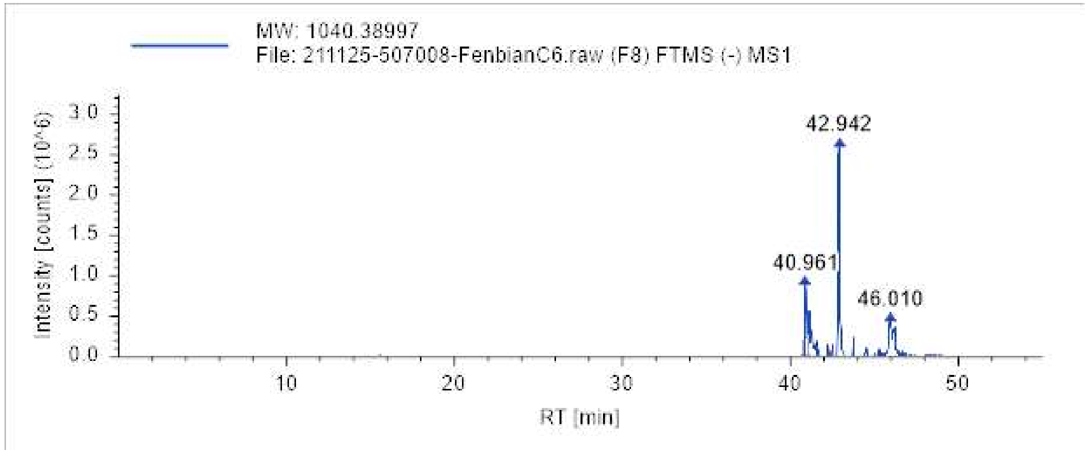

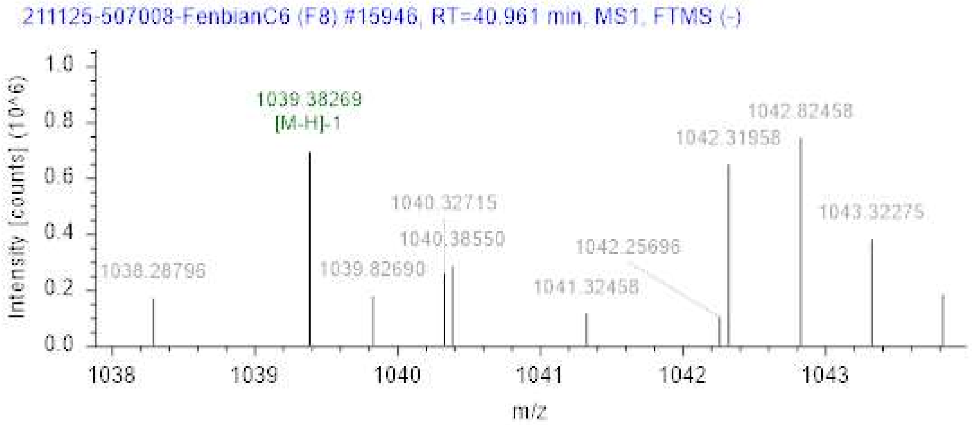

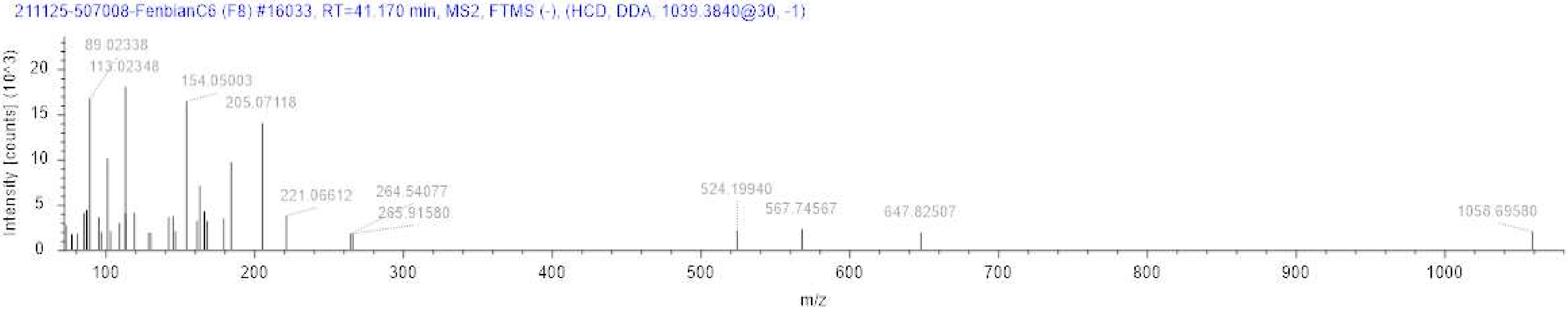

© Reported with Compound Discoverer 3.3

Structure	Name	RT [min]	Formula	Calc. MW	Areas
n/a		42.93	n/a	1040.39183	2.57e7

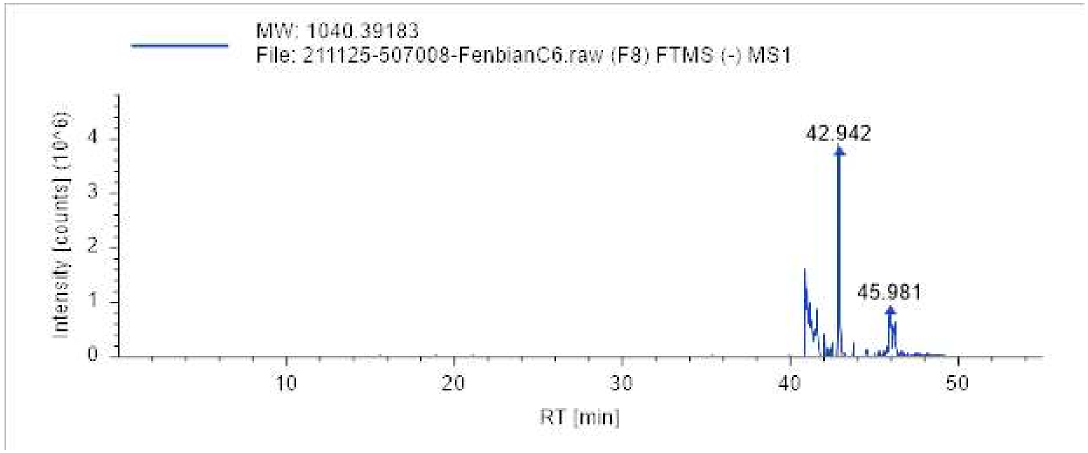

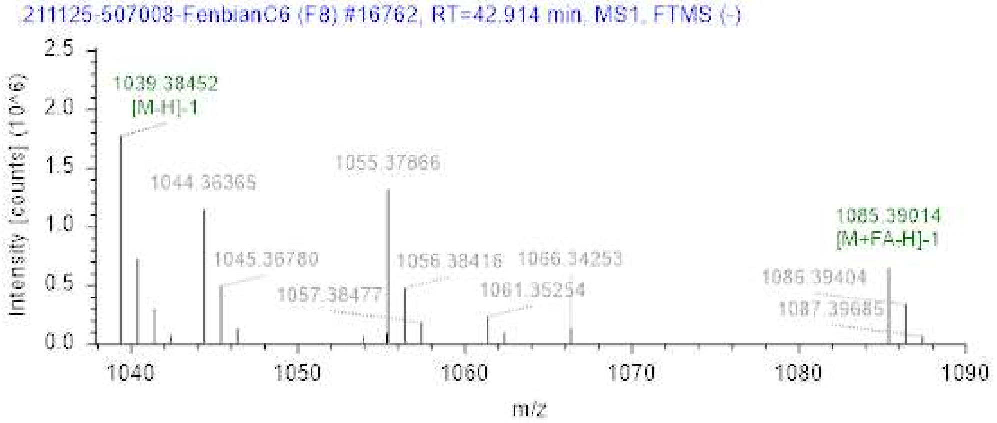

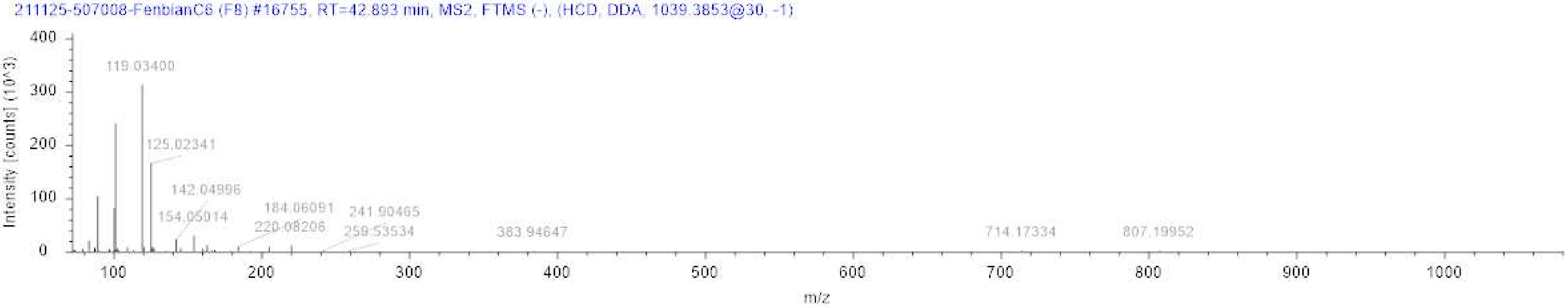

© Reported with Compound Discoverer 3.3

Compounds

Structure	Name	RT [min]	Formula	Calc. MW	Areas
n/a		44.47	n/a	1056.38260	3.08e7

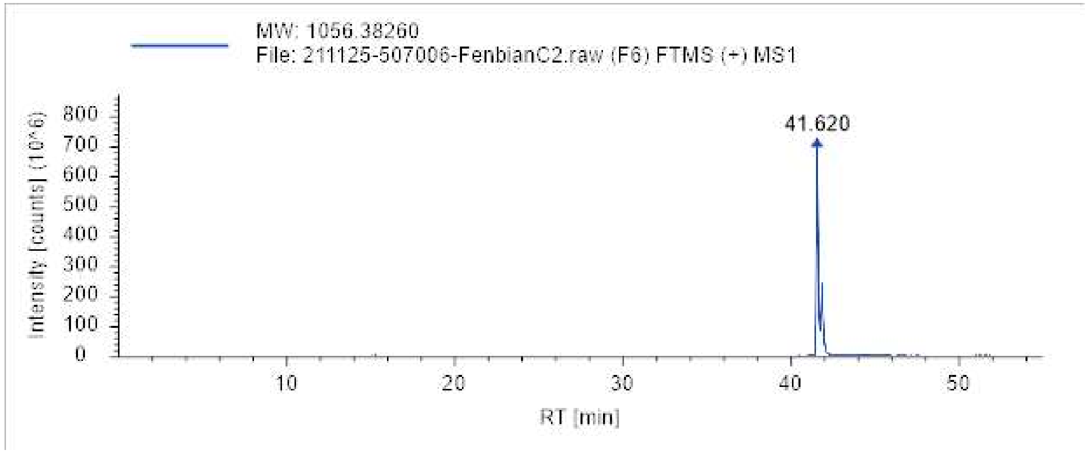

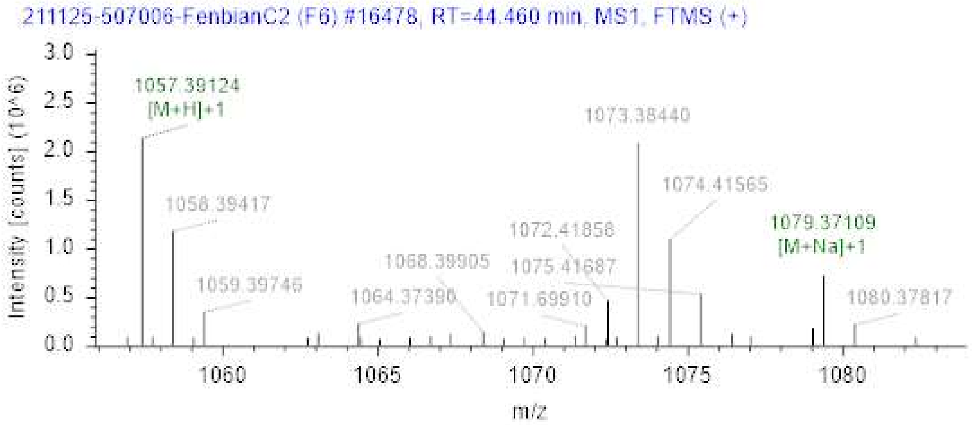

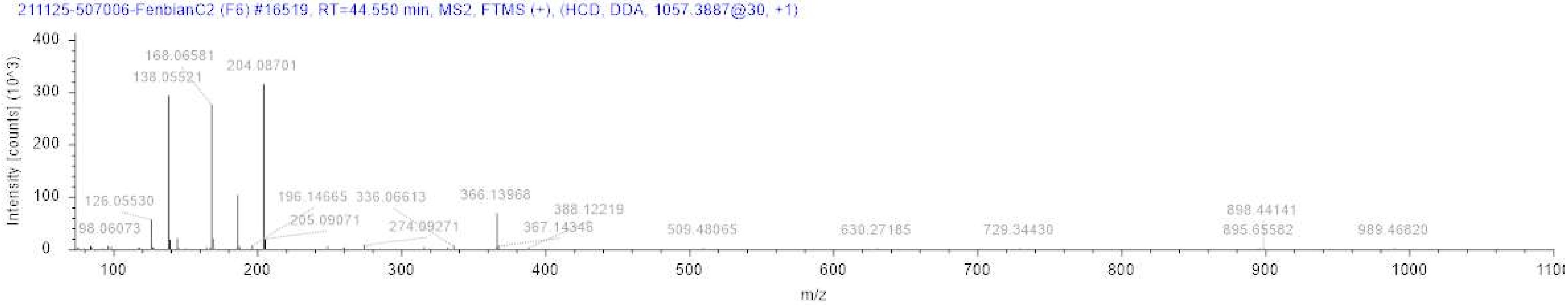

© Reported with Compound Discoverer 3.3

Structure	Name	RT [min]	Formula	Calc. MW	Areas
n/a		42.80	n/a	1056.38349	6.57e6 5.99e6 2.14e7

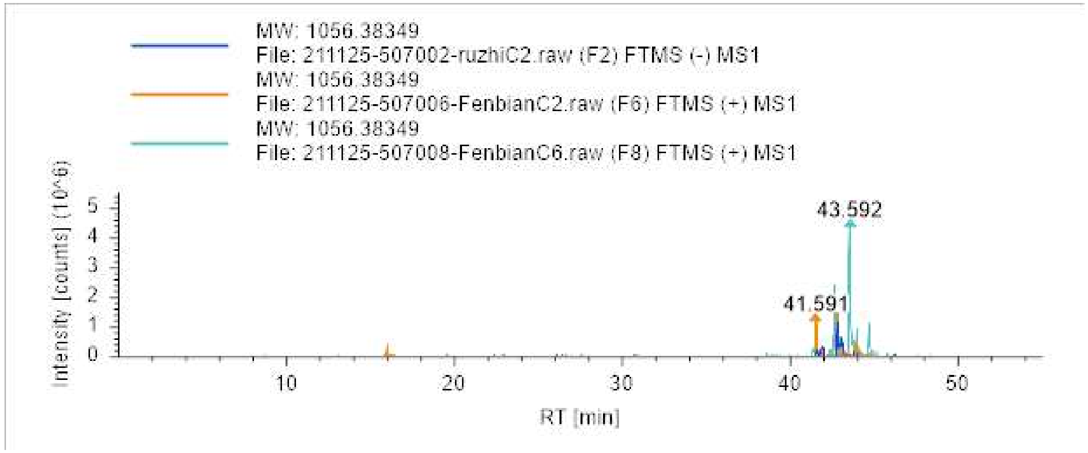

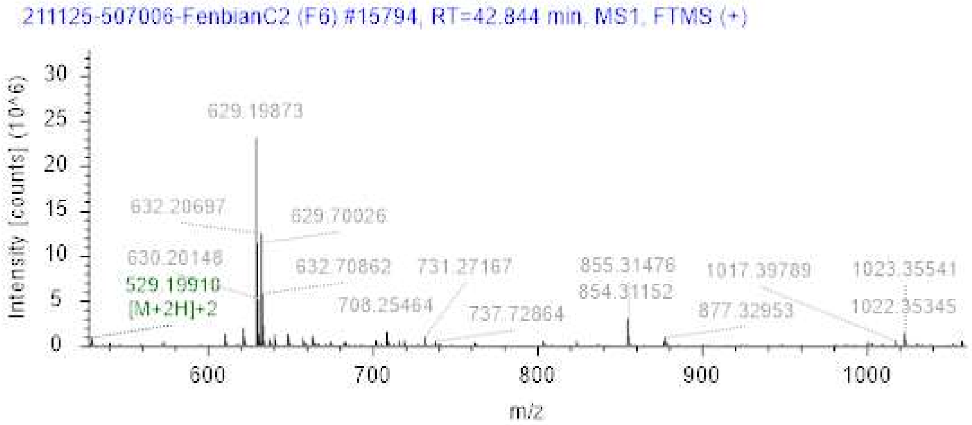

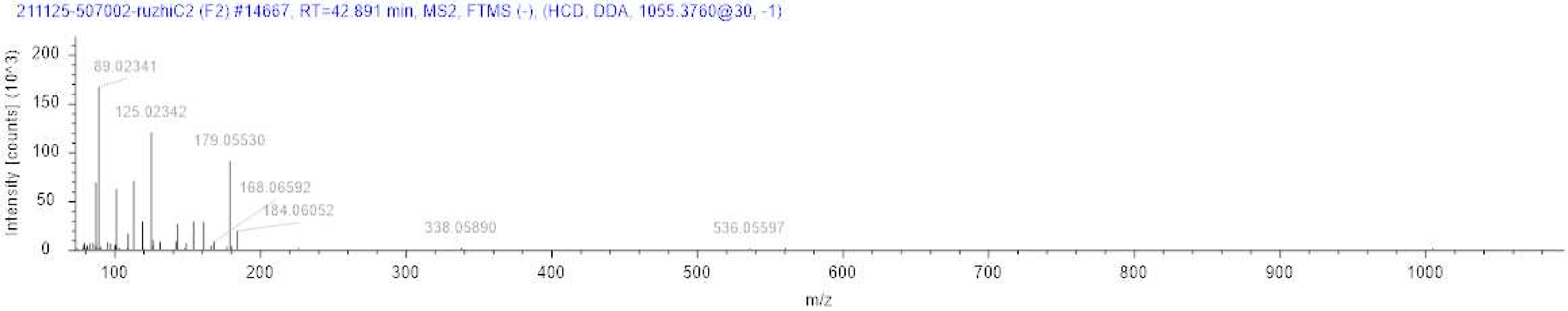

© Reported with Compound Discoverer 3.3

Compounds

Structure	Name	RT [min]	Formula	Calc. MW	Areas
n/a		43.58	n/a	1056.38393	3.23e7

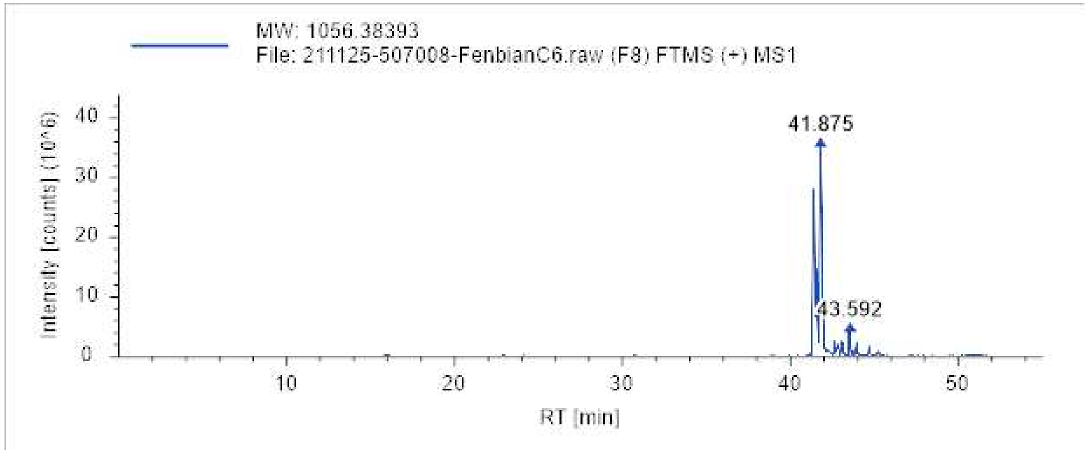

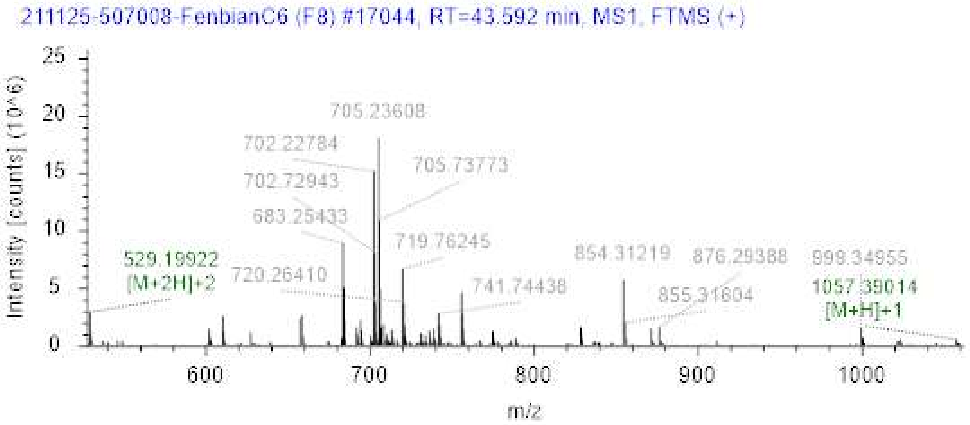

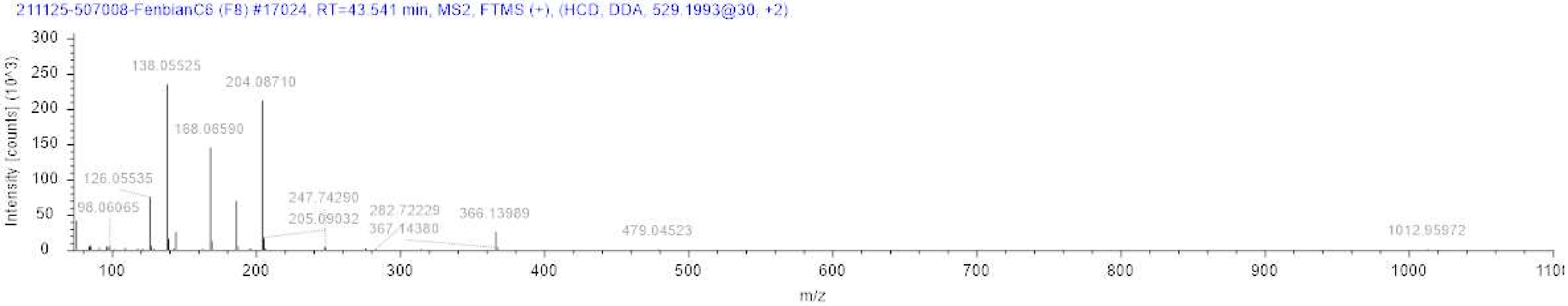

© Reported with Compound Discoverer 3.3

Compounds

Structure	Name	RT [min]	Formula	Calc. MW	Areas
n/a		41.88	n/a	1056.38402	1.06e7 9.15e8 1.57e9

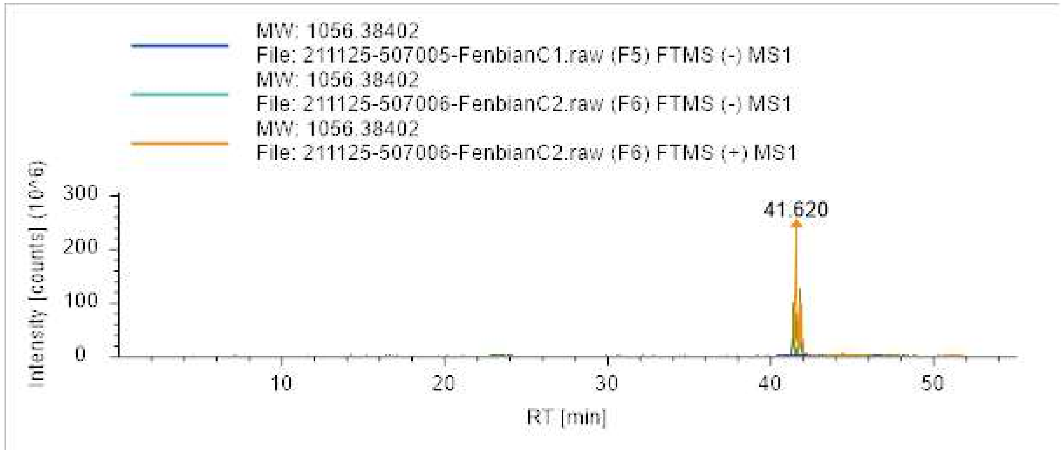

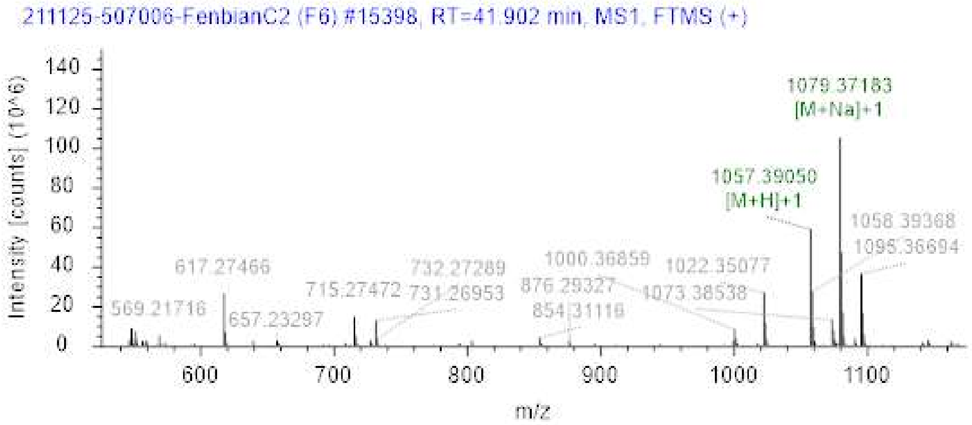

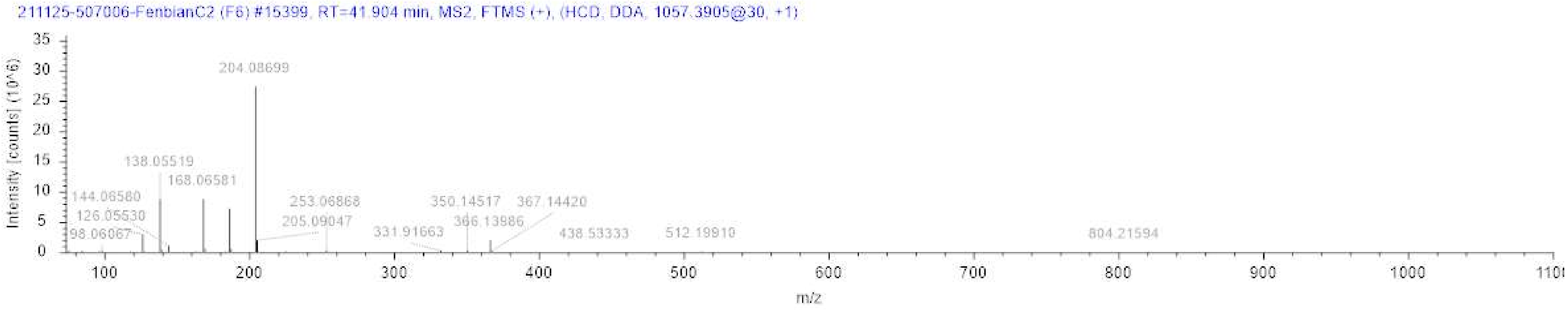

© Reported with Compound Discoverer 3.3

Compounds

Structure	Name	RT [min]	Formula	Calc. MW	Areas
n/a		43.59	n/a	1072.37749	8.64e6

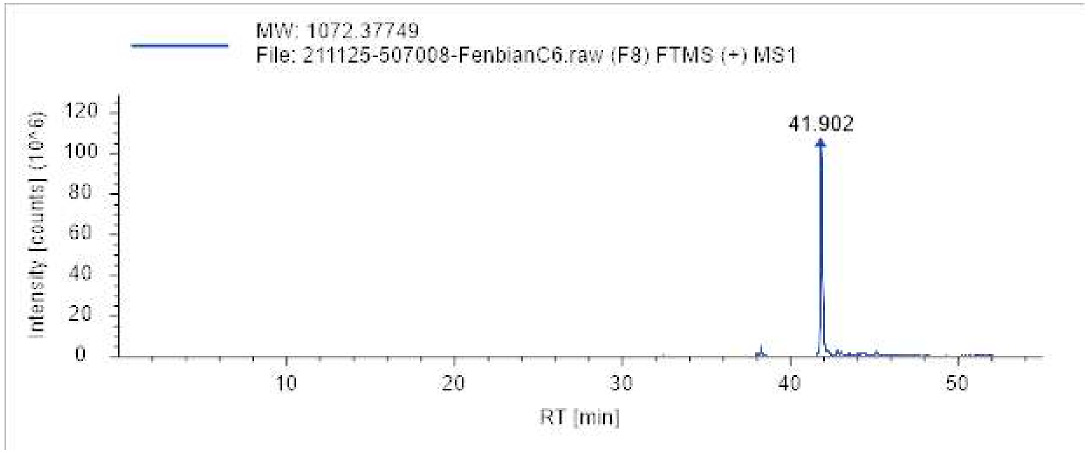

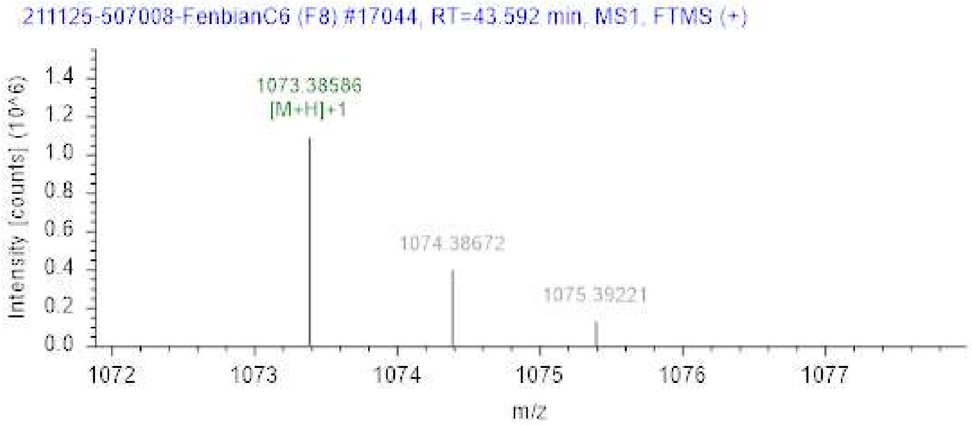

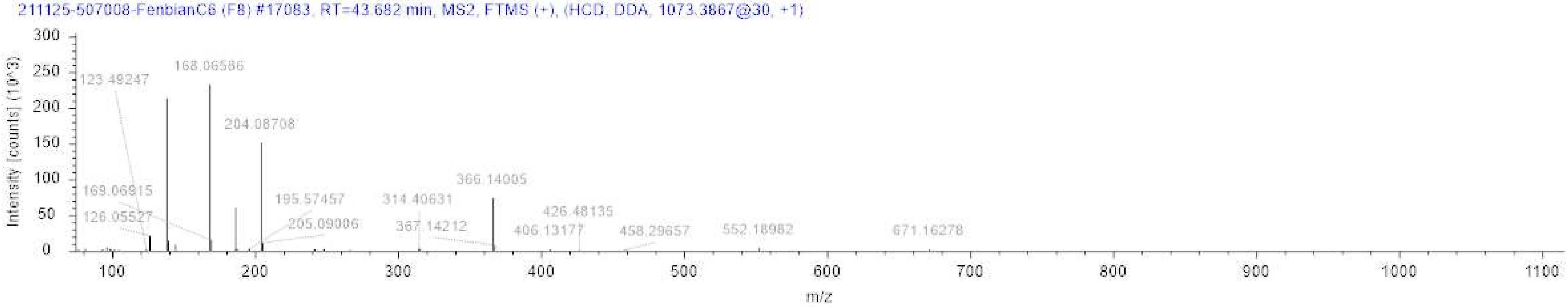

© Reported with Compound Discoverer 3.3

Structure	Name	RT [min]	Formula	Calc. MW	Areas
n/a		38.30	n/a	1072.37810	2.08e7 3.62e7

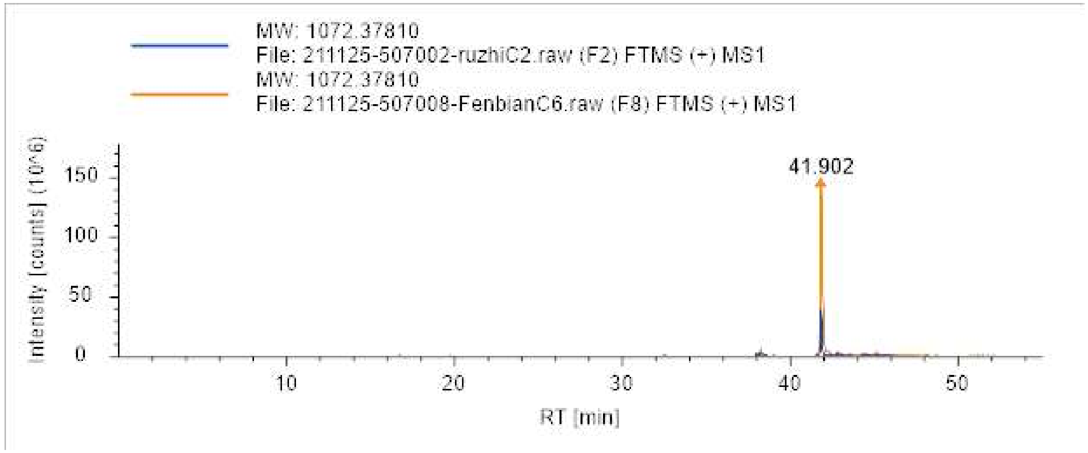

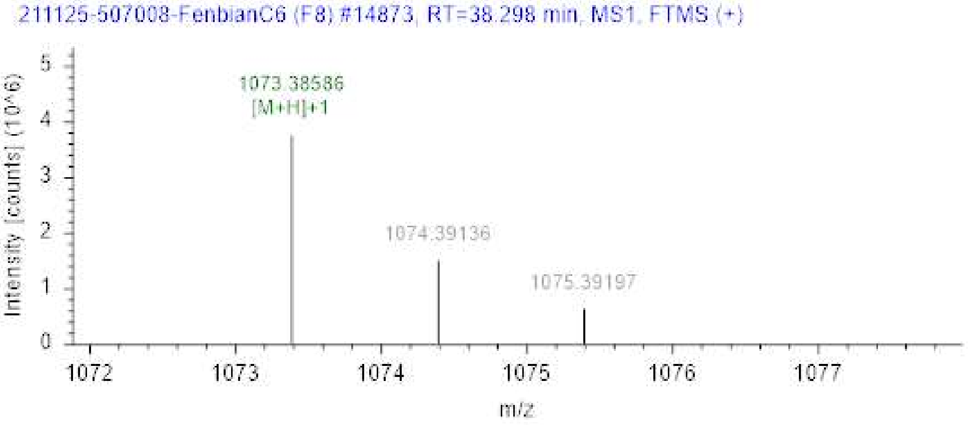

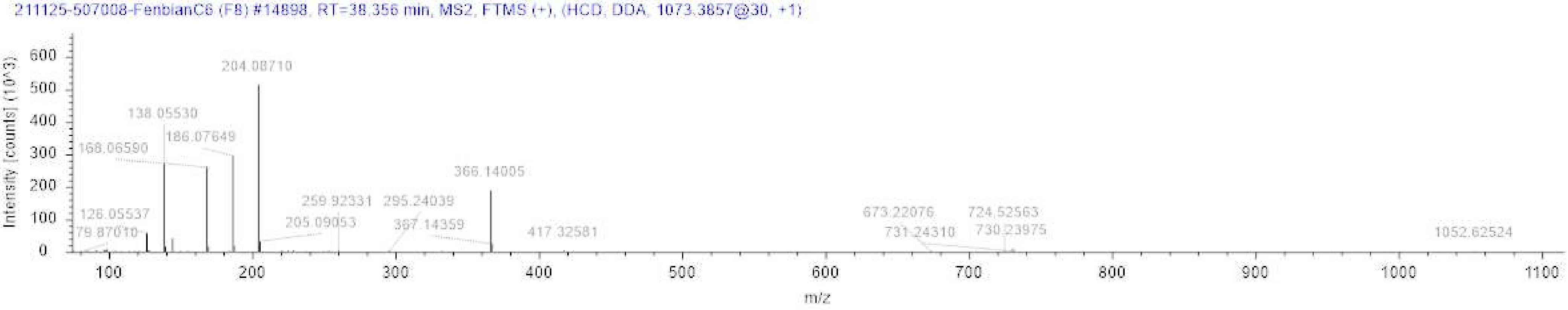

© Reported with Compound Discoverer 3.3

Compounds

Structure	Name	RT [min]	Formula	Calc. MW	Areas
n/a		45.19	n/a	1072.37894	3.14e7; 3.74e7

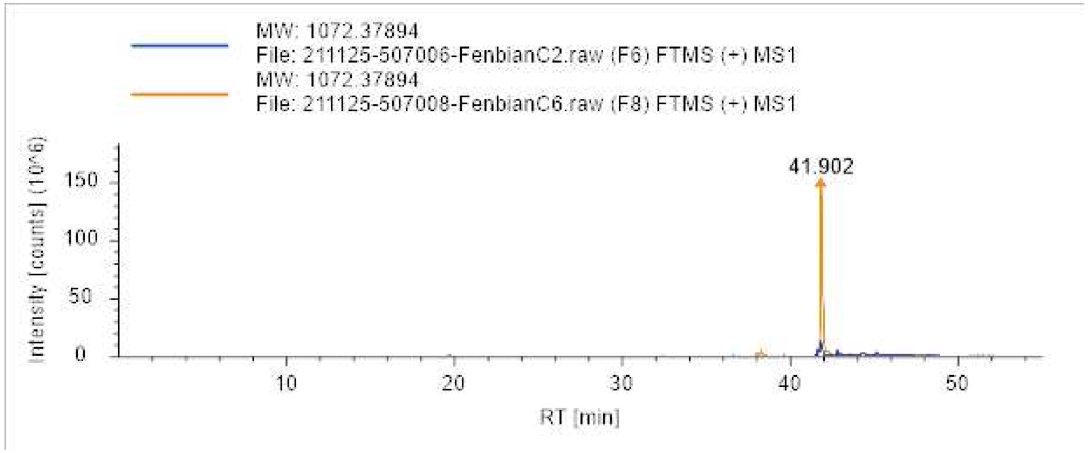

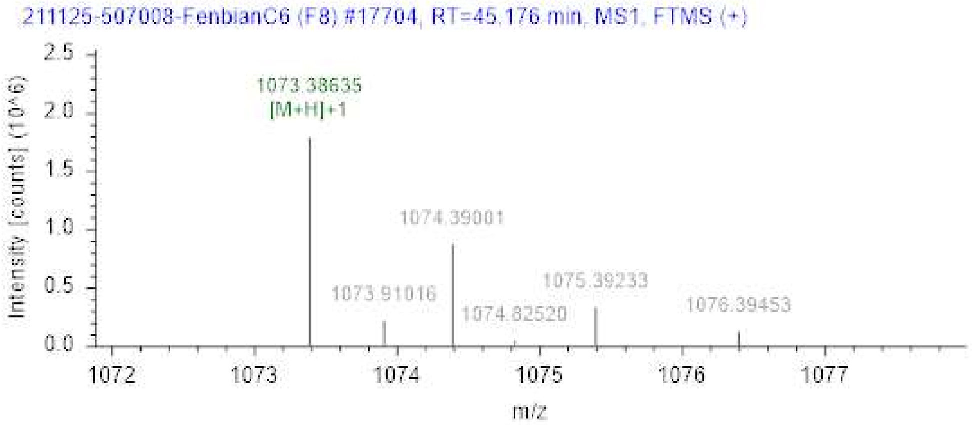

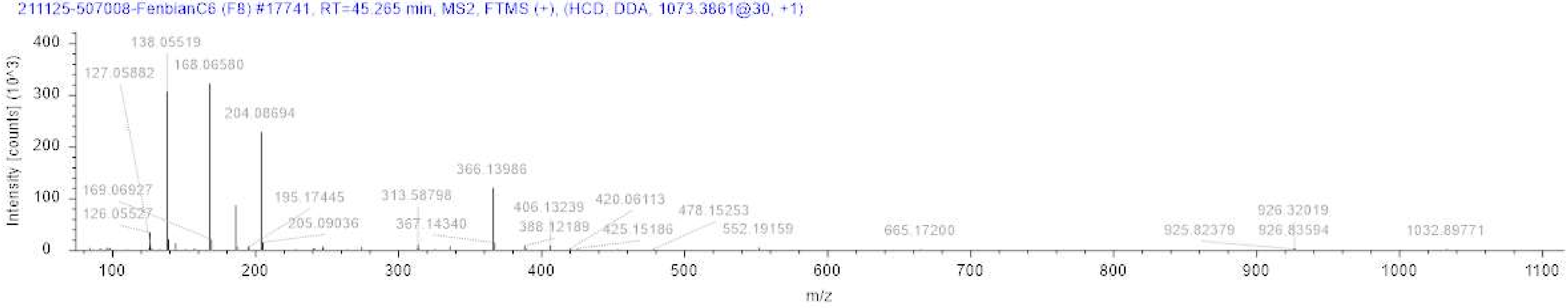

© Reported with Compound Discoverer 3.3

Structure	Name	RT [min]	Formula	Calc. MW	Areas
n/a		41.90	n/a	1072.37975	3.67e8 1.05e9 2.47e8 1.43e9 1.38e9 3.48e9

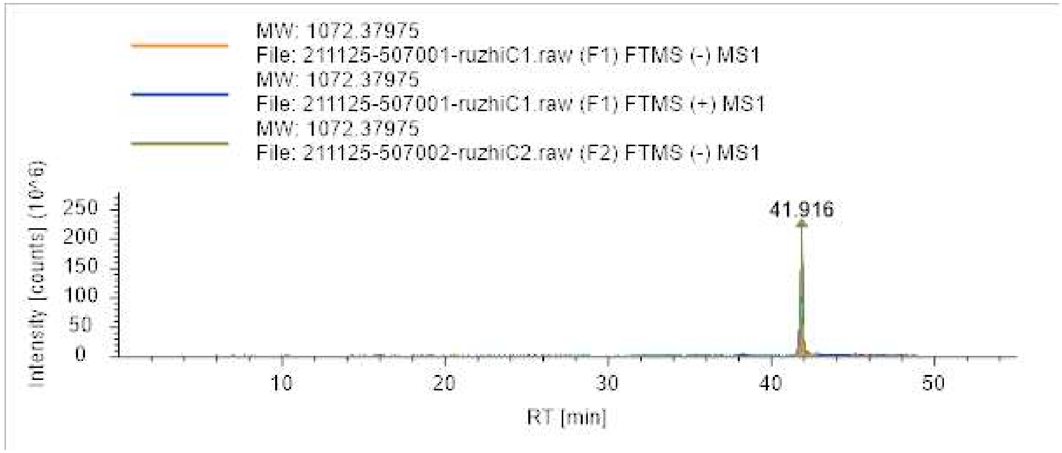

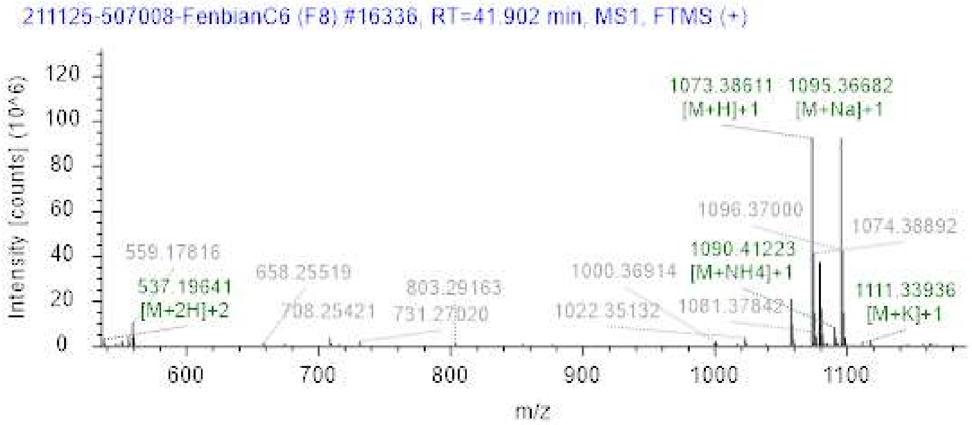

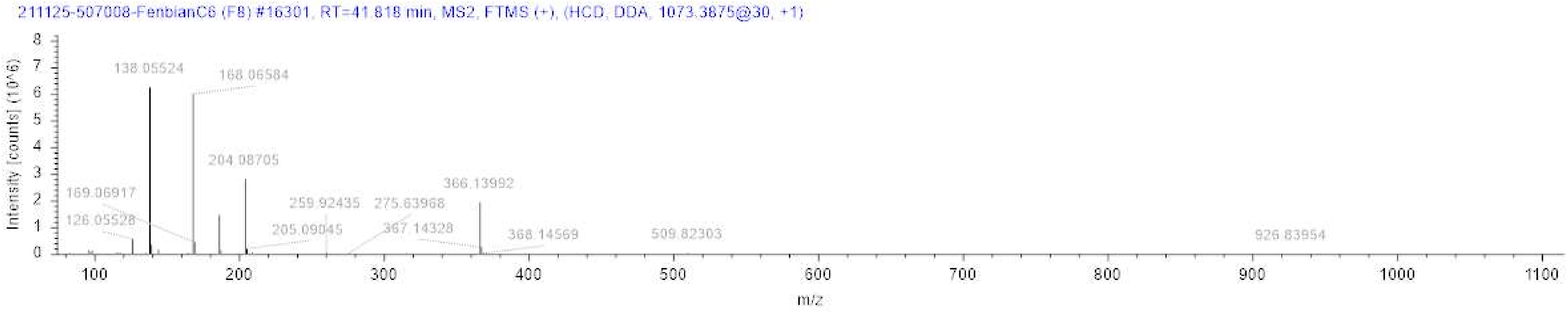

© Reported with Compound Discoverer 3.3

Structure	Name	RT [min]	Formula	Calc. MW	Areas
n/a		44.34	n/a	1073.38091	7.72e6

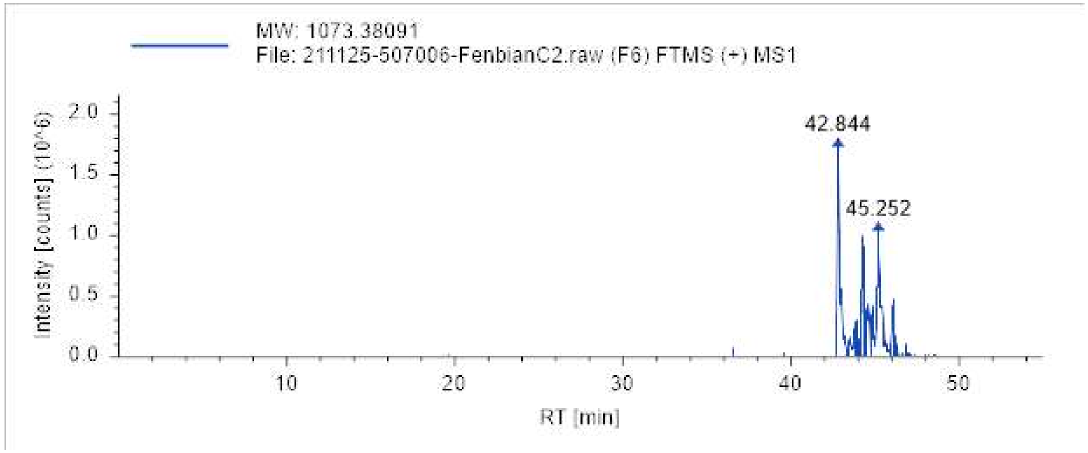

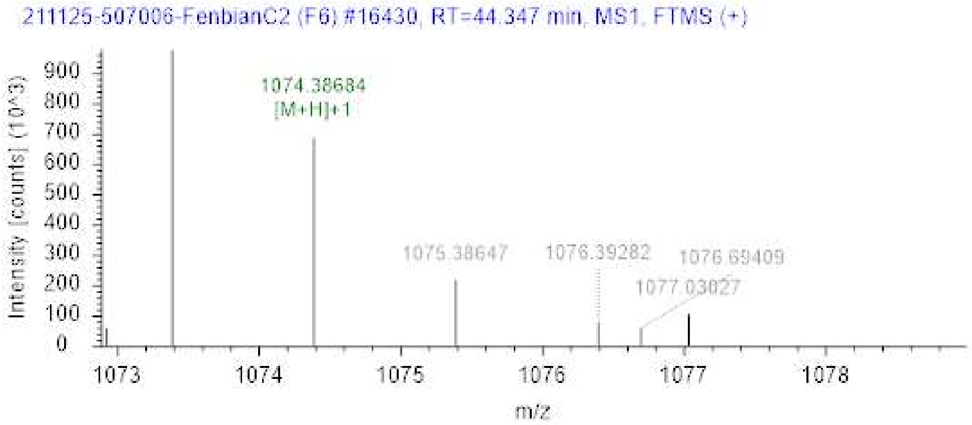

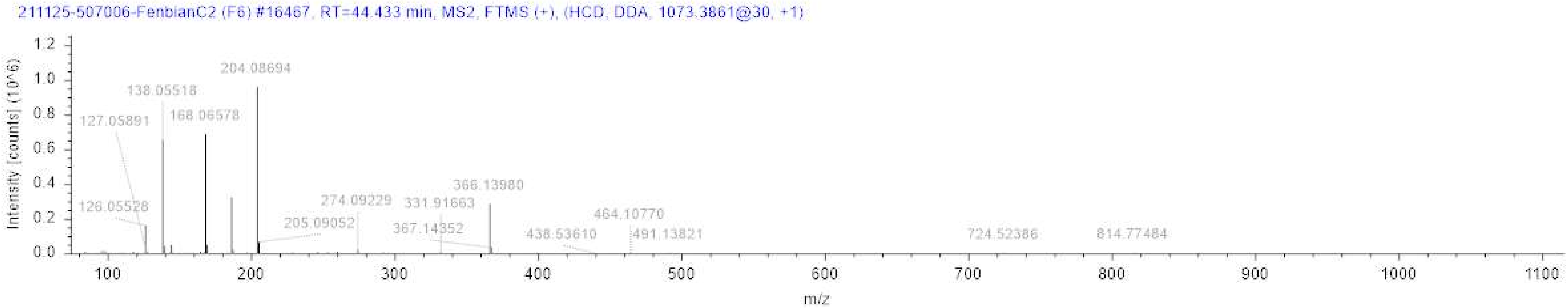

© Reported with Compound Discoverer 3.3

Structure	Name	RT [min]	Formula	Calc. MW	Areas
n/a		41.14	n/a	1097.40728	7.47e7

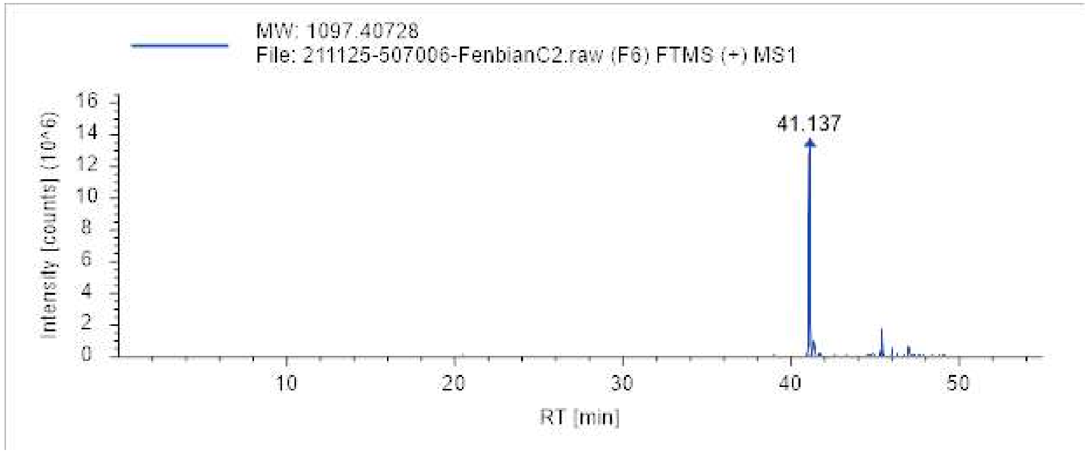

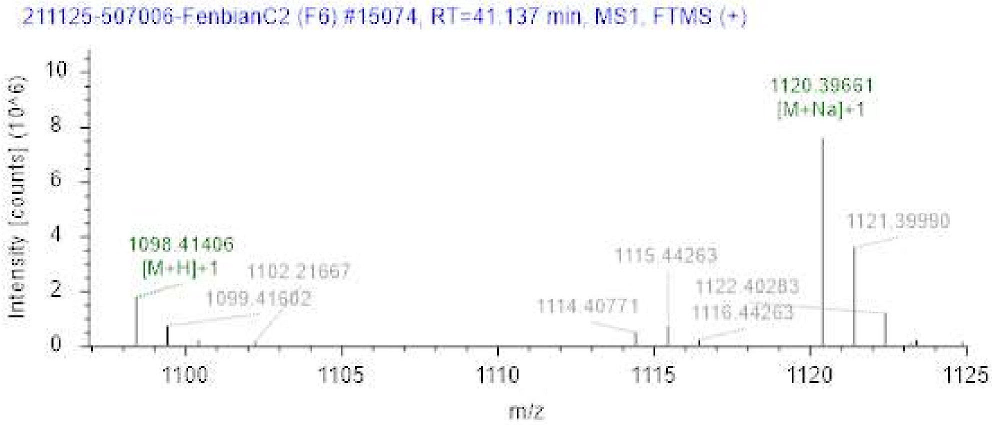

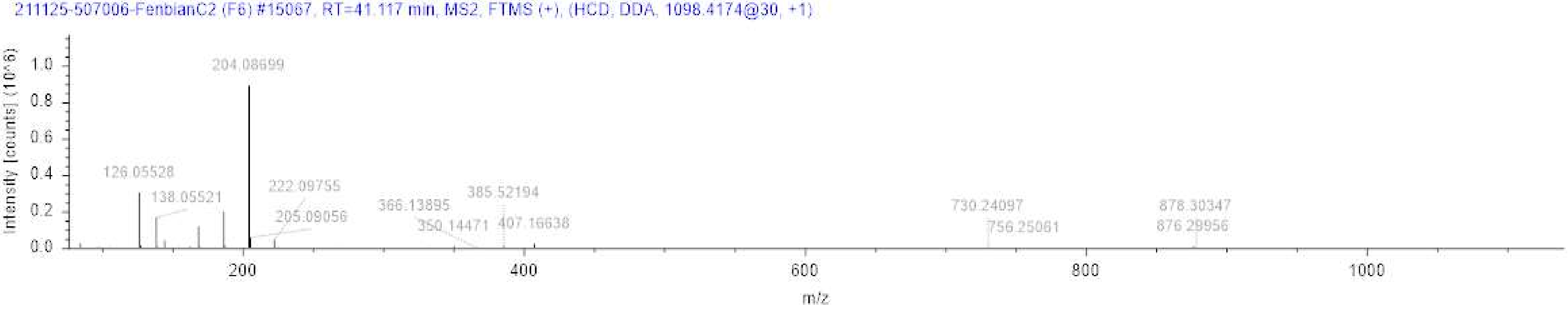

© Reported with Compound Discoverer 3.3

Structure	Name	RT [min]	Formula	Calc. MW	Areas
n/a		41.32	n/a	1113.40572	4.18e7 2.53e8

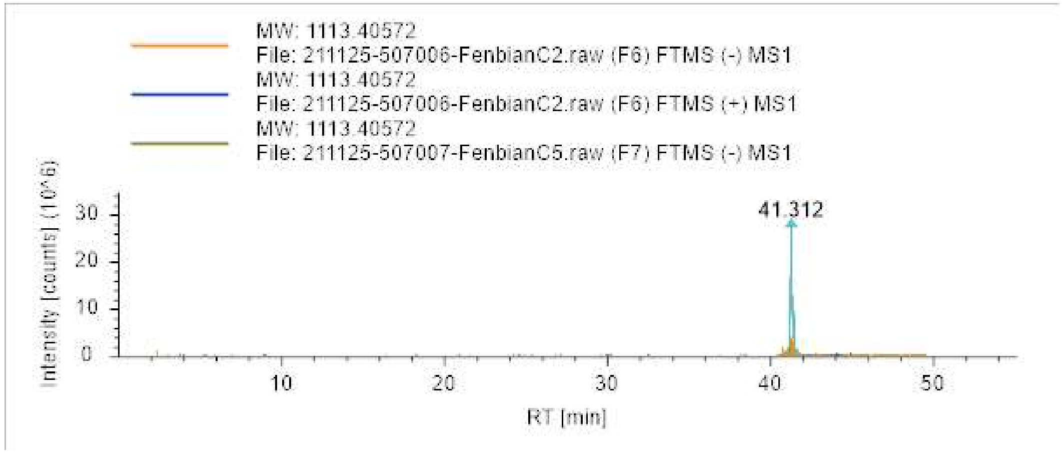

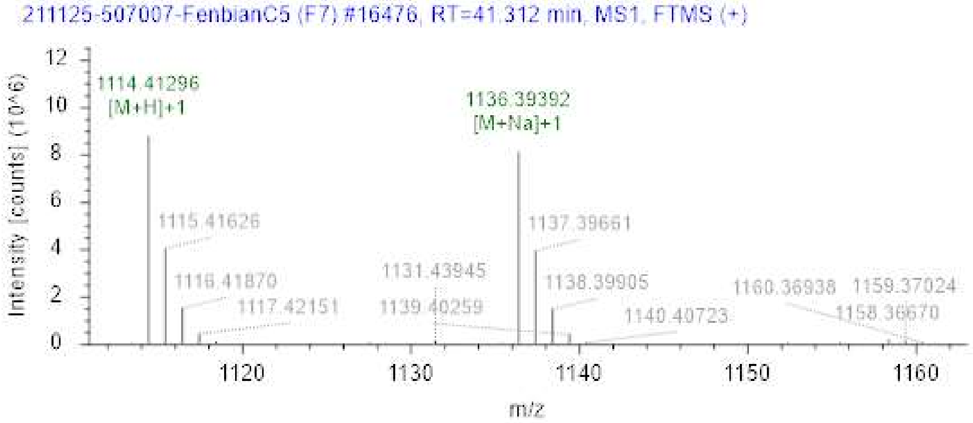

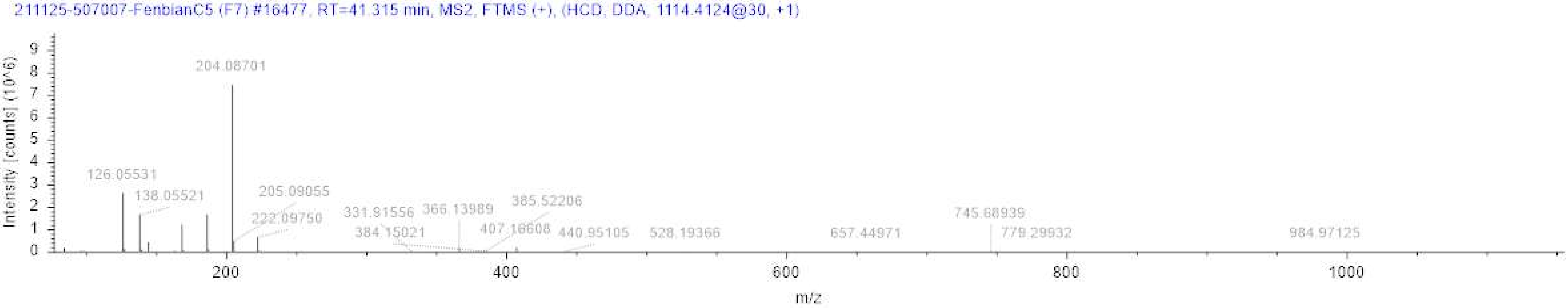

© Reported with Compound Discoverer 3.3

Compounds

Structure	Name	RT [min]	Formula	Calc. MW	Areas
n/a		30.70	n/a	1138.43205	8.69e7

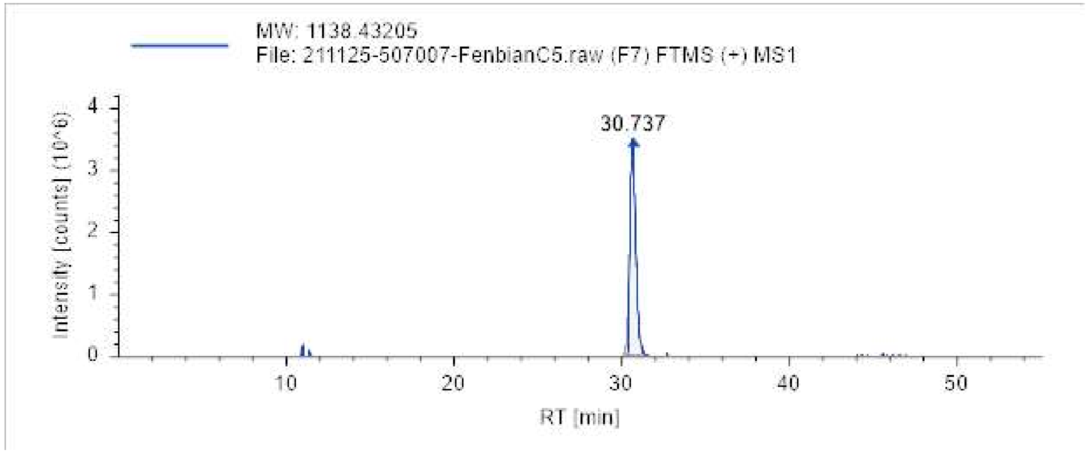

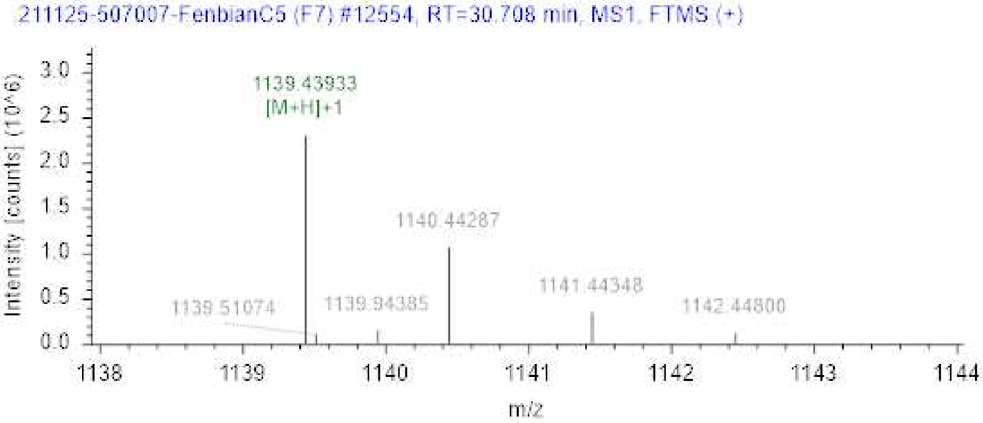

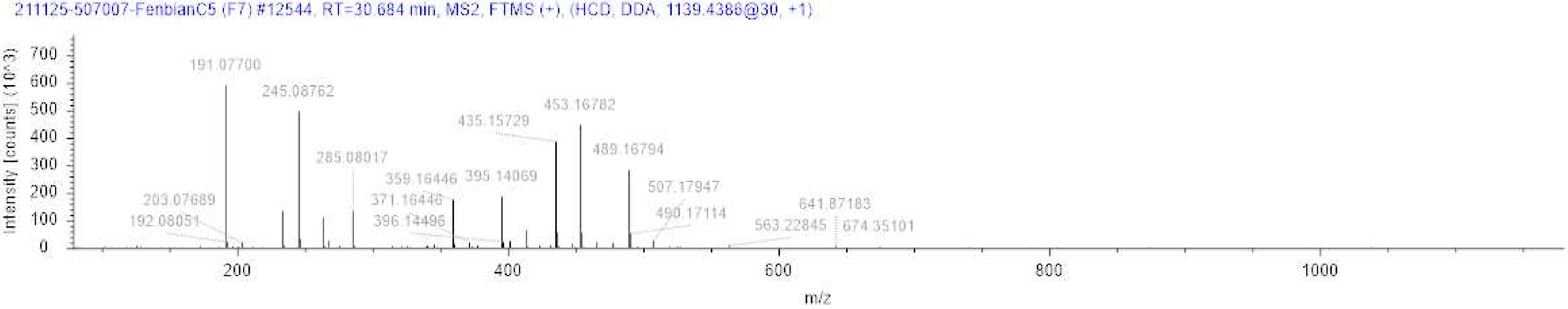

© Reported with Compound Discoverer 3.3

Structure	Name	RT [min]	Formula	Calc. MW	Areas
n/a		43.83	n/a	1144.39739	6.96e6

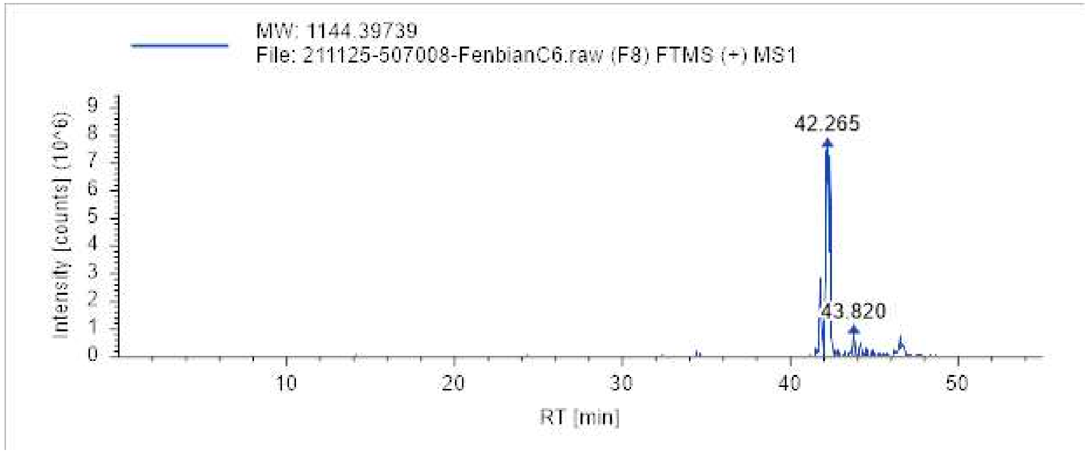

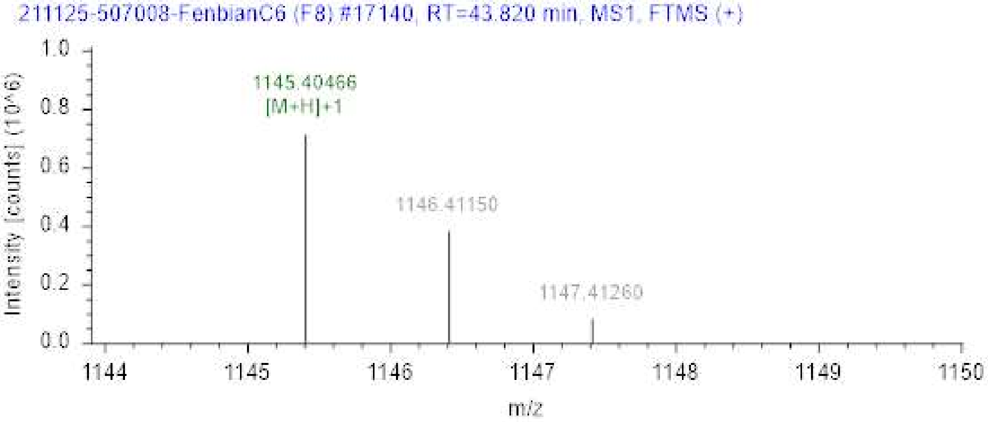

© Reported with Compound Discoverer 3.3

Structure	Name	RT [min]	Formula	Calc. MW	Areas
n/a		44.92	n/a	1144.39958	6.25e6

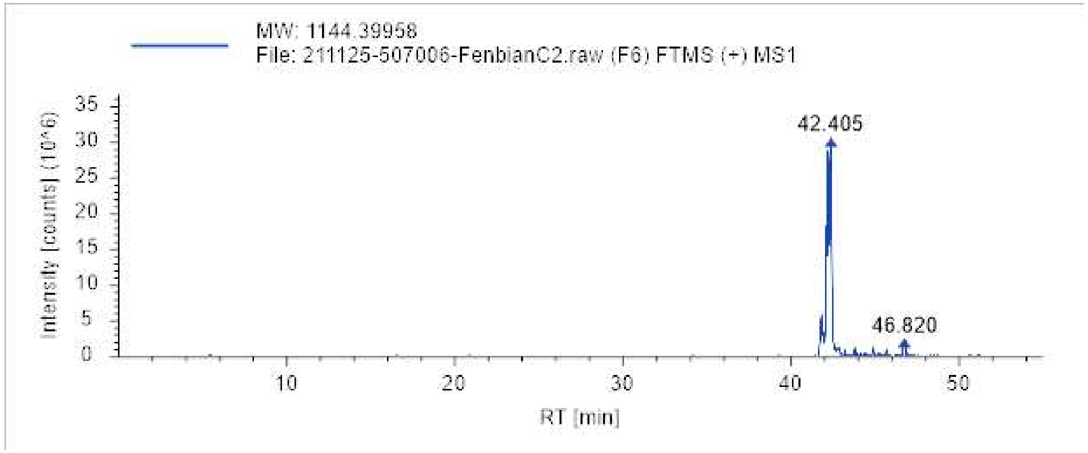

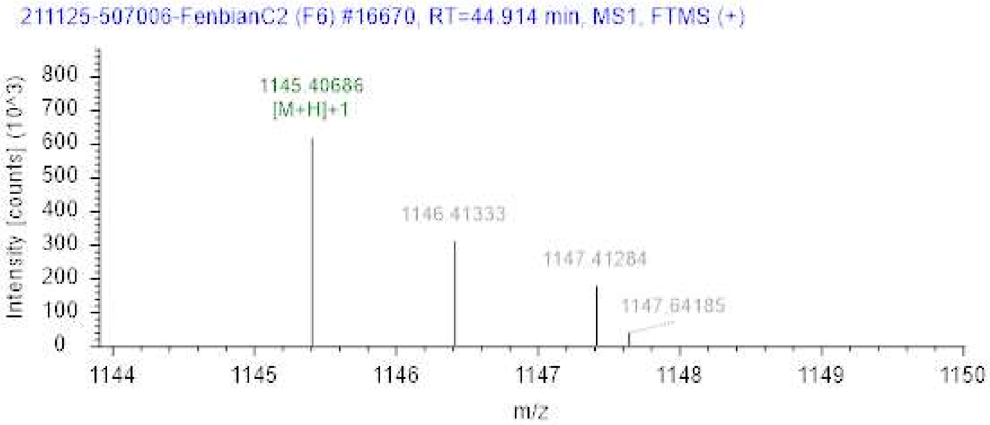

© Reported with Compound Discoverer 3.3

Compounds

Structure	Name	RT [min]	Formula	Calc. MW	Areas
n/a		42.35	n/a	1144.40018	2.94e7 2.02e7 5.97e8 4.36e7

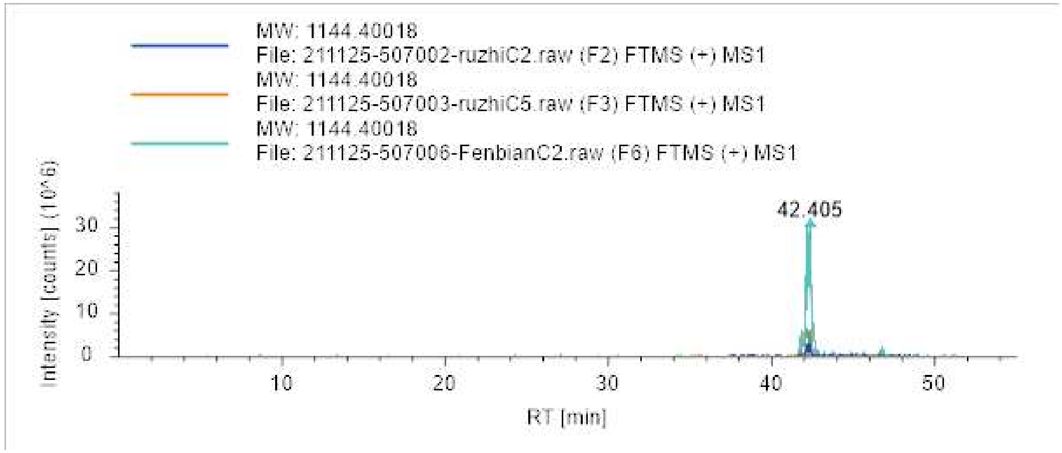

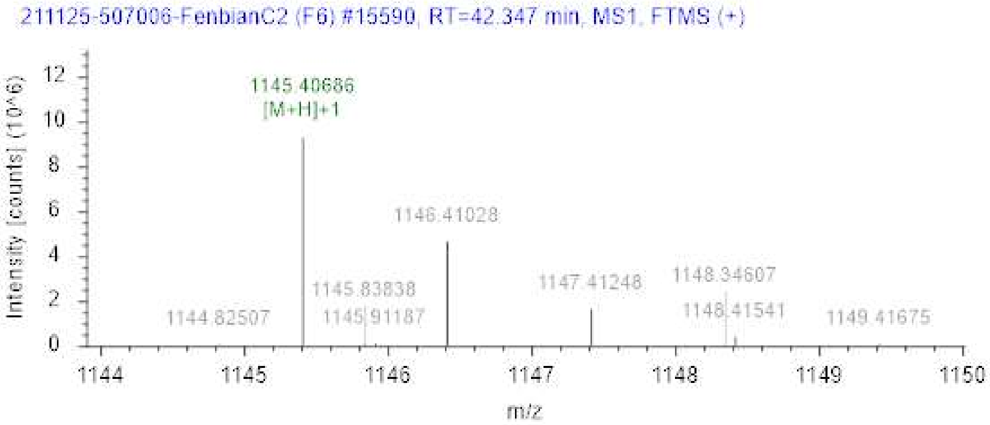

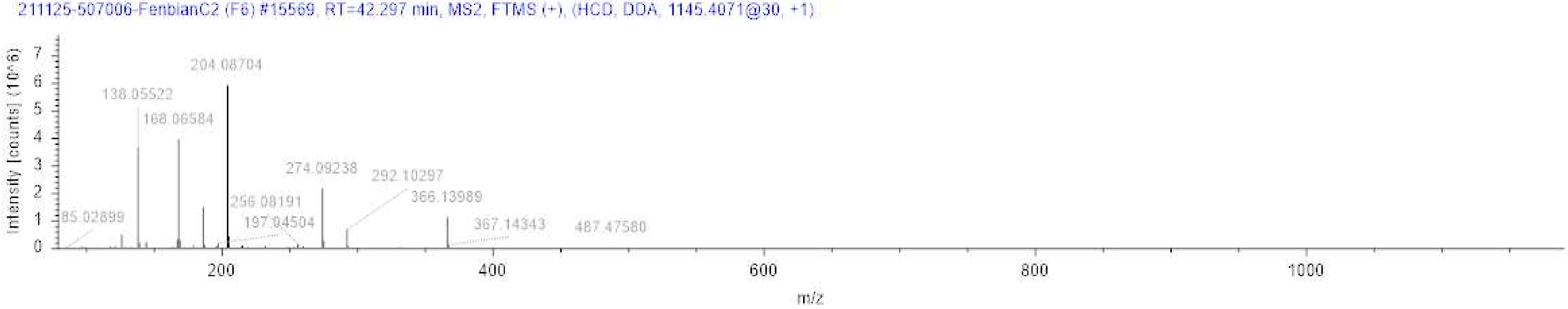

© Reported with Compound Discoverer 3.3

Compounds

Structure	Name	RT [min]	Formula	Calc. MW	Areas
n/a		41.84	n/a	1144.40098	1.22e7 2.17e7 2.03e7 2.63e8

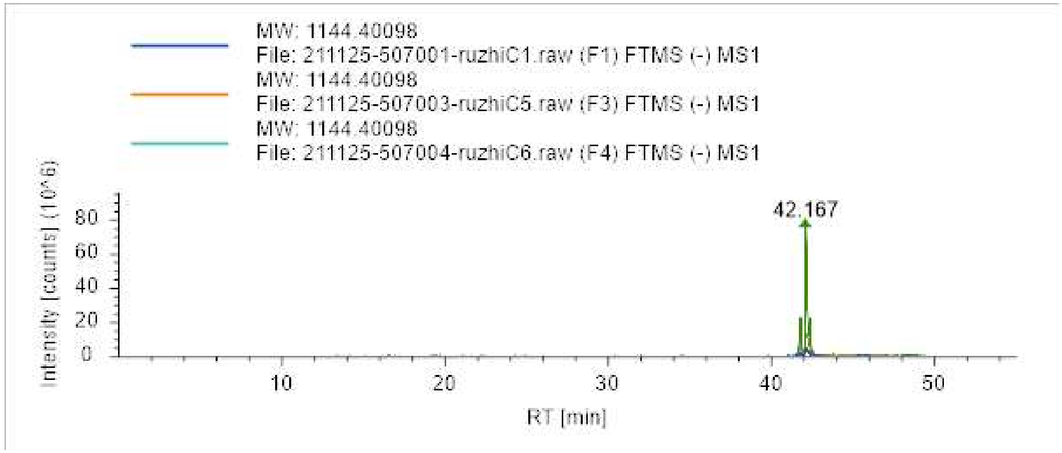

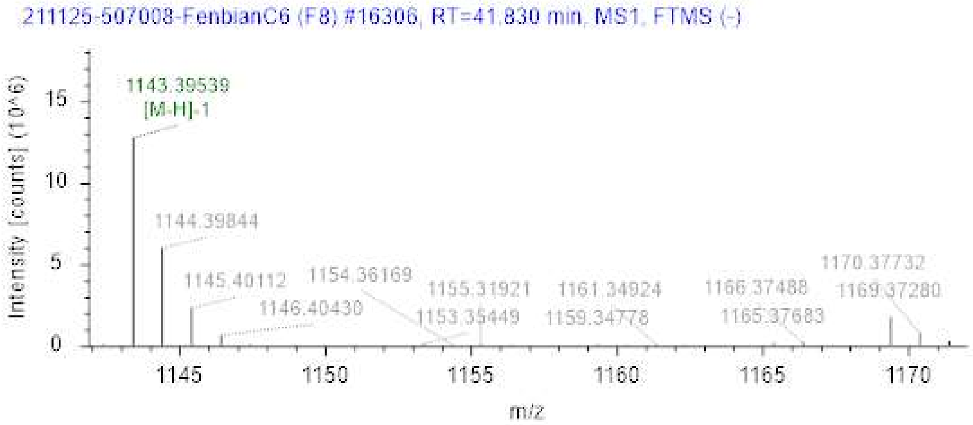

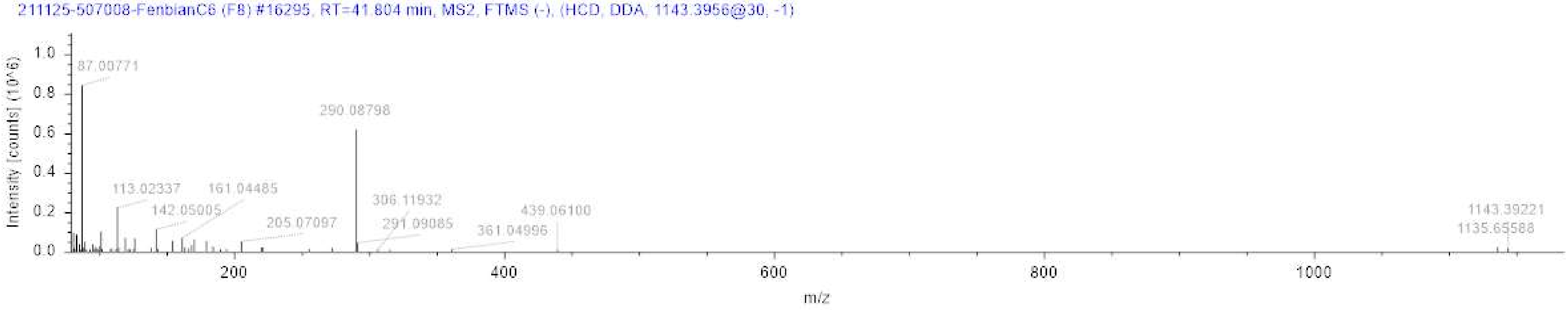

© Reported with Compound Discoverer 3.3

Compounds

Structure	Name	RT [min]	Formula	Calc. MW	Areas
n/a		42.36	n/a	1160.39656	1.11e8; 3.87e7

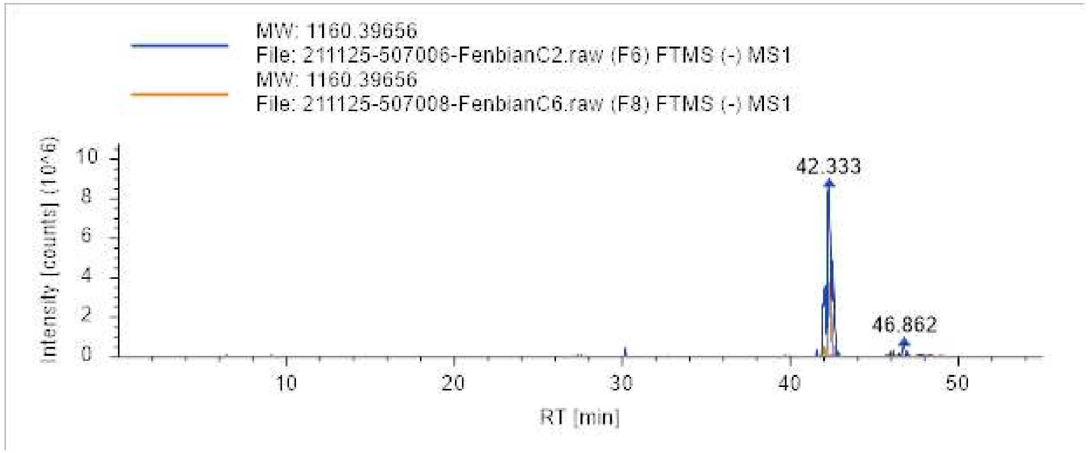

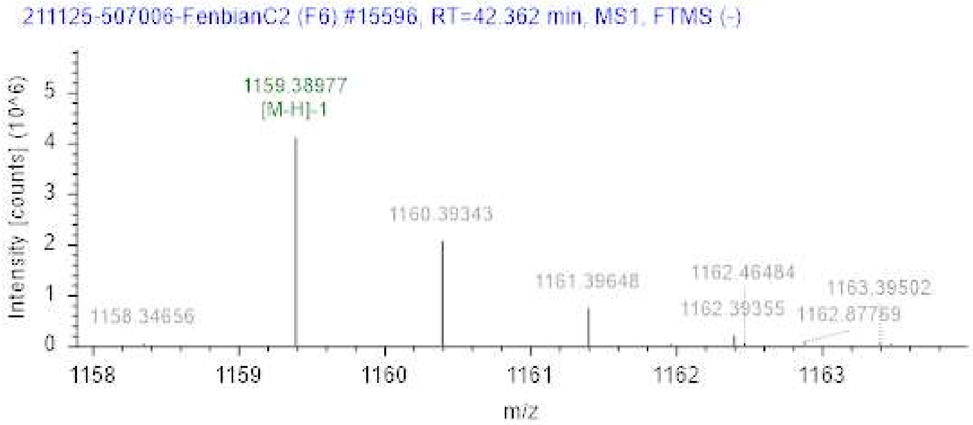

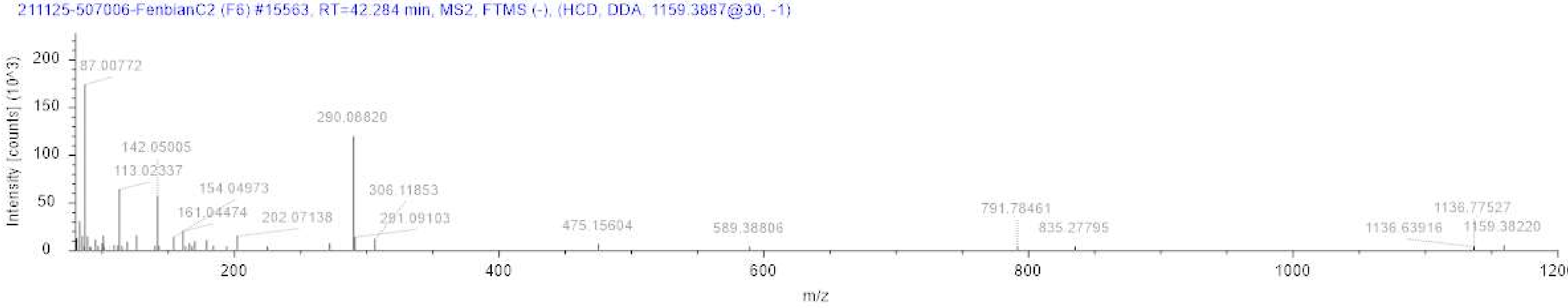

© Reported with Compound Discoverer 3.3

Compounds

Structure	Name	RT [min]	Formula	Calc. MW	Areas
n/a		42.59	n/a	1161.41713	1.53e7 | 9.61e6 | 1.37e7 | 3.42e8

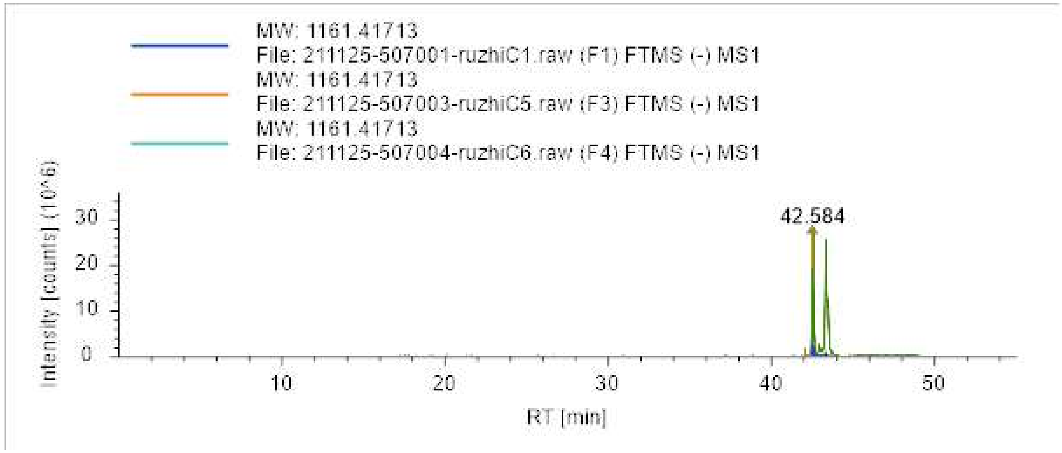

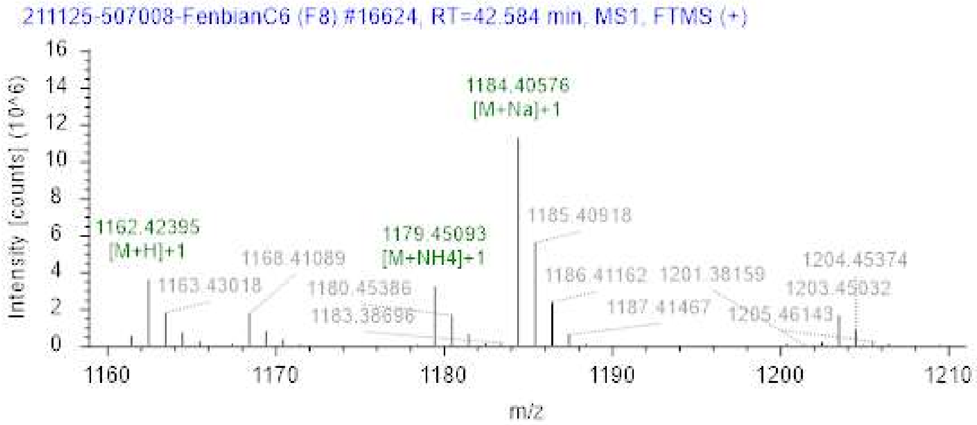

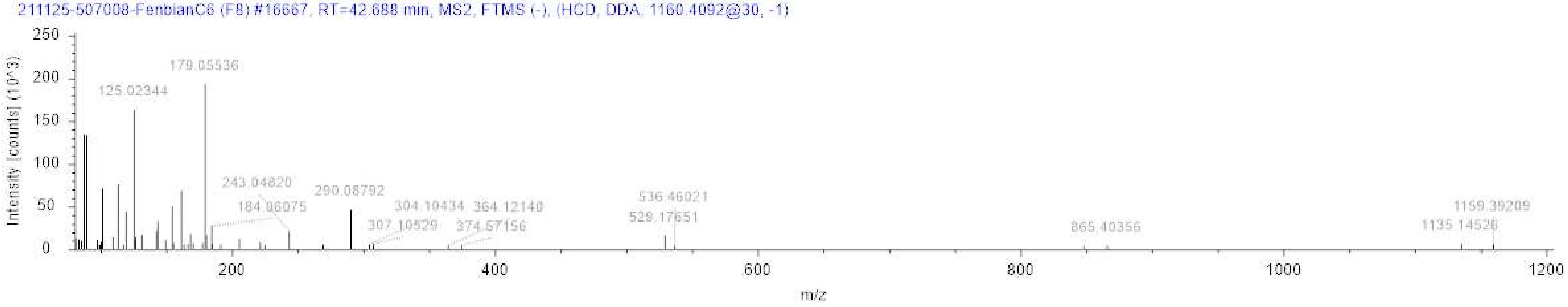

© Reported with Compound Discoverer 3.3

Compounds

Structure	Name	RT [min]	Formula	Calc. MW	Areas
n/a		43.41	n/a	1161.41806	7.14e6 1.82e7 2.67e8

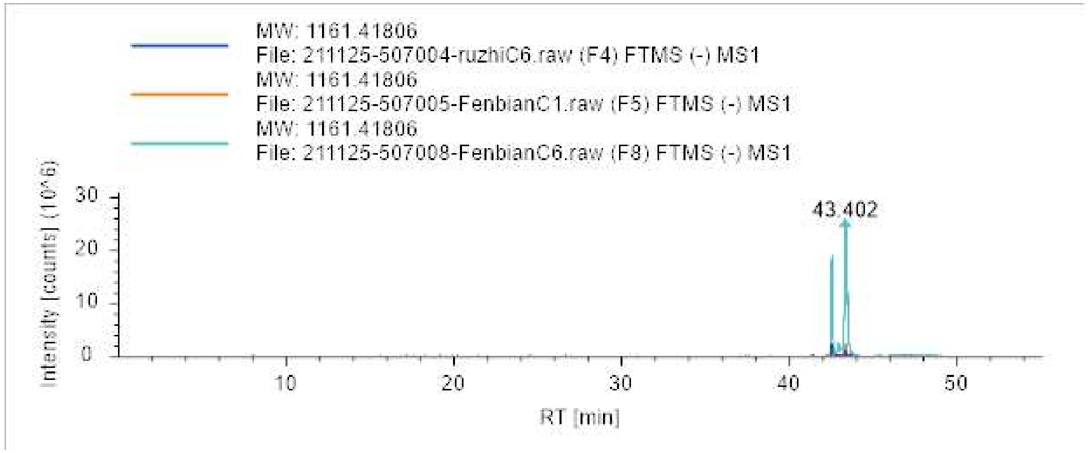

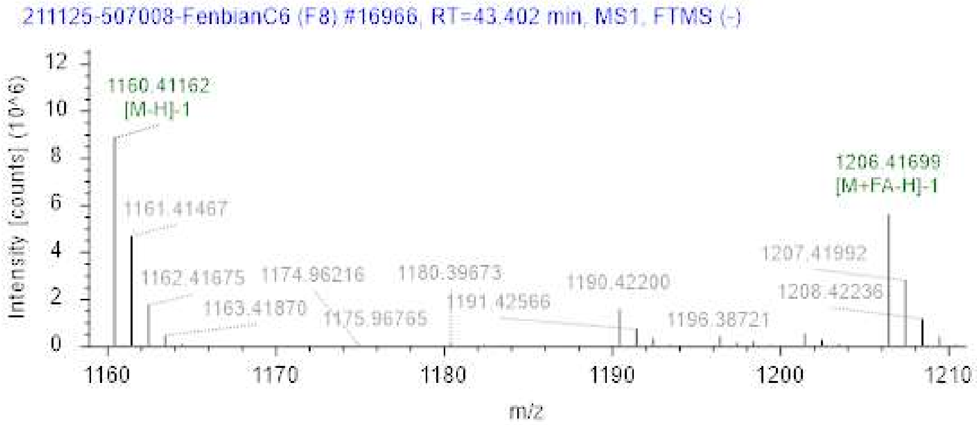

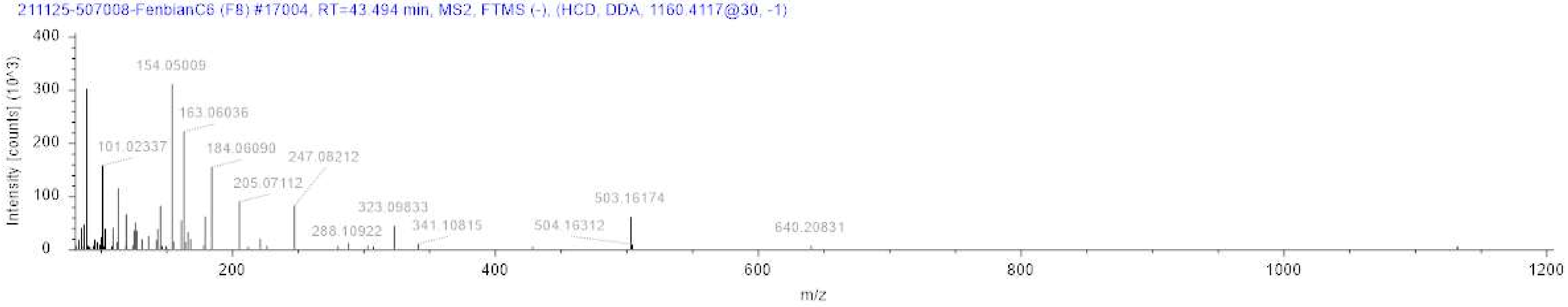

© Reported with Compound Discoverer 3.3

Compounds

Structure	Name	RT [min]	Formula	Calc. MW	Areas
n/a		46.34	n/a	1169.43547	4.77e6

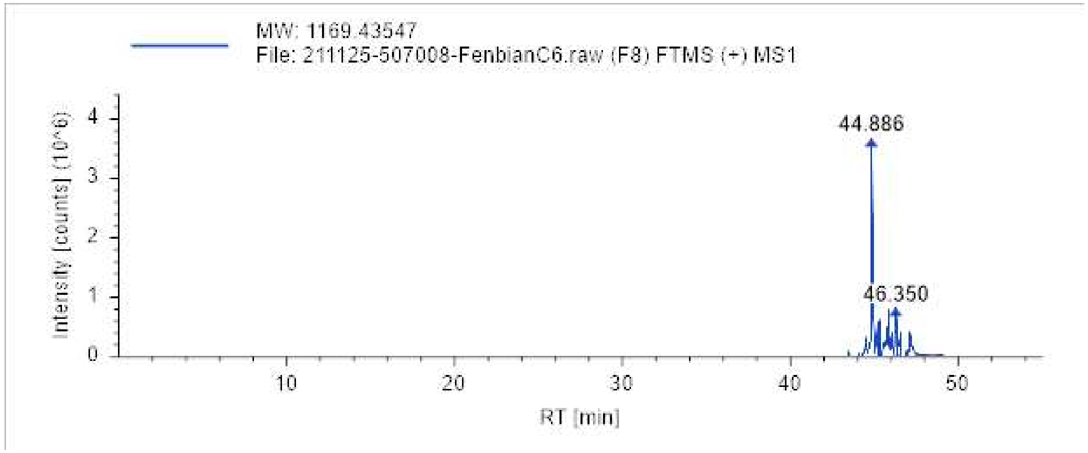

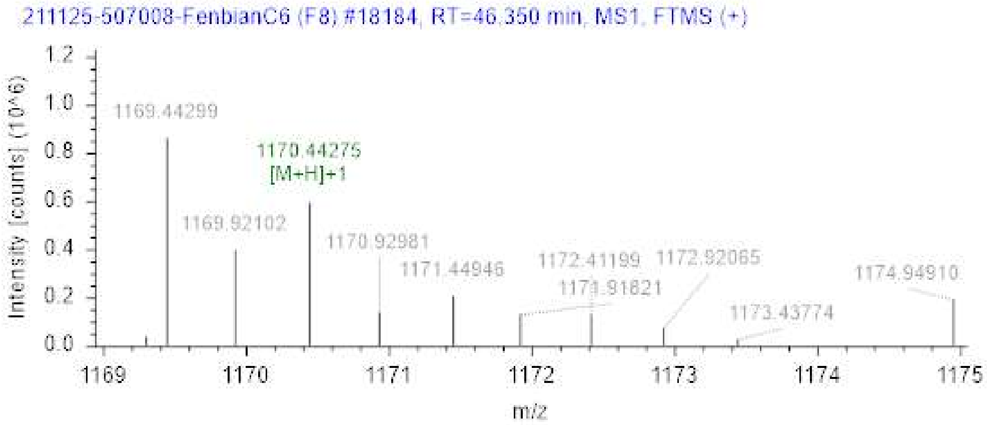

© Reported with Compound Discoverer 3.3

Structure	Name	RT [min]	Formula	Calc. MW	Areas
n/a		42.44	n/a	1177.38706	8.72e6

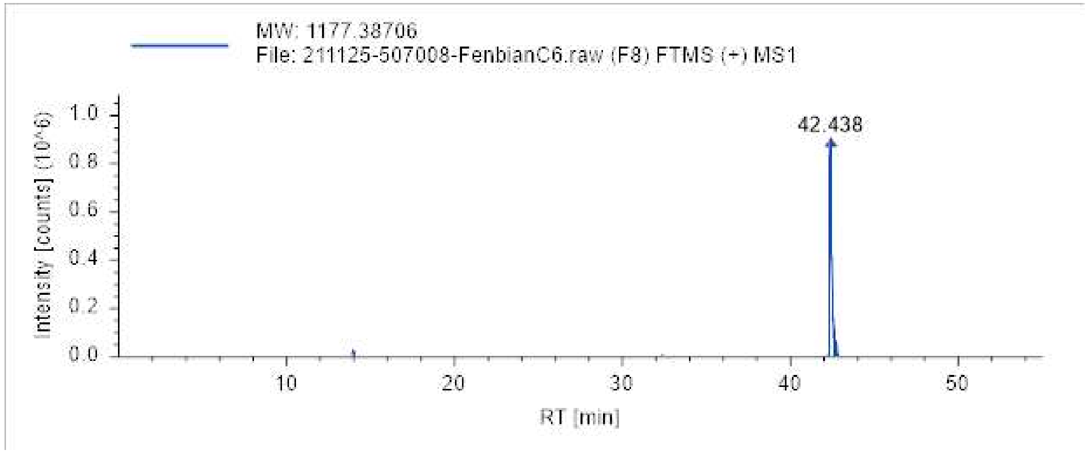

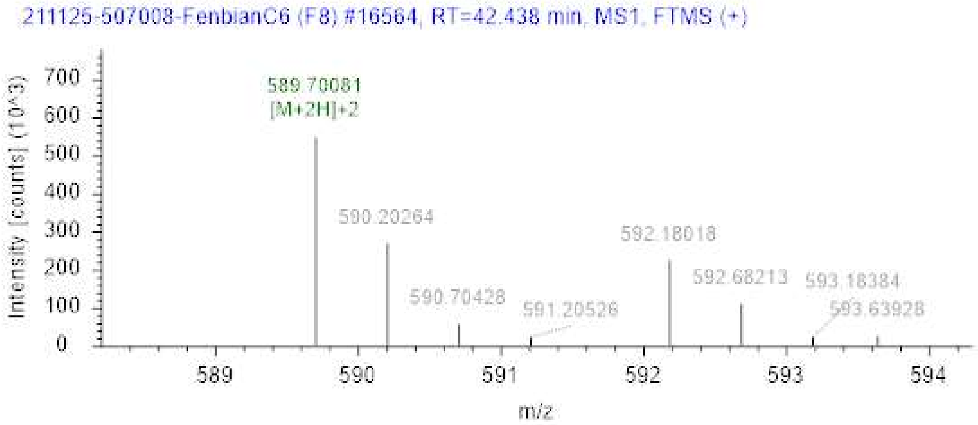

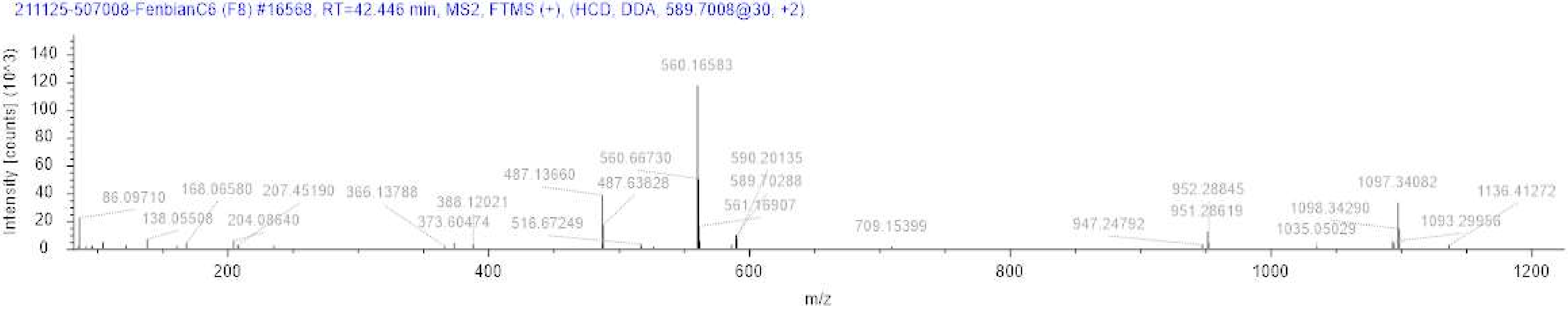

© Reported with Compound Discoverer 3.3

Structure	Name	RT [min]	Formula	Calc. MW	Areas
n/a		42.77	n/a	1177.41340	1.30e7

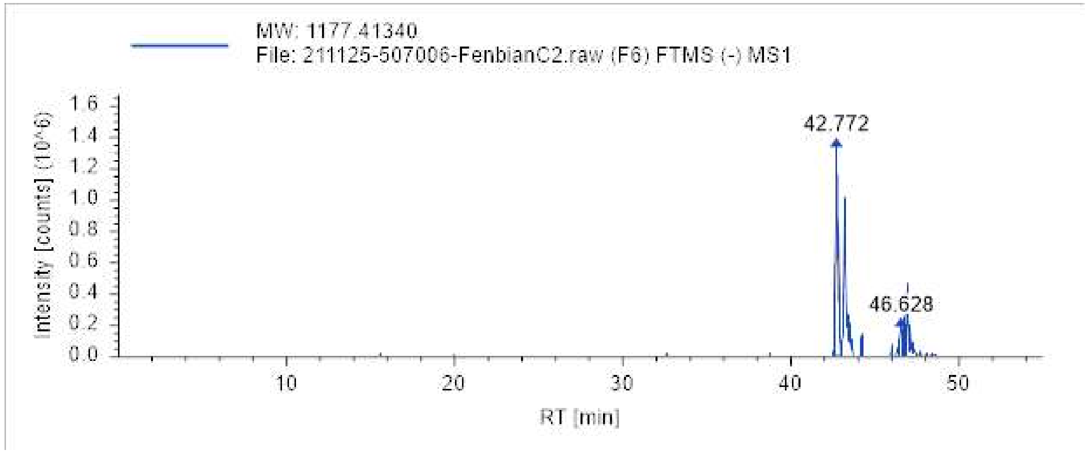

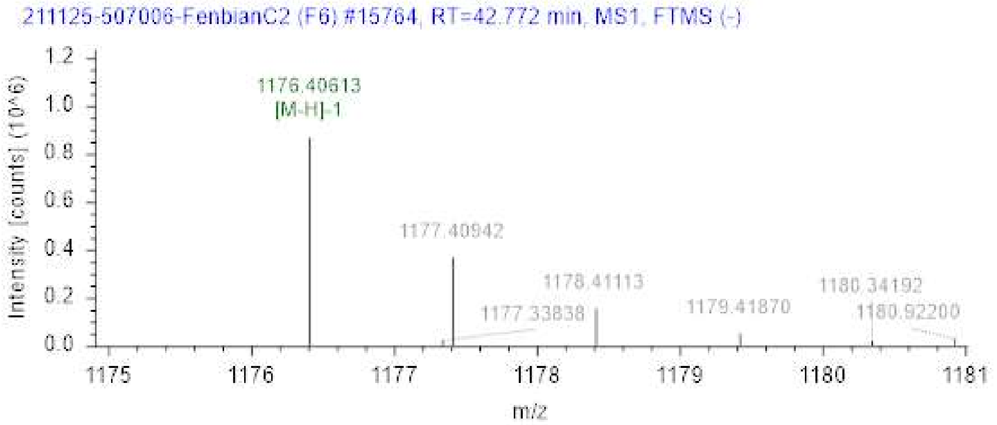

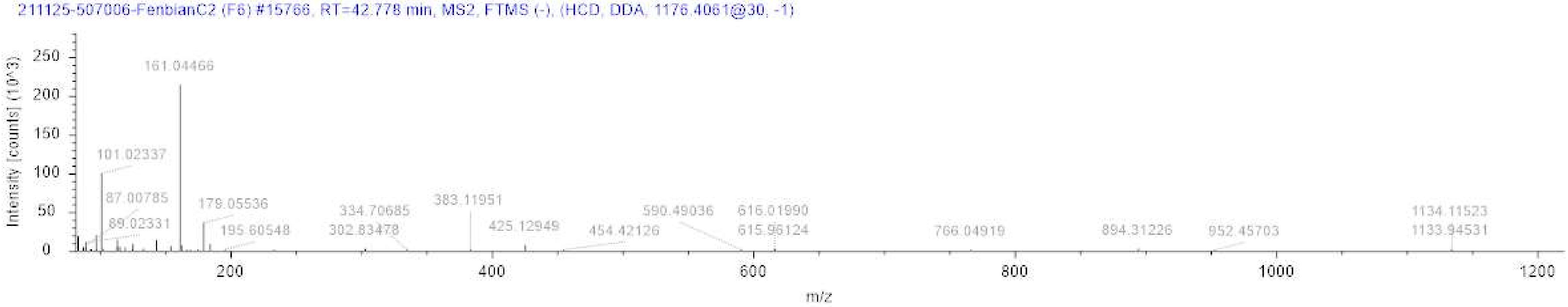

© Reported with Compound Discoverer 3.3

Compounds

Structure	Name	RT [min]	Formula	Calc. MW	Areas
n/a		43.93	n/a	1201.42026	9.96e6

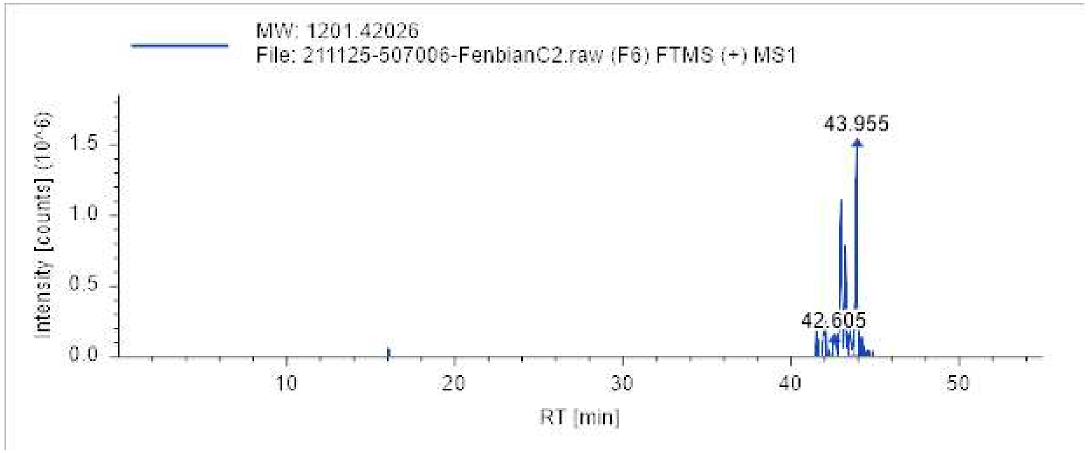

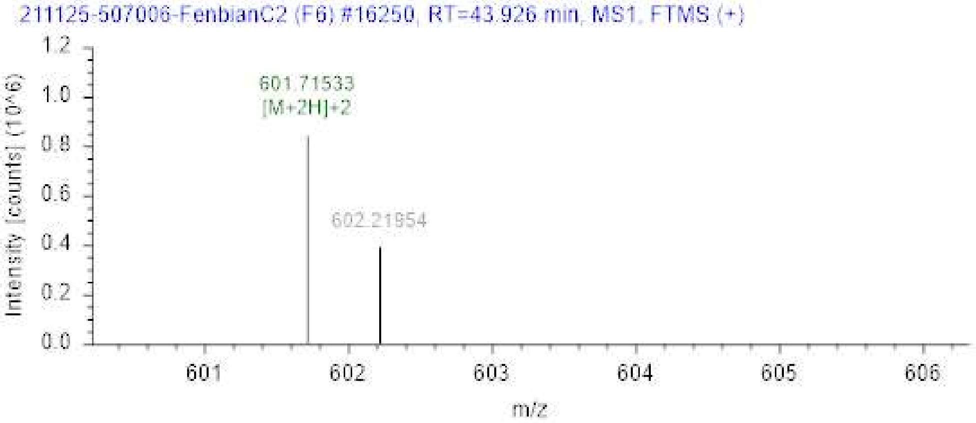

© Reported with Compound Discoverer 3.3

Structure	Name	RT [min]	Formula	Calc. MW	Areas
n/a		42.04	n/a	1201.42261	6.15e8 4.16e8

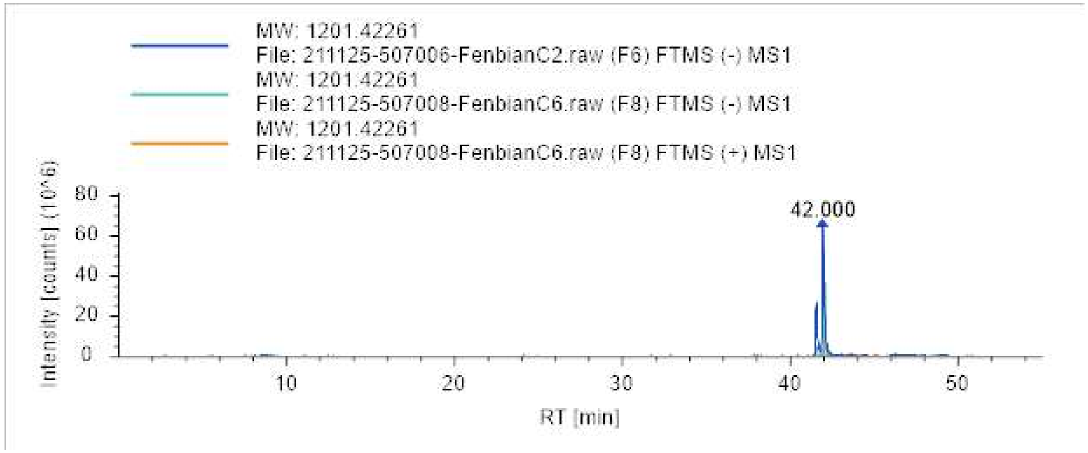

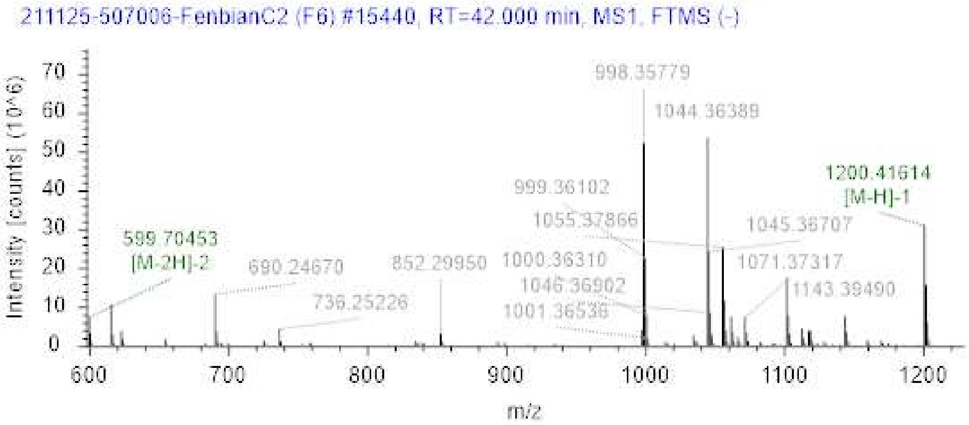

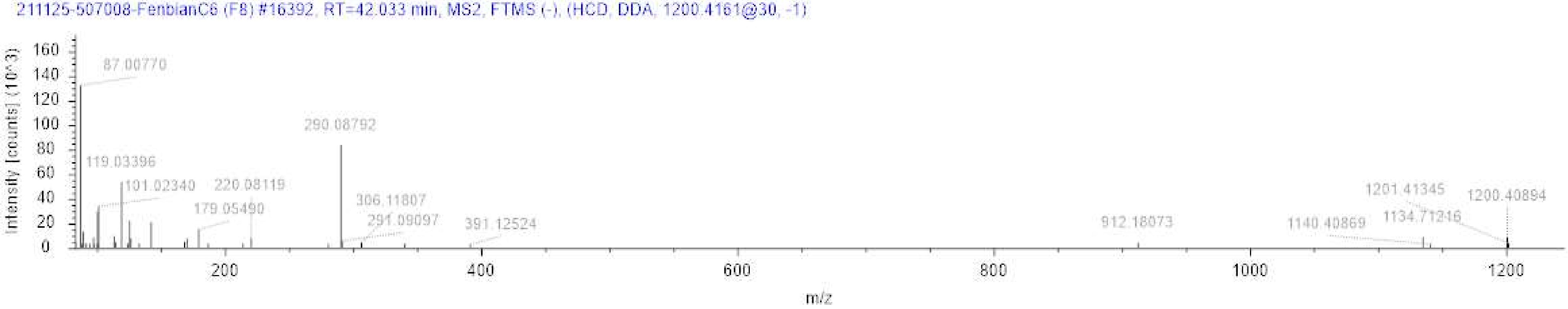

© Reported with Compound Discoverer 3.3

Structure	Name	RT [min]	Formula	Calc. MW	Areas
n/a		43.97	n/a	1202.44054	6.65e6 3.69e7 6.17e6

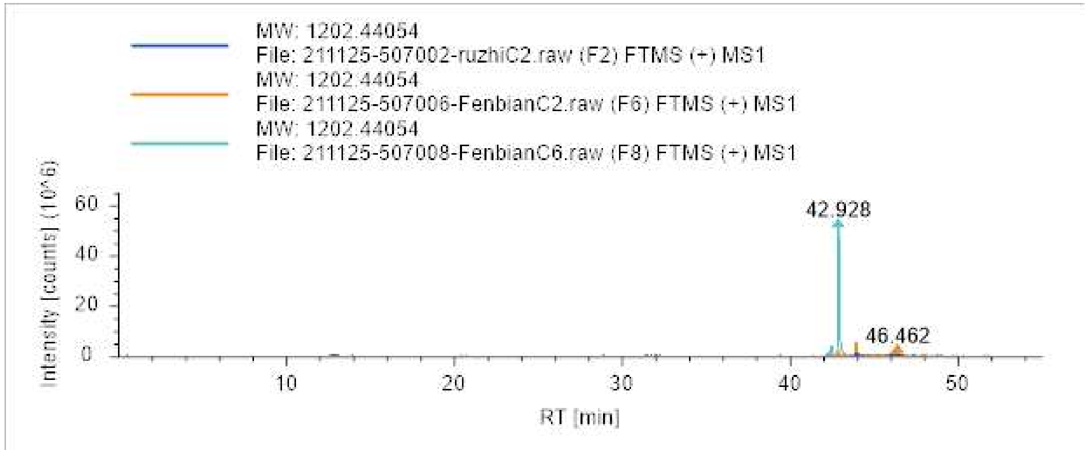

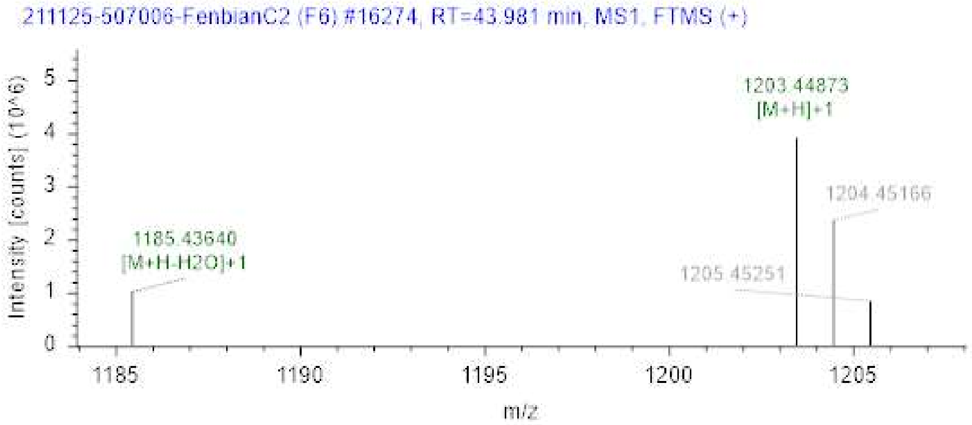

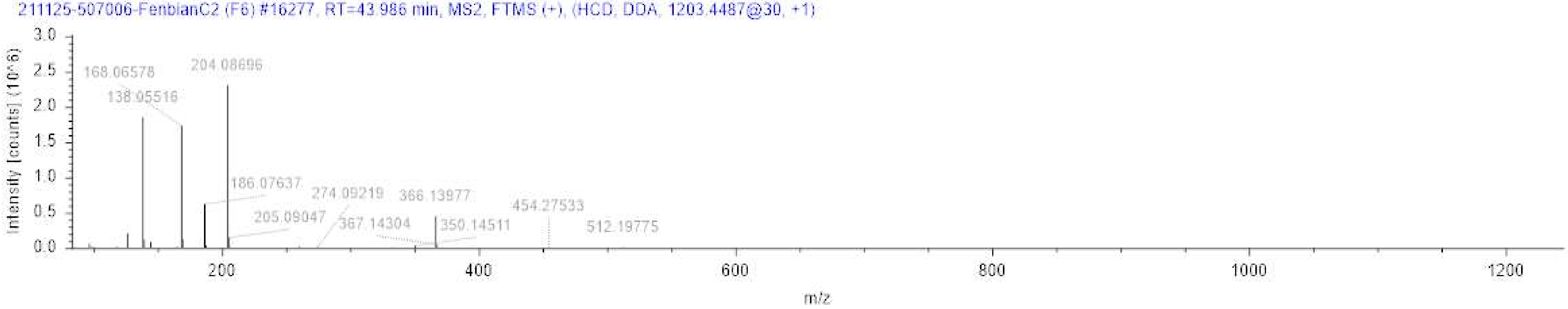

© Reported with Compound Discoverer 3.3

Structure	Name	RT [min]	Formula	Calc. MW	Areas
n/a		44.88	n/a	1202.44281	3.72e7 1.11e7

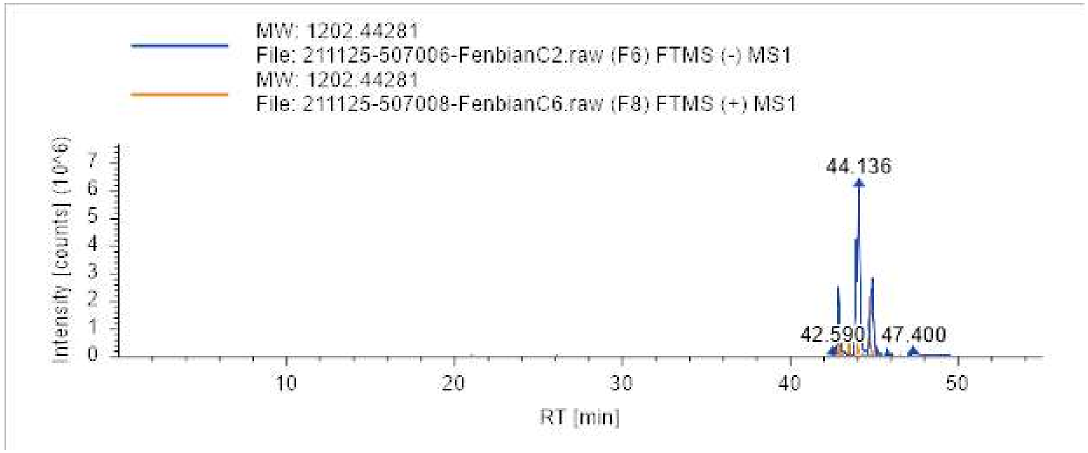

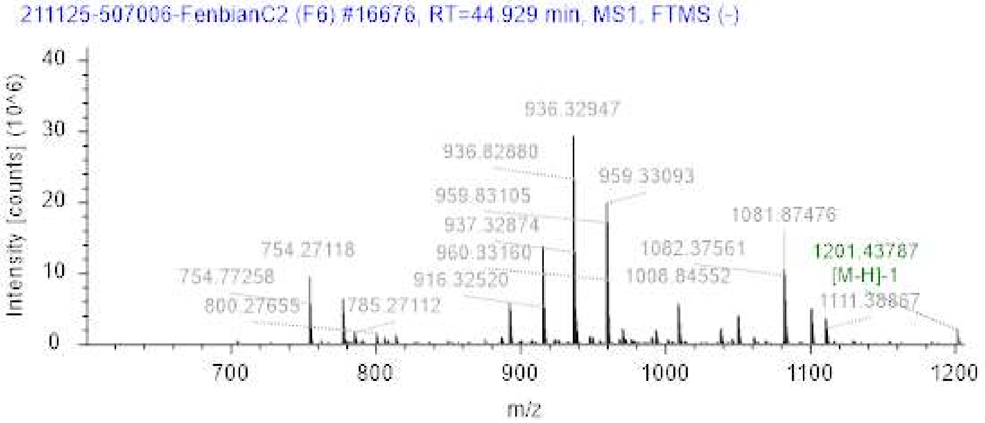

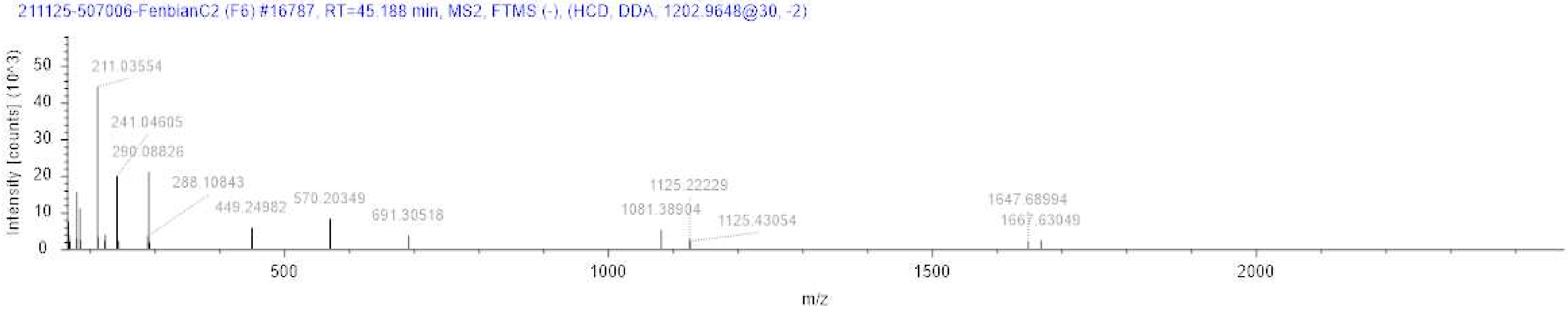

© Reported with Compound Discoverer 3.3

Compounds

Structure	Name	RT [min]	Formula	Calc. MW	Areas
n/a		42.94	n/a	1202.44326	4.99e7 1.04e8 1.64e9

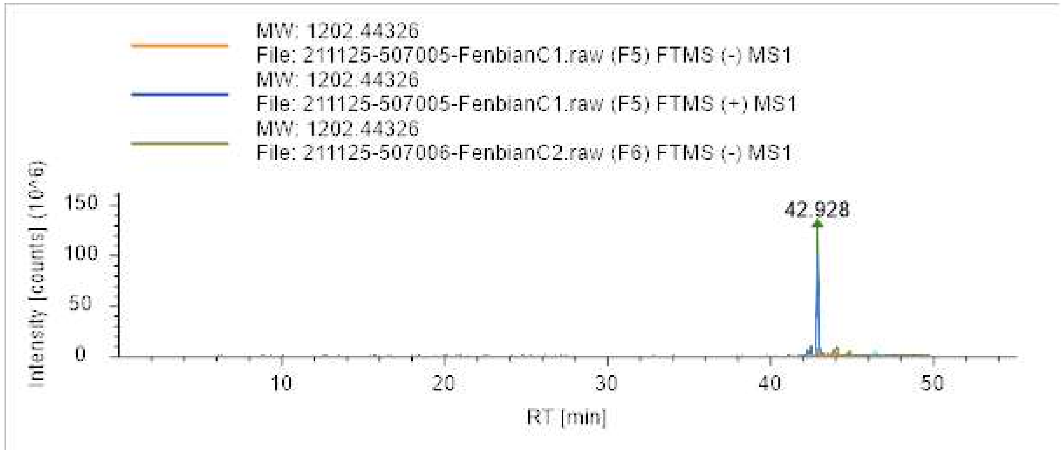

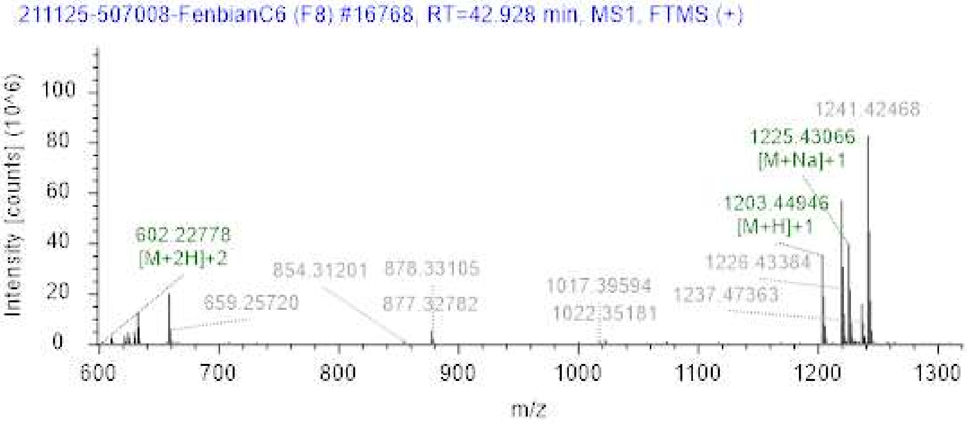

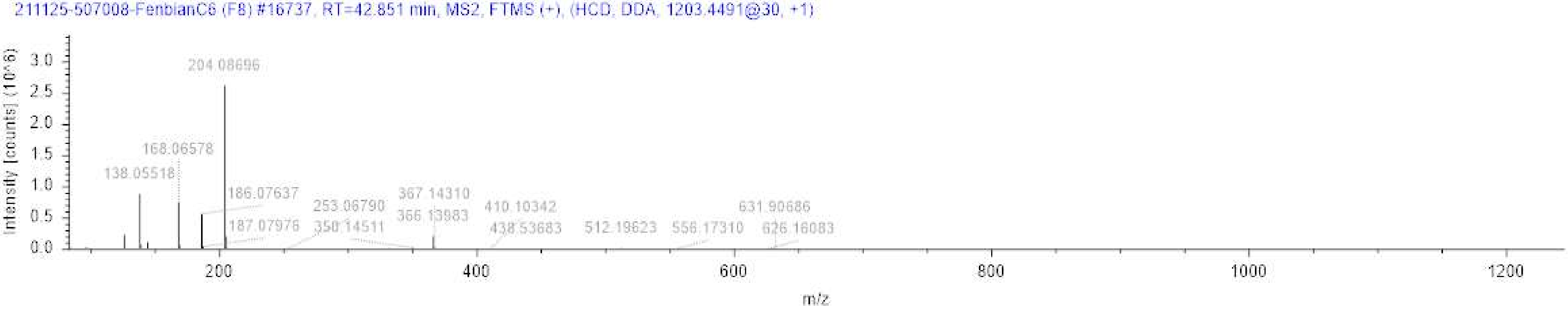

© Reported with Compound Discoverer 3.3

Compounds

Structure	Name	RT [min]	Formula	Calc. MW	Areas
n/a		44.13	n/a	1202.44371	1.17e8

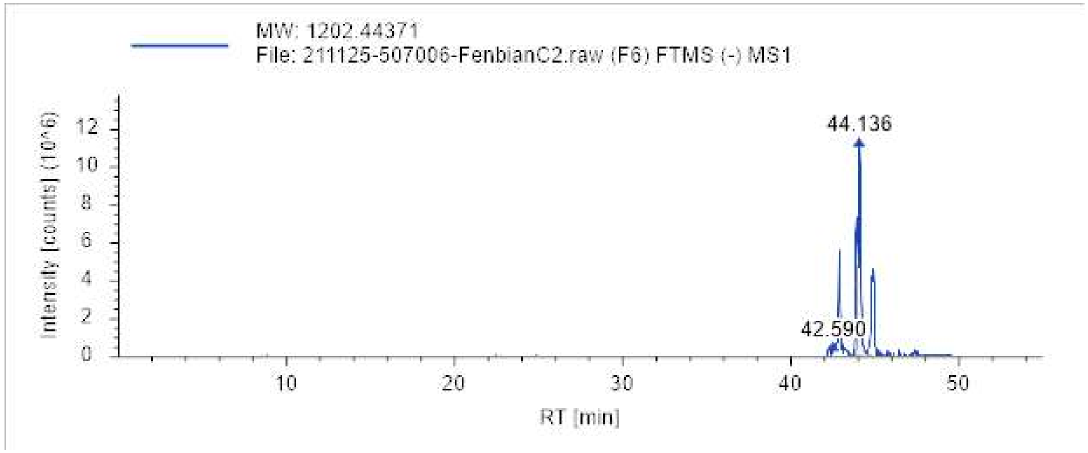

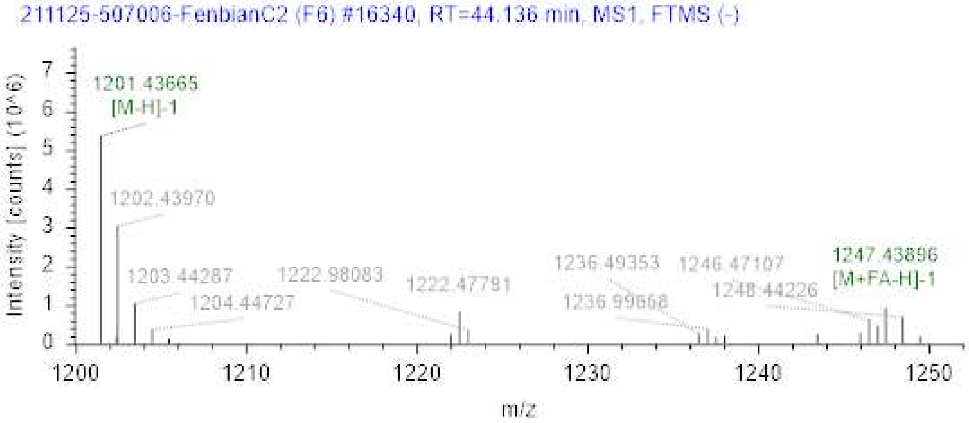

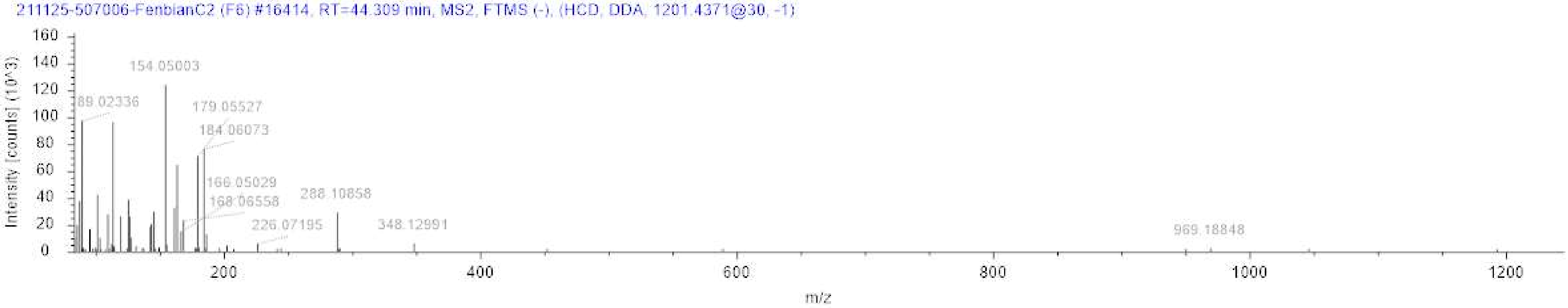

© Reported with Compound Discoverer 3.3

Structure	Name	RT [min]	Formula	Calc. MW	Areas
n/a		43.95	n/a	1218.43641	1.62e7; 1.48e8

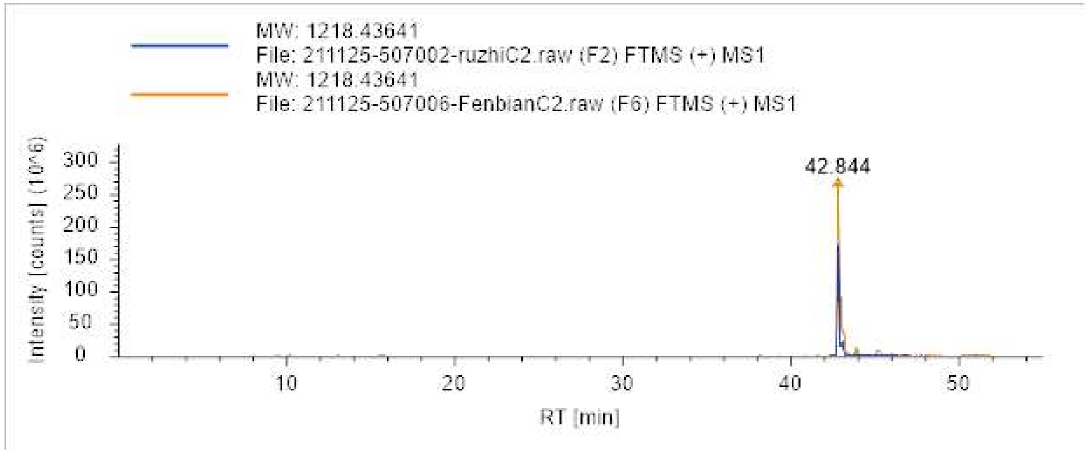

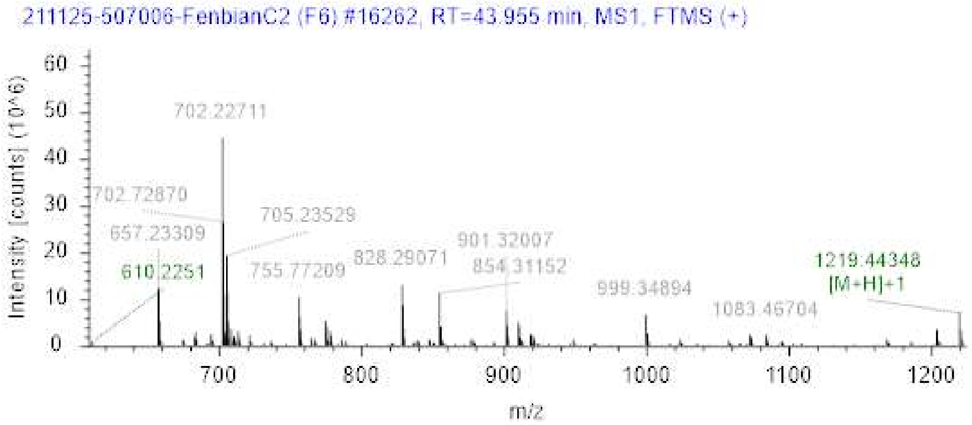

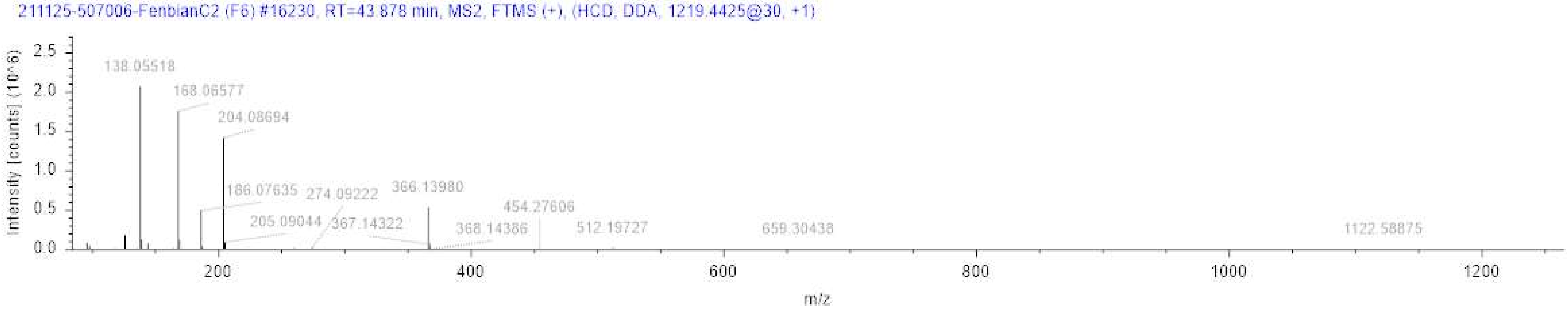

© Reported with Compound Discoverer 3.3

Compounds

Structure	Name	RT [min]	Formula	Calc. MW	Areas
n/a		42.86	n/a	1218.43699	1.60e9 4.46e9 4.79e8 1.16e9 5.85e9 9.28e9

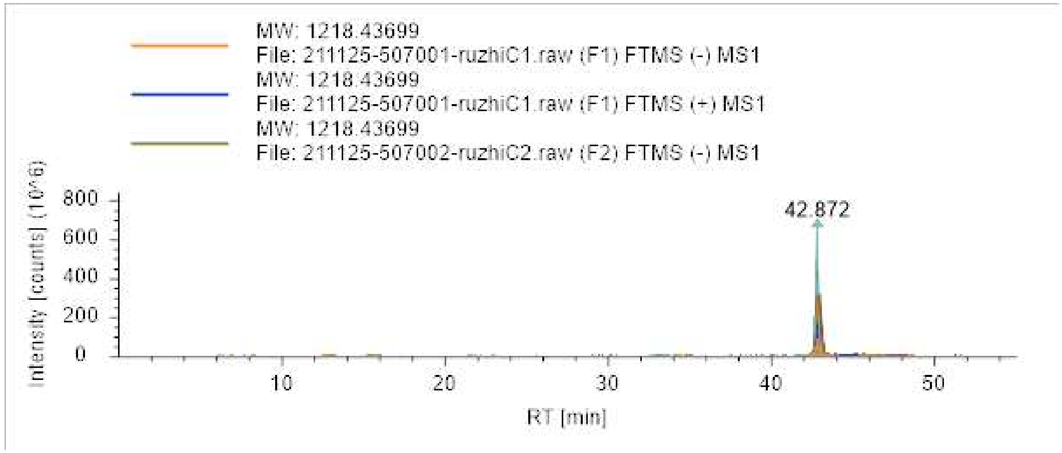

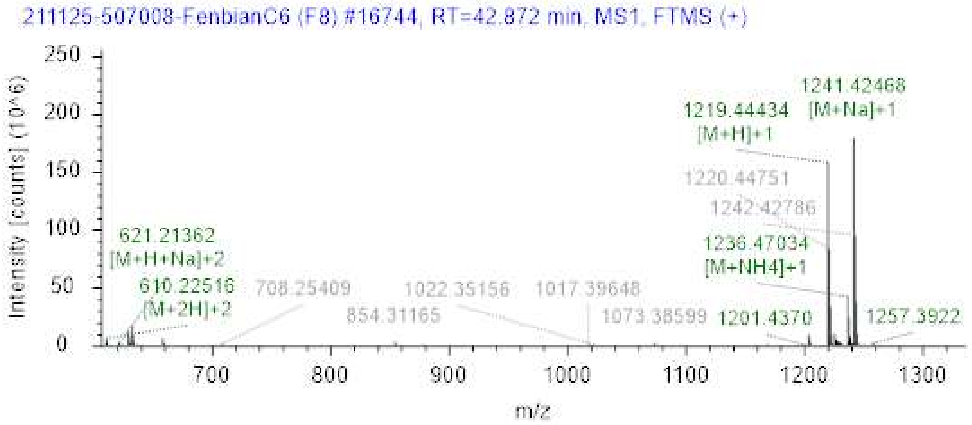

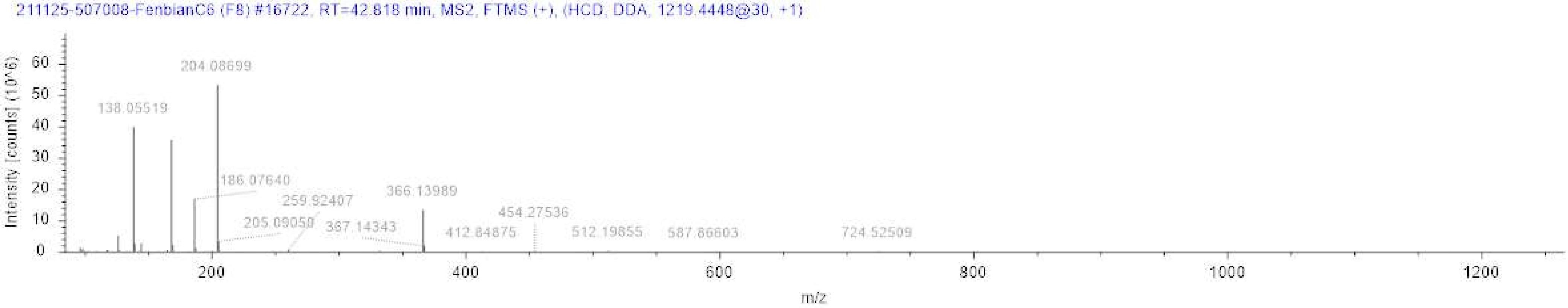

© Reported with Compound Discoverer 3.3

Compounds

Structure	Name	RT [min]	Formula	Calc. MW	Areas
n/a		45.21	n/a	1218.43700	2.29e7

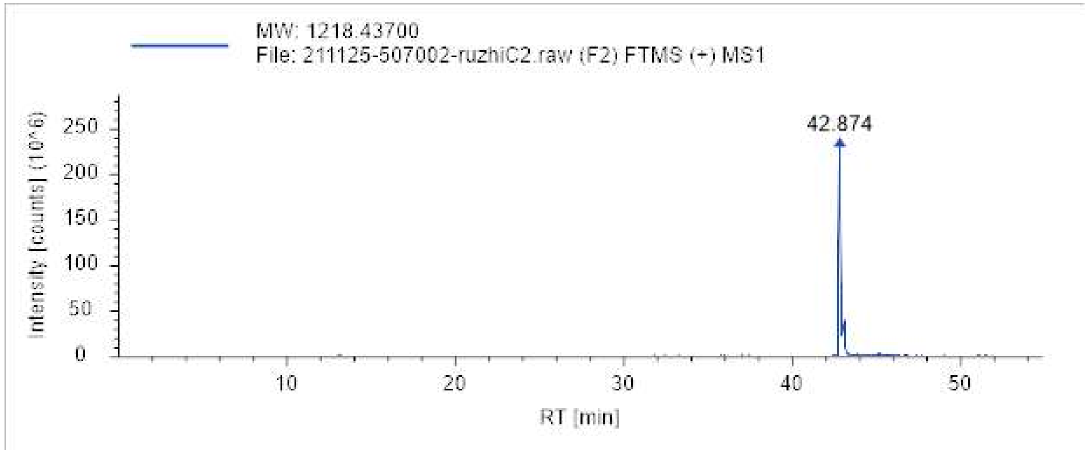

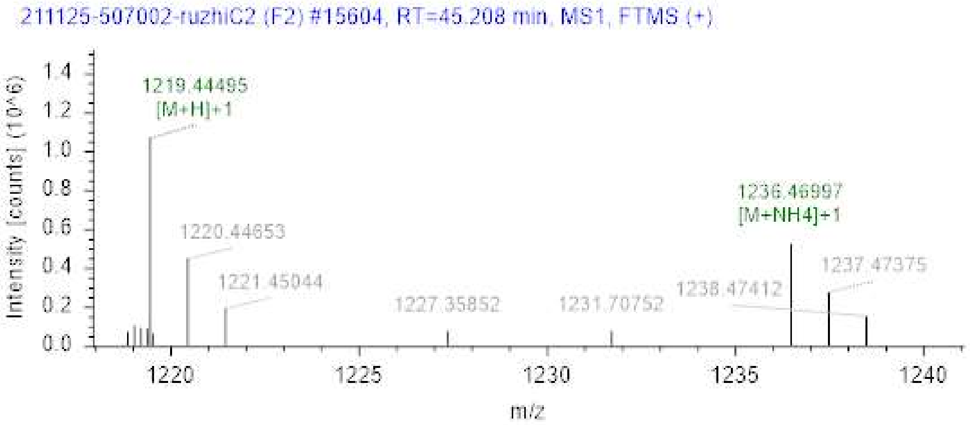

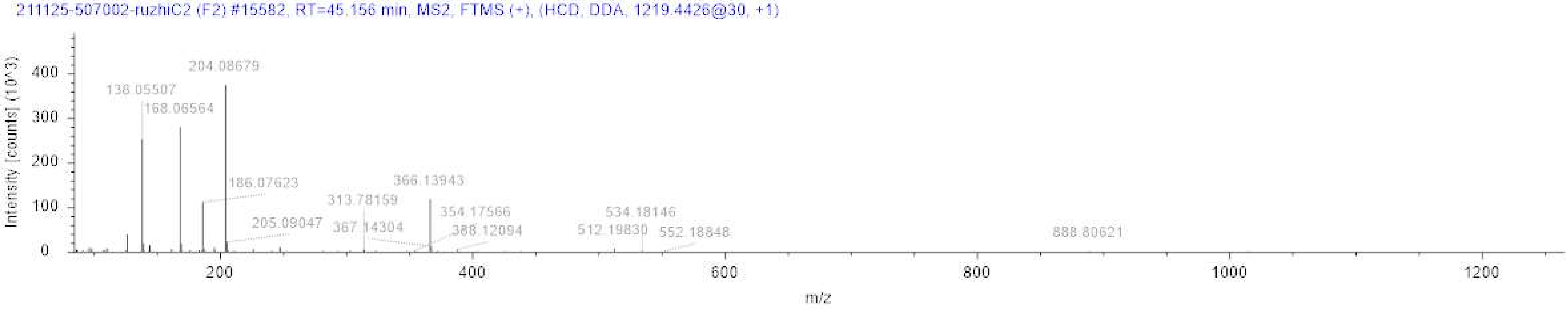

© Reported with Compound Discoverer 3.3

Compounds

Structure	Name	RT [min]	Formula	Calc. MW	Areas
n/a		42.88	n/a	1219.43784	3.96e6

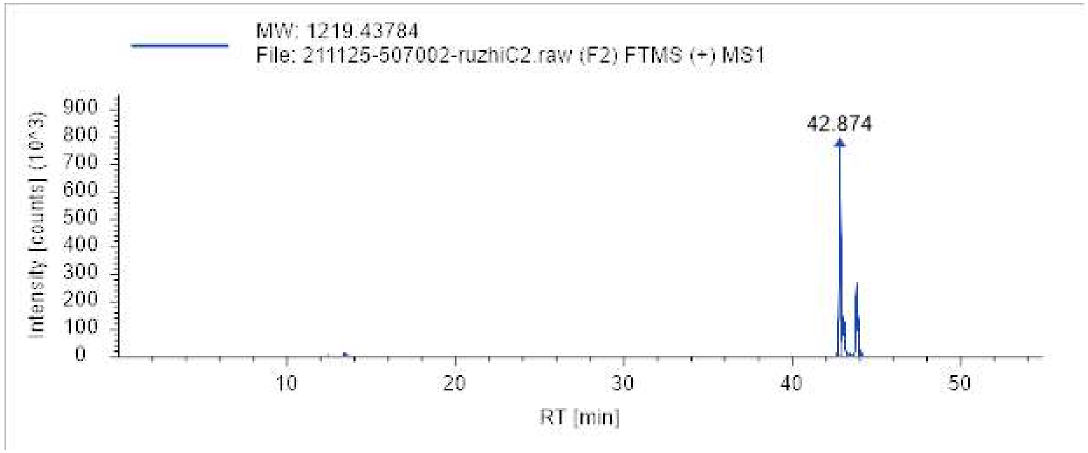

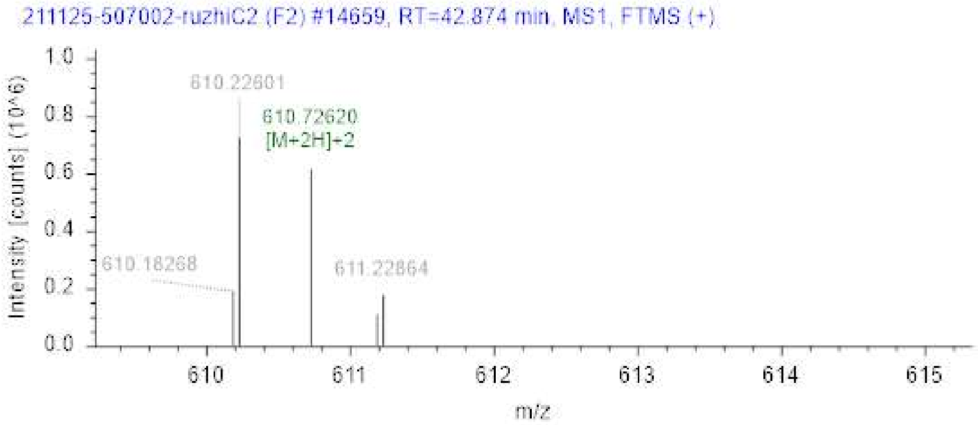

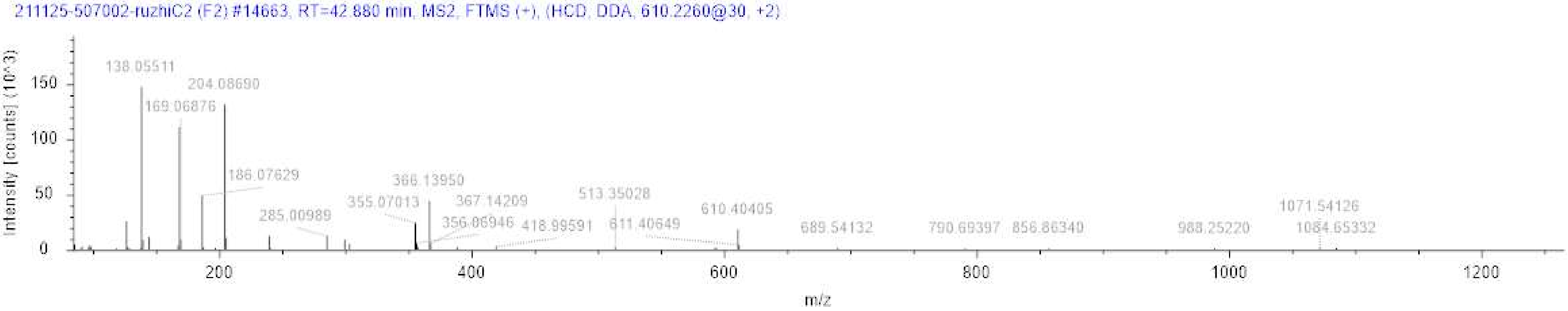

© Reported with Compound Discoverer 3.3

Structure	Name	RT [min]	Formula	Calc. MW	Areas
n/a		43.20	n/a	1234.43176	4.33e7

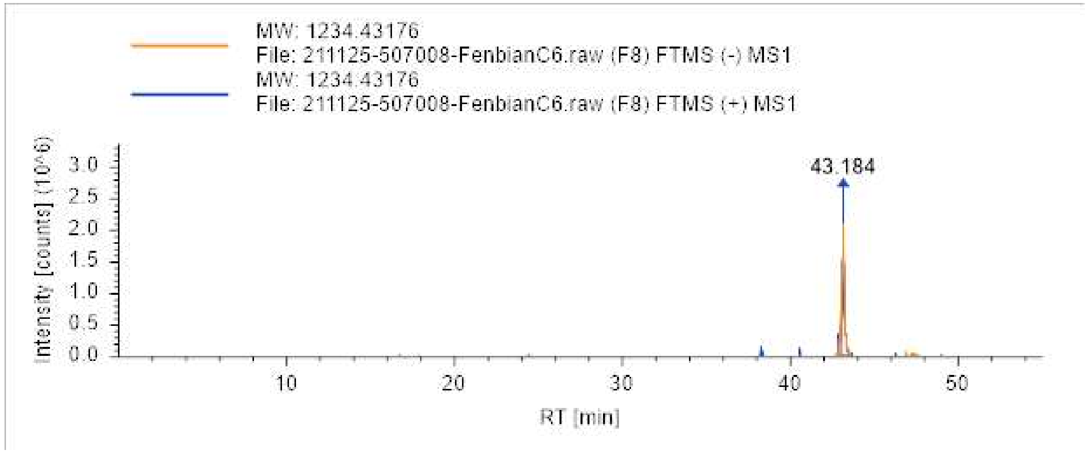

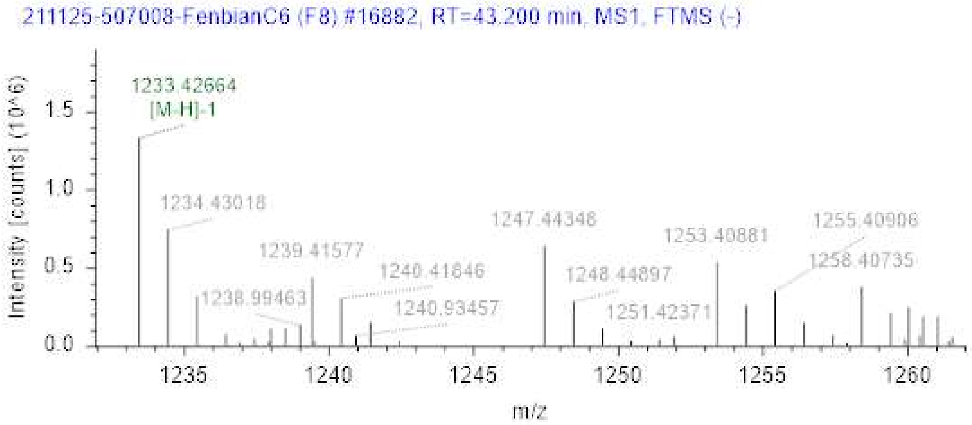

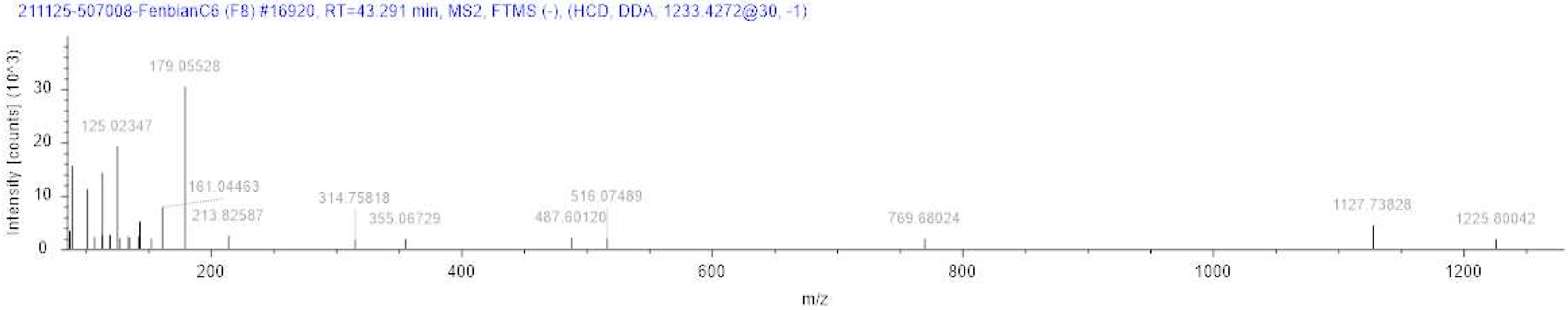

© Reported with Compound Discoverer 3.3

Structure	Name	RT [min]	Formula	Calc. MW	Areas
n/a		43.09	n/a	1248.41250	5.56e6

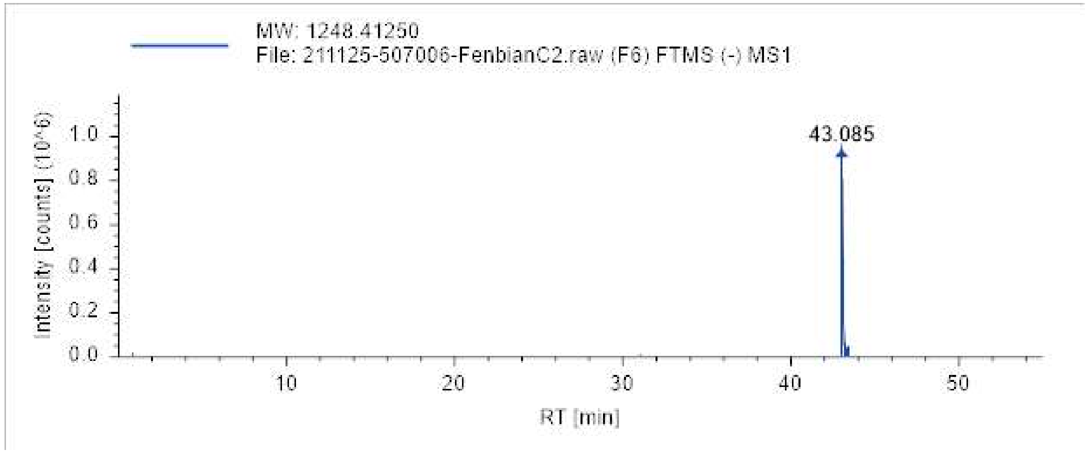

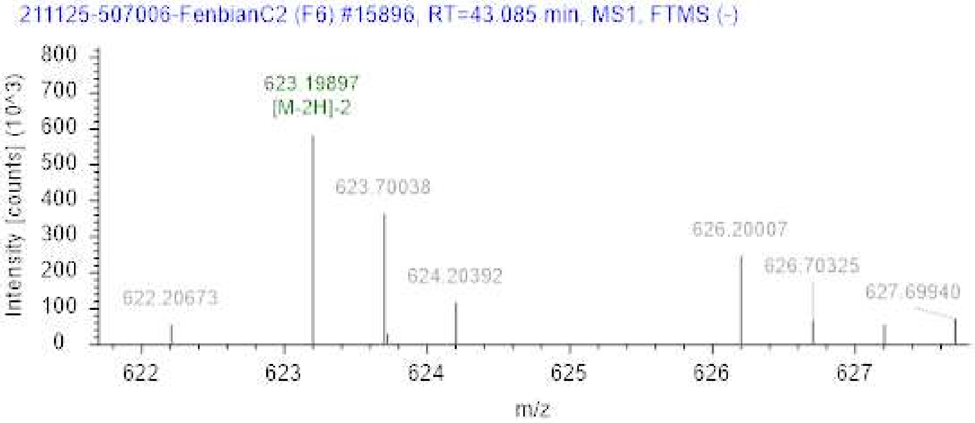

© Reported with Compound Discoverer 3.3

Compounds

Structure	Name	RT [min]	Formula	Calc. MW	Areas
n/a		41.94	n/a	1252.44522	1.43e7

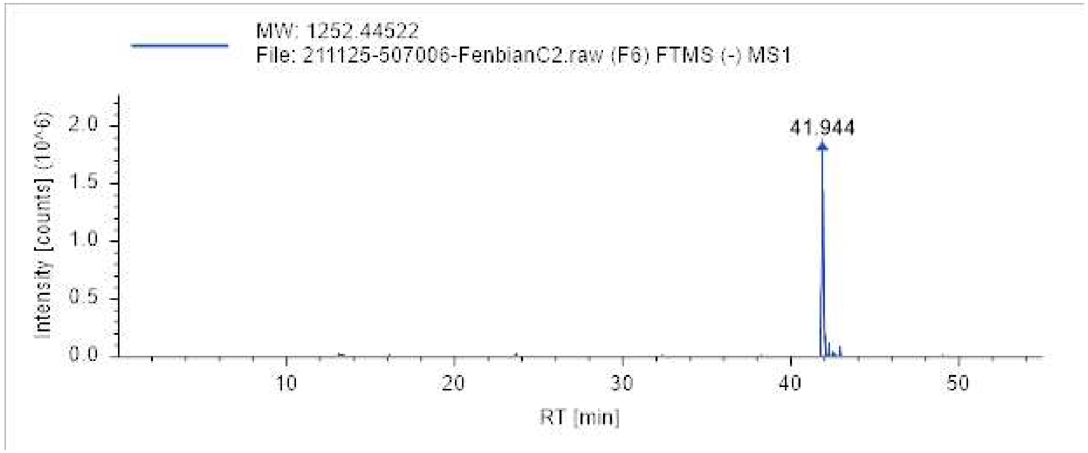

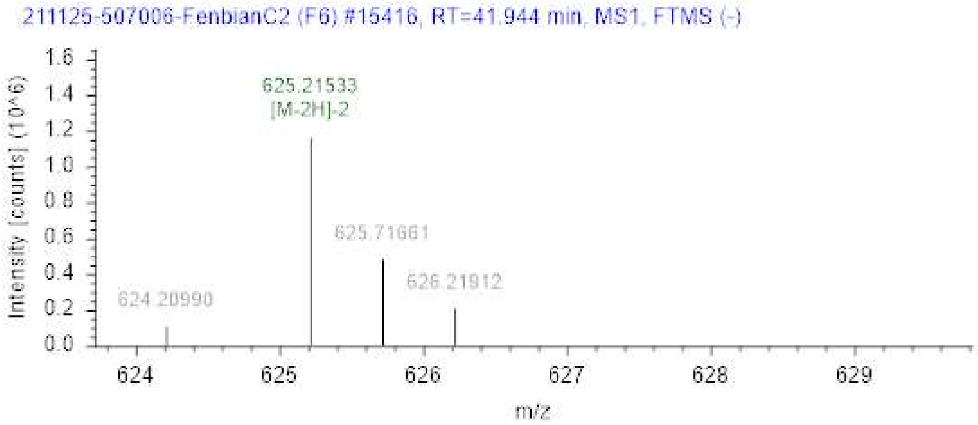

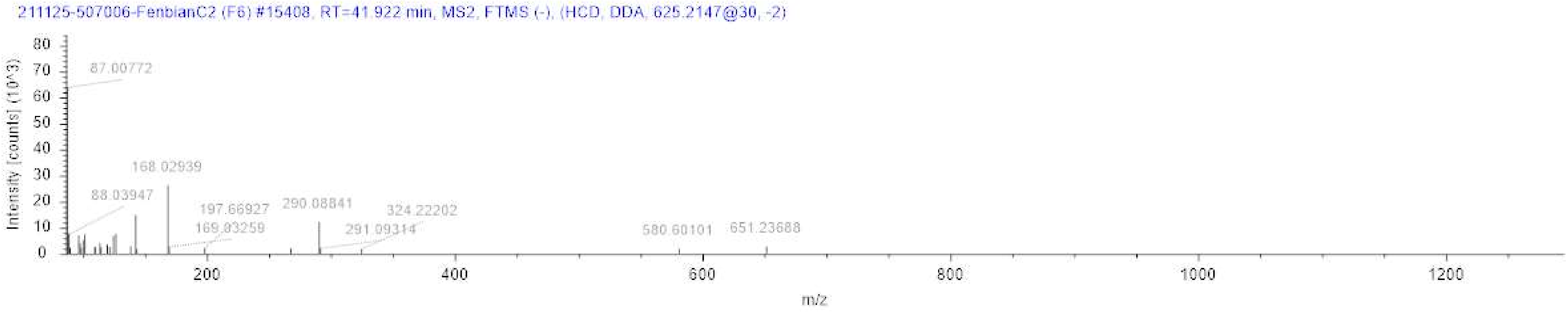

© Reported with Compound Discoverer 3.3

Structure	Name	RT [min]	Formula	Calc. MW	Areas
n/a		42.45	n/a	1259.46494	2.01e8

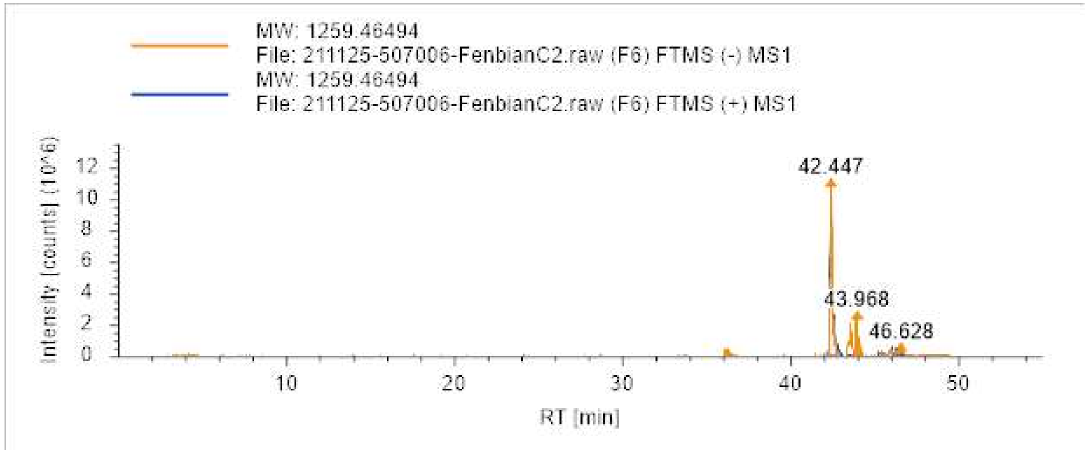

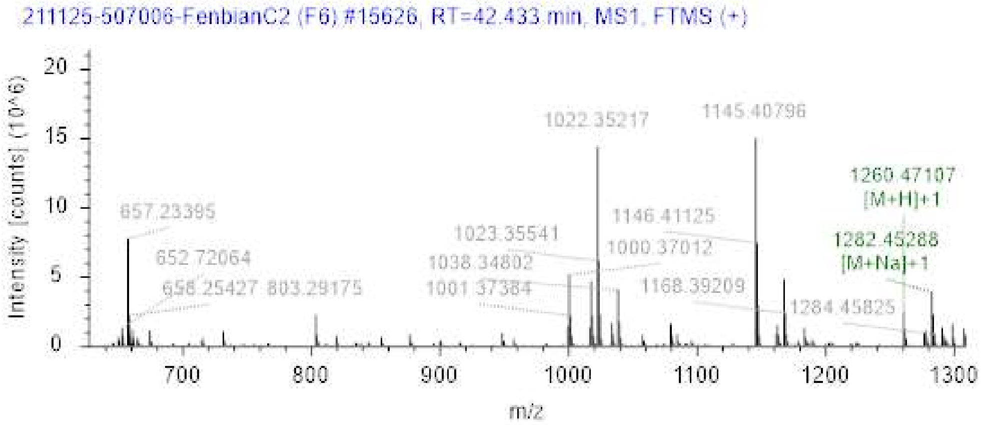

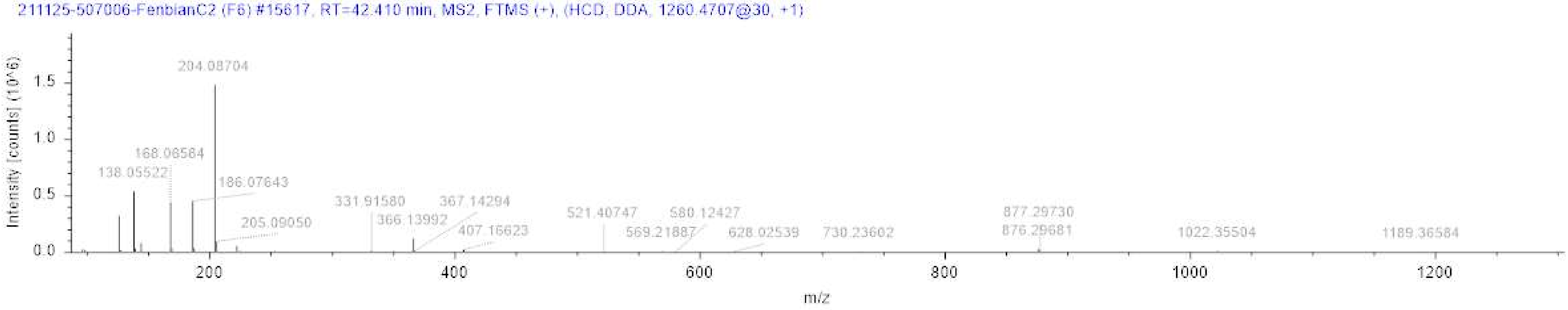

© Reported with Compound Discoverer 3.3

Structure	Name	RT [min]	Formula	Calc. MW	Areas
n/a		43.62	n/a	1259.46577	1.46e7

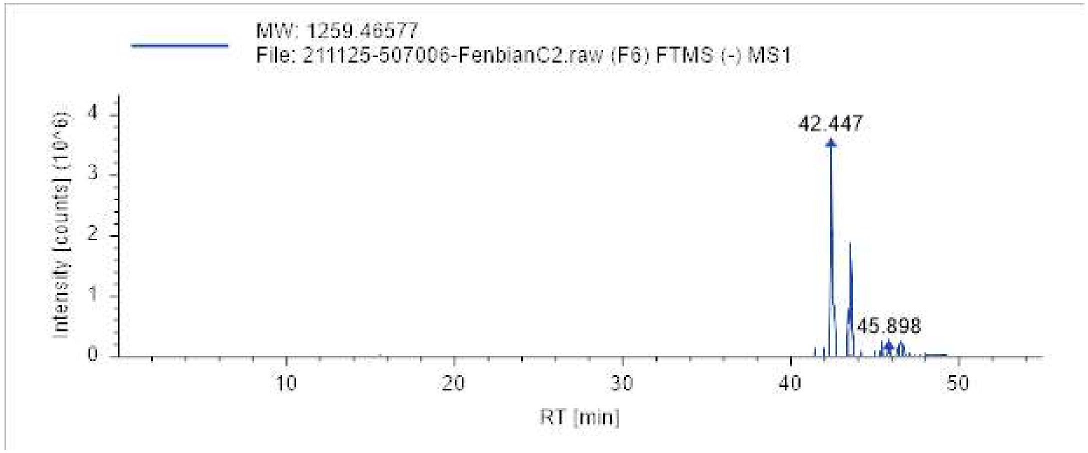

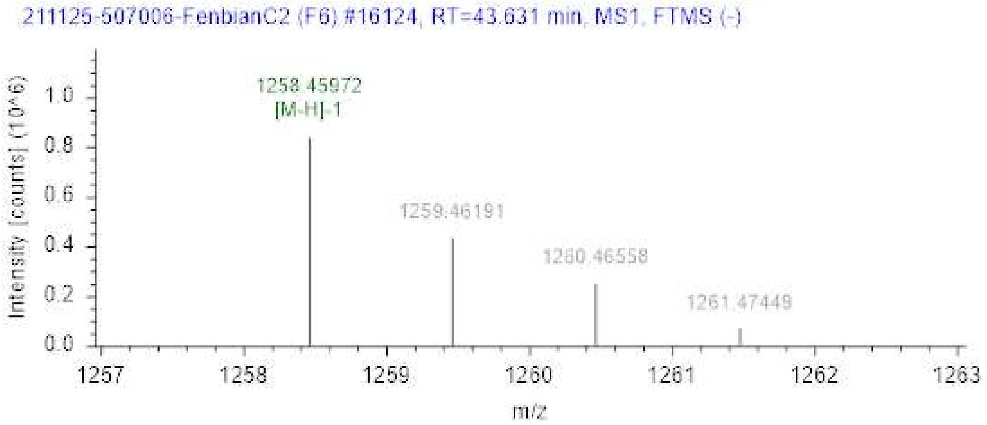

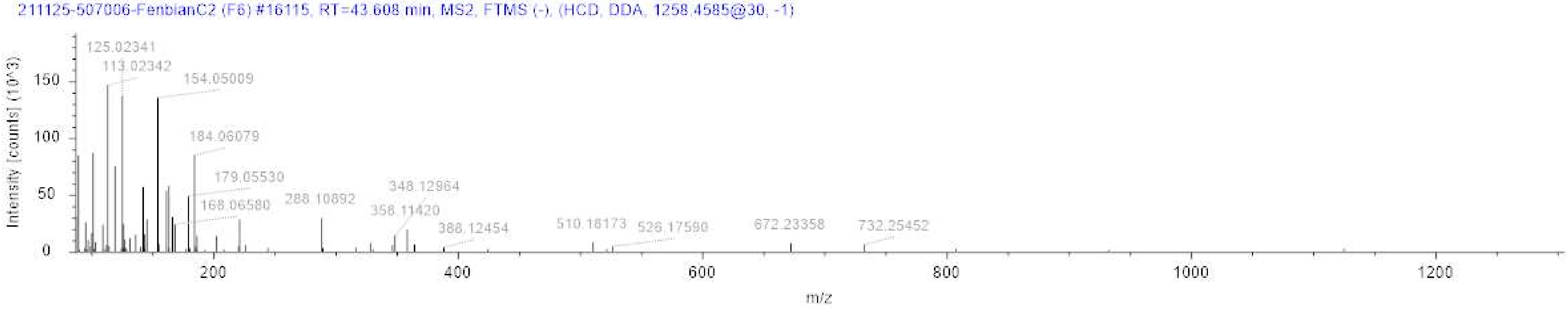

© Reported with Compound Discoverer 3.3

Compounds

Structure	Name	RT [min]	Formula	Calc. MW	Areas
n/a		42.52	n/a	1275.46030	4.25e8 1.22e7 7.91e7

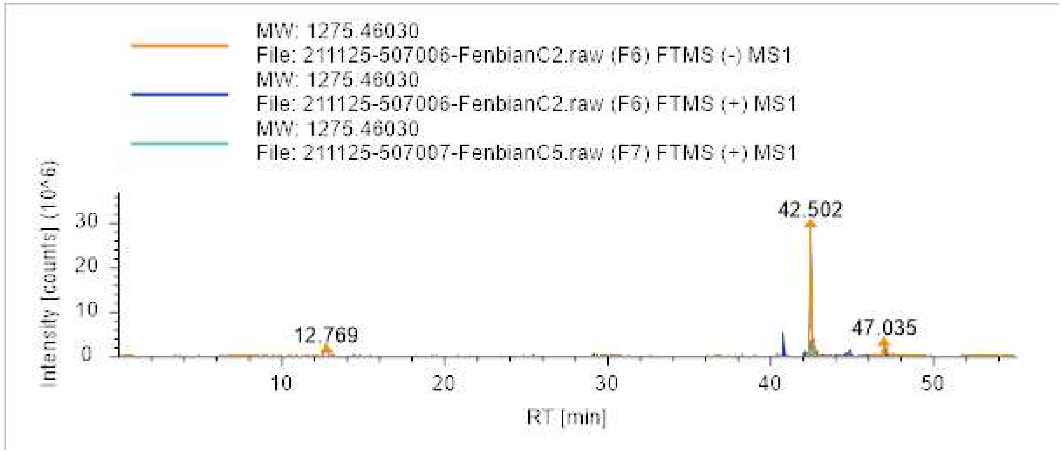

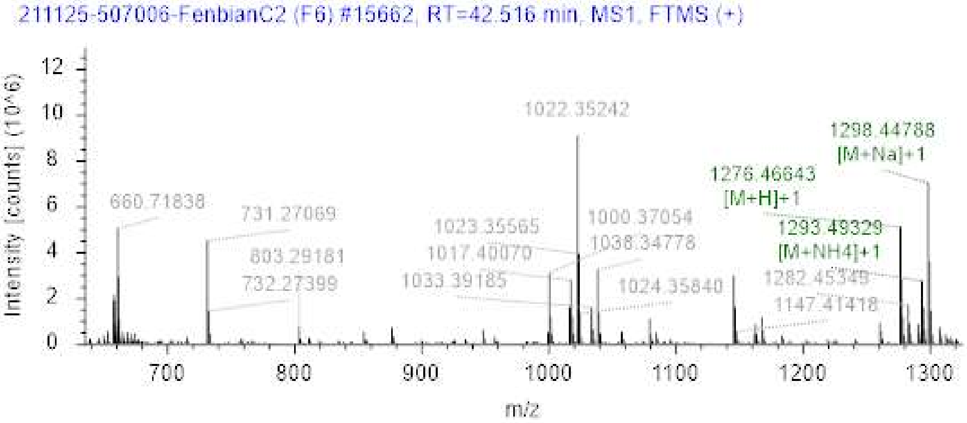

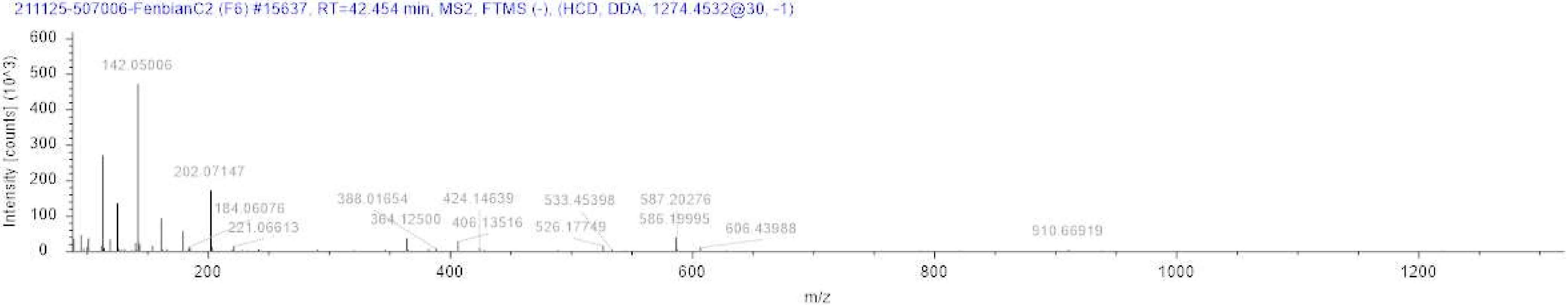

© Reported with Compound Discoverer 3.3

Structure	Name	RT [min]	Formula	Calc. MW	Areas
n/a		42.20	n/a	1289.43493	2.13e8 3.21e8 4.83e7 8.22e7 1.23e8

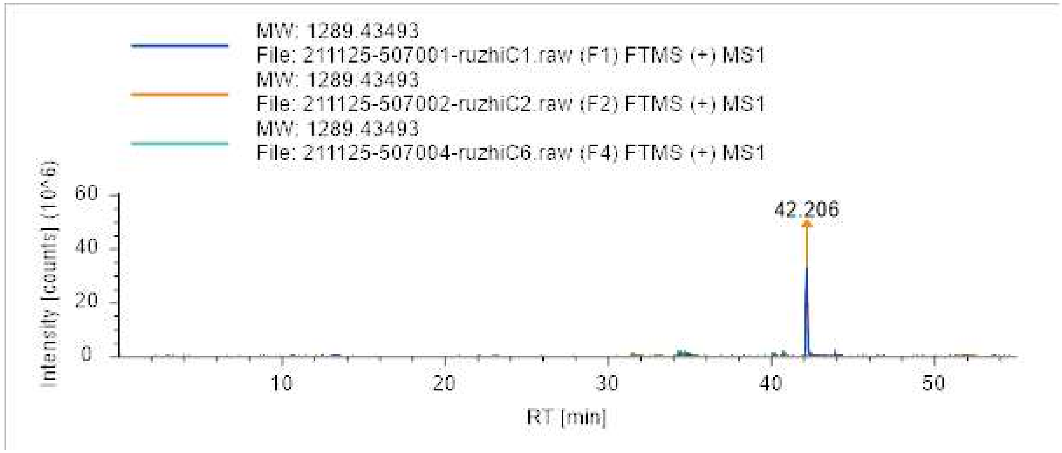

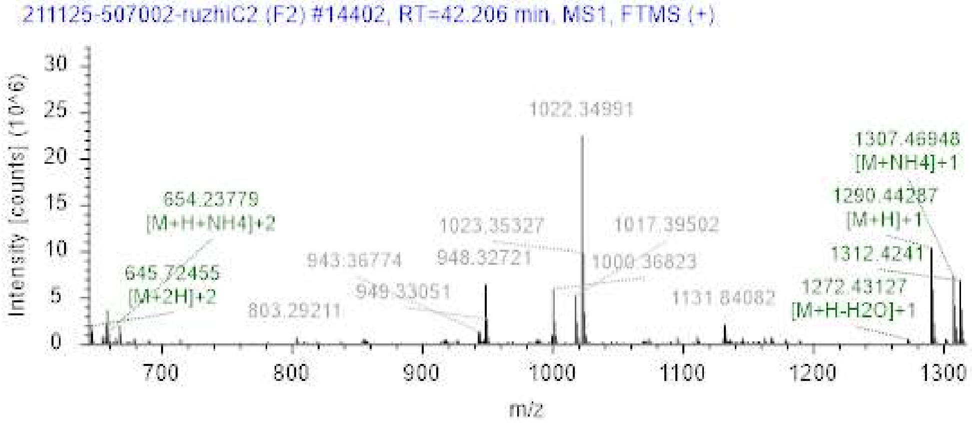

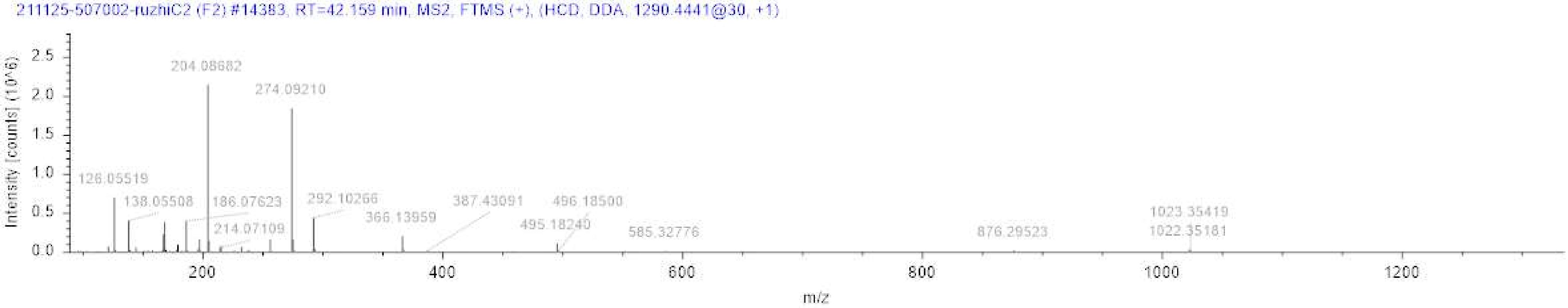

© Reported with Compound Discoverer 3.3

Structure	Name	RT [min]	Formula	Calc. MW	Areas
n/a		43.92	n/a	1289.43795	3.10e7 2.88e7

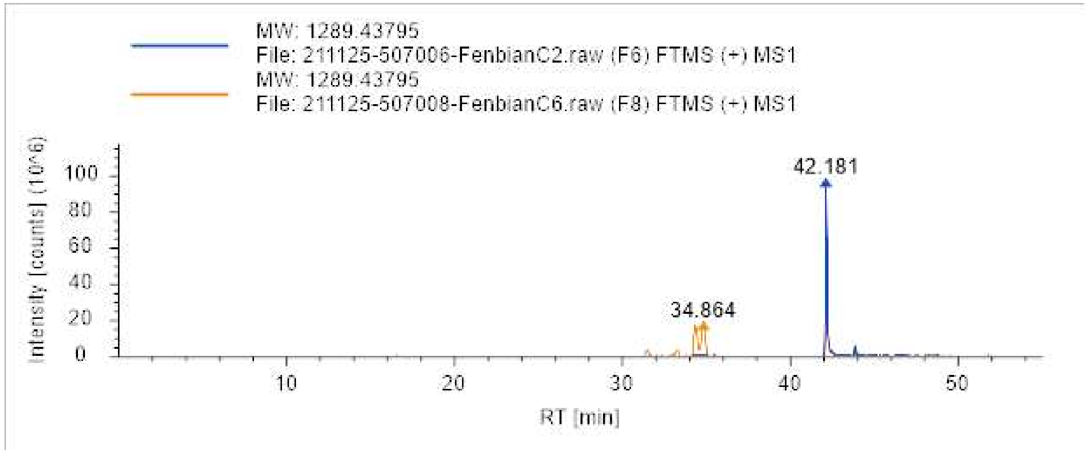

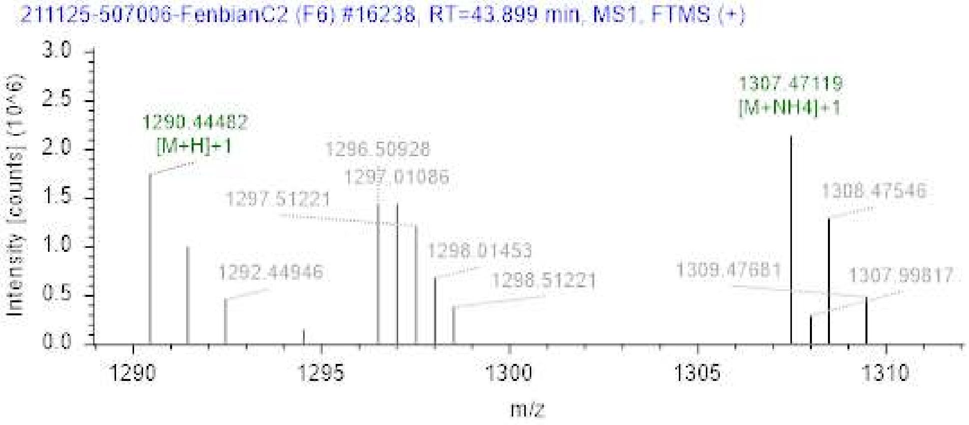

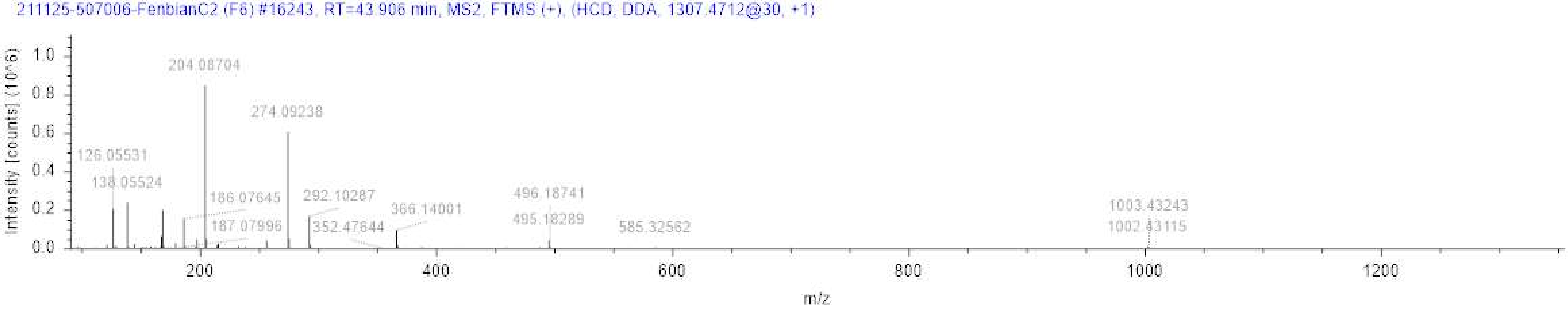

© Reported with Compound Discoverer 3.3

Compounds

Structure	Name	RT [min]	Formula	Calc. MW	Areas
n/a		42.19	n/a	1306.46194	6.62e6; 4.01e8

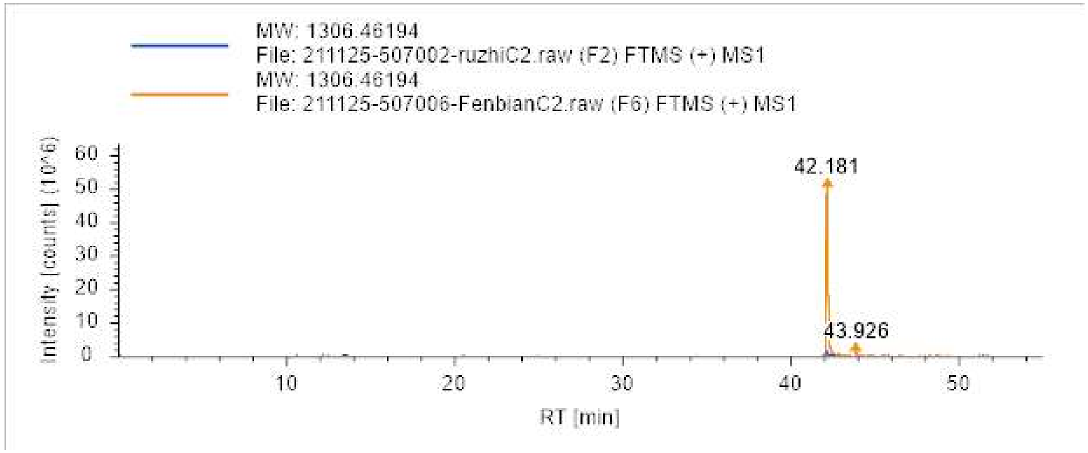

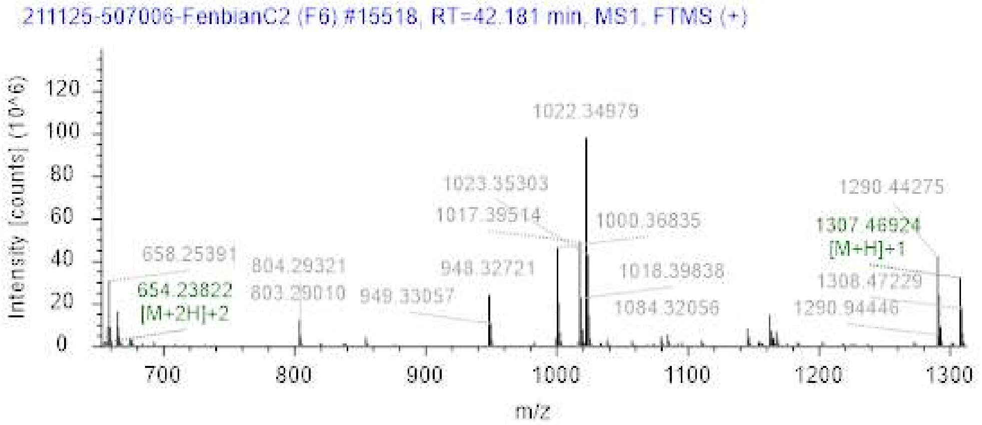

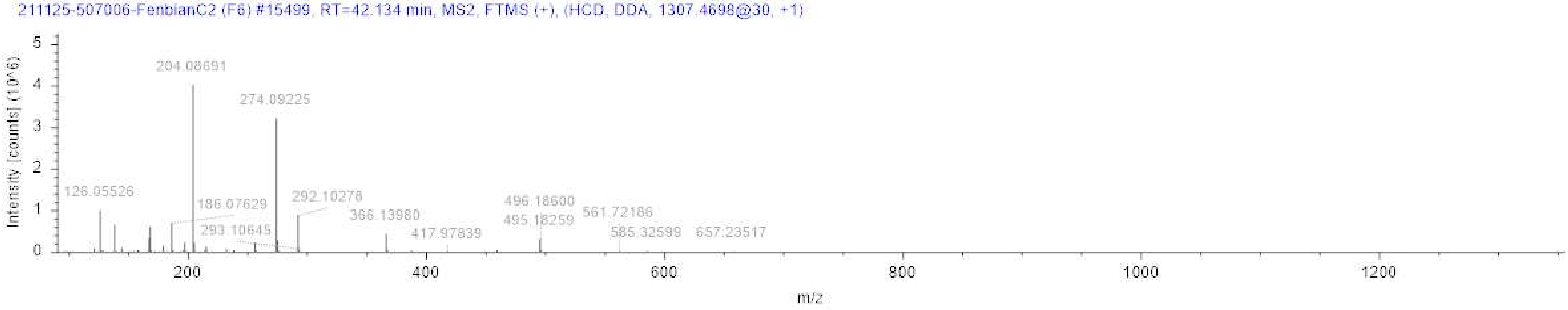

© Reported with Compound Discoverer 3.3

Structure	Name	RT [min]	Formula	Calc. MW	Areas
n/a		43.29	n/a	1347.47971	1.54e7

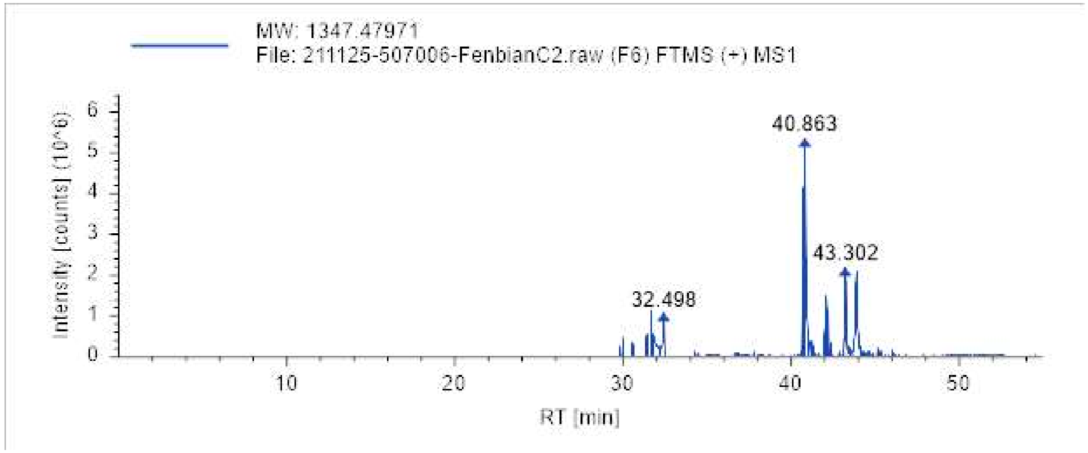

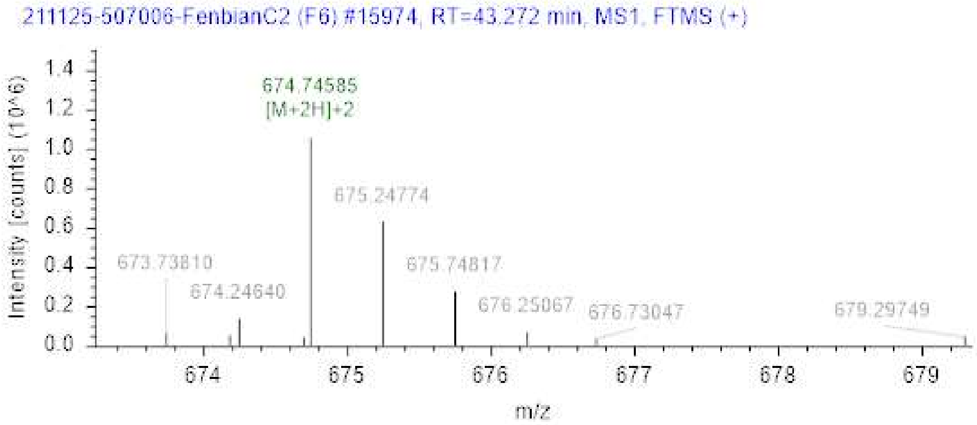

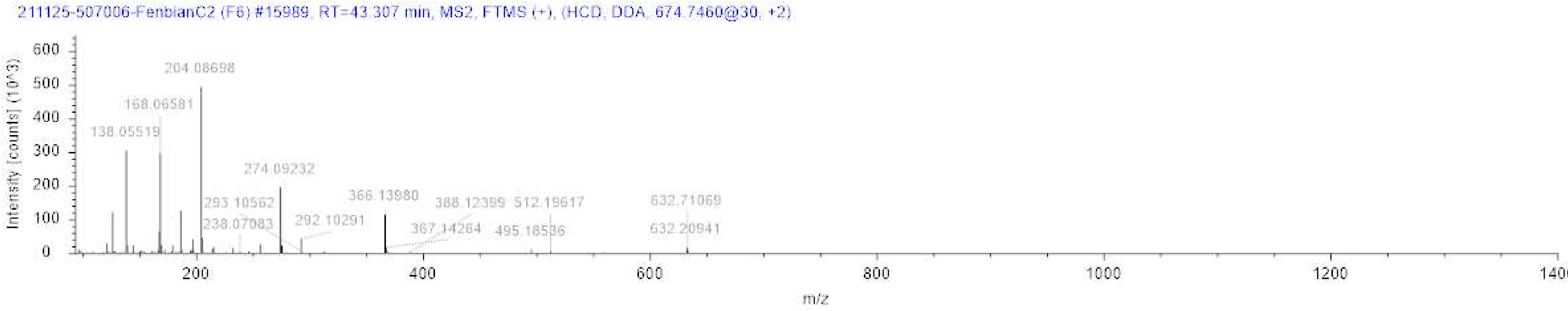

© Reported with Compound Discoverer 3.3

Structure	Name	RT [min]	Formula	Calc. MW	Areas
n/a		42.32	n/a	1347.48026	1.18e8 2.15e7

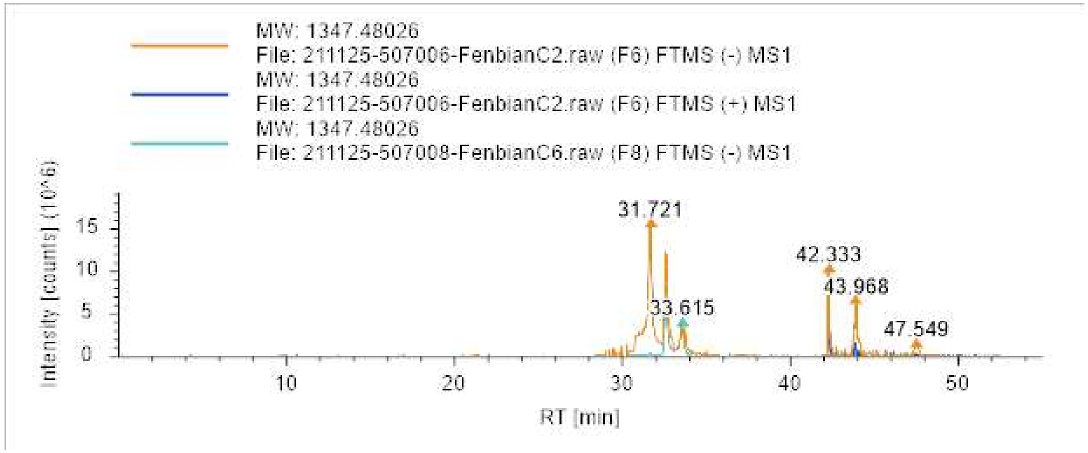

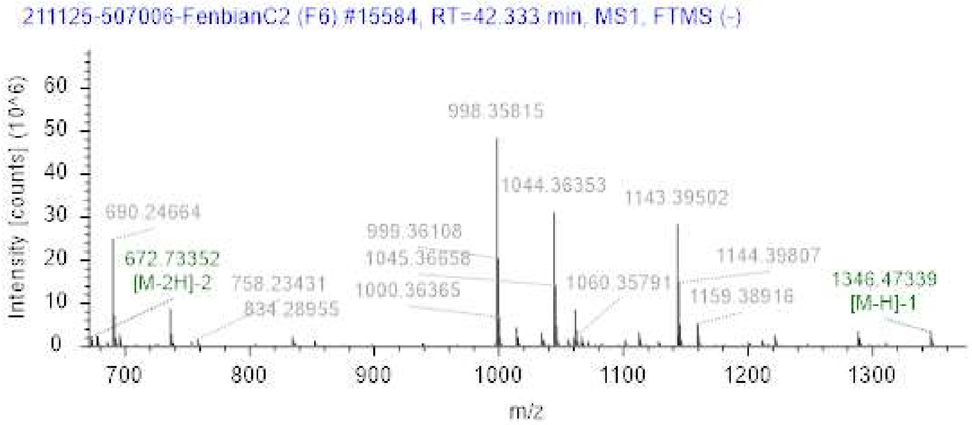

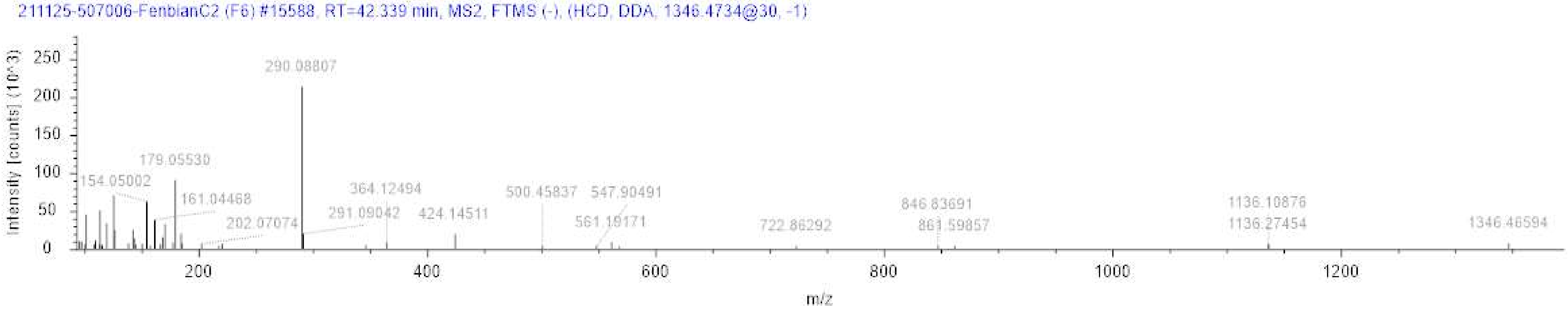

© Reported with Compound Discoverer 3.3

Compounds

Structure	Name	RT [min]	Formula	Calc. MW	Areas
n/a		44.75	n/a	1348.50112	1.55e7

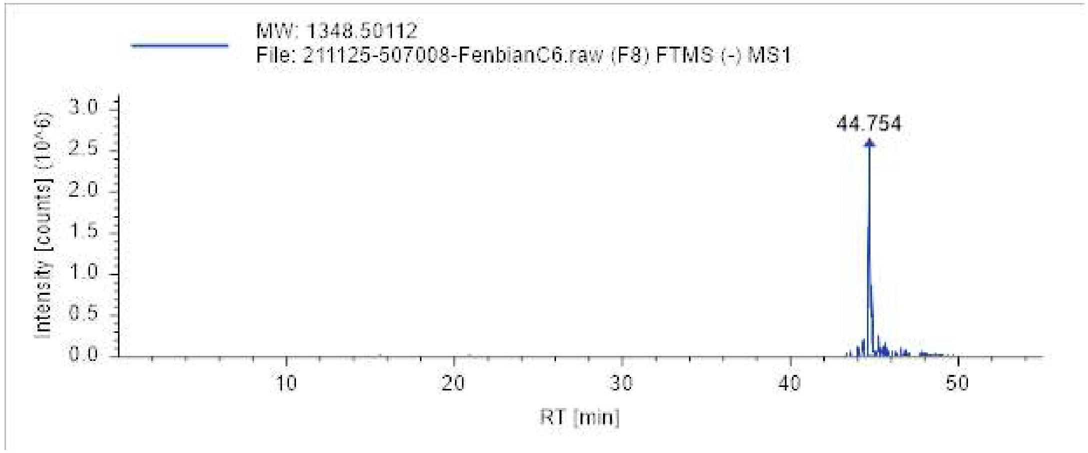

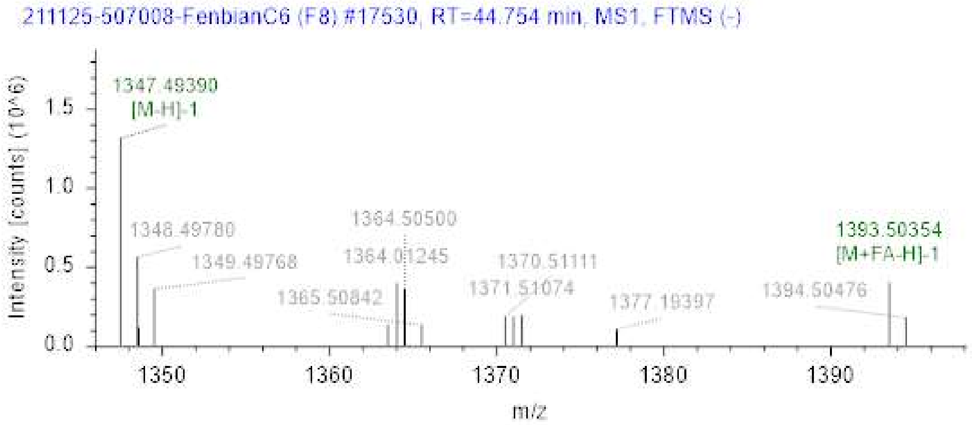

© Reported with Compound Discoverer 3.3

Compounds

Structure	Name	RT [min]	Formula	Calc. MW	Areas
n/a		43.82	n/a	1363.47222	8.36e6 1.14e8 8.21e7

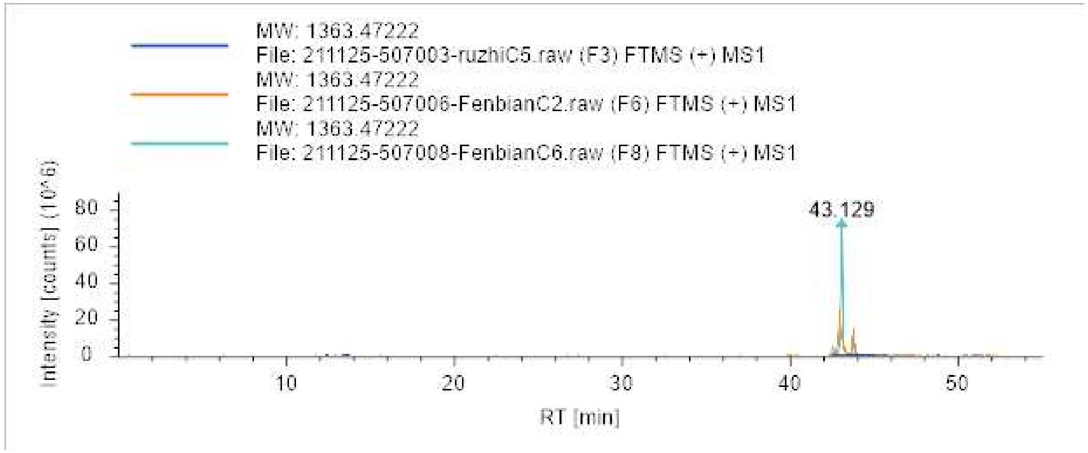

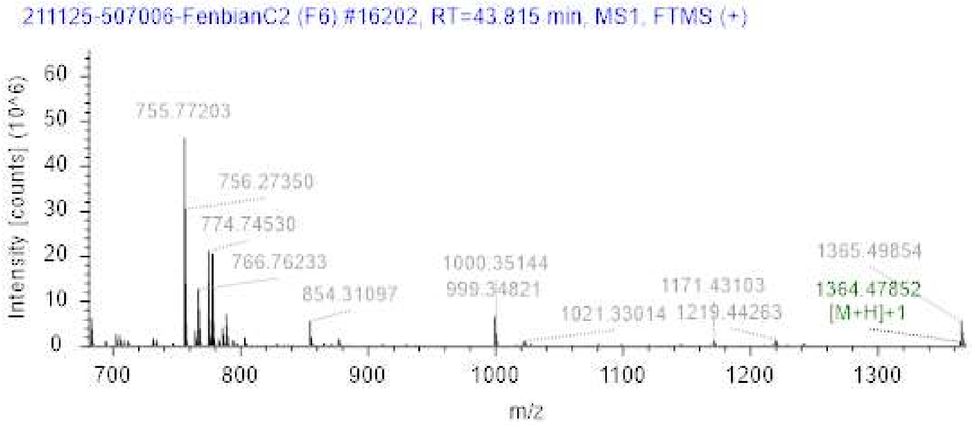

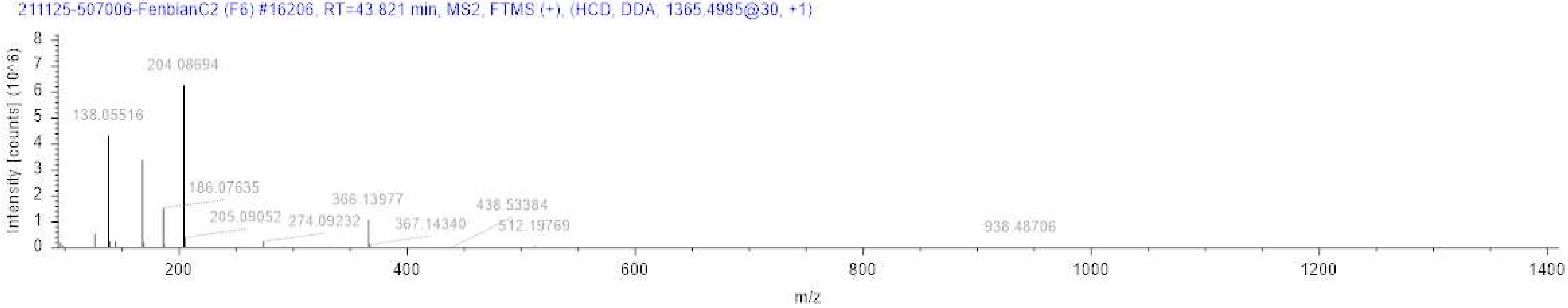

© Reported with Compound Discoverer 3.3

Compounds

Structure	Name	RT [min]	Formula	Calc. MW	Areas
n/a		45.00	n/a	1363.47392	1.56e7

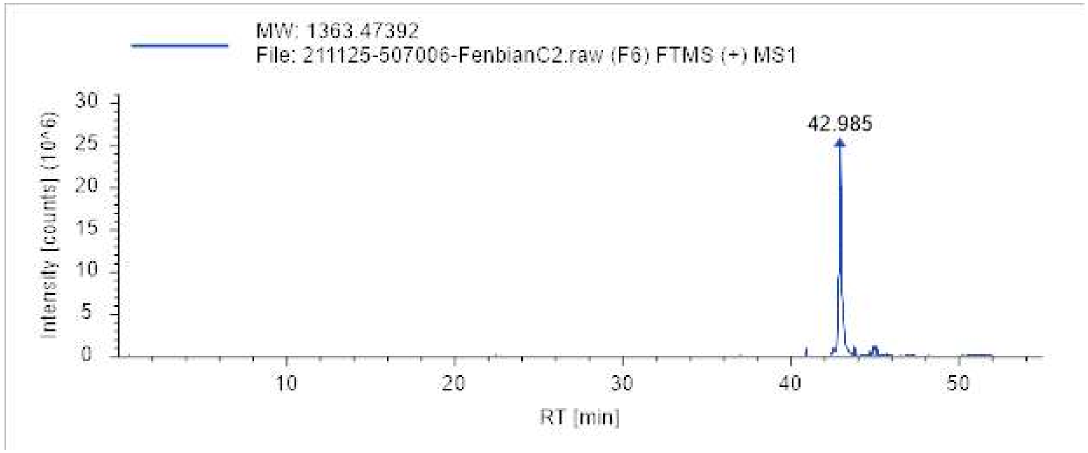

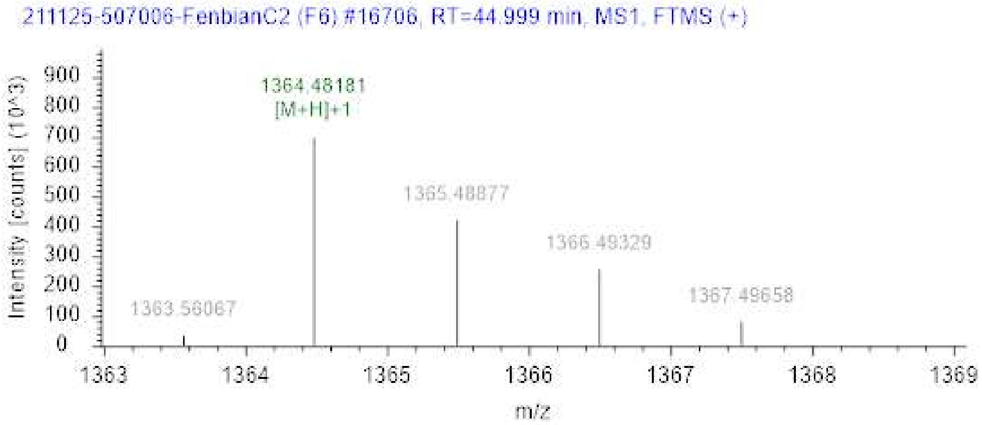

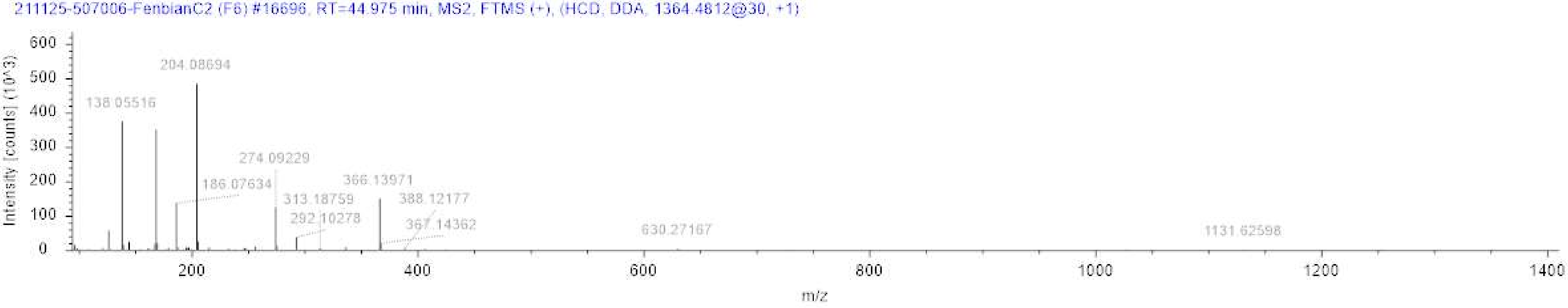

© Reported with Compound Discoverer 3.3

Compounds

Structure	Name	RT [min]	Formula	Calc. MW	Areas
n/a		43.08	n/a	1363.47552	1.94e8 2.65e8 7.59e7 3.91e8 1.55e9 2.59e9

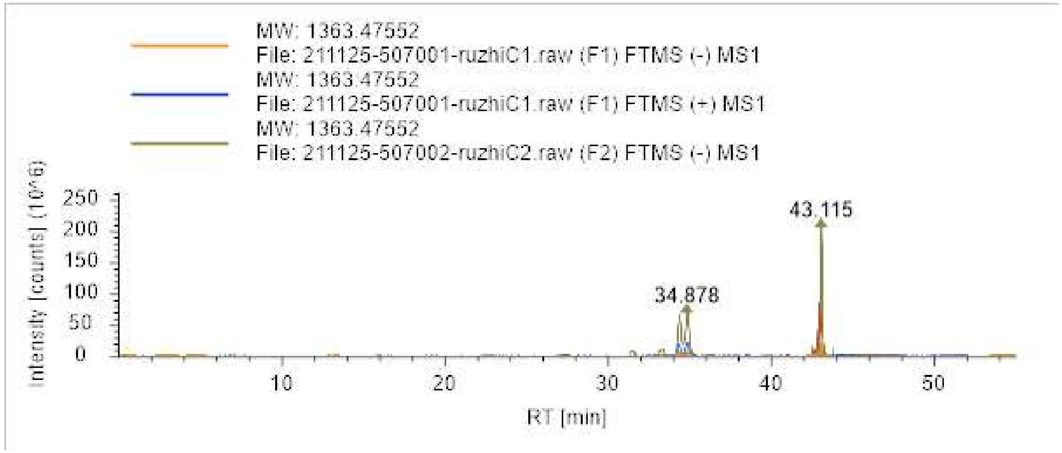

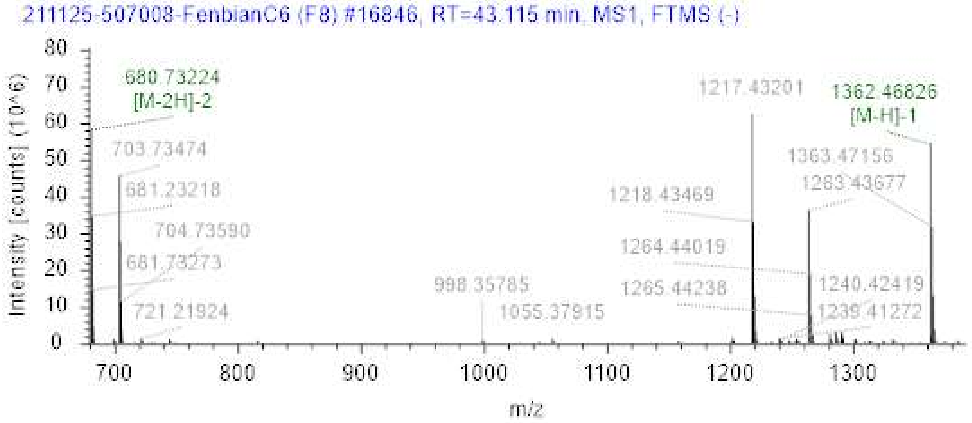

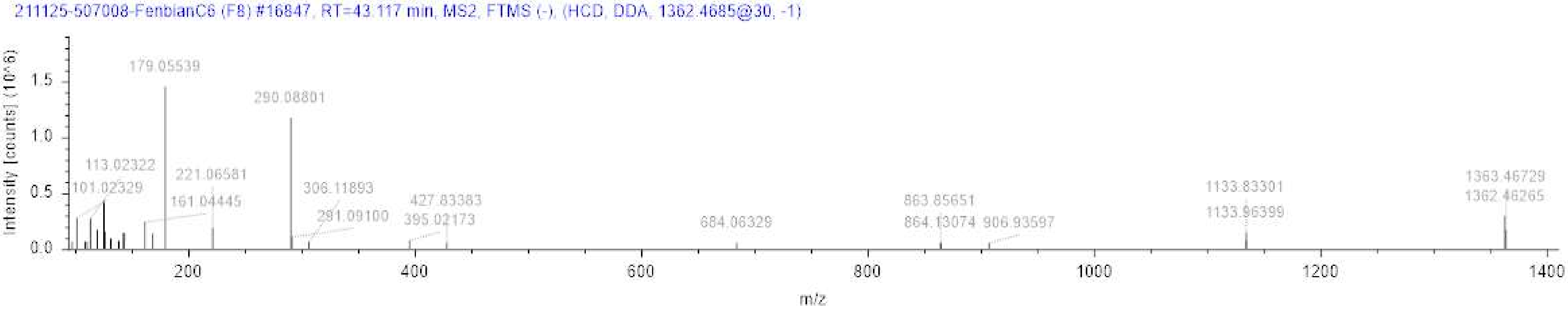

© Reported with Compound Discoverer 3.3

Structure	Name	RT [min]	Formula	Calc. MW	Areas
n/a		40.83	n/a	1364.49286	1.04e8 1.89e7

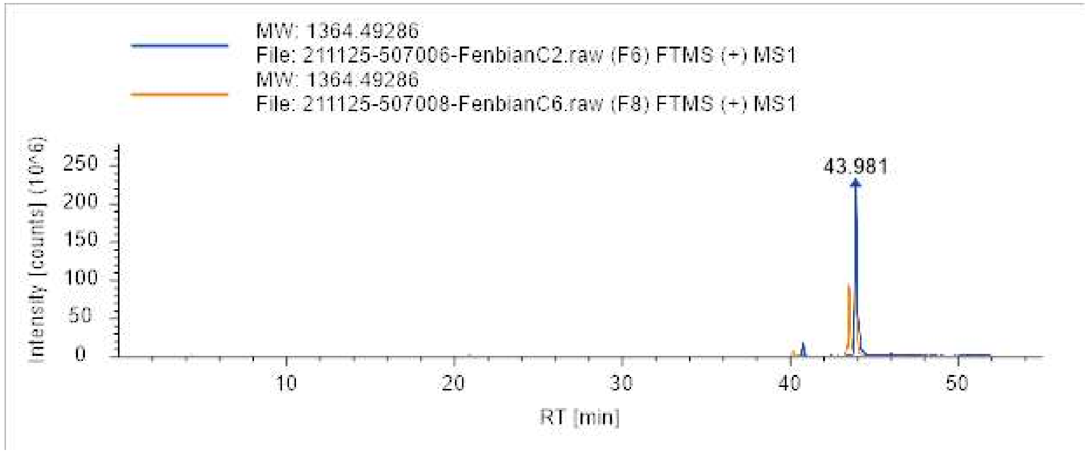

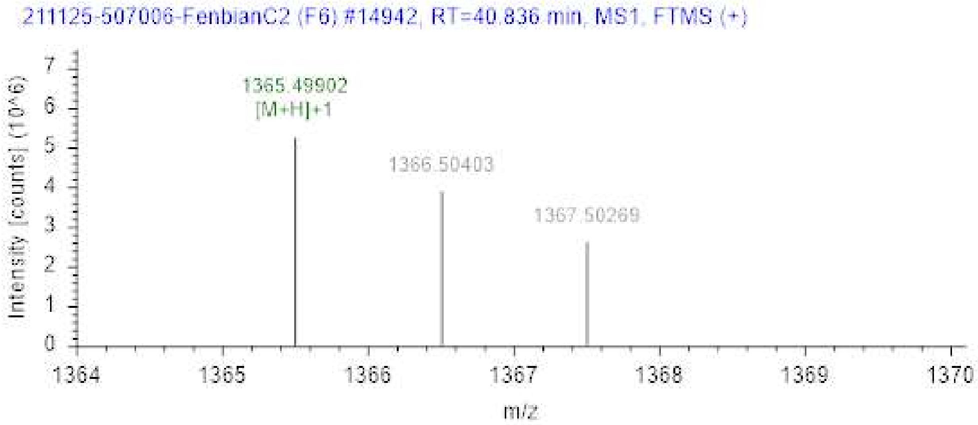

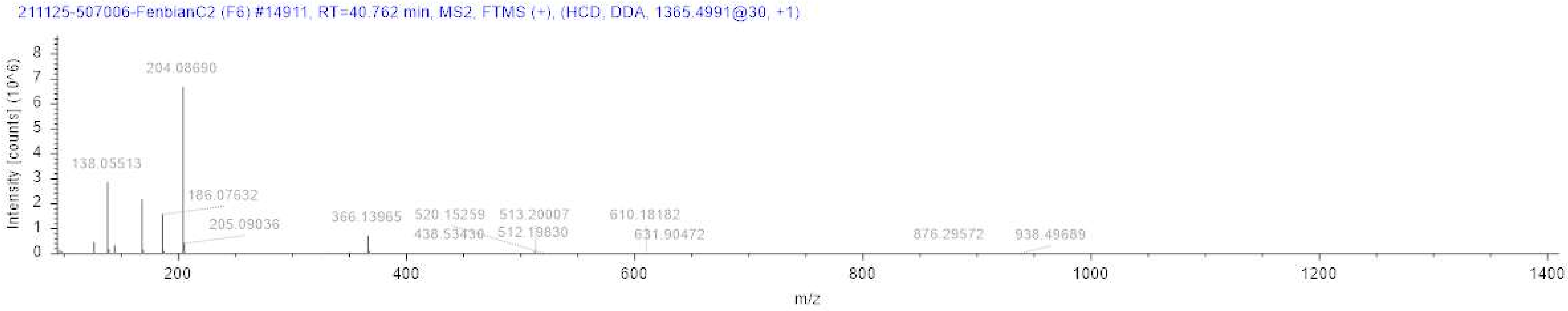

© Reported with Compound Discoverer 3.3

Compounds

Structure	Name	RT [min]	Formula	Calc. MW	Areas
n/a		44.74	n/a	1364.49314	2.26e7

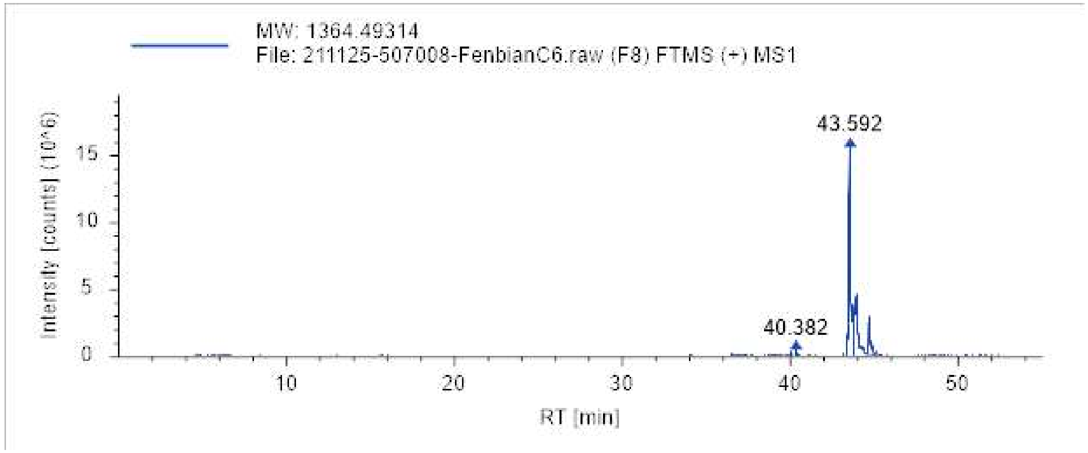

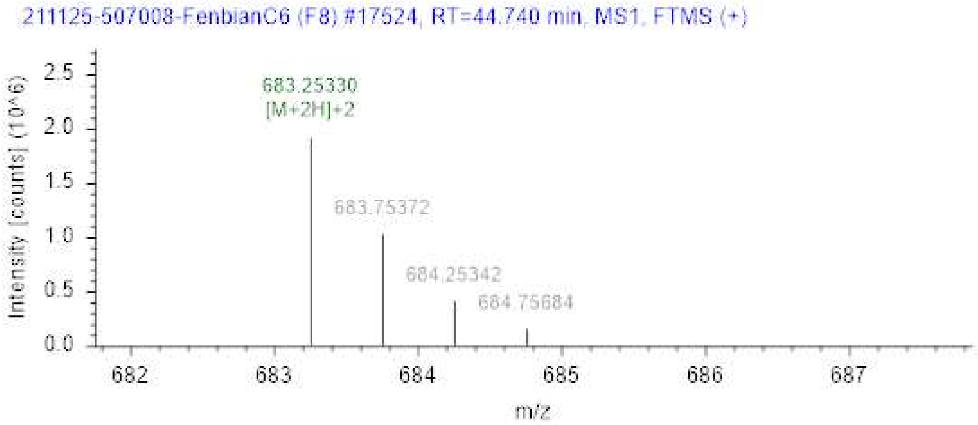

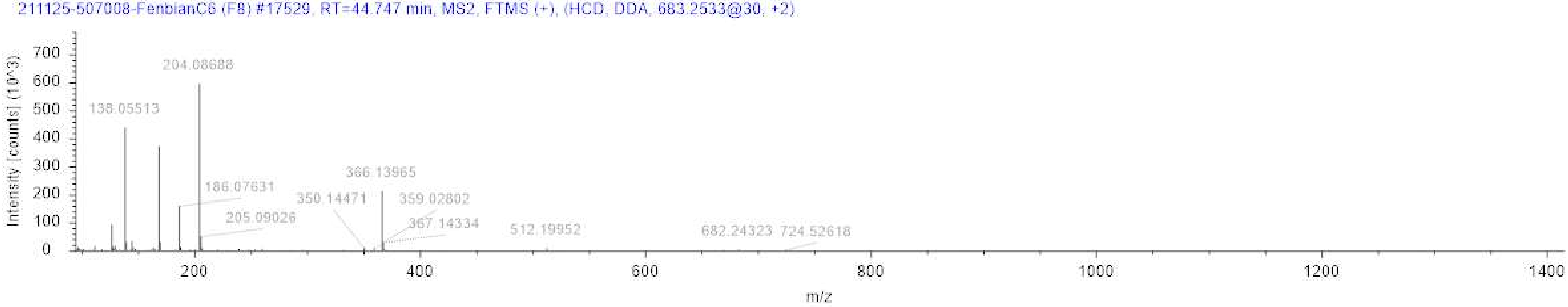

© Reported with Compound Discoverer 3.3

Compounds

Structure	Name	RT [min]	Formula	Calc. MW	Areas
n/a		43.95	n/a	1364.49415	1.33e9 3.63e9 1.21e9 4.56e8 6.48e9 8.44e9

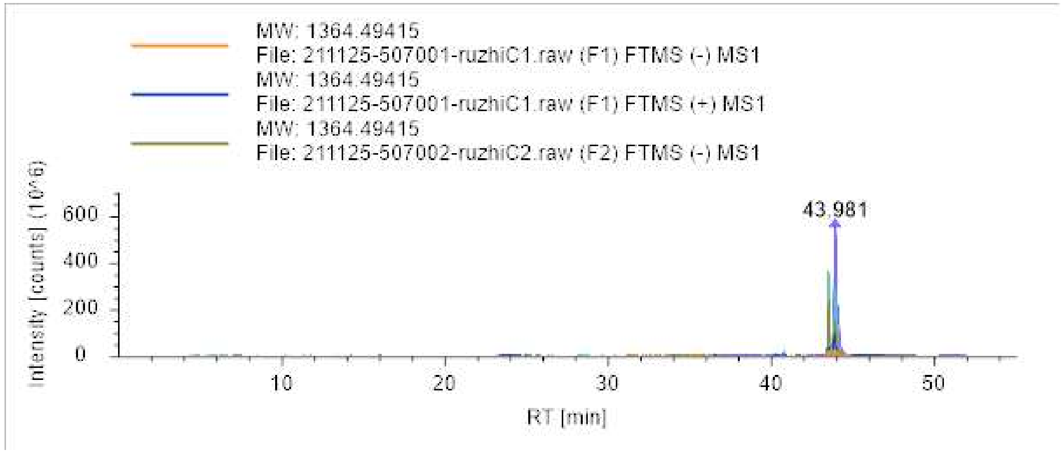

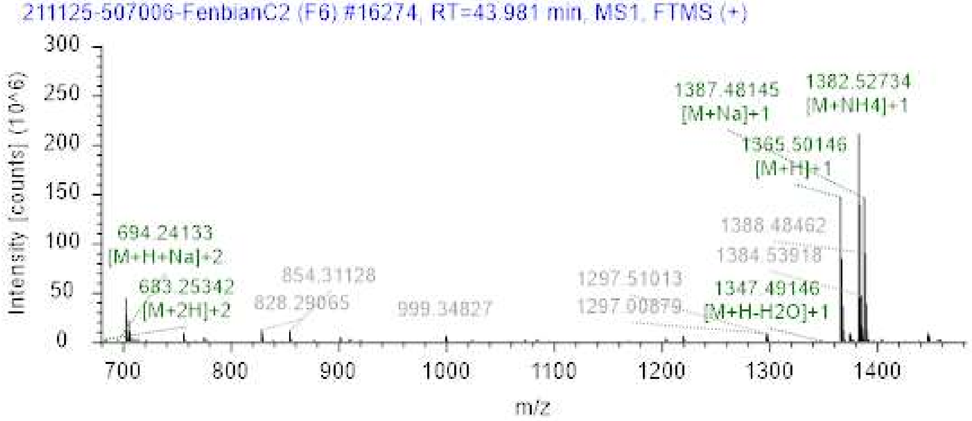

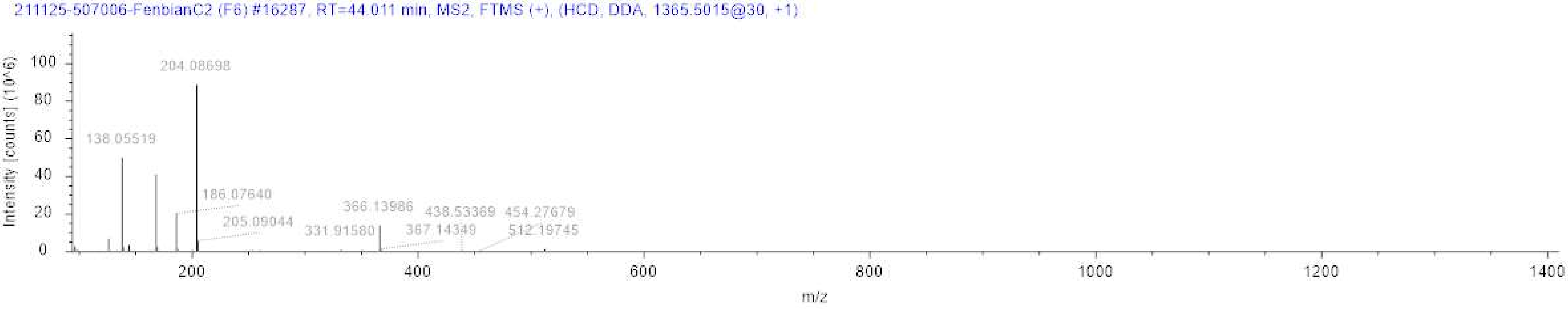

© Reported with Compound Discoverer 3.3

Structure	Name	RT [min]	Formula	Calc. MW	Areas
n/a		43.58	n/a	1364.49542	3.76e8 1.35e7 3.64e8 4.09e9

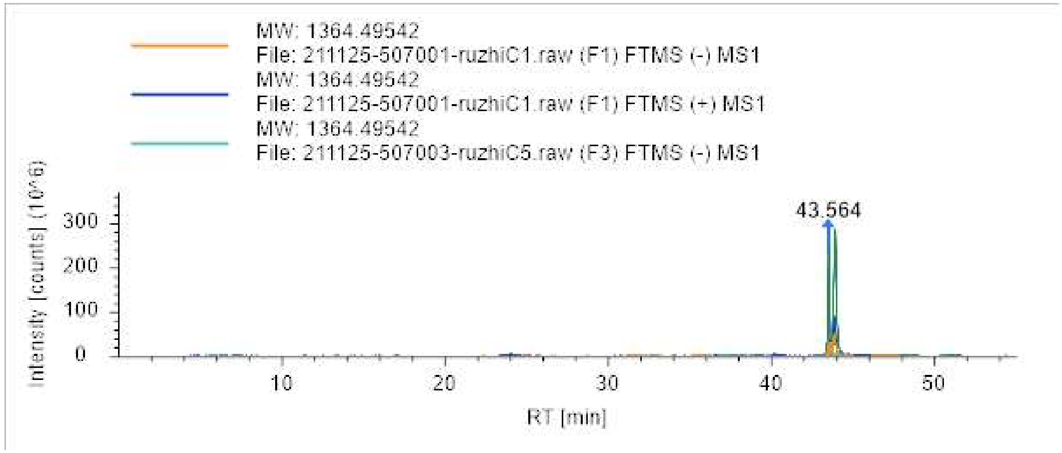

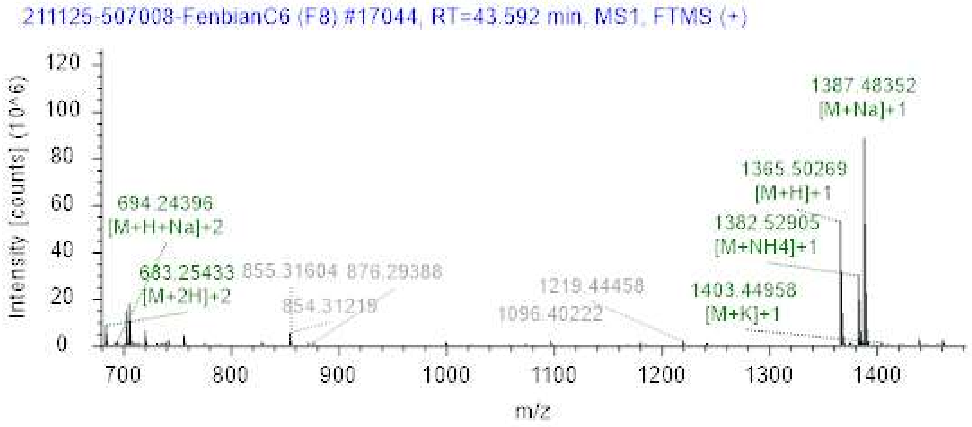

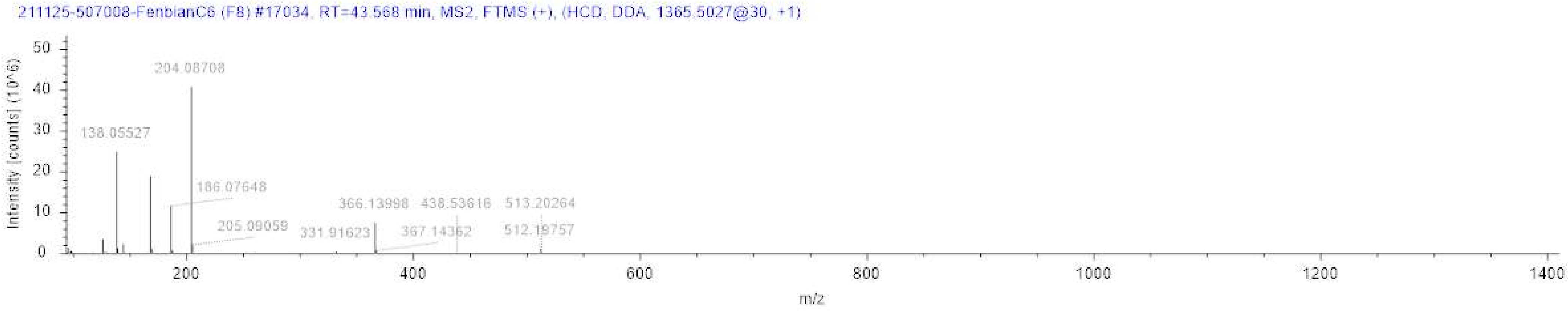

© Reported with Compound Discoverer 3.3

Compounds

Structure	Name	RT [min]	Formula	Calc. MW	Areas
n/a		44.73	n/a	1365.49870	8.86e6

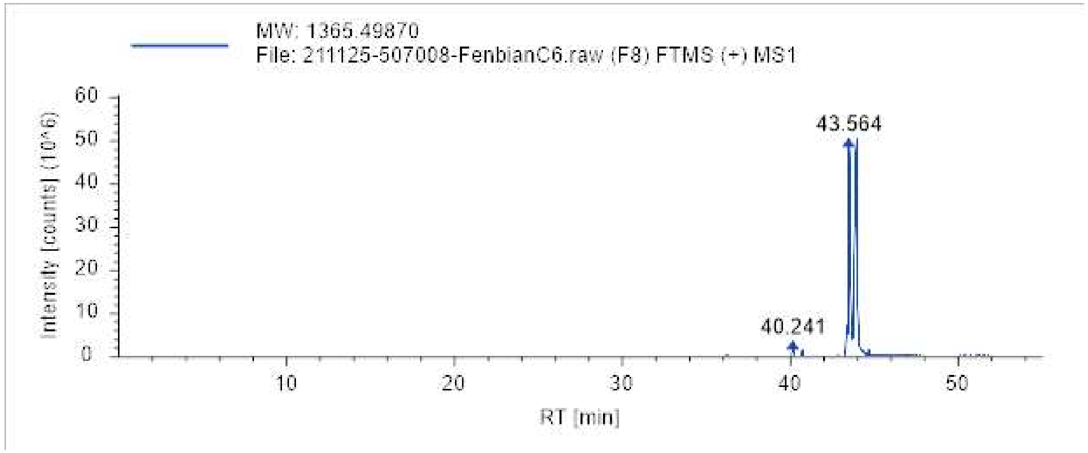

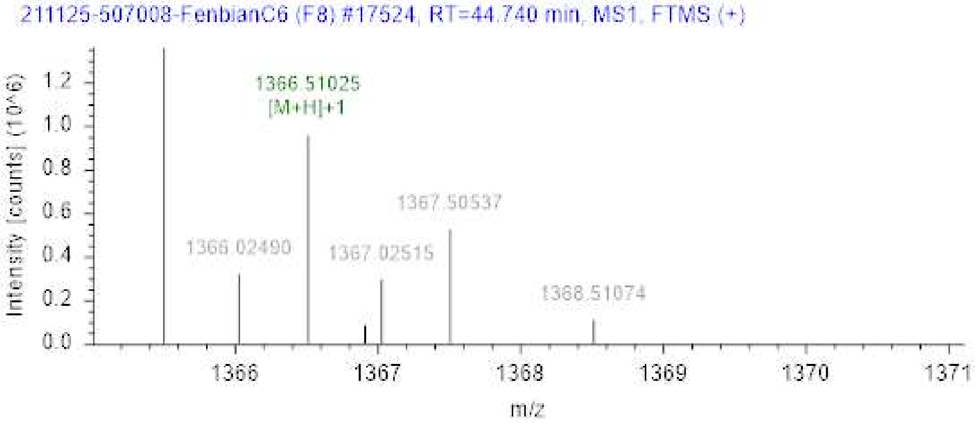

© Reported with Compound Discoverer 3.3

Compounds

Structure	Name	RT [min]	Formula	Calc. MW	Areas
n/a		44.07	n/a	1380.49403	4.61e7 5.07e7

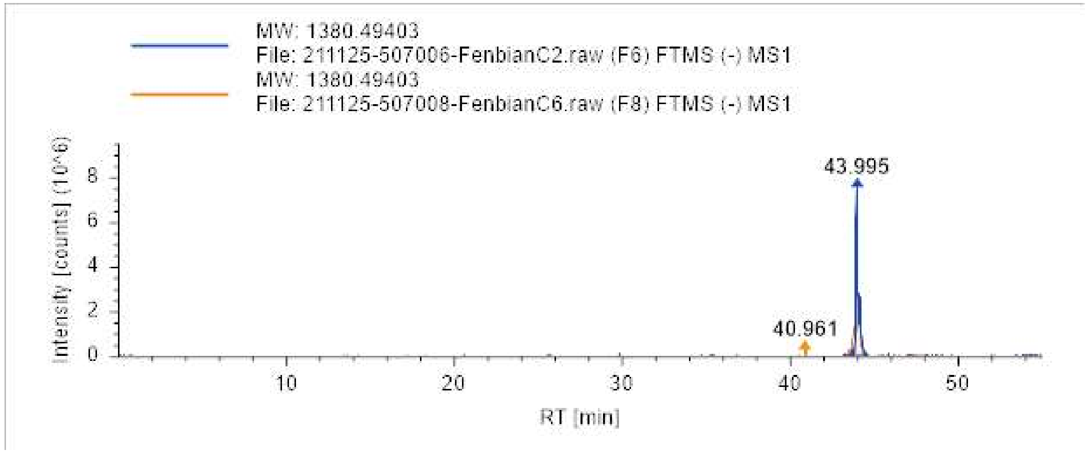

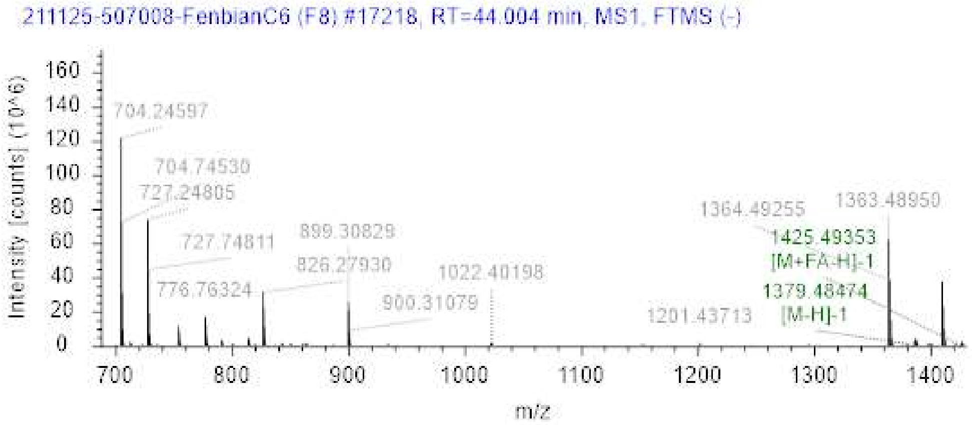

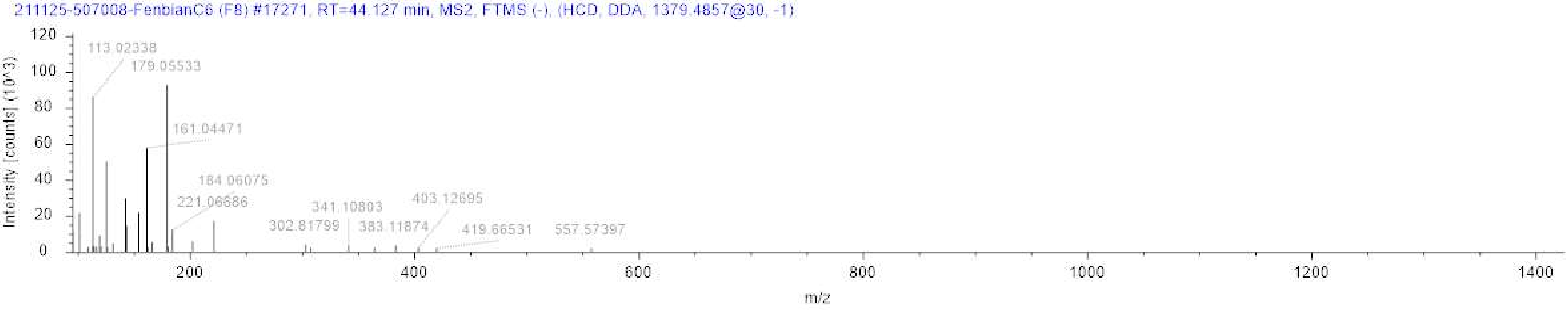

© Reported with Compound Discoverer 3.3

Compounds

Structure	Name	RT [min]	Formula	Calc. MW	Areas
n/a		20.14	n/a	1389.52524	1.26e7

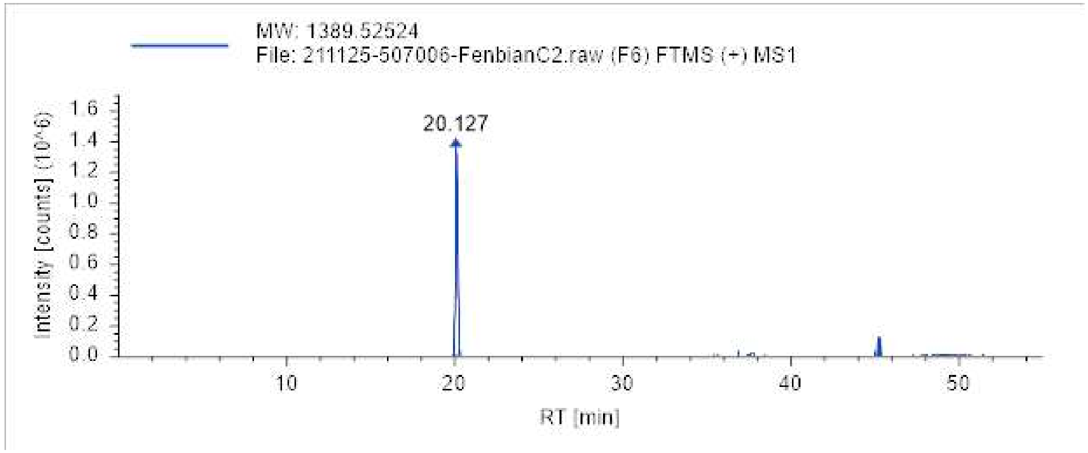

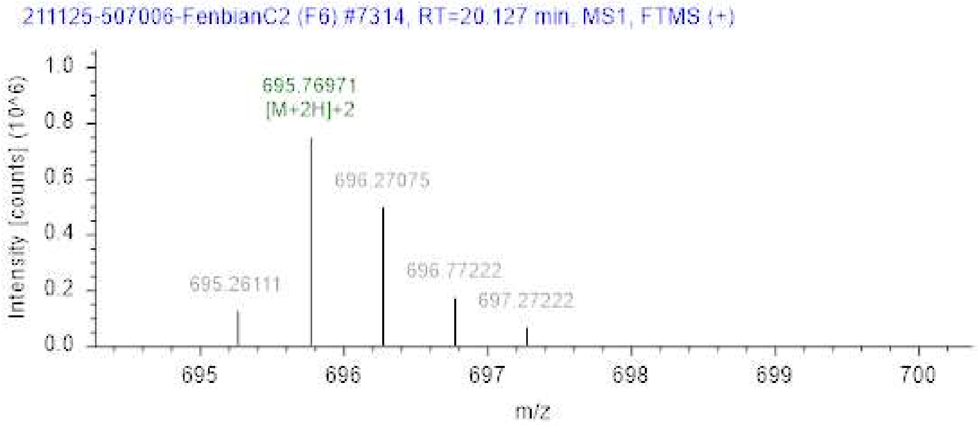

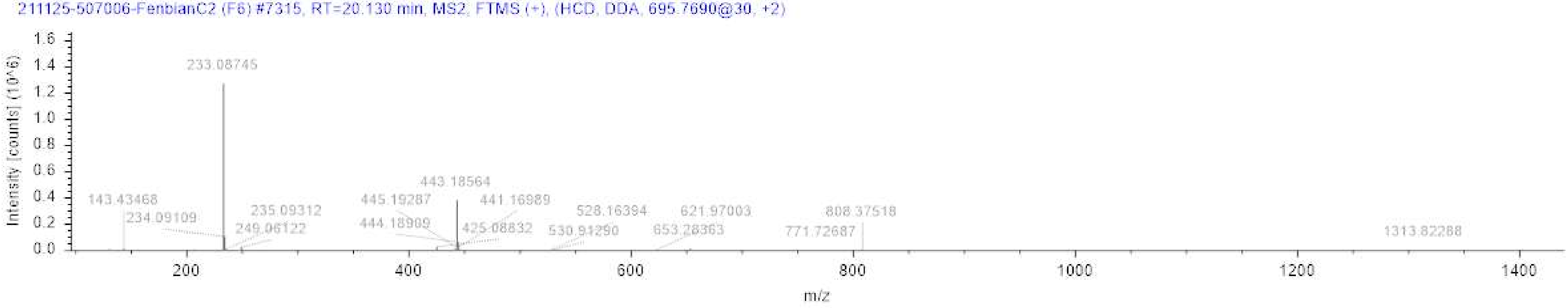

© Reported with Compound Discoverer 3.3

Structure	Name	RT [min]	Formula	Calc. MW	Areas
n/a		43.48	n/a	1405.52117	4.91e7

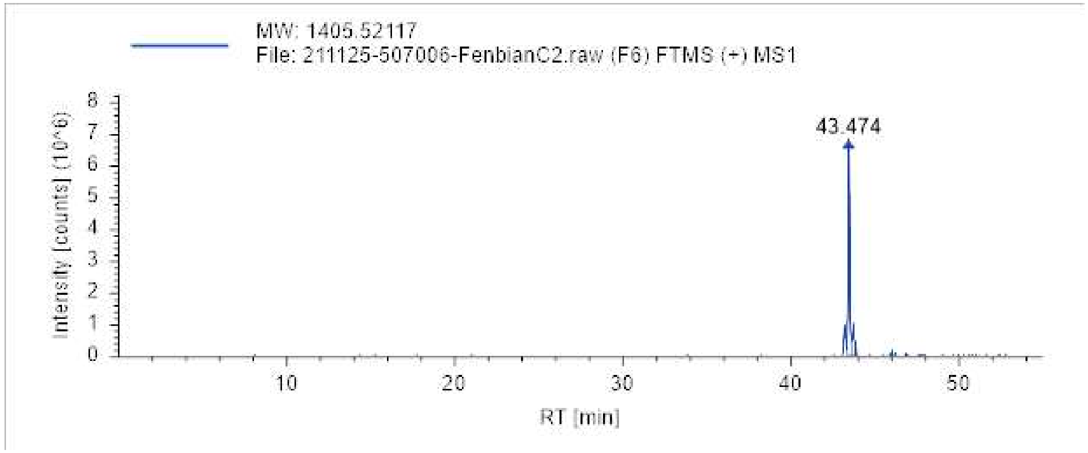

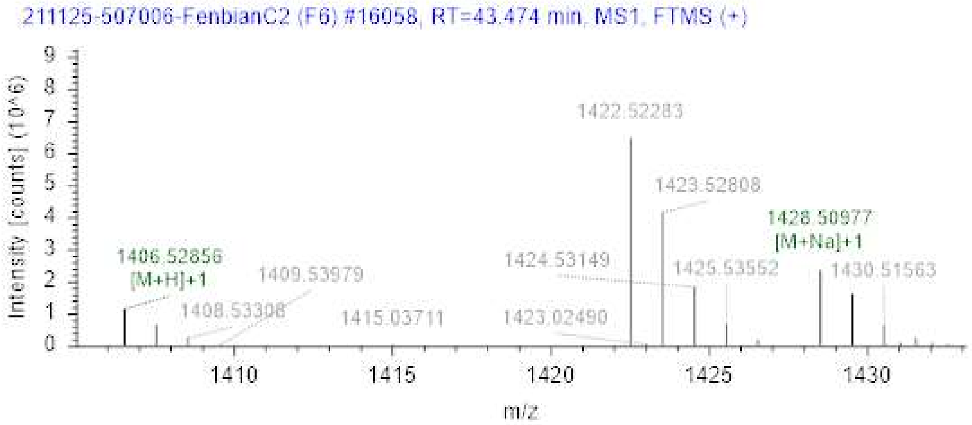

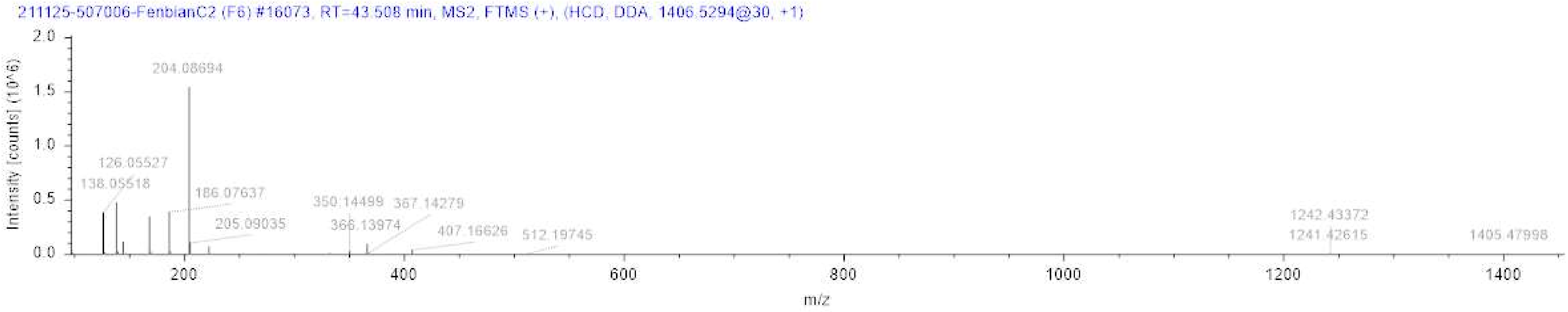

© Reported with Compound Discoverer 3.3

Compounds

Structure	Name	RT [min]	Formula	Calc. MW	Areas
n/a		44.46	n/a	1405.52327	9.93e6

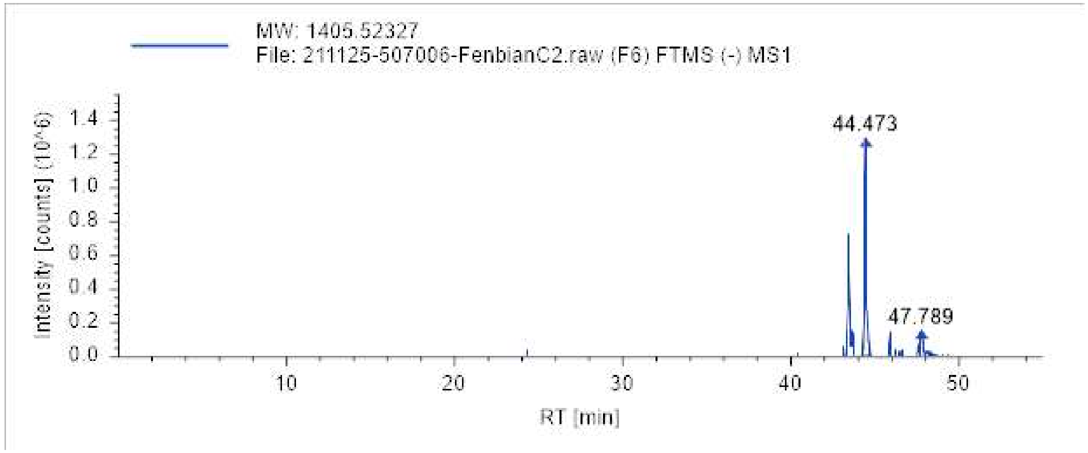

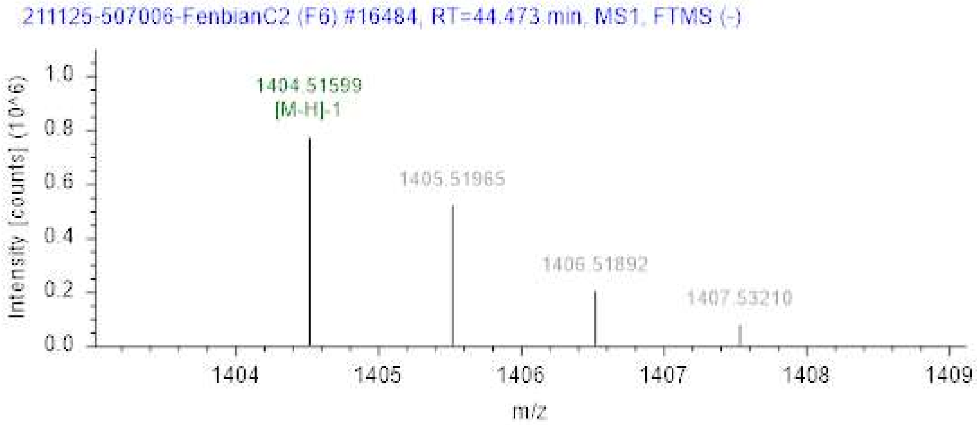

© Reported with Compound Discoverer 3.3

Compounds

Structure	Name	RT [min]	Formula	Calc. MW	Areas
n/a		45.52	n/a	1421.51255	2.64e7

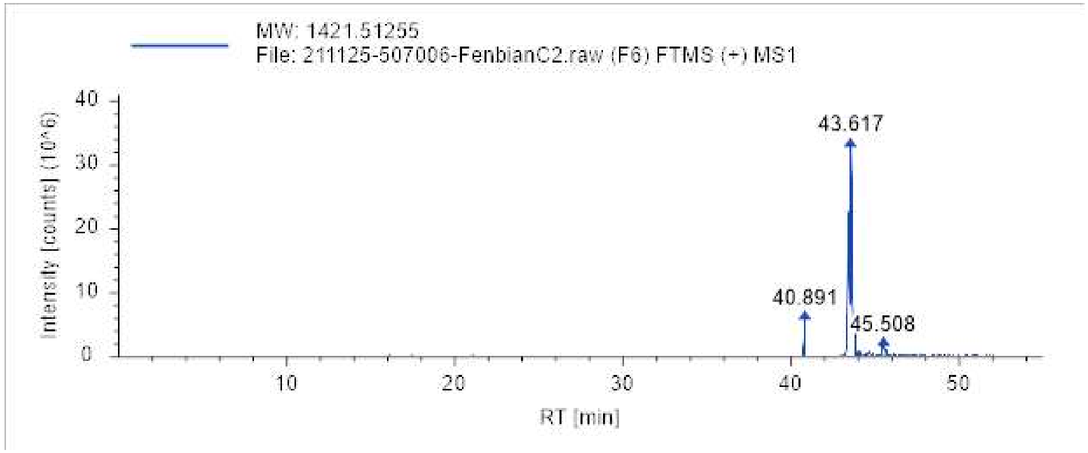

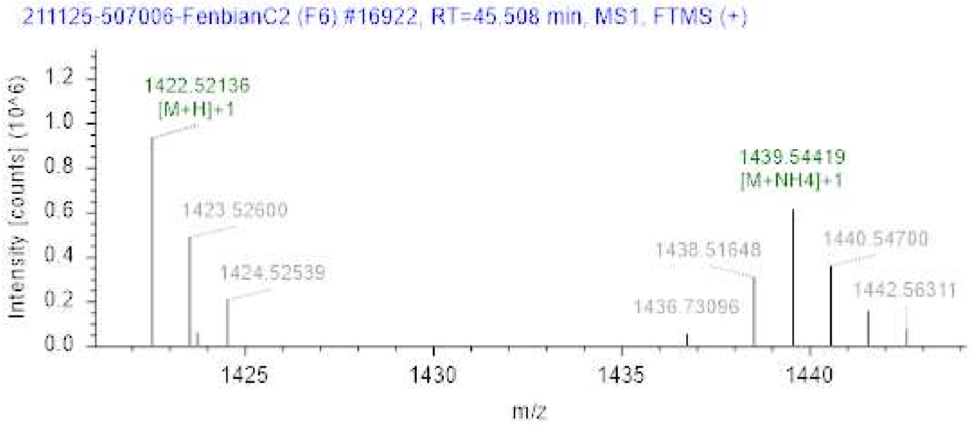

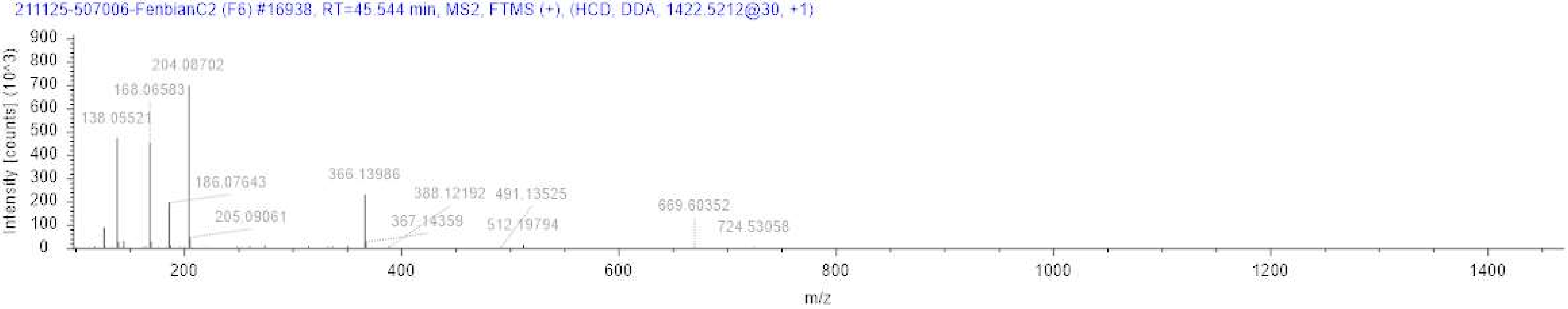

© Reported with Compound Discoverer 3.3

Structure	Name	RT [min]	Formula	Calc. MW	Areas
n/a		43.45	n/a	1421.51744	3.31e8

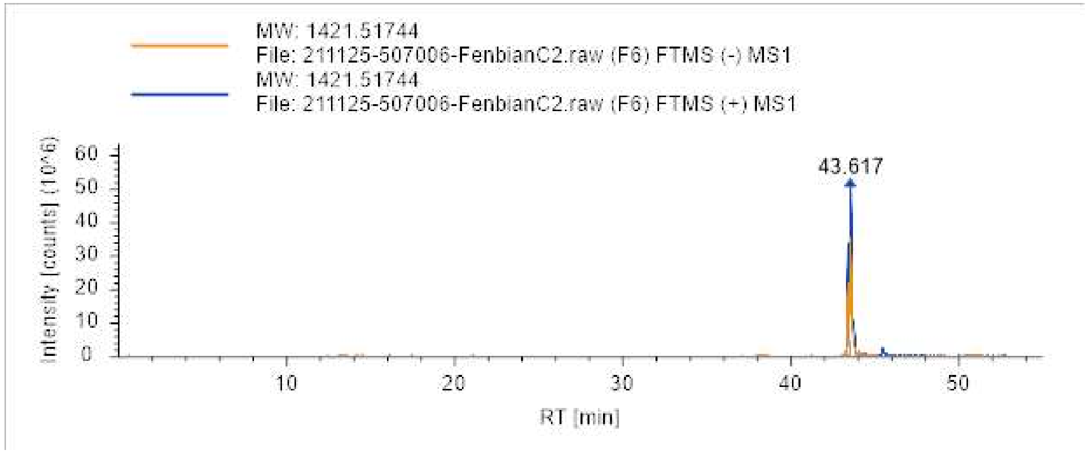

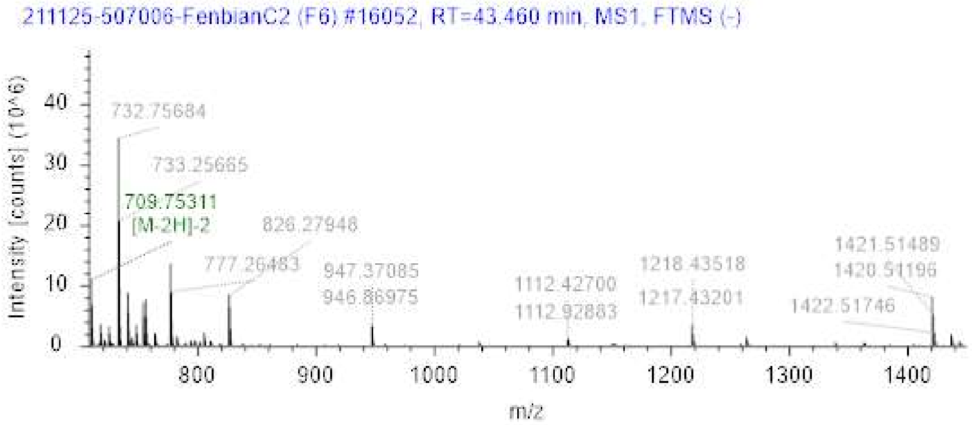

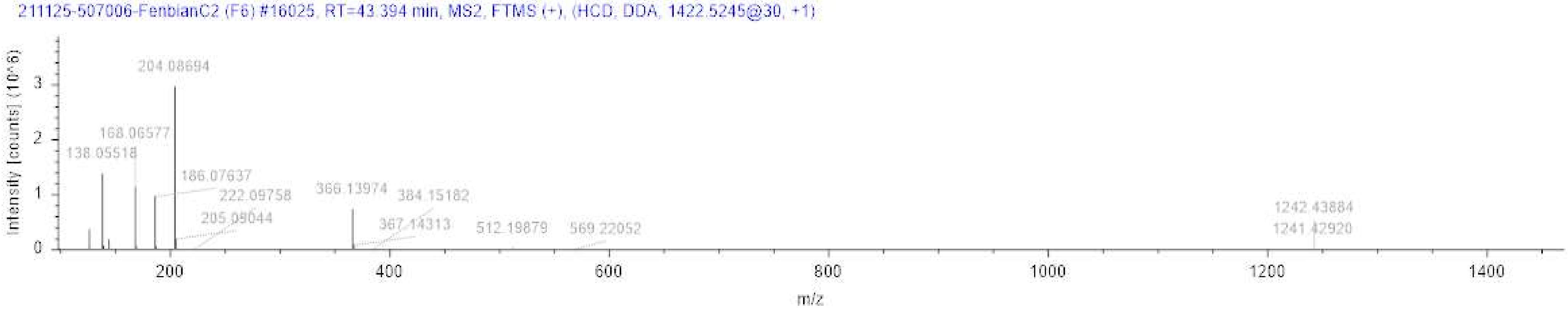

© Reported with Compound Discoverer 3.3

Structure	Name	RT [min]	Formula	Calc. MW	Areas
n/a		43.96	n/a	1428.49641	3.48e7

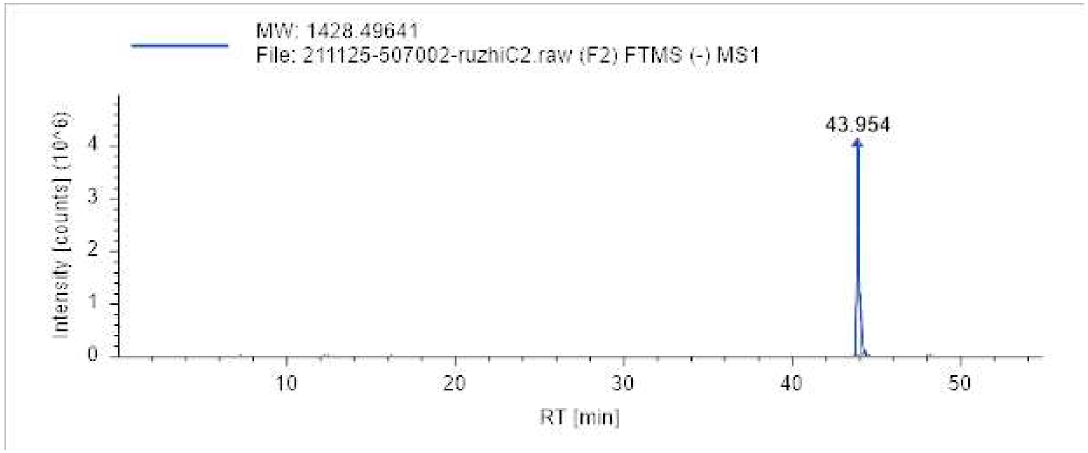

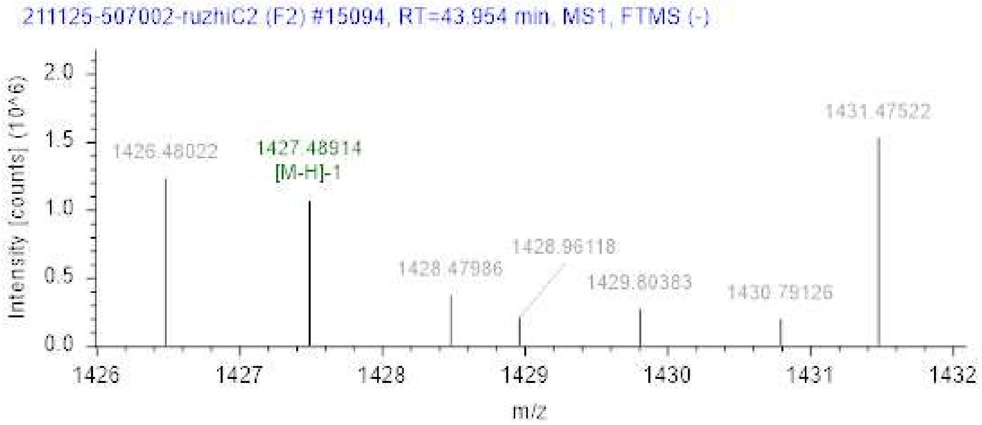

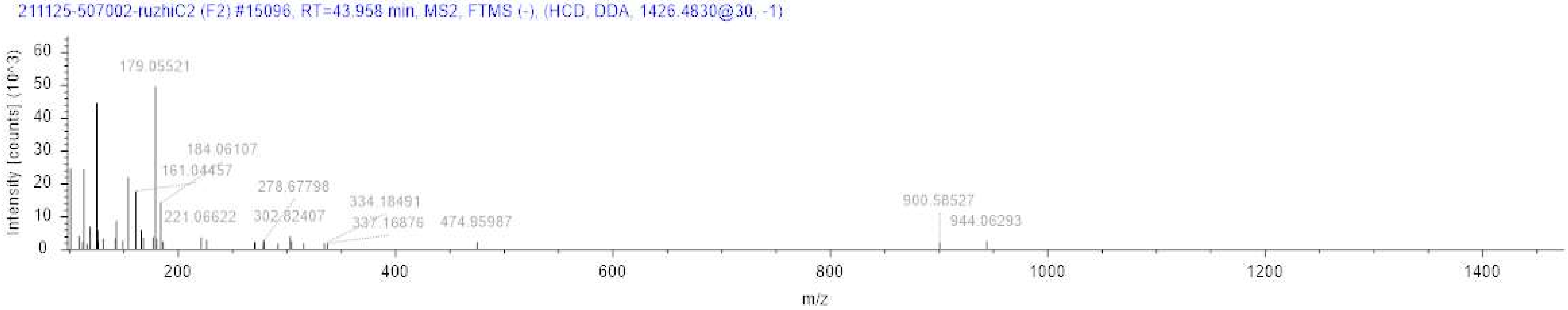

© Reported with Compound Discoverer 3.3

Structure	Name	RT [min]	Formula	Calc. MW	Areas
n/a		42.91	n/a	1435.49853	1.66e7 1.28e8 5.37e7 1.80e7 9.23e8 1.37e8

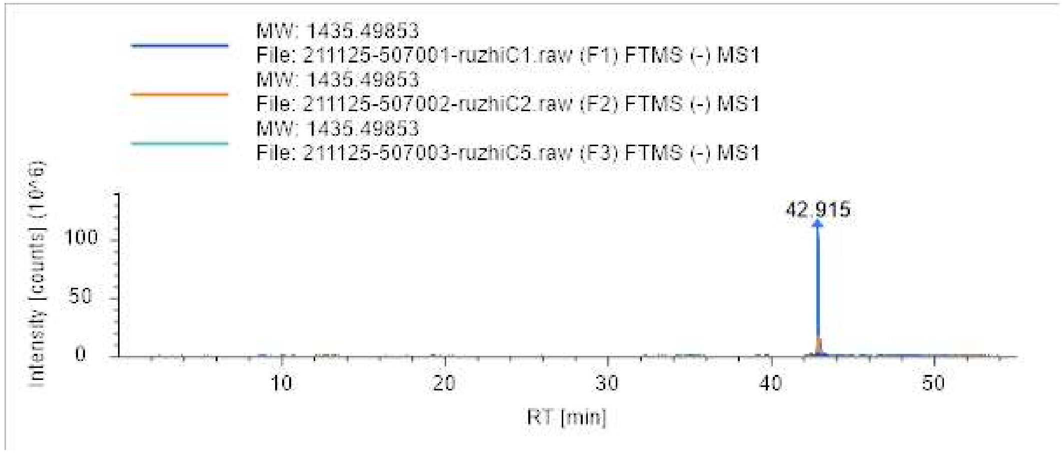

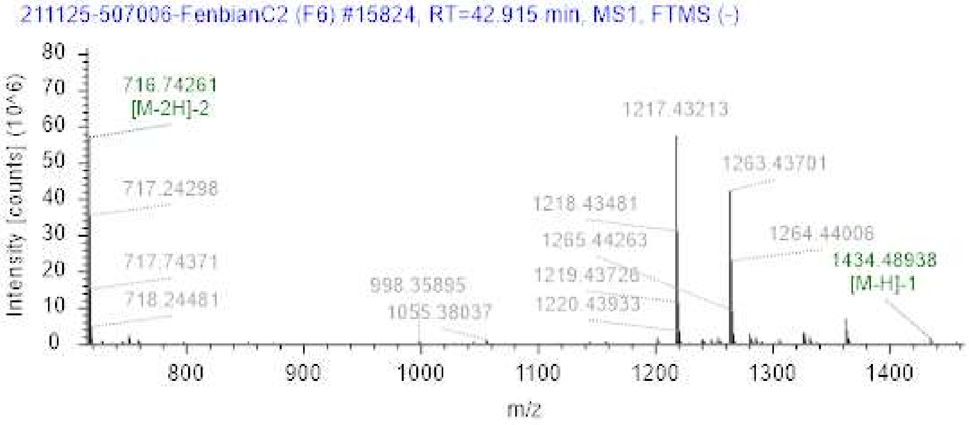

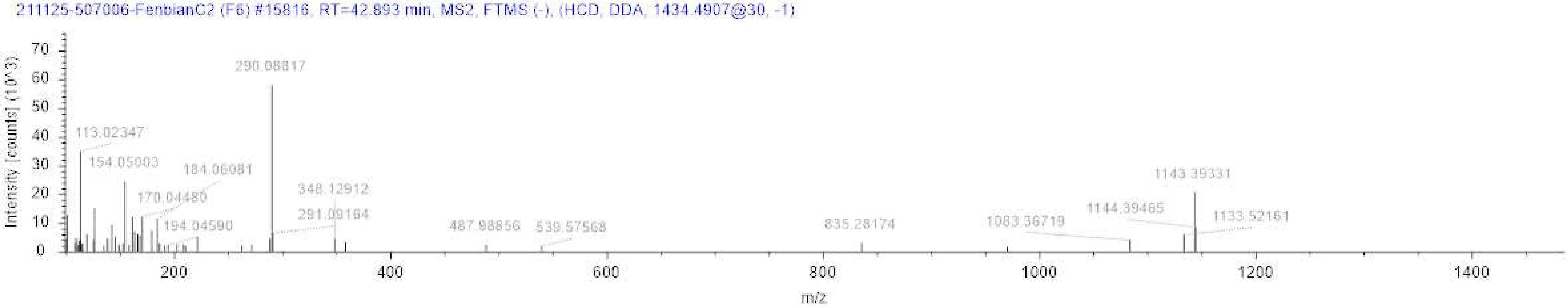

© Reported with Compound Discoverer 3.3

Structure	Name	RT [min]	Formula	Calc. MW	Areas
n/a		45.58	n/a	1437.50835	7.46e6

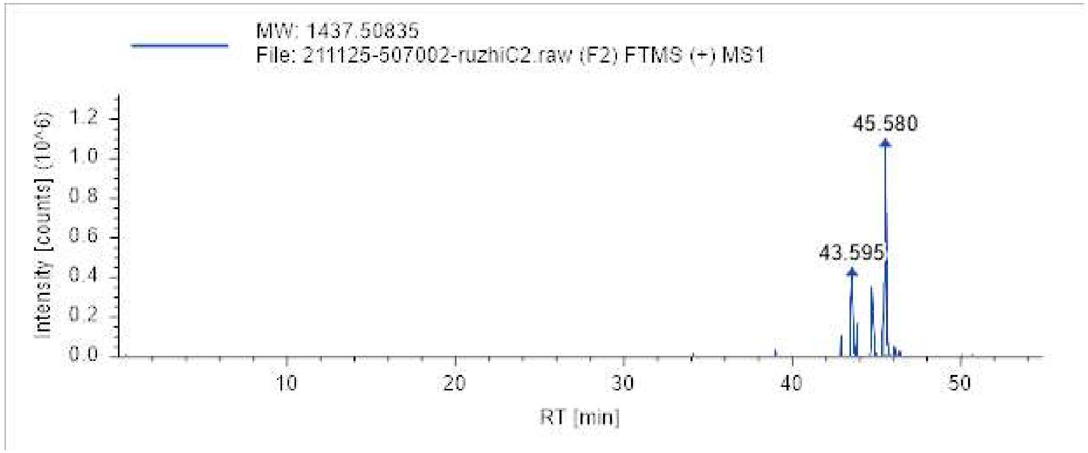

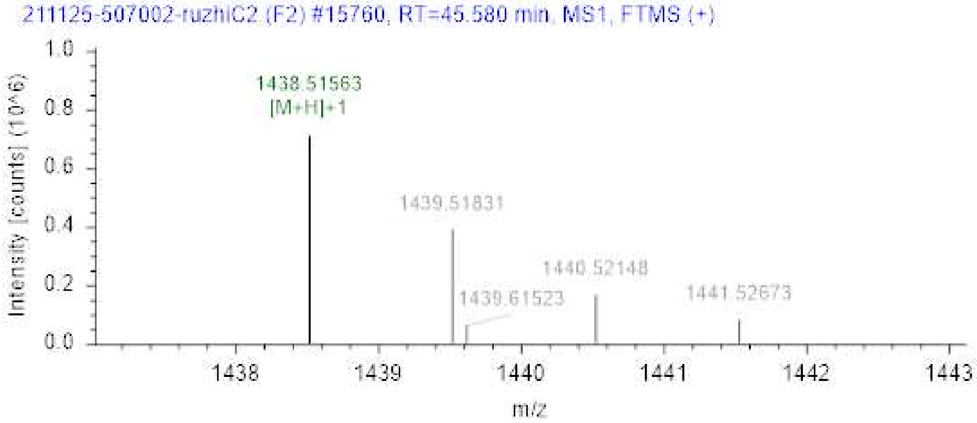

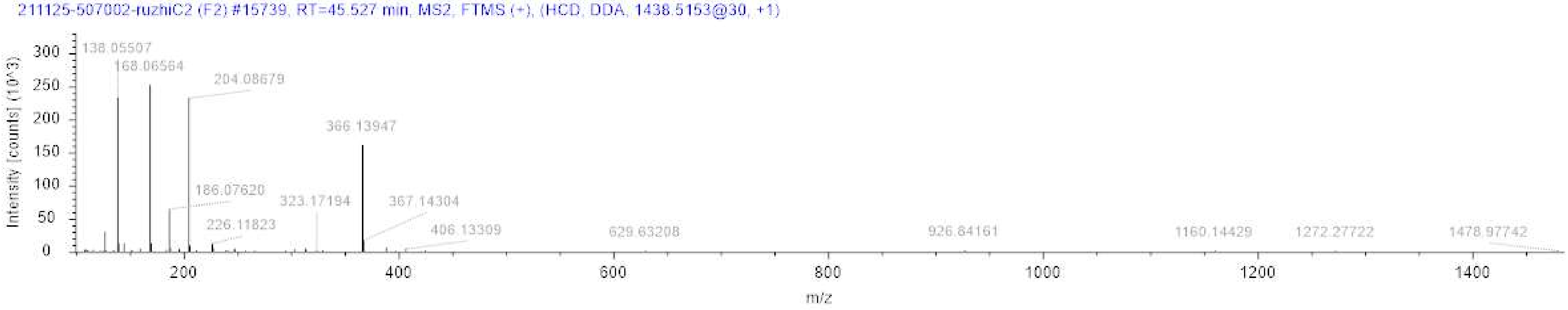

© Reported with Compound Discoverer 3.3

Compounds

Structure	Name	RT [min]	Formula	Calc. MW	Areas
n/a		44.43	n/a	1437.51010	1.69e7

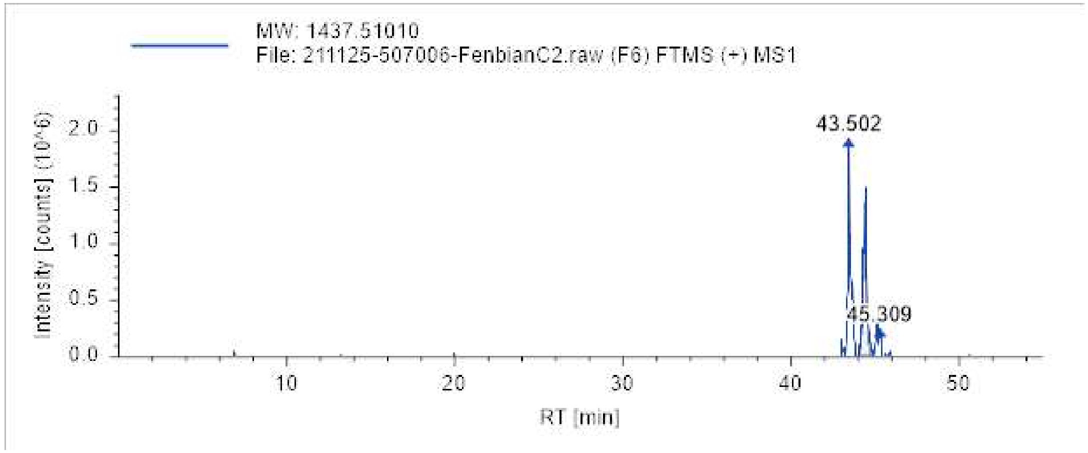

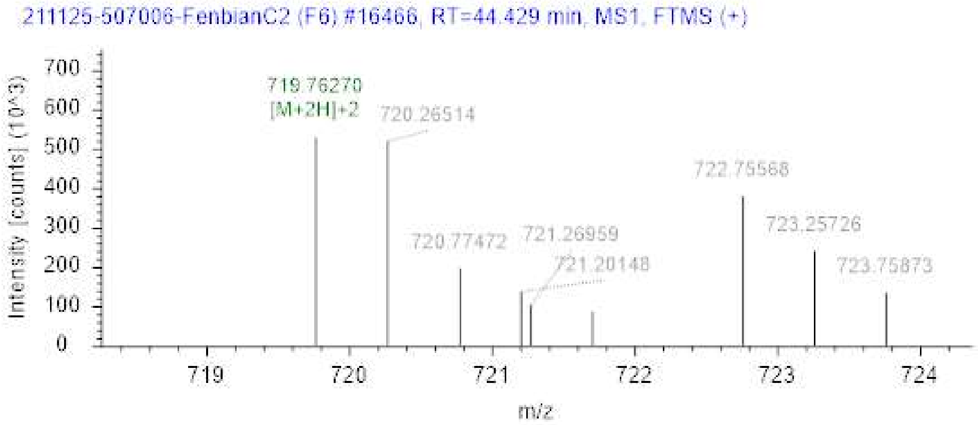

© Reported with Compound Discoverer 3.3

Compounds

Structure	Name	RT [min]	Formula	Calc. MW	Areas
n/a		43.57	n/a	1437.51257	4.02e7 3.74e7 1.13e7 5.91e7 1.73e8 3.95e8

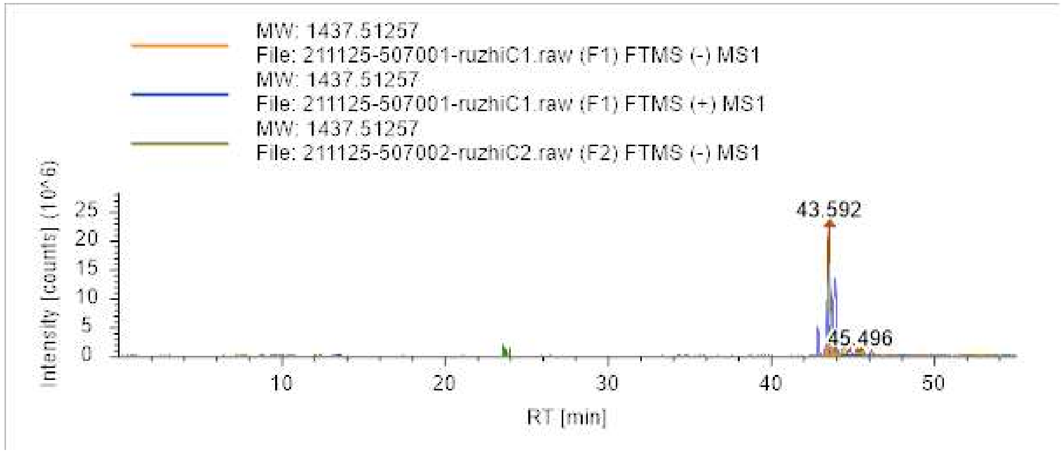

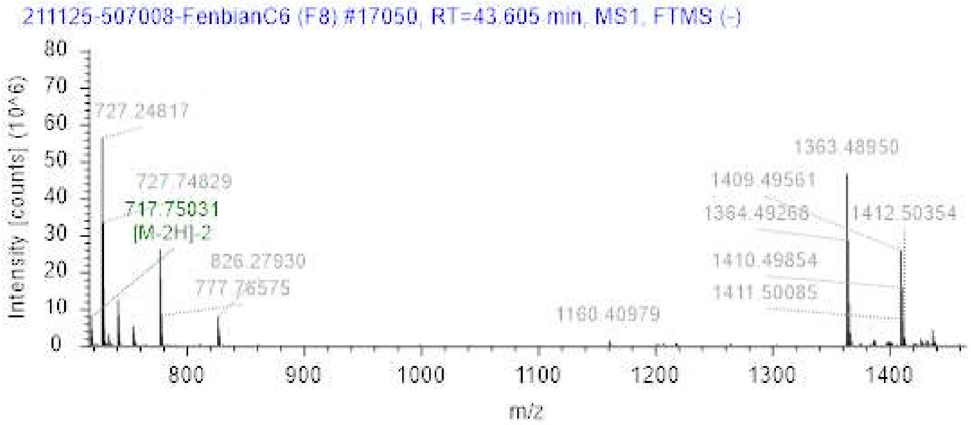

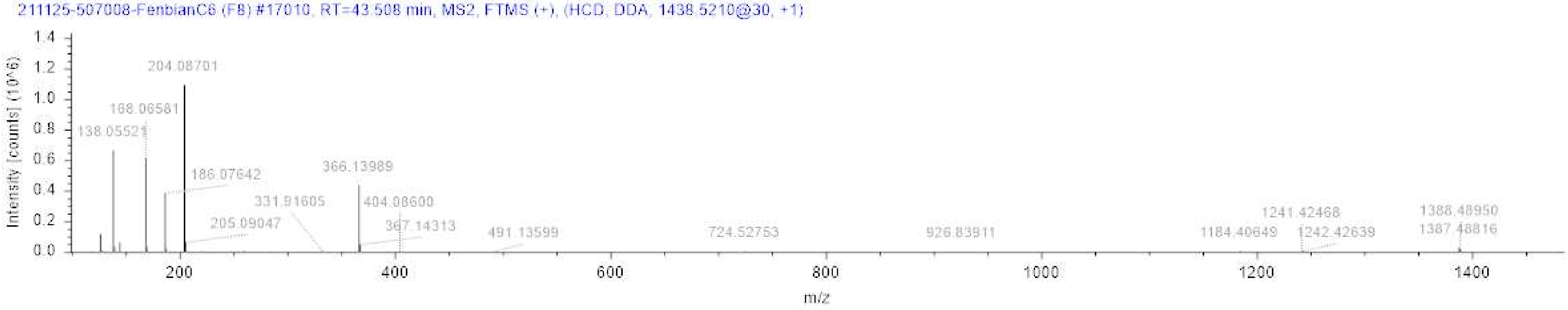

© Reported with Compound Discoverer 3.3

Compounds

Structure	Name	RT [min]	Formula	Calc. MW	Areas
n/a		43.25	n/a	1451.49198	3.52e7 7.80e6

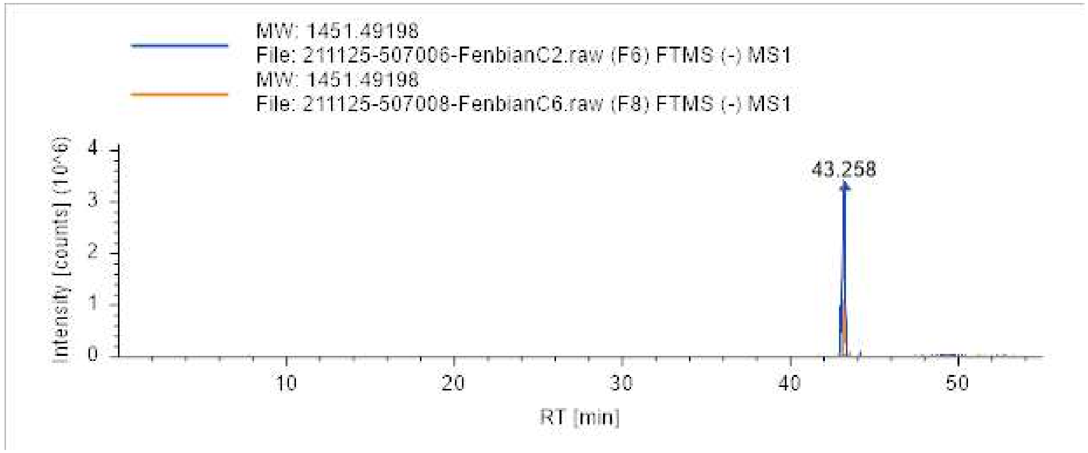

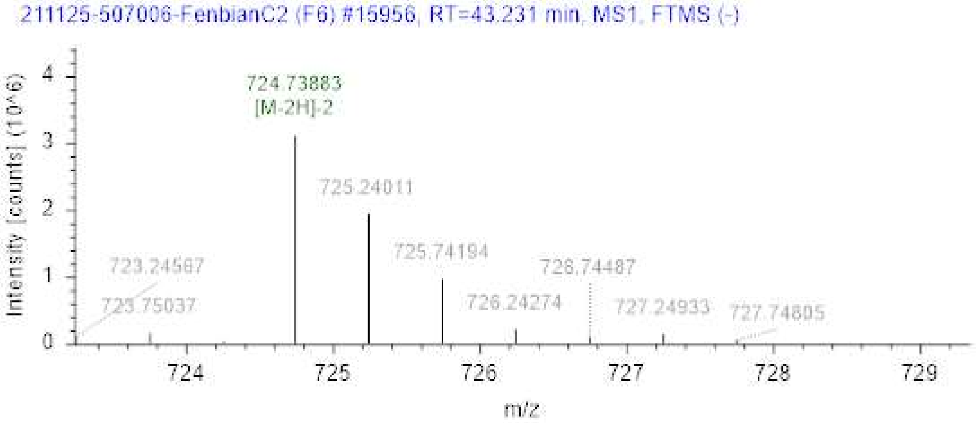

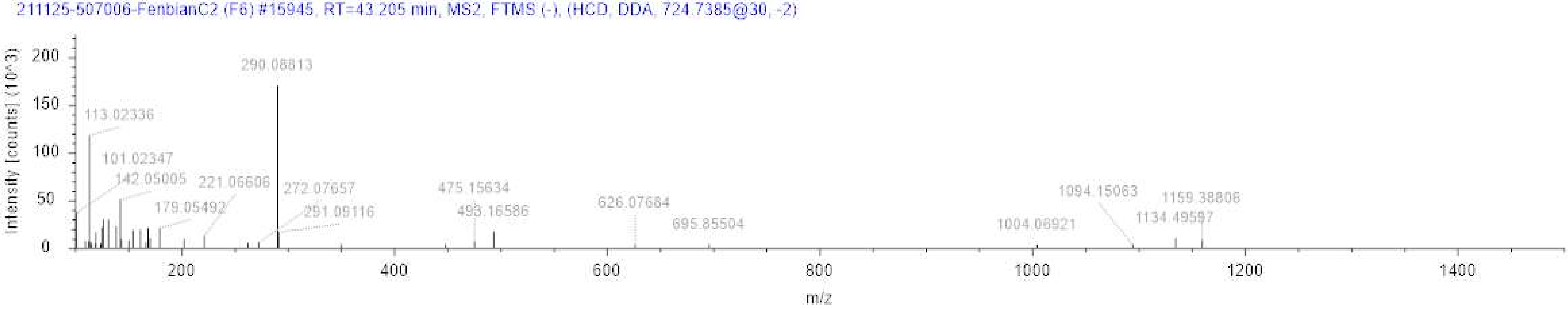

© Reported with Compound Discoverer 3.3

Compounds

Structure	Name	RT [min]	Formula	Calc. MW	Areas
n/a		44.73	n/a	1478.53115	8.99e6

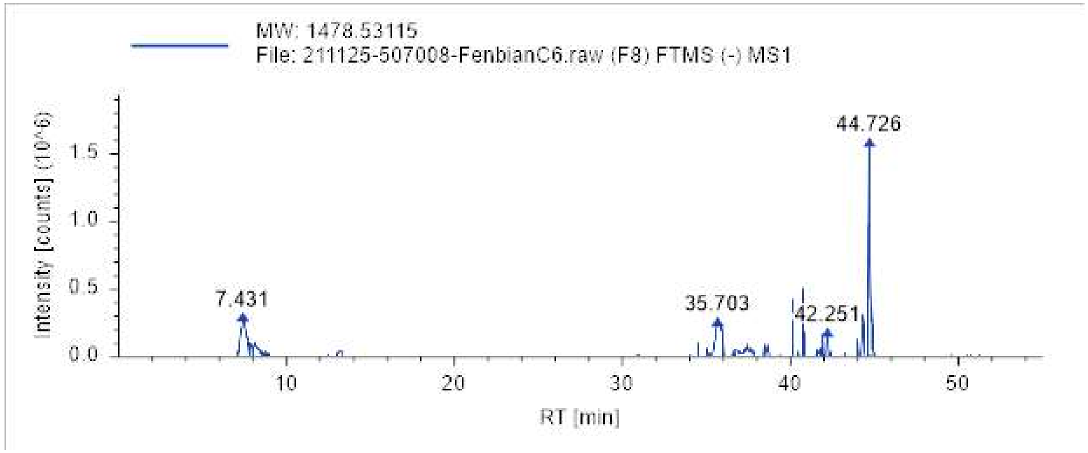

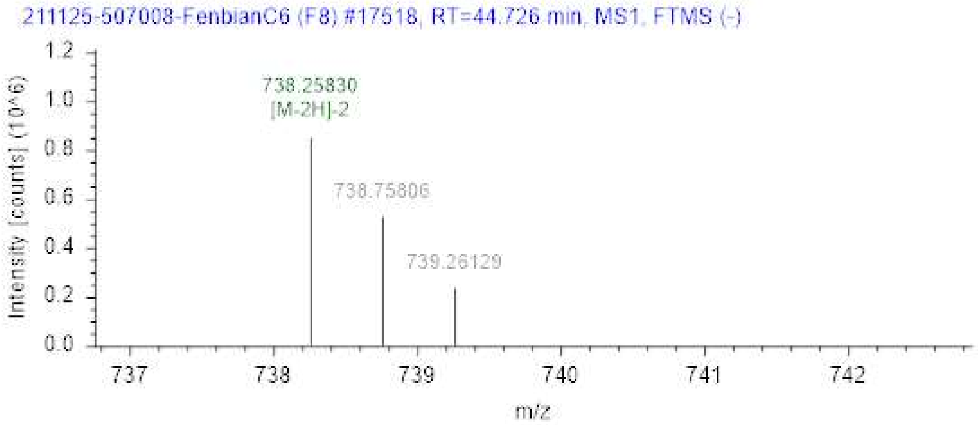

© Reported with Compound Discoverer 3.3

Compounds

Structure	Name	RT [min]	Formula	Calc. MW	Areas
n/a		43.15	n/a	1478.53904	2.40e7

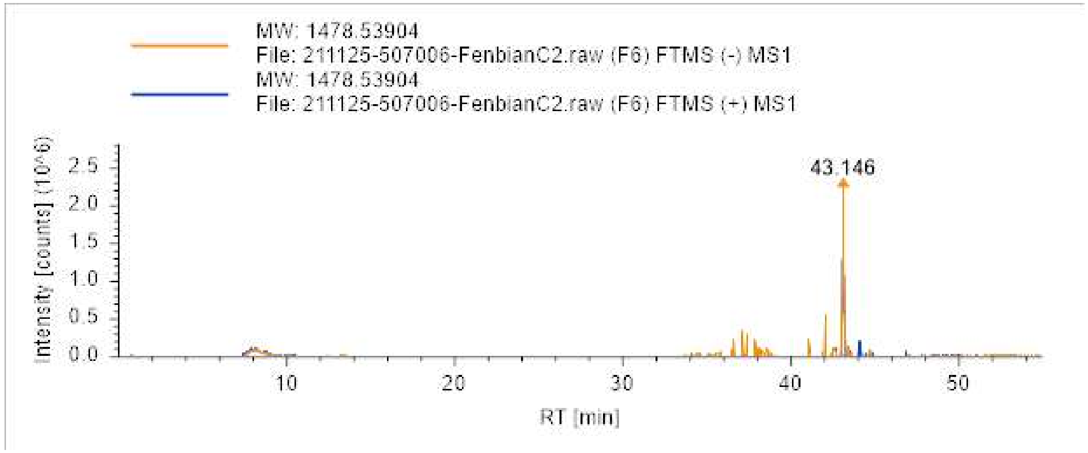

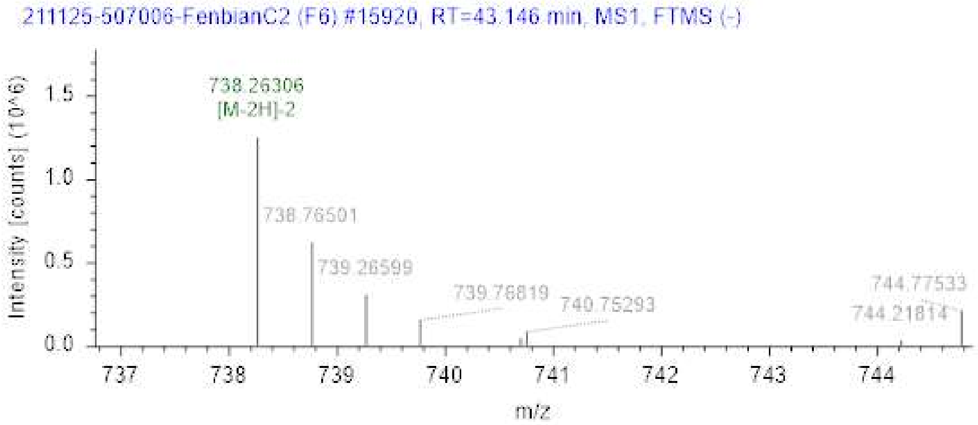

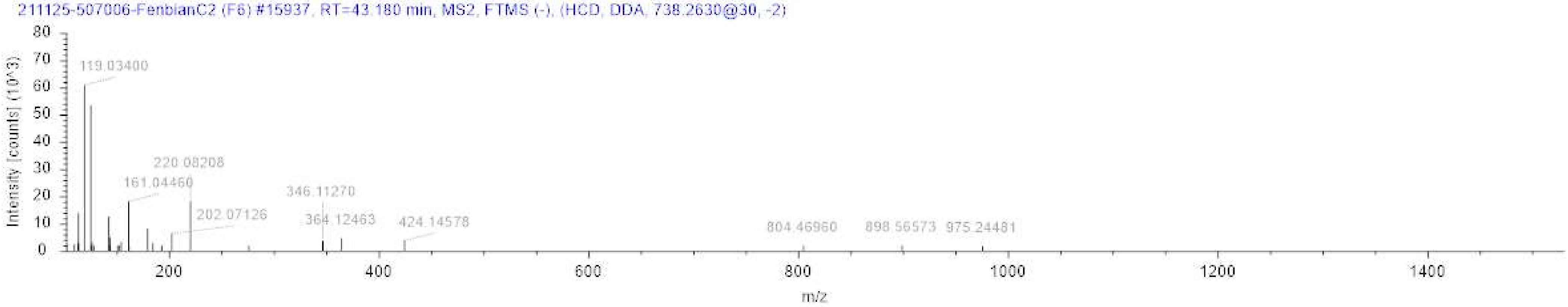

© Reported with Compound Discoverer 3.3

Compounds

Structure	Name	RT [min]	Formula	Calc. MW	Areas
n/a		38.30	n/a	1483.48714	9.30e6

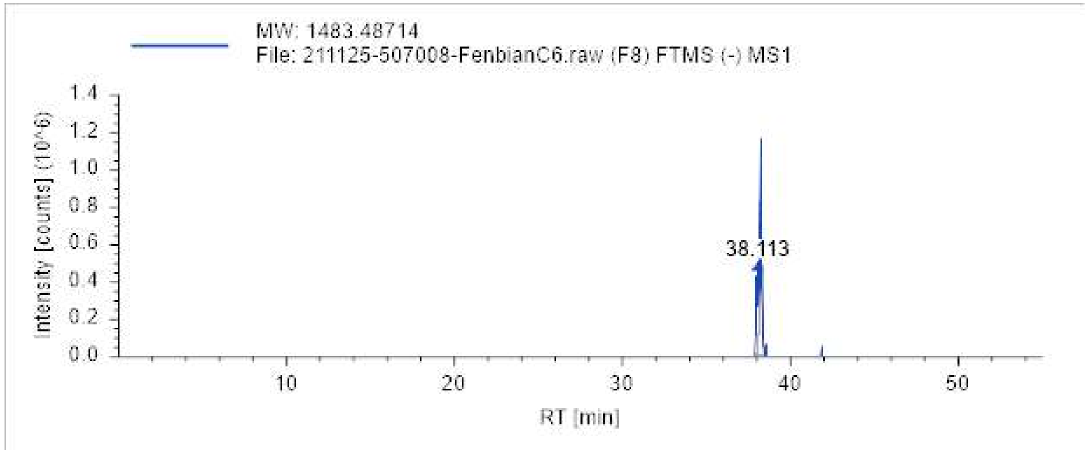

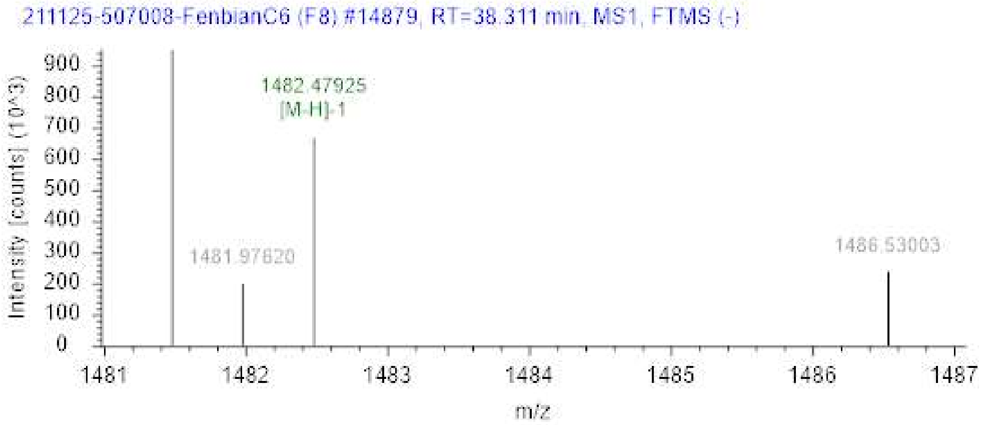

© Reported with Compound Discoverer 3.3

Structure	Name	RT [min]	Formula	Calc. MW	Areas
n/a		43.85	n/a	1492.51865	7.32e6

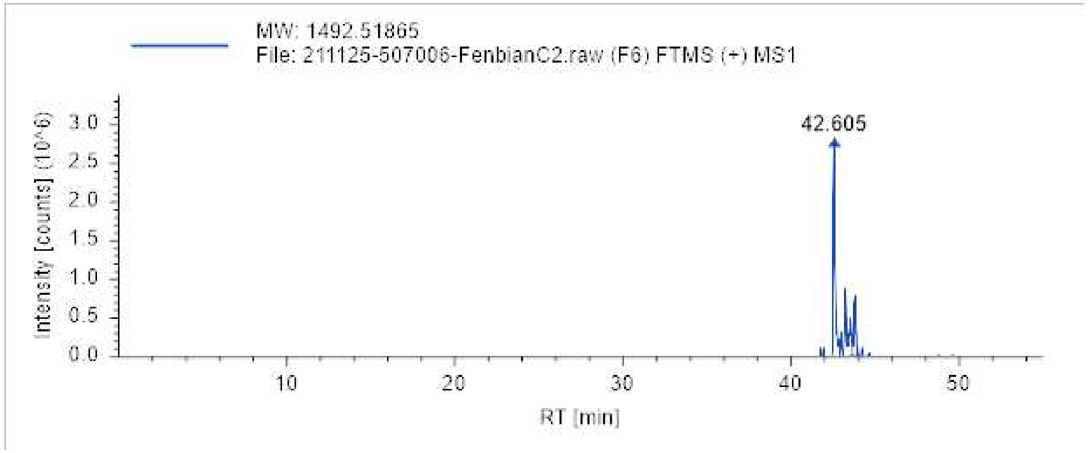

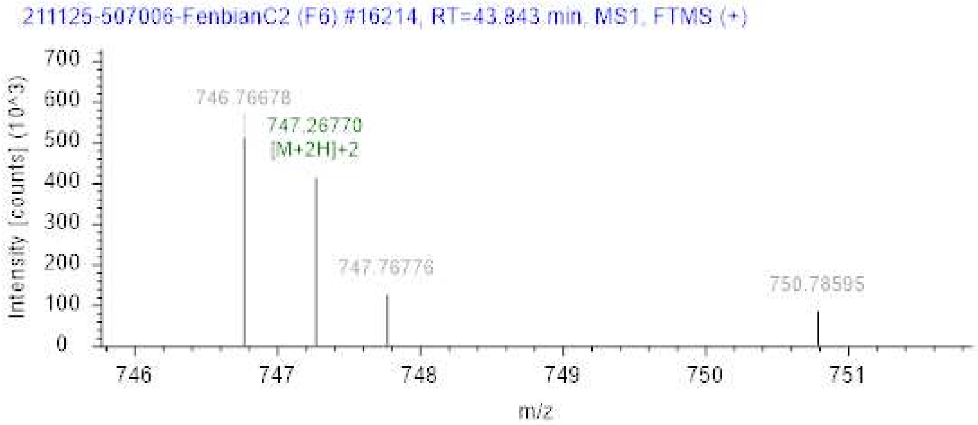

© Reported with Compound Discoverer 3.3

Compounds

Structure	Name	RT [min]	Formula	Calc. MW	Areas
n/a		42.60	n/a	1492.52311	2.88e8; 6.35e7

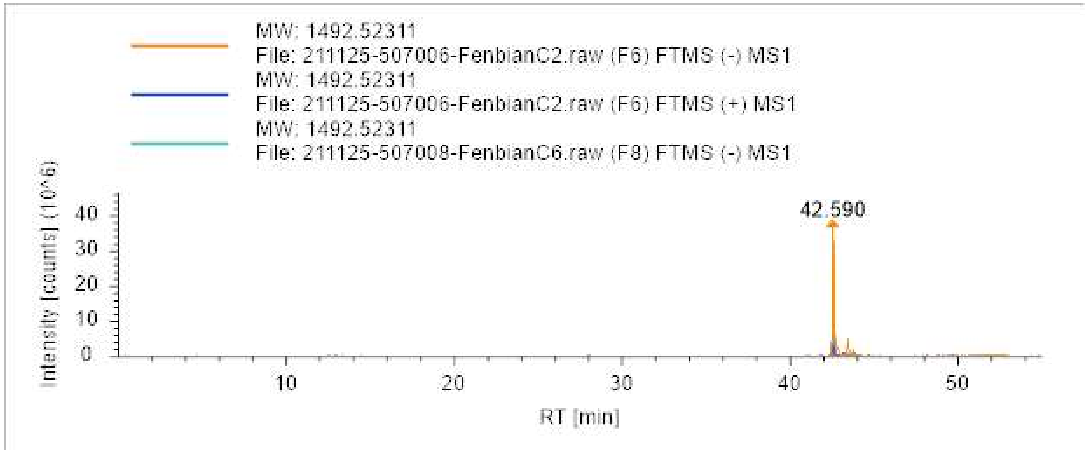

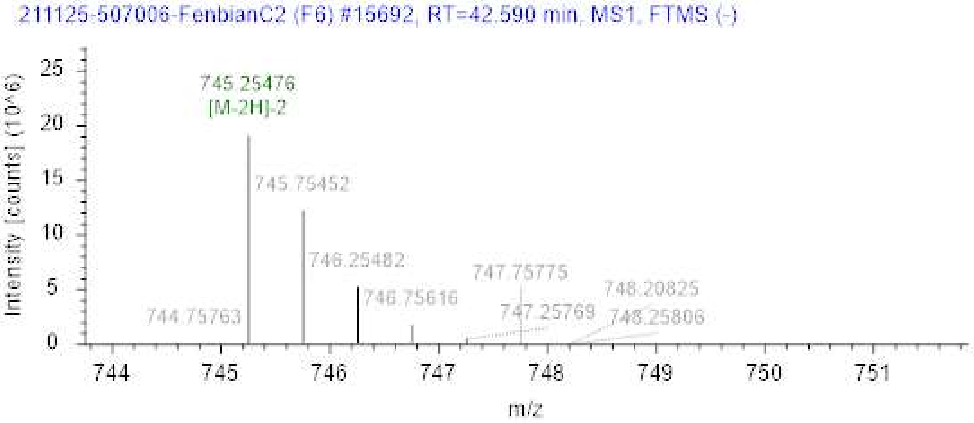

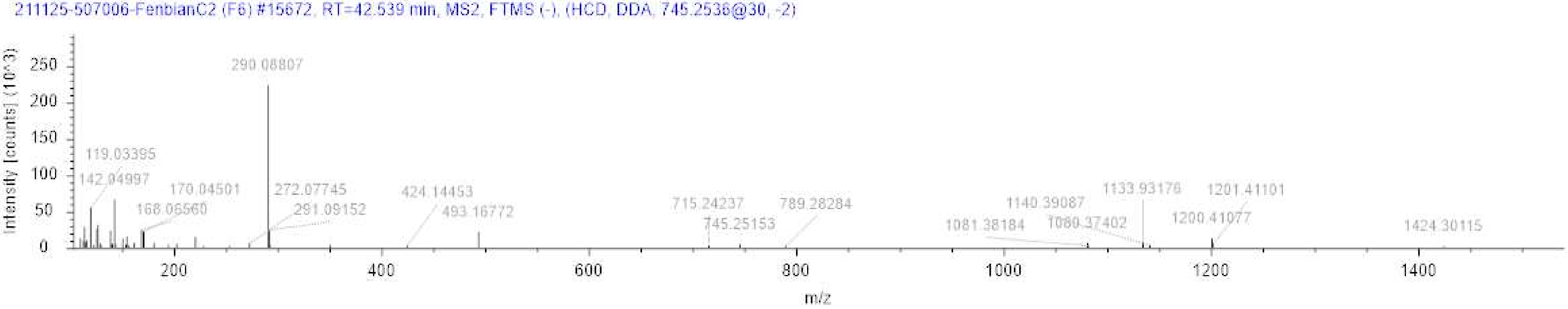

© Reported with Compound Discoverer 3.3

Structure	Name	RT [min]	Formula	Calc. MW	Areas
n/a		44.73	n/a	1496.54102	6.23e6 6.56e7

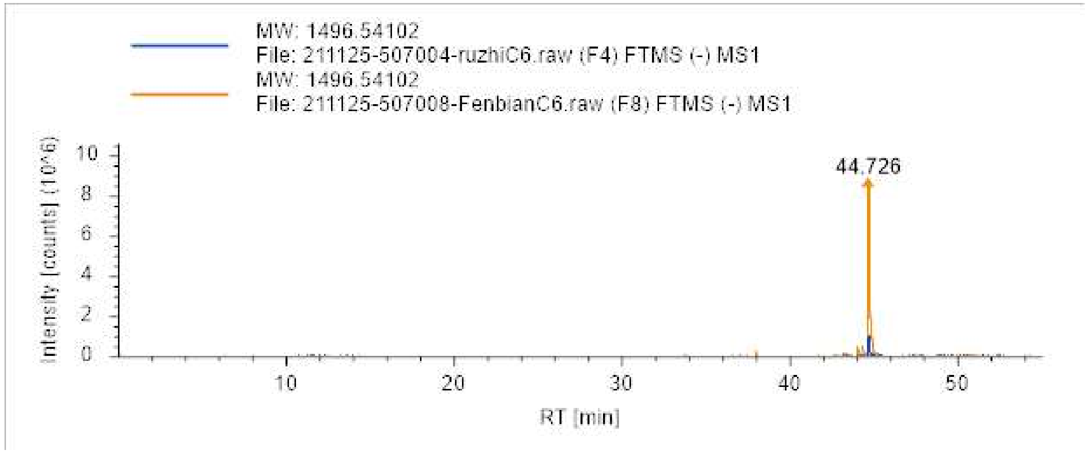

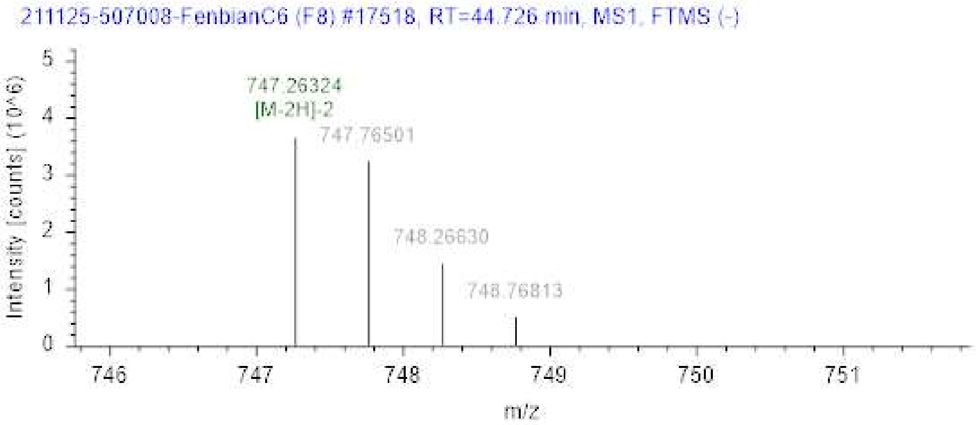

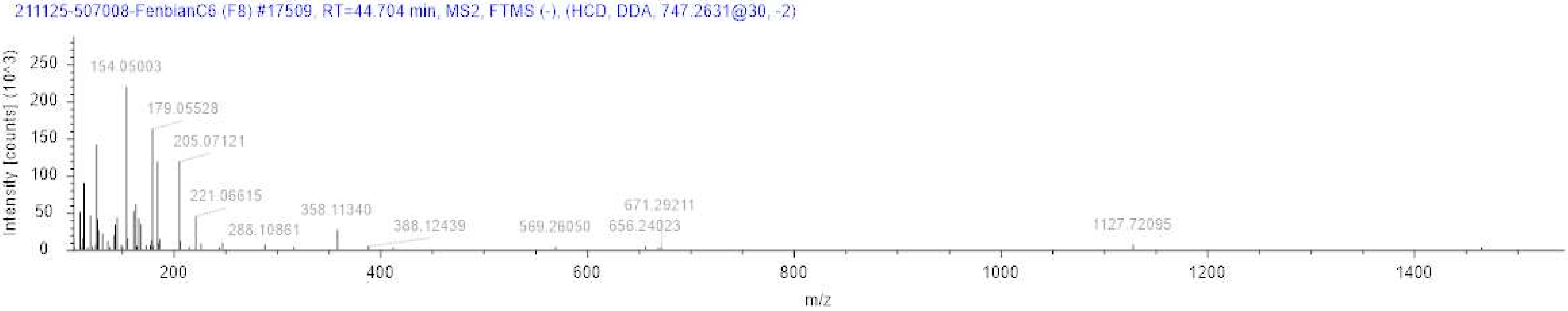

© Reported with Compound Discoverer 3.3

Compounds

Structure	Name	RT [min]	Formula	Calc. MW	Areas
n/a		43.28	n/a	1509.53001	3.74e7 7.32e8 2.58e8

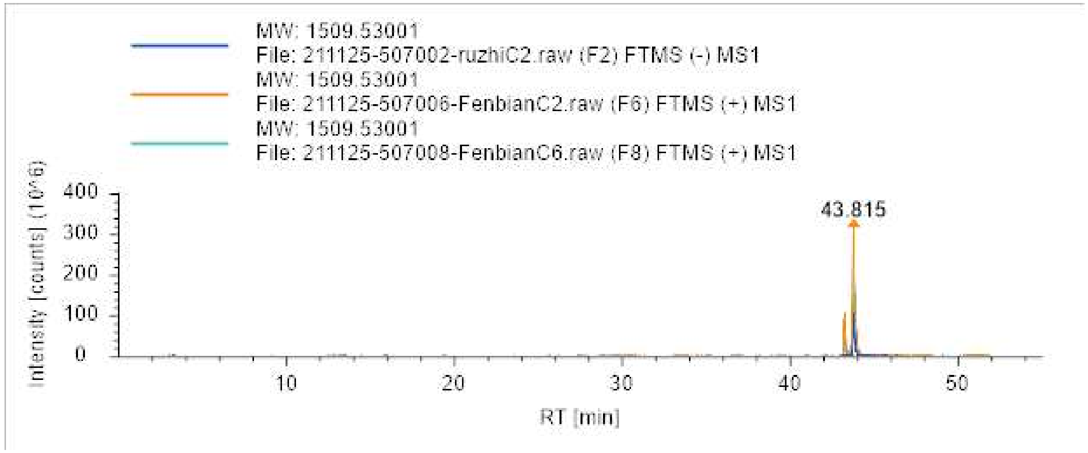

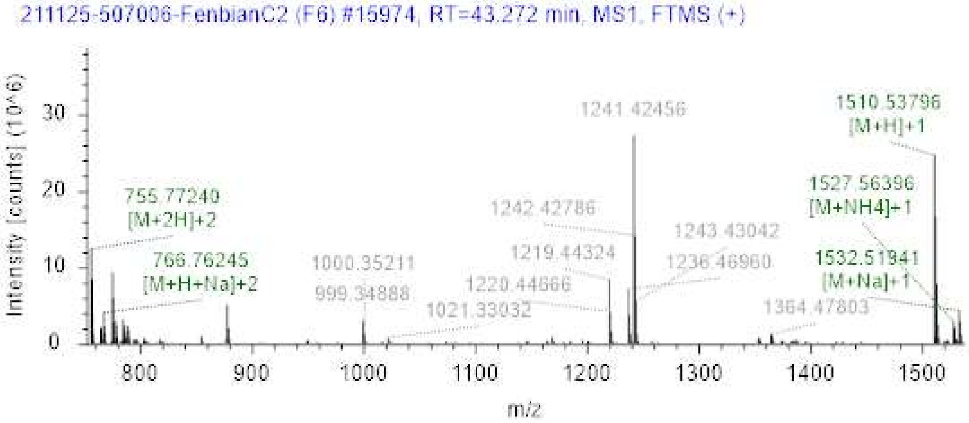

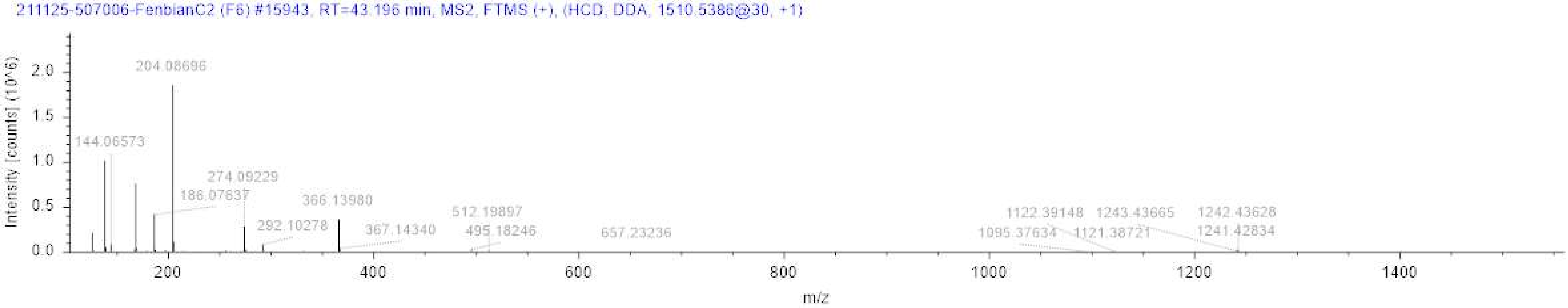

© Reported with Compound Discoverer 3.3

Compounds

Structure	Name	RT [min]	Formula	Calc. MW	Areas
n/a		43.82	n/a	1509.53671	8.54e8 1.34e9 5.16e8 6.45e8 3.84e9 2.63e9

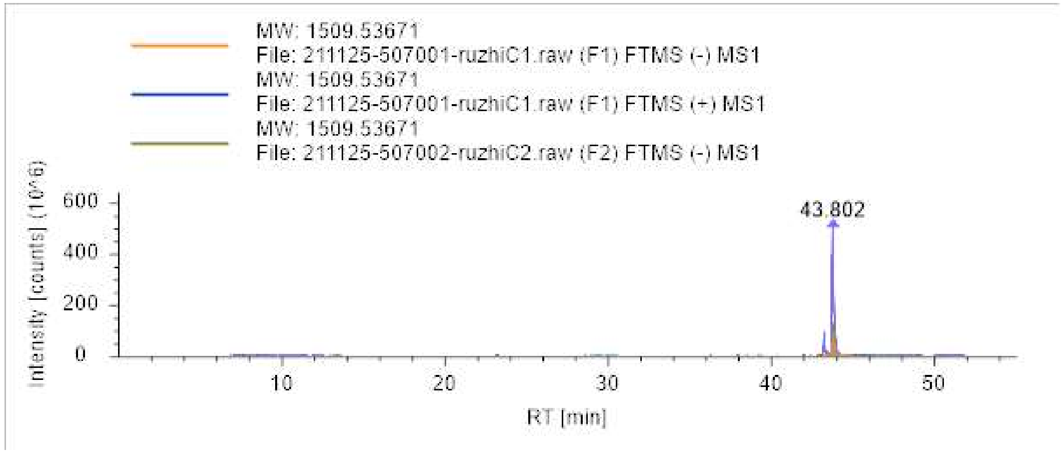

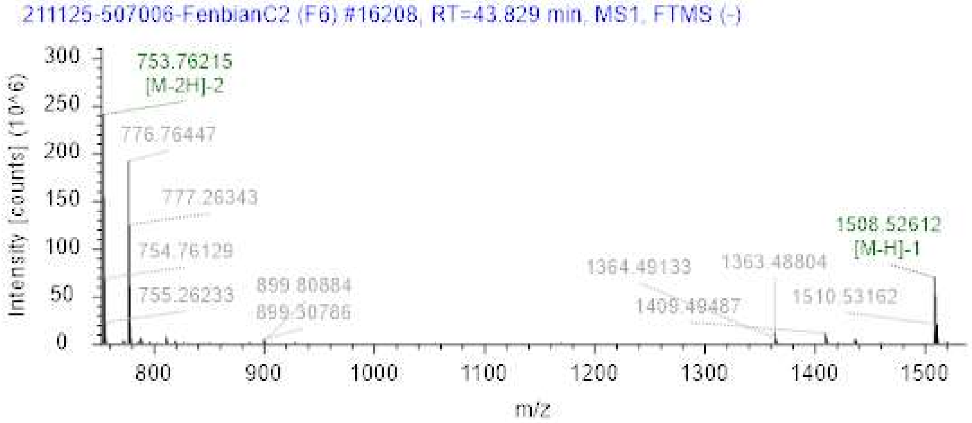

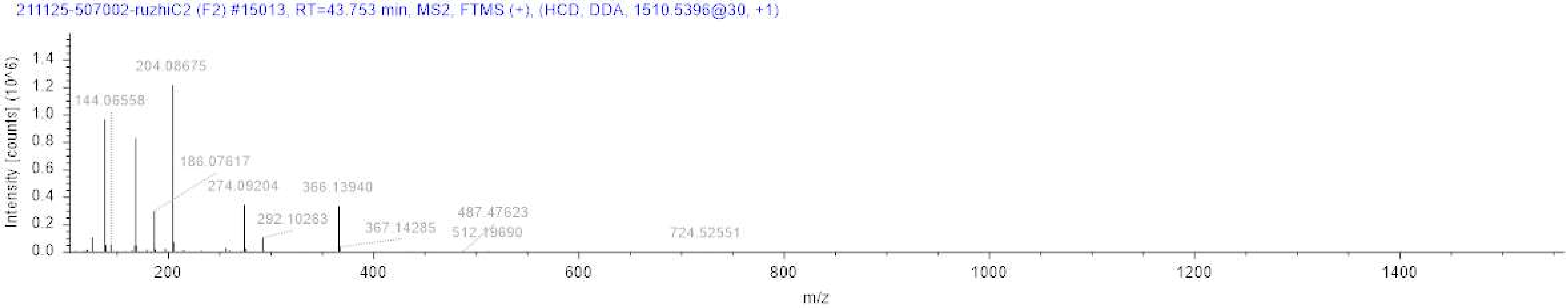

© Reported with Compound Discoverer 3.3

Structure	Name	RT [min]	Formula	Calc. MW	Areas
n/a		44.73	n/a	1510.55067	2.94e8, 2.98e8, 4.17e8, 3.53e7, 2.89e9

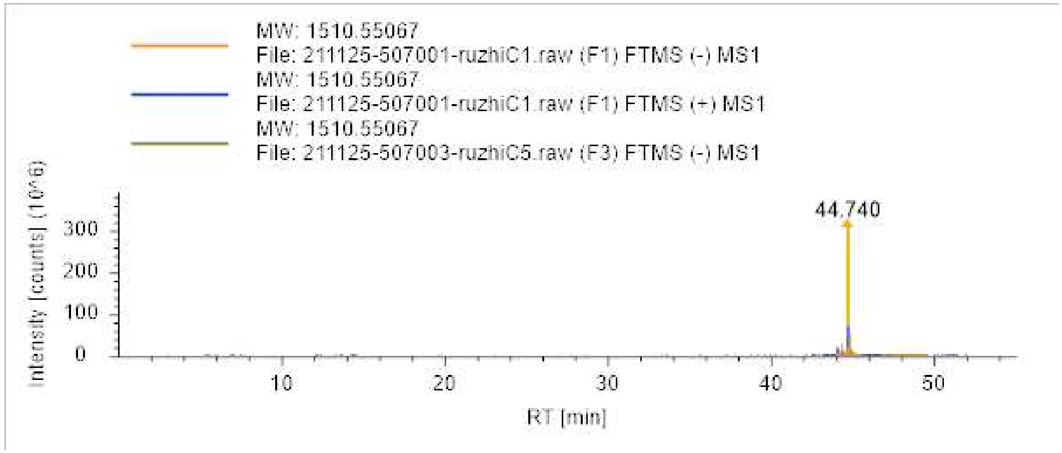

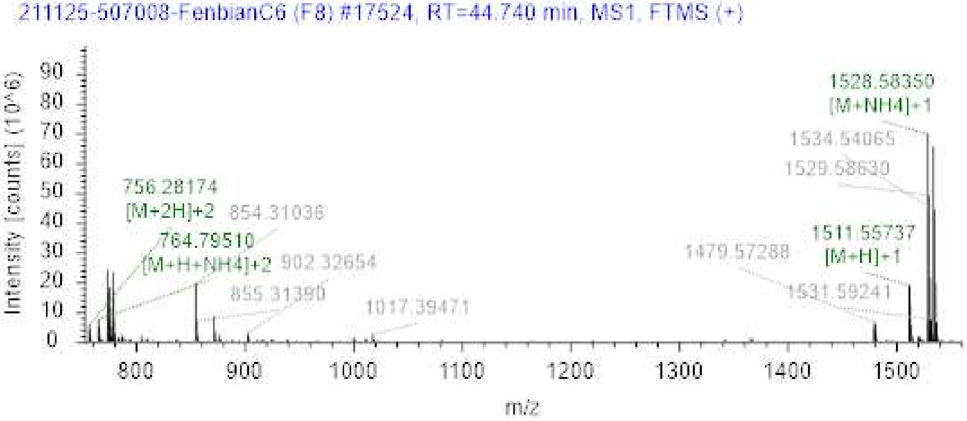

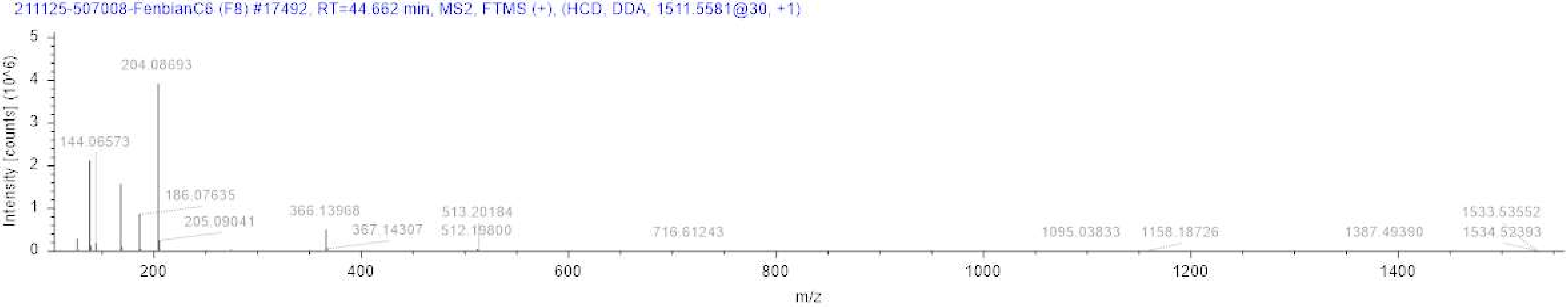

© Reported with Compound Discoverer 3.3

Structure	Name	RT [min]	Formula	Calc. MW	Areas
n/a		44.74	n/a	1511.55271	3.40e7

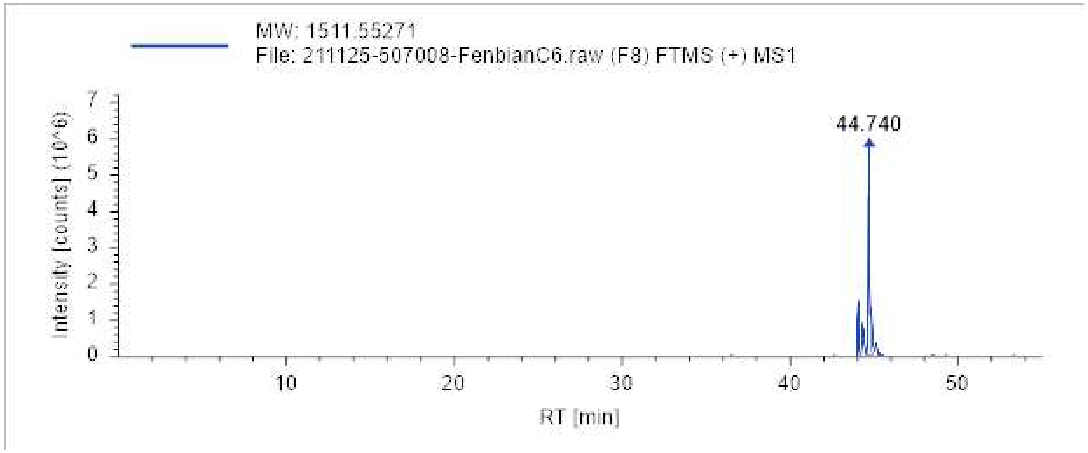

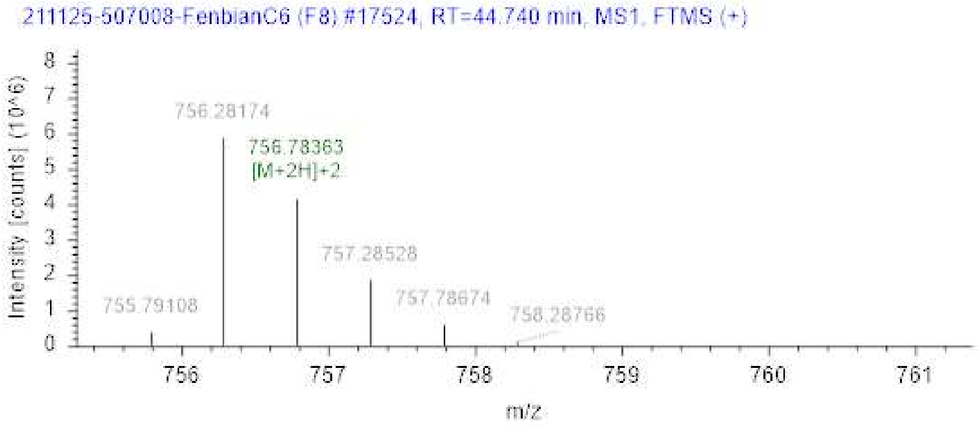

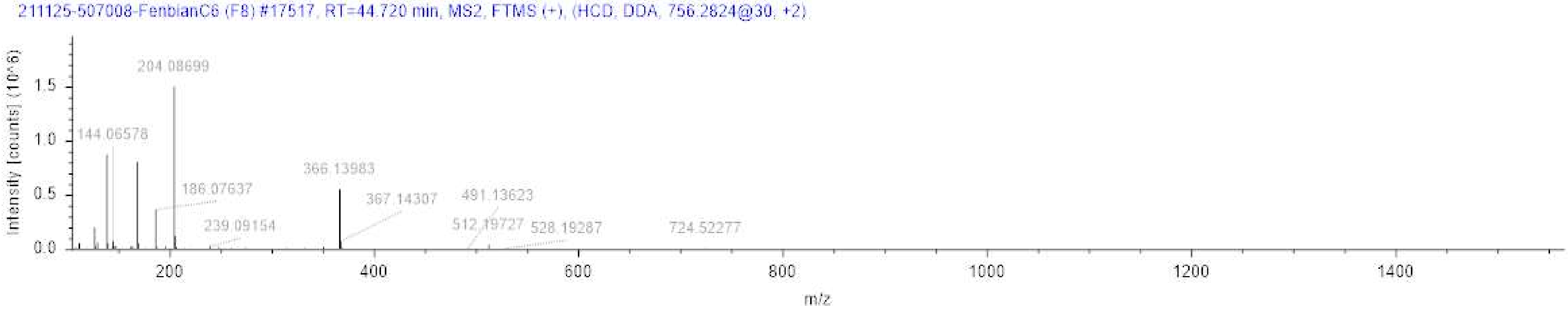

© Reported with Compound Discoverer 3.3

Compounds

Structure	Name	RT [min]	Formula	Calc. MW	Areas
n/a		42.66	n/a	1519.56235	2.72e7

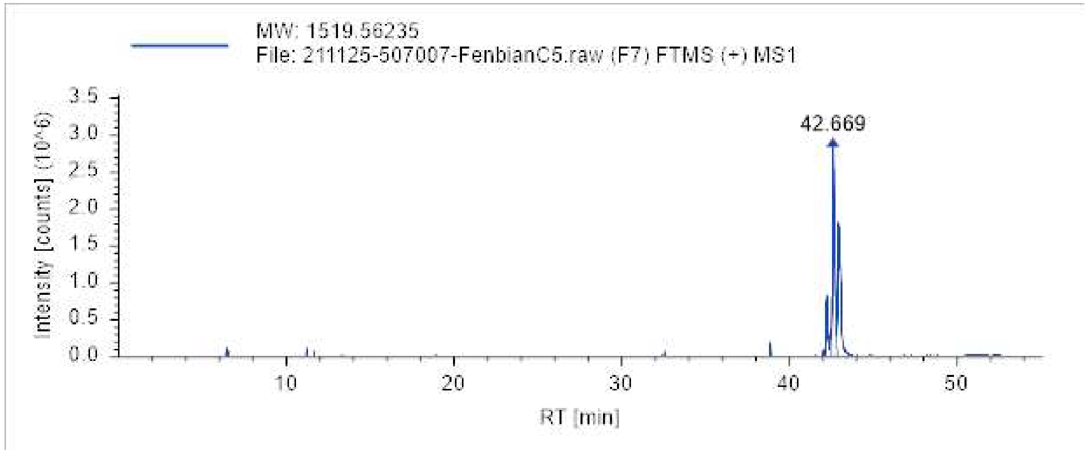

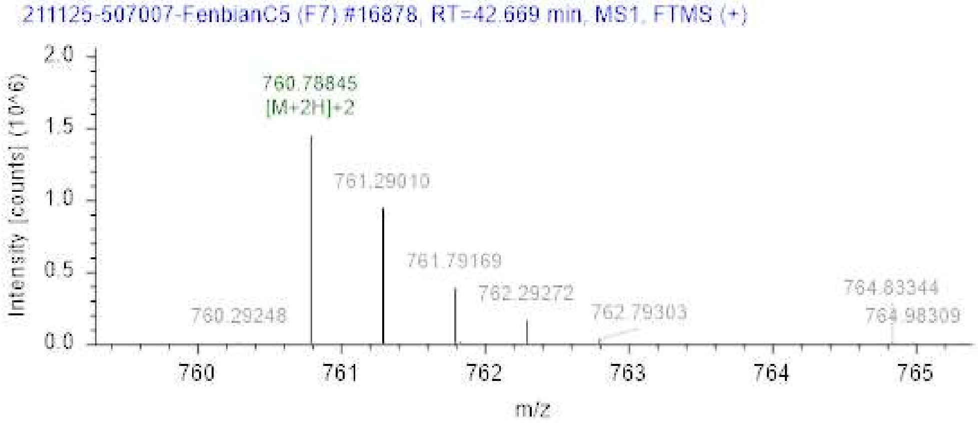

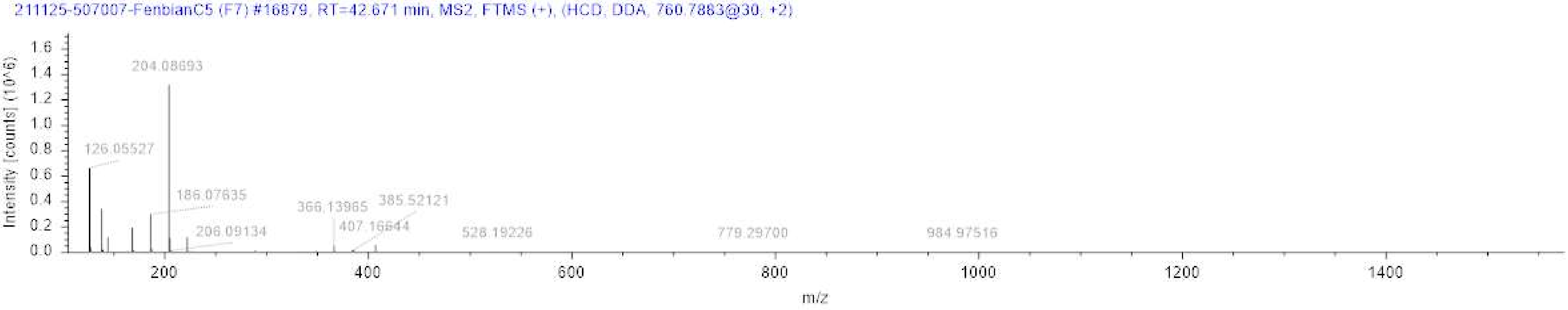

© Reported with Compound Discoverer 3.3

Compounds

Structure	Name	RT [min]	Formula	Calc. MW	Areas
n/a		43.76	n/a	1525.52925	1.66e7 1.25e7

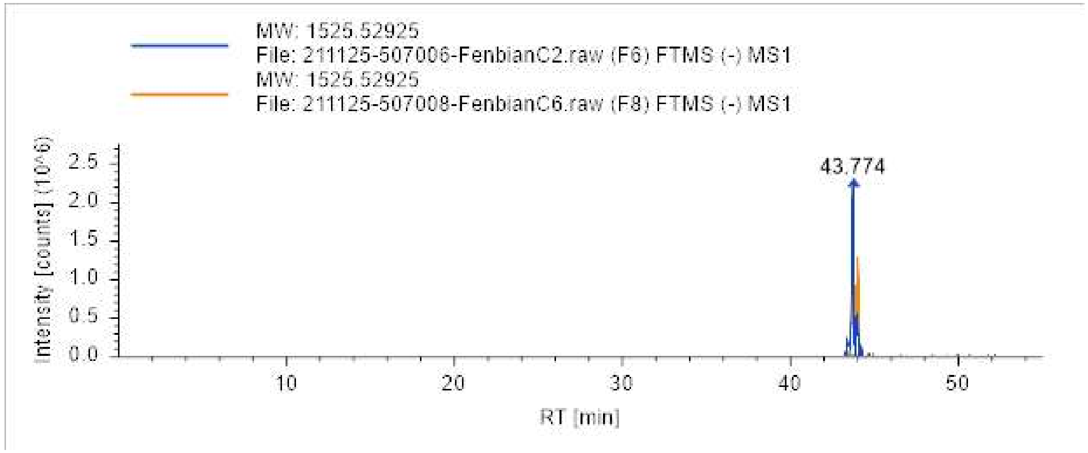

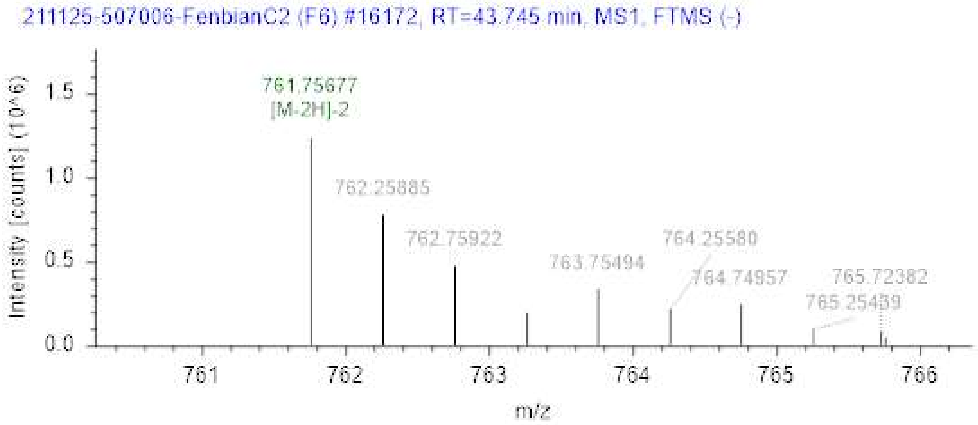

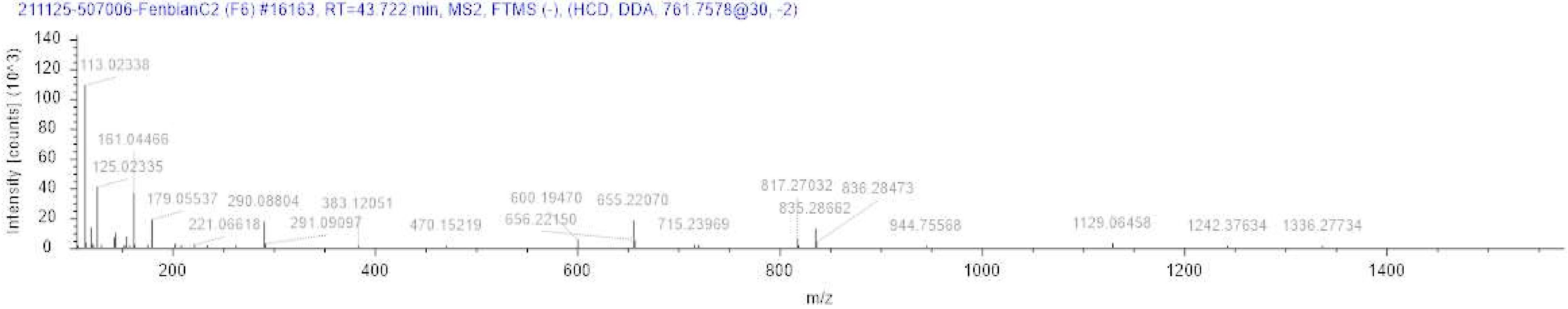

© Reported with Compound Discoverer 3.3

Compounds

Structure	Name	RT [min]	Formula	Calc. MW	Areas
n/a		45.02	n/a	1526.55125	8.65e6; 3.74e7; 2.49e7

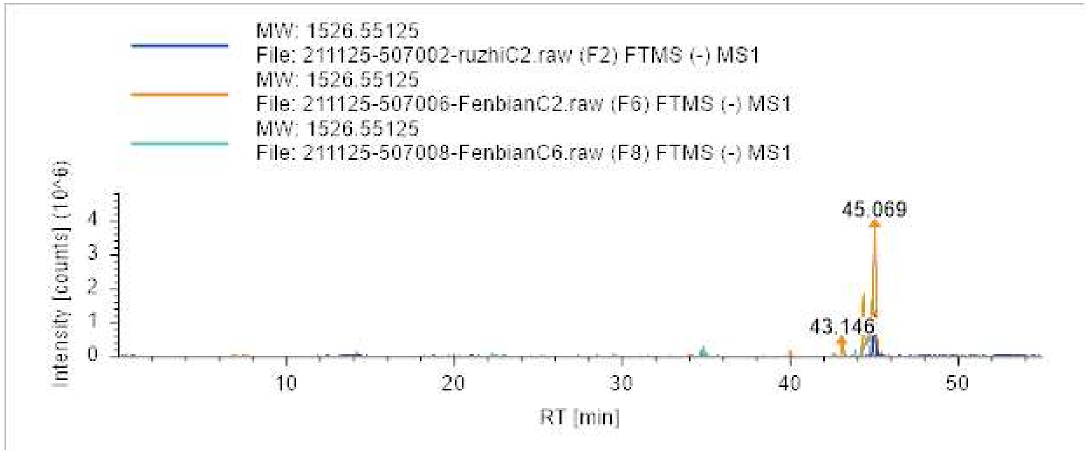

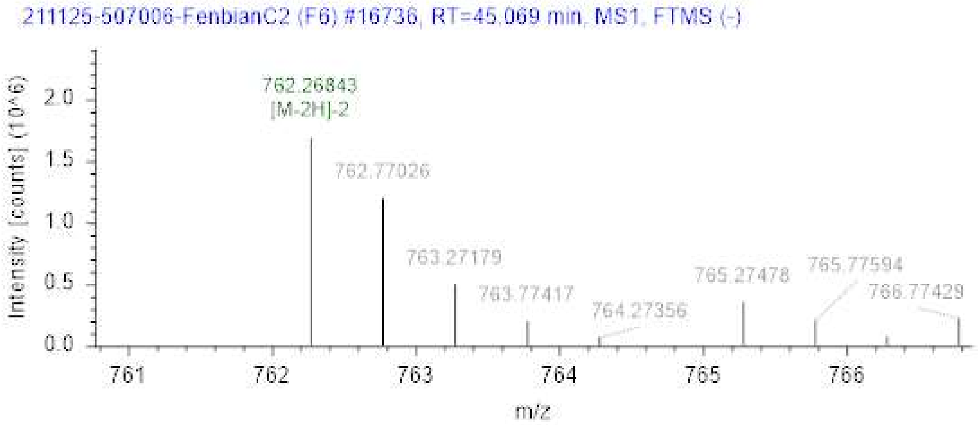

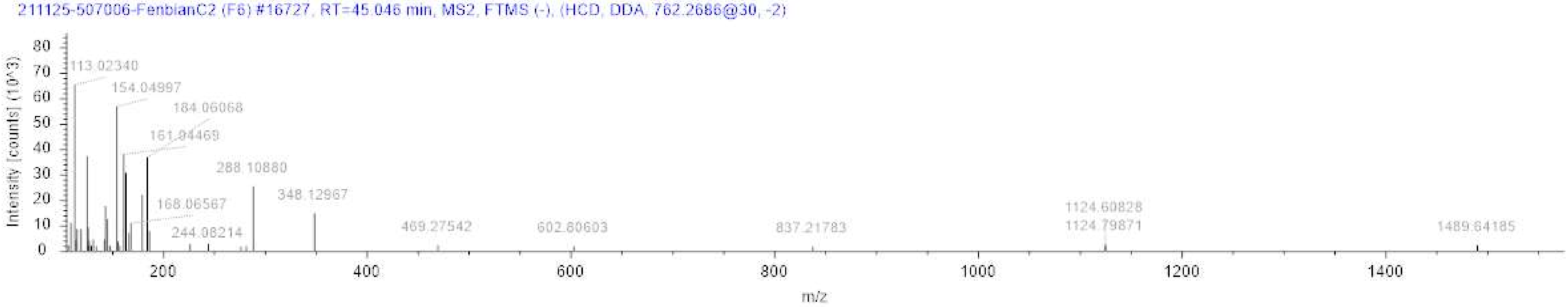

© Reported with Compound Discoverer 3.3

Compounds

Structure	Name	RT [min]	Formula	Calc. MW	Areas
n/a		43.84	n/a	1526.55575	4.15e7, 4.44e8, 3.22e7

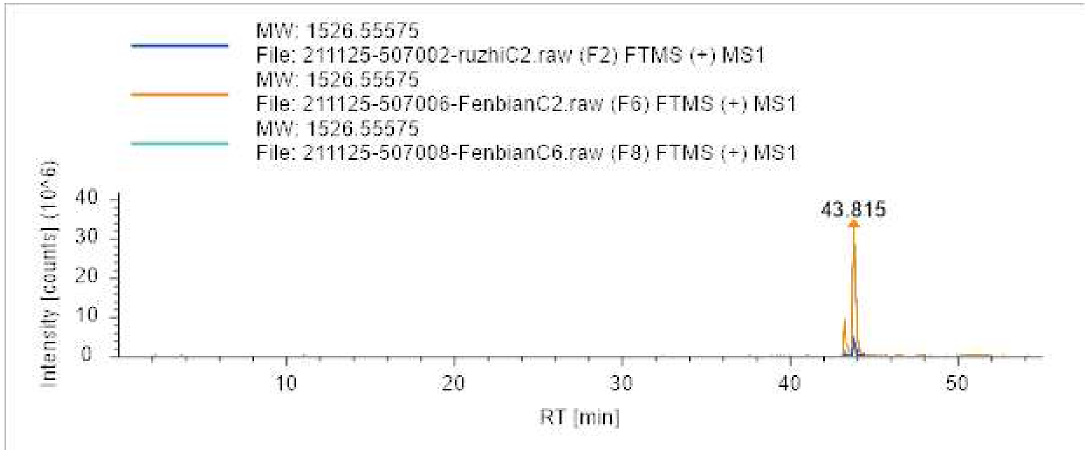

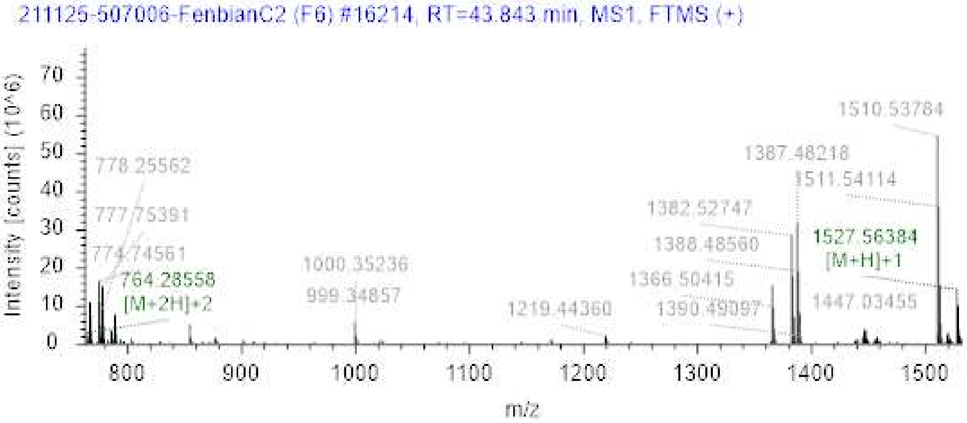

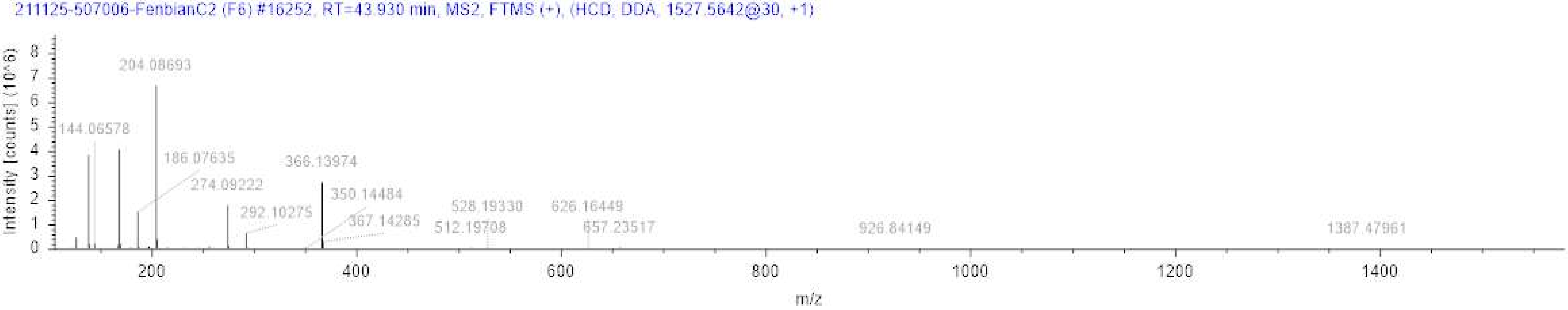

© Reported with Compound Discoverer 3.3

Structure	Name	RT [min]	Formula	Calc. MW	Areas
n/a		43.28	n/a	1528.56028	9.59e6

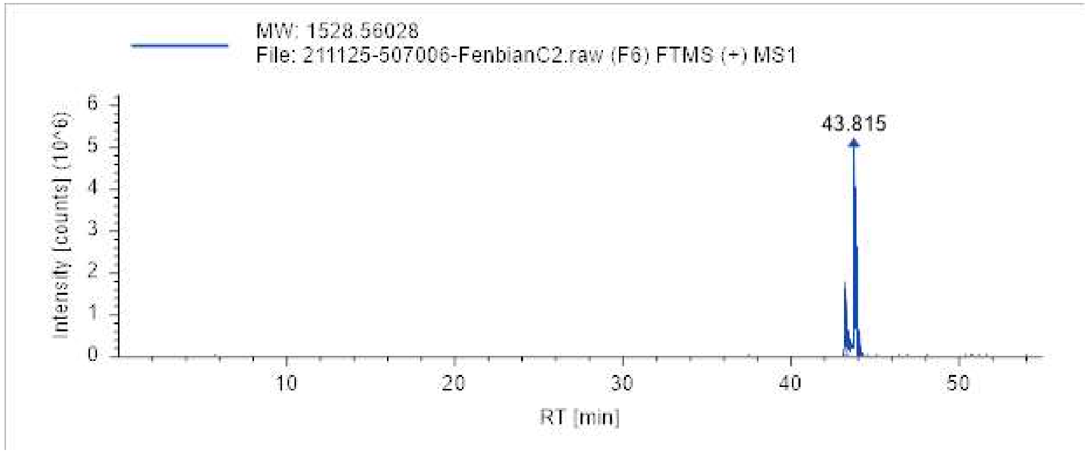

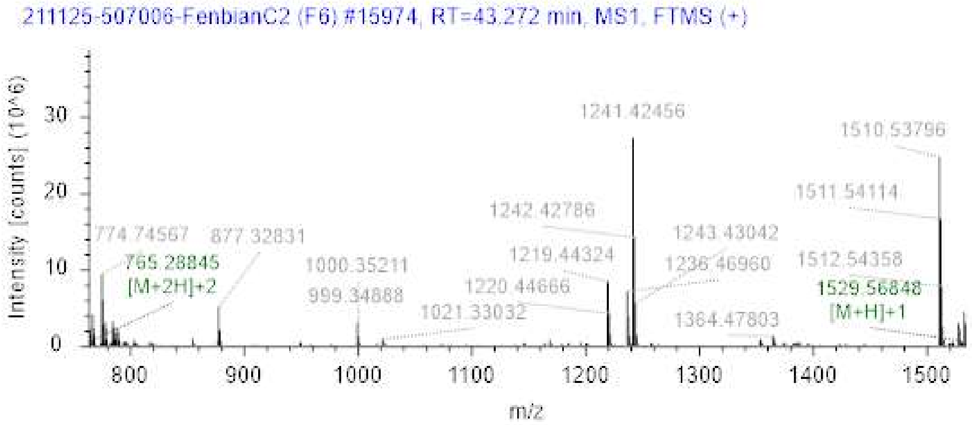

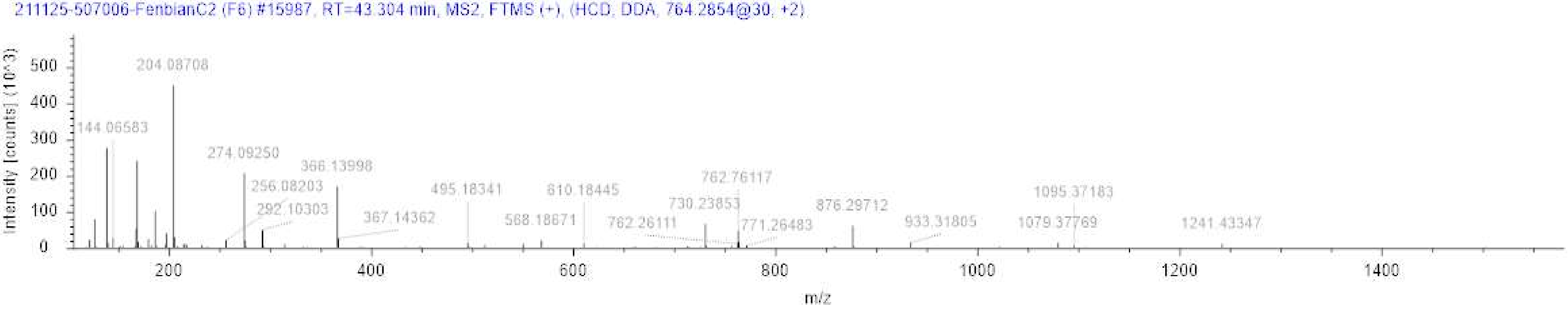

© Reported with Compound Discoverer 3.3

Compounds

Structure	Name	RT [min]	Formula	Calc. MW	Areas
n/a		44.90	n/a	1533.53843	4.95e6

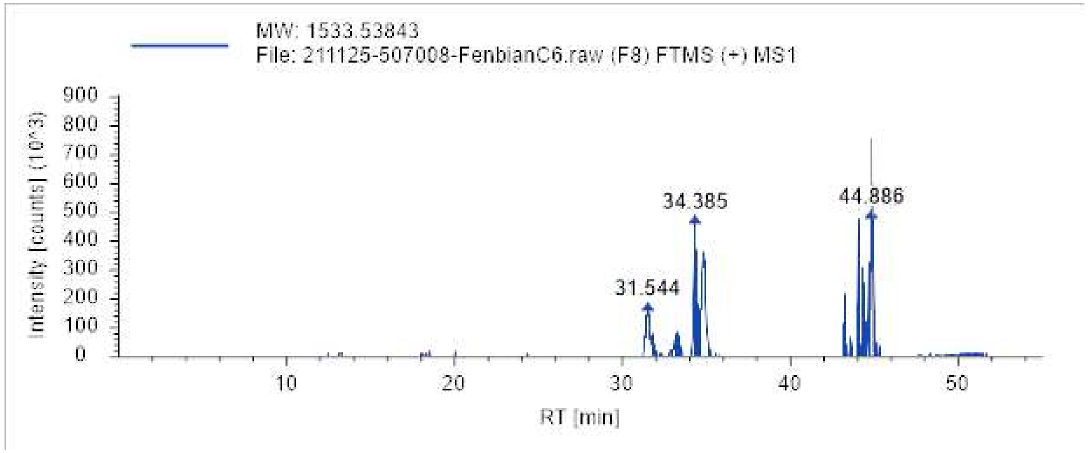

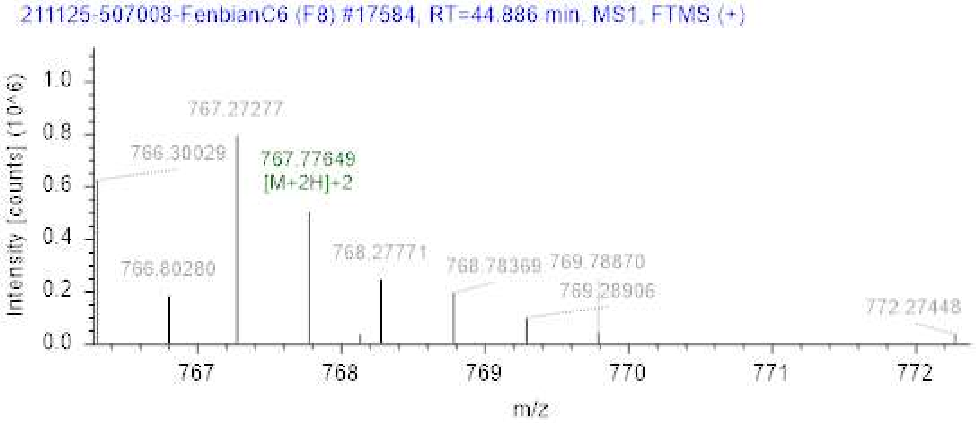

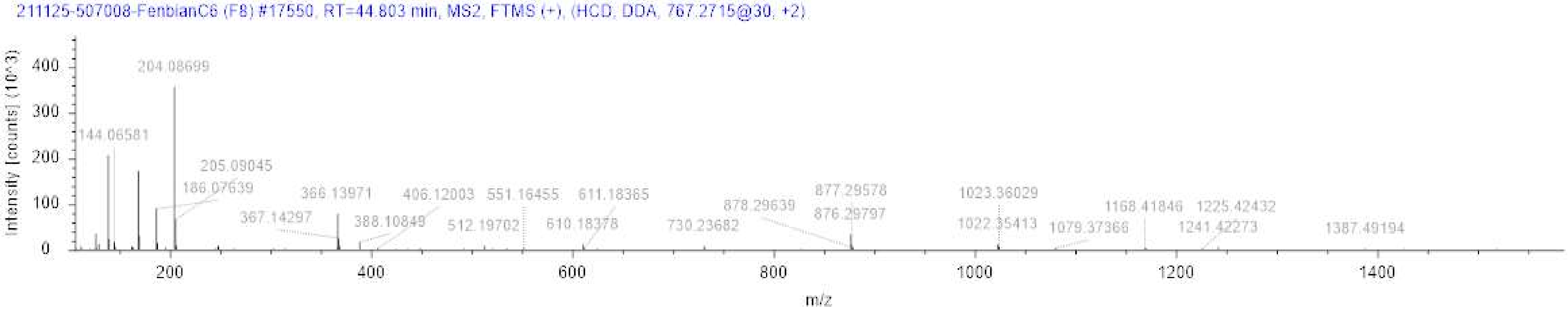

© Reported with Compound Discoverer 3.3

Compounds

Structure	Name	RT [min]	Formula	Calc. MW	Areas
n/a		43.40	n/a	1550.56631	9.44e6

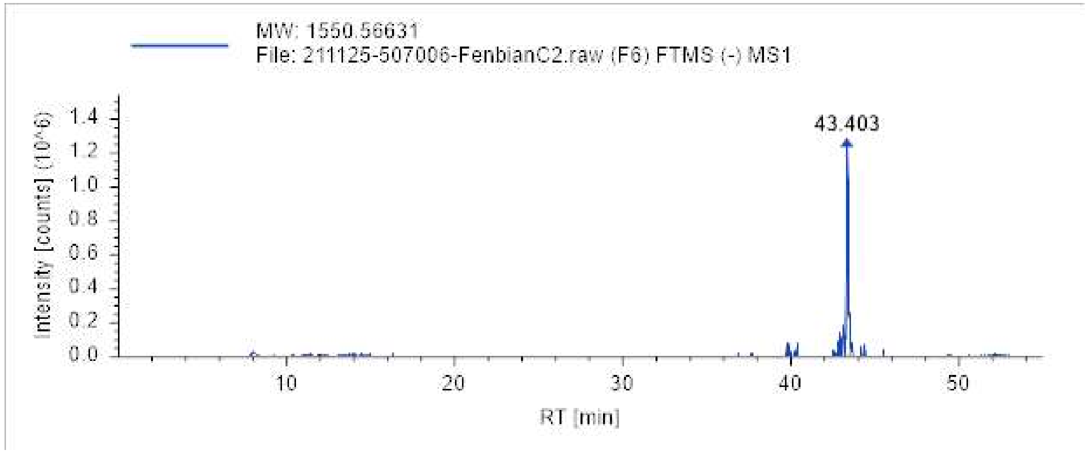

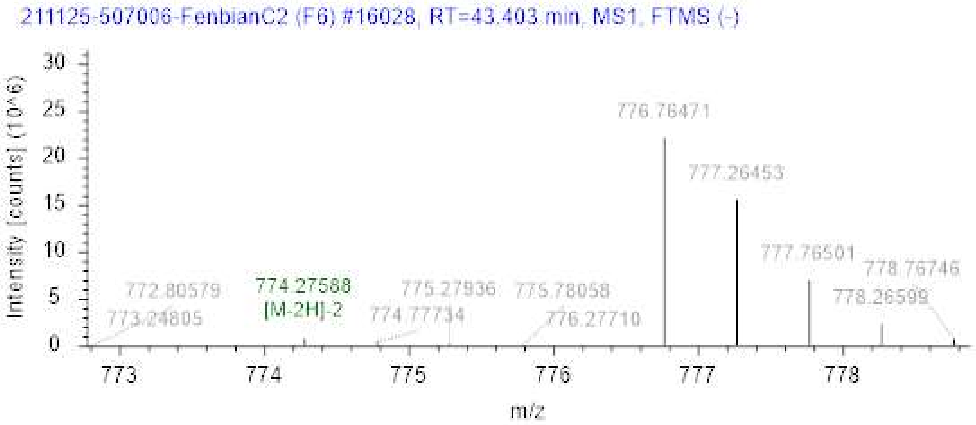

© Reported with Compound Discoverer 3.3

Compounds

Structure	Name	RT [min]	Formula	Calc. MW	Areas
n/a		43.26	n/a	1566.55179	7.95e7; 1.15e7

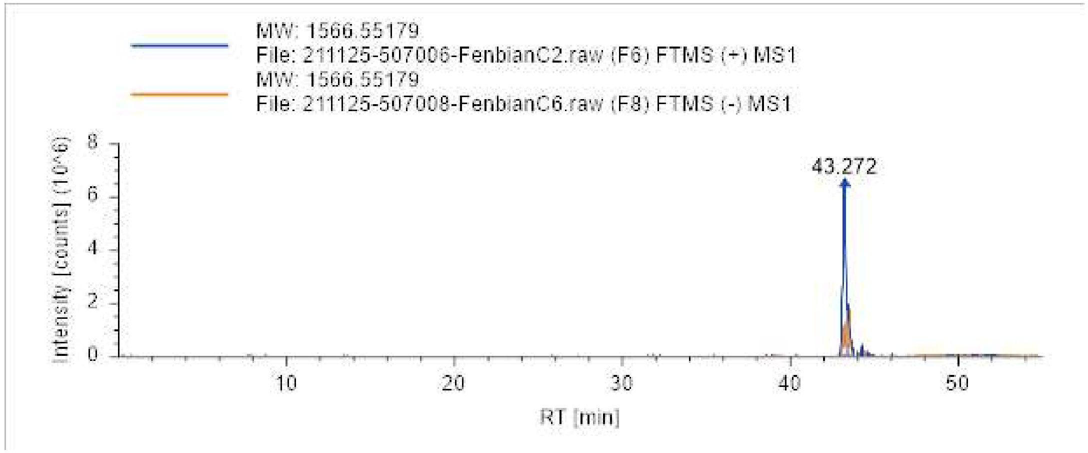

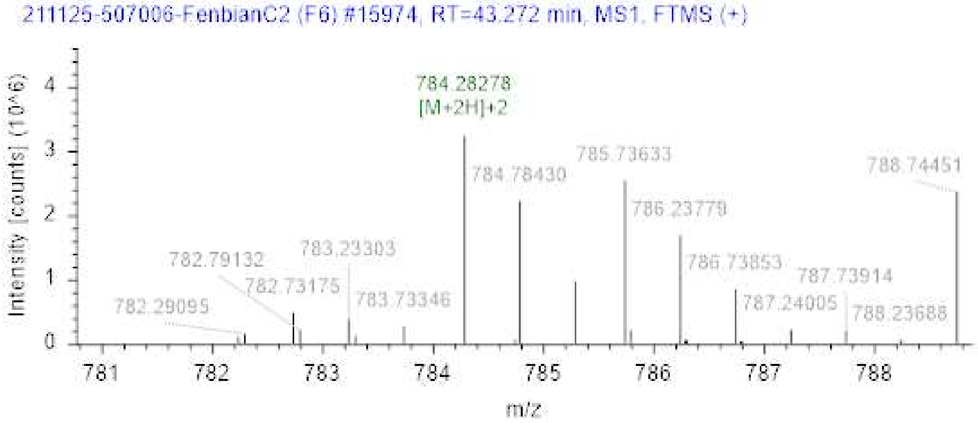

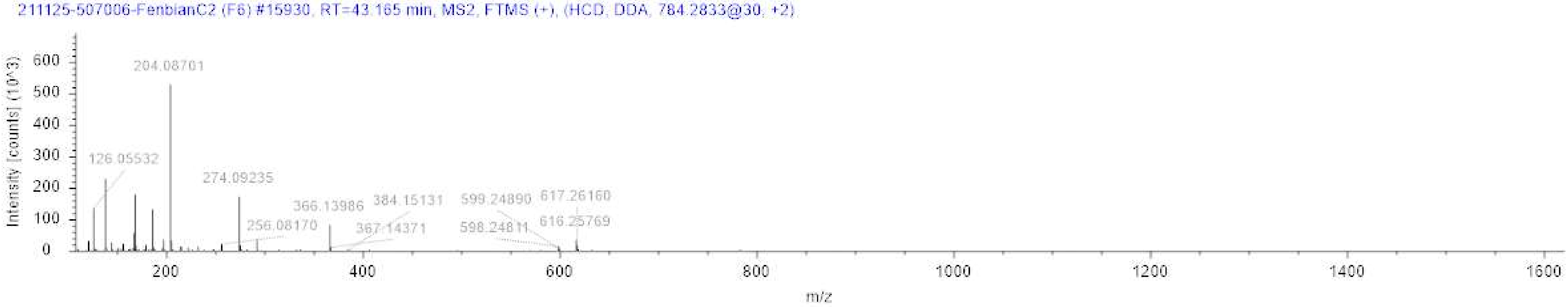

© Reported with Compound Discoverer 3.3

Compounds

Structure	Name	RT [min]	Formula	Calc. MW	Areas
n/a		44.47	n/a	1566.56003	8.61e7

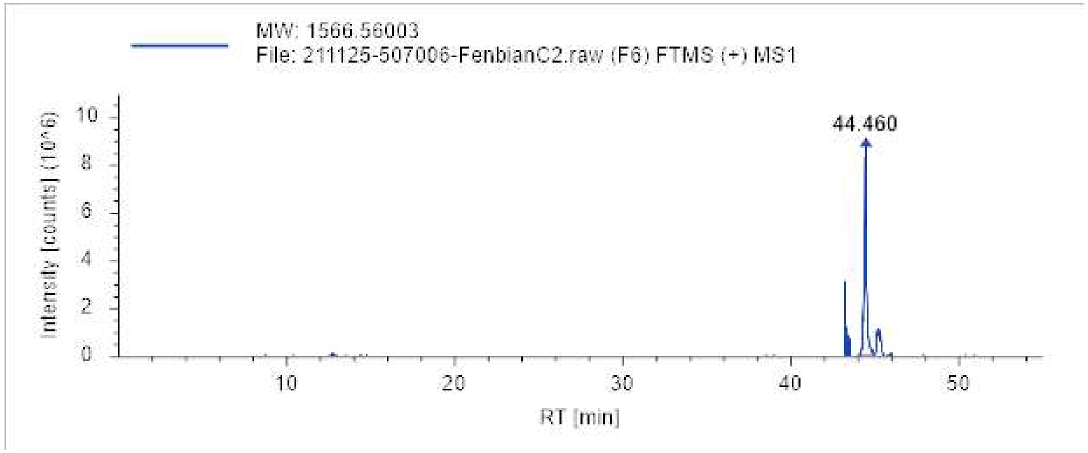

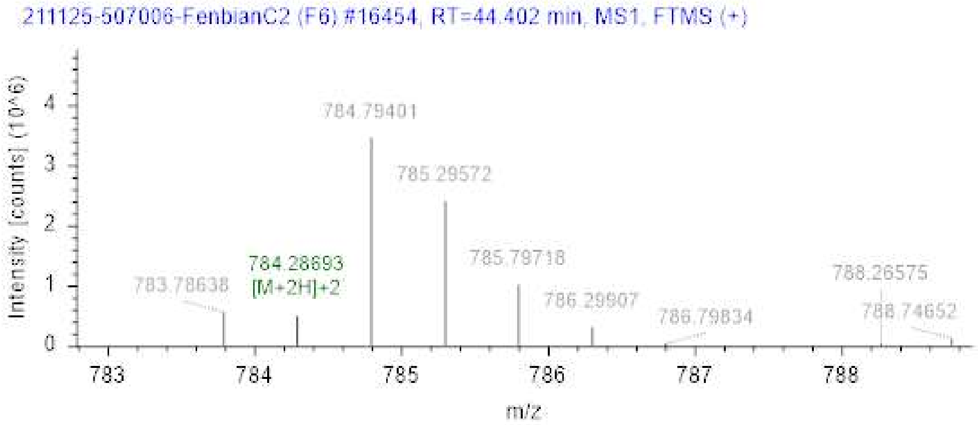

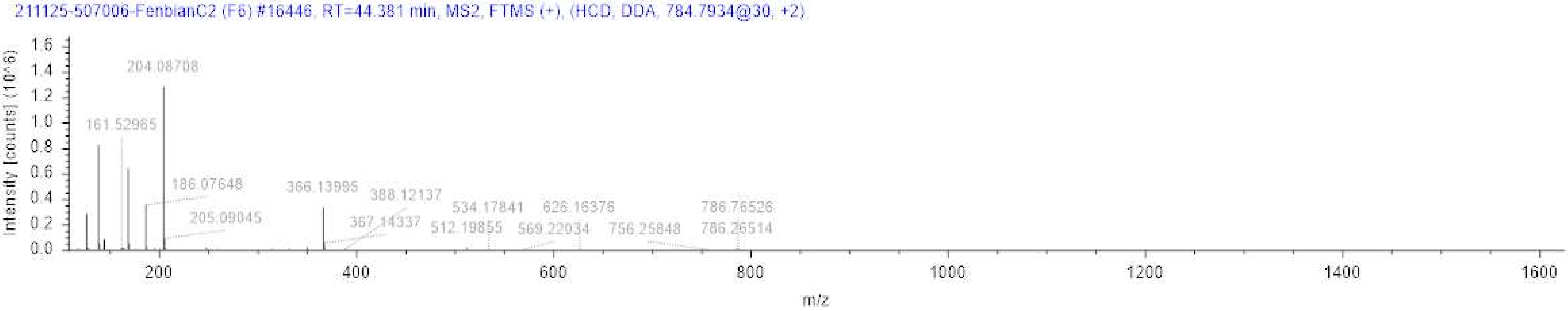

© Reported with Compound Discoverer 3.3

Compounds

Structure	Name	RT [min]	Formula	Calc. MW	Areas
n/a		43.26	n/a	1567.55791	8.43e6

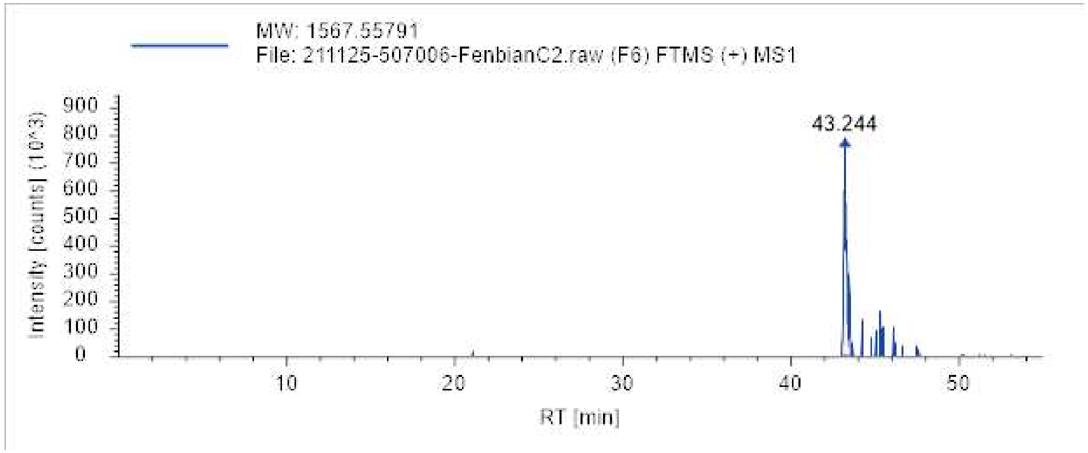

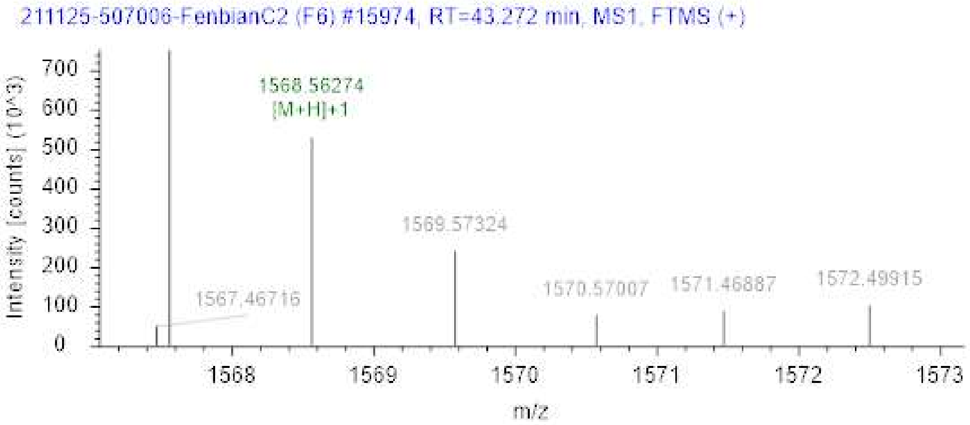

© Reported with Compound Discoverer 3.3

Structure	Name	RT [min]	Formula	Calc. MW	Areas
n/a		44.47	n/a	1567.57566	1.05e9 1.05e7

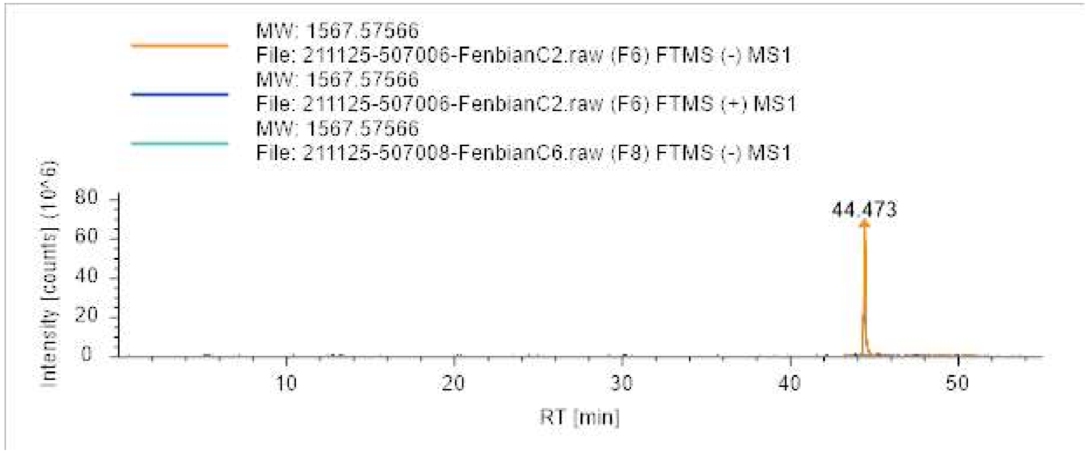

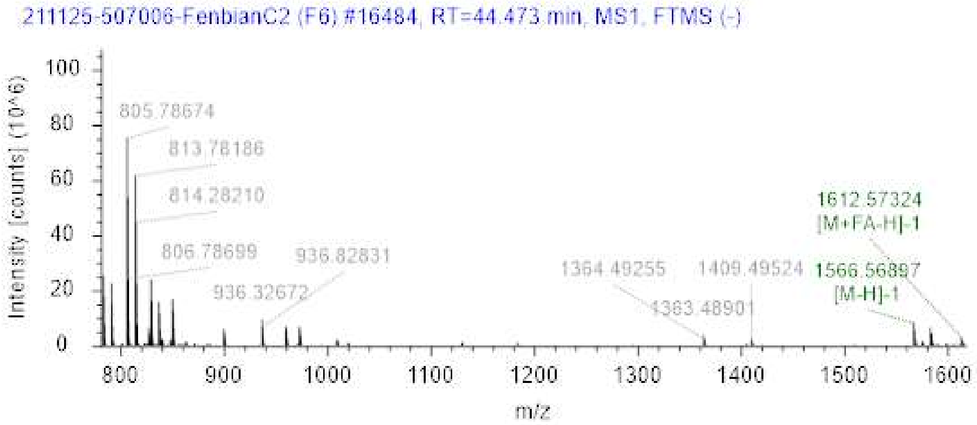

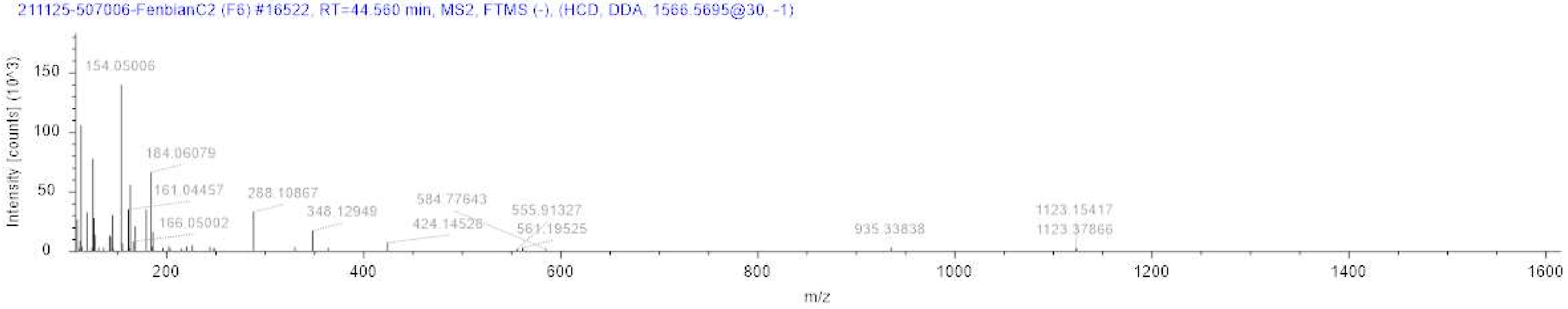

© Reported with Compound Discoverer 3.3

Compounds

Structure	Name	RT [min]	Formula	Calc. MW	Areas
n/a		45.28	n/a	1583.56835	7.84e7

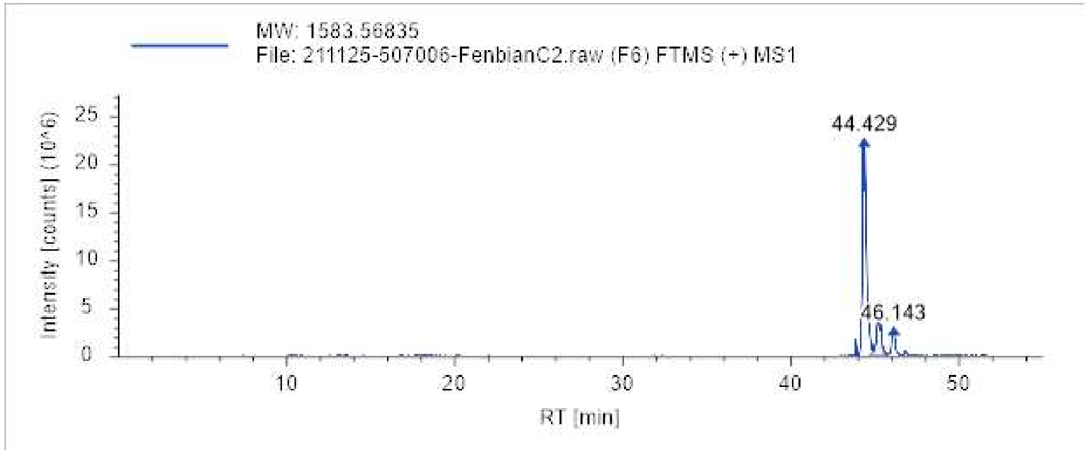

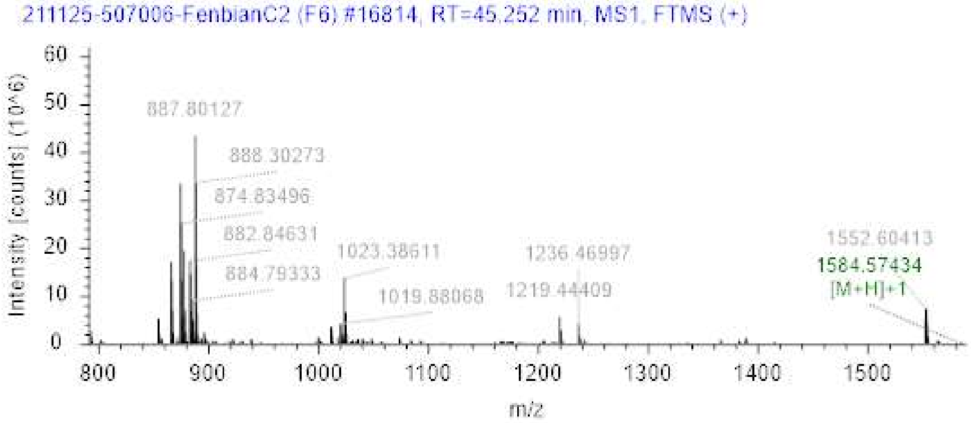

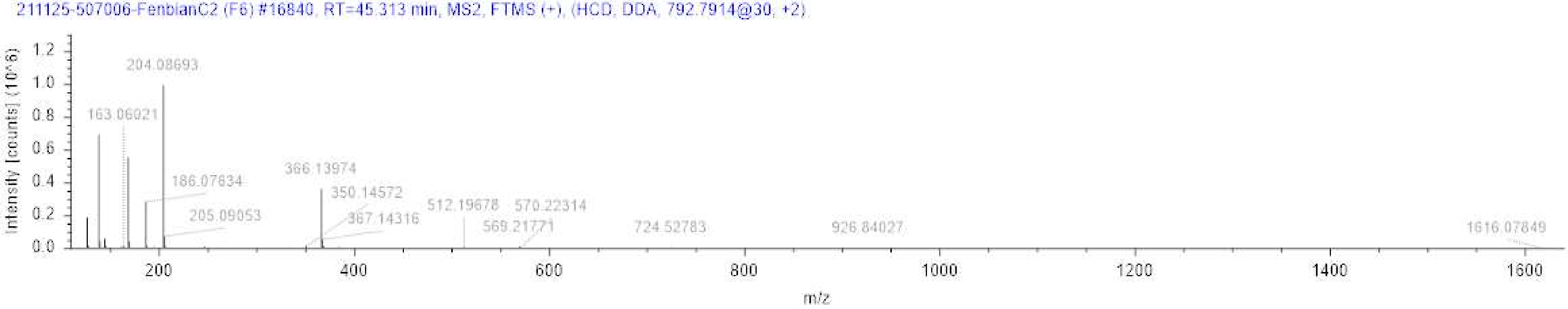

© Reported with Compound Discoverer 3.3

Compounds

Structure	Name	RT [min]	Formula	Calc. MW	Areas
n/a		46.15	n/a	1583.56841	4.09e7

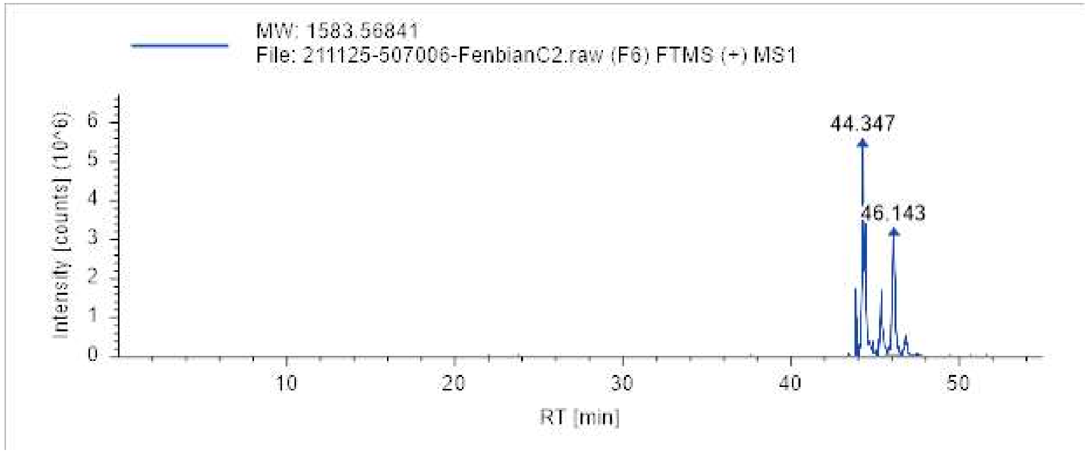

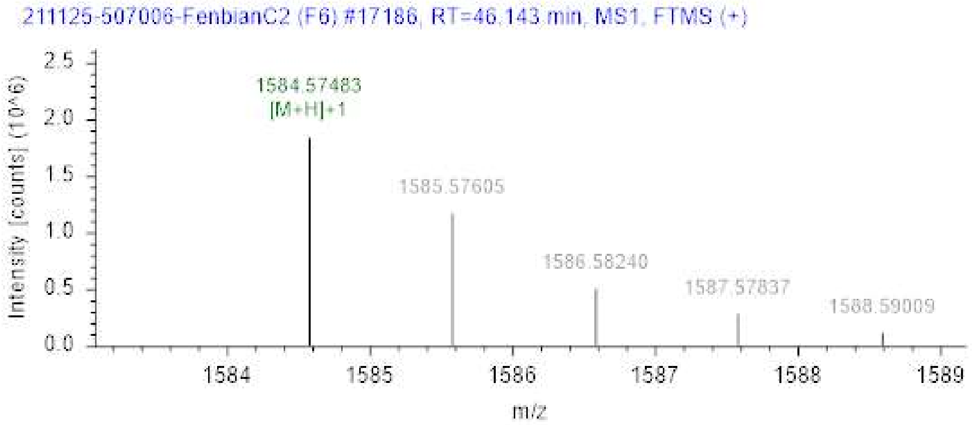

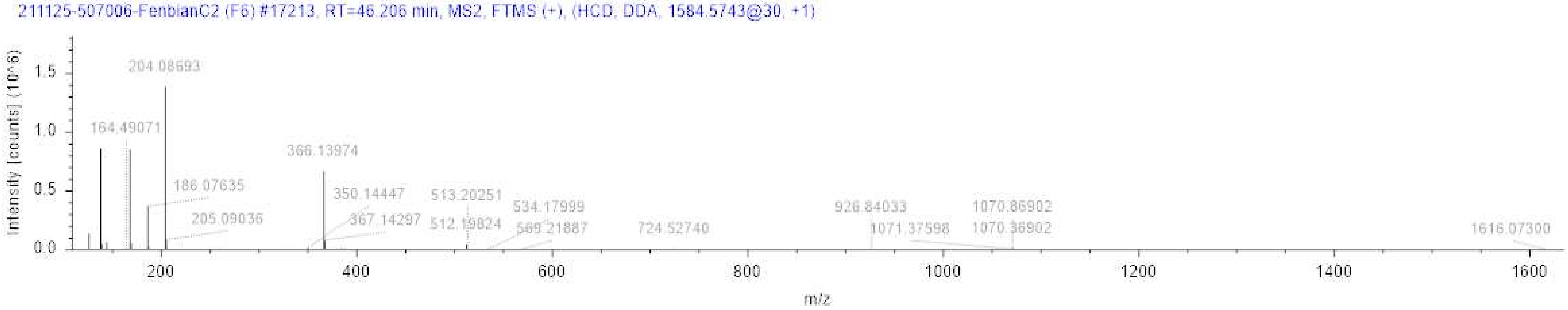

© Reported with Compound Discoverer 3.3

Compounds

Structure	Name	RT [min]	Formula	Calc. MW	Areas
n/a		44.44	n/a	1583.57076	7.64e7 1.86e8 6.96e7 6.48e7 1.68e9 5.58e8

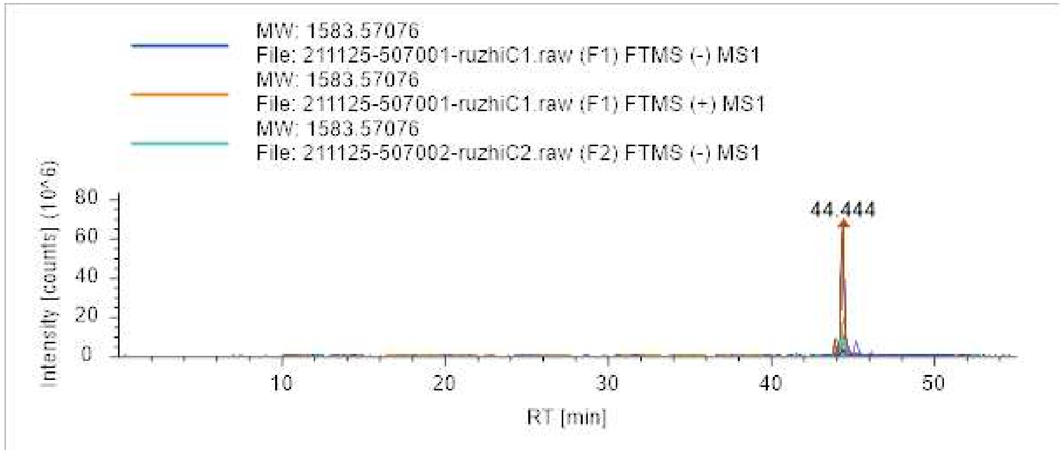

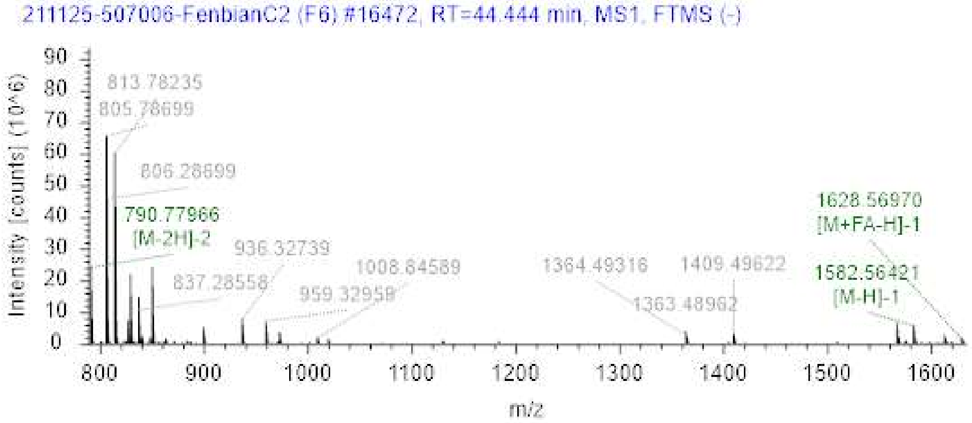

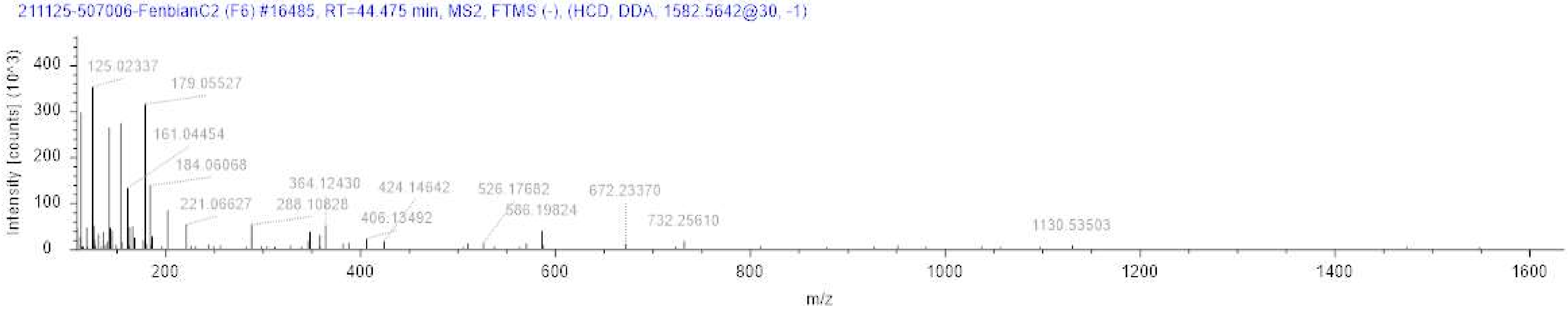

© Reported with Compound Discoverer 3.3

Compounds

Structure	Name	RT [min]	Formula	Calc. MW	Areas
n/a		44.46	n/a	1585.59177	6.88e7

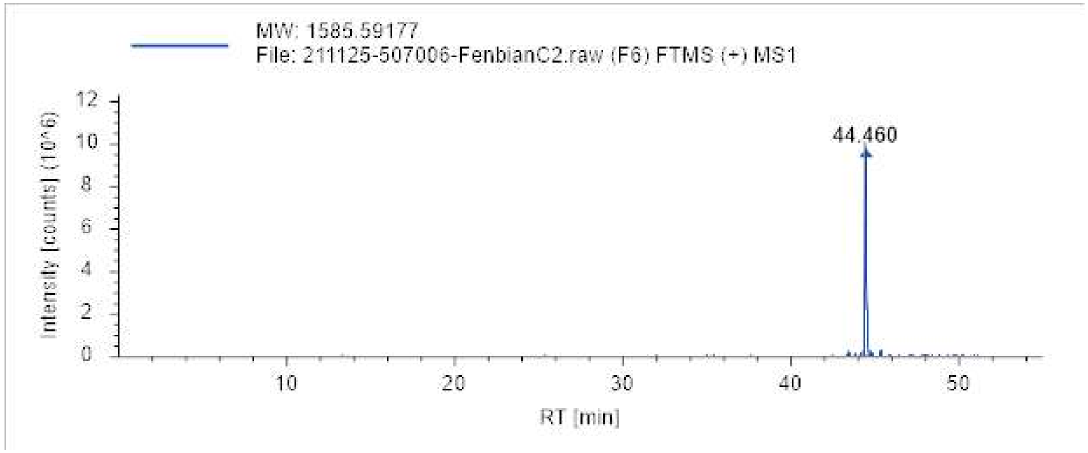

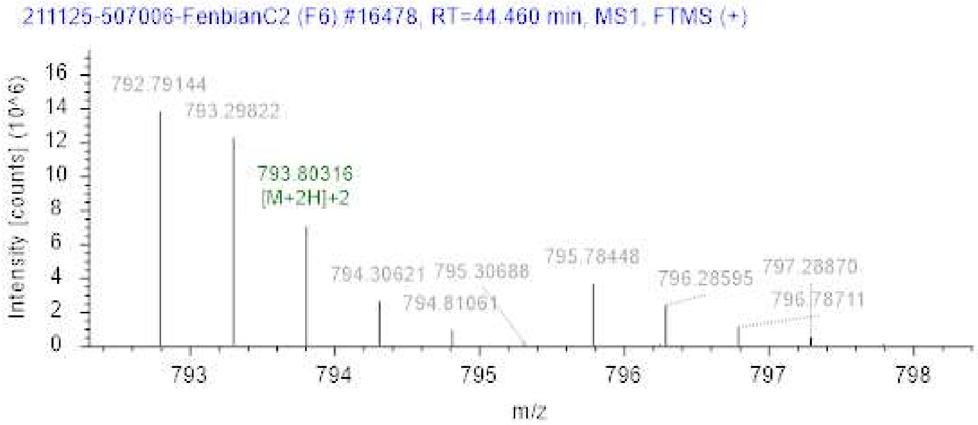

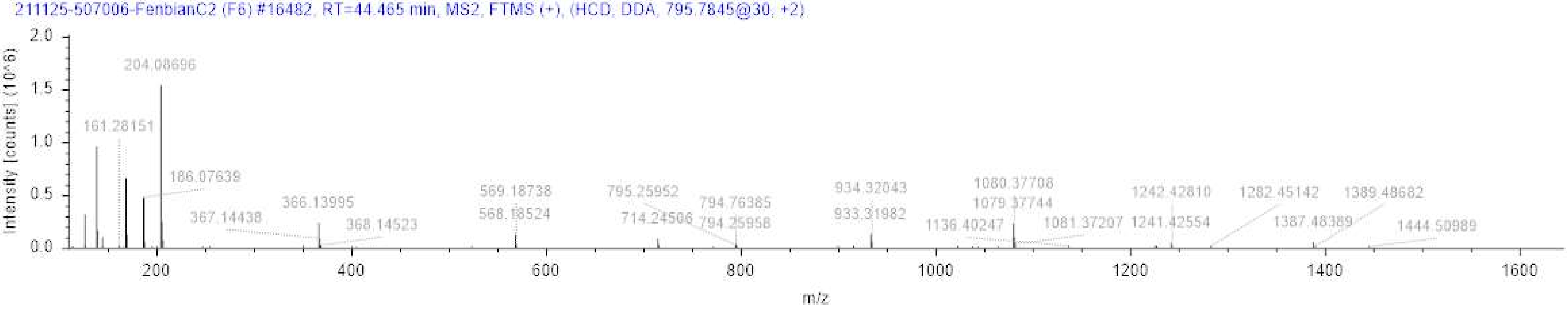

© Reported with Compound Discoverer 3.3

Compounds

Structure	Name	RT [min]	Formula	Calc. MW	Areas
n/a		44.14	n/a	1624.59782	9.28e7

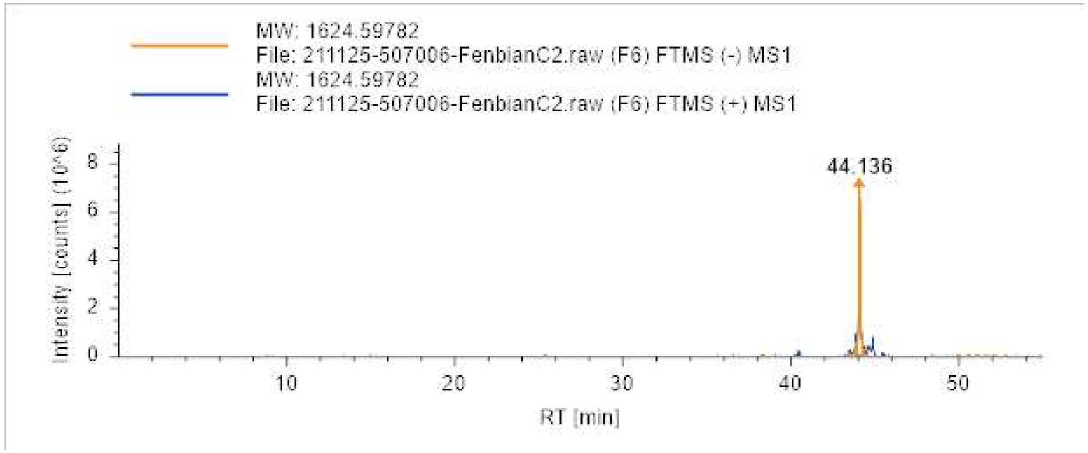

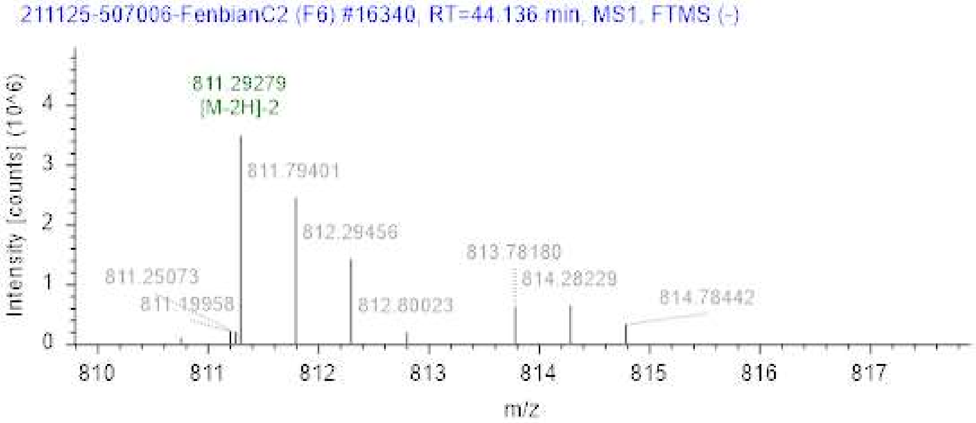

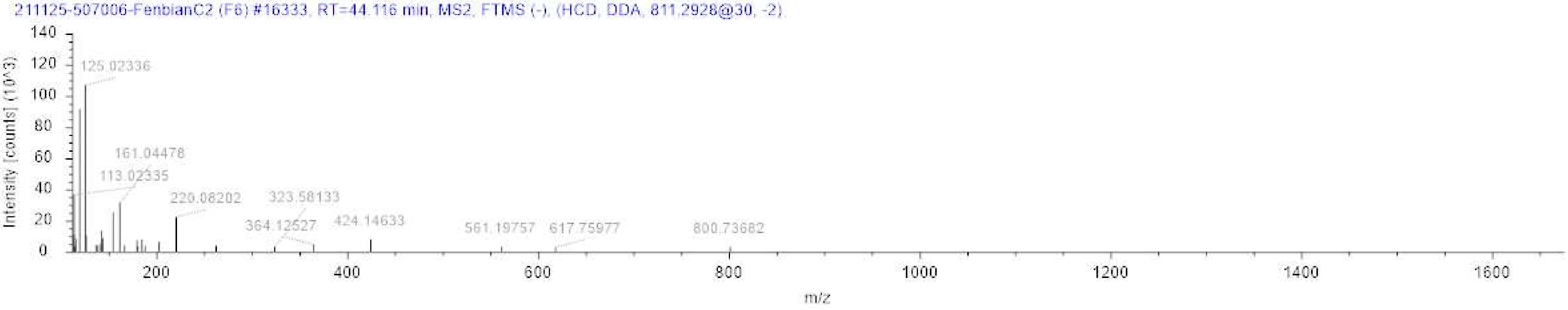

© Reported with Compound Discoverer 3.3

Compounds

Structure	Name	RT [min]	Formula	Calc. MW	Areas
n/a		44.05	n/a	1640.59165	1.73e7 7.92e7

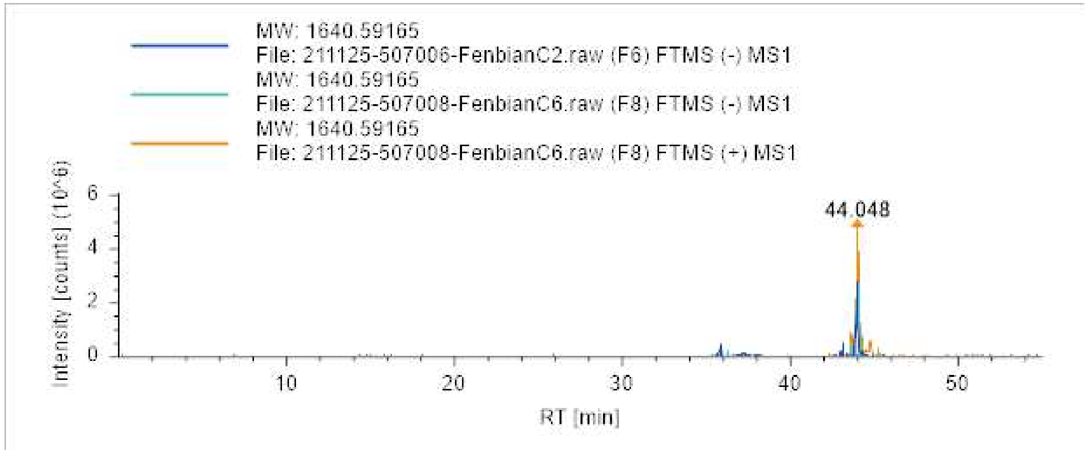

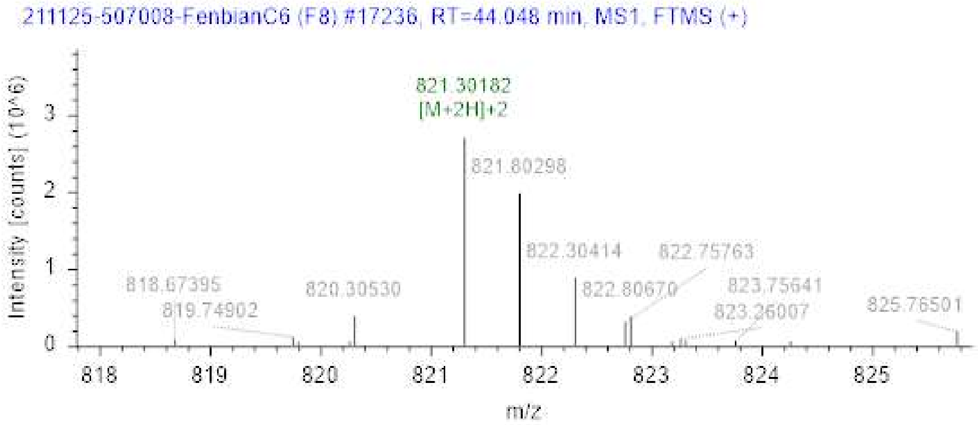

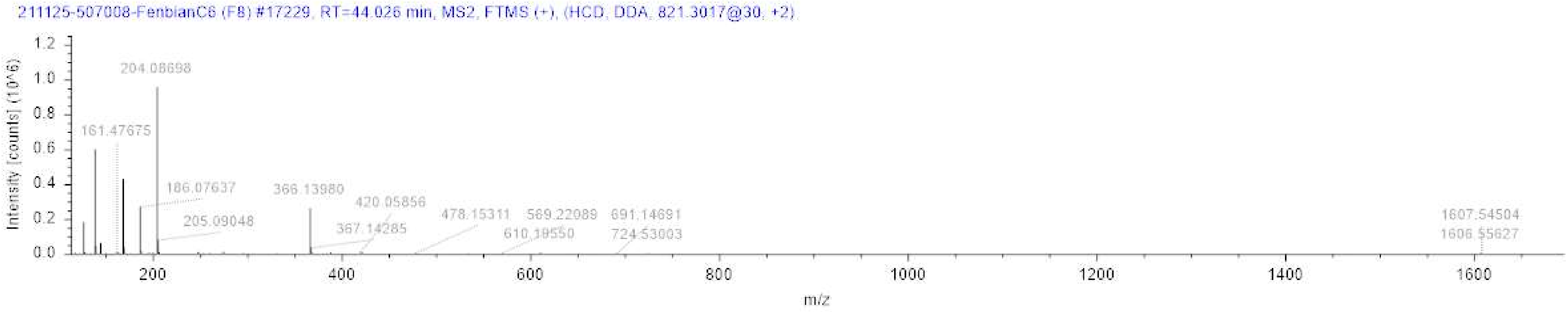

© Reported with Compound Discoverer 3.3

Structure	Name	RT [min]	Formula	Calc. MW	Areas
n/a		43.96	n/a	1654.57104	7.88e7 1.22e8 1.14e8 1.16e9 9.53e8

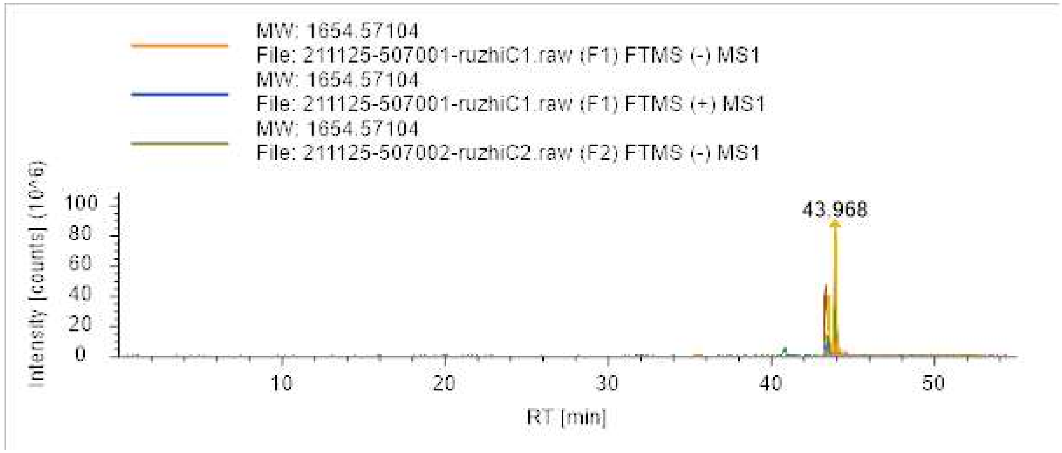

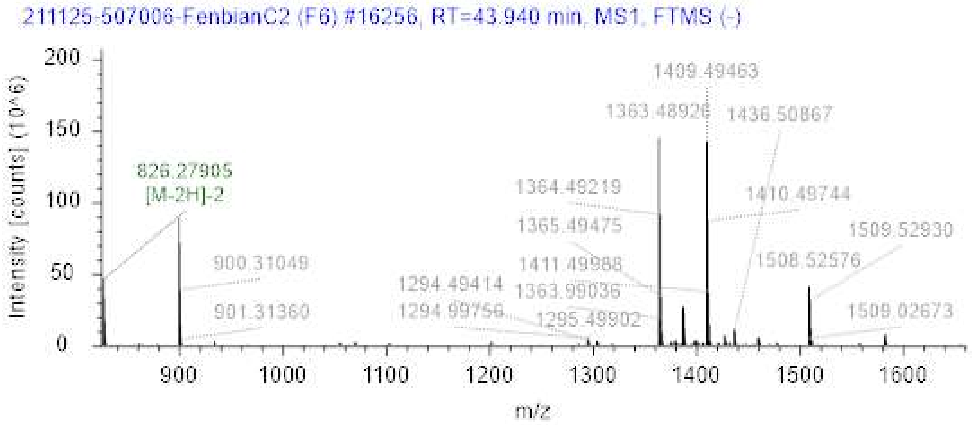

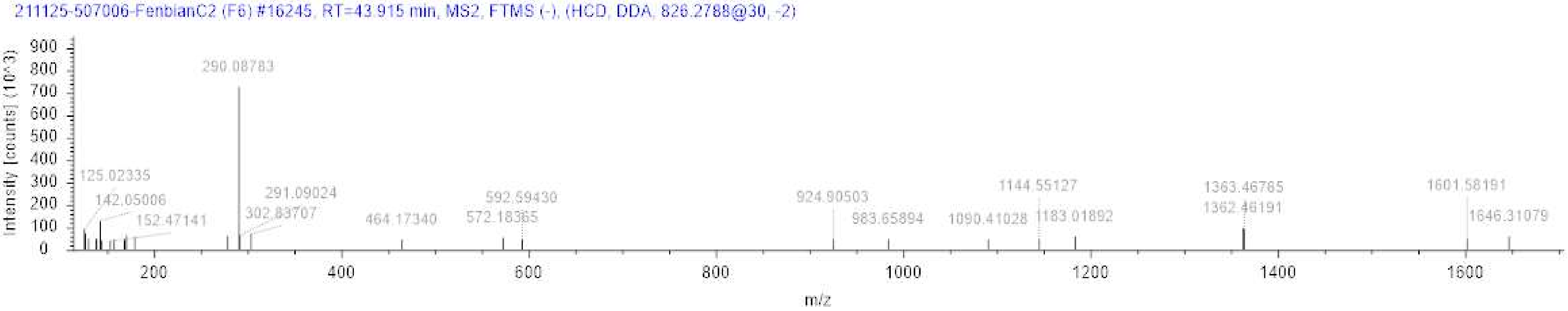

© Reported with Compound Discoverer 3.3

Structure	Name	RT [min]	Formula	Calc. MW	Areas
n/a		43.47	n/a	1654.57215	3.13e7 4.41e7 1.38e7 1.06e8 6.05e8 4.54e8

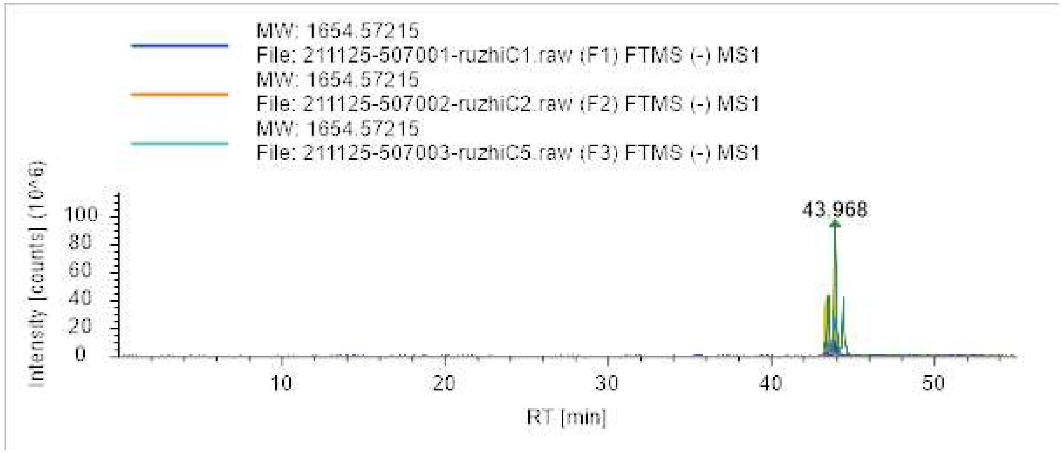

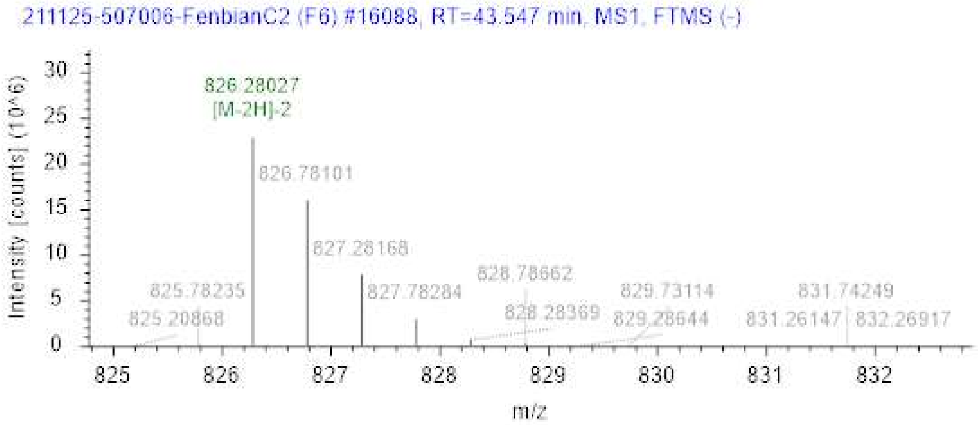

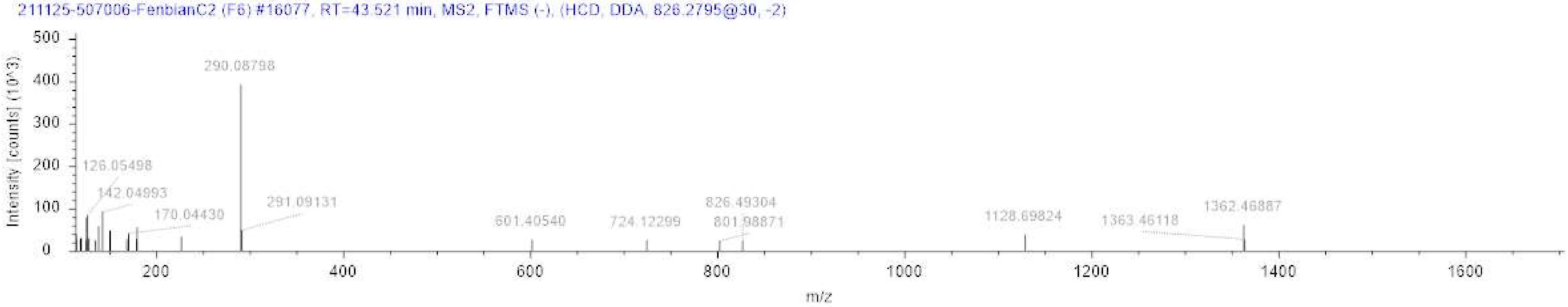

© Reported with Compound Discoverer 3.3

Compounds

Structure	Name	RT [min]	Formula	Calc. MW	Areas
n/a		44.23	n/a	1655.59403	1.45e7 1.70e7 3.76e7 9.12e7 5.26e8

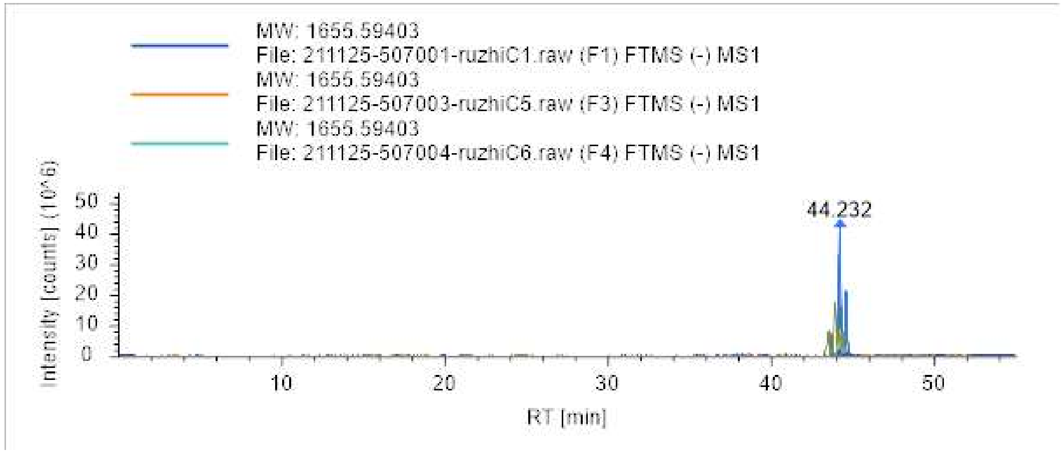

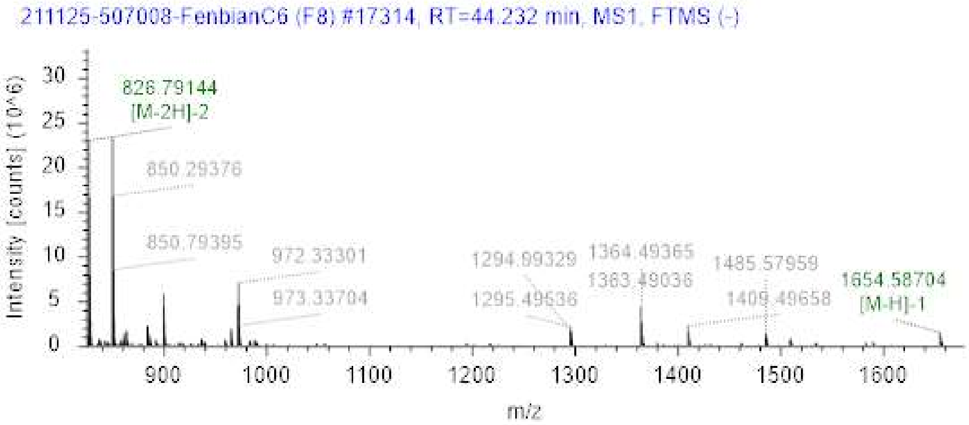

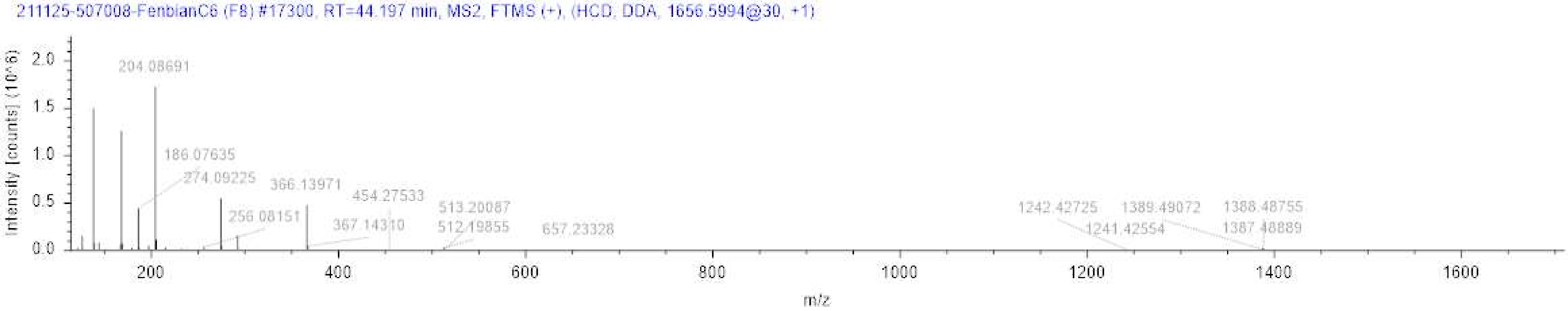

© Reported with Compound Discoverer 3.3

Structure	Name	RT [min]	Formula	Calc. MW	Areas
n/a		43.71	n/a	1657.59866	1.18e7

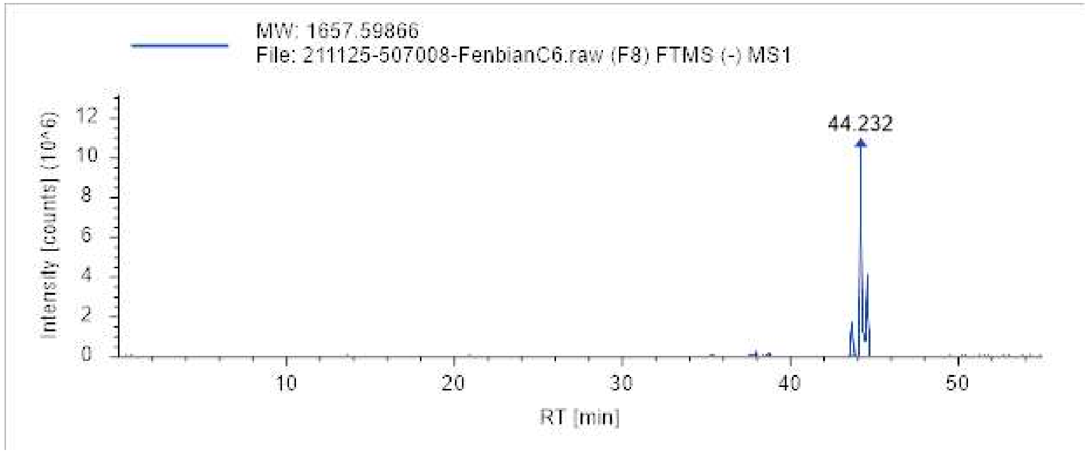

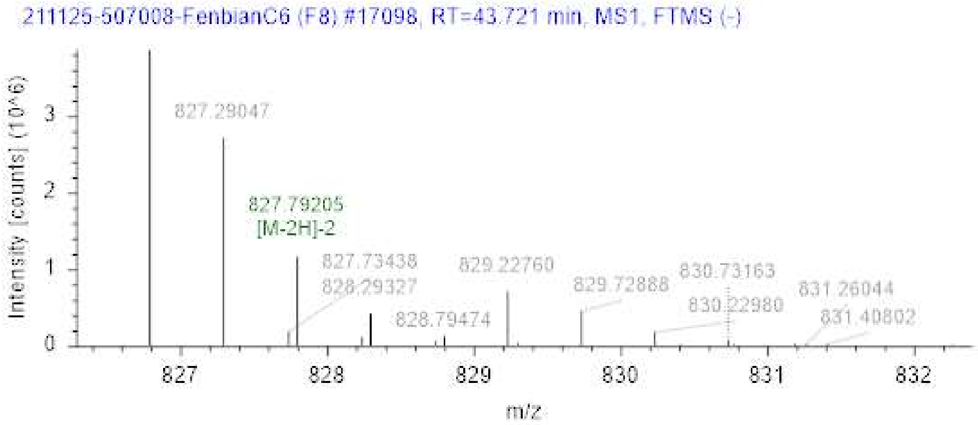

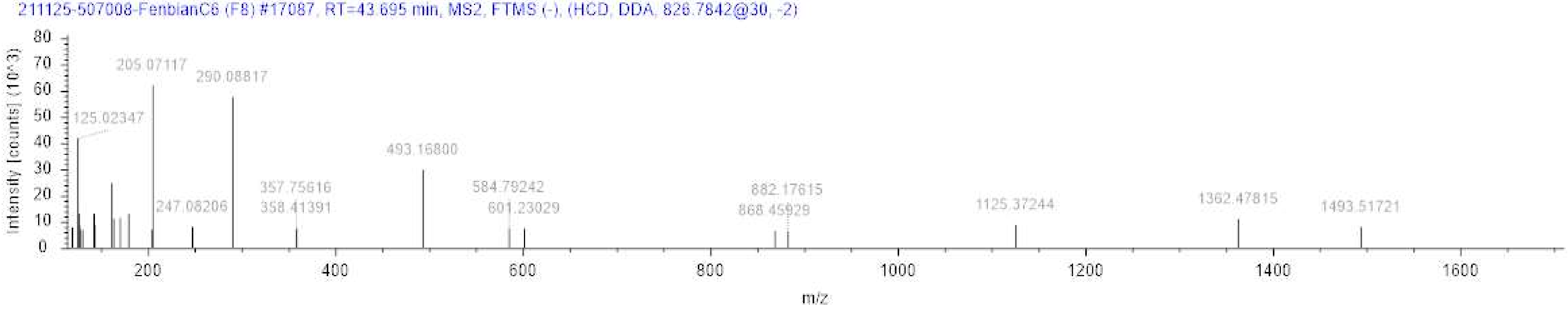

© Reported with Compound Discoverer 3.3

Compounds

Structure	Name	RT [min]	Formula	Calc. MW	Areas
n/a		42.56	n/a	1665.62033	7.18e6

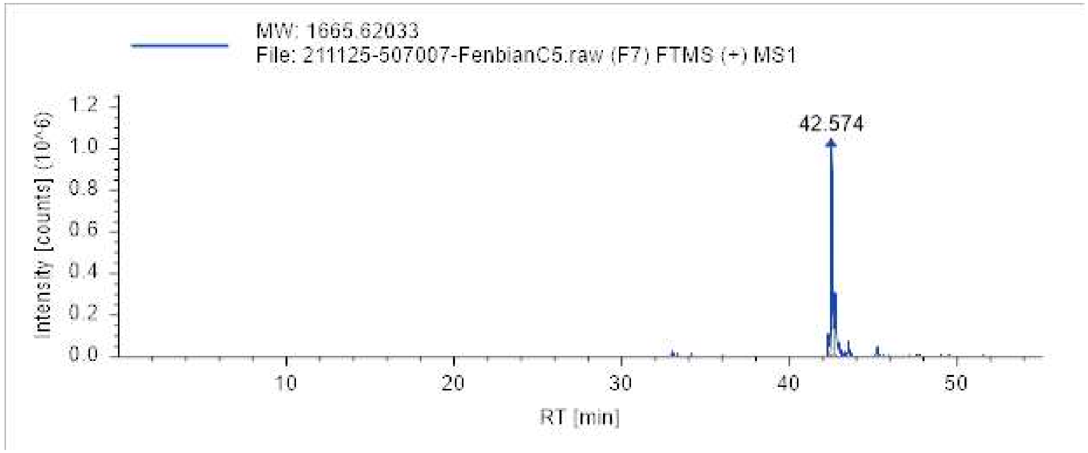

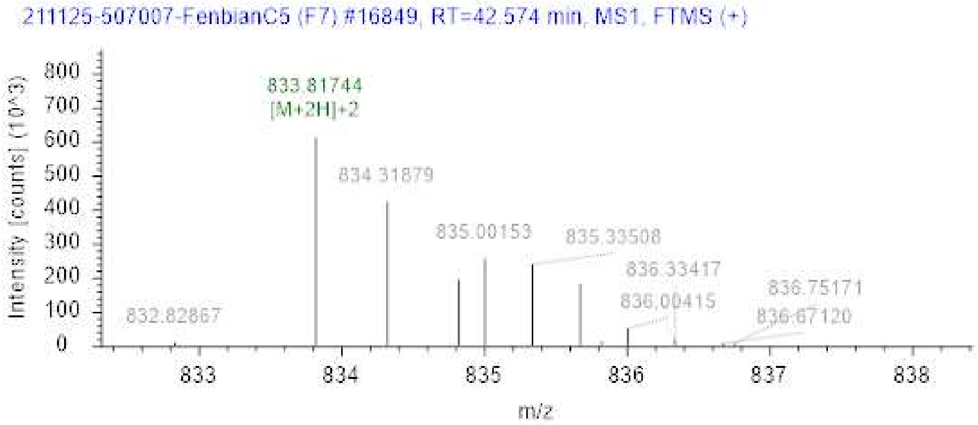

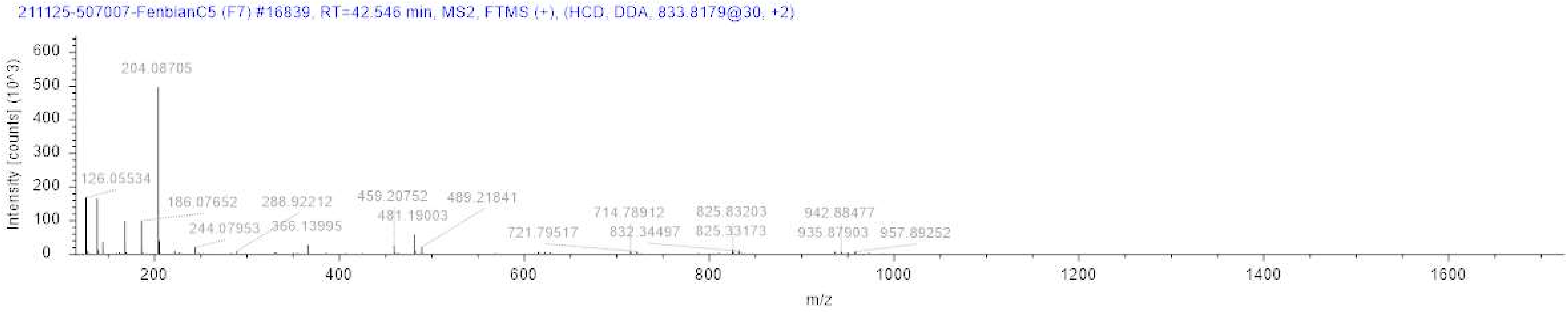

© Reported with Compound Discoverer 3.3

Structure	Name	RT [min]	Formula	Calc. MW	Areas
n/a		45.72	n/a	1681.61899	8.30e7

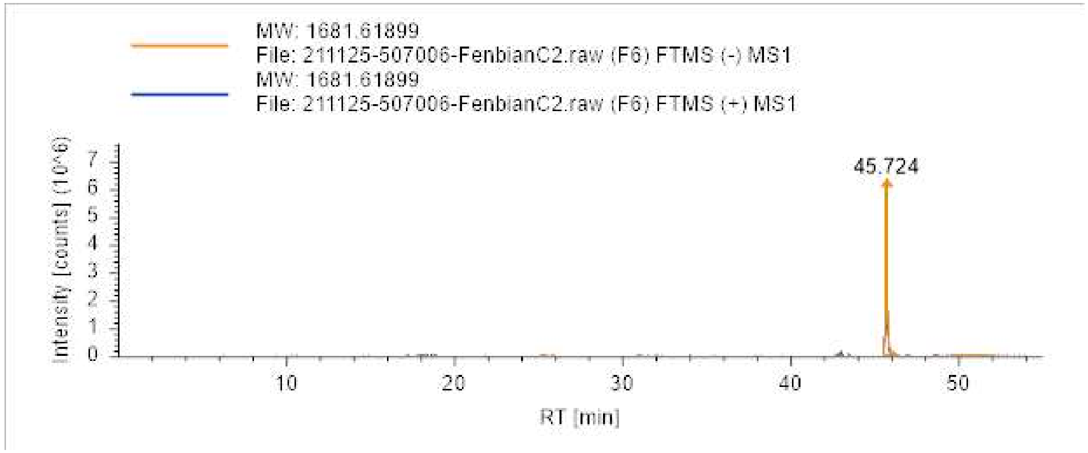

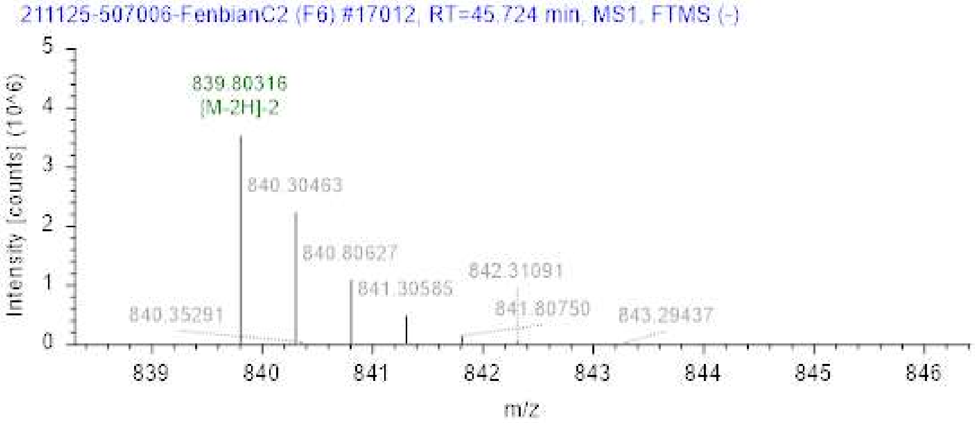

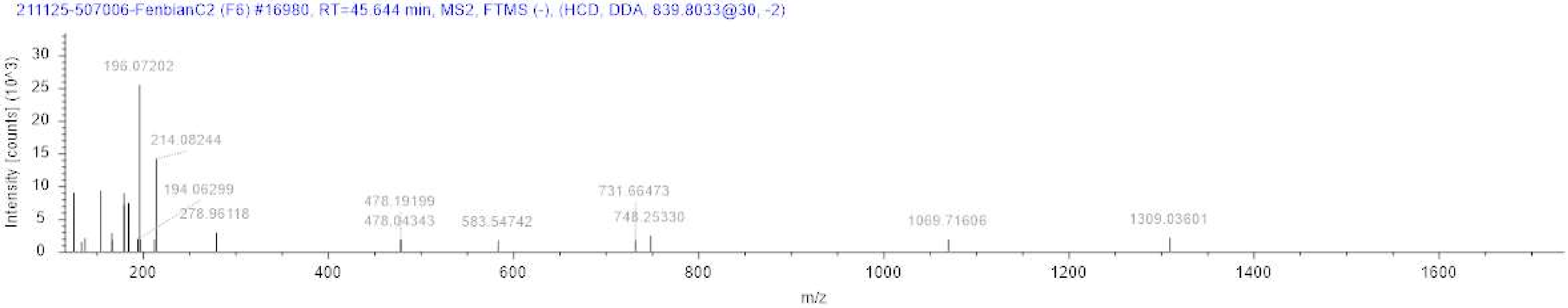

© Reported with Compound Discoverer 3.3

Compounds

Structure	Name	RT [min]	Formula	Calc. MW	Areas
n/a		44.35	n/a	1697.56582	1.23e7

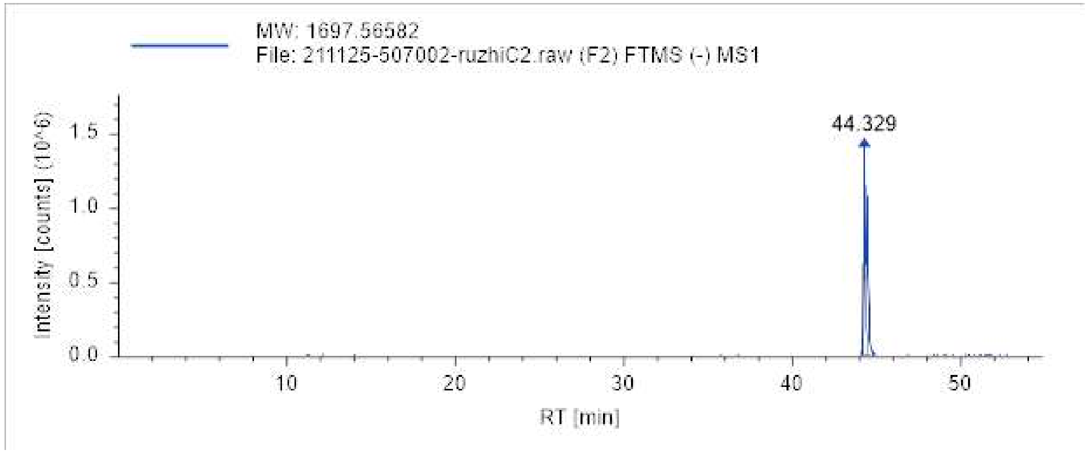

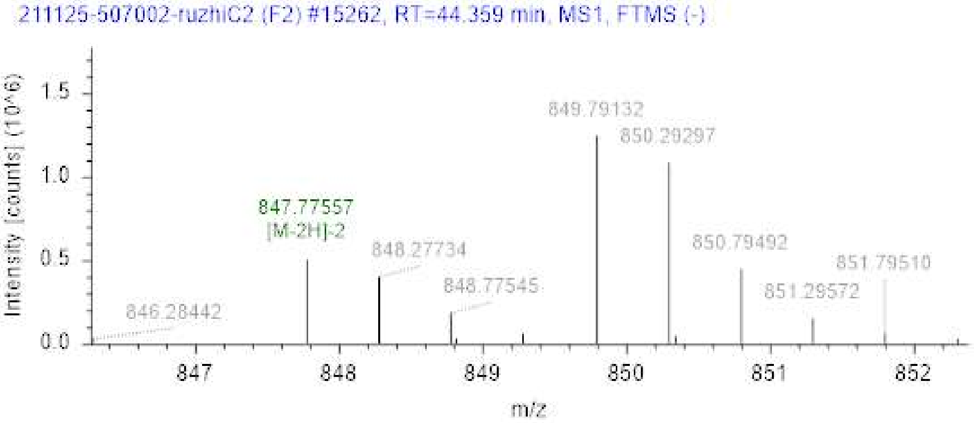

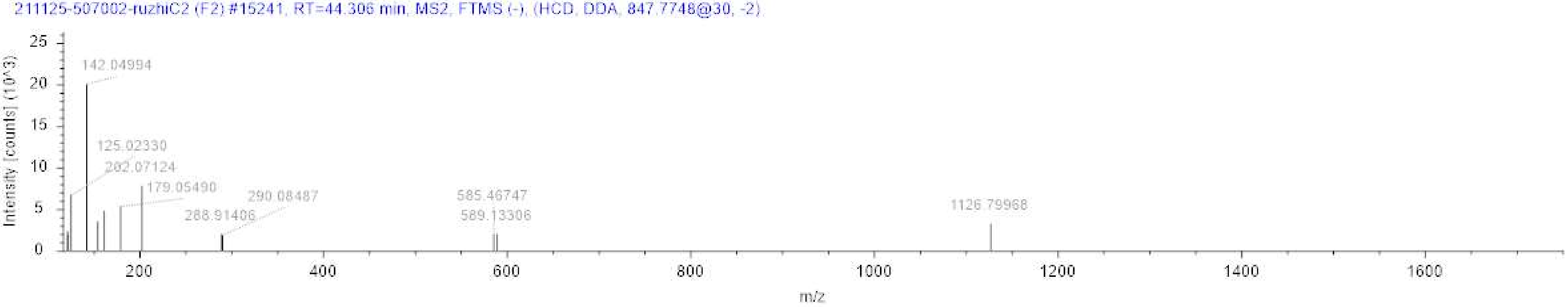

© Reported with Compound Discoverer 3.3

Compounds

Structure	Name	RT [min]	Formula	Calc. MW	Areas
n/a		44.25	n/a	1712.61432	5.36e7

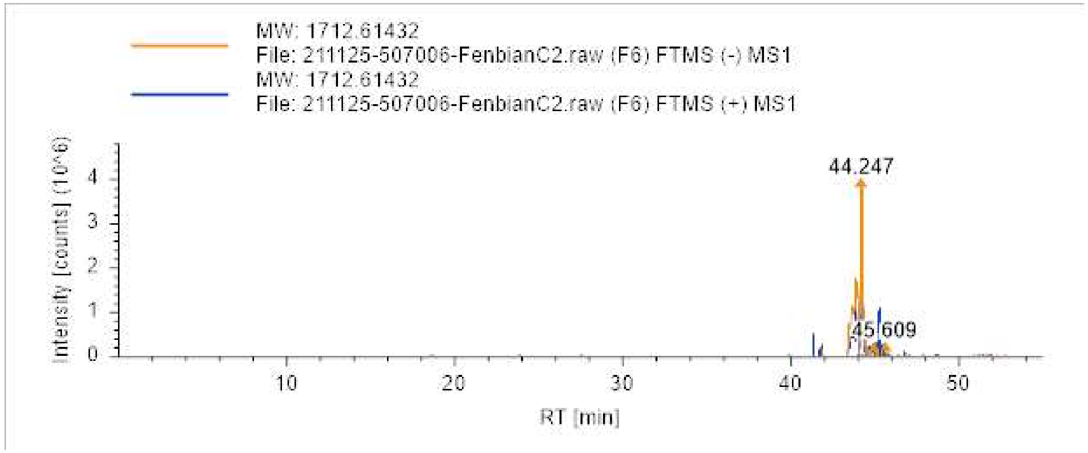

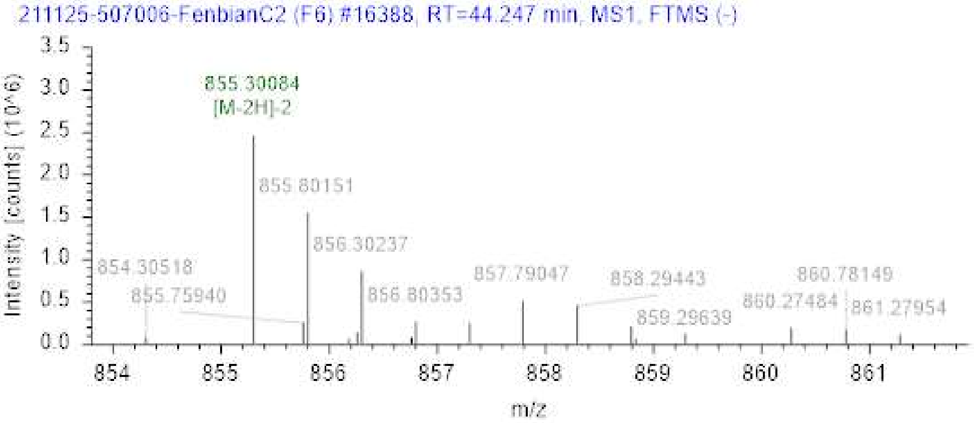

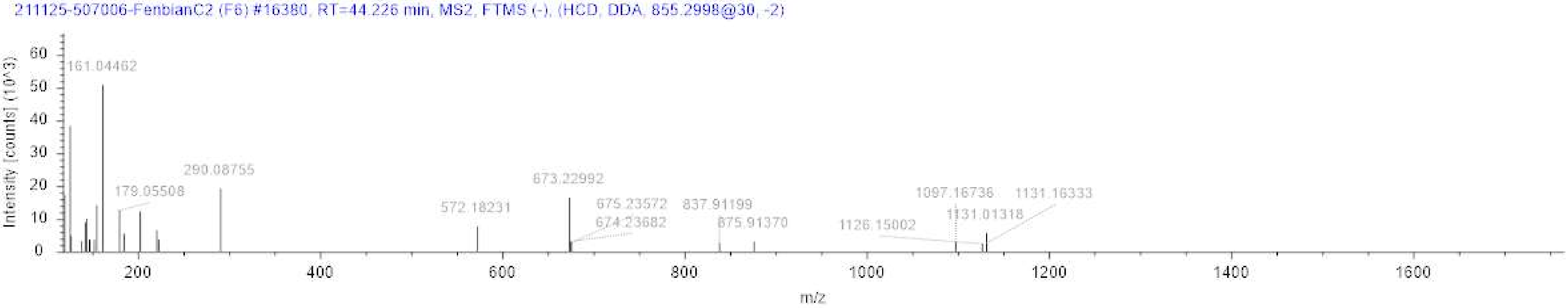

© Reported with Compound Discoverer 3.3

Compounds

Structure	Name	RT [min]	Formula	Calc. MW	Areas
n/a		43.05	n/a	1722.64072	9.90e6

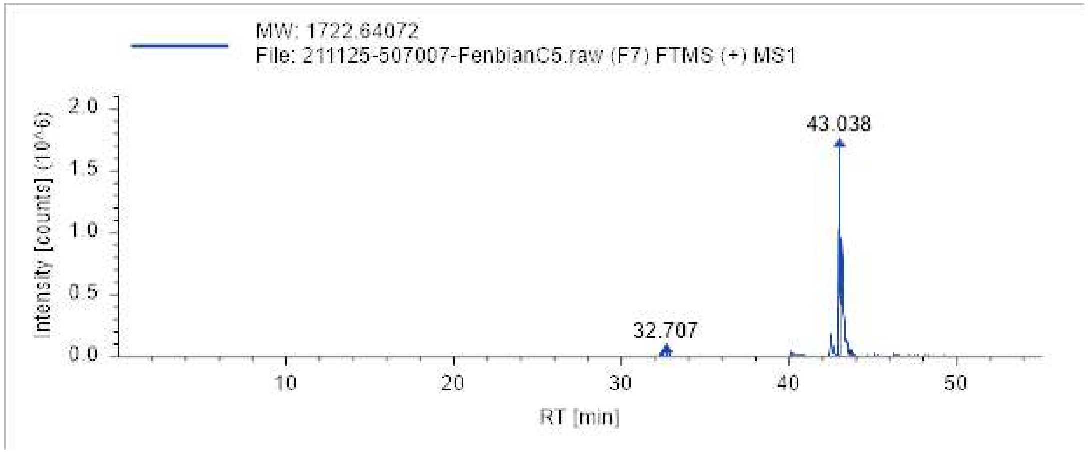

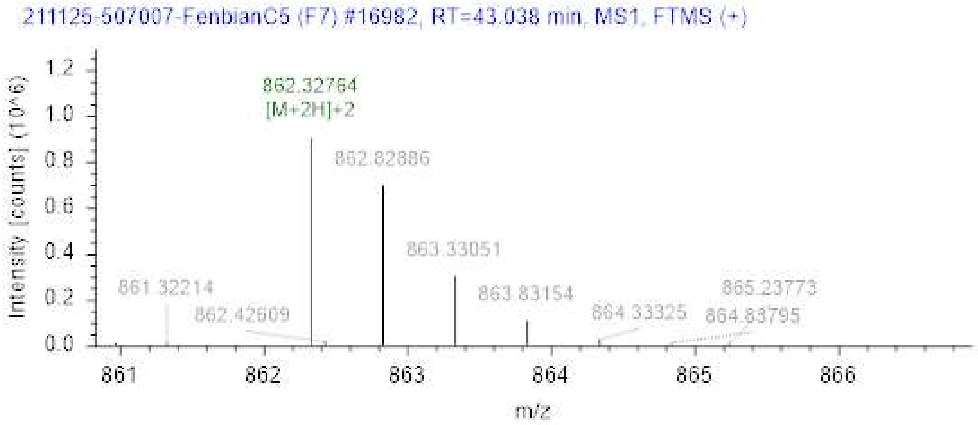

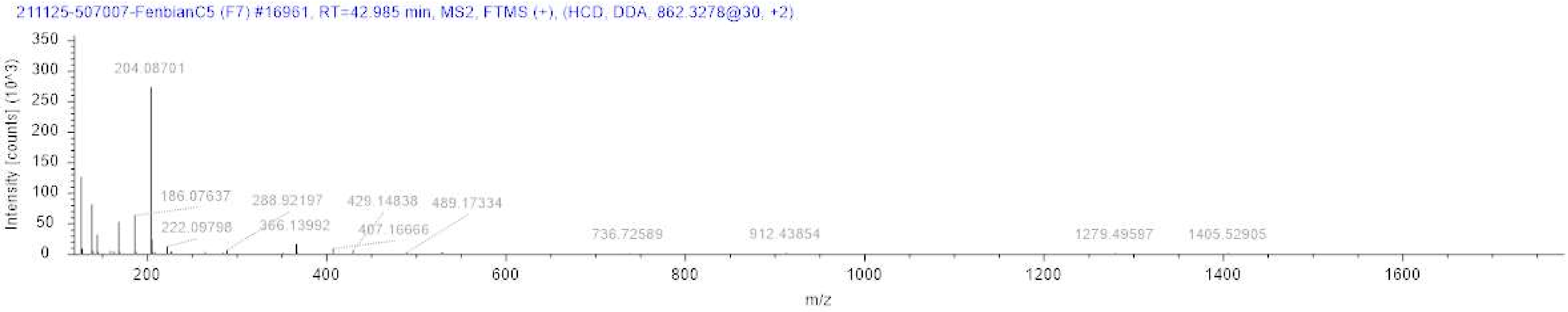

© Reported with Compound Discoverer 3.3

Structure	Name	RT [min]	Formula	Calc. MW	Areas
n/a		44.06	n/a	1728.60483	3.01e7

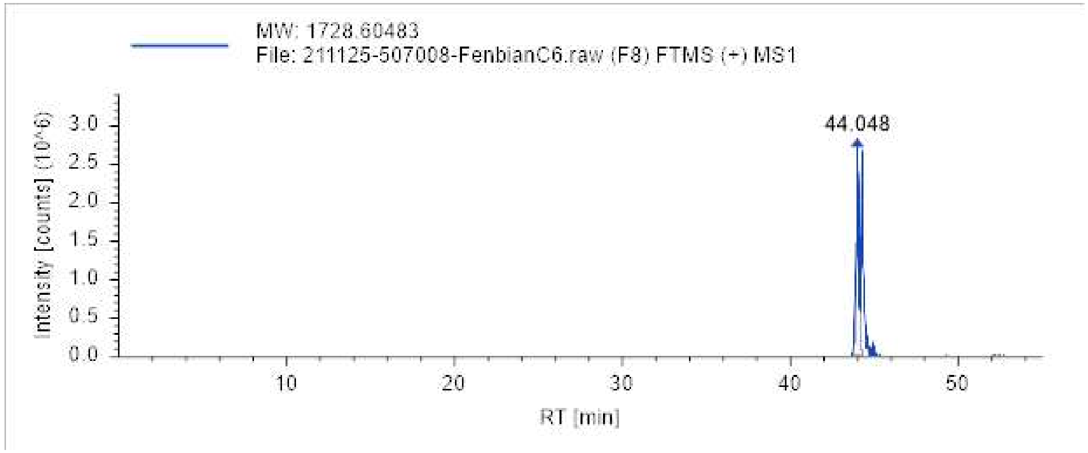

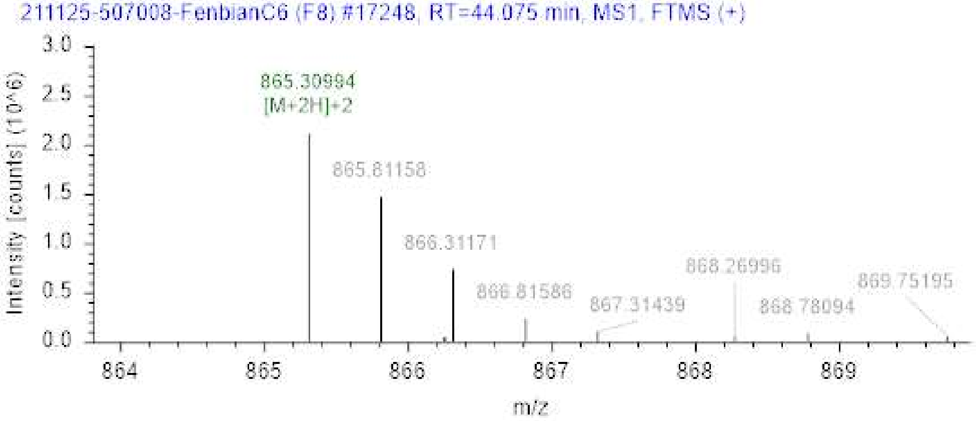

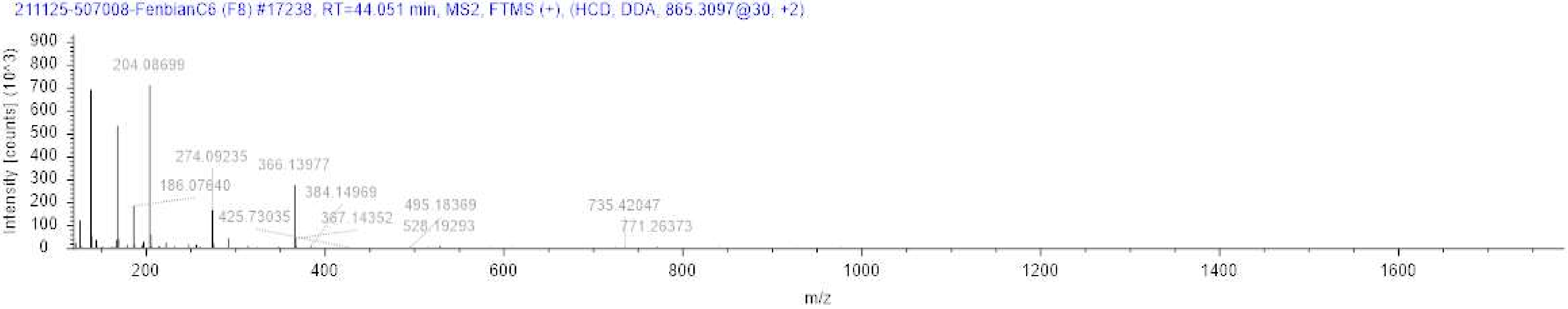

© Reported with Compound Discoverer 3.3

Structure	Name	RT [min]	Formula	Calc. MW	Areas
n/a		44.30	n/a	1728.60913	1.25e7 1.49e7 8.97e6 1.83e7 2.29e8 2.44e8

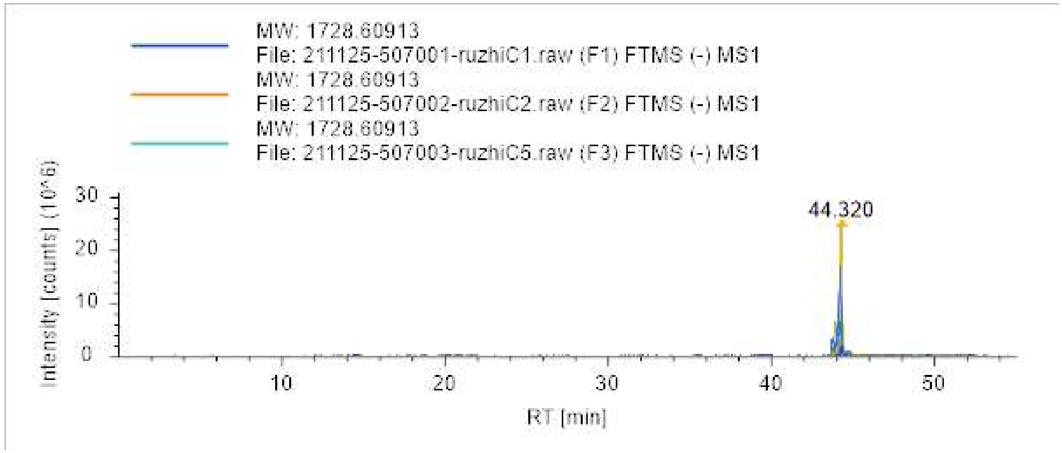

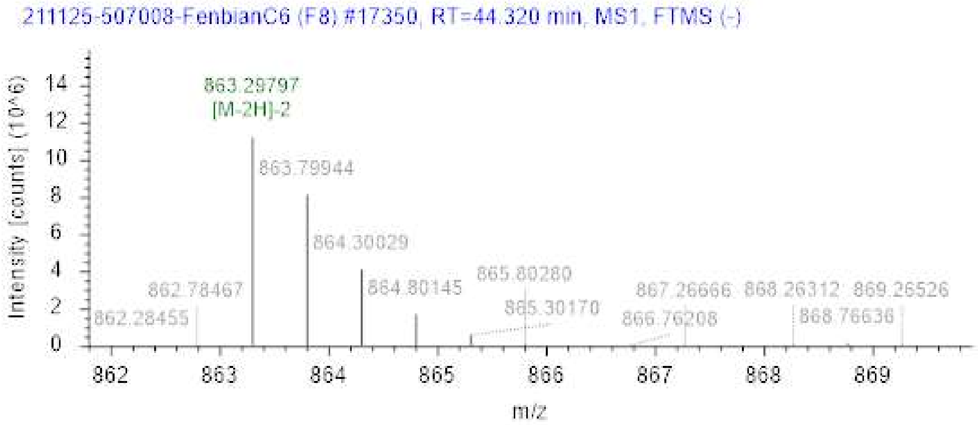

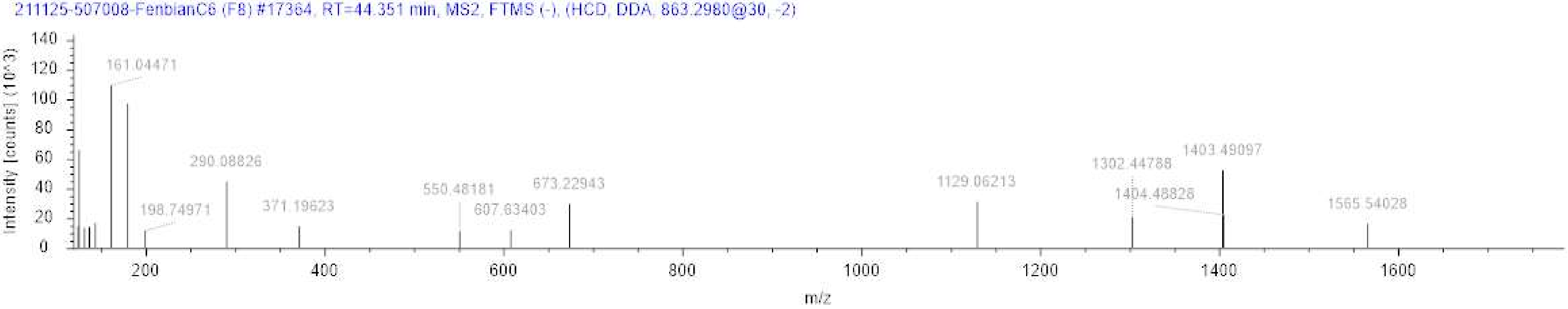

© Reported with Compound Discoverer 3.3

Compounds

Structure	Name	RT [min]	Formula	Calc. MW	Areas
n/a		46.05	n/a	1729.62656	2.43e7

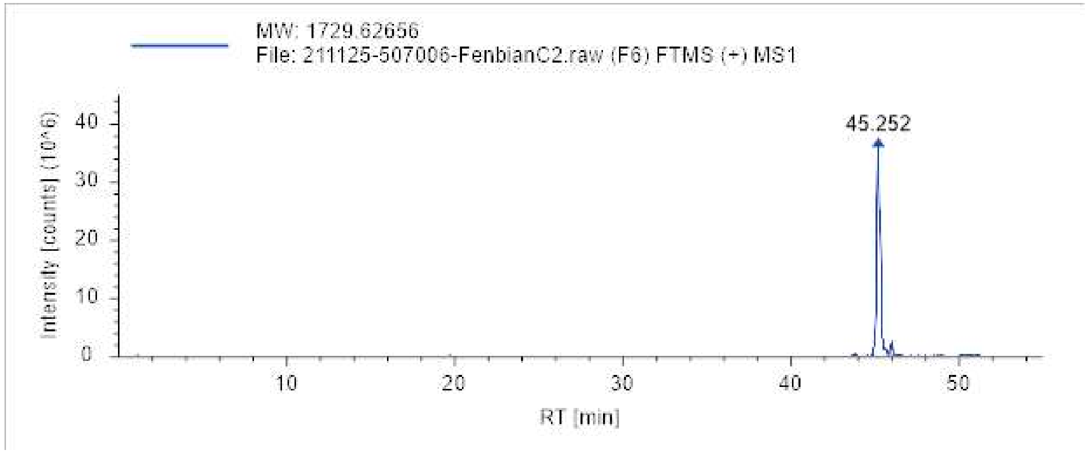

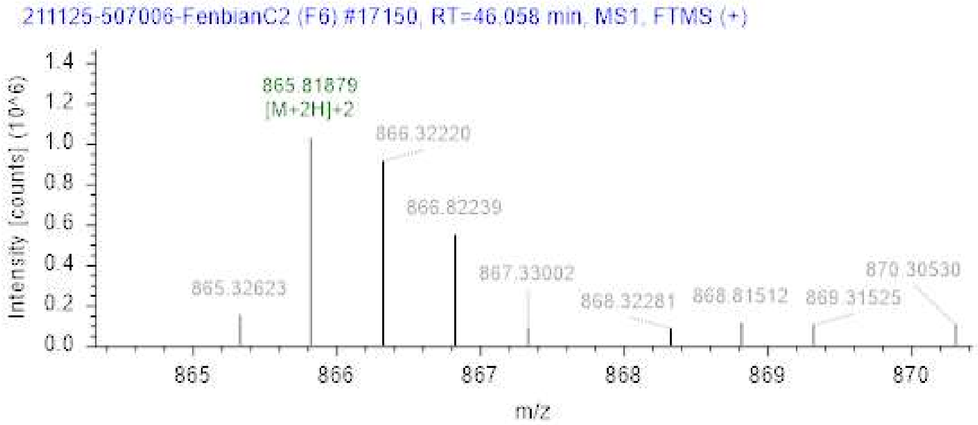

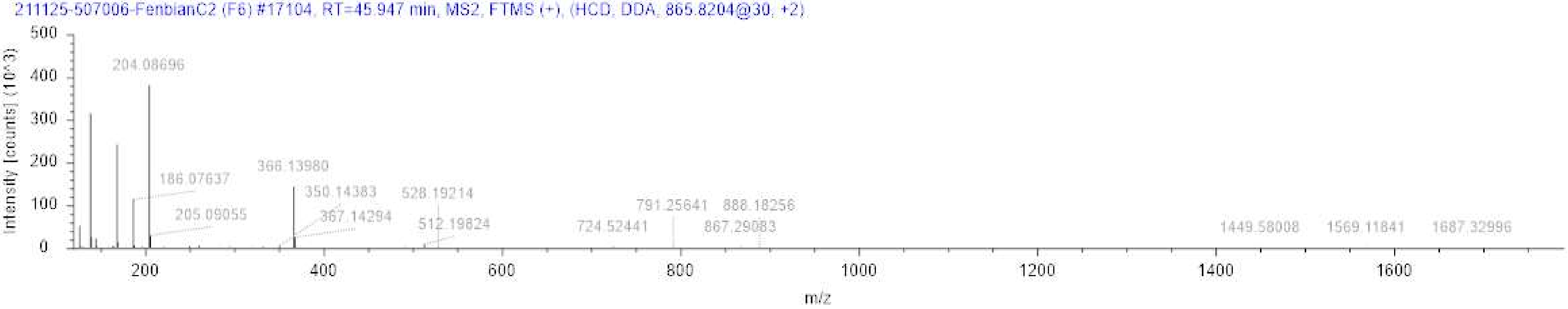

© Reported with Compound Discoverer 3.3

Structure	Name	RT [min]	Formula	Calc. MW	Areas
n/a		46.76	n/a	1729.62834	1.38e7

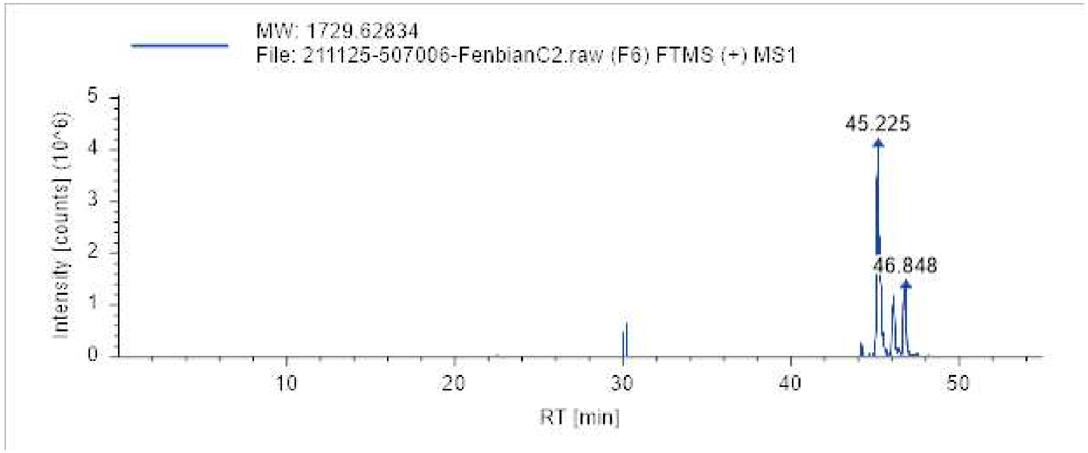

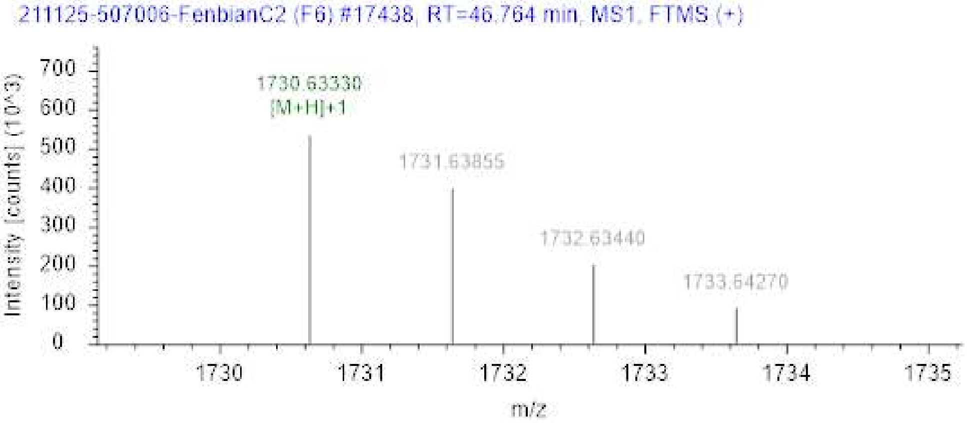

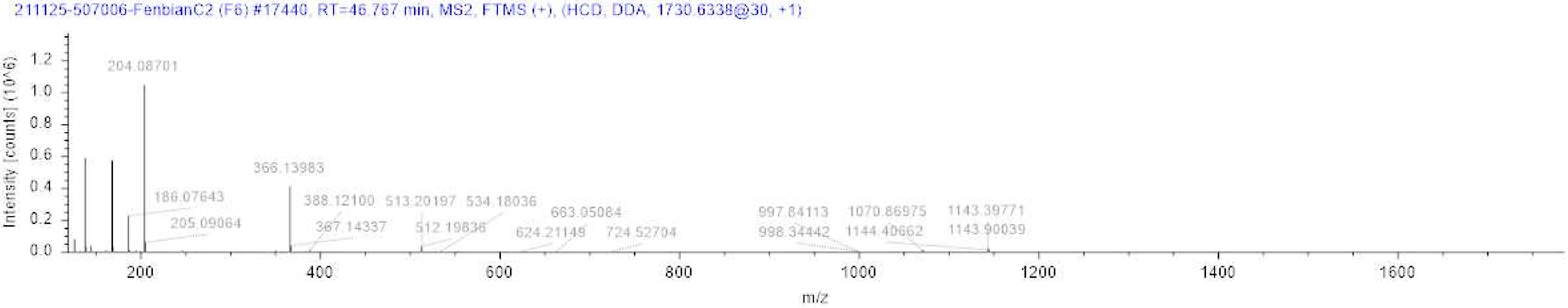

© Reported with Compound Discoverer 3.3

Compounds

Structure	Name	RT [min]	Formula	Calc. MW	Areas
n/a		45.21	n/a	1729.63153	1.21e8 2.92e8 9.39e7 5.28e7 2.38e9 5.24e8

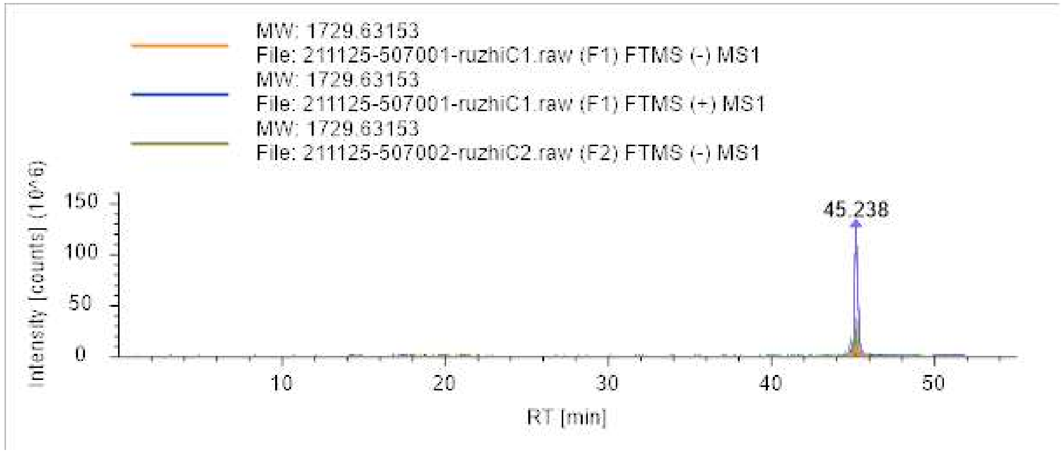

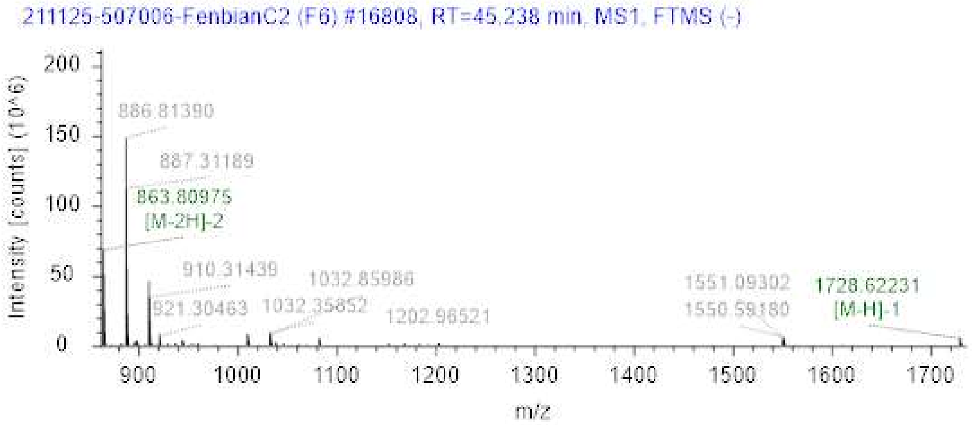

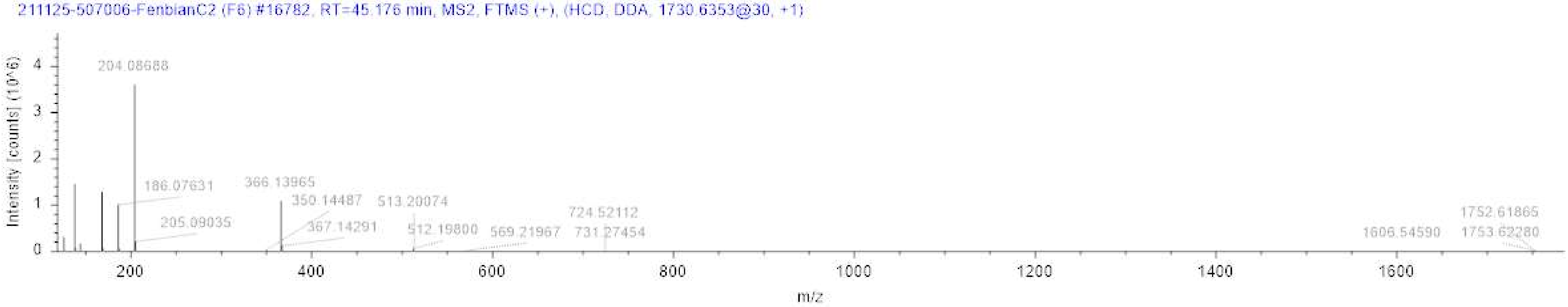

© Reported with Compound Discoverer 3.3

Structure	Name	RT [min]	Formula	Calc. MW	Areas
n/a		44.28	n/a	1745.63207	1.38e7 1.14e7

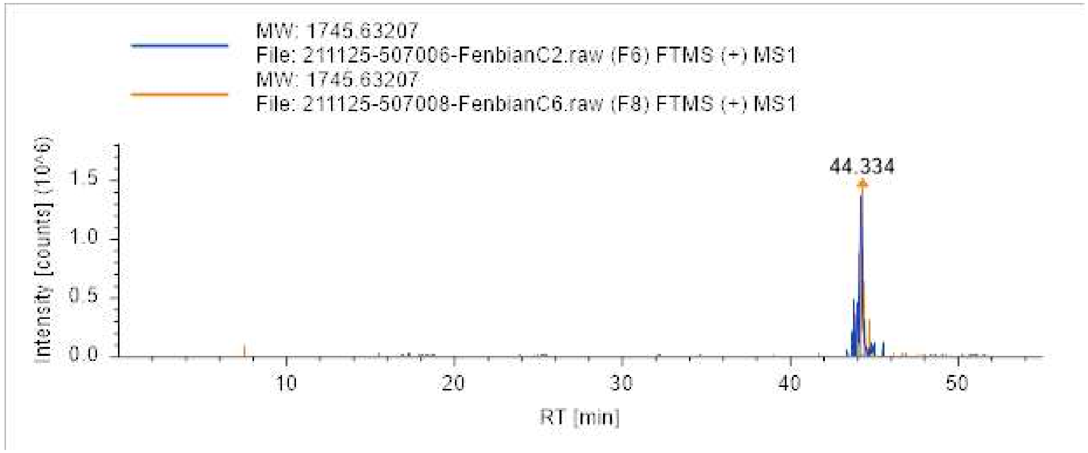

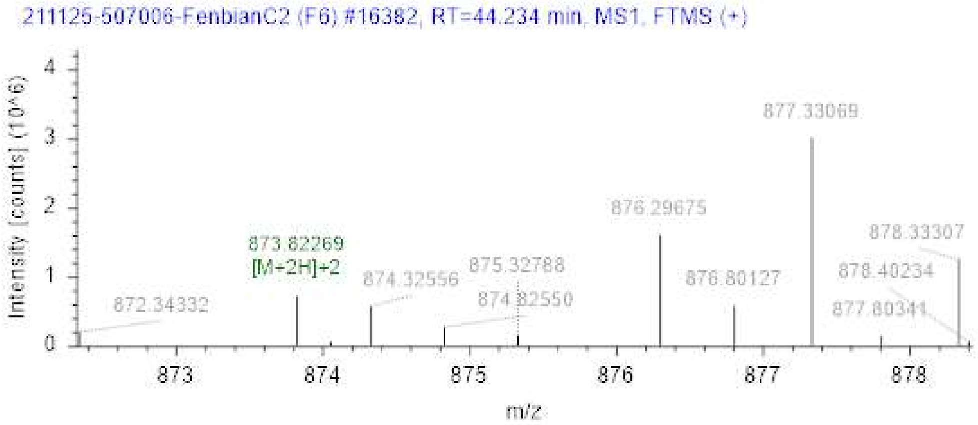

© Reported with Compound Discoverer 3.3

Compounds

Structure	Name	RT [min]	Formula	Calc. MW	Areas
n/a		43.60	n/a	1769.63377	3.99e7

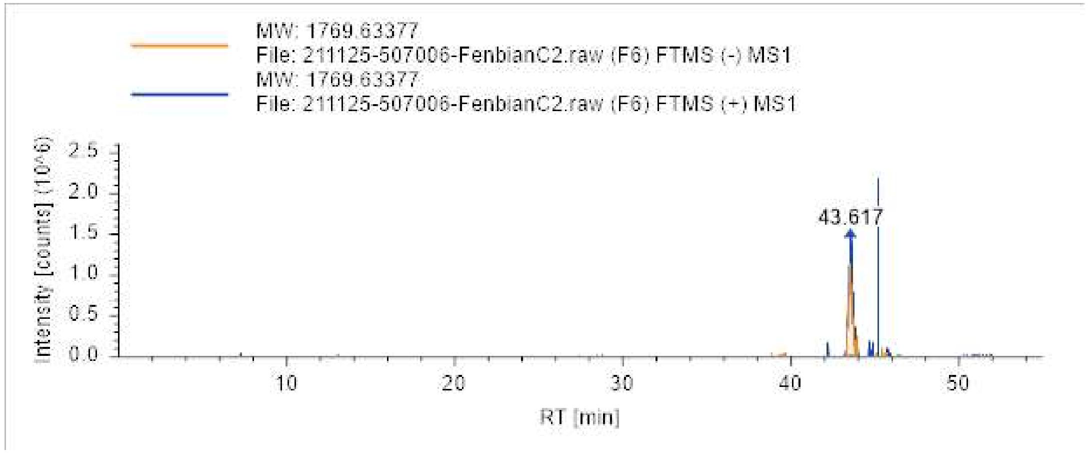

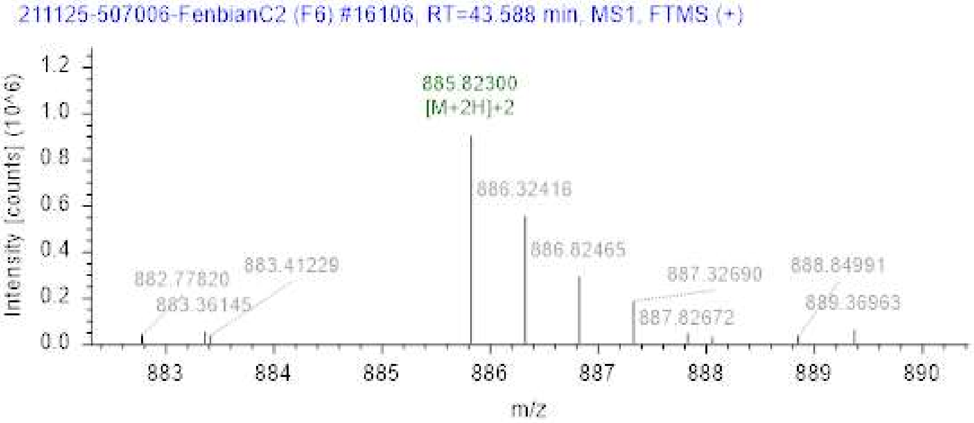

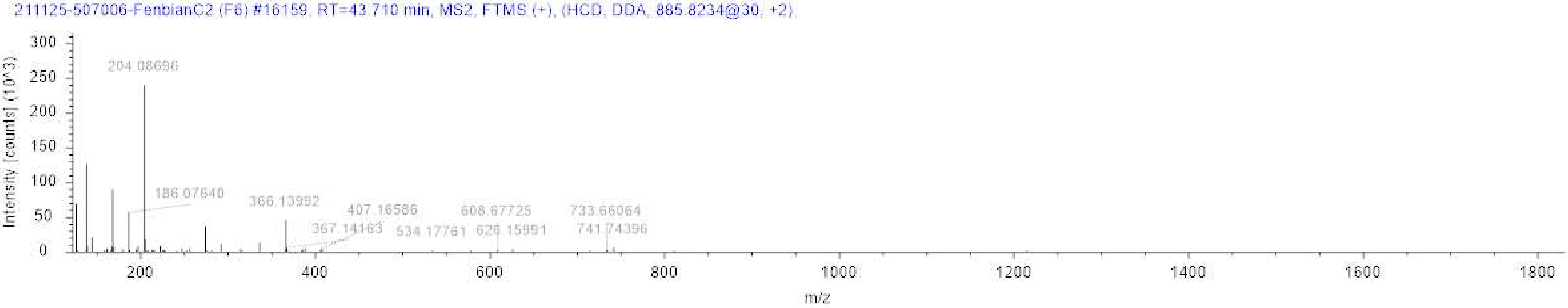

© Reported with Compound Discoverer 3.3

Structure	Name	RT [min]	Formula	Calc. MW	Areas
n/a		45.27	n/a	1770.65055	7.07e6; 3.99e7

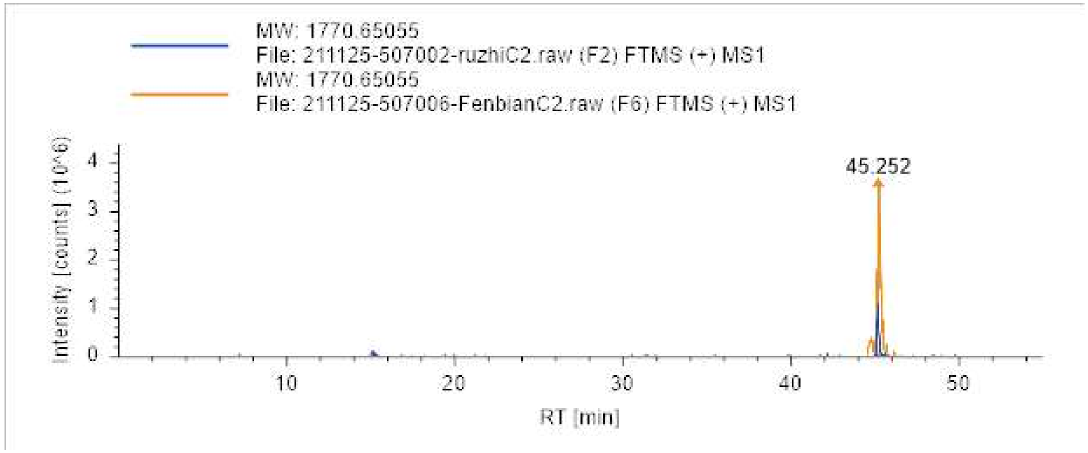

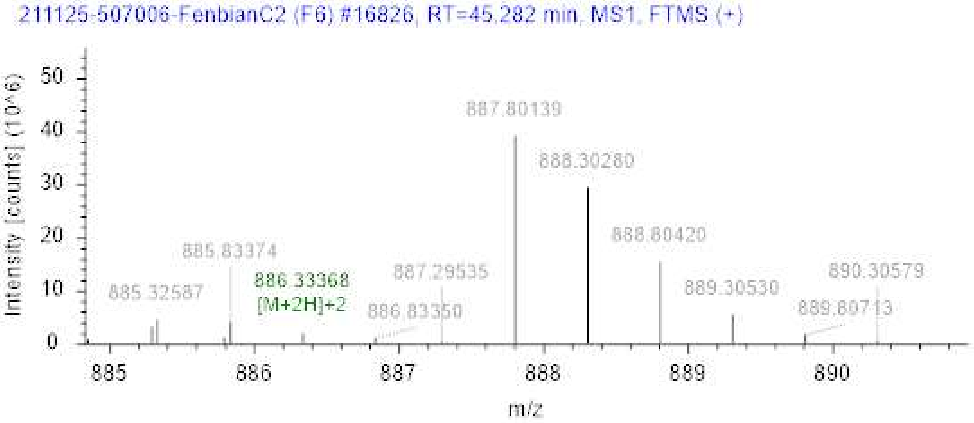

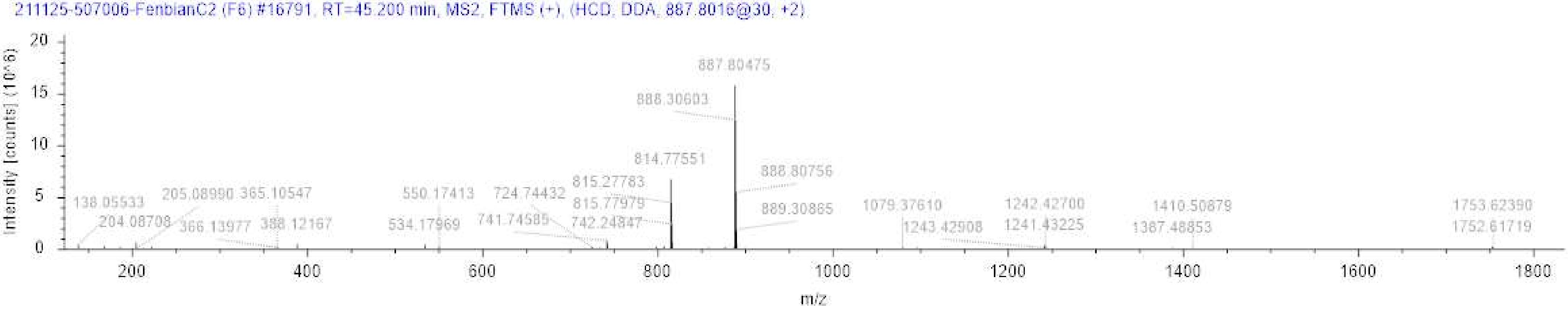

© Reported with Compound Discoverer 3.3

Compounds

Structure	Name	RT [min]	Formula	Calc. MW	Areas
n/a		44.81	n/a	1786.65066	4.07e8; 3.75e7

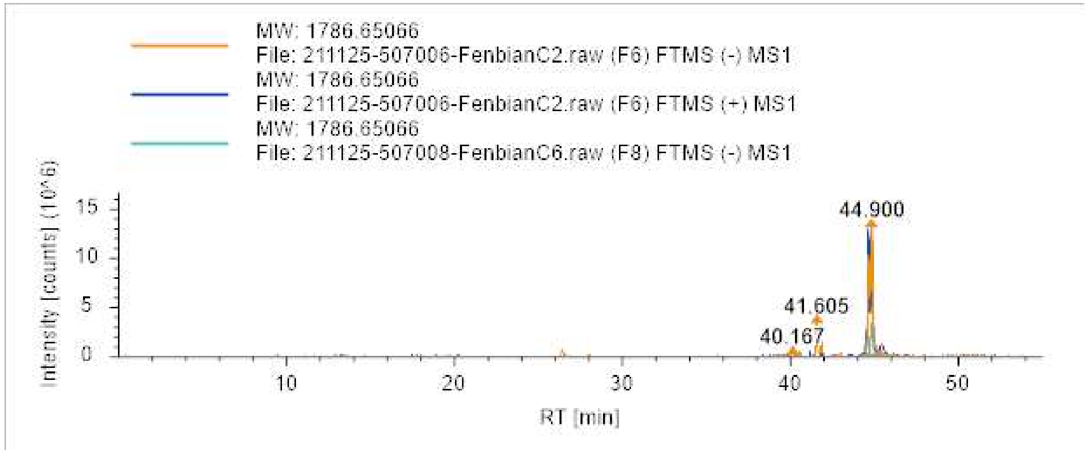

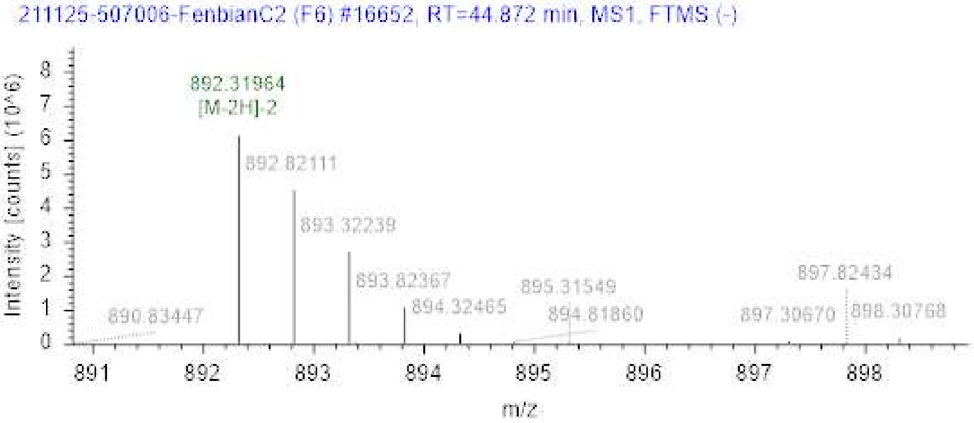

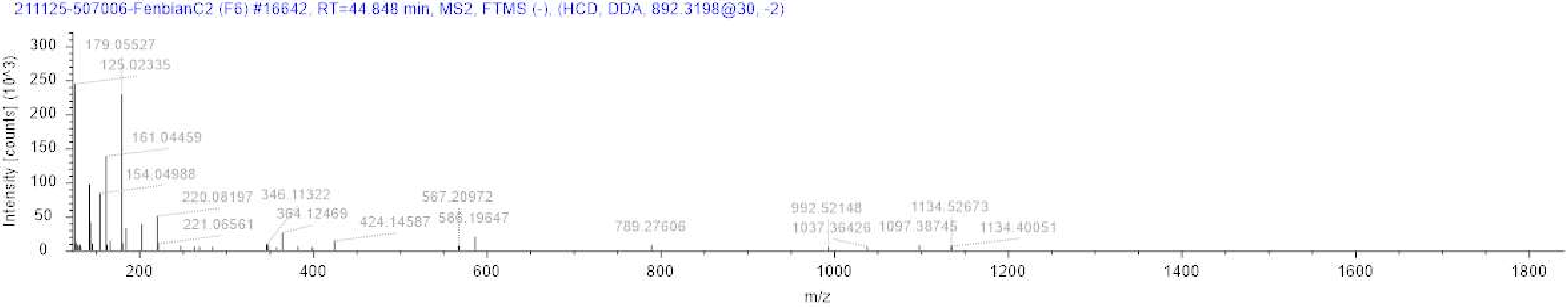

© Reported with Compound Discoverer 3.3

Compounds

Structure	Name	RT [min]	Formula	Calc. MW	Areas
n/a		45.19	n/a	1791.56015	2.71e7

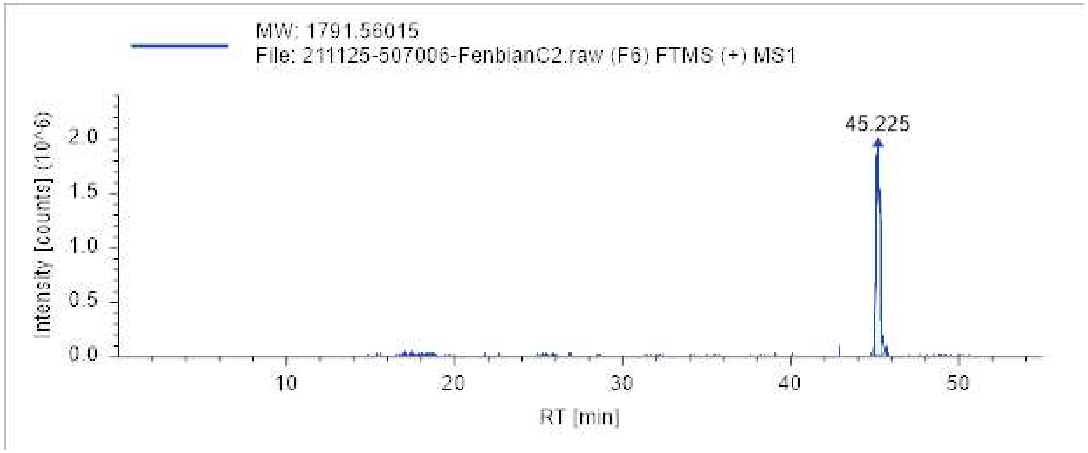

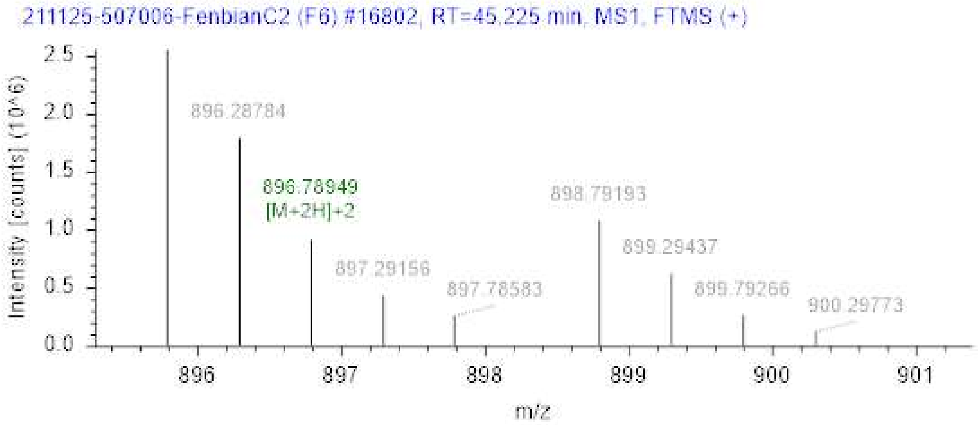

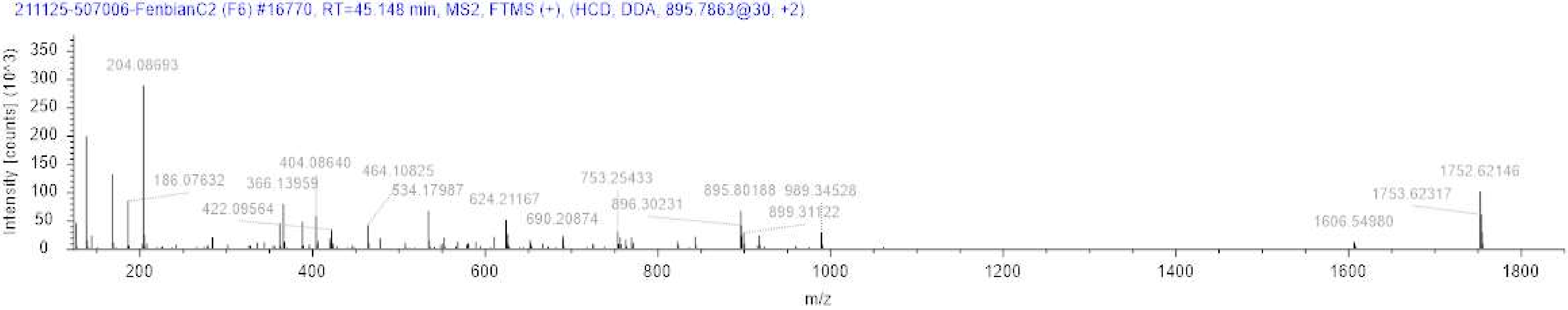

© Reported with Compound Discoverer 3.3

Compounds

Structure	Name	RT [min]	Formula	Calc. MW	Areas
n/a		44.79	n/a	1802.64280	2.94e7 8.23e7 4.46e7 6.84e7 1.34e8

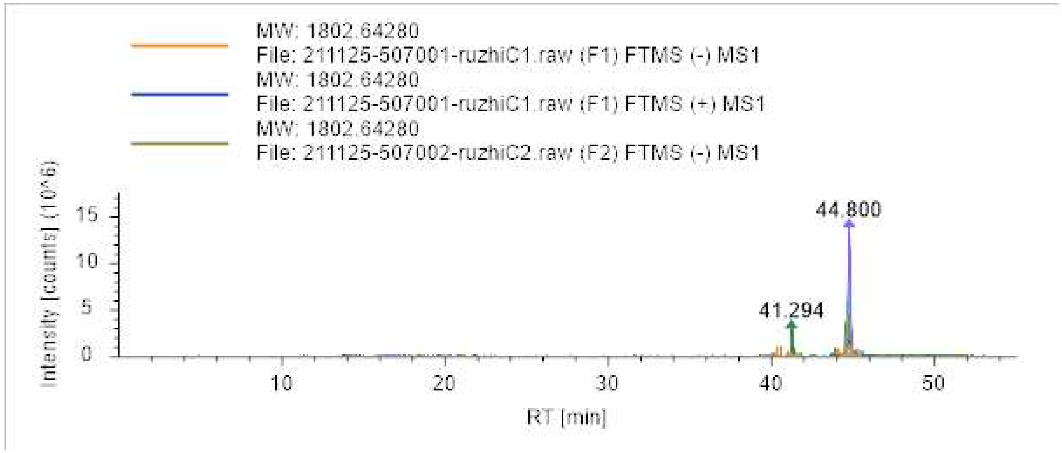

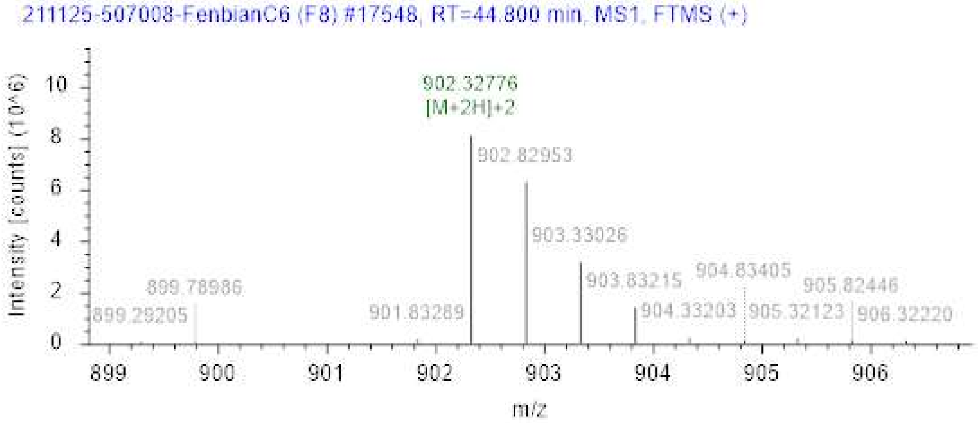

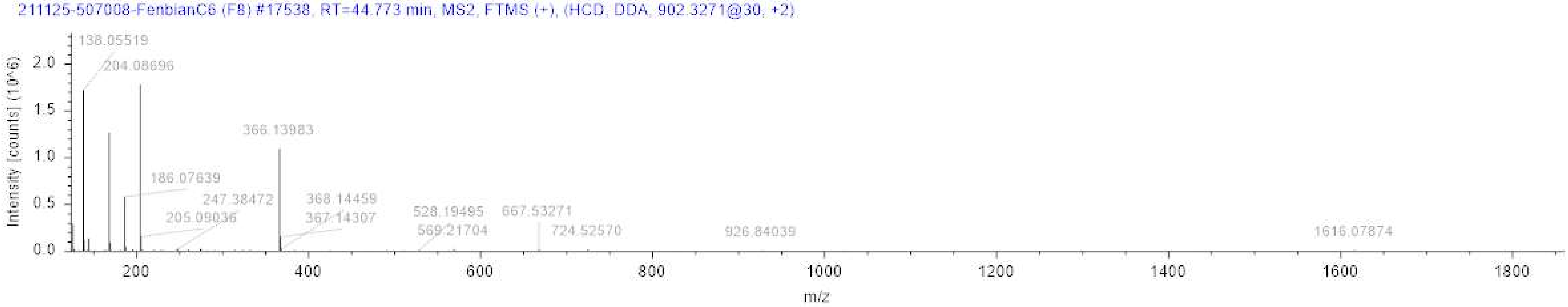

© Reported with Compound Discoverer 3.3

Structure	Name	RT [min]	Formula	Calc. MW	Areas
n/a		44.90	n/a	1803.67246	5.09e7

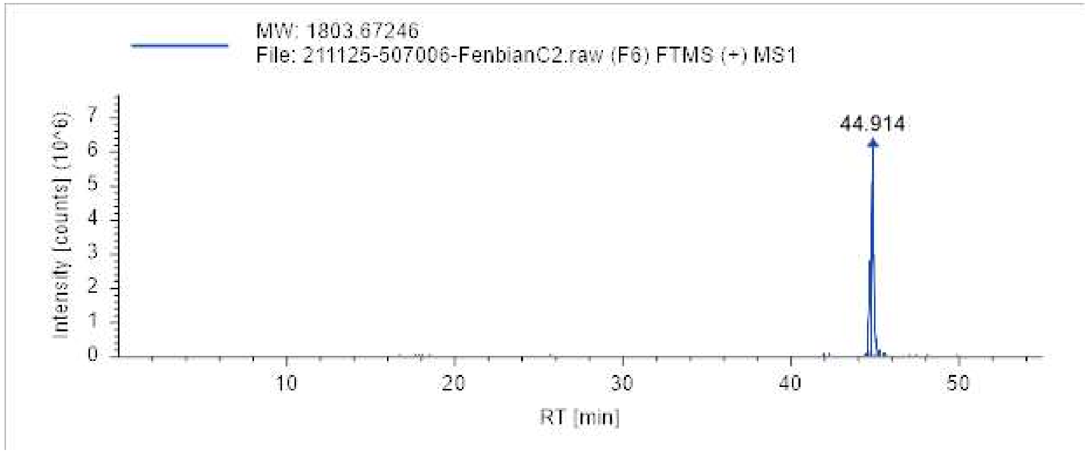

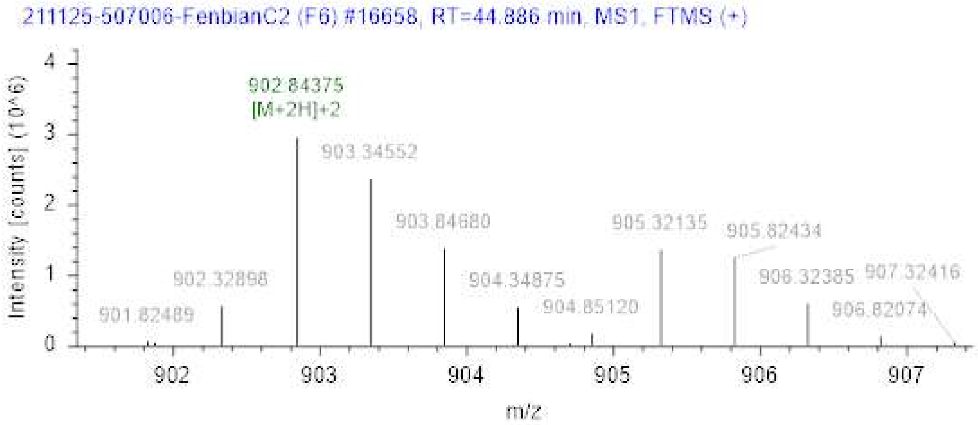

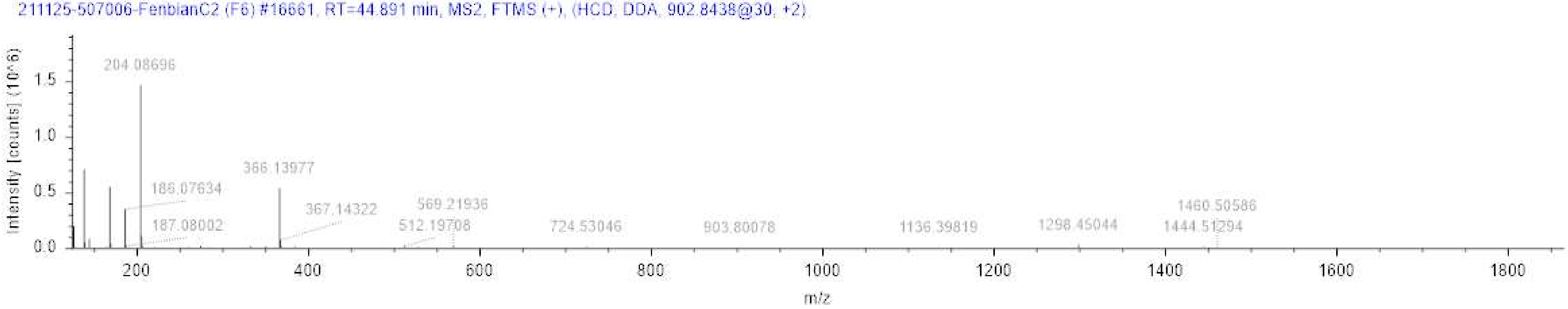

© Reported with Compound Discoverer 3.3

Compounds

Structure	Name	RT [min]	Formula	Calc. MW	Areas
n/a		43.81	n/a	1857.65038	5.02e7

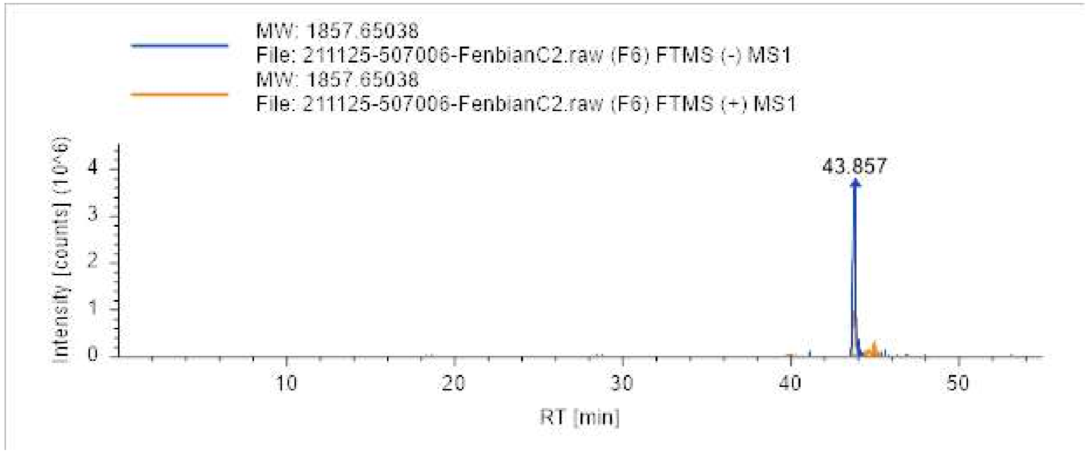

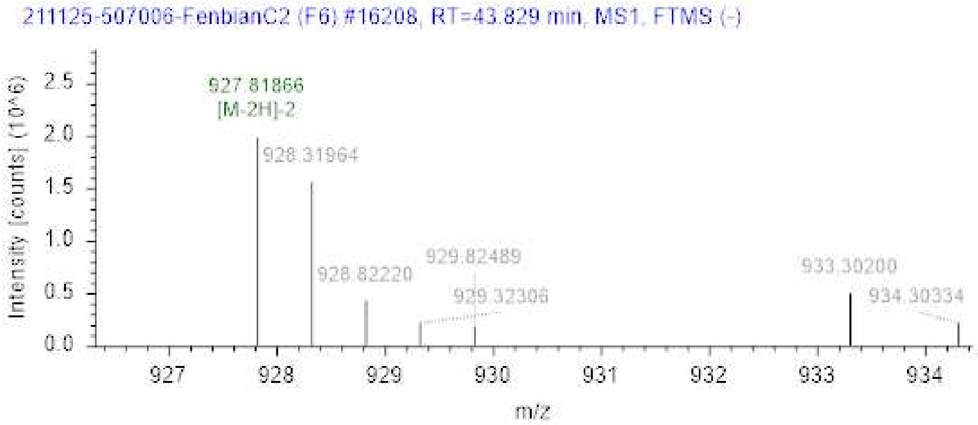

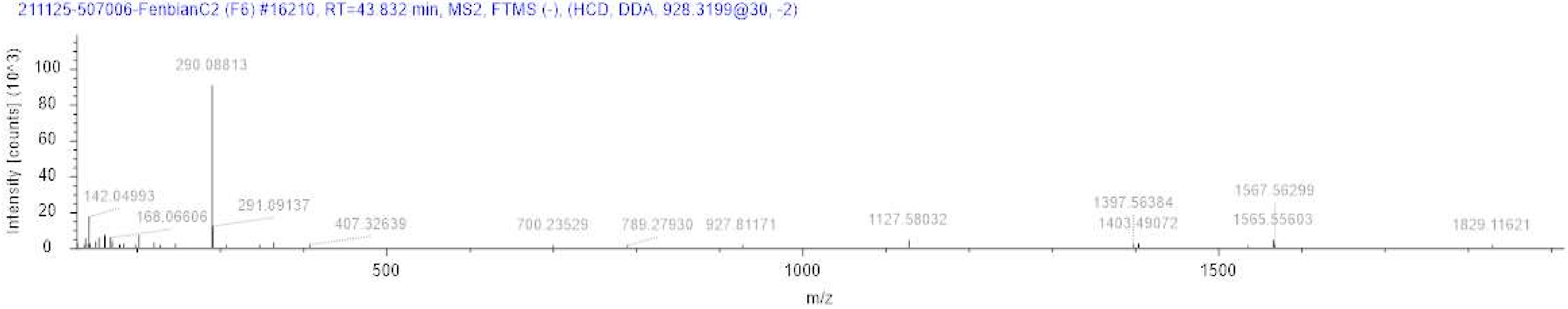

© Reported with Compound Discoverer 3.3

Structure	Name	RT [min]	Formula	Calc. MW	Areas
n/a		46.05	n/a	1861.67715	1.18e7

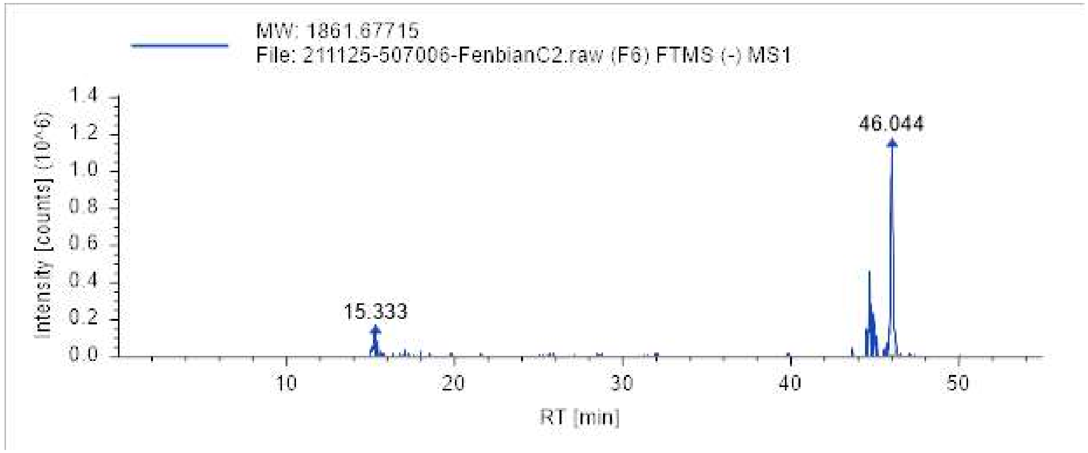

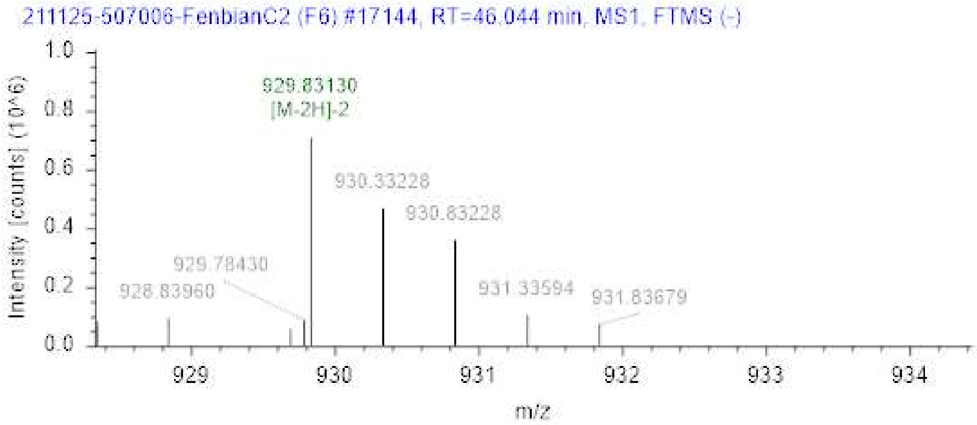

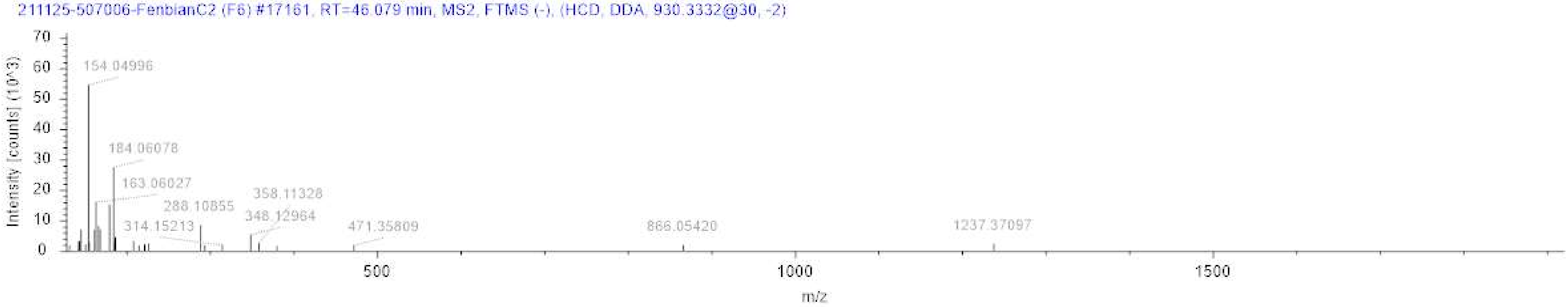

© Reported with Compound Discoverer 3.3

Compounds

Structure	Name	RT [min]	Formula	Calc. MW	Areas
n/a		44.83	n/a	1868.71035	4.72e6

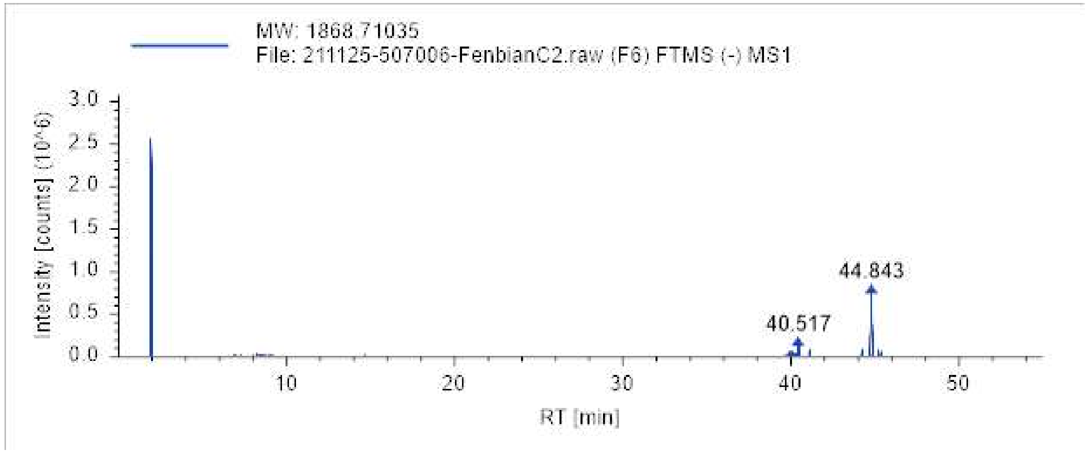

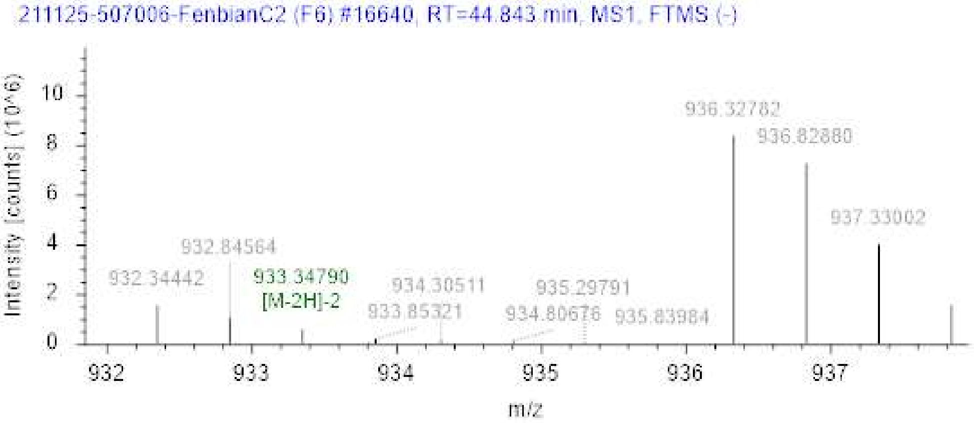

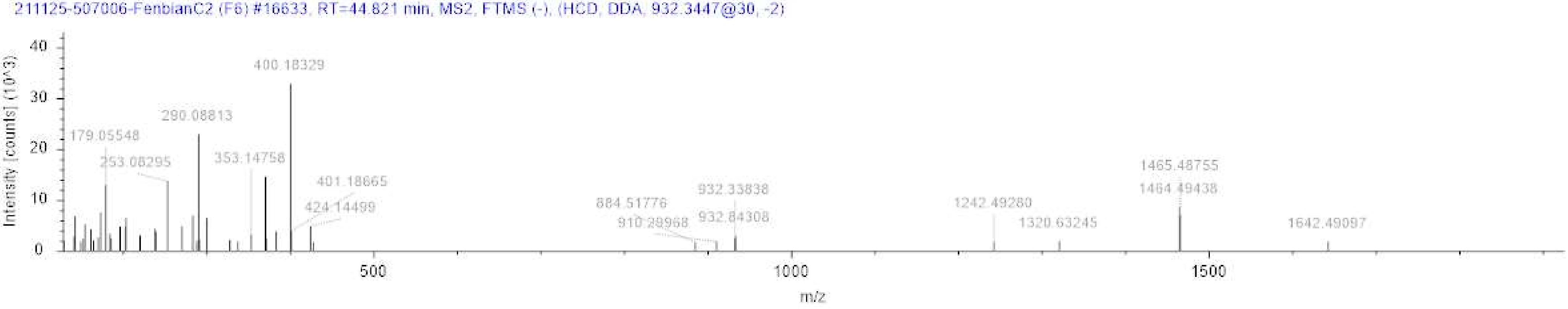

© Reported with Compound Discoverer 3.3

Structure	Name	RT [min]	Formula	Calc. MW	Areas
n/a		44.97	n/a	1874.66852	2.47e7 | 5.30e7 | 1.67e7 | 1.83e7 | | | | 2.46e8 |

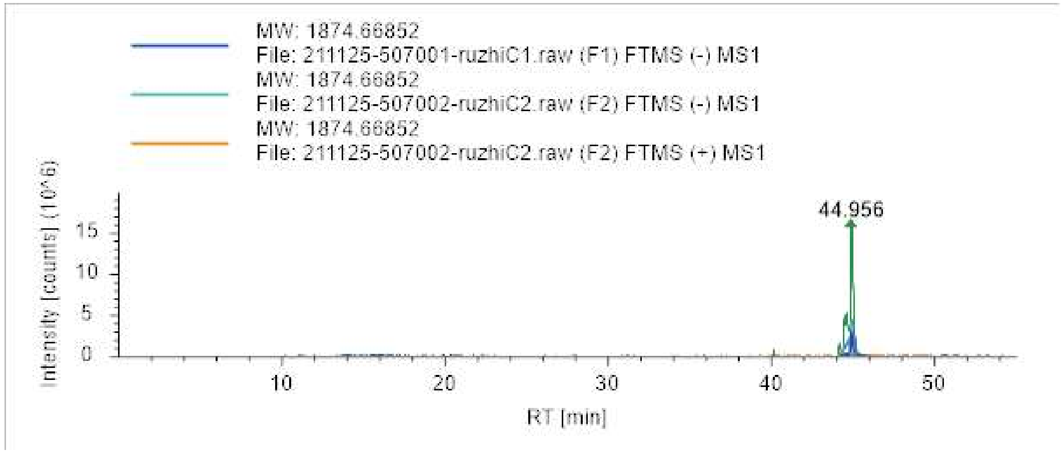

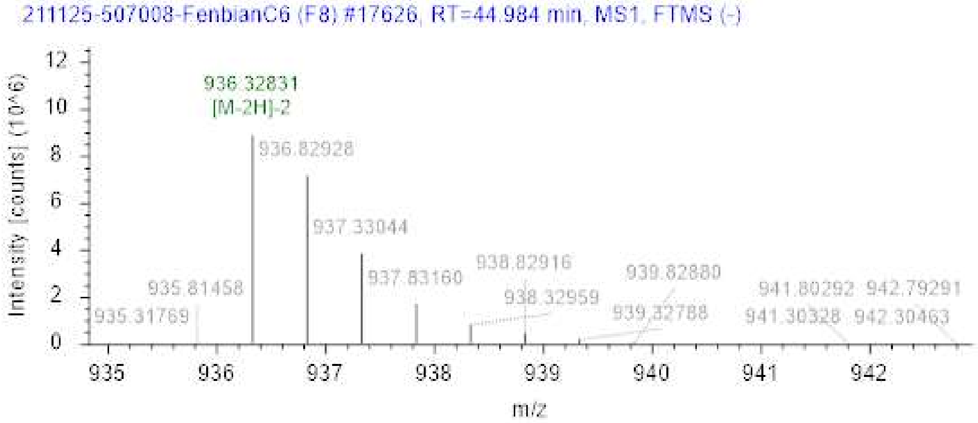

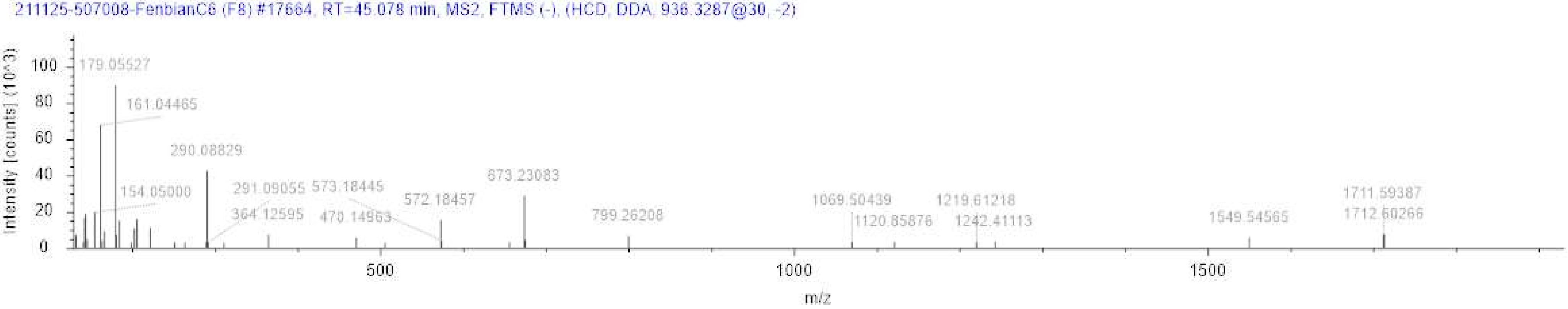

© Reported with Compound Discoverer 3.3

Structure	Name	RT [min]	Formula	Calc. MW	Areas
n/a		45.99	n/a	1875.68748	1.13e8 6.16e7 4.32e7

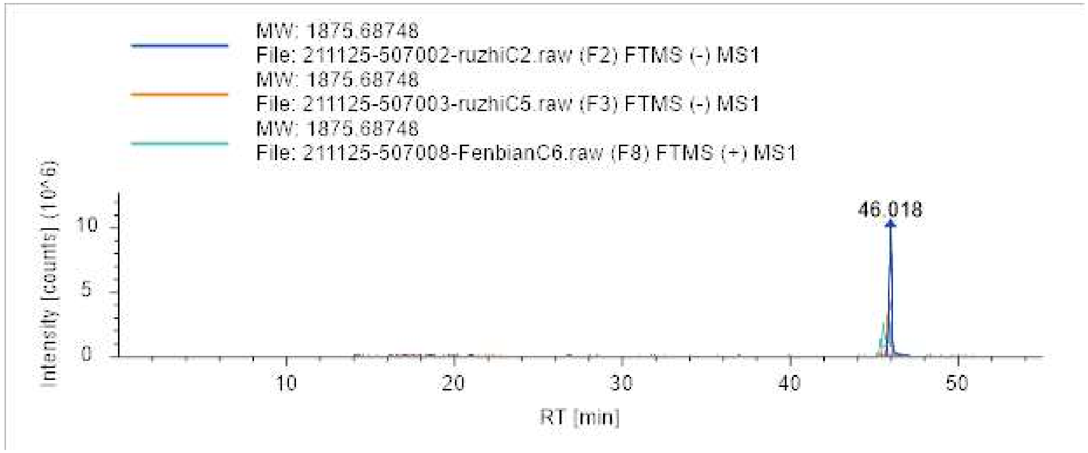

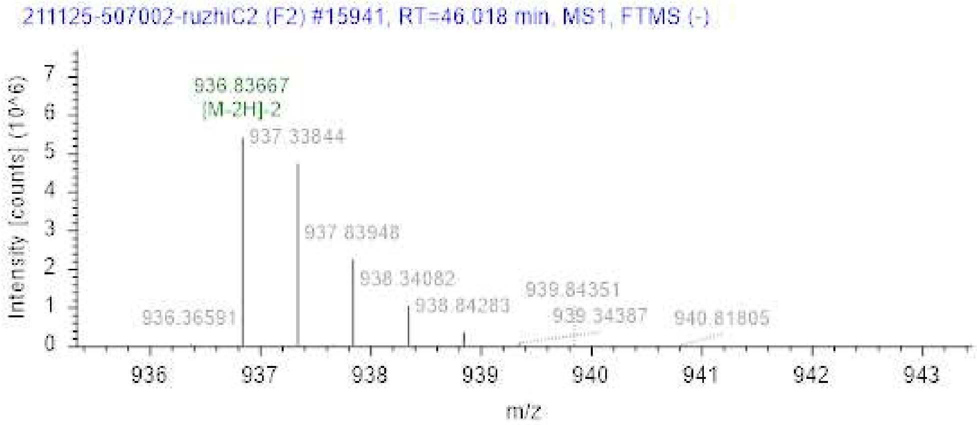

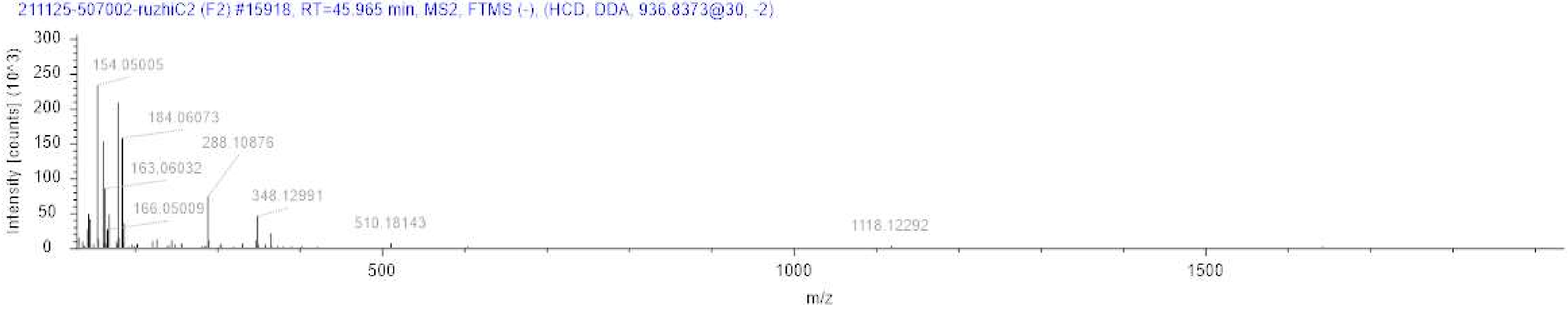

© Reported with Compound Discoverer 3.3

Compounds

Structure	Name	RT [min]	Formula	Calc. MW	Areas
n/a		45.86	n/a	1884.69590	1.30e7

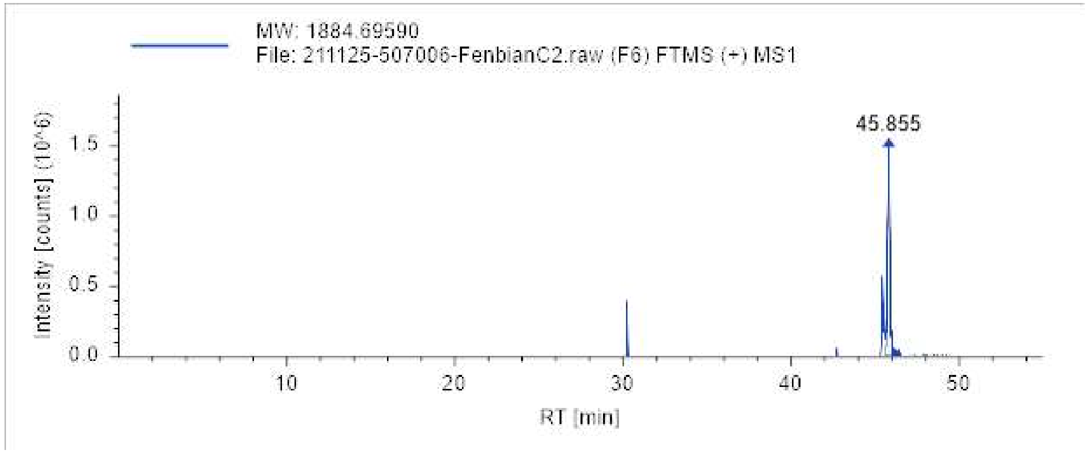

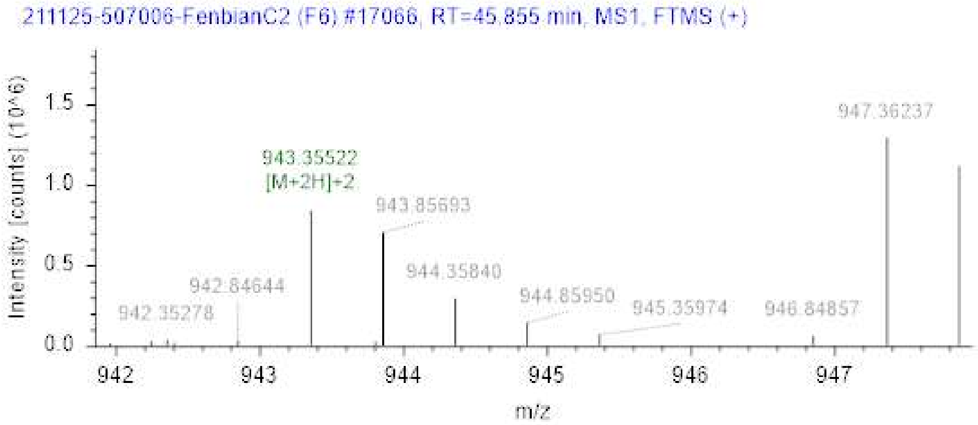

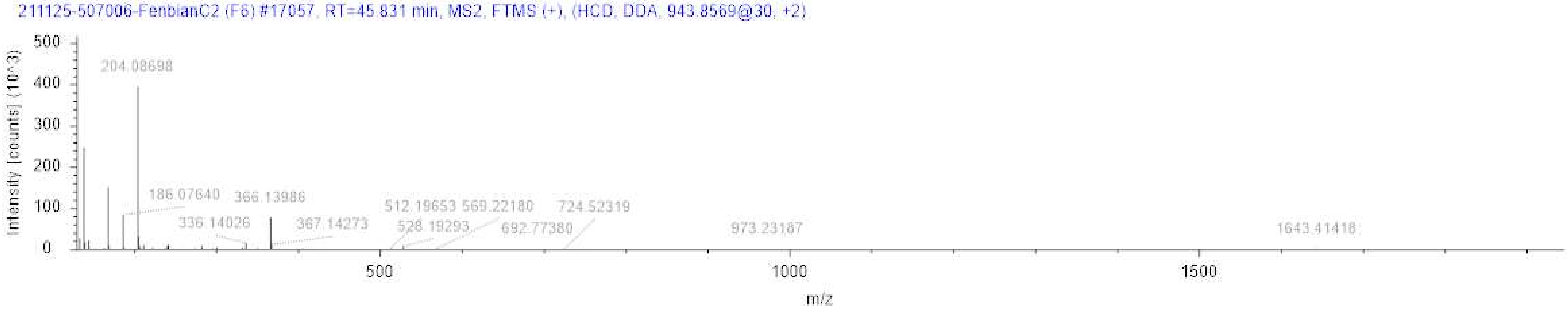

© Reported with Compound Discoverer 3.3

Structure	Name	RT [min]	Formula	Calc. MW	Areas
n/a		45.83	n/a	1900.69272	5.99e7

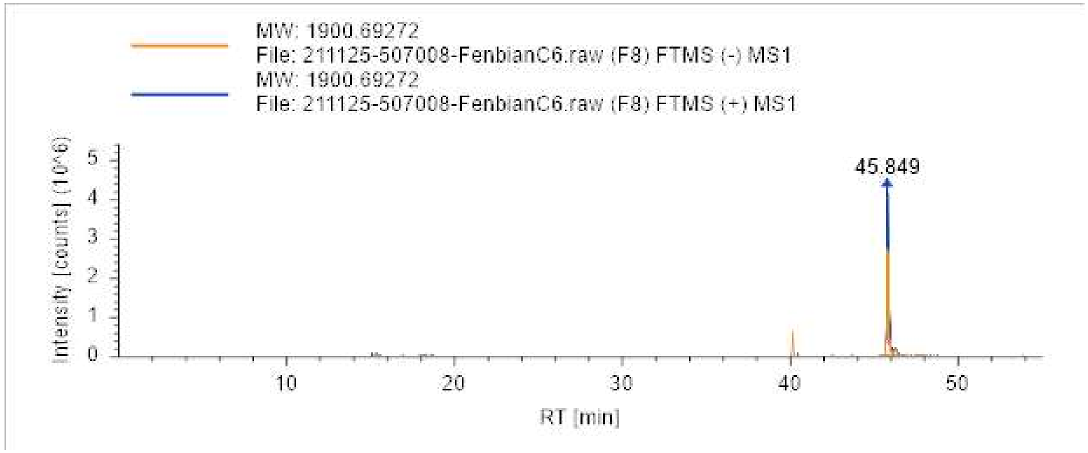

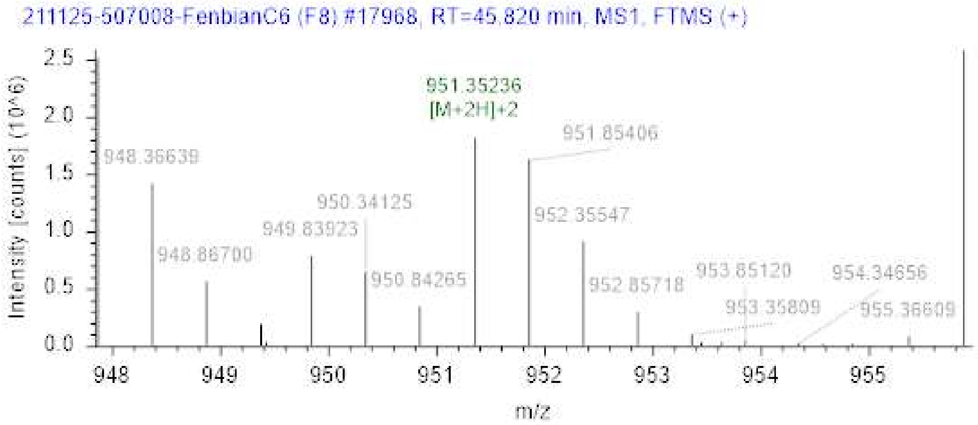

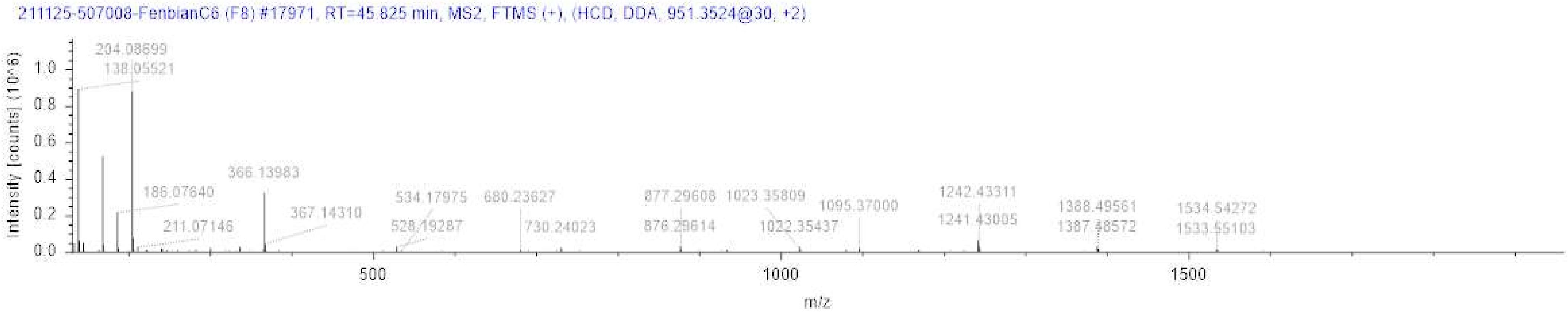

© Reported with Compound Discoverer 3.3

Compounds

Structure	Name	RT [min]	Formula	Calc. MW	Areas
n/a		44.96	n/a	1923.67971	7.10e7

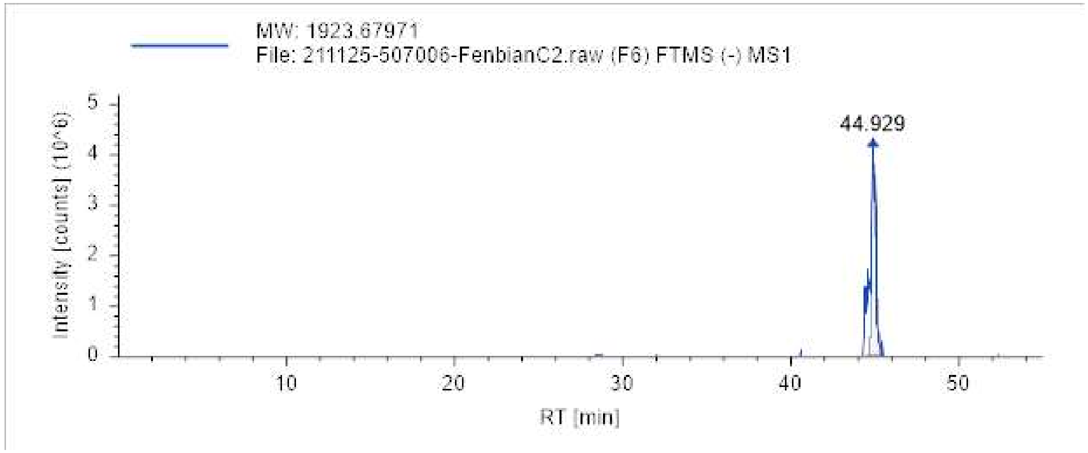

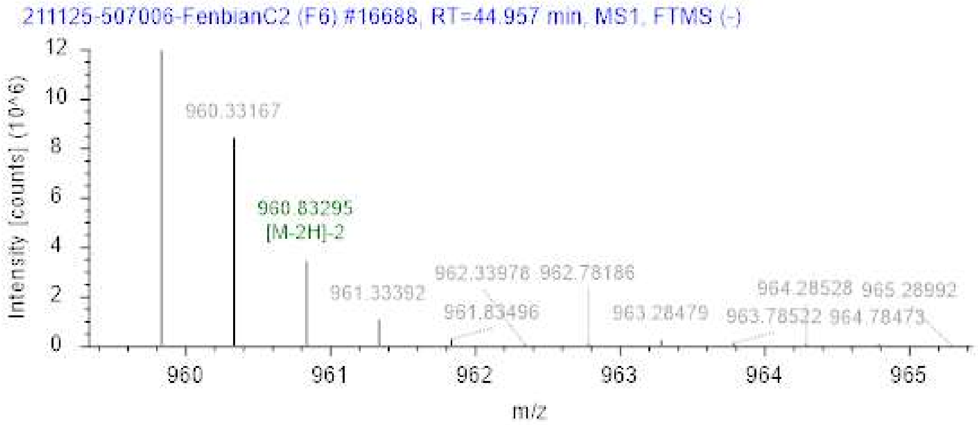

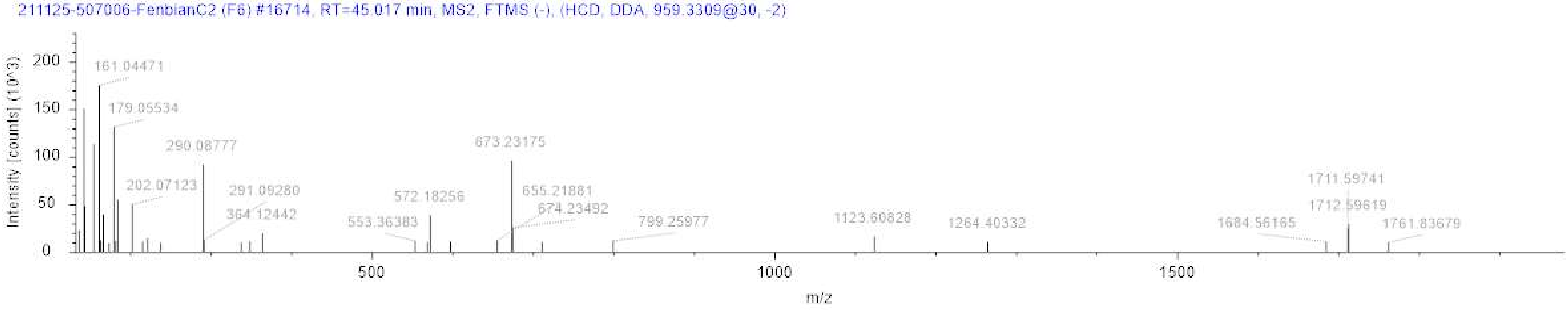

© Reported with Compound Discoverer 3.3

Compounds

Structure	Name	RT [min]	Formula	Calc. MW	Areas
n/a		44.69	n/a	1931.68674	1.10e7

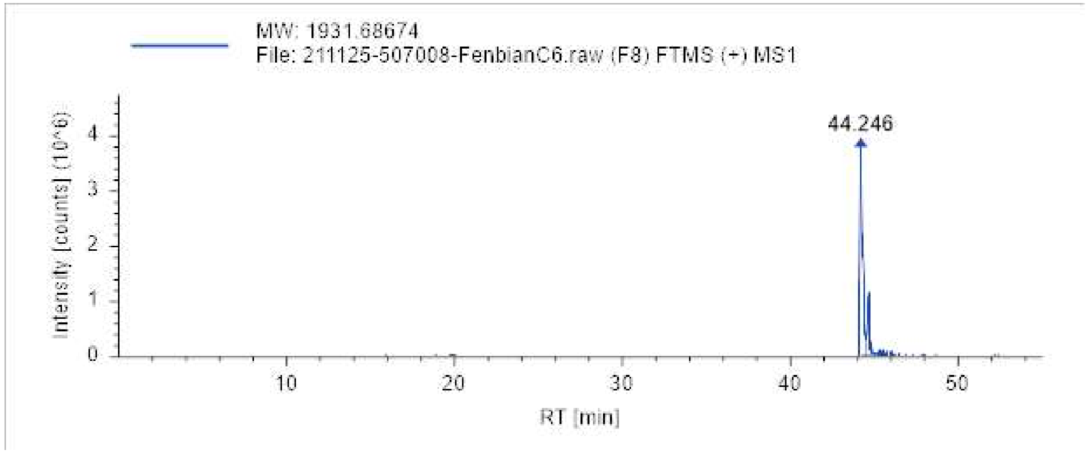

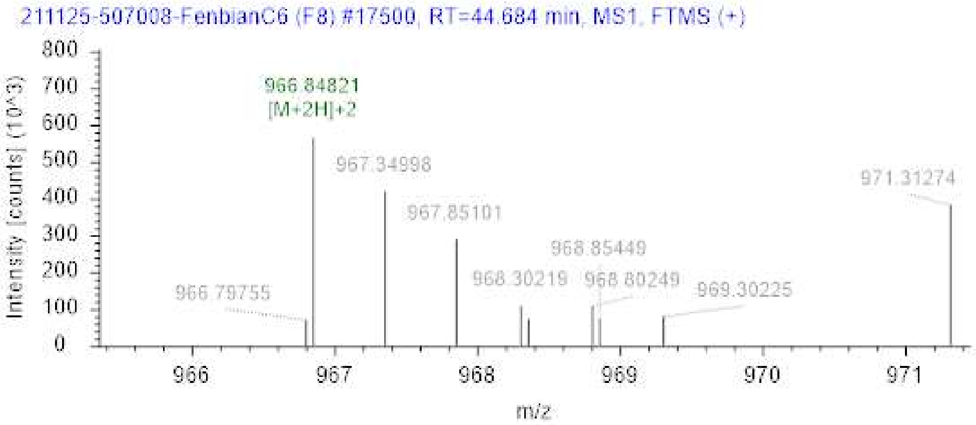

© Reported with Compound Discoverer 3.3

Compounds

Structure	Name	RT [min]	Formula	Calc. MW	Areas
n/a		45.51	n/a	1932.70530	2.02e7

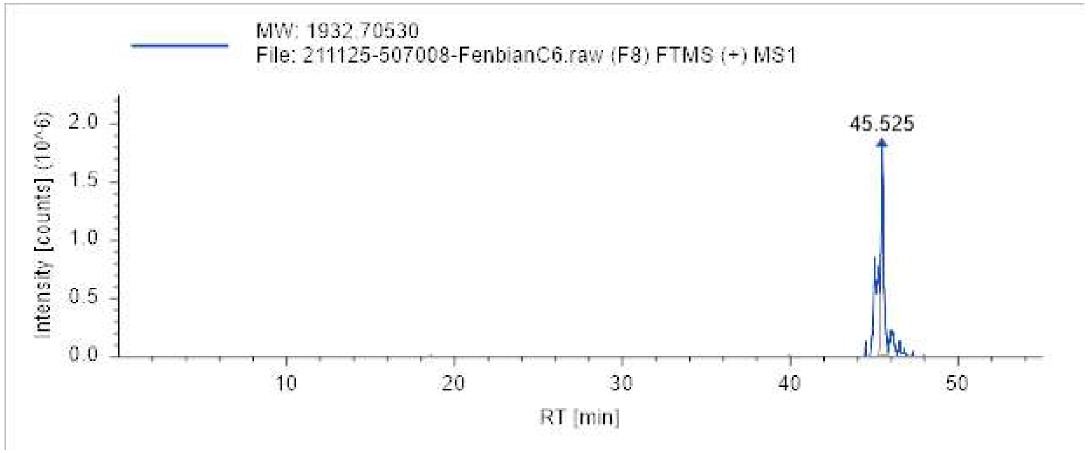

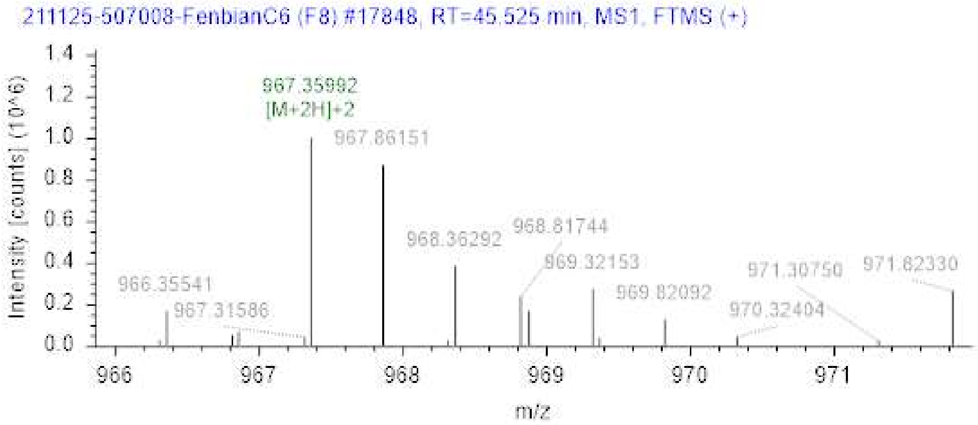

© Reported with Compound Discoverer 3.3

Compounds

Structure	Name	RT [min]	Formula	Calc. MW	Areas
n/a		44.26	n/a	1946.68496	7.28e6 2.68e7

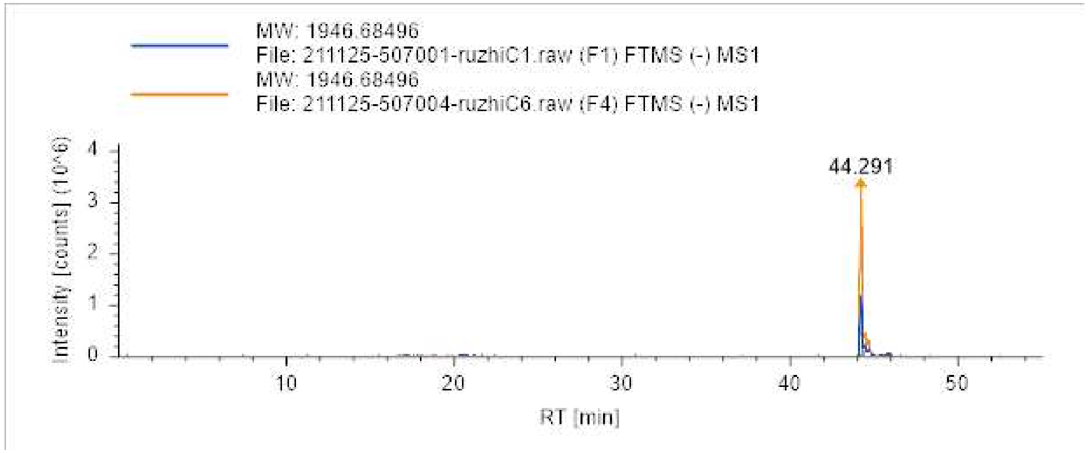

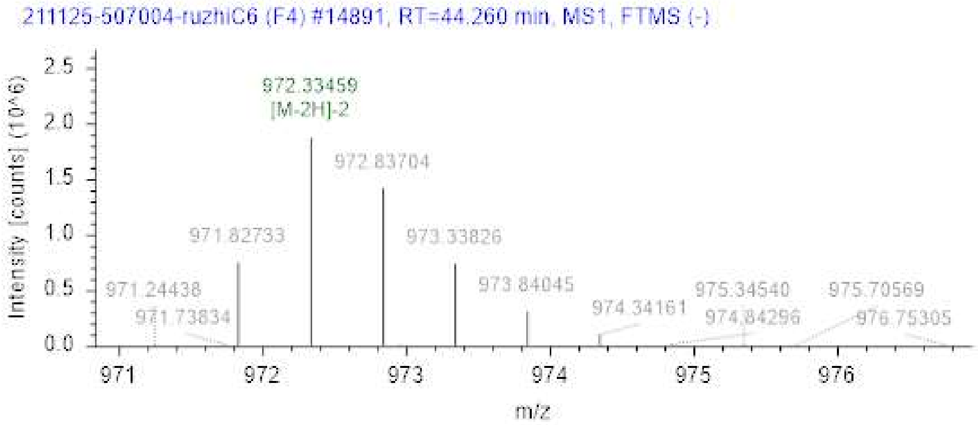

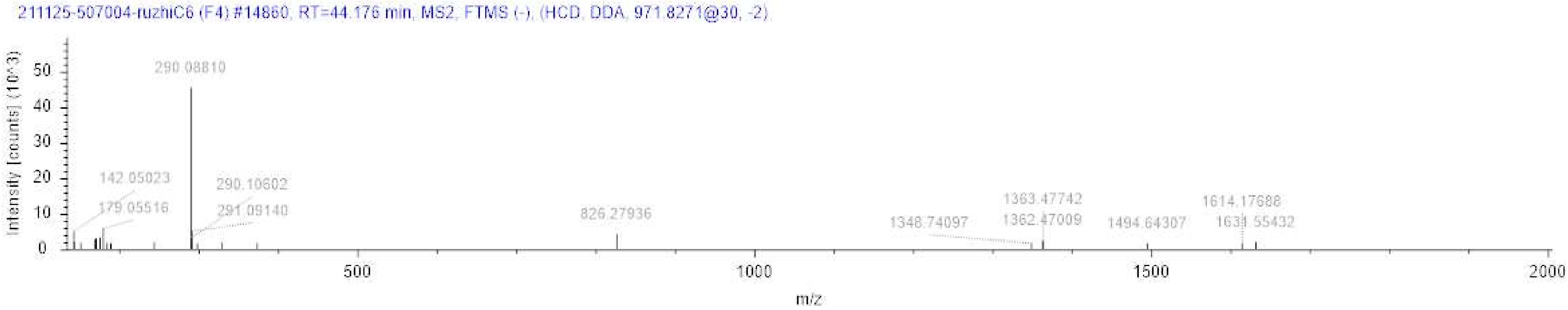

© Reported with Compound Discoverer 3.3

Compounds

Structure	Name	RT [min]	Formula	Calc. MW	Areas
n/a		46.18	n/a	1948.69895	1.55e7

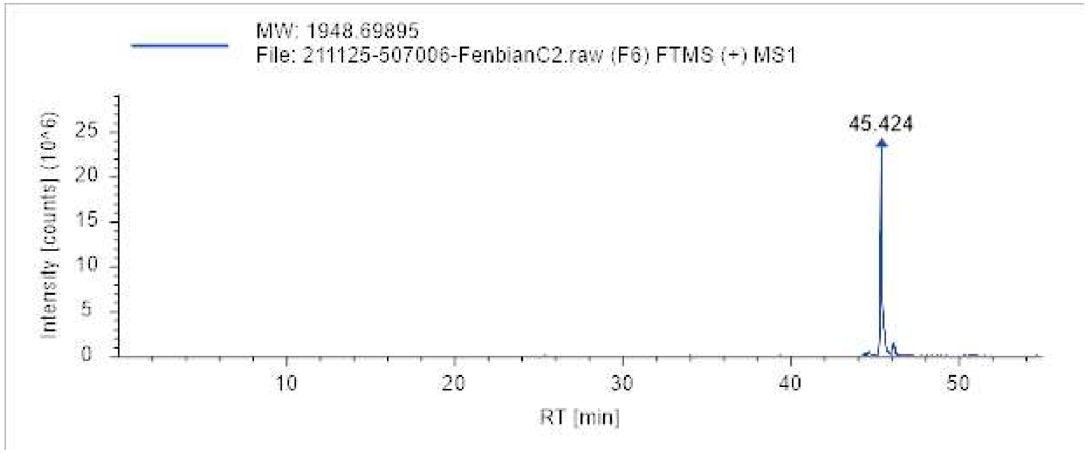

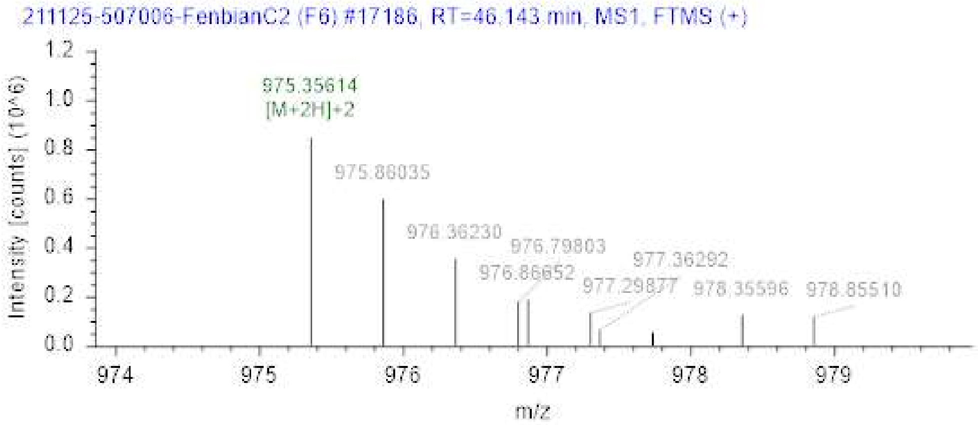

© Reported with Compound Discoverer 3.3

Compounds

Structure	Name	RT [min]	Formula	Calc. MW	Areas
n/a		45.50	n/a	1948.70085	1.63e7 1.61e8 2.06e7 1.43e8

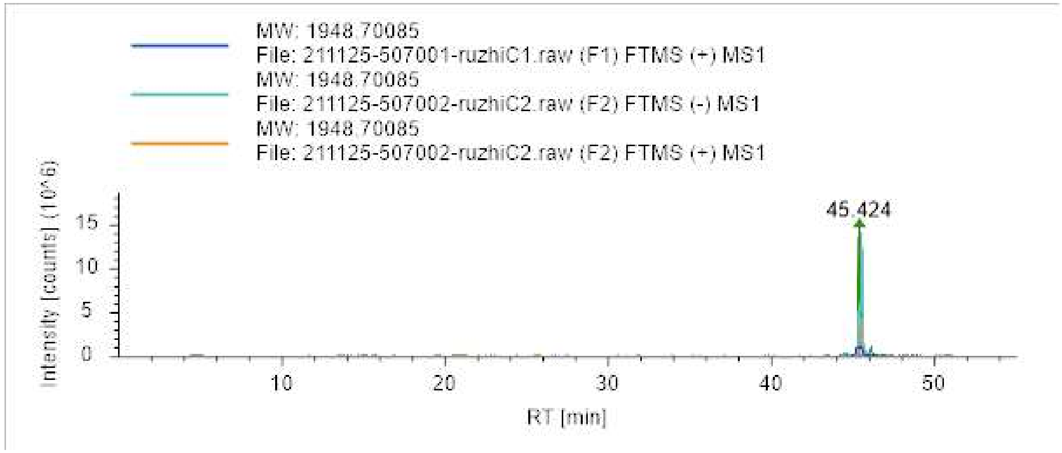

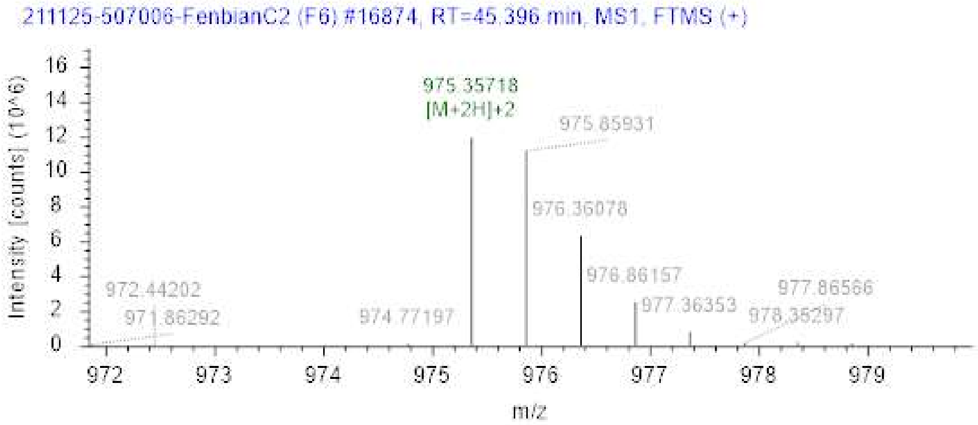

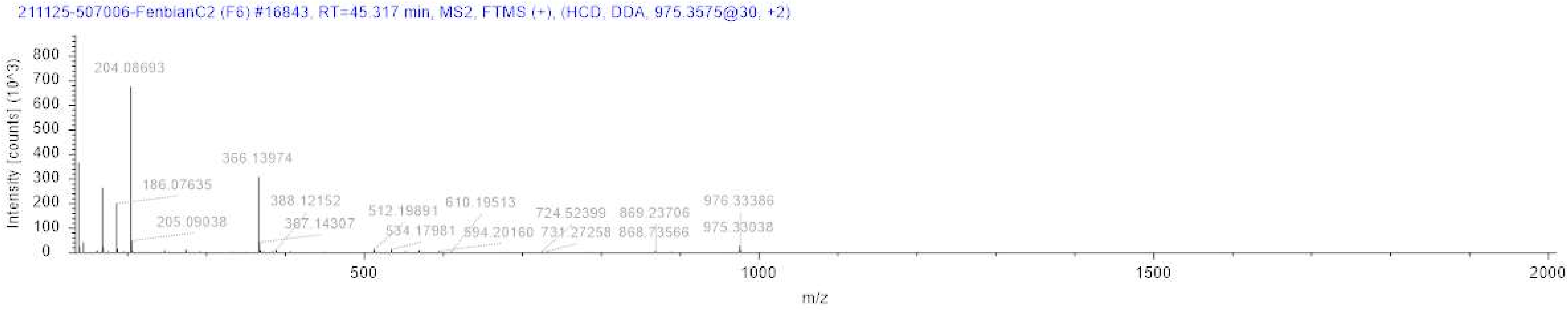

© Reported with Compound Discoverer 3.3

Structure	Name	RT [min]	Formula	Calc. MW	Areas
n/a		45.31	n/a	1949.70139	1.30e7

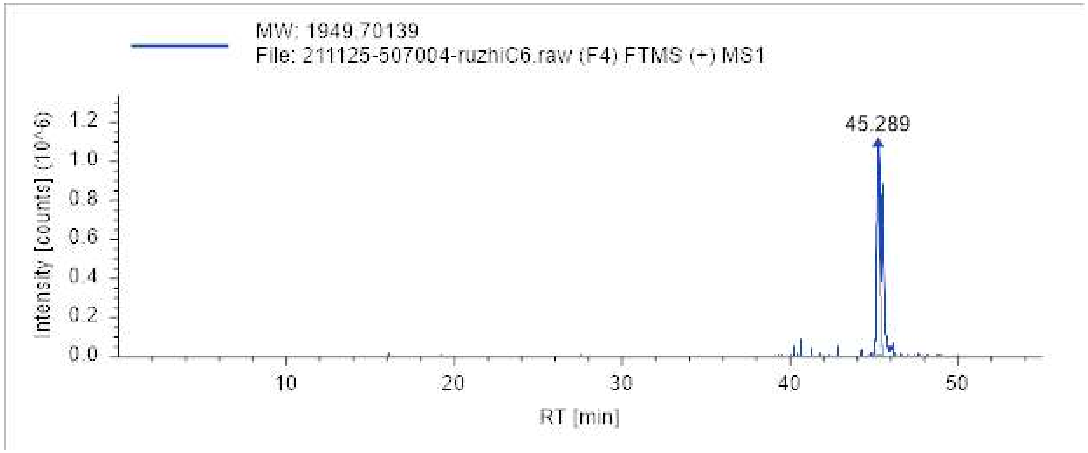

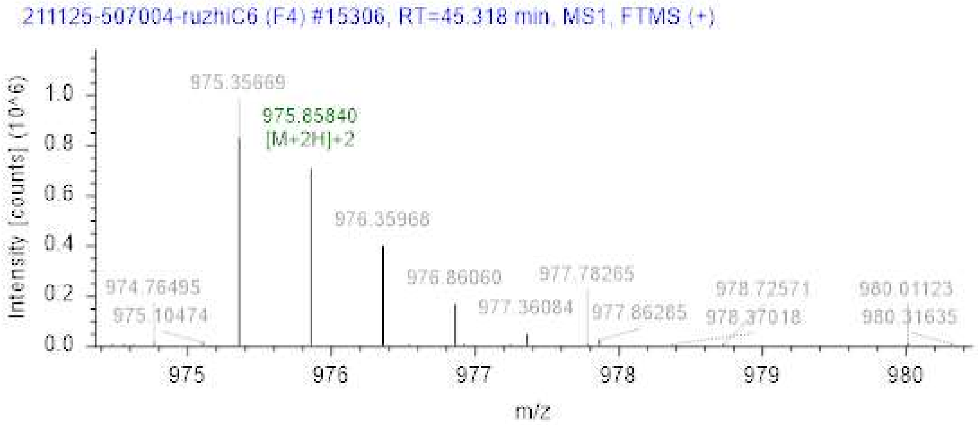

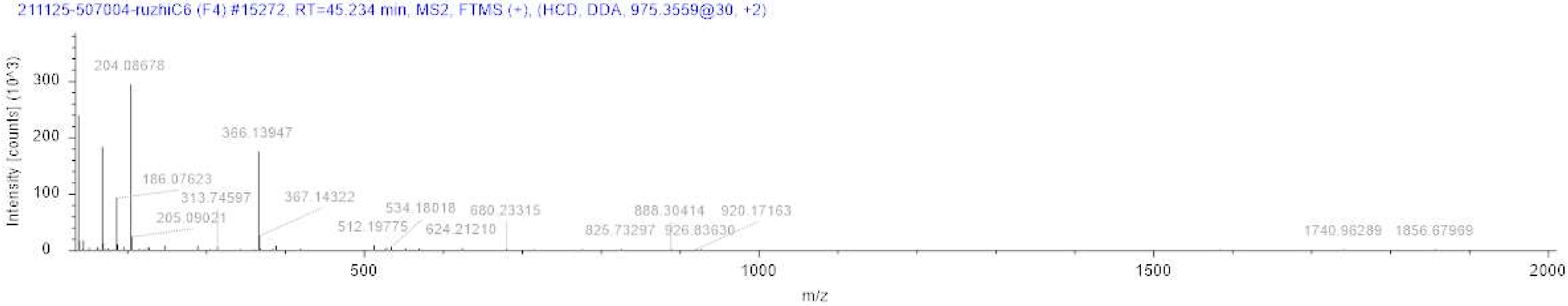

© Reported with Compound Discoverer 3.3

Compounds

Structure	Name	RT [min]	Formula	Calc. MW	Areas
n/a		45.57	n/a	1950.71109	8.95e6

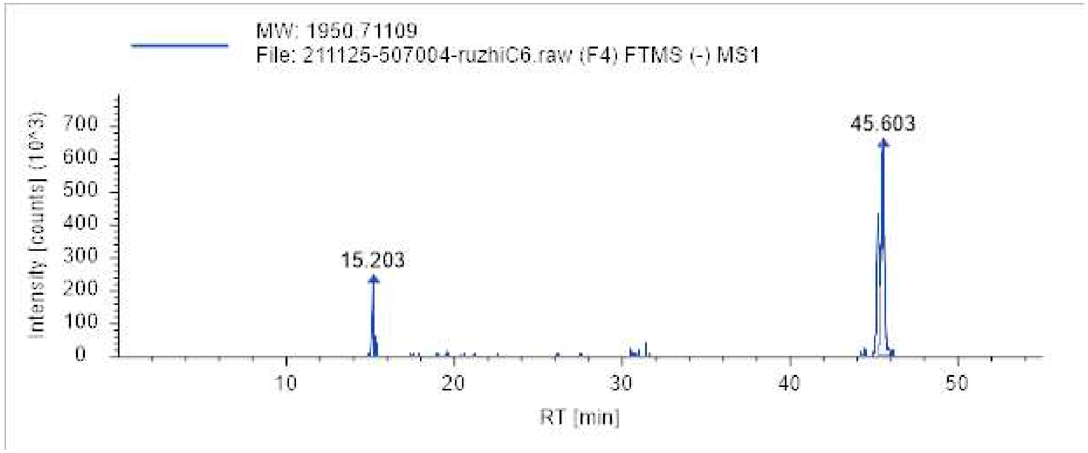

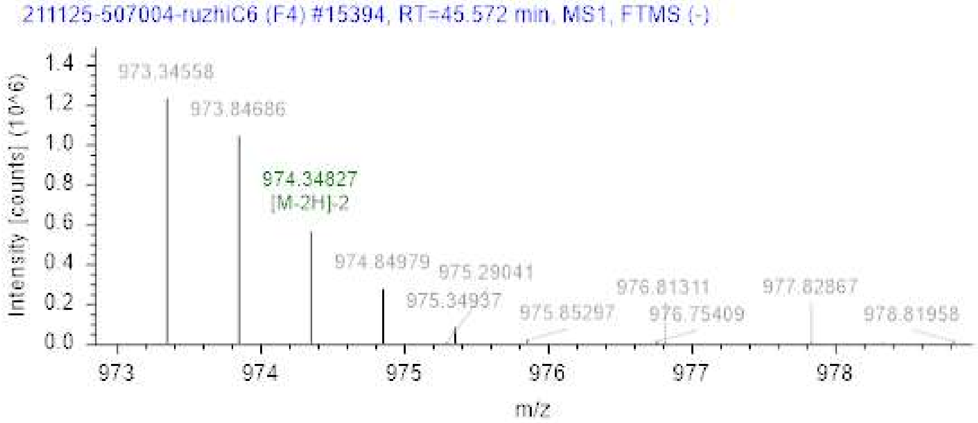

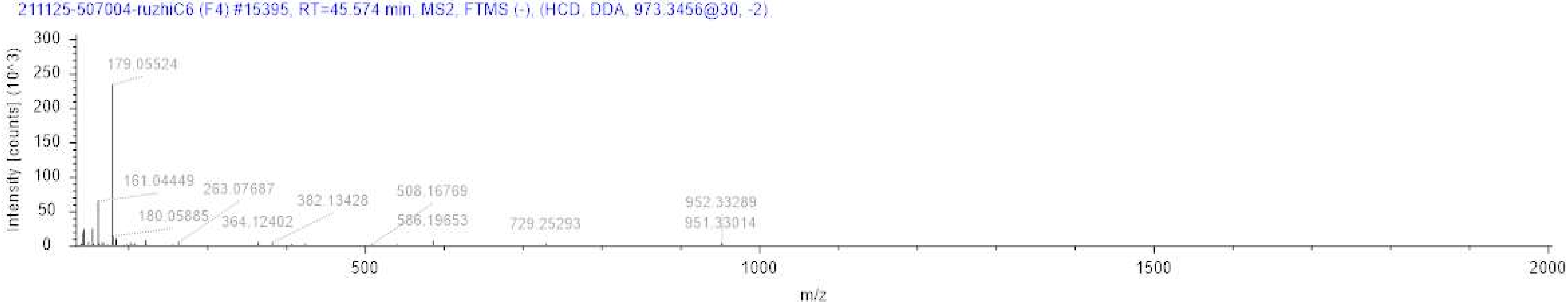

© Reported with Compound Discoverer 3.3

Compounds

Structure	Name	RT [min]	Formula	Calc. MW	Areas
n/a		44.73	n/a	2019.70576	3.82e8

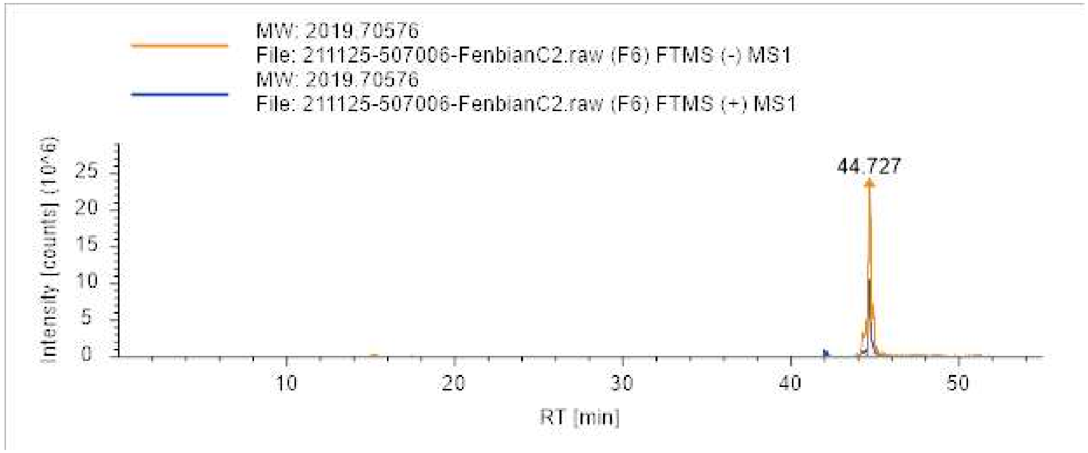

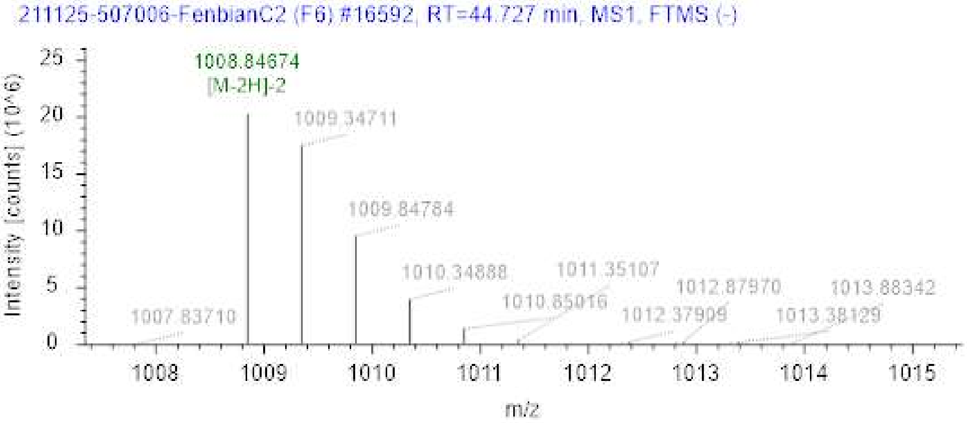

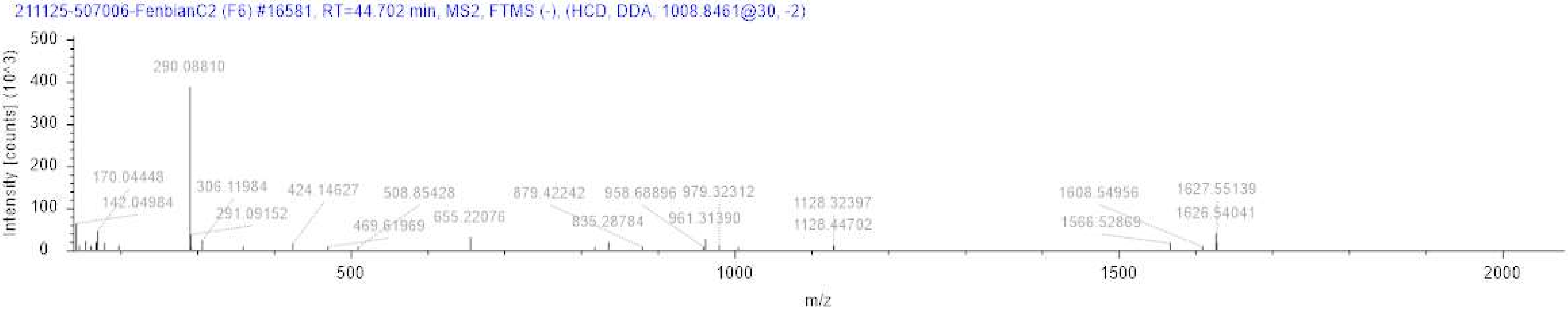

© Reported with Compound Discoverer 3.3

Compounds

Structure	Name	RT [min]	Formula	Calc. MW	Areas
n/a		45.33	n/a	2020.72275	6.99e7

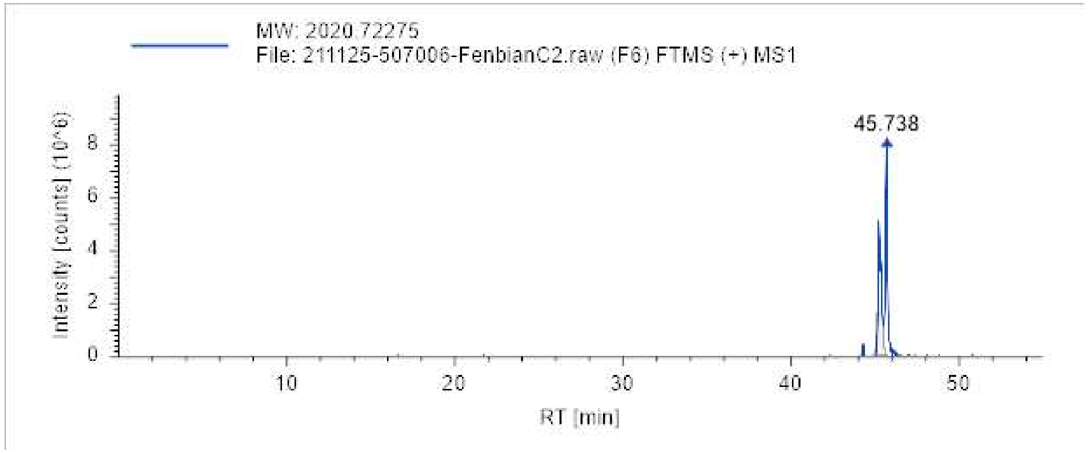

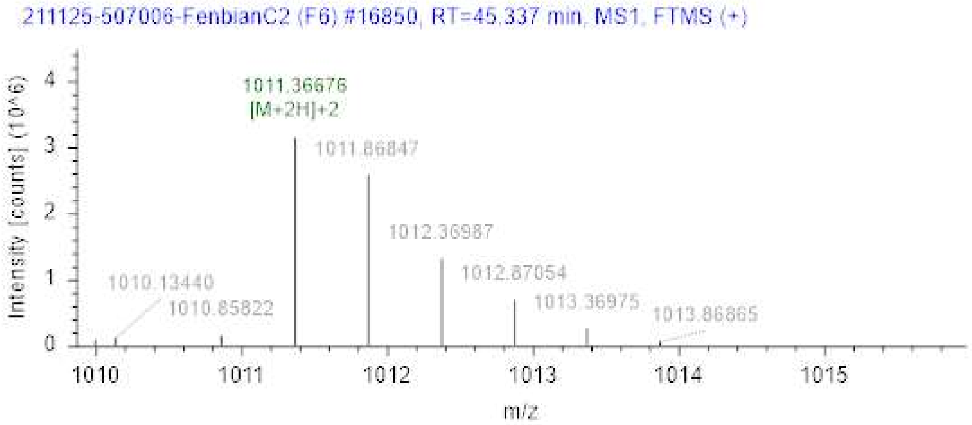

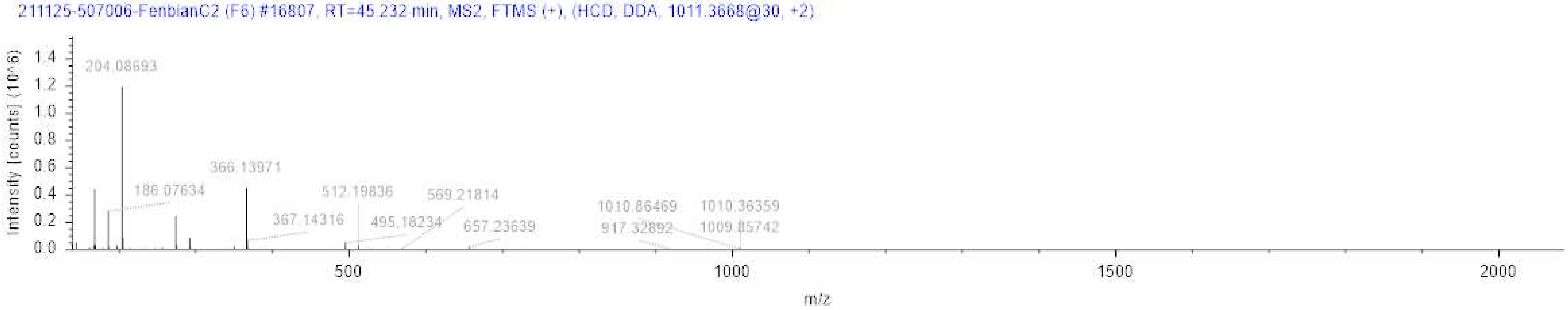

© Reported with Compound Discoverer 3.3

Structure	Name	RT [min]	Formula	Calc. MW	Areas
n/a		45.71	n/a	2037.72378	2.11e7

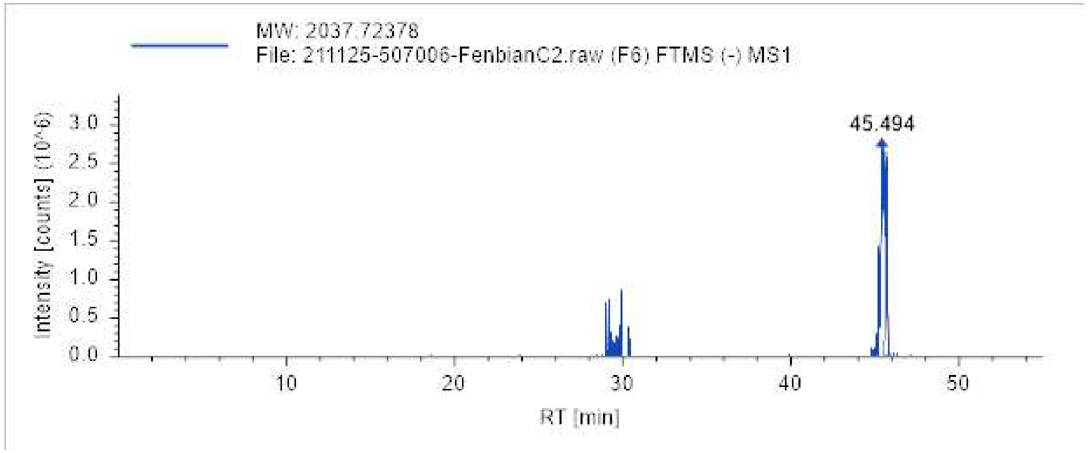

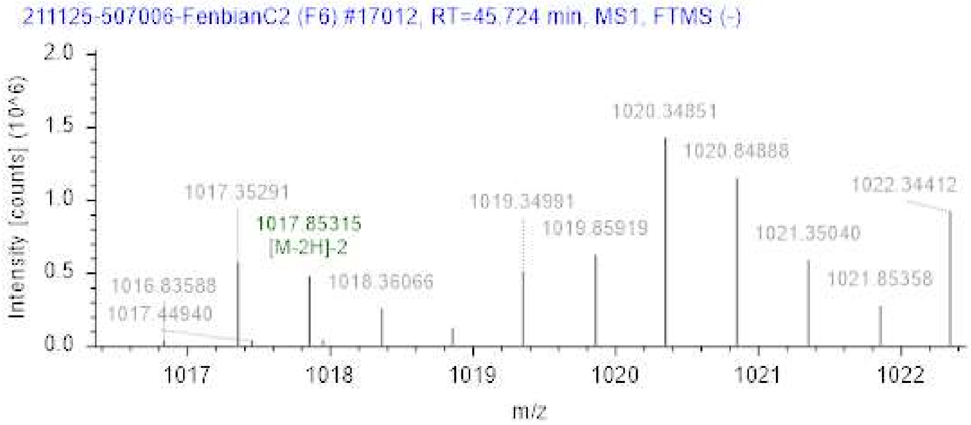

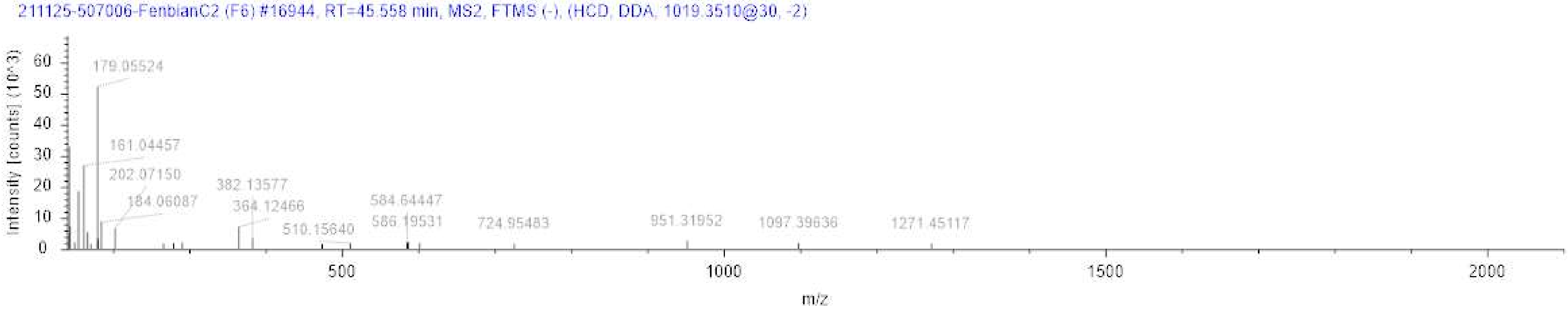

© Reported with Compound Discoverer 3.3

Compounds

Structure	Name	RT [min]	Formula	Calc. MW	Areas
n/a		46.01	n/a	2045.73477	1.28e7

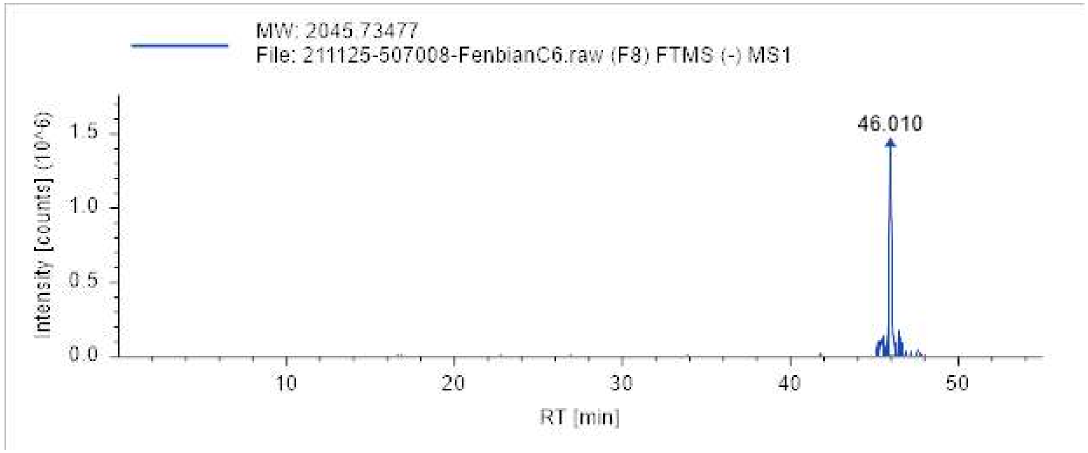

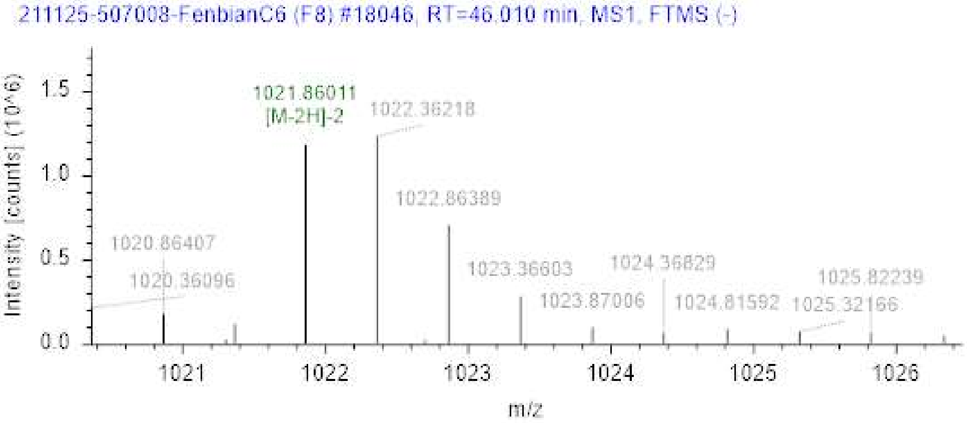

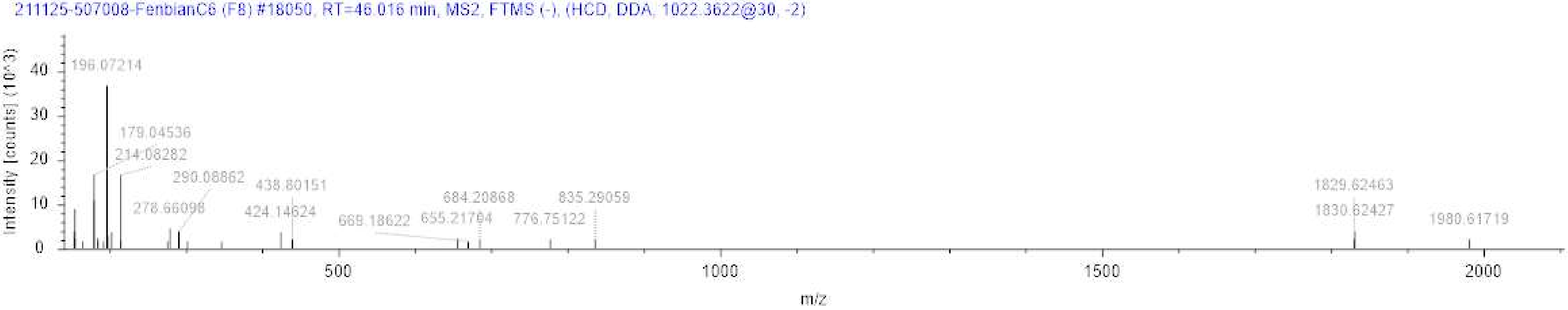

© Reported with Compound Discoverer 3.3

Structure	Name	RT [min]	Formula	Calc. MW	Areas
n/a		46.47	n/a	2046.75650	6.29e6

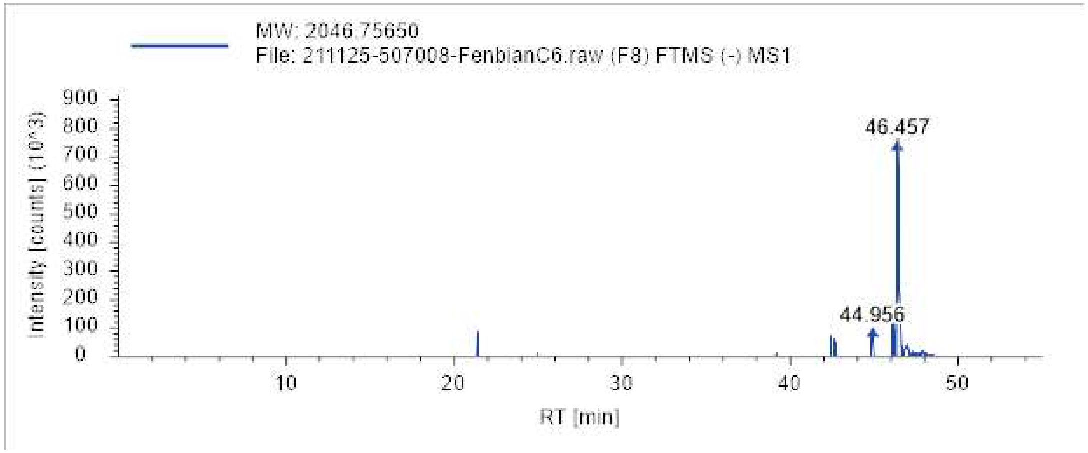

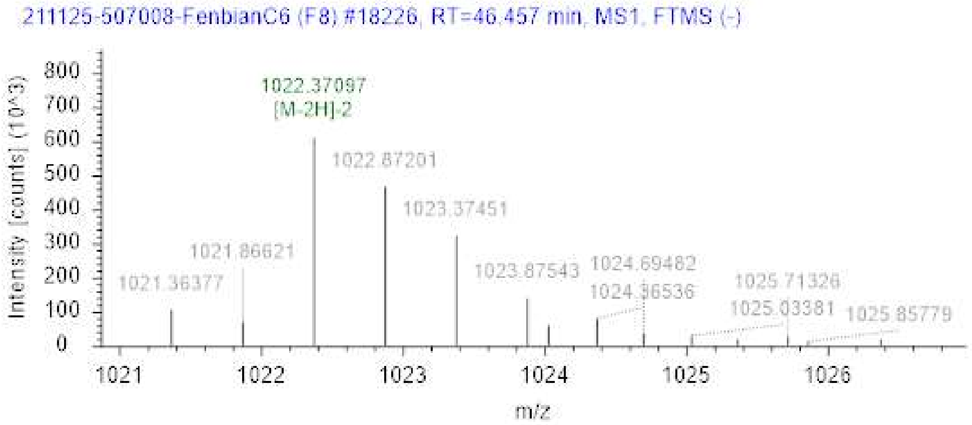

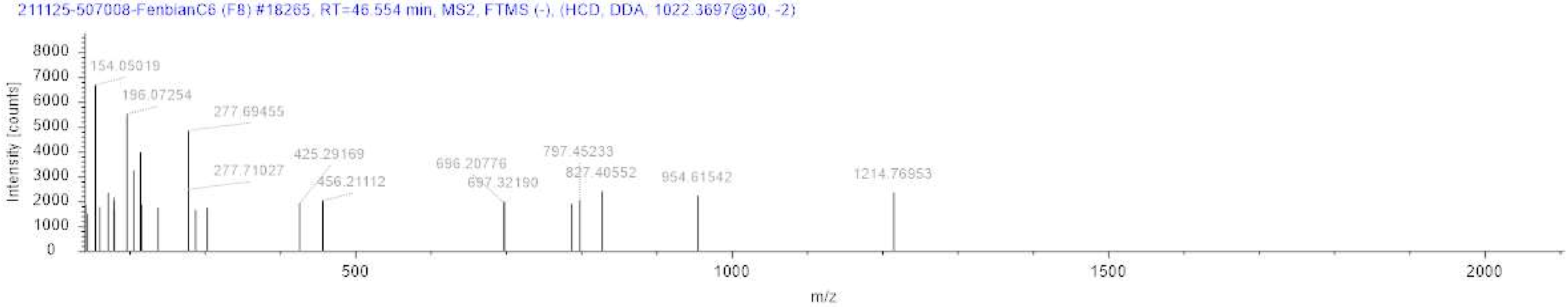

© Reported with Compound Discoverer 3.3

Structure	Name	RT [min]	Formula	Calc. MW	Areas
n/a		45.56	n/a	2062.69763	1.23e7 ; 3.09e7

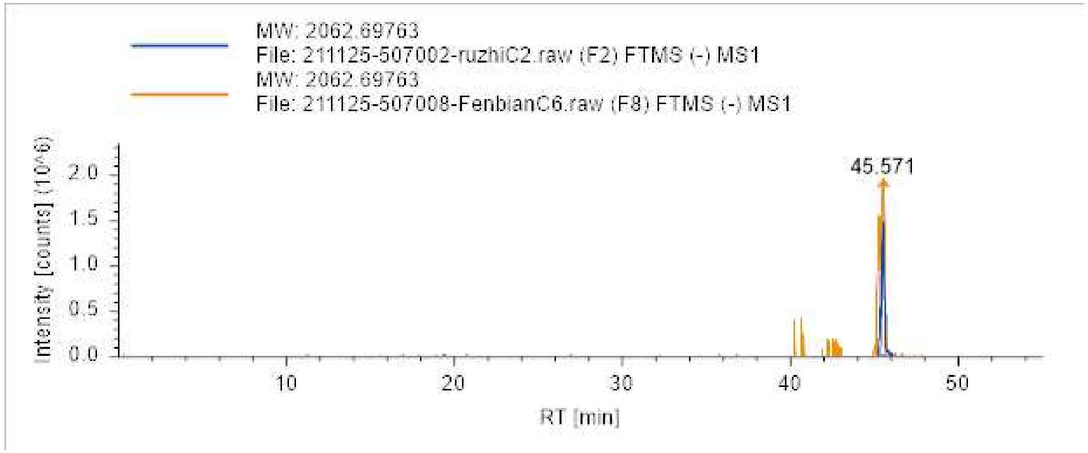

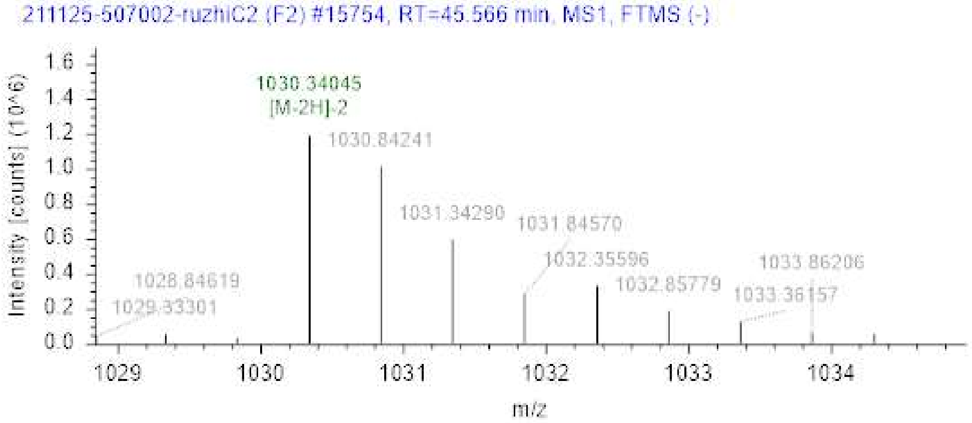

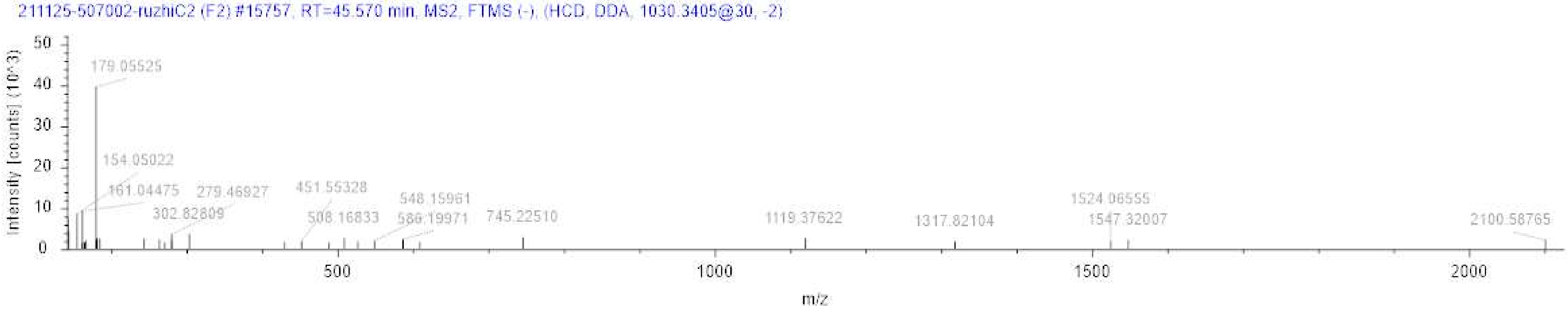

© Reported with Compound Discoverer 3.3

Compounds

Structure	Name	RT [min]	Formula	Calc. MW	Areas
n/a		45.71	n/a	2071.74063	8.33e6

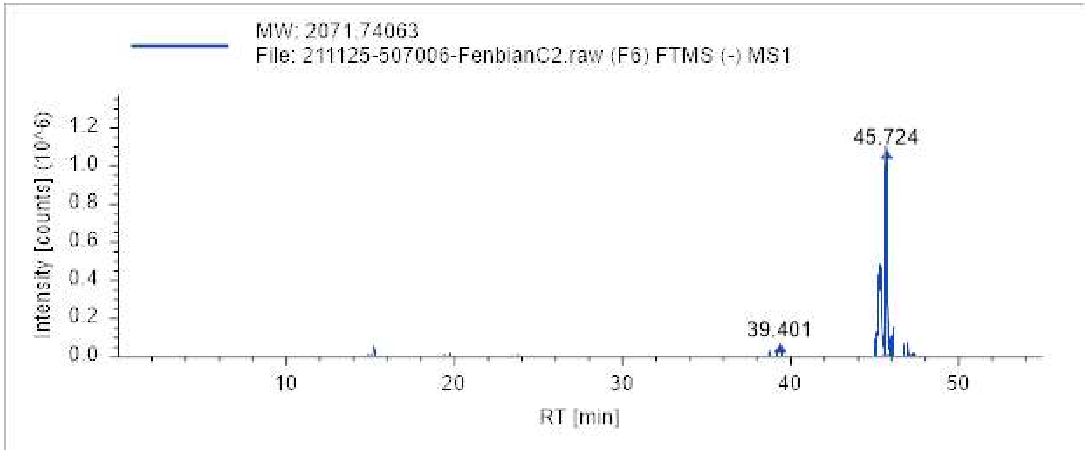

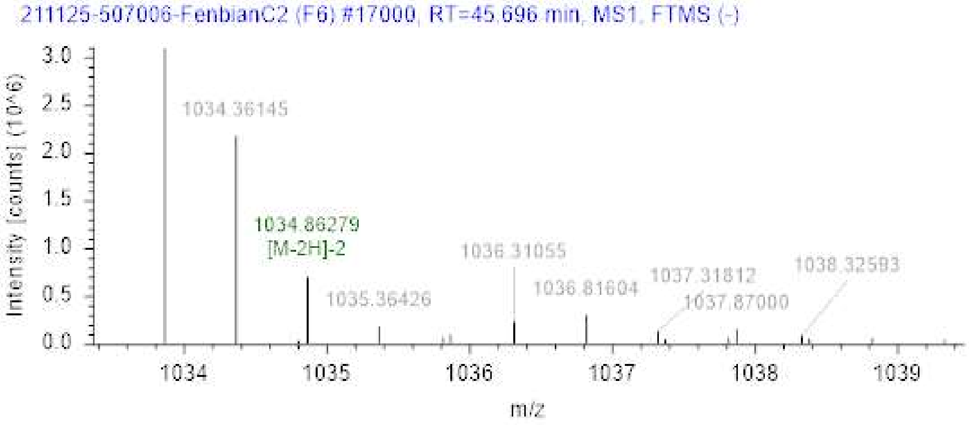

© Reported with Compound Discoverer 3.3

Compounds

Structure	Name	RT [min]	Formula	Calc. MW	Areas
n/a		44.95	n/a	2077.74131	2.49e7, 3.56e6

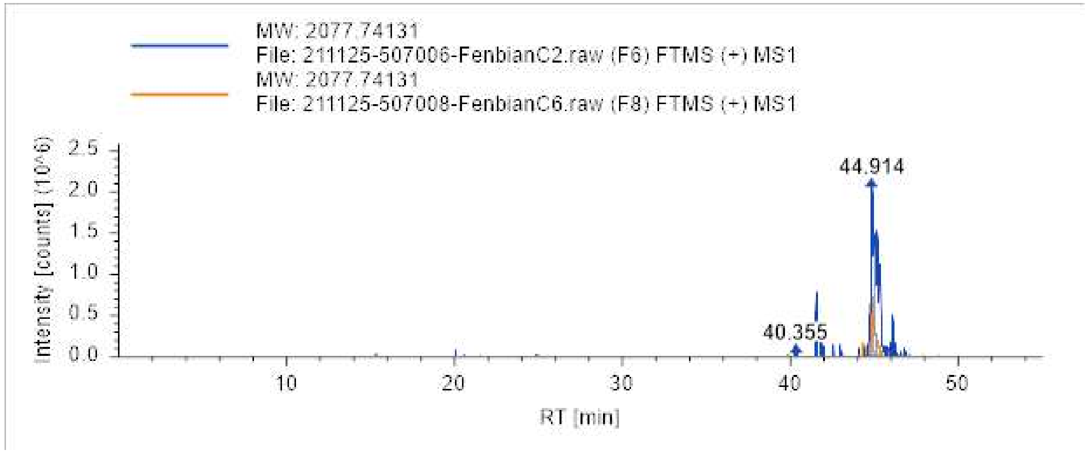

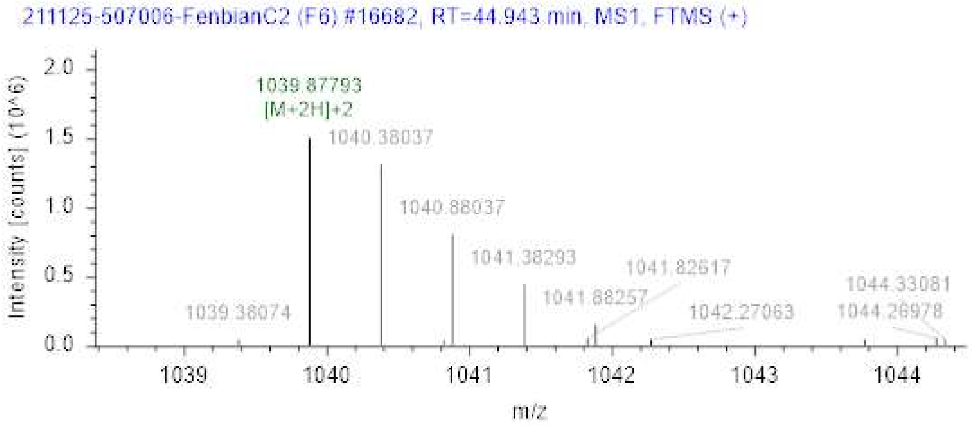

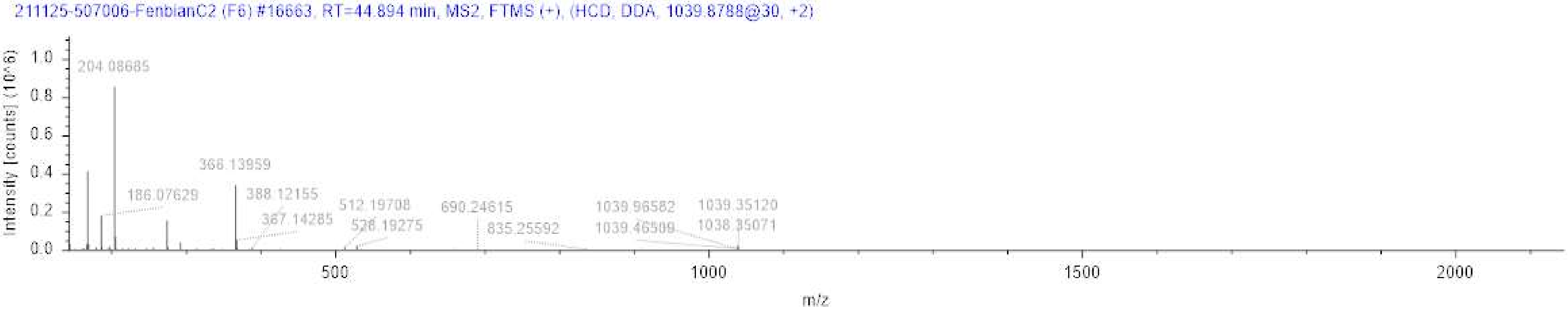

© Reported with Compound Discoverer 3.3

Compounds

Structure	Name	RT [min]	Formula	Calc. MW	Areas
n/a		46.27	n/a	2078.77310	1.23e7

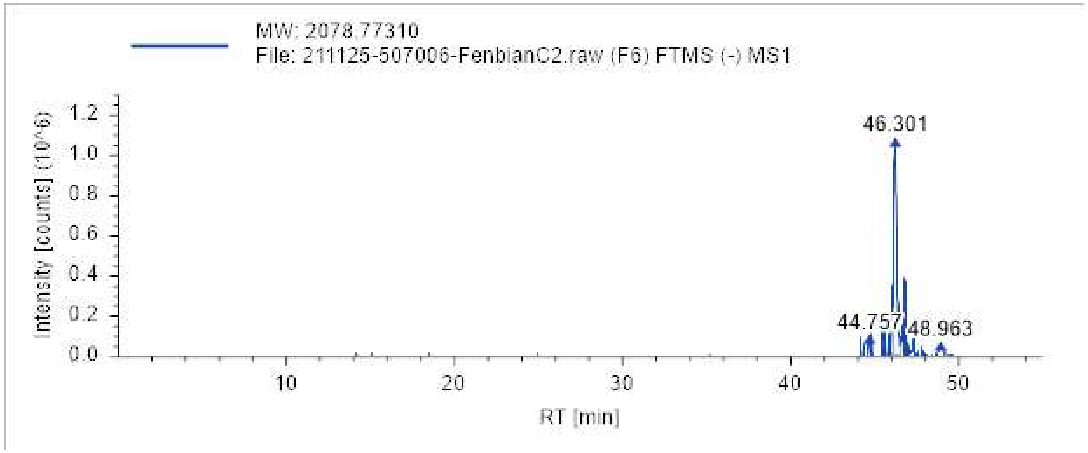

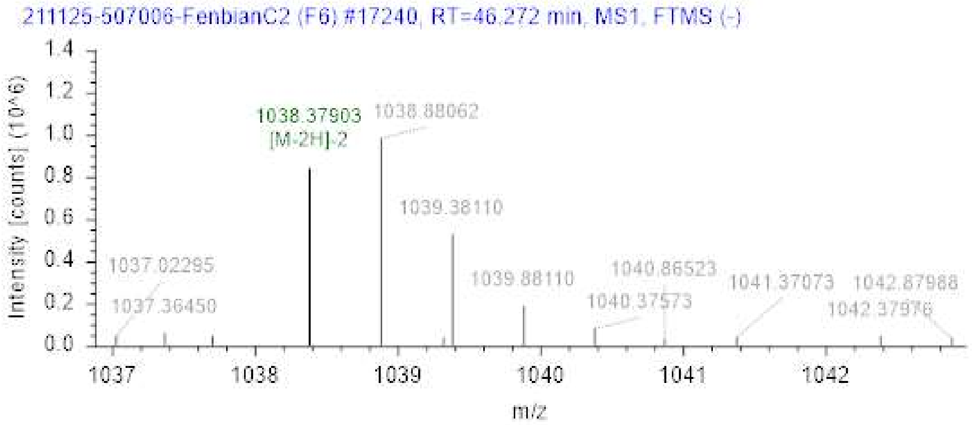

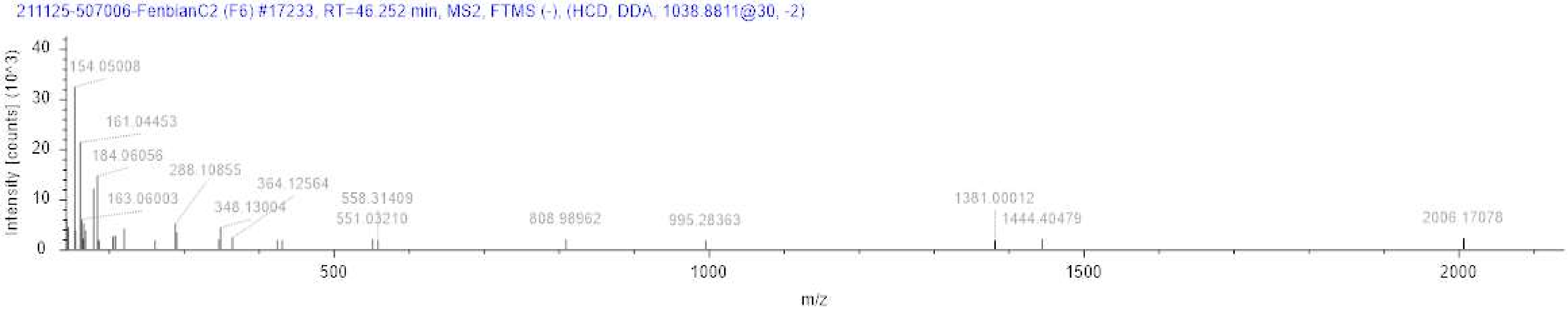

© Reported with Compound Discoverer 3.3

Structure	Name	RT [min]	Formula	Calc. MW	Areas
n/a		45.33	n/a	2093.74274	1.07e7; 6.79e6; 8.17e7

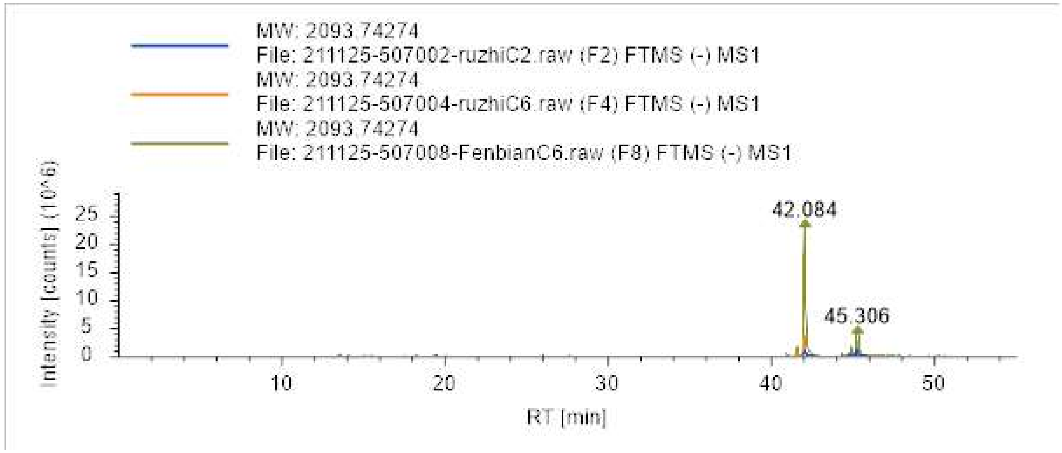

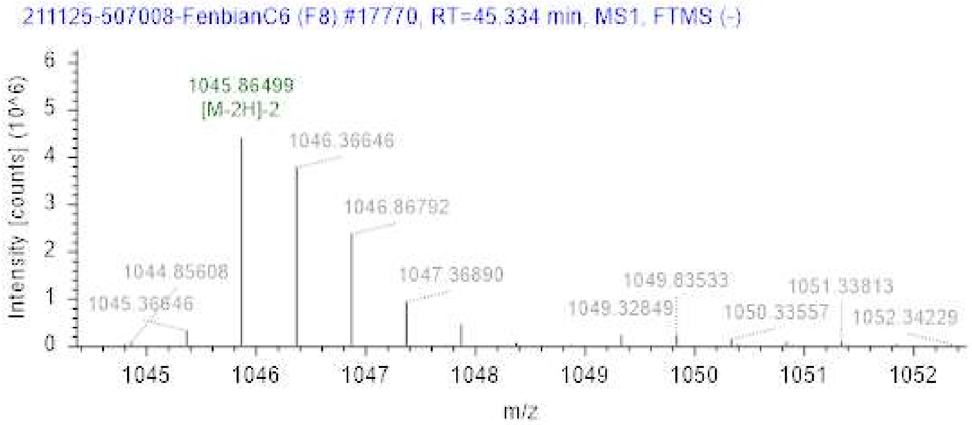

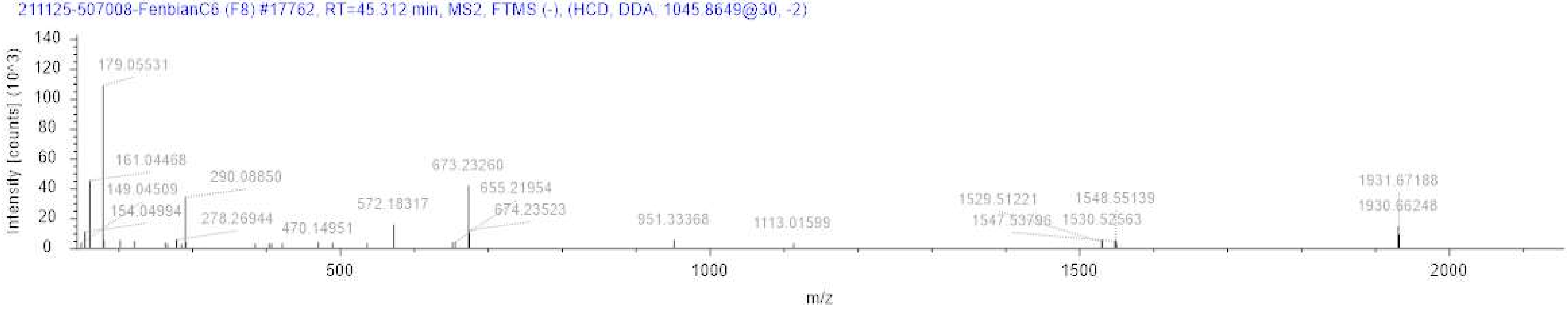

© Reported with Compound Discoverer 3.3

Compounds

Structure	Name	RT [min]	Formula	Calc. MW	Areas
n/a		46.16	n/a	2094.76709	2.64e7 9.64e7 2.08e7 3.03e7 5.67e8 2.94e8

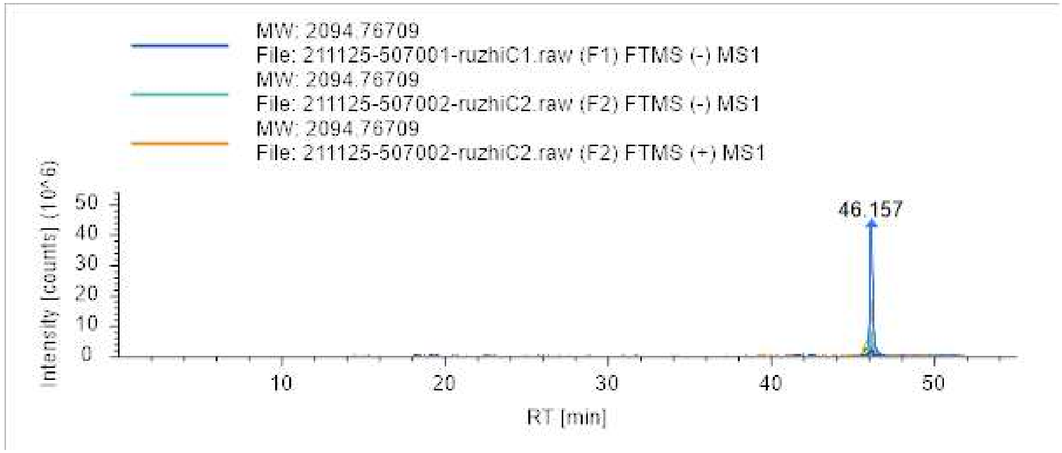

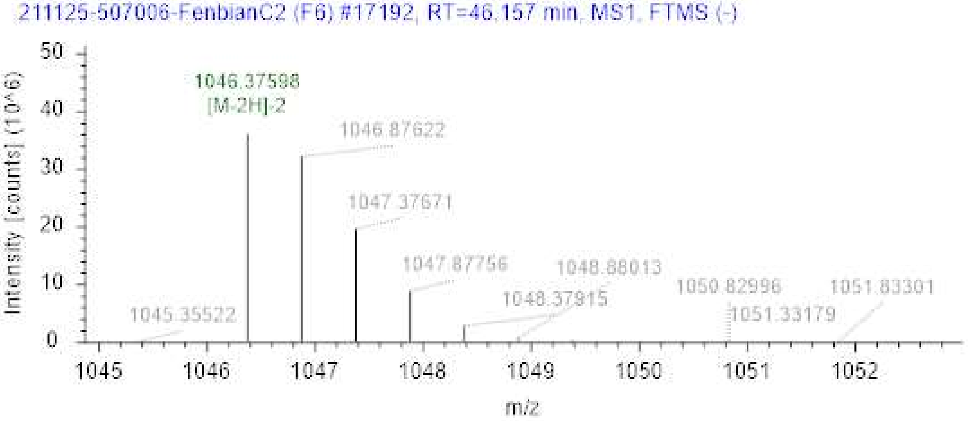

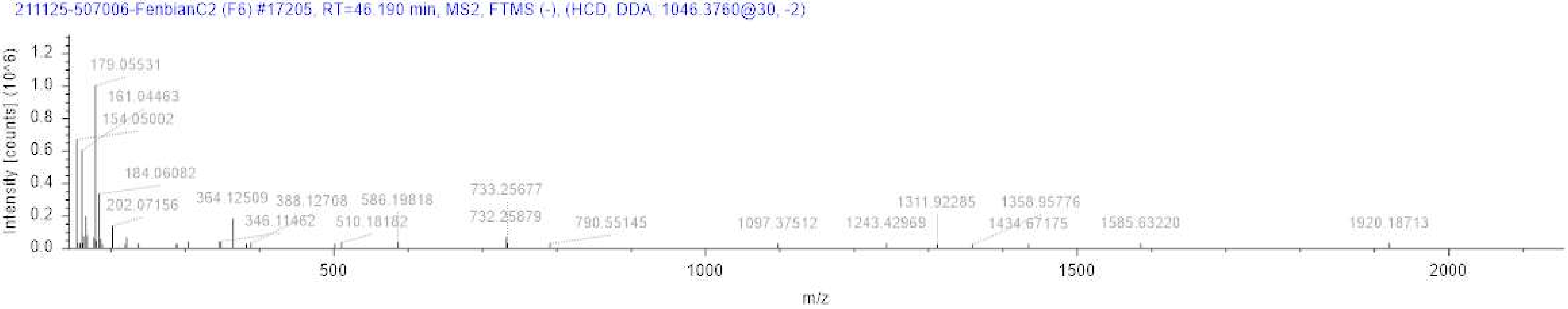

© Reported with Compound Discoverer 3.3

Compounds

Structure	Name	RT [min]	Formula	Calc. MW	Areas
n/a		45.33	n/a	2095.74831	4.00e7

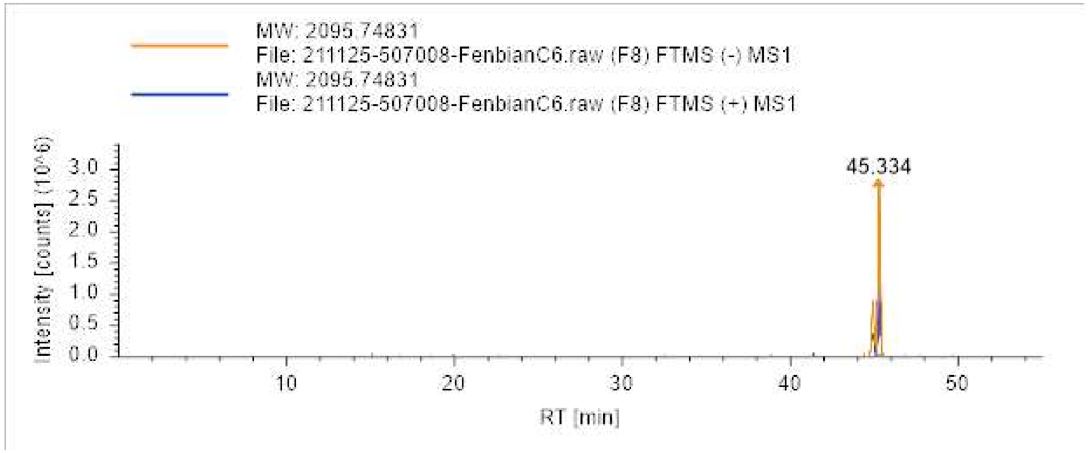

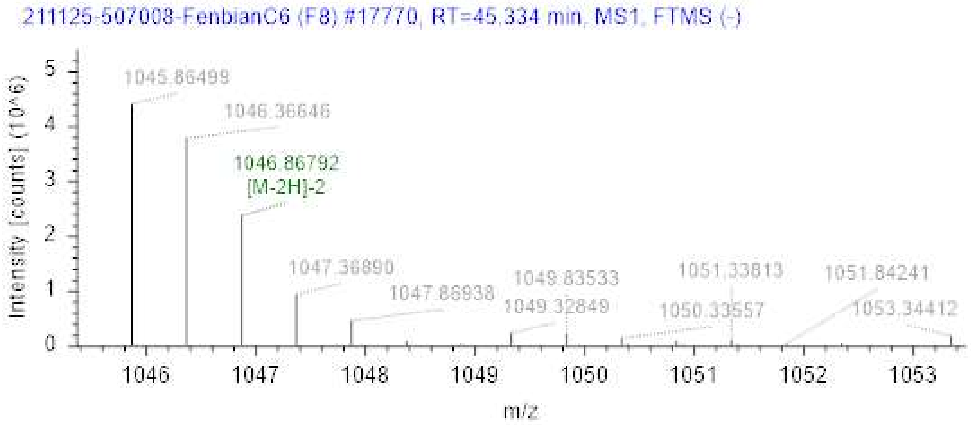

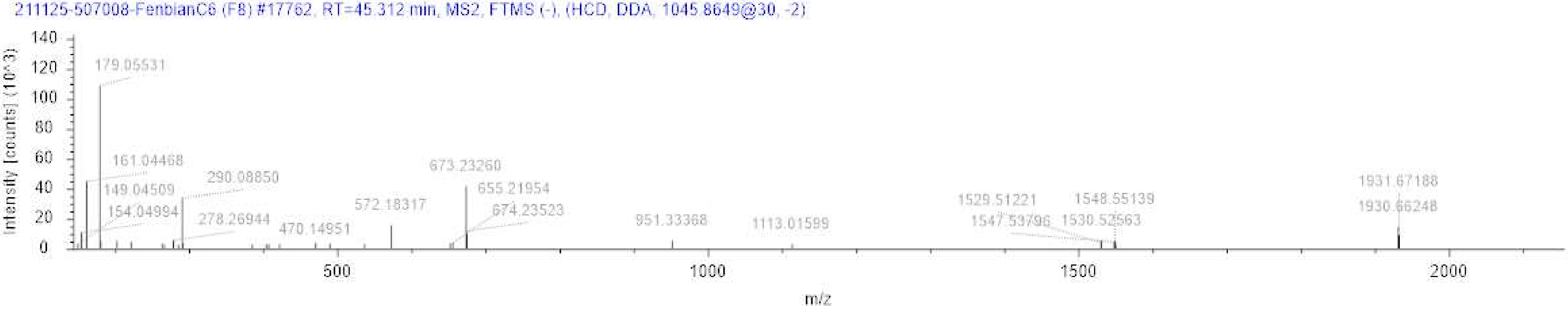

© Reported with Compound Discoverer 3.3

Compounds

Structure	Name	RT [min]	Formula	Calc. MW	Areas
n/a		45.31	n/a	2110.76401	1.25e7

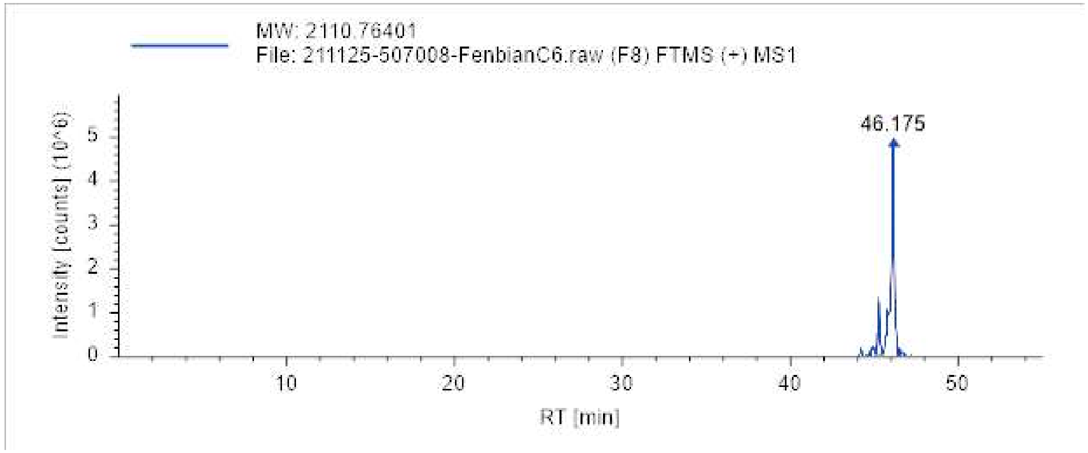

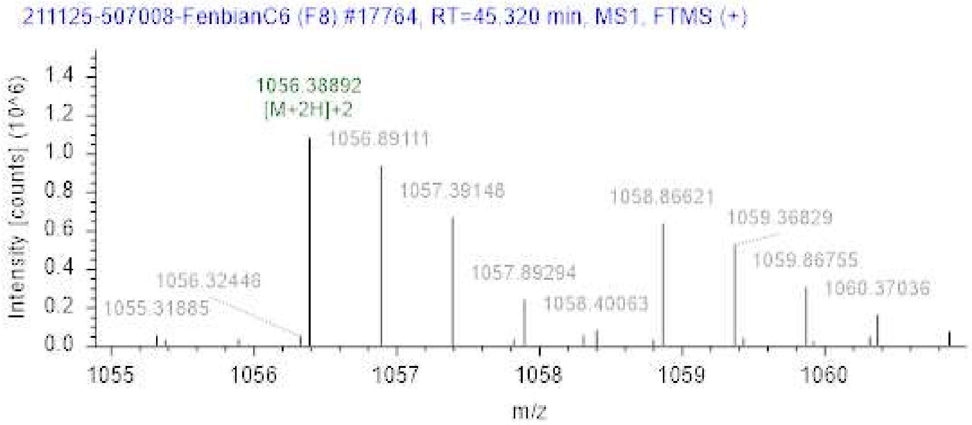

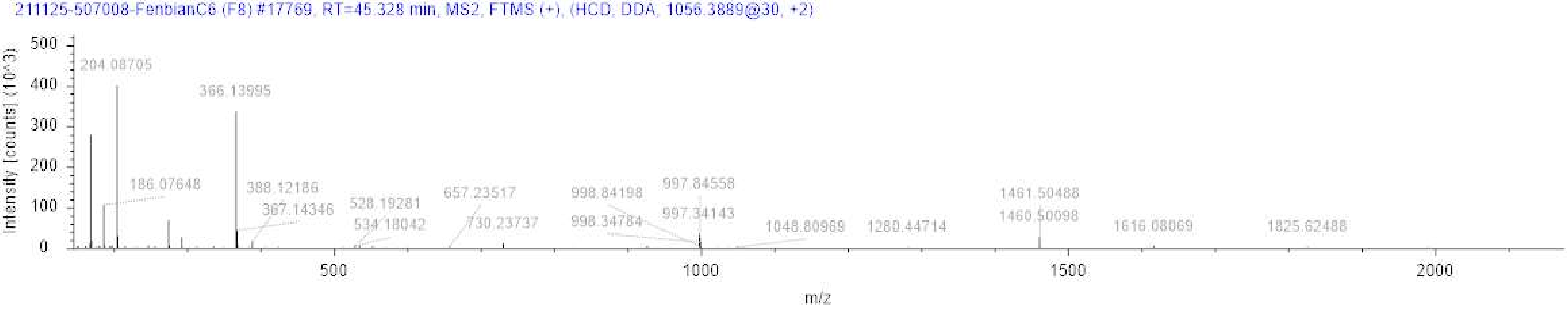

© Reported with Compound Discoverer 3.3

Structure	Name	RT [min]	Formula	Calc. MW	Areas
n/a		44.86	n/a	2128.80874	5.90e6

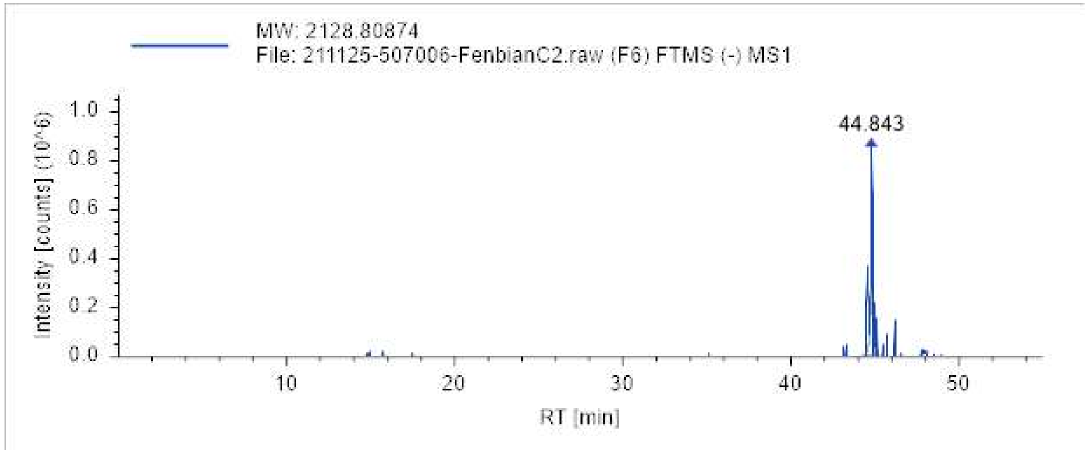

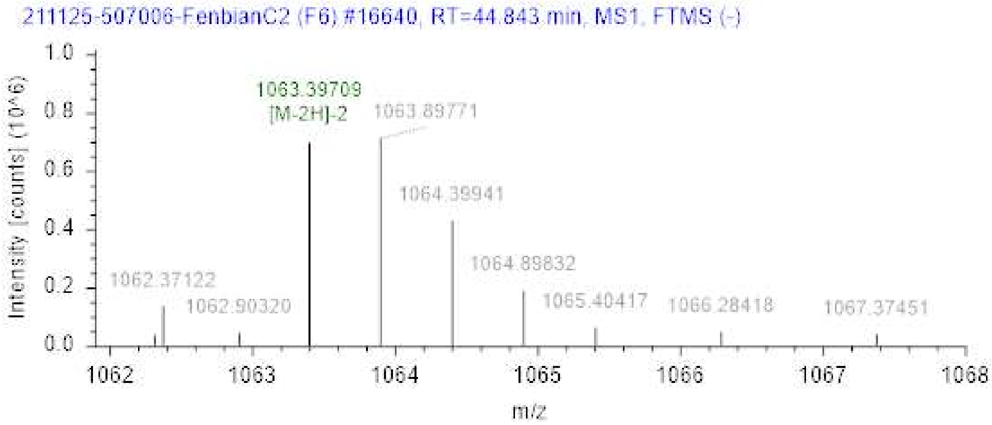

© Reported with Compound Discoverer 3.3

Structure	Name	RT [min]	Formula	Calc. MW	Areas
n/a		46.17	n/a	2128.80977	3.89e7 8.64e6 2.84e8 1.12e8

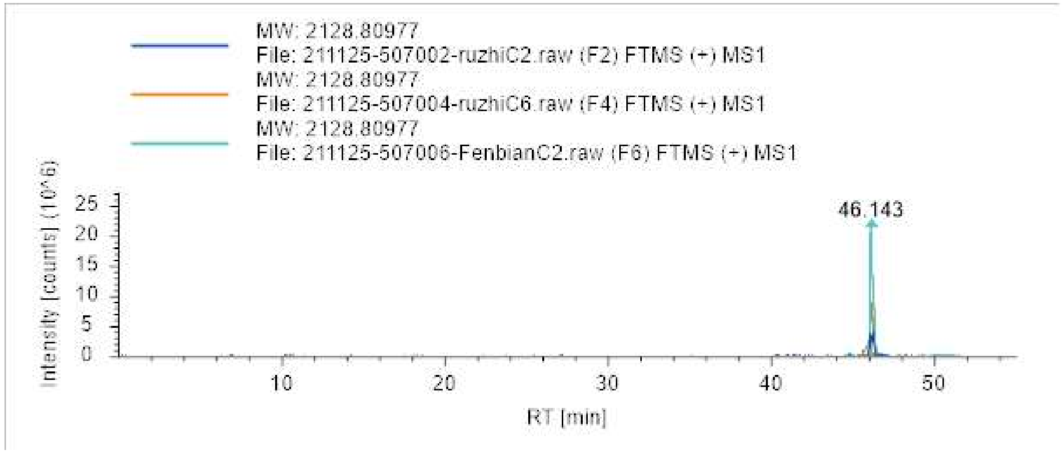

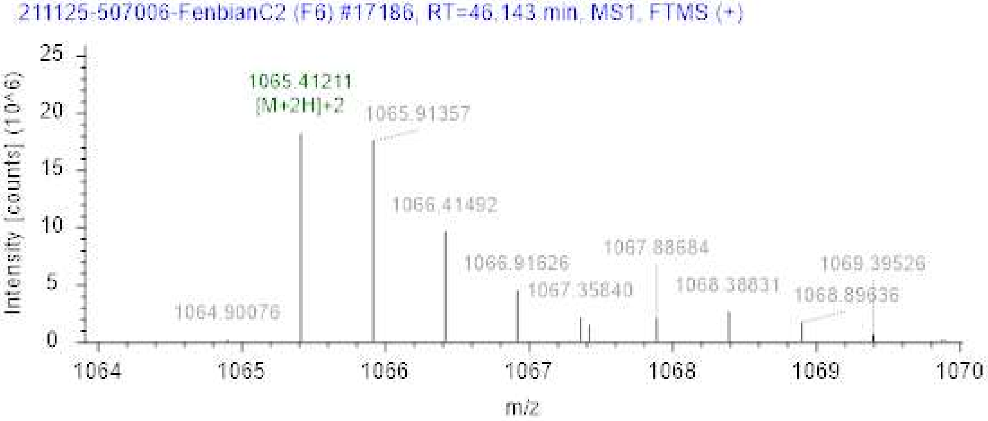

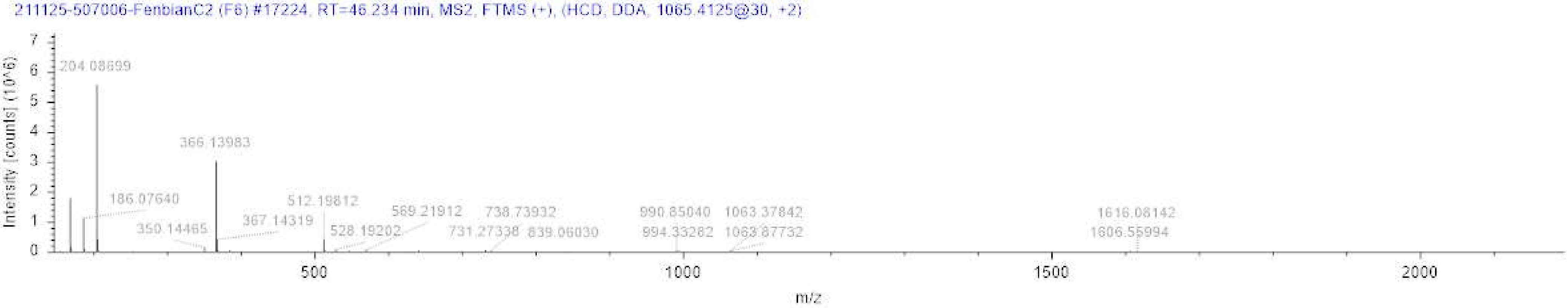

© Reported with Compound Discoverer 3.3

Structure	Name	RT [min]	Formula	Calc. MW	Areas
n/a		45.78	n/a	2151.78579	2.13e7

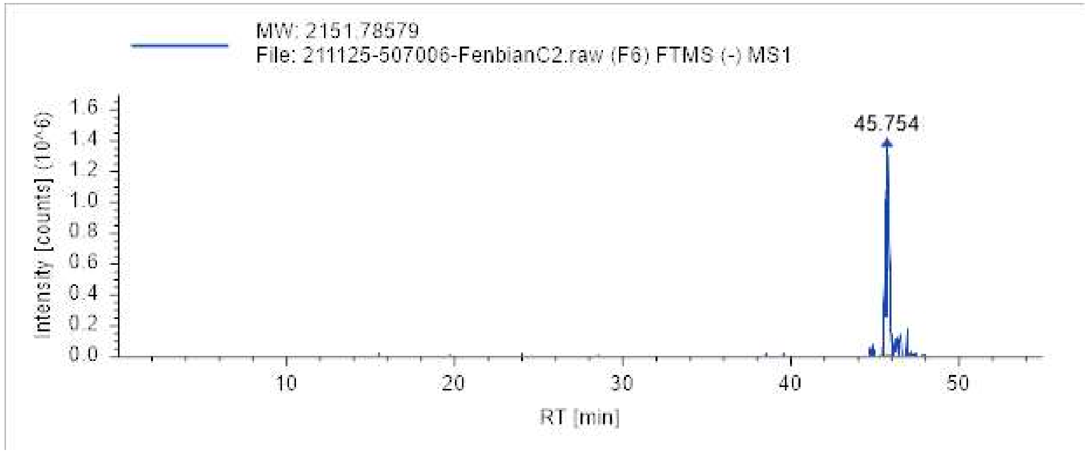

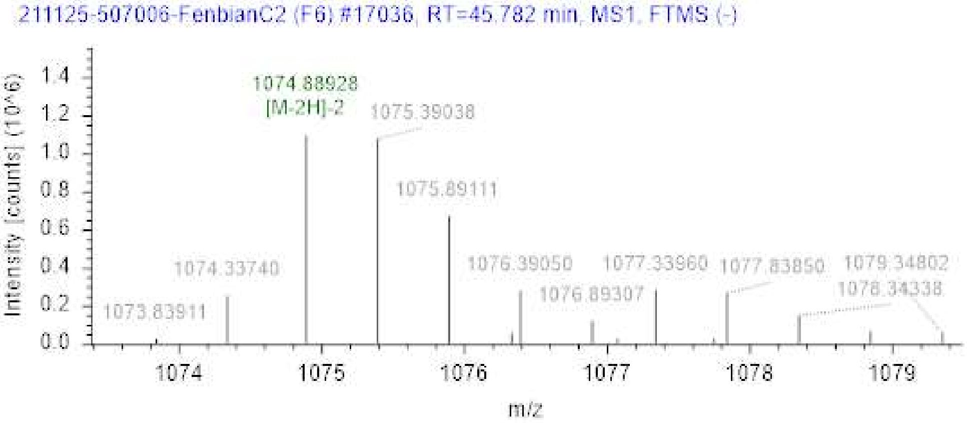

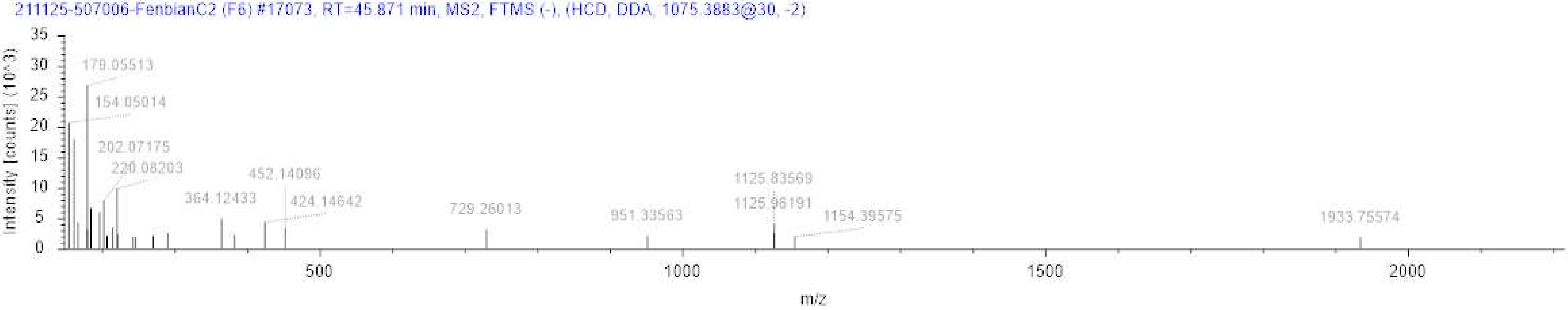

© Reported with Compound Discoverer 3.3

Compounds

Structure	Name	RT [min]	Formula	Calc. MW	Areas
n/a		44.93	n/a	2165.76479	3.84e7; 1.01e7; 3.25e8

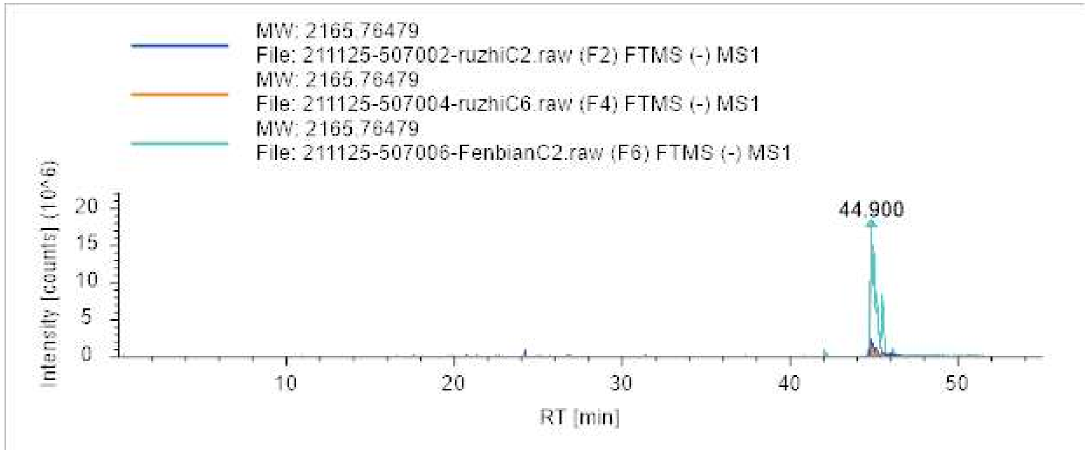

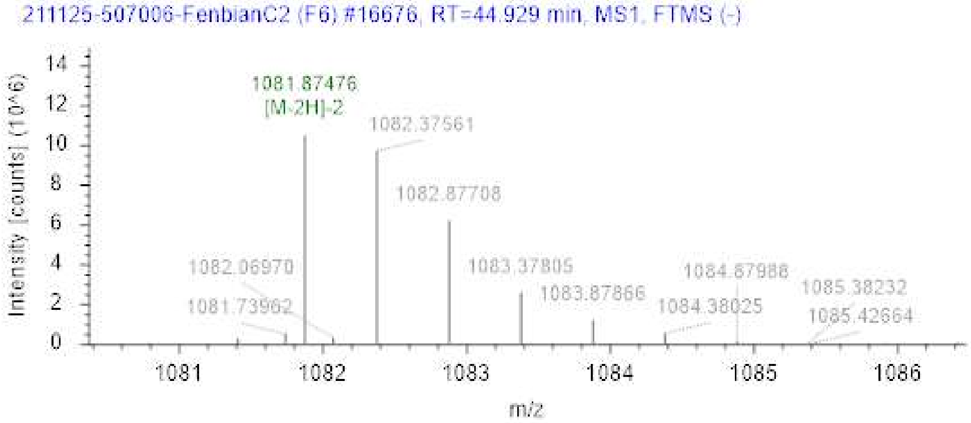

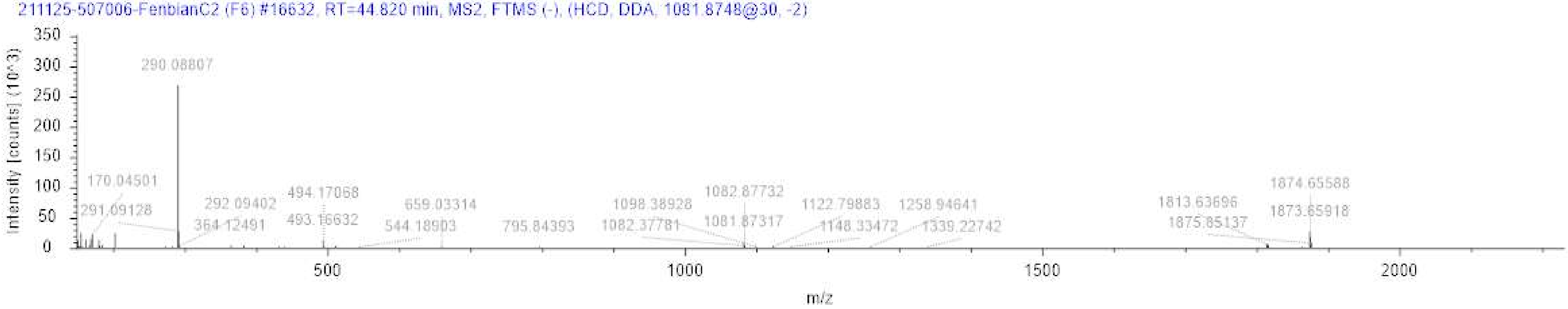

© Reported with Compound Discoverer 3.3

Compounds

Structure	Name	RT [min]	Formula	Calc. MW	Areas
n/a		45.86	n/a	2166.78347	5.34e7

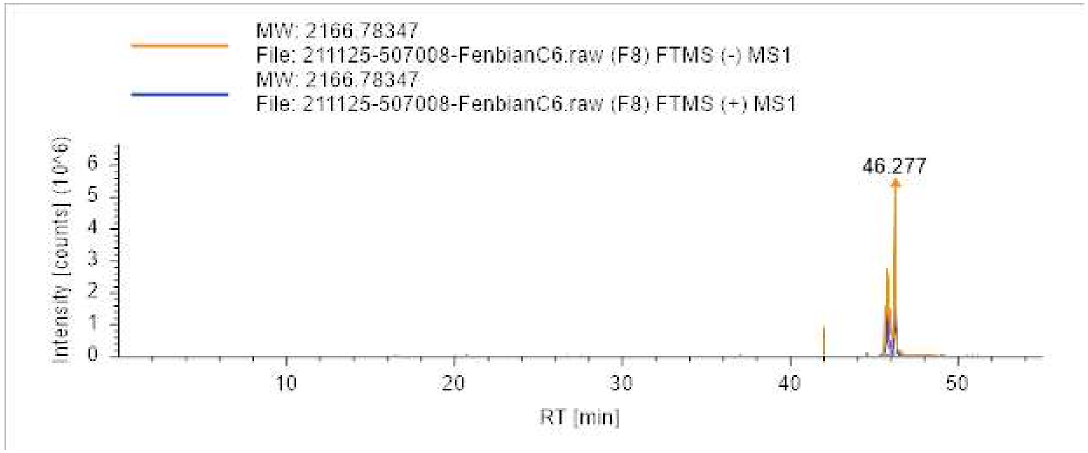

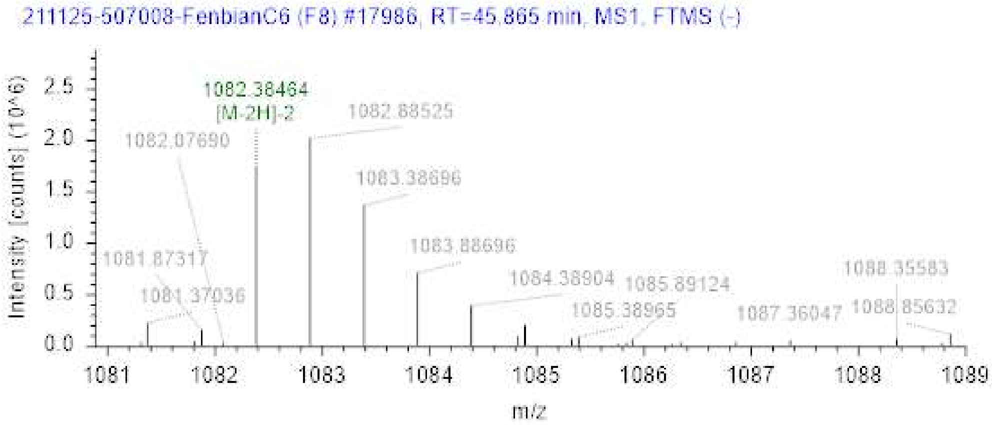

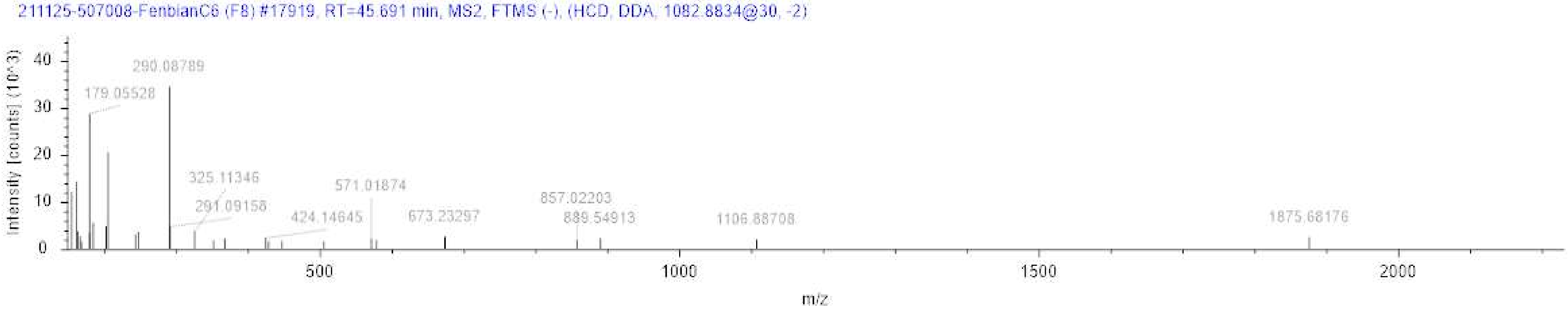

© Reported with Compound Discoverer 3.3

Compounds

Structure	Name	RT [min]	Formula	Calc. MW	Areas
n/a		45.72	n/a	2167.77752	2.10e7 1.40e7

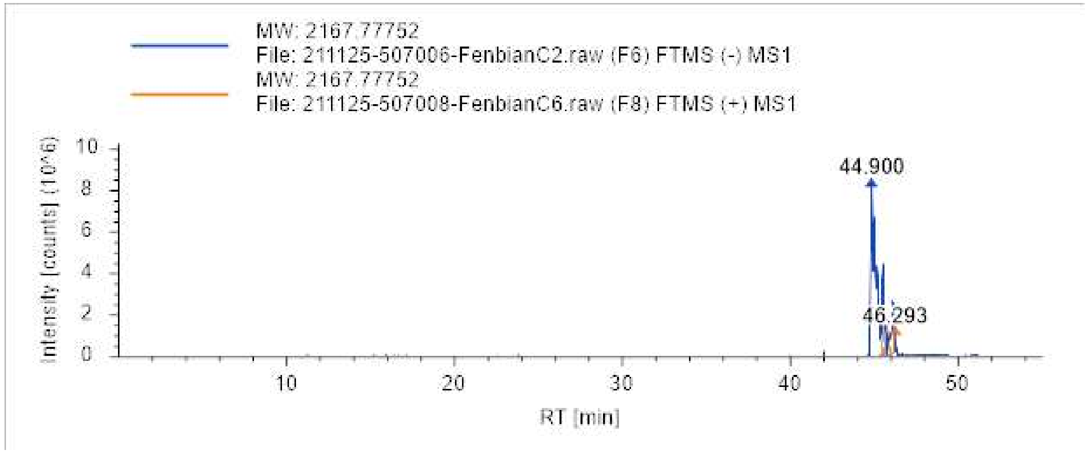

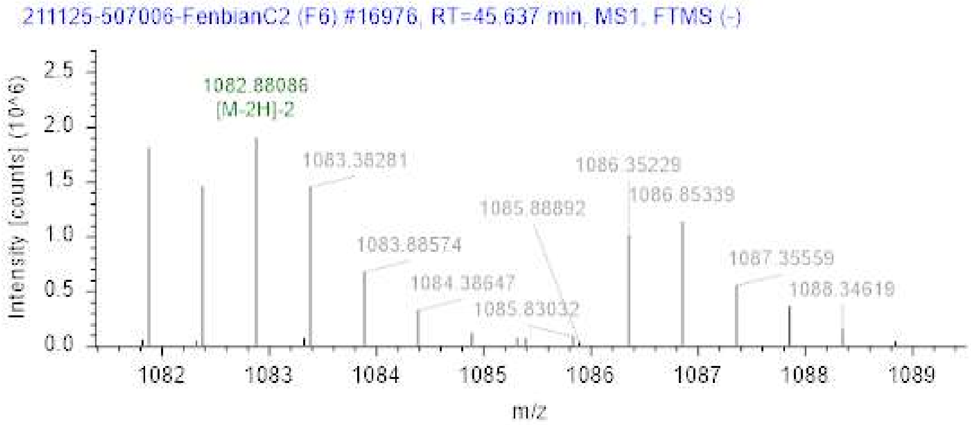

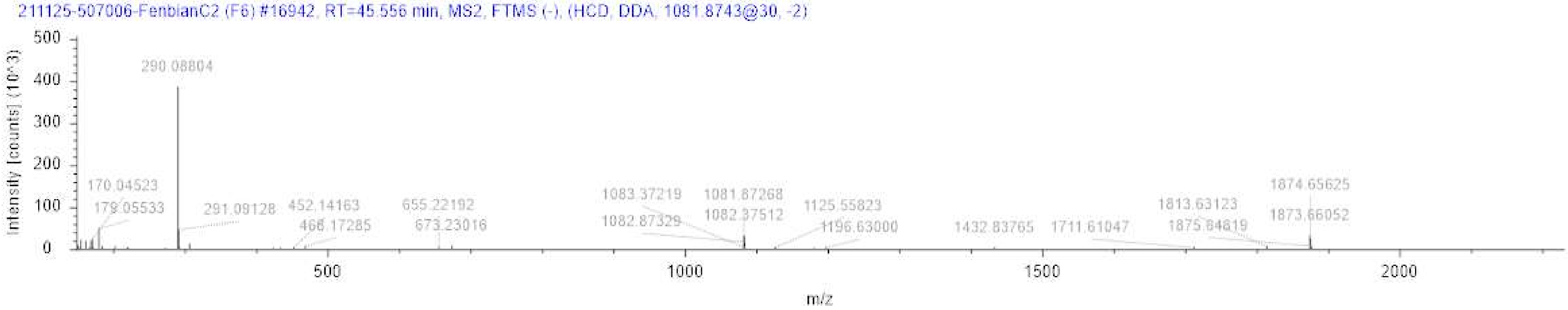

© Reported with Compound Discoverer 3.3

Structure	Name	RT [min]	Formula	Calc. MW	Areas
n/a		45.82	n/a	2168.78525	1.18e7

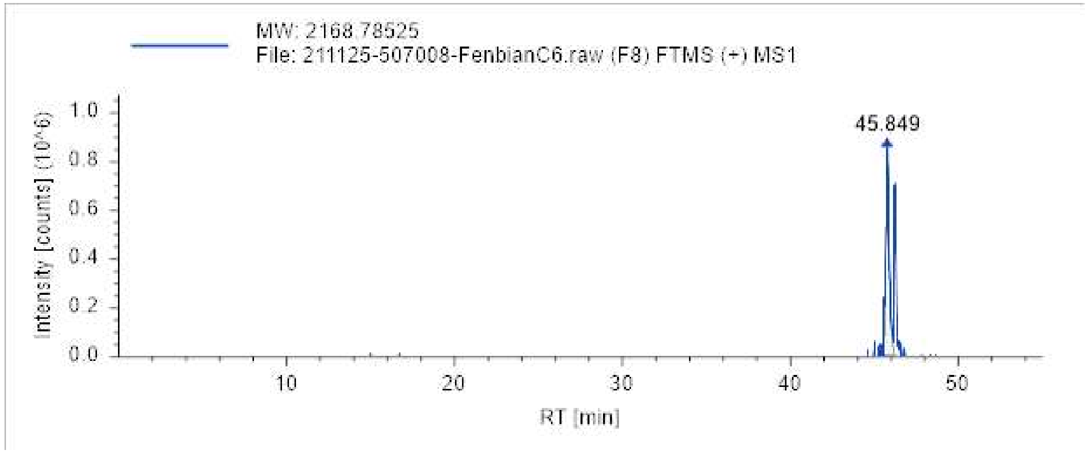

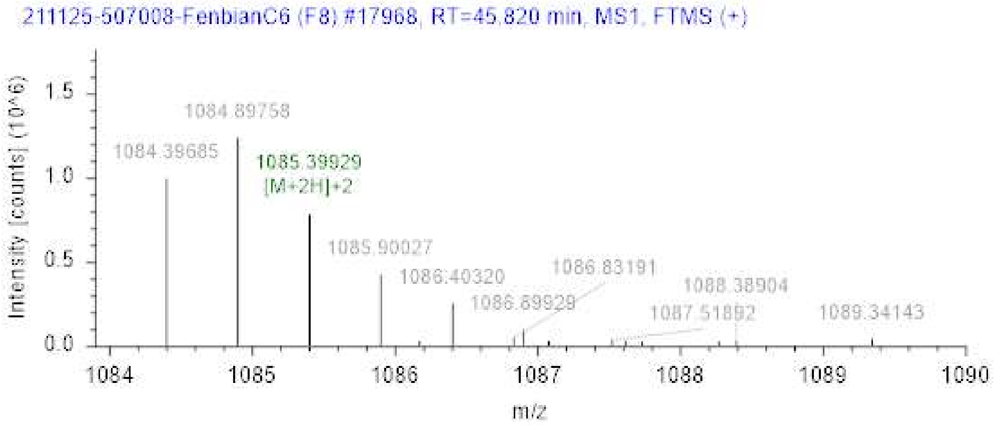

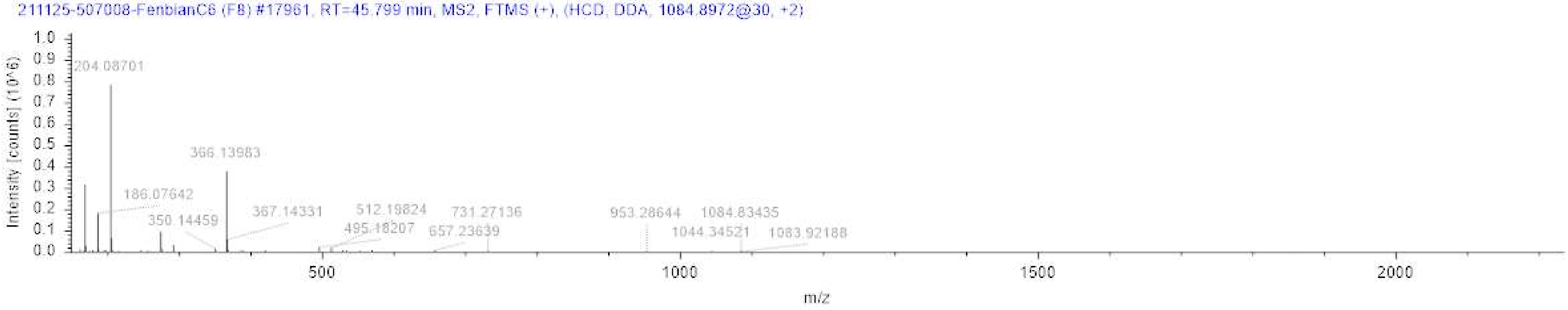

© Reported with Compound Discoverer 3.3

Compounds

Structure	Name	RT [min]	Formula	Calc. MW	Areas
n/a		46.18	n/a	2189.78457	2.56e7

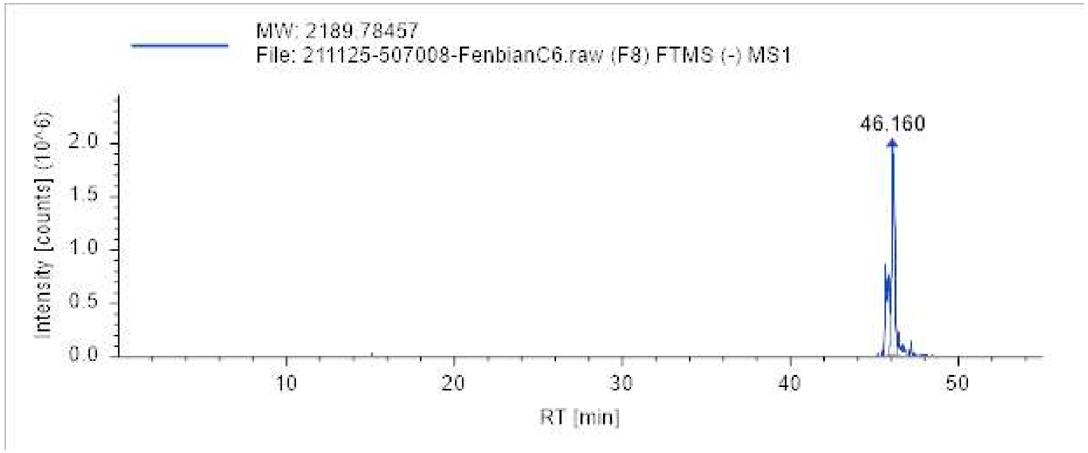

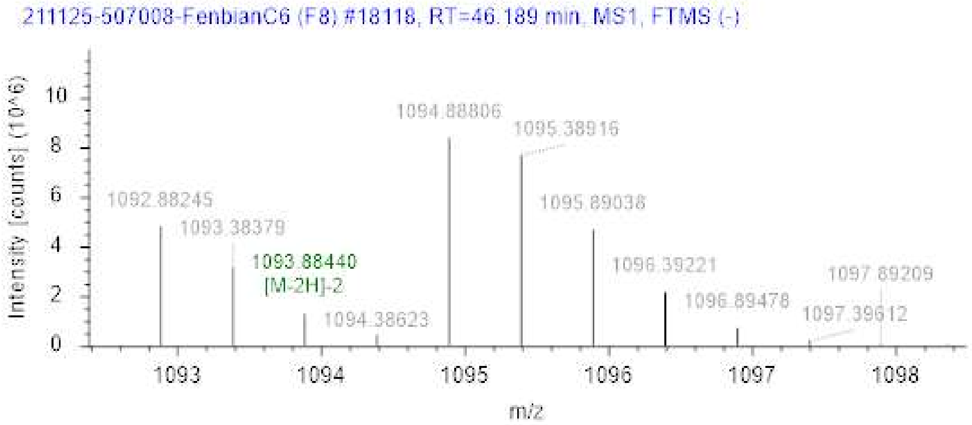

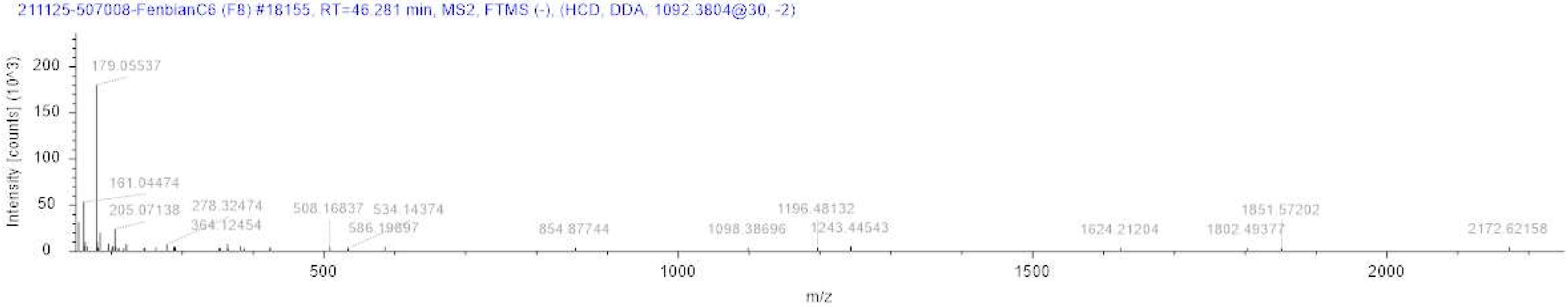

© Reported with Compound Discoverer 3.3

Compounds

Structure	Name	RT [min]	Formula	Calc. MW	Areas
n/a		47.05	n/a	2192.81156	1.66e7

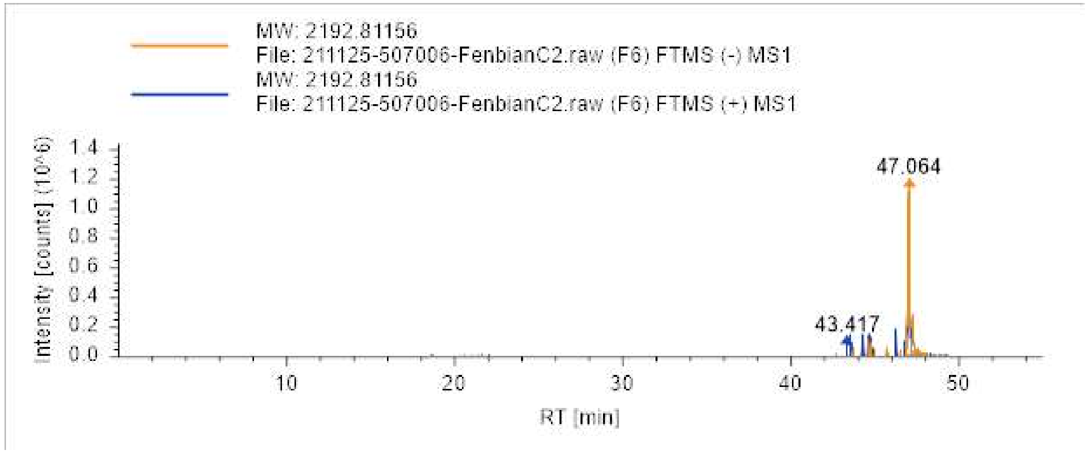

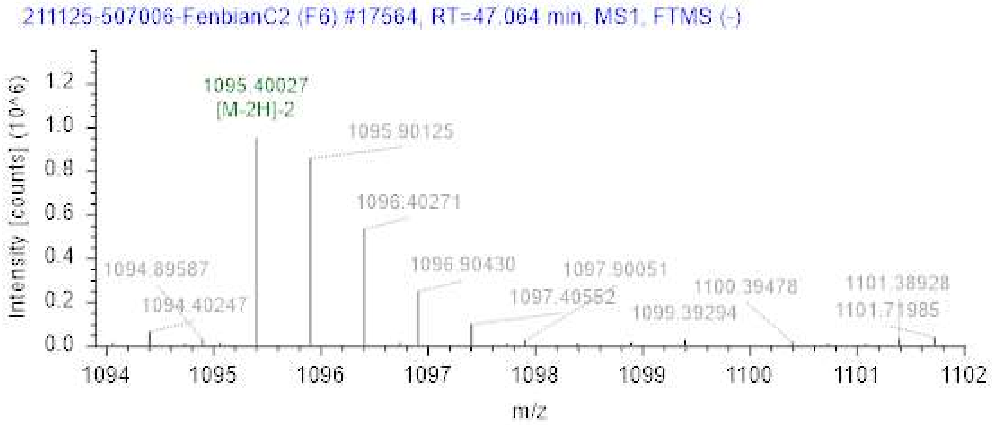

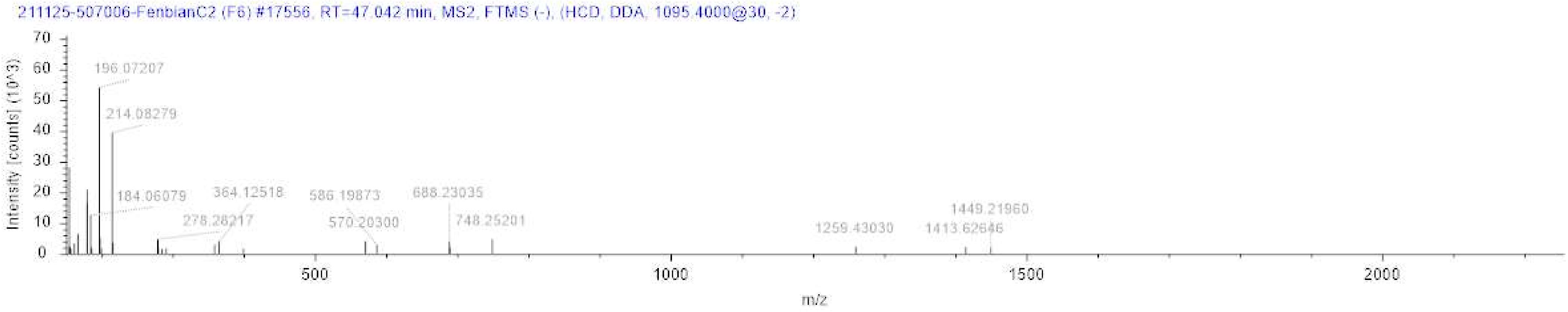

© Reported with Compound Discoverer 3.3

Compounds

Structure	Name	RT [min]	Formula	Calc. MW	Areas
n/a		47.04	n/a	2194.81973	4.18e6

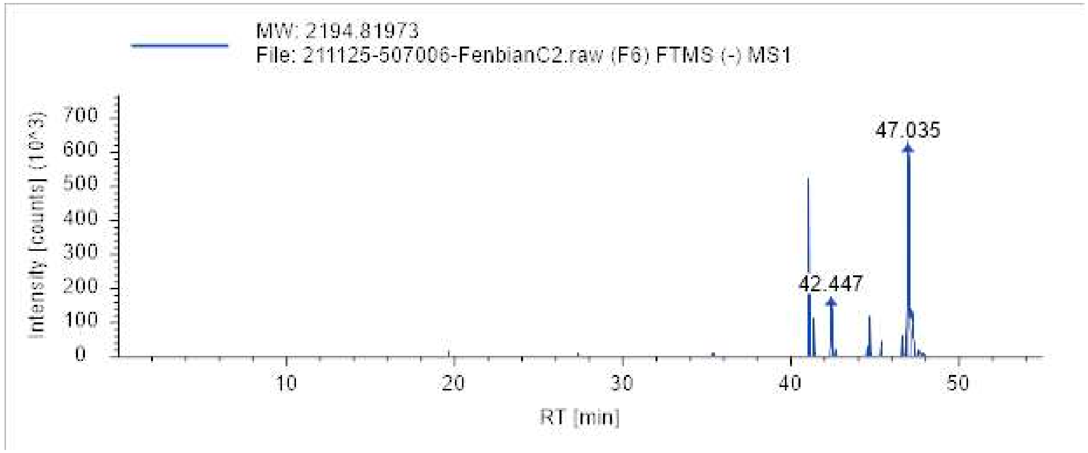

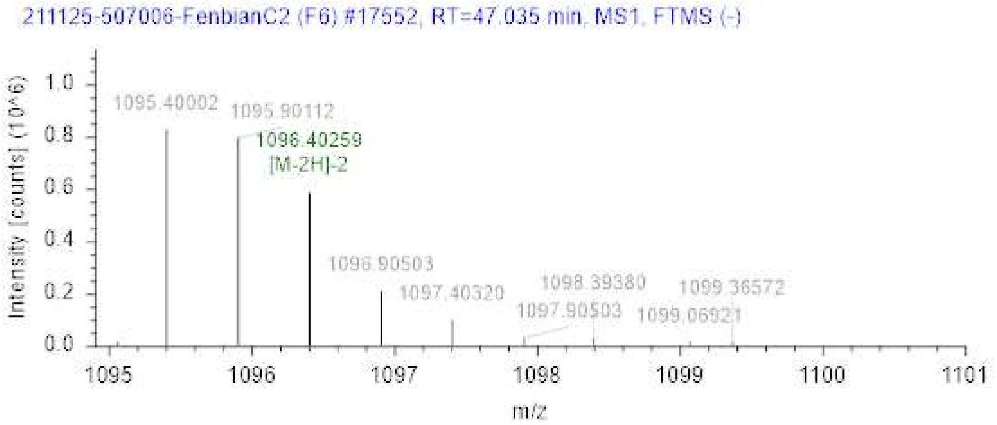

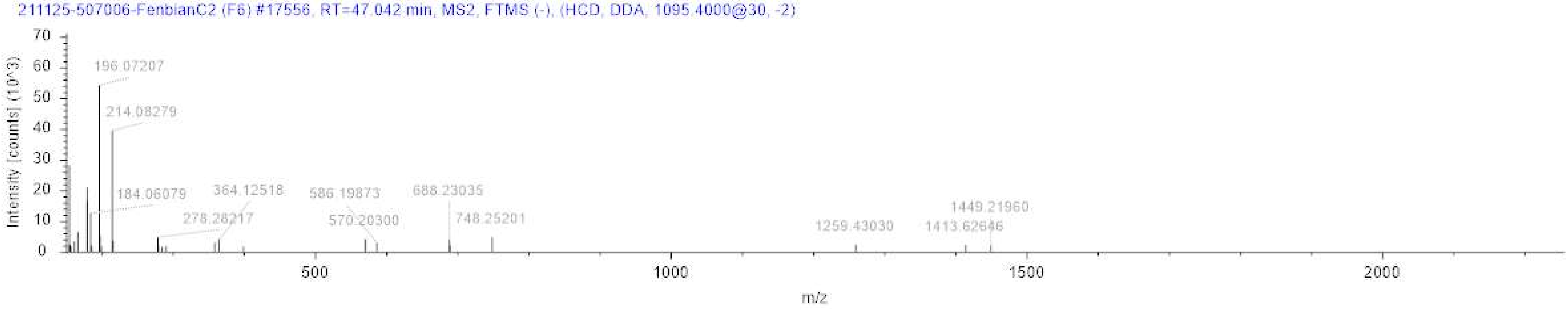

© Reported with Compound Discoverer 3.3

Structure	Name	RT [min]	Formula	Calc. MW	Areas
n/a		44.82	n/a	2222.78565	1.05e8 1.75e7

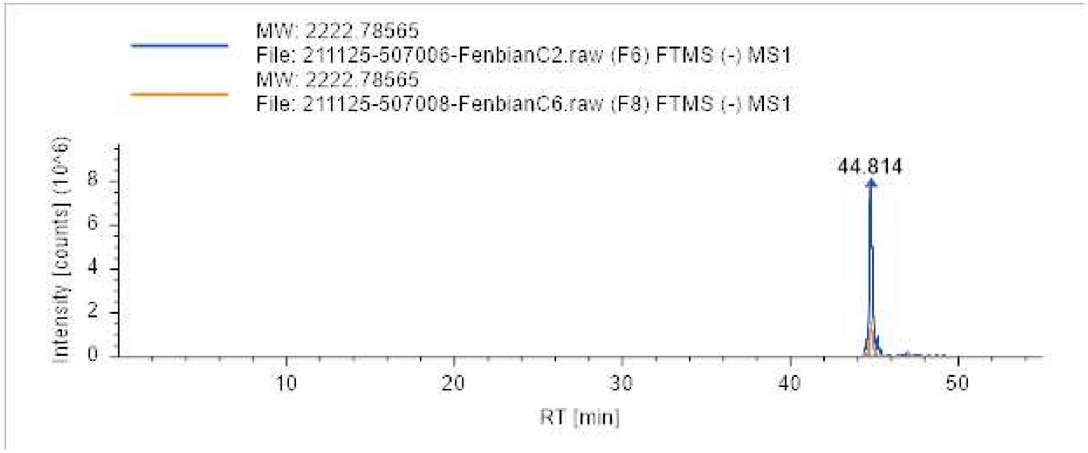

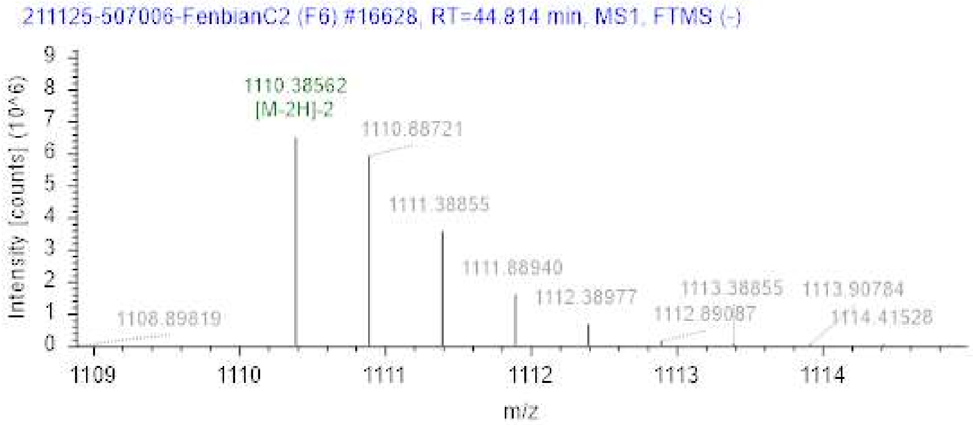

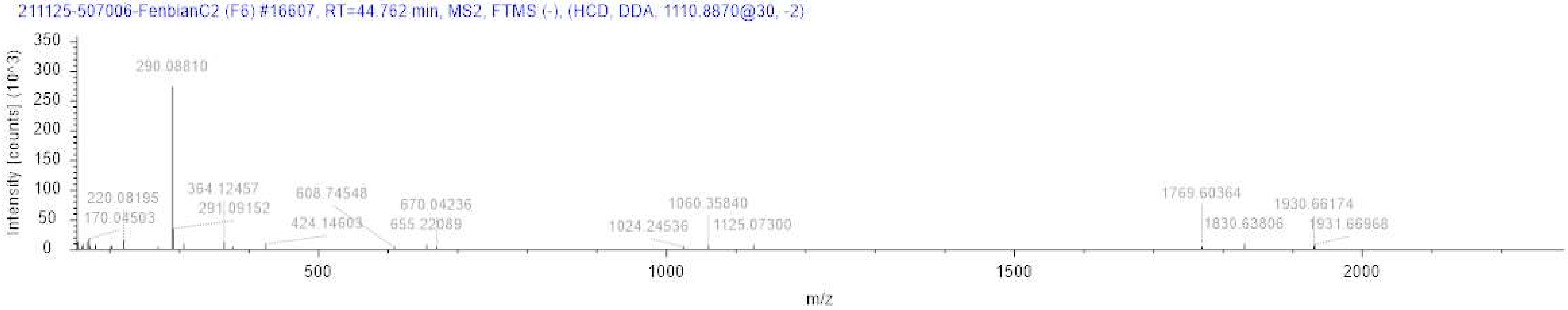

© Reported with Compound Discoverer 3.3

Structure	Name	RT [min]	Formula	Calc. MW	Areas
n/a		45.86	n/a	2239.79717	2.44e7; 4.23e7

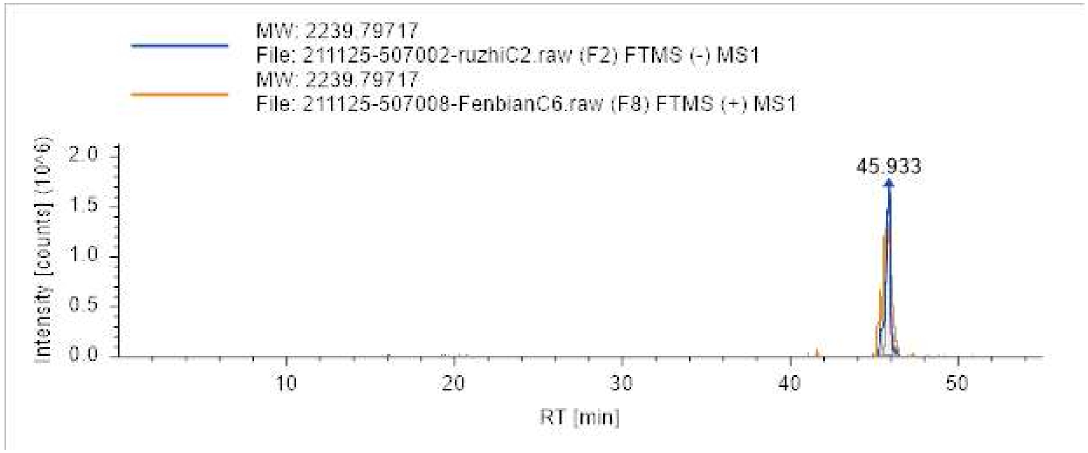

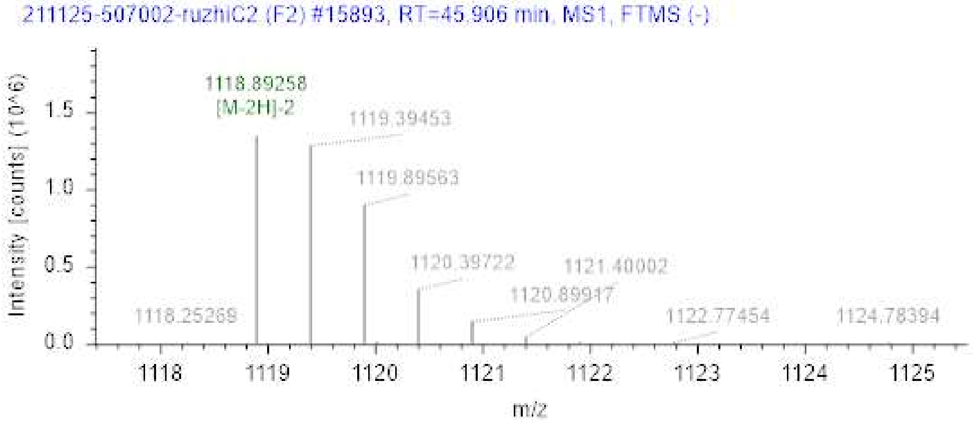

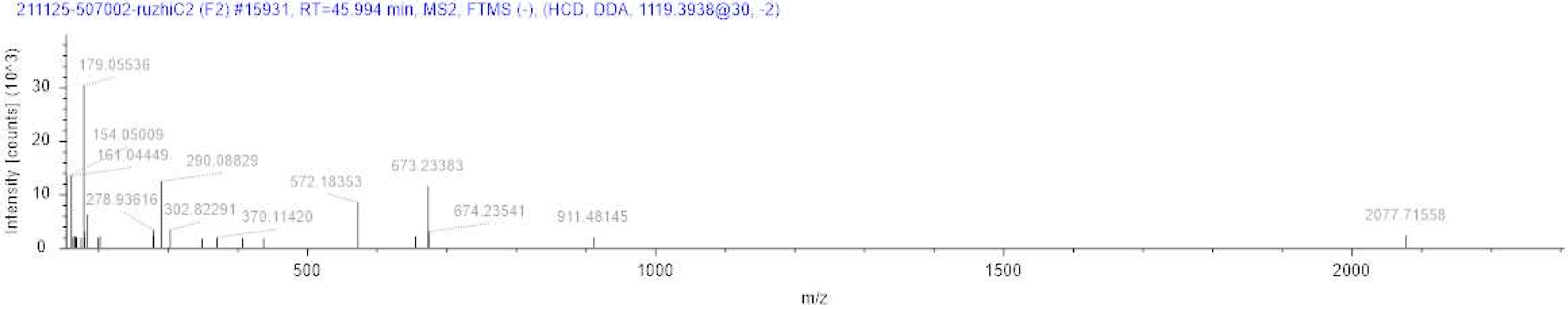

© Reported with Compound Discoverer 3.3

Compounds

Structure	Name	RT [min]	Formula	Calc. MW	Areas
n/a		45.46	n/a	2239.80410	5.29e7

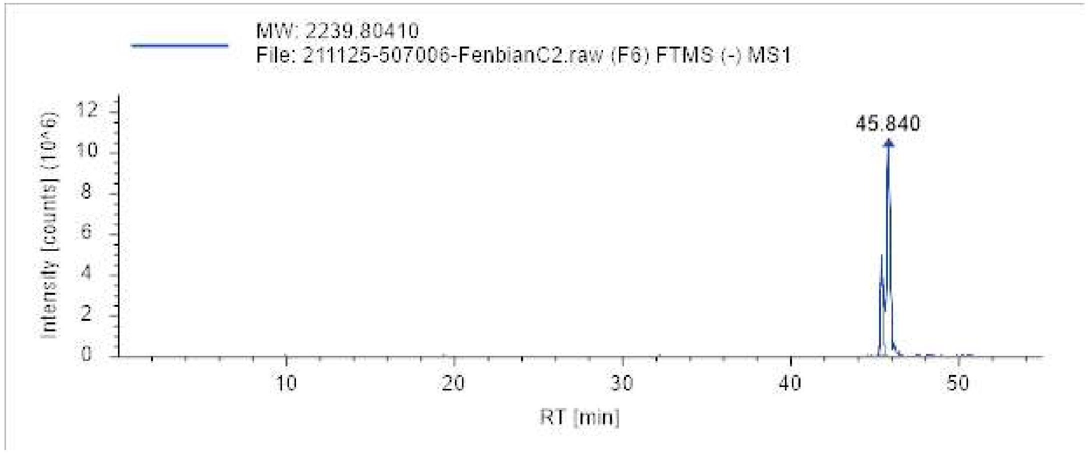

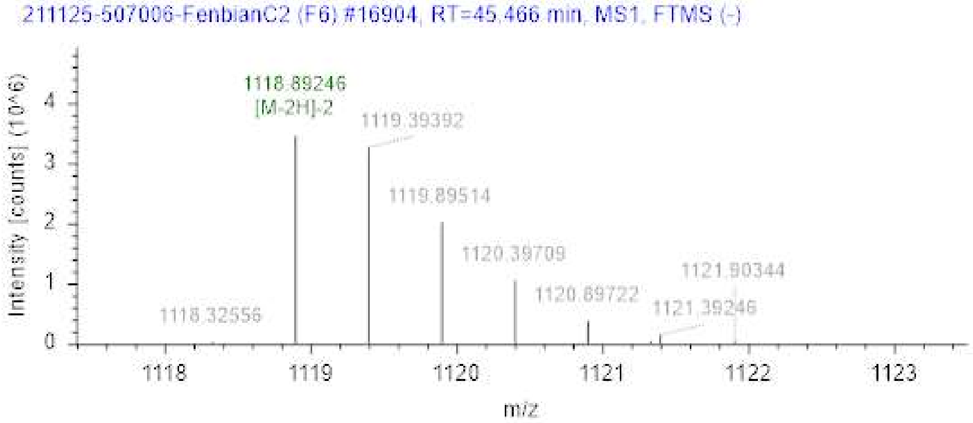

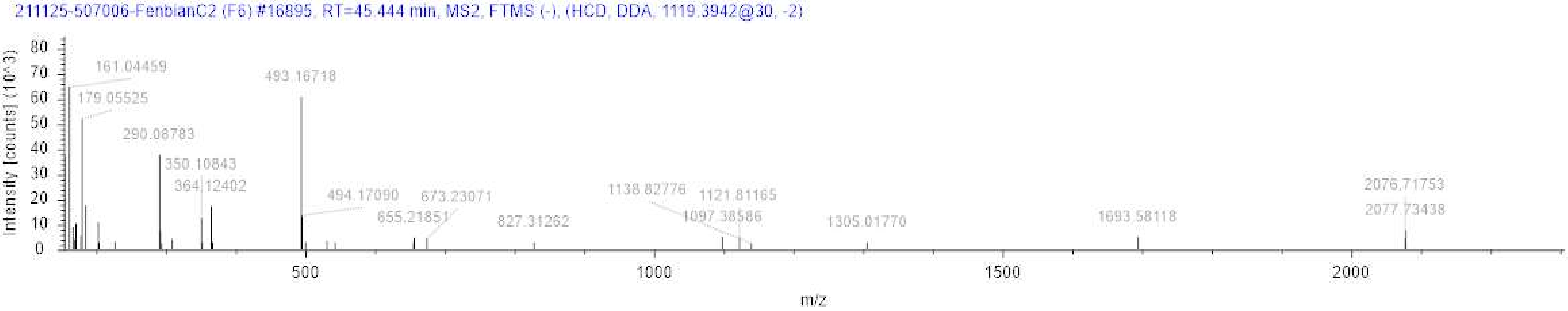

© Reported with Compound Discoverer 3.3

Structure	Name	RT [min]	Formula	Calc. MW	Areas
n/a		46.82	n/a	2240.82457	1.59e7 3.79e7 1.74e7 4.36e8 1.06e8

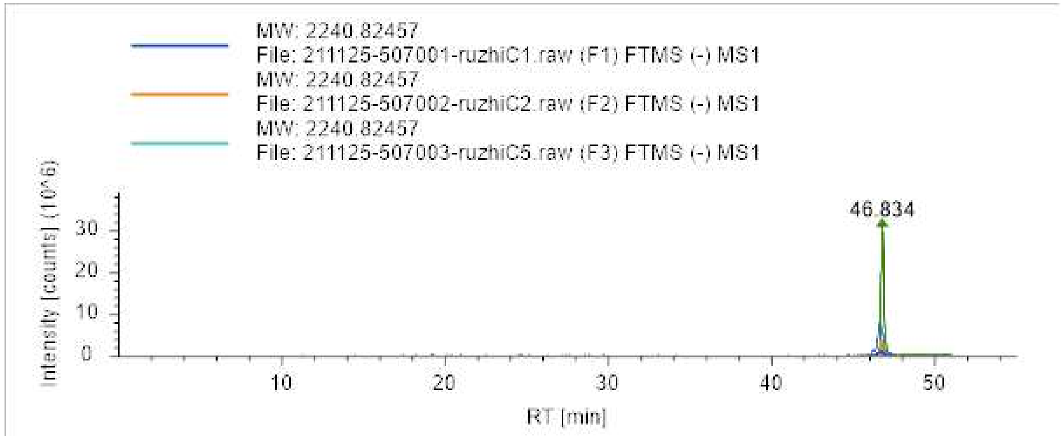

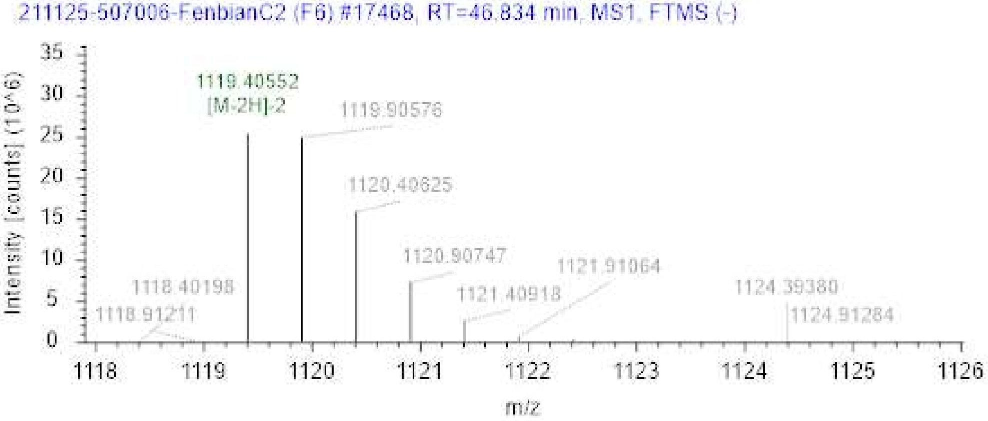

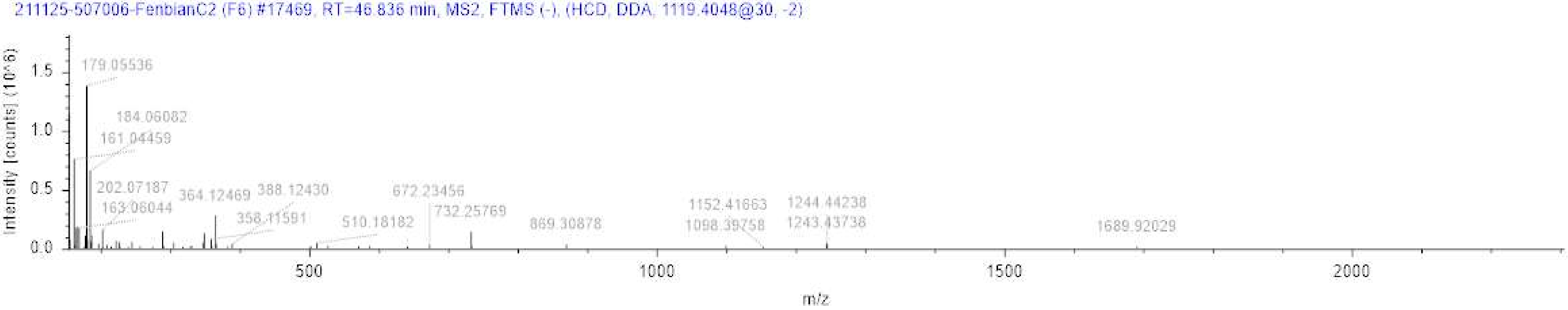

© Reported with Compound Discoverer 3.3

Compounds

Structure	Name	RT [min]	Formula	Calc. MW	Areas
n/a		46.83	n/a	2241.82581	1.35e7 | 3.00e7 | 9.29e6 | | | 3.13e8 | | |

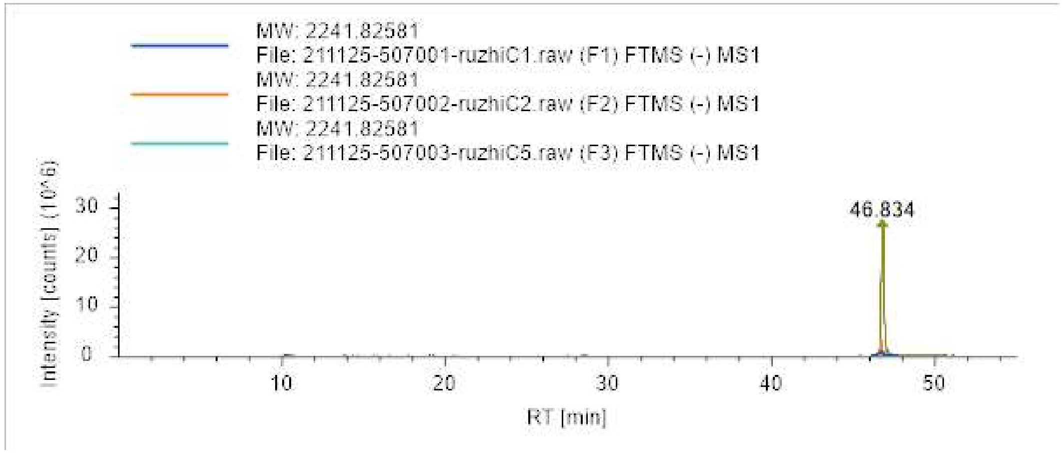

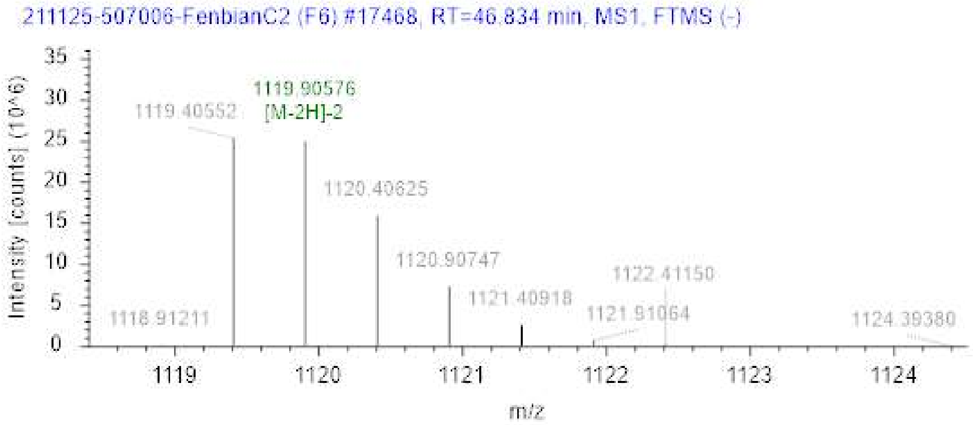

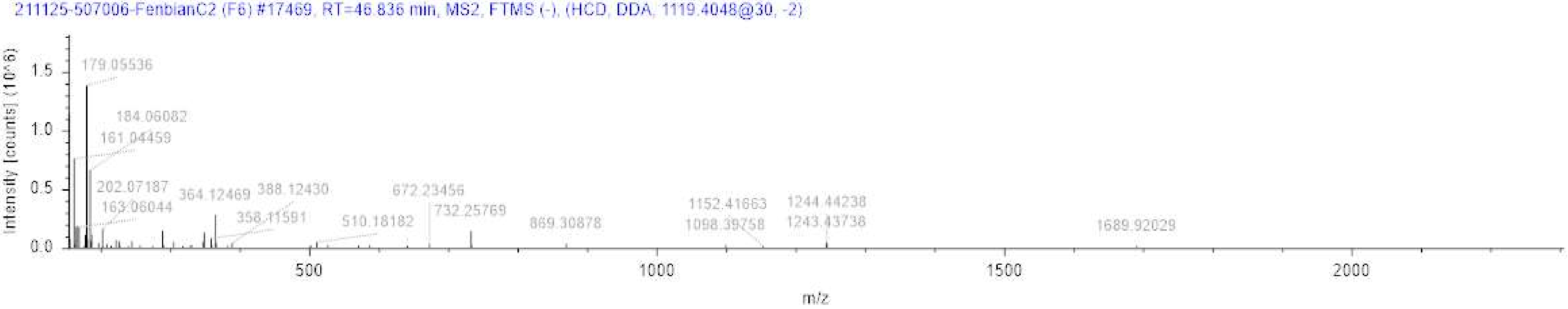

© Reported with Compound Discoverer 3.3

Structure	Name	RT [min]	Formula	Calc. MW	Areas
n/a		46.15	n/a	2254.75933	1.86e7; 2.06e7

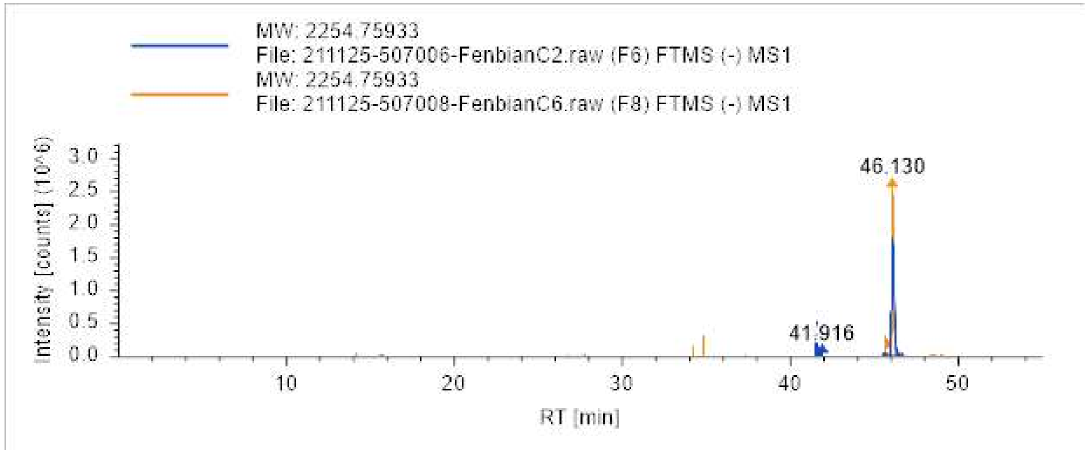

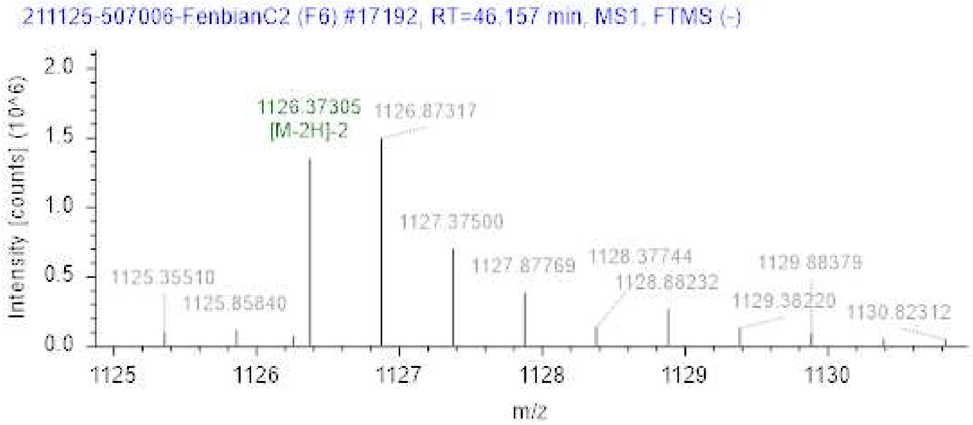

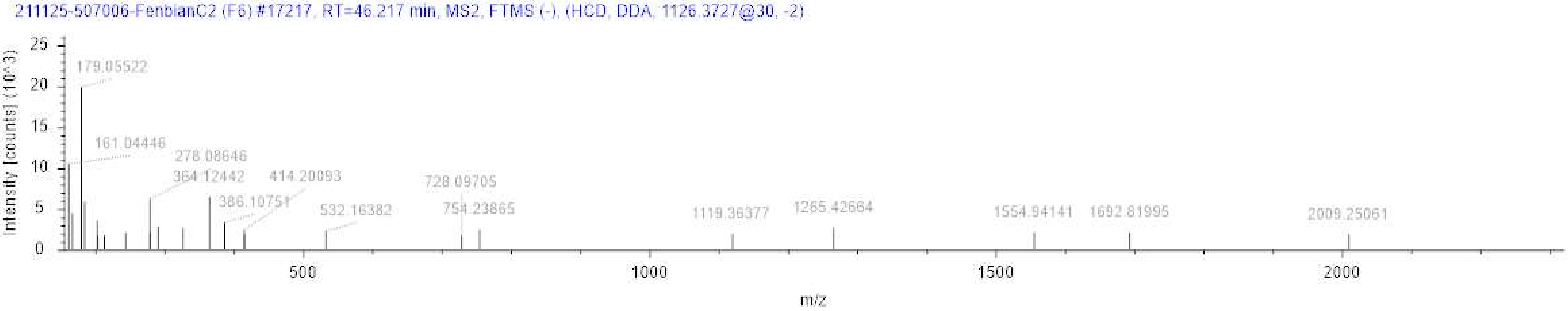

© Reported with Compound Discoverer 3.3

Compounds

Structure	Name	RT [min]	Formula	Calc. MW	Areas
n/a		46.82	n/a	2263.81094	1.70e7

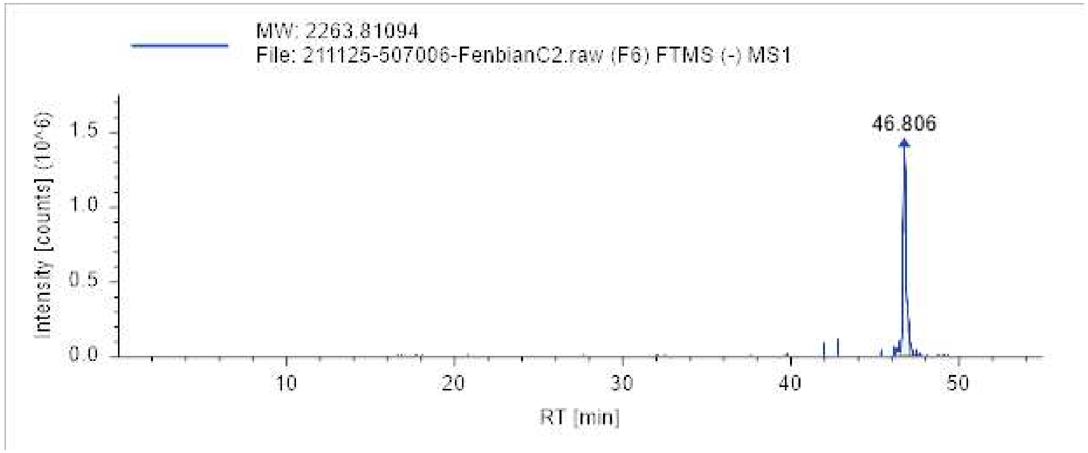

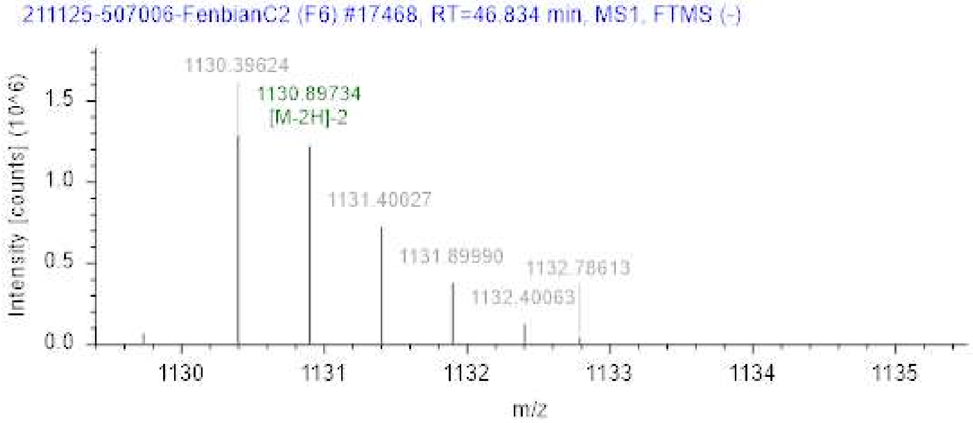

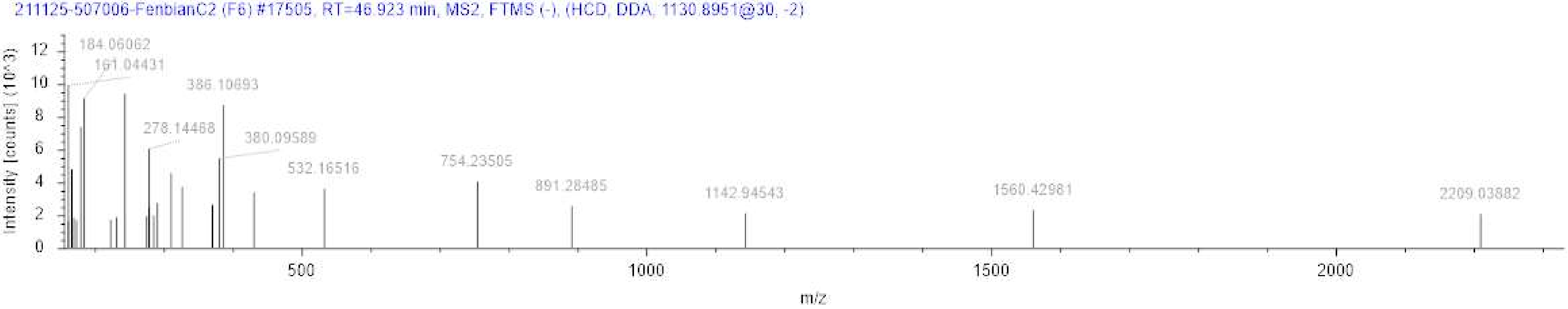

© Reported with Compound Discoverer 3.3

Compounds

Structure	Name	RT [min]	Formula	Calc. MW	Areas
n/a		46.58	n/a	2274.86997	5.99e7

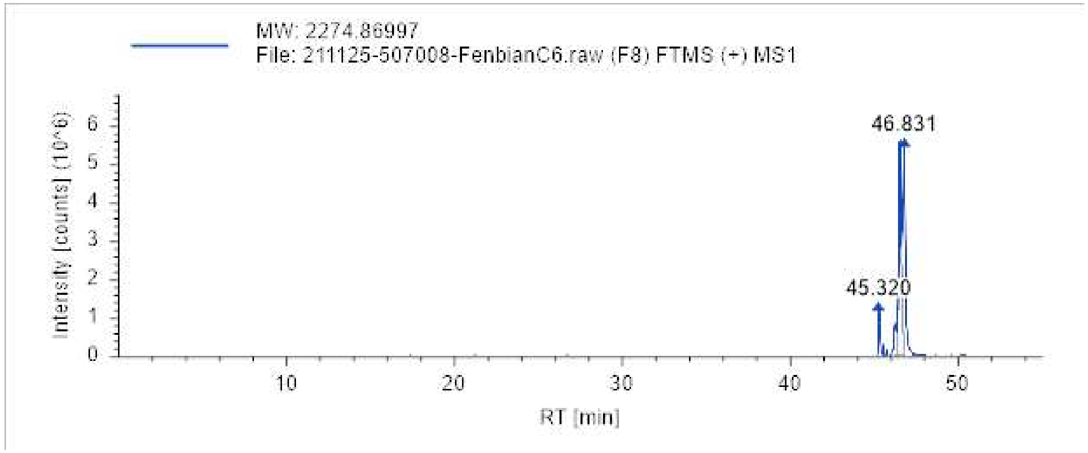

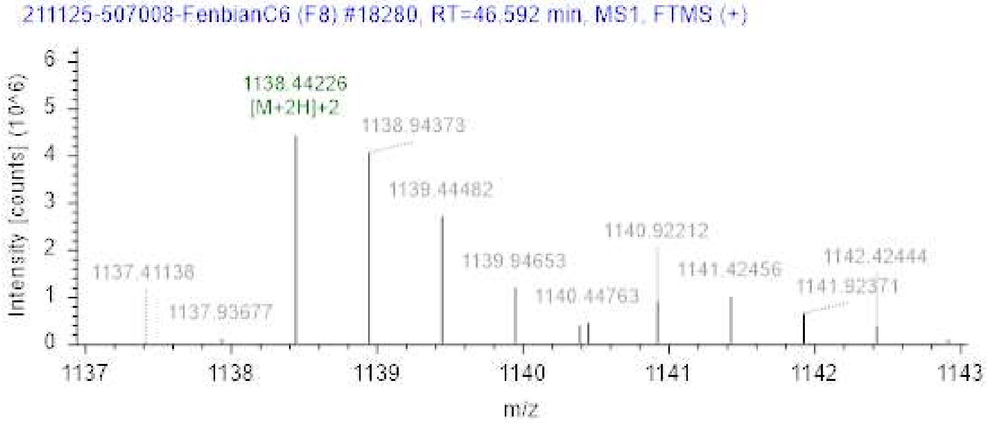

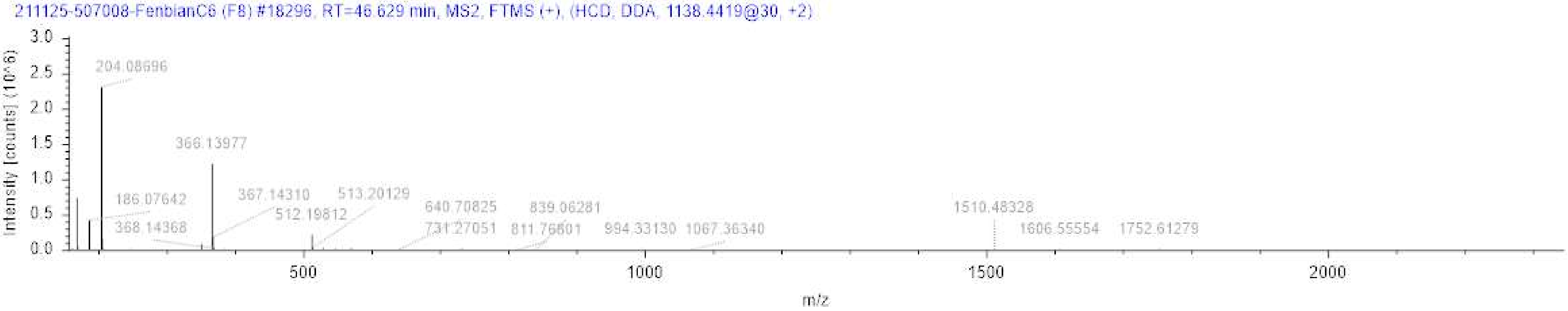

© Reported with Compound Discoverer 3.3

Structure	Name	RT [min]	Formula	Calc. MW	Areas
n/a		46.44	n/a	2297.84317	3.25e7

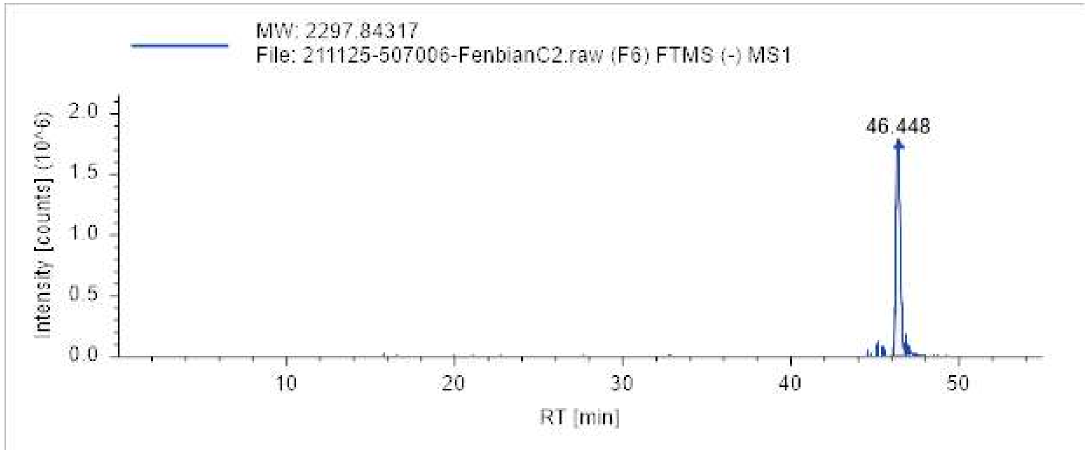

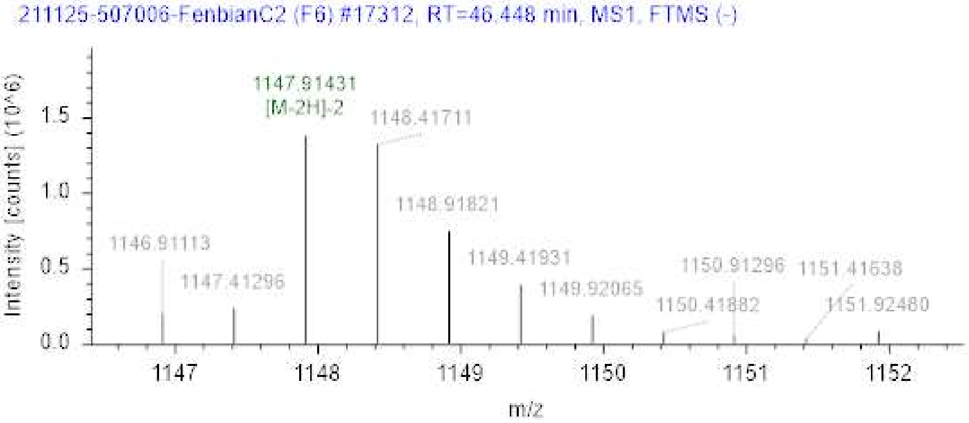

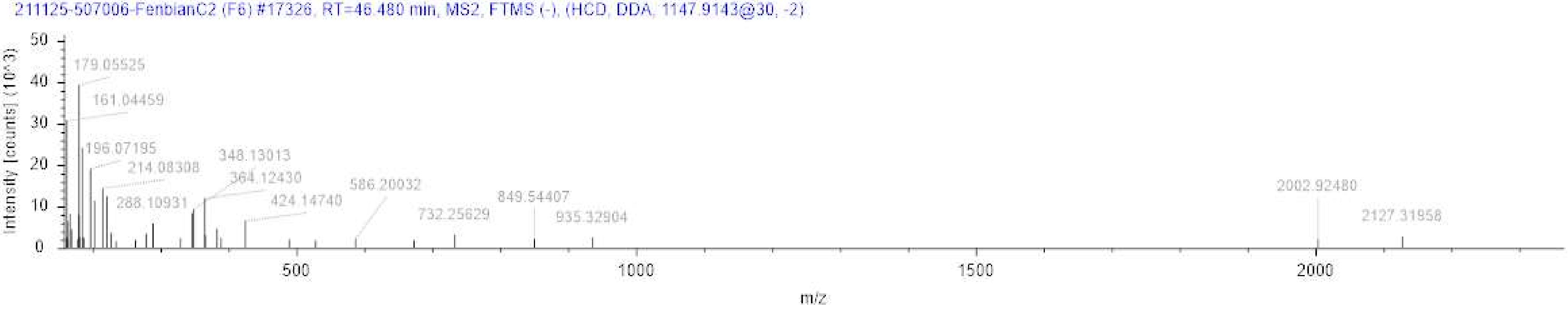

© Reported with Compound Discoverer 3.3

Structure	Name	RT [min]	Formula	Calc. MW	Areas
n/a		46.36	n/a	2313.83980	6.69e7; 4.61e7

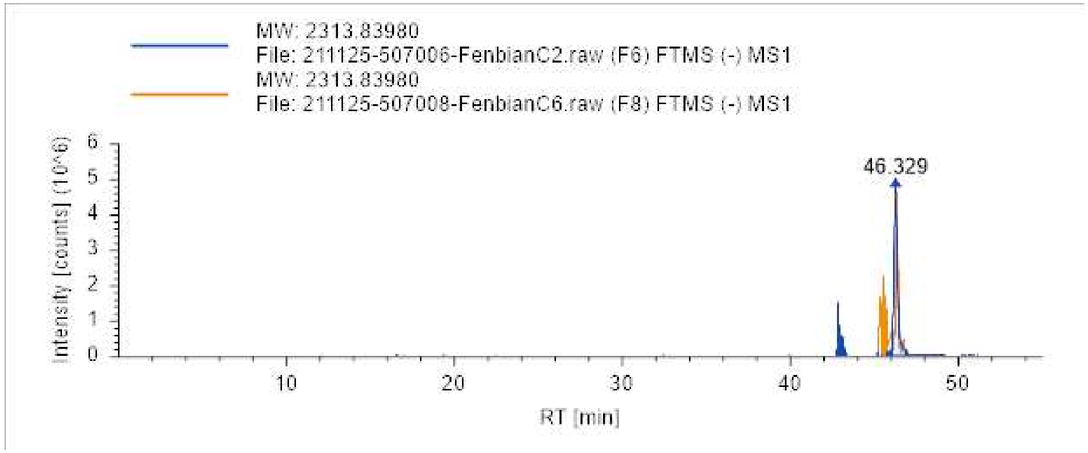

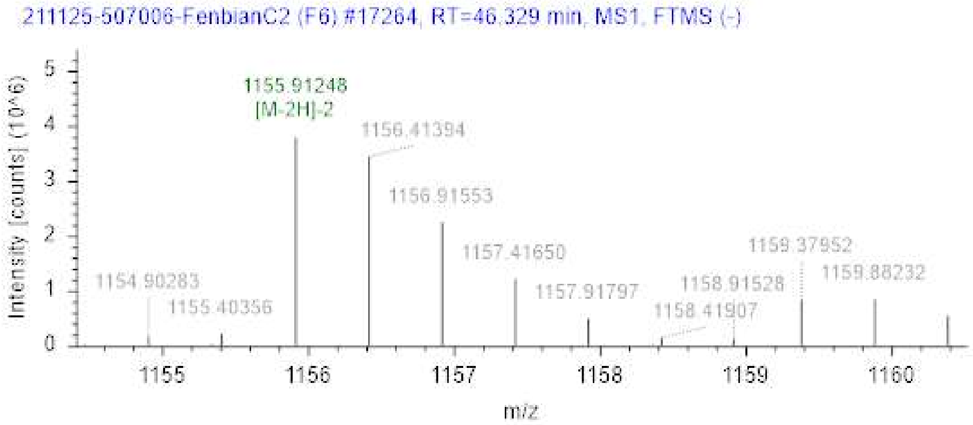

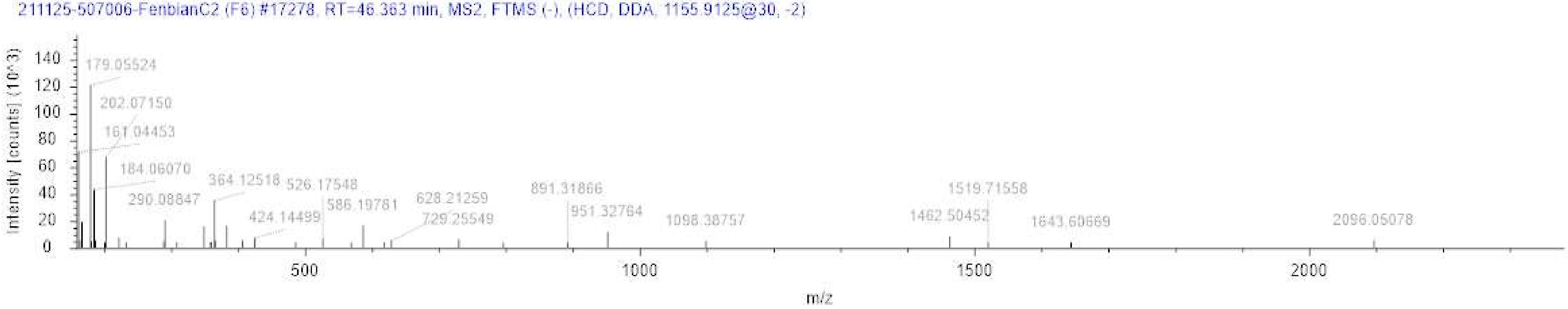

© Reported with Compound Discoverer 3.3
