# Supplementary material for: Comparative Analysis of Oligosaccharides in Breast Milk and Feces of Breast-Fed Infants by Using LC-QE-HF-MS: A Communication
Source: Nutrients. 2023 Feb 9;15(4):888. doi: 10.3390/nu15040888 (PMC9963387; doi:10.3390/nu15040888)
Supplement: Supplementary file 1 [file nutrients-15-00888-s001.zip › Supplementary Table S1.pdf]

Supplementary Table S1 OS identified and their peak area in breast milk of four women and feces of the women's babies

Composition	RT (min)	Mass		Ion	Peak area (×10^6)							
		Found	Cal		M1	F1	M2	F2	M3	F3	M4	F4
Hex2Fuc1	25.725	488.174	488.17301	H-	219.458	2887.122	0	150.489	109.871	657.914	76.532	2567.812
Hex2Fuc1	27.995	488.174	488.17323	H-	12517.335	0	0	30.802	7399.013	362.472	17748.014	74593.674
Hex2Fuc1	29.175	488.174	488.17286	H-	6450.043	751.868	26788.538	49590.884	21981.847	0	5774.232	28352.463
Hex2Fuc1	30.154	488.174	488.17372	H-	11416.400	690.603	27192.821	78800.155	23841.165	0	10865.295	6286.180
Hex2Fuc1	32.328	488.174	488.17357	H-	0	0	0	0	12.355	0	0	0
Hex2Fuc1	34.382	488.174	488.17252	H+	33.707	0	0	19.609	131.738	0	43.765	408.014
Hex2Fuc1	11.327	488.174	488.17164	H+	0	407.520	0	219.129	0	36.147	0	202.390
Hex2Fuc1	27.448	488.174	488.17346	H-	0	0	0	19.505	0	216.905	0	0
Hex2Fuc1	40.672	488.174	488.17386	H-	0	0	0	0	0	0	0	22.631
Hex3	24.121	504.169	504.16696	H+	0	0	0	0	942.852	0	912.418	0
Hex3	32.737	504.169	504.16871	H-	162.078	0	387.312	0	110.635	993.521	231.490	131.529
Hex3	7.24	504.169	504.1701	H+	0	2192.431	0	0	0	151.776	0	0
Hex3	17.567	504.169	504.16926	H-	0	0	0	0	0	26.094	0	0
Hex3	28.675	504.169	504.16929	H-	0	0	0	0	0	13.008	0	0
Hex3	30.849	504.169	504.16929	H-	0	0	0	0	0	16.415	0	0
Hex1HexNAc1Fuc1	26.474	529.2006	529.19886	H+	42.217	557.741	48.687	9460.326	89.975	0	0	1505.223
Hex1HexNAc1Fuc1	28.091	529.2006	529.19879	H+	0	0	0	5792.833	116.355	0	0	113.462
Hex1HexNAc1Fuc1	25.308	529.2006	529.19956	H-	0	1668.208	0	0	0	0	0	0
Hex1HexNAc1Fuc1	31.142	529.2006	529.20018	H+	0	0	0	0	0	0	0	20.790
Hex1HexNAc1Fuc1	42.253	529.2006	529.2005	H-	0	0	0	48.791	0	0	0	0
Hex2HexNAc1	38.3	545.1955	545.19487	H-	102.022	0	221.693	0	33.321	0	33.391	361.496
Hex2HexNAc1	40.616	545.1955	545.19541	H-	20.325	0	101.664	0	17.108	0	12.258	56.243
Hex2HexNAc1	28.448	545.1955	545.1942	H+	0	0	0	0	0	889.332	0	0

Hex2HexNAc1	29.638	545.1955	545.19485	H-	0	0	0	819.473	0	3827.781	0	0
Hex2HexNAc1	30.776	545.1955	545.19451	H+	0	11.941	0	185.871	0	0	0	371.069
Hex2HexNAc1	31.28	545.1955	545.19542	H+	0	0	0	0	0	0	0	90.255
Hex2HexNAc1	36.842	545.1955	545.19396	H+	0	0	0	48.165	0	0	0	0
Hex2HexNAc1	37.743	545.1955	545.19396	H+	0	0	0	38.406	0	0	0	0
Hex2HexNAc1	41.914	545.1955	545.19451	H+	0	0	0	41.937	0	0	0	13.619
Hex2HexNAc1	42.922	545.1955	545.19451	H+	0	0	0	0	0	0	0	29.421
Hex2HexNAc1	43.597	545.1955	545.19442	H+	0	0	0	29.490	0	0	0	0
Hex2HexNAc1	44.462	545.1955	545.19455	H+	0	0	0	66.997	0	0	0	0
Hex2HexNAc1	45.516	545.1955	545.19451	H+	0	0	0	11.131	0	0	0	0
Hex2Neu5Ac1	33.559	633.2115	633.2121	H+	133.021	0	1721.759	5356.232	1348.799	0	2165.603	3950.683
Hex2Neu5Ac1	36.285	633.2115	633.2115	H+	4395.956	0	7629.033	57805.957	2628.613	0	4349.560	41377.148
Hex2Neu5Ac1	38.372	633.2115	633.21181	H-	0	0	0	0	0	23.556	0	0
Hex2Neu5Ac1	39.265	633.2115	633.21168	H-	0	0	0	0	0	24.056	0	0
Hex2dHex2	29.169	634.2319	634.23004	H+	0	0	20.537	427.395	0	0	0	135.668
Hex2dHex2	31.532	634.2319	634.23119	H-	14.457	36.353	0	0	13.713	0	16.781	4931.706
Hex2dHex2	34.393	634.2319	634.23043	H+	2159.275	0	0	435.659	5783.113	0	4363.686	4113.217
Hex2dHex2	34.886	634.2319	634.23237	H-	2805.714	0	0	805.923	8115.135	0	4849.971	28144.947
Hex2dHex2	32.985	634.2319	634.23248	H-	0	15.631	0	0	0	0	0	47.750
Hex3dHex1	37.994	650.2268	650.22661	H-	31.957	0	0	0	0	13.282	651.691	0
Hex3dHex1	36.828	650.2268	650.22819	H-	0	0	0	37.610	0	0	0	895.199
Hex3dHex1	39.138	650.2268	650.22798	H-	0	0	0	18.274	0	0	0	51.465
Hex3dHex1	40.664	650.2268	650.22516	H+	0	0	0	0	0	0	0	18.226
Hex4	24.135	666.2217	666.21919	H+	5910.795	0	0	0	3361.263	0	3612.380	0
Hex4	39.385	666.2217	666.22274	H-	0	0	0	0	0	53.9491	0	0
Hex4	40.356	666.2217	666.22216	H-	0	0	0	0	0	0	0	16.358

Hex3dHex1	29.187	668.2374	668.23769	H-	8.458	0	42.045	756.861	33.886	0	9.461	237.105
Hex3dHex1	30.155	668.2374	668.23769	H-	11.425	0	61.742	961.067	44.609	0	10.787	209.410
Hex3dHex1	22.225	668.2374	668.23424	H+	0	81.199	0	0	0	0	0	0
Hex3dHex1	23.059	668.2374	668.23387	H+	0	0	0	5.109	0	0	0	0
Hex3dHex1	24.115	668.2374	668.23418	H+	0	13.332	0	0	0	0	0	0
Hex2HexNAc1dHex1	34.699	691.2534	691.25133	H-	51.664	0	0	0	0	24.797	0	0
Hex2HexNAc1dHex1	40.237	691.2534	691.25369	H-	24.533	0	0	0	16.759	0	43.834	398.952
Hex2HexNAc1dHex1	40.824	691.2534	691.25281	H-	60.046	0	276.529	1810.579	98.497	0	44.099	441.196
Hex2HexNAc1dHex1	42.235	691.2534	691.25355	H-	33.180	0	173.786	1574.509	139.559	0	10.643	102.350
Hex2HexNAc1dHex1	35.766	691.2534	691.25561	H+	0	0	0	0	0	0	0	152.627
Hex2HexNAc1dHex1	36.845	691.2534	691.25121	H+	0	0	0	179.525	0	0	0	0
Hex2HexNAc1dHex1	37.74	691.2534	691.25482	H-	0	0	0	422.143	0	0	0	0
Hex2HexNAc1dHex1	39.456	691.2534	691.25288	H-	0	0	0	0	0	7.883	0	0
Hex2HexNAc1dHex1	43.454	691.2534	691.25121	H+	0	0	0	6.652	0	0	0	0
Hex2HexNAc1dHex1	44.469	691.2534	691.25231	H+	0	0	0	29.446	0	0	0	0
Hex3HexNAc1	38.296	707.2483	707.2477	H+	9006.763	0	17654.915	178.260	5489.345	0	5093.748	29565.223
Hex3HexNAc1	40.242	707.2483	707.2468	H+	22.717	0	0		8.732	0	15.718	205.058
Hex3HexNAc1	40.752	707.2483	707.2464	H+	101.975	0	89.596	514.460	92.860	0	39.778	431.379
Hex3HexNAc1	41.916	707.2483	707.24669	H+	0	0	8.267		0	0	10.503	45.538
Hex3HexNAc1	44.317	707.2483	707.24651	H+	0	0	24.917	42.046	0	0	0	0
Hex3HexNAc1	45.252	707.2483	707.24675	H+	0	0	39.746	168.273	0	0	0	0
Hex3HexNAc1	43.482	707.2483	707.24694	H+	0	0	0	34.760	0	0	0	0
Hex2Neu5Ac1dHex1	38.938	779.2694	779.27001	H-	13.256	0	68.962	1067.626	43.787	0	23.331	142.055
Hex3dHex2	40.665	796.2847	796.28482	H-	29.824	46.287	0	0	27.817	0	113.368	1464.331
Hex3dHex2	39.261	796.2847	796.2881	H-	0	0	0	0	0	0	0	14.957
Hex2HexNAc1dHex2	42.092	837.3111	837.31117	H-	48.683	13.591	0	104.494	128.411	0	56.772	622.419

Hex2HexNAc1dHex2	43.34	837.3111	837.31212	H-	0	9.295	0	11.115	11.333	0	0	43.018
Hex2HexNAc1dHex2	41.653	837.3111	837.31196	H-	0	0	0	0	0	0	0	15.802
Hex3HexNAc1dHex1	40.243	853.3062	853.30667	H+	2557.166	411.169	90.197	389.645	2357.976	0	3896.360	18654.371
Hex3HexNAc1dHex1	42.093	853.3062	853.30306	H+	10.2854	0	0	0	23.122	0	10.300	118.443
Hex3HexNAc1dHex1	42.86	853.3062	853.30324	H+	5.764	0	13.747	26.030	0	0	0	34.128
Hex3HexNAc1dHex1	43.579	853.3062	853.30344	H+	4.752	0	0	0	0	0	4.696	63.676
Hex3HexNAc1dHex1	44.74	853.3062	853.30301	H+	16.164	0	0	0	13.333	0	28.463	314.558
Hex4HexNAc1	41.021	869.3011	869.30053	H-	12.955	0	25.542	111.194	0	22.747	23.851	954.340
Hex3HexNAc2	42.715	910.3277	910.32561	H+	0	0	0	0	0	0	10.128	83.045
Hex3HexNAc2	40.372	910.3277	910.32608	H+	0	0	0	366.459	0	16.670	0	1888.414
Hex3HexNAc2	41.61	910.3277	910.32494	H+	0	0	0	65.172	0	0	0	0
Hex3HexNAc1Neu5Ac1	40.979	998.3437	998.3434	H+	212.144	0	444.027	10258.056	342.444	0	130.877	5445.071
Hex3HexNAc1Neu5Ac1	43.884	998.3437	998.34099	H+	8.605	0	18.275	142.587	4.928	0	6.279	75.676
Hex3HexNAc1Neu5Ac1	43.225	998.3437	998.34092	H+	0	0	0	40.822	0	0	0	19.547
Hex3HexNAc1Neu5Ac1	45.721	998.3437	998.3402	H+	0	0	0	31.400	0	0	0	0
Hex3HexNAc1dHex2	42.095	999.3641	999.36352	H-	4689.819	1553.738	684.564	9809.400	10153.034	0	5085.320	43908.025
Hex3HexNAc1dHex2	41.099	999.3641	999.36003	H+	0	8.807	0	0	0	0	0	0
Hex3HexNAc1dHex2	44.776	999.3641	999.36022	H+	0	0	0	0	0	0	0	25.081
Hex2HexNAc1Neu5Ac1dHex1	42.195	1000.3594	1000.36518	H+	0	0	62.385	631.308	0	0	0	0
Hex4HexNAc1dHex1	41.513	1015.359	1015.35942	H+	0	0	0	0	0	0	17.305	104.093
Hex4HexNAc1dHex1	42.329	1015.359	1015.35874	H+	89.167	0	120.426	147.276	33.337	0	25.334	388.117
Hex4HexNAc1dHex1	43.967	1015.359	1015.35749	H+	0	0	0	12.144	0	0	0	0
Hex1HexNAc2Neu5Ac1dHex1	43.803	1023.3754	1023.38042	H+	5.115	0	0	0	0	0	0	0
Hex3HexNAc2dHex1	42.799	1056.3856	1056.38349	H+	0	0	6.570	0	0	0	0	0
Hex3HexNAc2dHex1	41.88	1056.3856	1056.38402	H+	0	10.638	0	915.234	0	0	0	1573.072
Hex3HexNAc2dHex1	43.585	1056.3856	1056.38393	H+	0	0	0	0	0	0	0	32.267

Hex3HexNAc2dHex1	44.472	1056.3856	1056.3826	H+	0	0	0	30.799	0	0	0	0
Hex4HexNAc2	38.303	1072.3805	1072.3781	H+	0	0	20.762	0	0	0	0	36.186
Hex4HexNAc2	41.899	1072.3805	1072.37975	H+	366.697	0	1049.638	1382.189	247.063	0	1428.476	3484.694
Hex4HexNAc2	43.593	1072.3805	1072.37749	H+	0	0	0	0	0	0	0	8.641
Hex4HexNAc2	45.193	1072.3805	1072.37894	H+	0	0	0	31.406	0	0	0	37.409
Hex3HexNAc1Neu5Ac1dHex1	41.842	1144.4016	1144.40098	H-	12.211	0	0	0	21.720	0	20.308	0
Hex3HexNAc1Neu5Ac1dHex1	42.346	1144.4016	1144.40018	H+	0	0	29.429	0	20.164	0	0	0
Hex3HexNAc1Neu5Ac1dHex1	43.826	1144.4016	1144.39739	H+	0	0	0	0	0	0	0	6.963
Hex3HexNAc1Neu5Ac1dHex1	44.921	1144.4016	1144.39958	H+	0	0	0	6.246	0	0	0	0
Hex4HexNAc1dHex2	42.586	1161.4169	1161.41713	H-	15.305	0	0	0	9.607	0	13.710	342.135
Hex4HexNAc1dHex2	43.411	1161.4169	1161.41806	H-	0	18.246	0	0	0	0	7.135	267.490
Hex3HexNAc2dHex2	43.971	1202.4433	1202.44054	H+	0	0	6.652	36.928	0	0	0	6.170
Hex3HexNAc2dHex2	42.938	1202.4433	1202.44326	H-	0	49.872	0	104.196	0	0	0	1638.071
Hex3HexNAc2dHex2	44.878	1202.4433	1202.44281	H-	0	0	0	37.196	0	0	0	11.084
Hex3HexNAc2dHex2	44.132	1202.4433	1202.44371	H-	0	0	0	117.257	0	0	0	0
Hex4HexNAc2dHex1	42.856	1218.4384	1218.43699	H+	1595.457	0	4459.341	5845.568	479.162	0	1155.245	9281.284
Hex4HexNAc2dHex1	43.946	1218.4384	1218.43641	H+	0	0	16.227	147.810	0	0	0	0
Hex4HexNAc2dHex1	45.211	1218.4384	1218.437	H+	0	0	22.892	0	0	0	0	0
Hex3HexNAc2Neu5Ac1	42.883	1219.4337	1219.43784	H+	0	0	3.962	0	0	0	0	0
Hex3HexNAc1Neu5Ac2	42.201	1289.4391	1289.43493	H+	212.976	0	321.325	82.162	0	0	48.268	123.410
Hex3HexNAc1Neu5Ac2	43.918	1289.4391	1289.43795	H+	0	0	0	31.001	0	0	0	28.782
Hex4HexNAc1Neu5Ac1dHex1	42.188	1306.4543	1306.46194	H+	0	0	6.624	401.445	0	0	0	0
Hex4HexNAc2Neu5Ac1	43.075	1363.4758	1363.47552	H-	194.007	0	264.706	1551.842	75.920	0	391.279	2587.927
Hex4HexNAc2Neu5Ac1	43.817	1363.4758	1363.47222	H+	0	0	0	114.184	8.362	0	0	82.145
Hex4HexNAc2Neu5Ac1	44.997	1363.4758	1363.47392	H+	0	0	0	15.614	0	0	0	0
Hex4HexNAc2dHex2	43.58	1364.4963	1364.49542	H-	376.237	0	0	0	13.489	0	364.288	4088.065

Hex4HexNAc2dHex2	43.954	1364.4963	1364.49415	H+	1333.441	0	3633.410	6480.815	1208.225	0	456.260	8439.879
Hex4HexNAc2dHex2	40.825	1364.4963	1364.49286	H+	0	0	0	104.265	0	0	0	18.889
Hex4HexNAc2dHex2	44.739	1364.4963	1364.49314	H+	0	0	0	0	0	0	0	22.573
Hex6dHex3	43.963	1428.501	1428.49641	H-	0	0	34.756	0	0	0	0	0
Hex3HexNAc1Neu5Ac2dHex1	42.909	1435.4969	1435.49853	H-	16.568	0	128.159	923.017	53.660	0	18.029	136.613
Hex5HexNAc3	43.569	1437.5127	1437.51257	H-	40.2071	0	37.351	173.317	11.314	0	59.080	395.084
Hex5HexNAc3	45.577	1437.5127	1437.50835	H+	0	0	7.459	0	0	0	0	0
Hex5HexNAc3	44.433	1437.5127	1437.5101	H+	0	0	0	16.896	0	0	0	0
Hex4HexNAc4	44.73	1496.5499	1496.54102	H-	0	0	0	0	0	0	6.229	65.614
Hex4HexNAc2Neu5Ac1dHex1	43.275	1509.5338	1509.53001	H-	0	0	37.397	732.137	0	0	0	258.175
Hex4HexNAc2Neu5Ac1dHex1	43.82	1509.5338	1509.53671	H-	854.357	0	1340.873	3841.486	516.304	0	644.835	2626.177
Hex4HexNAc2dHex3	44.729	1510.5542	1510.55067	H-	293.820	0	0	35.297	298.266	0	416.892	2887.741
Hex5HexNAc2dHex2	43.841	1526.5491	1526.55575	H+	0	0	41.519	443.946	0	0	0	32.175
Hex5HexNAc2dHex2	45.024	1526.5491	1526.55125	H-	0	0	8.647	37.382	0	0	0	24.892
Hex5HexNAc3dHex1	44.444	1583.5706	1583.57076	H+	76.444	0	185.626	1681.867	69.565	0	64.810	557.505
Hex5HexNAc3dHex1	45.277	1583.5706	1583.56835	H+	0	0	0	78.421	0	0	0	0
Hex5HexNAc3dHex1	46.151	1583.5706	1583.56841	H+	0	0	0	40.888	0	0	0	0
Hex4HexNAc2Neu5Ac2	43.472	1654.5713	1654.57215	H-	31.293	0	44.107	604.674	13.799	0	105.601	453.991
Hex4HexNAc2Neu5Ac2	43.959	1654.5713	1654.57104	H+	78.759	0	121.851	1161.557	0	0	113.780	952.643
Hex4HexNAc2Neu5Ac1dHex2	44.23	1655.5917	1655.59403	H-	14.526	0	0	91.201	16.953	0	37.627	525.667
Hex9HexNAc1	44.351	1697.5757	1697.56582	H-	0	0	12.264	0	0	0	0	0
Hex5HexNAc3Neu5Ac1	44.299	1728.6082	1728.60913	H-	12.499	0	14.880	228.660	8.974	0	18.276	243.893
Hex5HexNAc3Neu5Ac1	44.064	1728.6082	1728.60483	H+	0	0	0	0	0	0	0	30.051
Hex5HexNAc3dHex2	45.215	1729.6285	1729.63153	H-	121.294	0	291.727	2382.338	93.888	0	52.827	523.968
Hex5HexNAc3dHex2	46.05	1729.6285	1729.62656	H+	0	0	0	24.304	0	0	0	0
Hex5HexNAc3dHex2	46.761	1729.6285	1729.62834	H+	0	0	0	13.802	0	0	0	0

Hex4HexNAc4dHex2	45.265	1770.6552	1770.65055	H+	0	0	7.069	39.950	0	0	0	0
Hex6HexNAc4	44.787	1802.6449	1802.6428	H+	29.438	0	82.340	68.414	0	0	44.578	133.586
Hex5HexNAc3Neu5Ac1dHex1	44.971	1874.666	1874.66852	H-	24.658	0	52.972	0	16.742	0	18.292	246.360
Hex5HexNAc3dHex3	45.991	1875.6864	1875.68748	H-	0	0	113.232	0	61.621	0	0	43.234
Hex4HexNAc2Neu5Ac2dHex2	44.264	1946.6871	1946.68496	H-	7.283	0	0	0	0	0	26.761	0
Hex6HexNAc4dHex1	45.502	1948.7028	1948.70085	H-	16.332	0	160.840	143.215	0	0	20.624	0
Hex6HexNAc4dHex1	46.177	1948.7028	1948.69895	H+	0	0	0	15.475	0	0	0	0
Hex5HexNAc4Neu5Ac1	45.313	1949.6981	1949.70139	H+	0	0	0	0	0	0	13.049	0
Hex5HexNAc4dHex2	45.569	1950.7185	1950.71109	H+	0	0	0	0	0	0	8.954	0
Hex10HexNAc2	45.563	2062.7079	2062.69763	H-	0	0	12.300	0	0	0	0	30.860
Hex6HexNAc4Neu5Ac1	45.326	2093.7404	2093.74274	H-	0	0	10.676	0	0	0	6.792	81.652
Hex6HexNAc4dHex2	46.159	2094.7607	2094.76709	H-	26.438	0	963.624	566.721	20.764	0	30.320	293.650
Hex3HexNAc8	46.165	2128.8042	2128.80977	H-	0	0	38.921	283.511	0	0	8.639	112.336
Hex3HexNAc8	44.857	2128.8042	2128.80874	H+	0	0	0	5.905	0	0	0	0
Hex5HexNAc3Neu5Ac2dHex1	44.926	2165.7615	2165.76479	H-	0	0	38.365	325.342	0	0	10.110	0
Hex6HexNAc4Neu5Ac1dHex1	45.856	2239.7981	2239.79717	H-	0	0	24.392	0	0	0	0	42.305
Hex6HexNAc4Neu5Ac1dHex1	45.458	2239.7981	2239.8041	H-	0	0	0	52.851	0	0	0	0
Hex6HexNAc4dHex3	46.817	2240.8186	2240.82457	H-	15.888	0	37.859	435.501	17.370	0	0	106.066
Hex5HexNAc4Neu5Ac1dHex2	46.827	2241.814	2241.82581	H-	13.480	0	30.034	313.107	9.290	0	0	0
