# Supplementary material for: Comparative Analysis of Oligosaccharides in Breast Milk and Feces of Breast-Fed Infants by Using LC-QE-HF-MS: A Communication
Source: Nutrients. 2023 Feb 9;15(4):888. doi: 10.3390/nu15040888 (PMC9963387; doi:10.3390/nu15040888)
Supplement: Supplementary file 1 [file nutrients-15-00888-s001.zip › Supplementary Table S2.pdf]

Supplementary Table S2 The composition and their peak area of OS only identified in feces of four women's babies

| Composition | RT (min) | Mass | | Ion | Peak area (×10$^6$) | | | |
|---|---|---|---|---|---|---|---|---|
| | | Found | Cal | | F1 | F2 | F3 | F4 |
| Hex1HexNAc1dHex1 | 19.649 | 547.2112 | 547.2112 | H- | 0 | 0 | 42.384 | 0 |
| Hex1HexNAc1dHex1 | 21.385 | 547.2112 | 547.2112 | H- | 0 | 0 | 84.675 | 0 |
| Hex1HexNAc1dHex1 | 22.756 | 547.2112 | 547.2112 | H- | 0 | 0 | 58.600 | 0 |
| Hex2HexNAc1 | 23.202 | 563.2061 | 563.20619 | H- | 0 | 0 | 14.816 | 0 |
| Hex2Neu5Ac1 | 13.612 | 651.2221 | 651.22118 | H+ | 0 | 0 | 6.580 | 0 |
| Hex1HexNAc1Neu5Ac1 | 31.704 | 674.2381 | 674.24043 | H- | 0 | 232.227 | 0 | 0 |
| Hex1HexNAc1Neu5Ac1 | 32.65 | 674.2381 | 674.23906 | H+ | 0 | 239.684 | 0 | 244.460 |
| Hex1HexNAc1Neu5Ac1 | 33.592 | 674.2381 | 674.23625 | H+ | 0 | 147.452 | 0 | 0 |
| Hex1HexNAc1dHex2 | 31.17 | 675.2585 | 675.25807 | H+ | 0 | 0 | 0 | 272.790 |
| Hex1HexNAc1dHex2 | 32.523 | 675.2585 | 675.25817 | H+ | 0 | 0 | 0 | 7.035 |
| Hex1HexNAc1dHex2 | 34.535 | 675.2585 | 675.25568 | H+ | 38.937 | 0 | 0 | 0 |
| Hex1HexNAc1dHex2 | 37.447 | 675.2585 | 675.25565 | H+ | 0 | 0 | 832.727 | 0 |
| Hex1HexNAc1Neu5Ac1 | 2.27 | 692.2487 | 692.25136 | H- | 21.914 | 0 | 0 | 0 |
| Hex2HexNAc1dHex1 | 29.642 | 709.264 | 709.26448 | H- | 0 | 0 | 12.691 | 0 |
| Hex2HexNAc1dHex1 | 30.116 | 709.264 | 709.26448 | H- | 0 | 0 | 14.131 | 0 |
| Hex1HexNAc2dHex1 | 40.655 | 732.28 | 732.27949 | H- | 0 | 9.875 | 0 | 0 |
| Hex2HexNAc2 | 37.259 | 748.2749 | 748.27496 | H- | 0 | 449.043 | 80.571 | 149.341 |
| Hex2HexNAc2 | 40.169 | 748.2749 | 748.27416 | H+ | 0 | 0 | 9.944 | 0 |
| Hex2dHex3 | 34.895 | 780.2898 | 780.28661 | H+ | 0 | 0 | 0 | 9.008 |
| Hex2dHex3 | 37.993 | 780.2898 | 780.28833 | H- | 6.389 | 0 | 0 | 237.661 |
| Hex2dHex3 | 39.3 | 780.2898 | 780.28679 | H+ | 7.297 | 0 | 0 | 0 |
| Hex1HexNAc3 | 33.303 | 789.3015 | 789.30232 | H- | 0 | 0 | 0 | 19.283 |
| Hex1HexNAc3 | 34.913 | 789.3015 | 789.30177 | H- | 0 | 0 | 0 | 49.435 |

| | | | | | | | | |
|---|---|---|---|---|---|---|---|---|
| Hex3Neu5Ac1 | 40.148 | 795.2643 | 795.26426 | H- | 0 | 72.415 | 0 | 90.911 |
| Hex2Neu5Ac1dHex1 | 25.732 | 797.28 | 797.28108 | H- | 0 | 0 | 0 | 29.004 |
| Hex2Neu5Ac1dHex1 | 30.144 | 797.28 | 797.28177 | H- | 0 | 131.385 | 0 | 265.206 |
| Hex2Neu5Ac1dHex1 | 38.3 | 797.28 | 797.28065 | H- | 0 | 0 | 0 | 21.183 |
| Hex2HexNAc1Neu5Ac1 | 39.401 | 836.2909 | 836.29242 | H- | 0 | 279.781 | 0 | 203.853 |
| Hex2HexNAc1Neu5Ac1 | 42.204 | 836.2909 | 836.28863 | H+ | 0 | 21.845 | 0 | 0 |
| Hex1HexNAc1Neu5Ac1dHex1 | 26.481 | 838.3066 | 838.30702 | H- | 0 | 0 | 0 | 10.764 |
| Hex1HexNAc1Neu5Gc1dHex1 | 30.096 | 854.3015 | 854.30214 | H- | 0 | 0 | 11.314 | 0 |
| Hex2HexNAc1Neu5Gc1 | 41.038 | 870.2964 | 870.29595 | H+ | 0 | 0 | 0 | 20.073 |
| Hex2HexNAc2dHex1 | 26.486 | 894.3328 | 894.33284 | H- | 0 | 18.209 | 0 | 0 |
| Hex2HexNAc2dHex1 | 40.203 | 894.3328 | 894.33231 | H+ | 0 | 107.398 | 0 | 0 |
| Hex2HexNAc2dHex1 | 41.602 | 894.3328 | 894.33336 | H- | 0 | 96.156 | 0 | 10.063 |
| Hex2Neu5Ac2 | 36.372 | 942.3175 | 942.31996 | H- | 0 | 0 | 0 | 16.821 |
| Hex2HexNAc3 | 39.239 | 951.3543 | 951.35124 | H+ | 0 | 0 | 151.148 | 0 |
| Hex4dHex2 | 30.134 | 958.3375 | 958.33569 | H+ | 0 | 0 | 0 | 31.443 |
| Hex4dHex2 | 40.224 | 958.3375 | 958.33601 | H- | 0 | 8.792 | 0 | 0 |
| Hex1HexNAc1Neu5Ac1Neu5Gc1 | 41.321 | 981.3284 | 981.33165 | H+ | 0 | 13.039 | 0 | 0 |
| Hex1HexNAc1Neu5Ac1Neu5Gc1 | 44.94 | 999.339 | 999.3449 | H+ | 0 | 11.839 | 0 | 0 |
| Hex3HexNAc1dHex2 | 28.106 | 1017.3747 | 1017.37471 | H- | 0 | 13.587 | 0 | 0 |
| Hex5HexNAc1 | 45.438 | 1031.3539 | 1031.3492 | H- | 0 | 41.673 | 0 | 0 |
| Hex4HexNAc1dHex1 | 29.619 | 1033.3696 | 1033.37019 | H- | 0 | 41.267 | 0 | 0 |
| Hex2HexNAc2Neu5Ac1 | 40.348 | 1039.3702 | 1039.37153 | H- | 0 | 9.908 | 0 | 0 |
| Hex2HexNAc2dHex2 | 42.926 | 1040.3907 | 1040.39183 | H- | 0 | 0 | 0 | 25.700 |
| Hex2HexNAc2dHex2 | 40.974 | 1040.3905 | 1040.38997 | H- | 0 | 0 | 0 | 7.838 |
| Hex2HexNAc2Neu5Gc1 | 44.338 | 1073.3759 | 1073.38091 | H+ | 0 | 7.724 | 0 | 0 |
| Hex2HexNAc3dHex1 | 41.14 | 1097.4122 | 1097.40728 | H+ | 0 | 74.693 | 0 | 0 |

| | | | | | | | | | |
|---|---|---|---|---|---|---|---|---|---|
| Hex3HexNAc3 | 41.317 | 1113.4071 | 1113.40572 | H- | 0 | 41.832 | 253.399 | 0 |
| Hex1HexNAc4dHex1 | 30.703 | 1138.4388 | 1138.43205 | H+ | 0 | 0 | 86.906 | 0 |
| Hex4HexNAc1Neu5Ac1 | 42.361 | 1160.3965 | 1160.39656 | H- | 0 | 111.273 | 0 | 38.745 |
| Hex1HexNAc2Neu5Ac1dHex2 | 46.343 | 1169.4333 | 1169.43547 | H+ | 0 | 0 | 0 | 4.774 |
| Hex5HexNAc1dHex1 | 42.769 | 1177.4117 | 1177.4134 | H- | 0 | 12.978 | 0 | 0 |
| Hex2HexNAc1Neu5Gc2 | 42.436 | 1177.3867 | 1177.38706 | H+ | 0 | 0 | 0 | 8.715 |
| Hex3HexNAc2Neu5Ac1 | 42.044 | 1201.4231 | 1201.42261 | H- | 0 | 614.642 | 0 | 415.988 |
| Hex3HexNAc2Neu5Ac1 | 43.931 | 1201.4231 | 1201.42026 | H+ | 0 | 9.962 | 0 | 0 |
| Hex5HexNAc2 | 43.199 | 1234.4333 | 1234.43176 | H- | 0 | 0 | 0 | 43.321 |
| Hex4Neu5Ac2 | 43.09 | 1248.4125 | 1248.4125 | H- | 0 | 5.561 | 0 | 0 |
| Hex5HexNAc2 | 41.942 | 1252.4439 | 1252.44522 | H- | 0 | 14.348 | 0 | 0 |
| Hex3HexNAc3dHex1 | 42.446 | 1259.465 | 1259.46494 | H- | 0 | 201.261 | 0 | 0 |
| Hex3HexNAc3dHex1 | 43.621 | 1259.465 | 1259.46577 | H- | 0 | 14.572 | 0 | 0 |
| Hex4HexNAc3 | 42.521 | 1275.46 | 1275.4603 | H- | 0 | 425.240 | 12.218 | 79.109 |
| Hex3HexNAc2Neu5Ac1dHex1 | 42.323 | 1347.481 | 1347.48026 | H- | 0 | 117.933 | 0 | 21.534 |
| Hex3HexNAc2Neu5Ac1dHex1 | 43.287 | 1347.481 | 1347.47971 | H+ | 0 | 15.380 | 0 | 0 |
| Hex3HexNAc2dHex3 | 44.749 | 1348.5014 | 1348.50112 | H- | 0 | 0 | 0 | 15.482 |
| Hex3HexNAc2Neu5Ac1dHex1 | 44.727 | 1365.4916 | 1365.4987 | H+ | 0 | 0 | 0 | 8.856 |
| Hex5HexNAc2dHex1 | 44.068 | 1380.4912 | 1380.49403 | H- | 0 | 46.099 | 0 | 50.748 |
| Hex2HexNAc3dHex3 | 20.137 | 1389.528 | 1389.52524 | H+ | 0 | 12.576 | 0 | 0 |
| Hex3HexNAc3dHex2 | 43.479 | 1405.5229 | 1405.52117 | H+ | 0 | 49.125 | 0 | 0 |
| Hex3HexNAc3dHex2 | 44.462 | 1405.5229 | 1405.52327 | H- | 0 | 9.932 | 0 | 0 |
| Hex4HexNAc3dHex1 | 43.448 | 1421.5178 | 1421.51744 | H+ | 0 | 331.369 | 0 | 0 |
| Hex4HexNAc3dHex1 | 45.518 | 1421.5178 | 1421.51255 | H+ | 0 | 26.423 | 0 | 0 |
| Hex4HexNAc1Neu5Ac2 | 43.246 | 1451.4919 | 1451.49198 | H- | 0 | 35.218 | 0 | 7.800 |
| Hex4HexNAc4 | 43.148 | 1478.5393 | 1478.53904 | H- | 0 | 24.043 | 0 | 0 |

| | | | | | | | | |
|---|---|---|---|---|---|---|---|---|
| Hex4HexNAc4 | 44.734 | 1478.5393 | 1478.53115 | H- | 0 | 0 | 0 | 8.986 |
| Hex4HexNAc1Neu5Gc2 | 38.302 | 1483.4817 | 1483.48714 | H- | 0 | 0 | 0 | 9.305 |
| Hex3HexNAc2Neu5Ac2 | 42.6 | 1492.5186 | 1492.52311 | H- | 0 | 288.340 | 0 | 63.542 |
| Hex3HexNAc2Neu5Ac2 | 43.852 | 1492.5186 | 1492.51865 | H+ | 0 | 7.319 | 0 | 0 |
| Hex3HexNAc2Neu5Ac1dHex2 | 44.741 | 1511.5495 | 1511.55271 | H+ | 0 | 0 | 0 | 34.006 |
| Hex3HexNAc5 | 42.664 | 1519.5659 | 1519.56235 | H+ | 0 | 0 | 27.200 | 0 |
| Hex5HexNAc2Neu5Ac1 | 43.757 | 1525.5287 | 1525.52925 | H- | 0 | 16.643 | 0 | 12.523 |
| Hex4HexNAc2dHex3 | 43.281 | 1528.5648 | 1528.56028 | H+ | 0 | 9.588 | 0 | 0 |
| Hex2HexNAc3Neu5Ac2 | 44.896 | 1533.545 | 1533.53843 | H+ | 0 | 0 | 0 | 4.951 |
| Hex3HexNAc3Neu5Ac1dHex1 | 43.4 | 1550.5604 | 1550.56631 | H- | 0 | 9.443 | 0 | 0 |
| Hex4HexNAc3Neu5Ac1 | 43.263 | 1566.5553 | 1566.55179 | H- | 0 | 79.536 | 0 | 11.540 |
| Hex4HexNAc3Neu5Ac1 | 44.469 | 1566.5553 | 1566.56003 | H+ | 0 | 86.127 | 0 | 0 |
| Hex4HexNAc3dHex2 | 44.468 | 1567.5757 | 1567.57566 | H- | 0 | 1048.744 | 0 | 10.452 |
| Hex2HexNAc3Neu5Ac1Neu5Gc1 | 43.26 | 1567.5507 | 1567.55791 | H+ | 0 | 8.426 | 0 | 0 |
| Hex4HexNAc3dHex2 | 44.464 | 1585.5863 | 1585.59177 | H+ | 0 | 68.808 | 0 | 0 |
| Hex4HexNAc4dHex1 | 44.137 | 1624.5972 | 1624.59782 | H- | 0 | 92.793 | 0 | 0 |
| Hex5HexNAc4 | 44.046 | 1640.592 | 1640.59165 | H- | 0 | 17.315 | 0 | 79.158 |
| Hex3HexNAc2Neu5Ac1dHex3 | 43.708 | 1657.6074 | 1657.59866 | H- | 0 | 0 | 0 | 11.822 |
| Hex3HexNAc5dHex1 | 42.564 | 1665.6238 | 1665.62033 | H+ | 0 | 0 | 7.176 | 0 |
| Hex4HexNAc5 | 45.717 | 1681.6187 | 1681.61899 | H- | 0 | 83.046 | 0 | 0 |
| Hex4HexNAc3Neu5Ac1dHex1 | 44.246 | 1712.6132 | 1712.61432 | H- | 0 | 53.556 | 0 | 0 |
| Hex3HexNAc6 | 43.045 | 1722.6453 | 1722.64072 | H+ | 0 | 0 | 9.898 | 0 |
| Hex6HexNAc3dHex1 | 44.279 | 1745.6234 | 1745.63207 | H+ | 0 | 13.790 | 0 | 11.417 |
| Hex4HexNAc4Neu5Ac1 | 43.6 | 1769.6346 | 1769.63377 | H- | 0 | 39.893 | 0 | 0 |
| Hex5HexNAc4dHex1 | 44.814 | 1786.65 | 1786.65066 | H- | 0 | 406.790 | 0 | 37.529 |
| Hex2HexNAc1Neu5Gc4 | 45.194 | 1791.5673 | 1791.56015 | H+ | 0 | 27.103 | 0 | 0 |

| | | | | | | | | |
|---|---|---|---|---|---|---|---|---|
| Hex3HexNAc2Neu5Ac1dHex4 | 44.896 | 1803.6653 | 1803.67246 | H+ | 0 | 50.890 | 0 | 0 |
| Hex4HexNAc3Neu5Ac2 | 43.812 | 1857.6508 | 1857.65038 | H+ | 0 | 50.237 | 0 | 0 |
| Hex5HexNAc5 | 46.052 | 1861.6821 | 1861.67715 | H- | 0 | 11.839 | 0 | 0 |
| Hex3HexNAc6dHex1 | 44.831 | 1868.7032 | 1868.71035 | H- | 0 | 4.720 | 0 | 0 |
| Hex4HexNAc6 | 45.859 | 1884.6981 | 1884.6959 | H+ | 0 | 12.977 | 0 | 0 |
| Hex2HexNAc4Neu5Ac2dHex1 | 45.833 | 1900.693 | 1900.69272 | H- | 0 | 0 | 0 | 59.910 |
| Hex8HexNAc3 | 44.958 | 1923.6711 | 1923.67971 | H- | 0 | 70.971 | 0 | 0 |
| Hex5HexNAc4Neu5Ac1 | 44.692 | 1931.6875 | 1931.68674 | H+ | 0 | 0 | 0 | 10.964 |
| Hex5HexNAc4dHex2 | 45.514 | 1932.7079 | 1932.7053 | H+ | 0 | 0 | 0 | 20.227 |
| Hex5HexNAc3Neu5Ac2 | 44.73 | 2019.7035 | 2019.70576 | H- | 0 | 382.077 | 0 | 0 |
| Hex5HexNAc3Neu5Ac1dHex2 | 45.327 | 2020.7239 | 2020.72275 | H+ | 0 | 69.863 | 0 | 0 |
| Hex5HexNAc3Neu5Ac2 | 45.708 | 2037.7141 | 2037.72378 | H- | 0 | 21.076 | 0 | 0 |
| Hex2HexNAc4Neu5Ac3 | 46.005 | 2045.7303 | 2045.73477 | H- | 0 | 0 | 0 | 12.824 |
| Hex5HexNAc6 | 46.469 | 2046.7509 | 2046.7565 | H- | 0 | 0 | 0 | 6.288 |
| Hex7HexNAc3dHex2 | 45.706 | 2071.7446 | 2071.74063 | H- | 0 | 8.333 | 0 | 0 |
| Hex5HexNAc4Neu5Ac1dHex1 | 44.947 | 2077.7454 | 2077.74131 | H+ | 0 | 24.927 | 0 | 3.561 |
| Hex5HexNAc4dHex3 | 46.265 | 2078.7659 | 2078.7731 | H- | 0 | 12.280 | 0 | 0 |
| Hex5HexNAc4Neu5Ac1dHex1 | 45.335 | 2095.7561 | 2095.74831 | H- | 0 | 0 | 0 | 40.010 |
| Hex7HexNAc4dHex1 | 45.31 | 2110.7556 | 2110.76401 | H+ | 0 | 0 | 0 | 12.537 |
| Hex6HexNAc5dHex1 | 45.777 | 2151.7822 | 2151.78579 | H- | 0 | 21.309 | 0 | 0 |
| Hex5HexNAc3Neu5Ac1dHex3 | 45.856 | 2166.7818 | 2166.78347 | H- | 0 | 0 | 0 | 53.354 |
| Hex7HexNAc5 | 45.722 | 2167.7771 | 2167.77752 | H- | 0 | 21.030 | 0 | 14.045 |
| Hex5HexNAc5Neu5Gc1 | 45.824 | 2168.7725 | 2168.78525 | H+ | 0 | 0 | 0 | 11.828 |
| Hex3HexNAc4Neu5Ac3 | 46.184 | 2189.7727 | 2189.78457 | H- | 0 | 0 | 0 | 25.562 |
| Hex5HexNAc6dHex1 | 47.052 | 2192.8088 | 2192.81156 | H- | 0 | 16.575 | 0 | 0 |
| Hex4HexNAc6dHex2 | 47.043 | 2194.8245 | 2194.81973 | H- | 0 | 4.176 | 0 | 0 |

| | | | | | | | | |
|---|---|---|---|---|---|---|---|---|
| Hex5HexNAc4Neu5Ac2 | 44.824 | 2222.783 | 2222.78565 | H- | 0 | 104.510 | 0 | 17.489 |
| Hex5HexNAc4Neu5Gc2 | 46.149 | 2254.7727 | 2254.75933 | H- | 0 | 18.650 | 0 | 20.551 |
| Hex4HexNAc5Neu5Ac2 | 46.822 | 2263.8096 | 2263.81094 | H- | 0 | 16.995 | 0 | 0 |
| Hex3HexNAc8dHex1 | 46.582 | 2274.8621 | 2274.86997 | H+ | 0 | 0 | 0 | 59.939 |
| Hex6HexNAc5dHex2 | 46.438 | 2297.8401 | 2297.84317 | H- | 0 | 32.518 | 0 | 0 |
| Hex7HexNAc5dHex1 | 46.357 | 2313.835 | 2313.8398 | H- | 0 | 66.931 | 0 | 0 |
